# DNA barcoding of medicinal orchids in Asia

**DOI:** 10.1038/s41598-021-03025-0

**Published:** 2021-12-08

**Authors:** Bhakta Bahadur Raskoti, Rita Ale

**Affiliations:** Biodiversity Conservation Initiative, Nepalgunj, Banke Nepal

**Keywords:** Plant molecular biology, PCR-based techniques

## Abstract

Growing popularity of herbal medicine has increased the demand of medicinal orchids in the global markets leading to their overharvesting from natural habitats for illegal trade. To stop such illegal trade, the correct identification of orchid species from their traded products is a foremost requirement. Different species of medicinal orchids are traded as their dried or fresh parts (tubers, pseudobulbs, stems), which look similar to each other making it almost impossible to identify them merely based on morphological observation. To overcome this problem, DNA barcoding could be an important method for accurate identification of medicinal orchids. Therefore, this research evaluated DNA barcoding of medicinal orchids in Asia where illegal trade of medicinal orchids has long existed. Based on genetic distance, similarity-based and tree-based methods with sampling nearly 7,000 sequences from five single barcodes (ITS, ITS2, *matK*, *rbcL*, *trnH-psbA* and their seven combinations), this study revealed that DNA barcoding is effective for identifying medicinal orchids. Among single locus, ITS performed the best barcode, whereas ITS + *matK* exhibited the most efficient barcode among multi-loci. A barcode library as a resource for identifying medicinal orchids has been established which contains about 7,000 sequences of 380 species (i.e. 90%) of medicinal orchids in Asia.

## Introduction

Orchids are significant sources of secondary metabolites (e.g. alkaloid, flavonoid, terpenoid etc.) with chemical compounds (phytochemicals) such as Moscatin, Erianin, Gastrodin^1,2^. These phytochemicals have numerous medicinal properties that are important for human healthcare. For example, Goodyerin isolated from *Goodyera schlectendaliana*^3^ has sedative and anticonvulsant activities^4^; Gastrodin found in *Gastrodia elata*^5^ is effective in variety of neurological disease^6^; Dendrobine extracted from *Dendrobium nobile*^7^ is effective for influenza A virus^8^. Indeed, orchids were earlier considered more for their medicinal values rather than their beauty of colorful flowers and have long been used as medicine in different parts of the world^2,9–11^. Although the medicinal usage of orchids was apparently first recognized in the twenty-eighth century BC^9,12^, the history of medicinal usages of orchids dates back to the seventeenth century based on the official literature such as “Chinese Pharmacopeia”^13,14^. Presently, there are about 600 species of orchids that are widely used for traditional medicine in different parts of the world^11^.

**Figure 1:**
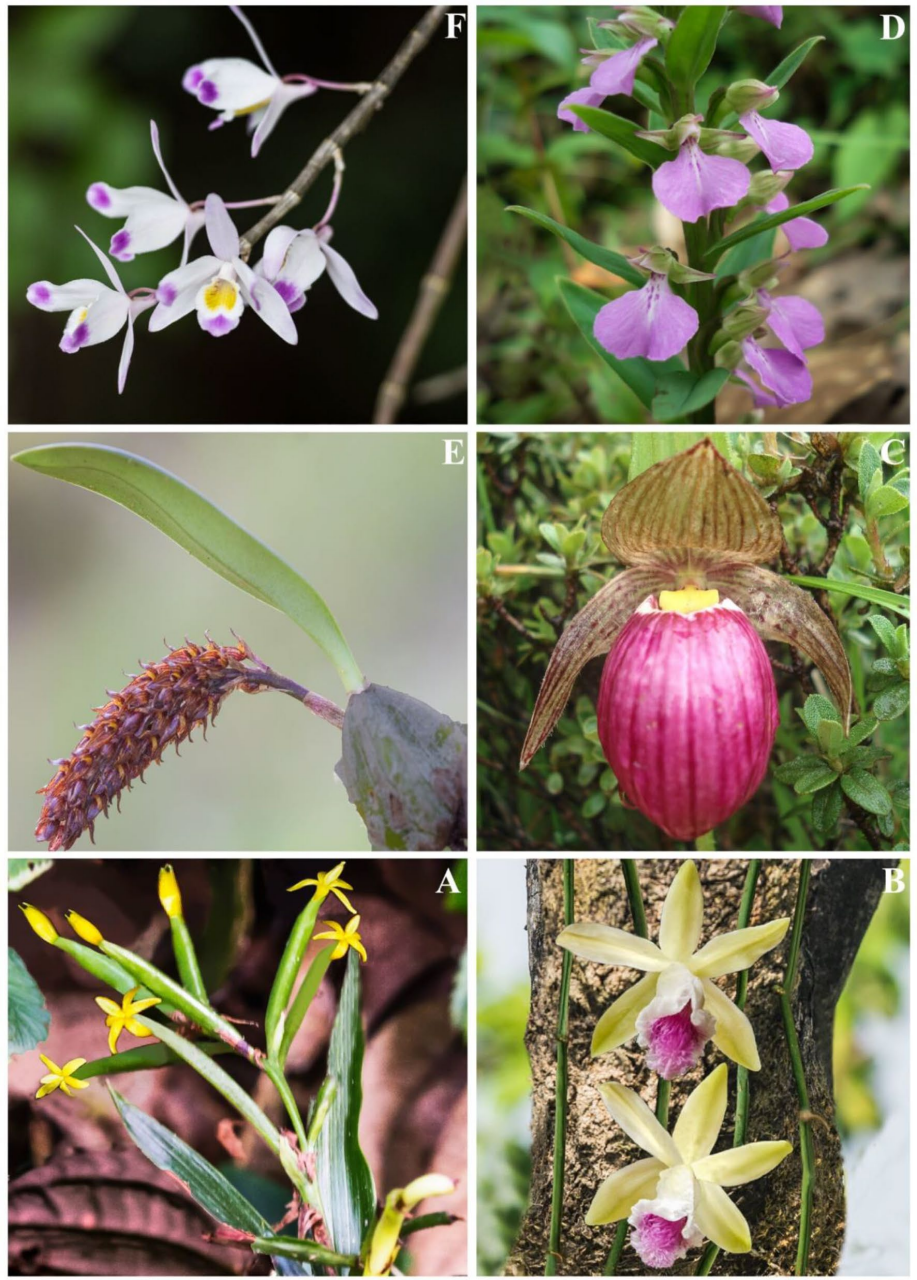
Representative species of medicinal orchids from five subfamilies. **(A)** *Apostasia wallichii* (Apostasioideae), **(B)** *Vanilla aphylla* (Vanilloideae), **(C)** *Cypripedium himalaicum* (Cypripedioideae), **(D)** *Brachycorythis obcordata* (Orchidoideae), **(E)** *Bulbophyllum careyanum* (Epidendroideae), **(F)** *Dendrobium amoenum* (Epidendroideae). Photographs by Bhakta B. Raskoti. Images merged in Adobe Illustrator CC v17.0. https://www.adobe.com.

Asia is considered as a hotspot of medicinal orchids due to the widespread commercial usages of orchids species as traditional medicine and the occurrence of these species in natural habitats. Based on various literatures, more than 70% medicinal orchid species are found in Asia^10,11^ representing all five subfamily (i.e. Apostasioideae, Vanilloideae, Cypripedioideae, Orchidoideae and Epidendroideae) of Orchidaceae (for example, Fig. 1). Usages of medicinal orchids are very popular in East Asia (mainly in China, Japan and Korea)^11,15^, South East Asia (especially in Thailand, Malaysia, Indonesia, Myanmar and Philippines)^11,16,17^, South Asia (usually in Bangladesh, Bhutan, India, Nepal, Pakistan)^10,11^ and western Asia (such as in Iran)^11^.

Growing popularity of herbal medicine in the twenty-first century has increased the demand of medicinal orchids in the global markets that has led to the overharvesting and illegal trade of wild orchids. There are several legislations for conservation and sustainable management of wild orchids. The most notable one is inclusion of all orchid species in the Convention on International Trade of Endangered Species of Fauna and Flora (CITES) in Appendices I or II^18^, which means that the collection and trade of orchids from wild habitat are banned. Despite several efforts to adopt national legislations and international treaties for conservation of wild orchids, high volumes of medicinal orchids are widely and illegally traded in the national, regional and international markets^19–22^. Overexploitation and illegal trade of wild orchids for medicinal usages have driven many orchid species towards extinction, for example; *Dendrobium officinale* Kimura & Migo and *Dendrobium huoshanense* C. Z. Tang et S. J. Cheng (a closely related species of *Dendrobium moniliforme* based on molecular phylogeny^23^) have been harvested from the wild habitats for medicinal use since 1200 years ago^19,20^, which enforced to enlist these species under critically endangered species^24,25^. To control overexploitation and illegal trade, the first and foremost crucial step is to correctly identify illegally traded orchids at their species level which in turn can help to understand their original locality (natural habitats) and to monitor that specific area for conservation and sustainable management.

**Figure 2:**
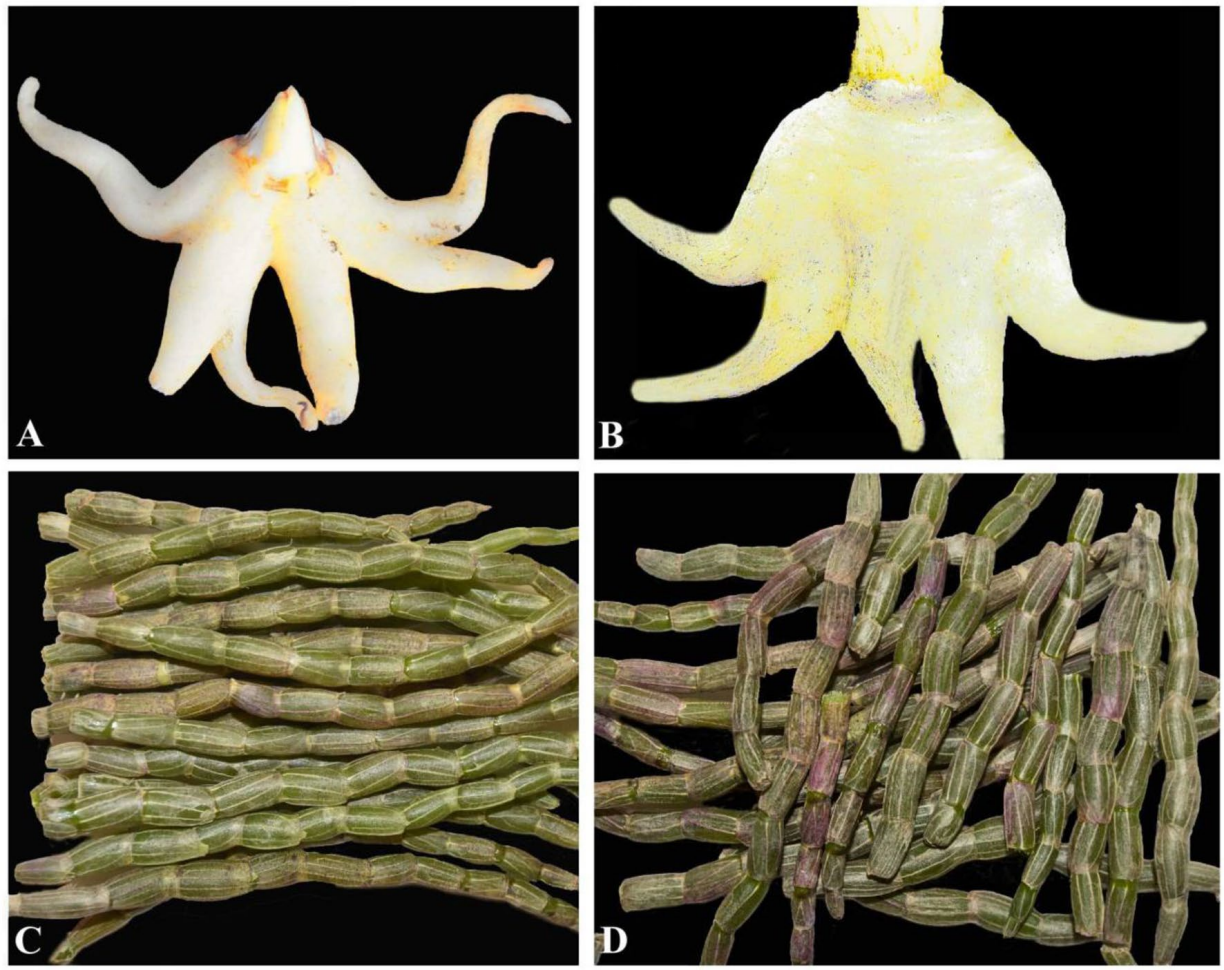
Tuber of *Gymnadenia orchidis* **(A)** and *Dactylorhiza incarnata* **(B)**, stems of *Dendrobium officinale* **(C)** and *Dendrobium moniliforme* **(D)**. Photographs by Bhakta B. Raskoti. Images merged in Adobe Illustrator CC v17.0. https://www.adobe.com.

Medicinal orchids are usually traded in the form of dried or fresh parts of plants such as pseudobulbs, tubers, leaves, stems and flowers. Morphological characters of these dried or fresh parts of different species look very similar and are difficult to differentiate from each other, for example, tuber of *Gymnadenia* and *Dactylorhiza* (Fig. 2A,B), stem of different species of *Dendrobium* (Fig. 2C,D), leaves of *Vanda* and *Aerides,* pseudobulb of many species of *Coelogyne* and *Bulbophyllum*. Accurate identification of orchid species from such dried or fresh materials based on morphological observation is almost impossible. To overcome this problem, DNA barcoding method can play a vital role for proper species level identification of medicinal orchids. Although few studies have been conducted on DNA barcoding of orchids using nuclear and plastid markers^26–31^, no concrete effort has been made so far focusing on DNA barcoding of medicinal orchids. Furthermore, in the vast majority of prior molecular works on orchids (in DNA barcoding and phylogenetic analysis), genomic DNA was extracted from leaf samples (e.g.^28–31^). But for the medicinal orchids, DNA extraction from tuber, stem and pseudobulb are equally important because these parts are major commodities of trade. Therefore, this research aims to evaluate DNA barcoding of medicinal orchids in Asia (where collection and trade of huge quantity of medicinal orchids have long existed) by using five barcodes (ITS, ITS2, *matK*, *rbcL* and *trnH-psbA*), which have been used in previous studies for DNA barcoding of angiosperms including orchids^29–34^. The specific objectives of this study were to: (1) test the use of existing protocol for DNA extraction from tuber, stem and pseudobulb of medicinal orchids and evaluate the success of amplification and sequencing, (2) assess efficacy of barcodes for identification of medicinal orchids and (3) establish a barcode dataset as a resource library for identification of medicinal orchids.

## Results

### Sequence success and characteristics

In this study, a total of 6986 sequences (including 431 newly generated sequences) were assembled to evaluate the five candidate barcodes (ITS, ITS2, *matK*, *rbcL* and *trnH-psbA* and their possible combinations). Sampling comprised 380 species belonging to 94 genera from five subfamily of Orchidaceae (Table 1). Our sample represents 90% species of medicinal orchids in Asia, which included all highly traded species. Sequence in the data matrix comprised 1823 (ITS), 1833 (ITS2 mainly excised from the aforementioned ITS sequences), 1414 (*matK*), 1109 (*rbcL*) and 807 (*trnH-psbA*) (Table 2). Summary of taxon sampling including sample ID, tissue source, gene bank accessions of newly generated and accessions of downloaded sequences are provided in Table 1, Table 2, Supplementary Table S1 and S2. In the aligned datasets, the maximum number of representative sequences of a species was limited to 20 individuals, and in the final data matrix about 80% species represented at least two individual sequences except for *trnH-psbA*.

**Table 1:** Summary of taxon sampling in this study.

Subfamily	Epidendroideae	Orchidoideae	Cypripedioideae	Vanilloideae	Apostasioideae	Total
No. of genus	60	27	2	3	2	94
No. of species	264	93	16	4	3	380
No. of sequences	5,157	1,547	237	26	19	6986
**Source of tissue for newly generated sequences**						
Leaves	55	31	–	–	–	86
Stem	14	–	–	–	–	14
Tuber	–	21	–	–	–	21
Pseudobulb	12	–	–	–	–	12

**Table 2:** Sequence characteristics of different DNA markers evaluated in this study.

DNA region	PCR and sequencing success rate (%)	No. of sequence	Sequence length		Genetic distance	
			Max	Min	Inter-specific	Intra-specific
ITS	99	1823	769	385	0–0.64	0–0.46
ITS2	–	1830	308	134	0–0.60	0–0.43
*matK*	96	1415	1813	361	0–0.07	0–0.06
*rbcL*	99	1107	1352	370	0–0.02	0–0.10
*trnH-psbA*	97	807	961	540	0–0.05	0–0.17

DNA extraction from tuber, pseudobulb and stem was 99% successful whereas extraction for the few samples that failed were mainly from stem and pseudobulb. The PCR amplification and sequencing success rates were high for ITS, *rbcL* (99%) and *trnH-psbA* (97%) (Table1). For *matK*, 96% of the sequences were successfully amplified and sequenced. Sequencing failed for 5 individuals because of polymorphic sites (double peaks) or a poly-G structure in the trace file. Failed sequences were re-sequenced. Some sequences were successfully obtained in the second attempt, but few samples still failed to generate readable sequences which were mainly from Orchidoideae. In total, about 97% of sequences were successfully sequenced. Sequence alignment was most consistent for *rbcL* and *matK*, followed by ITS. Conversely, *trnH-psbA* contains inversions as well as insertions and high level of sequence length variation, which makes the alignment extremely time consuming in comparison to other markers.

### Genetic distances and barcode gaps

The results of genetic distance indicated that ITS had the highest interspecific variation (0–0.64), followed by ITS2 (0–0.60), whereas *rbcL* was the most conserved and displayed the lowest interspecific divergence (0–0.02). Likewise, intraspecific genetic distance ranged highest in ITS (0–0.46) followed by ITS2 (0–0.43) and lowest in *rbcL* (0–0.10) and *matK* (0–06) (Table 2).

**Figure 3:**
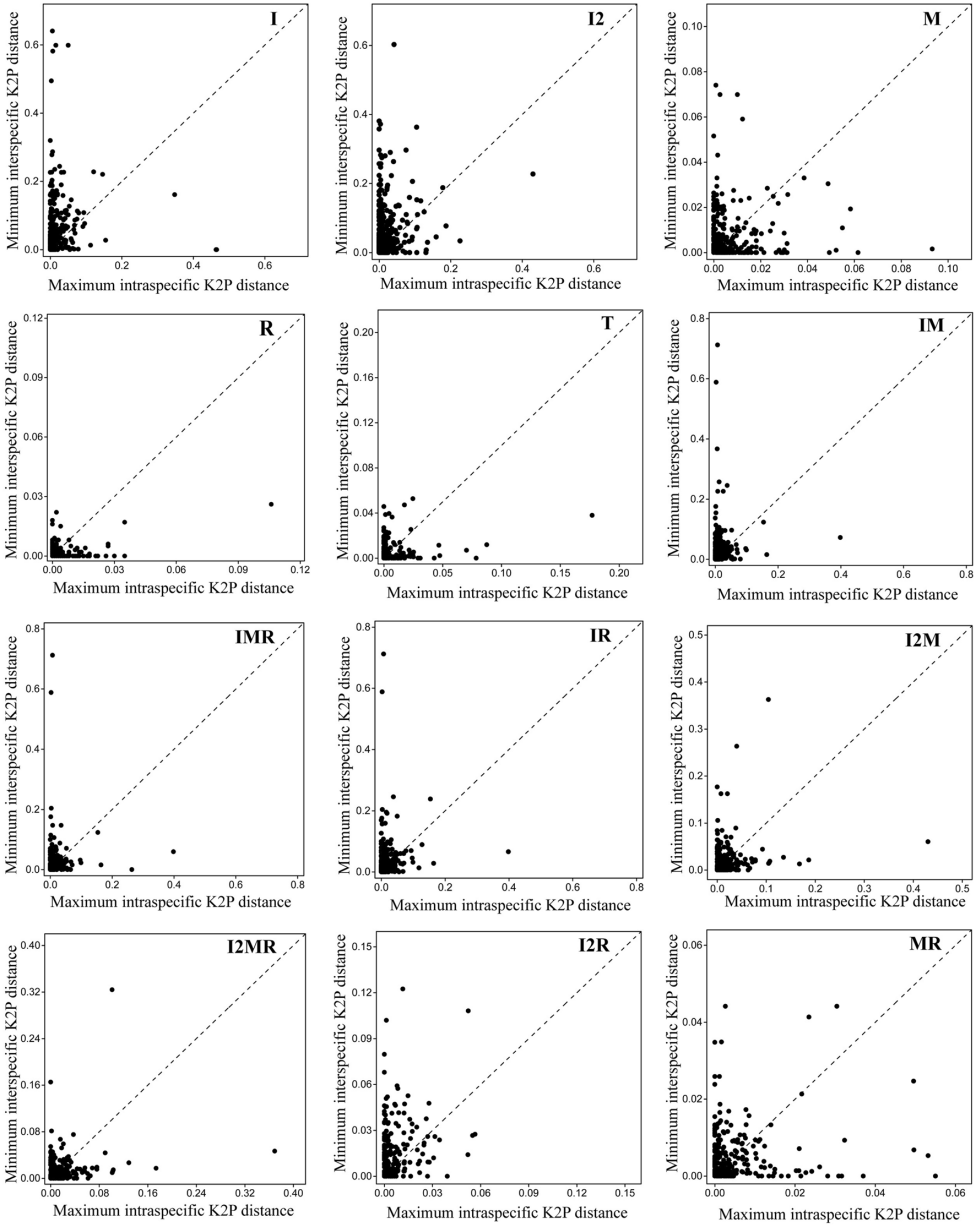
Scatter plots of the maximum intraspecific versus minimum interspecific K2P distance for five single markers and seven combinations (I, ITS; I2, ITS2; M, *matK*; R, *rbcL*; T, *trnH-psbA*).

The genetic distance method based on histograms did not detect distinct barcoding gaps and showed overlap between intra- and interspecific distance (Supplementary Fig. S1). In contrary to histograms, results based on scatter plots approach did detect barcoding gaps, which had different resolution between barcodes (Fig. 3, Table 3). Among single locus, ITS demonstrated the highest barcode gaps (84%), followed by ITS2 (80%), *trnH-psbA* (69.41%), *matK* (64.33%) and *rbcL* (60.33%). Among 2-loci combinations, ITS + *matK* exhibited highest (80.13%) barcode gaps followed by ITS2 + *matK* (71.11%), whereas ITS + *rbcL* and ITS2 + *rbcL* exhibited nearly similar barcode gaps (i.e. 69.05% and 69.84% respectively). The lowest barcode gaps were detected by *matK* + *rbcL* (63.20%). The combinations of 3-loci, ITS + *matK* + *rbcL* and ITS2 + *matK* + *rbcL* exhibited nearly equal barcode gaps i.e. 67.42% and 66.03% respectively (Table 3).

**Table 3:** Identification success rates obtained using Distance and NJ tree methods for the five single markers and seven combinations.

DNA region	Distance method (%)	NJ tree method (%)
ITS	84.71	90.27
ITS2	80.00	72.15
*matK*	64.33	67.56
*rbcL*	60.00	53.01
*trnH-psbA*	69.41	66.11
ITS + *matK*	80.13	80.04
ITS + *matK* + *rbcL*	67.42	70.20
ITS + *rbcL*	69.05	70.18
ITS2 + *matK*	71.11	70.00
ITS2 + *matK* + *rbcL*	66.03	68.24
ITS2 + *rbcL*	69.84	70.14
*matK* + *rbcL*	63.20	70.12

Result based on the BM analysis, ITS performed highest (91.62%) identification ability among the single barcode followed by *trnH*–*psbA* (84%), ITS2 (83.45%), *matK* (71.20%) and *rbcL* (43.10%). Among the multi-loci combinations, ITS + *rbcL* had the highest identification power (84.60%) followed by ITS + *matK* (80.51%), whereas the lowest capacity was exhibited by *matK* + *rbcL* (49.04) (Table 3). Based on the BCM method, the best-performing barcode for single locus was ITS (90.83%) followed by *trnH*–*psbA* (84.85%), whereas identification power was lowest for *rbcL* (43.10%) (Table 4). Among the combinations of 2-loci, the species identification performance was ranked as ITS + *rbcL* > ITS + *matK* > ITS2 + *rbcL* > ITS2 + *matK* > *matK* + *rbcL*. At the 3-locus combinations, the identification abilities were relatively lower than 2-loci combinations with resolution of 56.51% (ITS2 + *matK* + *rbcL*) to 63.55% (ITS + *matK* + *rbcL*) (Table 4).

**Table 4:** Species identification success based on best match and best close match.

DNA region	Best match (%)			Best close match (%)		
	Correct	Ambiguous	Incorrect	Correct	Ambiguous	Incorrect
ITS	91.62	4.18	4.18	90.83	4.12	3.95
ITS2	83.45	11.91	4.63	82.09	11.71	3.41
*matK*	71.72	18.72	9.54	71.65	18.72	9.47
*rbcL*	43.10	49.09	7.80	43.10	49.09	7.80
*trnH-psbA*	84.99	6.41	8.59	84.85	6.27	8.32
ITS + *matK*	80.51	1.08	18.41	80.15	1.08	18.77
ITS + *matK* + *rbcL*	66.33	0.30	33.35	63.55	0.23	25.80
ITS + *rbcL*	84.60	1.30	14.00	82.09	1.30	12.27
ITS2 + *matK*	66.32	1.20	32.40	64.71	1.10	27.48
ITS2 + *matK* + *rbcL*	59.14	0.93	39.91	56.51	0.68	33.36
ITS2 + *rbcL*	77.09	2.50	20.39	75.57	2.39	18.10
*matK* + *rbcL*	49.04	2.02	48.93	49.04	1.90	47.13

### Species discrimination

Neighbour-joining (NJ) trees obtained from the majority of barcodes exhibited nearly similar topology (Supplementary Figs. S2-S13) and agreed with the core phylogenetic hypothesis of Orchidaceae (e.g.^35^). Species discrimination rates among single-locus ranged from 53% (*rbcL*) to 90% (ITS), where *matK* and *trnH*–*psbA* performed 67.56% and 66.11% respectively. Among the 2-loci combinations, discrimination range comprised 70.12% (*matK* + *rbcL*) to 80% (ITS + *matK*). In the 3-loci combinations, highest species discrimination was exhibited by ITS + *matK* + *rbcL* (70.20%), whereas ITS2 + *matK* + *rbcL* had 68.24% (Table 3).

## Discussion

In the vast majority of the prior studies of DNA barcoding of Orchidaceae, genomic DNA was extracted from leaves (e.g.^28–31^) but illegal trade of medicinal orchids usually occurs by exporting their different parts such as tuber, stem, pseudobulb etc. Therefore, it is important to test and develop a protocol for DNA extraction from illegally traded parts of orchids for their accurate identification. In this study, we extracted DNA from the tuber, pseudobulb and stem samples, which were collected from traders or directly from wild habitats. Our study observed that DNA extraction is possible from tuber, pseudobulb and stem of medicinal orchids following existing protocol. This study found a high rate of PCR amplification and sequencing success for ITS, *rbcL*, *matK* as well as *trnH-psbA*, which is consistent with previous studies^29–31,34^. Comparatively, *trnH-psbA* seems to be slightly less successful due to presence of poly (T). Besides, some *trnH-psbA* sequences obtained with indels (i.e. insertion and inversions) caused complexity in data alignment. Such issue was also observed in previous studies^29,31^. Similarly, sequencing success rate of *matK* was relatively low due to presence of Poly (G). But generally, DNA extraction, PCR amplification and sequencing for ITS, *matK*, *rbcL* and *trnH-psbA* have no serious issues for DNA barcoding of medicinal orchids.

**Figure 4:**
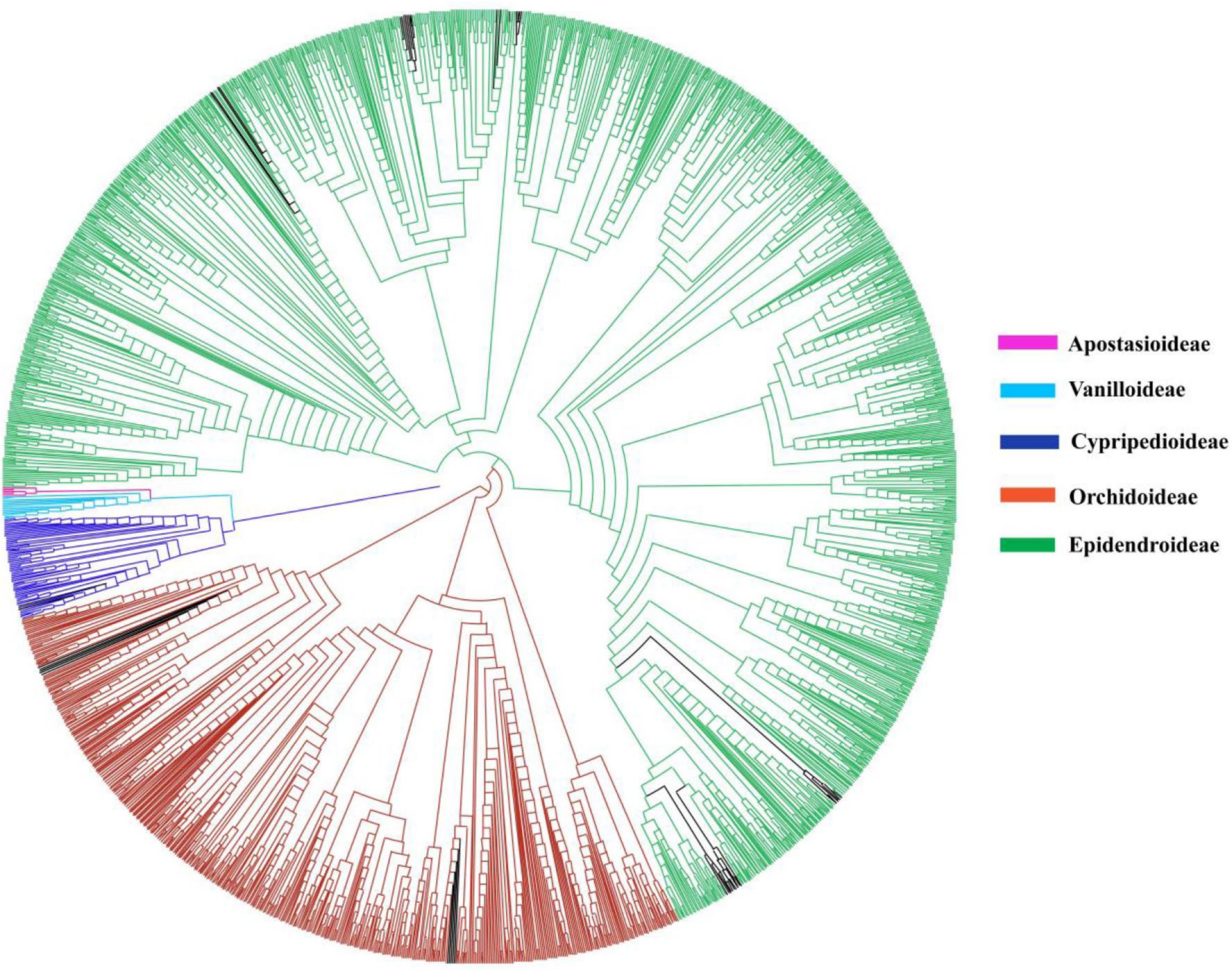
Neighbour-joining tree based on the ITS barcode. Coloured clades (except black) represent species that were correctly identified, and different sub-families are colour-coded. Black clades represent species that were not identified successfully. Details are included in Fig. S2.

Based on different analytical methods (genetic distance, BM, BCM, NJ), ITS performed the highest identification rate among the single barcode region (Fig. 4, Tables 3, 4, Supplementary Fig. S1). This rapidly evolving nuclear gene has the highest variable sites that contribute to the efficacy of species discrimination^36,37^. Our result is consistent with previous studies^29,31,32,36^. On the other hand, *matK, rbcL* and *trnH-psbA* (plastid barcodes) exhibited lower resolution than the ITS (nuclear barcode). This could be due to lower substitution rates found in the plastid region. Therefore, these plastid barcodes alone are not recommended for DNA barcoding of medicinal orchids. The low resolution of the plastid region has been reported in different seed plants including orchids^38,39^. In some studies^27,40^, other plastid barcodes such as *ndhF*, *ycf1*, *trnL-trnlF* (not evaluated in this study) were also found effective in DNA barcoding of Orchidaceae. However, in general these plastid barcodes are not commonly used in Orchidaceae. Therefore, additional studies are required to evaluate efficacy of *ndhF*, *ycf1*, *trnL-trnlF* for the DNA barcoding of the medicinal orchids.

Different combinations of two and three markers from ITS, ITS2, *matK*, *rbcL* were analysed in this study. Due to the comparatively low number of sampled species and sequences along with several indels, we excluded *trnH-psbA* in concatenation that may create robust missing data in the data matrix. Such missing data may have a negative impact on the results of phylogeny or tree-based DNA barcoding^41–44^. Although few studies have proposed *trnH-psbA* as a key barcode^36,45^, in this study *trnH-psbA* performed weak in species resolution except in BM and BCM analysis (Table 4). Moreover, several problems of *trnH-psbA* such as high frequency of length variation and the presence of inversions and insertions (which create complications to use it as a DNA barcode) have been reported^46–48^.

In the combinations of two barcode region, ITS + *matK* exhibited highest degree of species discrimination capability (Table 3) which is consistent with previous studies^29,31^. As an alternative to ITS + *matK*, a combination of ITS + *rbcL* could be a supplementary choice for the DNA barcoding of medicinal orchids (Table 4). The usefulness of this option (i.e. ITS + *rbcL*) is important particularly when *matK* amplification fails. The use of *matK* as a barcode has been criticized mainly because primers may be taxon specific or universal primers may not be available for all taxa^49^. Although *matK* amplification is not an immense problem in orchids, some studies from other groups of plants reported that *matK* sequencing is successful only after using up to 10-primer pairs^47^.

The combination of *matK* + *rbcL* exhibited relatively low efficacy for species identification, possibly because these two plastid markers are more conserved and lack sufficient variable sites. Therefore, *matK* + *rbcL* cannot be an ideal barcode for the medicinal orchids. Similar results were also reported in previous studies in different groups of plants^32,38^. By contrast, *matK* + *rbcL* was proposed as a core barcode for land plants^36,46,50^. Our samples were delimited to a single family (Orchidaceae) i.e. relatively more closely related species than in the sampling by^36,46,50^, indicating that *matK* + *rbcL* is not effective for barcoding of medicinal orchids.

Combinations of 3-locus candidates were unable to increase resolution rates, as they exhibited comparatively lower resolution than the combinations of 2-locus. Such kinds of results have been also reported in the previous studies^30,31^. Thus, combinations of 3-barcode regions are not recommended as efficient DNA barcode for the identification of medicinal orchids.

In this study, some species belonging to *Coelogyne*, *Cymbidium*, *Dendrobium* (Epidendroideae) and *Goodyera* (Orchidoideae) were not correctly identified by NJ method with majority of markers (Fig. 4, Supplementary Figs. S2-S12). Within the *Coelogyne*, two species i.e. *Coelogyne fimbriata* and *Coelogyne ovalis* were not distinctly identified. These two species are morphologically very similar and also share their geographical distribution ranges, which may lead to misidentification during sample collection. Besides, taxonomic identity of these two species is based on morphological studies but lacks assessment at molecular level, therefore likely to be the same species as assigned in taxonomic revision of *Coelogyne* section Fuliginosae (Orchidaceae)^51^. In the *Dendrobium*, *D.* *moniliforme* was not monophyletic, although a species should be monophyletic for the effectiveness of DNA barcoding^52^. *D*. *moniliforme* was also not resolved in the phylogenetic analysis of *Dendrobium*^23^ where several samples with the name ‘*Dendrobium* *moniliforme*’ were nested into different lineages. Possible reason could be improper taxonomic treatment or existence of cryptic species within *D. moniliforme*. In the *Goodyera*, *G.* *kwangtungensis* failed to distinguish itself from *G. schlechtendaliana*. It may be due to incorrect identification during sample collection possibly caused by their similar morphological characters. Another possibility is that these two species may be the same; the assignment of separate taxonomic identity of these species may be due to presence of ambiguous characters from the result of hybridization and polyploidization. *G.* *kwangtungensis* and *G. schlechtendaliana* are also not resolved in the molecular phylogenetic analysis^53^. Further studies are necessary to clarify the taxonomic status of these unresolved species.

A perfect DNA barcode usually should exhibit high interspecific but low intraspecific distances^36,45^, which can be clearly demonstrated either using histograms (for e.g.^31,40^) or scatter plots (for e.g.^29,32,33^). In this study, histograms approach failed to detect clear barcoding gaps (Supplementary Fig. S1), indicating that intraspecific genetic distance and interspecific genetic distance distributions overlapped with each other. This result is also in line with previous studies^29,32–34^. Conversely, barcoding gaps were detected using scatter plots where ITS performed the best among five single barcodes and ITS + *matK* presented the best among the multi-locus barcodes (Fig. 3). In the previous studies, barcode gaps were detected using scatter plots in varying degree between the barcodes^29,32,33^. The results of this study revealed that a rapidly evolving gene ITS is a powerful barcode in DNA barcoding gap assessment as well as efficacy of species identification success rate of medicinal orchids. Besides, ITS region is necessary in each of the most powerful multi-locus barcodes (i.e. ITS + *matK* and ITS + *rbcL*) indicating that ITS (having maximum intra- and interspecific genetic divergence comparisons) plays an important role to enhance barcode performance. Therefore, we recommend ITS region to be incorporated into the core barcode of medicinal orchids. This condition was also suggested by authors such as^38,45^, and strong positive effects of ITS locus have been reported in prior studies in different groups of plants^29,31,39^. Although few concerns have been raised about the use of ITS locus mainly due to fungal contamination^54,55^, but in orchids, ITS amplification and sequencing are already established and most commonly used in vast majority of molecular phylogenetic as well as DNA barcoding studies (for e.g.^26,29,31^).

## Conclusions

Based on three different analytical methods (genetic distance, similarity-based and tree-based) with sampling nearly 7000 sequences from five single barcodes (ITS, ITS2, *matK*, *rbcL*, *trnH-psbA* and their seven combinations), this study revealed that DNA barcoding is effective for identifying medicinal orchids. Among single locus, ITS performed the best barcode (amplification, sequencing and species identification). Among combined barcode loci, ITS + *matK* exhibited the most efficient barcode for the DNA barcoding of medicinal orchids. Alternative to ITS + *matK*, a combination of ITS + *rbcL* could be another multi-locus barcode option. This study indicated that a rapidly evolving gene ITS is important for the DNA barcoding of the medicinal orchids. Based on genetic distance analysis, we also suggest using scatter plots instead of histograms to detect the presence of DNA barcoding gaps in the medicinal orchids. Furthermore, the success rate of amplification and sequencing is high and the existing protocol is applicable for DNA extraction from tuber, stem and pseudobulb of medicinal orchids. A barcode library (assembling sequences from five loci ITS, ITS2, *matK*, *rbcL* and *trnH-psbA*) as a resource for identifying medicinal orchids has been established which contains about 7,000 sequences of 380 species (i.e. 90%) of medicinal orchids of Asia. Future studies will enhance this barcode library mainly by adding sequences from the remaining 10% species.

## Methods

### Sampling strategy

We compiled a checklist of medicinal orchids from Asia based on published literatures (e. g.^10,11^, and then the accepted species name was assigned following The Plant List and various recently published papers. Medicinal orchids in the checklist (within Asia) comprised 422 species and represent all five subfamily (i.e. Apostasioideae, Vanilloideae, Cypripedioideae, Orchidoideae and Epidendroideae) of Orchidaceae.

In total, 6,555 sequences were retrieved from the National Center for Biotechnology Information (NCBI) (see Supplementary Table S1). For this, priorities were given to those sequences, which are already published in papers or have provided voucher specimens so that misidentified sequences can be avoided. In cases where large numbers of sequences were available for a species per marker, we selected the ones, which have good quality, the longest sequences and represent from different geographical regions. Furthermore, the downloaded sequences from NCBI were filtered according to the following criteria: (1) omitted sequences having length less than 300 bp but this criteria is not applied for some ITS2 region; (2) excluded sequences lacking voucher specimens; and (3) discarded sequences having taxa without specific names (such as *Habenaria *sp. and *Bulbophyllum* cff. etc.).

In this study, 431 sequences were newly generated from 134 individuals representing 48 species that were collected from different localities of Nepal (mainly from community based forest). The national guidelines were followed for the collection and use of plants. The plant samples collected for the present study are currently neither included in the IUCN red list nor listed as protected plants. Although these plants are included in CITES Appendix II, there was no any provision to take collection permit during the time of fieldwork. The localities of species collected in this work are not from protected area; hence no permits were required. However, we did inform the related community forest user groups (local institutions under the district forest based on Forest Act 1993 enacted by Ministry forest and Environment, Government of Nepal) and took verbal consent for specimen collection.

We reviewed related floras, monographs and compared specimens with printed as well as online images including available images of type specimens for the species identification. Species were formally identified by Dr. Bhakta Bahadur Raskoti, Biodiversity Conservation Initiative, Nepal. All newly generated sequences have been submitted to NCBI (Supplementary Table S2). Voucher specimens were deposited in National Herbarium and Plant Laboratories (KATH), voucher number are also available publicly in NCBI gene bank accession records.

### DNA extraction, PCR amplification and sequencing

Total genomic DNA was extracted from plant leaves dried in silica-gel following modified CTAB protocol^56^. We also extracted genomic DNA from tuber, stem, pseudobulb (total 47 samples from 20 species) dried in silica-gel. For this DNA was extracted following STE-CTAB protocol^57^. Amplification of DNA regions was performed using a polymerase chain reaction (PCR) following the reference^58^. The sequencing reactions were performed using the Applied Bio-systems Prism Bigdye Terminator Cycle Sequencing (Applied Bio-systems, Foster City, CA) following the manufacture’s instructions. Primer pairs for PCR and sequencing used in this study are provided in Supplementary Table S3.

### Data analysis

Forward and reverse sequencing output files were edited and assembled using ContigExpress Application 6.0 (InforMax, Inc.). Assembled sequences were initially aligned using Clustal X^59^ and then manually adjusted in BIOEDIT version 7^60^. Altogether twelve barcodes were evaluated including five single loci (ITS, ITS2, *matK*, *rbcL*, *trnH-psbA*) and seven combinations (ITS + *matK*, ITS + *rbcL*, ITS2 + *matK*, ITS2 + *rbcL*, *matK* + *rbcL*, ITS + *matK* + *rbcL*, ITS2 + *matK* + *rbcL*) using following methods.

### Genetic distance-based method

The genetic pairwise distance for each marker was calculated in MEGA X^61^ using the Kimura 2-parameter model, and we investigated the minimum interspecific distance and maximum intraspecific distance for each species using custom R script. To detect the barcode gaps, scatter plots were generated using R version 3.6.3^62^. In scatter plots, each dot represents a species and the dot above the 1:1 slope indicates a barcoding gap^32,33,63^. We counted the number of species having barcoding gaps for each marker; finally these barcode gaps were calculated in percentage. We also used histograms to detect barcoding gaps for every single and multi-loci barcode. Histograms were generated from the distribution of intraspecific and interspecific genetic distances obtained from pairwise summary function using the program TaxonDNA^64^.

### Similarity-based method

To assess the proportion of accurate species identification, best match (BM) and best close match (BCM) functions were implemented in the TaxonDNA^64^. For BM analysis, identification was considered correct when query and best match sequences were from the same species, ambiguous when they were from both the same and different species, or incorrect when they belonged to different species^33,64^. For BCM, species identification was considered correct if a query matched all conspecific sequences within the 95% pairwise genetic threshold^33,64^. In BM and BCM analysis we deleted all species represented by a single sequence.

### Tree-based method

To evaluate discriminatory power of single and multi-locus barcodes, unrooted neighbour-joining (NJ) trees were constructed in MEGA X^61^. For this pairwise deletion based on the p-distance model following protocols for species level discrimination in the closely related species were applied^34,64,65^. A species was considered successfully identified only when all conspecific individuals formed a monophyletic clade.

## Supplementary Information

Supplementary Figure S1.

Supplementary Figure S2.

Supplementary Figure S3.

Supplementary Figure S4.

Supplementary Figure S5.

Supplementary Figure S6.

Supplementary Figure S7.

Supplementary Figure S8.

Supplementary Figure S9.

Supplementary Figure S10.

Supplementary Figure S11.

Supplementary Figure S12.

Supplementary Figure S13.

Supplementary Table S1.

Supplementary Table S2.

Supplementary Table S3.

Supplementary Information 1.

Supplementary Information 2.

Supplementary Information 3.

Supplementary Information 4.

Supplementary Information 5.

## Data Availability

GenBank accession numbers for nucleotide sequences: see Supplementary Table S1 and Table S2. DNA sequences: Aligned sequences Supplementary Data S1–S5.

## References

[1] Gutiérrez RMP (2010). Orchids: A review of uses in traditional medicine, its phytochemistry and pharmacology. J. Med. Plant Res..

[2] Hossain MM (2011). Therapeutic orchids: traditional uses and recent advances—An overview. Fitoterapia.

[3] Du XM, Sun NY, Shoyama Y (2000). Flavonoids from *Goodyera schlechtendaliana*. Phytochemistry.

[4] Du XM, Sun NY, Takizawa N, Guo YT, Shoyama Y (2002). Sedative and anticonvulsant activities of goodyerin, a flavonol glycoside from *Goodyera schlechtendaliana*. Phytother. Res..

[5] Liu XJ, Yang Y (1958). Studies on constituents of Tian ma (*Gastrodia elata* Bl.) I. Extraction and identification of Vanilyalcohol. J. Shanghai Med. Univ..

[6] Sun XF, Wang W, Wang DQ, Du GY (2004). Research progress of neuroprotective mechanisms of *Gastrodia elata* and its preparation. China J. Chin. Mater. Med..

[7] Suzuki H, Keimatsu I, Ito M (1932). Alkaloid of the Chinese drug “Chin-Shih-Hu” II. Dendrobine. J. Pharm. Soc. Jpn..

[8] Li R (2017). Anti-influenza A virus activity of dendrobine and its mechanism of action. J. Agric. Food Chem..

[9] Bulpitt CJ, Li Y, Bulpitt PF, Wang J (2007). The use of orchids in Chinese medicine. J. R. Soc. Med..

[10] Pant B, Raskoti BB (2013). Medicinal orchids of Nepal.

[11] Teoh ES (2016). Medicinal Orchids of Asia.

[12] Reinikka MA (1995). A History of the Orchid.

[13] Berliocchi, L., & Griffiths, M. *Orchid in Lore and Legend*. (Timber Press, 2000).

[14] Guthrie D (1945). A History of Medicine.

[15] Zhao, Z., Yang, Z., & Iida, O. Supply and cultivation of medicinal plants in Japan. in *Current Review of Chinese Medicine: Quality Control of Herbs and Herbal Medicine.* (eds Leung, P. C., Fong, H. & Xue, C. C.). 59–72. (World Scientific Publishing Company, 2006).

[16] Chuakul W (2002). Ethnomedical uses of Thai orchidaceous plants. Mohidol Univ. J. Pharm. Sci..

[17] San, M. M., Aung, N. M., Soe, H. S., & Kyaw, Y. M. M. Study on distribution and medicinal values of wild orchids in Matu Pe Township, Southern Chin State. in *The Republic of Myanmar Ministry of Environmental Conservation and Forestry, Forest Department Leaflet*. Vol. 30. (2015).

[18] CITES. *Criteria for the Inclusion of Species in Appendices I, II and III, Valid from 2020 08–28*. https://cites.org/sites/default/files/eng/app/2020/E-Appendices-2020-08-28.pdf (2020).

[19] Cheng J (2019). An assessment of the Chinese medicinal *Dendrobium* industry: Supply, demand and sustainability. J. Ethnopharmacol..

[20] He P, Song X, Luo Y, He M (2009). Reproductive biology of *Dendrobium officinale* (Orchidaceae) in Danxia landform. China J. Chin. Mater. Med..

[21] Hinsley A (2018). A review of the trade in orchids and its implications for conservation. Bot. J. Linn. Soc..

[22] Subedi A (2013). Collection and trade of wild-harvested orchids in Nepal. J. Ethnobiol. Ethnomed..

[23] Xiang XG (2013). Molecular systematics of *Dendrobium* (Orchidaceae, Dendrobieae) from mainland Asia based on plastid and nuclear sequences. Mol. Phylogenet. Evol..

[24] China Plant Specialist Group. *Dendrobium huoshanense*. *The IUCN Red List of Threatened Species* 2004: e.T46661A11074076. 10.2305/IUCN.UK.2004.RLTS.T46665A11074270.en. Accessed 18 Sep 2020.

[25] China Plant Specialist Group. *Dendrobium officinale*. The IUCN Red List of Threatened Species 2004: e.T46665A11074270. http://dx.doi.org/10.2305/IUCN.UK.2004.RLTS.T46665A11074270.en. Accessed 18 Sep 2020.

[26] De Boer HJ (2017). DNA metabarcoding of orchid-derived products reveals widespread illegal orchid trade. Proc. R. Soc. B.

[27] Ghorbani A, Gravendeel B, Selliah S, Zarré S, De Boer H (2017). DNA barcoding of tuberous Orchidoideae: A resource for identification of orchids used in Salep. Mol. Ecol. Resour..

[28] Kim HM, Oh SH, Bhandari GS, Kim CS, Park CW (2014). DNA barcoding of Orchidaceae in Korea. Mol. Ecol. Resour..

[29] Li Y, Tong Y, Xing F (2016). DNA barcoding evaluation and its taxonomic implications in the recently evolved genus *Oberonia* Lindl. (Orchidaceae) in China. Front. Plant Sci..

[30] Xiang XG, Hu HAO, Wang WEI, Jin XH (2011). DNA barcoding of the recently evolved genus *Holcoglossum* (Orchidaceae: Aeridinae): A test of DNA barcode candidates. Mol. Ecol. Resour..

[31] Xu S (2015). Evaluation of the DNA barcodes in *Dendrobium* (Orchidaceae) from mainland Asia. PLoS ONE.

[32] Liu JX (2014). Identification of species in the angiosperm family Apiaceae using DNA barcodes. Mol. Ecol. Resour..

[33] Xu SZ, Li ZY, Jin XH (2018). DNA barcoding of invasive plants in China: A resource for identifying invasive plants. Mol. Ecol. Resour..

[34] Yan LJ (2015). DNA barcoding of *Rhododendron* (Ericaceae), the largest Chinese plant genus in biodiversity hotspots of the Himalaya-Hengduan Mountains. Mol. Ecol. Resour..

[35] Givnish TJ (2015). Orchid phylogenomics and multiple drivers of their extraordinary diversification. Proc. R. Soc. B.

[36] Kress WJ, Wurdack KJ, Zimmer EA, Weigt LA, Janzen DH (2005). Use of DNA barcodes to identify flowering plants. Proc. Natl. Acad. Sci. U.S.A..

[37] Sass C, Little DP, Stevenson DW, Specht CD (2007). DNA barcoding in the Cycadales: Testing the potential of proposed barcoding markers for species identification of cycads. PLoS ONE.

[38] Chen J, Zhao JT, Erickson DL, Xia N, Kress WJ (2015). Testing DNA barcodes in closely related species of *Curcuma* (Zingiberaceae) from Myanmar and China. Mol. Ecol. Resour..

[39] Gao T, Yao H, Song J, Zhu Y, Liu C, Chen S (2010). Evaluating the feasibility of using candidate DNA barcodes in discriminating species of the large Asteraceae family. BMC Evol. Biol..

[40] Li H (2021). The specific DNA barcodes based on chloroplast genes for species identification of Orchidaceae plants. Sci. Rep..

[41] Hovmoller R, Knowles LL, Kubatko LS (2013). Effects of missing data on species tree estimation under the coalescent. Mol. Phylogenet. Evol..

[42] Xi Z, Liu L, Davis CC (2016). The impact of missing data on species tree estimation. Mol. Biol. Evol..

[43] Wiens JJ, Moen DS (2008). Missing data and the accuracy of Bayesian phylogenetics. J. Syst. Evol..

[44] Wiens JJ (2003). Incomplete taxa, incomplete characters, and phylogenetic accuracy: Is there a missing data problem?. J. Vertebr. Paleontol..

[45] Kress WJ, Erickson DL (2007). A two-locus global DNA barcode for land plants, the coding *rbcL* gene complements the non-coding *trnH-psbA* spacer region. PLoS ONE.

[46] CBOL Plant Working Group (2009). A DNA barcode for land plants. Proc. Natl. Acad. Sci. U.S.A..

[47] Fazekas AJ (2008). Multiple multilocus DNA barcodes from the plastid genome discriminate plant species equally well. PLoS ONE.

[48] Whitlock BA, Hale AM, Groff PA (2010). Intraspecific inversions pose a challenge for the *trnH-psbA* plant DNA barcode. PLoS ONE.

[49] Bafeel SO (2011). Comparative evaluation of PCR success with universal primers of maturase K (*matK*) and ribulose-1, 5-bisphosphate carboxylase oxygenase large subunit (*rbcL*) for barcoding of some arid plants. Plant Omics.

[50] Chase MW (2007). A proposal for a standardised protocol to barcode all land plants. Taxon.

[51] Pelser PB, Gravendeel B, De Vogel EF (2000). Revision of *Coelogyne* section Fuliginosae (Orchidaceae). Blumea.

[52] Hebert PDN, Cywinska A, Ball SL (2003). Biological identifications through DNA barcodes. Proc. R. Soc. B..

[53] Hu C (2016). Phylogenetic analysis of a ‘jewel orchid’ genus *Goodyera* (Orchidaceae) based on DNA sequence data from nuclear and plastid regions. PLoS ONE.

[54] Cullings KW, Vogler DR (1998). A 5.8S nuclear ribosomal RNA gene sequence database. Mol. Ecol..

[55] Hollingsworth PM (2011). Refining the DNA barcode for land plants. Proc. Natl. Acad. Sci. U.S.A..

[56] Doyle JJ, Doyle JL (1987). A rapid isolation procedure from small quantities of fresh leaf tissue. Phytochem. Bull..

[57] Shepherd LD, McLay TGB (2011). Two micro-scale protocols for the isolation of DNA from polysaccharide-rich plant tissue. J. Plant Res..

[58] Raskoti BB (2016). A phylogenetic analysis of molecular and morphological characters of *Herminium* (Orchidaceae, Orchideae): Evolutionary relationships, taxonomy, and patterns of character evolution. Cladistics.

[59] Larkin MA (2007). Clustal W and Clustal X version 2.0. Bioinformatics.

[60] Hall TA (1999). BioEdit: A user-friendly biological sequence alignment editor and analysis program for Windows 95/98/NT. Nucleic Acids Symp. Ser..

[61] Tamura K, Stecher G, Peterson D, Filipski A, Kumar S (2013). MEGA6: Molecular evolutionary genetics analysis version 6.0. Mol. Biol. Evol..

[62] R Development Core Team. *R: A Language and Environment for Statistical Computing*. (R Foundation for Statistical Computing, 2020).

[63] Collins R, Cruickshank R (2013). The seven deadly sins of DNA barcoding. Mol. Ecol. Resour..

[64] Meier R, Shiyang K, Vaidya G, Ng PK (2006). DNA barcoding and taxonomy in Diptera: A tale of high intraspecific variability and low identification success. Syst. Biol..

[65] Srivathsan A, Meier R (2012). On the inappropriate use of Kimura-2 parameter (K2P) divergences in the DNA-barcoding literature. Cladistics.

