# Supplementary material for: DNA barcoding of medicinal orchids in Asia
Source: Sci Rep. 2021 Dec 8;11:23651. doi: 10.1038/s41598-021-03025-0 (PMC8654824; doi:10.1038/s41598-021-03025-0)
Supplement: Supplementary file 1 — Supplementary Figure S1. [file 41598_2021_3025_MOESM1_ESM.pdf]

Figure S1 Relative distribution of intra-specific and inter-specific genetic distances for all single and combination of barcodes in medicinal orchids. X-axis denotes genetic distances, and the Y-axis denotes the percentage of occurrences.

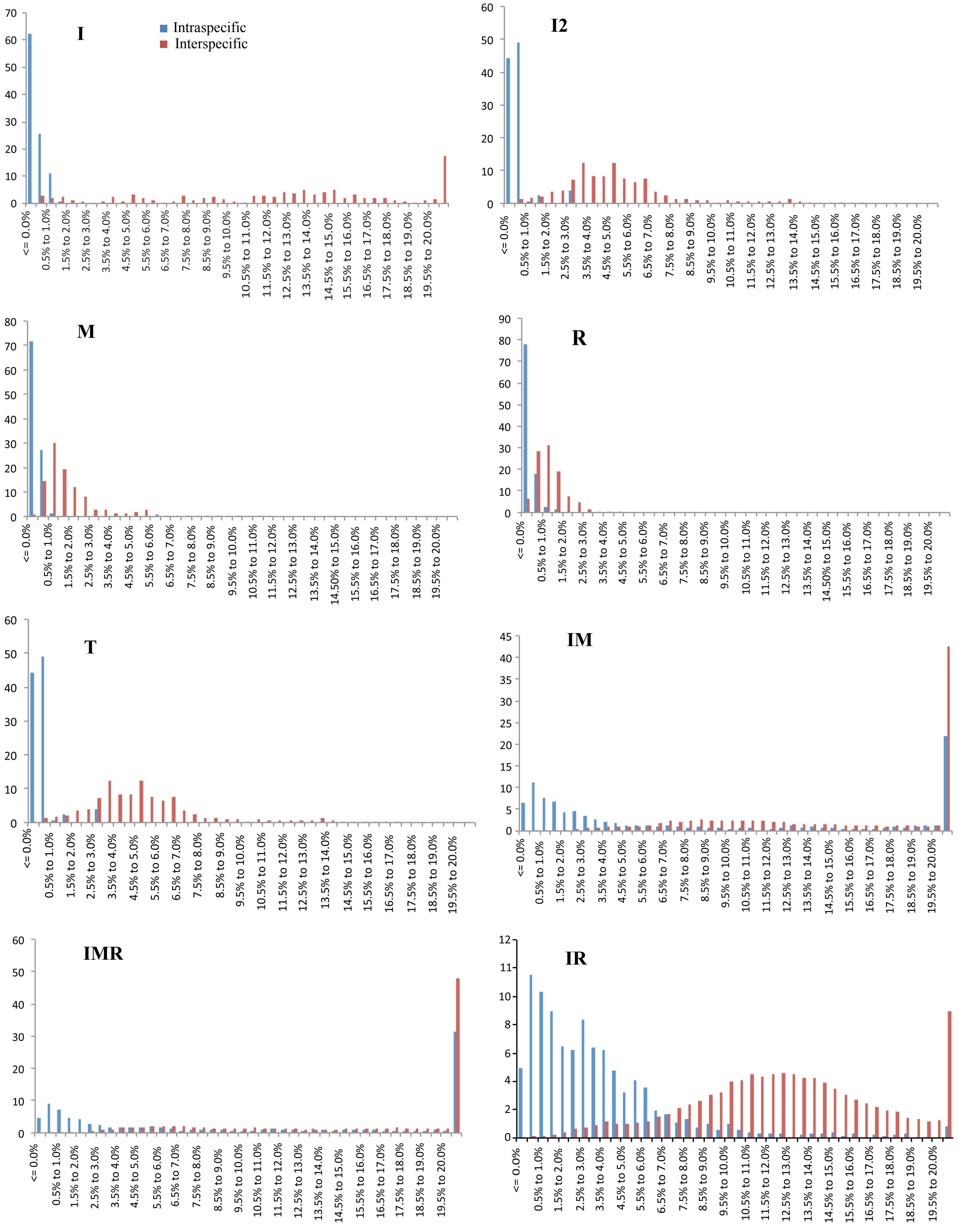

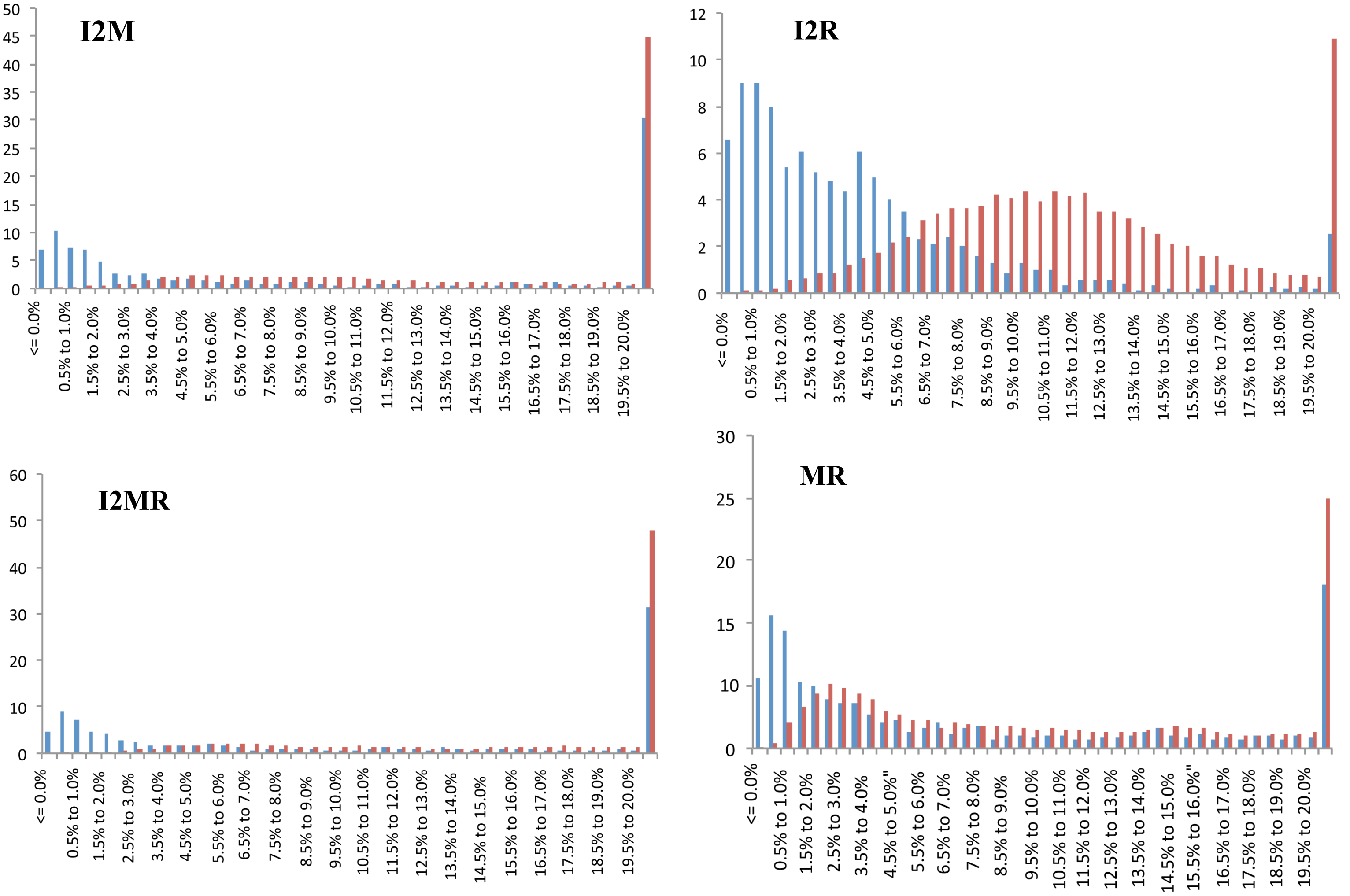

I = ITS
I2 = ITS2
M = *matK*
R = *rbcL*
T = *trnH-psbA*
IM = ITS + *matK*
IMR = ITS + *matK* + *rbcL*
IR = ITS + *rbcL*
I2M = ITS2 + *matK*
I2MR = ITS2 + *matK* + *rbcL*
I2R = ITS2 + *rbcL*
MR = *matK* + *rbcL*
