# Supplementary material for: DNA barcoding of medicinal orchids in Asia
Source: Sci Rep. 2021 Dec 8;11:23651. doi: 10.1038/s41598-021-03025-0 (PMC8654824; doi:10.1038/s41598-021-03025-0)
Supplement: Supplementary file 16 — Supplementary Table S3. [file 41598_2021_3025_MOESM16_ESM.docx]

**Table S3** Primers used for PCR amplification and sequencing in this study.

| **Region** | **Primers** | **Sequence (5-3)** | **Reference** |
| --- | --- | --- | --- |
| *rbcL* | 1F  1360R | ATG TCA CCA CAA ACA GAA AC  CTT CAC AAG CAG CAG CTA GTT C | 1 |
| ITS  ITS2 | 17SE  26SE  5.8 F | ACG AAT TCA TGG TCC GGT GAA GTG TTC G  TAG AAT TCC CCG GTT CGC TCG CCG TTA C  GCCTGGGCGTCACGC | 2 |
| *matK* | 390F  1326R | CGA TCT ATT CAT TCA ATA TTT C  TCT AGC ACA CGA AAG TCG AAG T | 3 |
| *trnH-psbA* | trnH  psbA | CGC GCA TGG TGG ATT CAC AAT CC  GTT ATG CAT GAACGT AAT GCT C | 4 |

References

1. Goldman, D. H. *et al.* Phylogenetics of Arethuseae (Orchidaceae) based on plastid *matK* and *rbcL* sequences. *Syst. Bot.* *26*(3), 670–695 (2001).

2. Sun, Y., Skinner, D. Z., Liang, G. H., & Hulbert, S. H. Phylogenetic analysis of *Sorghum* and related taxa using internal transcribed spacers of nuclear ribosomal DNA. *Theor. Appl. Genet.* *89*(1), 26–32 (1994).

3. Cuénoud, P. *et al*. Molecular phylogenetics of Caryophyllales based on nuclear 18S rDNA and plastid *rbcL*, *atpB*, and *matK* DNA sequences. *Am. J. Bot.* *89*(1), 132–144 (2002).

4. Shaw, J. *et al*. The tortoise and the hare II: relative utility of 21 noncoding chloroplast DNA sequences for phylogenetic analysis. *Am. J. Bot.* *92*(1), 142–166 (2005).
