# Supplementary material for: DNA barcoding of medicinal orchids in Asia
Source: Sci Rep. 2021 Dec 8;11:23651. doi: 10.1038/s41598-021-03025-0 (PMC8654824; doi:10.1038/s41598-021-03025-0)
Supplement: Supplementary file 2 — Supplementary Figure S2. [file 41598_2021_3025_MOESM2_ESM.pdf]

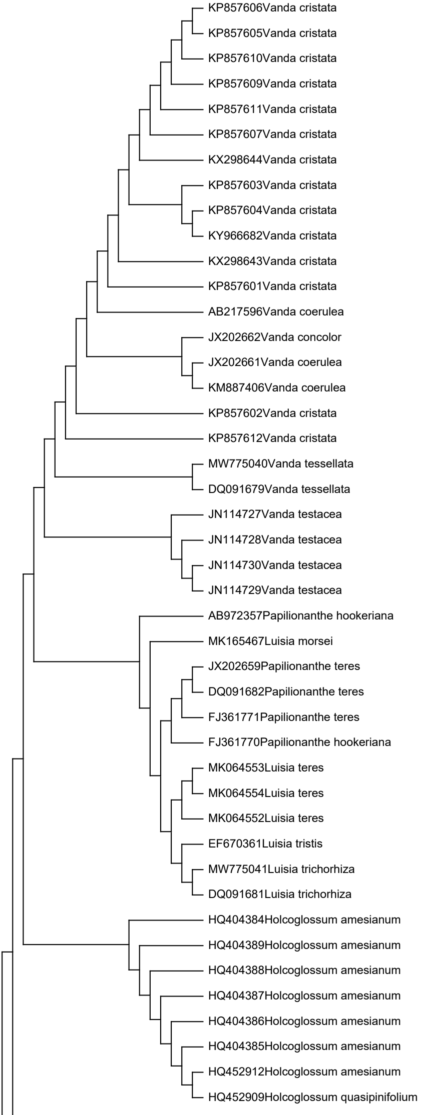
KP857606Vanda cristata
KP857605Vanda cristata
KP857610Vanda cristata
KP857609Vanda cristata
KP857611Vanda cristata
KP857607Vanda cristata
KX298644Vanda cristata
KP857603Vanda cristata
KP857604Vanda cristata
KY966682Vanda cristata
KX298643Vanda cristata
KP857601Vanda cristata
AB217596Vanda coerulea
JX202662Vanda concolor
JX202661Vanda coerulea
KM887406Vanda coerulea
KP857602Vanda cristata
KP857612Vanda cristata
MW775040Vanda tessellata
DQ091679Vanda tessellata
JN114727Vanda testacea
JN114728Vanda testacea
JN114730Vanda testacea
JN114729Vanda testacea
AB972357Papilionanthe hookeriana
MK165467Luisia morsei
JX202659Papilionanthe teres
DQ091682Papilionanthe teres
FJ361771Papilionanthe teres
FJ361770Papilionanthe hookeriana
MK064553Luisia teres
MK064554Luisia teres
MK064552Luisia teres
EF670361Luisia tristis
MW775041Luisia trichorhiza
DQ091681Luisia trichorhiza
HQ404384Holcoglossum amesianum
HQ404389Holcoglossum amesianum
HQ404388Holcoglossum amesianum
HQ404387Holcoglossum amesianum
HQ404386Holcoglossum amesianum
HQ404385Holcoglossum amesianum
HQ452912Holcoglossum amesianum
HQ452909Holcoglossum quasipinifolium

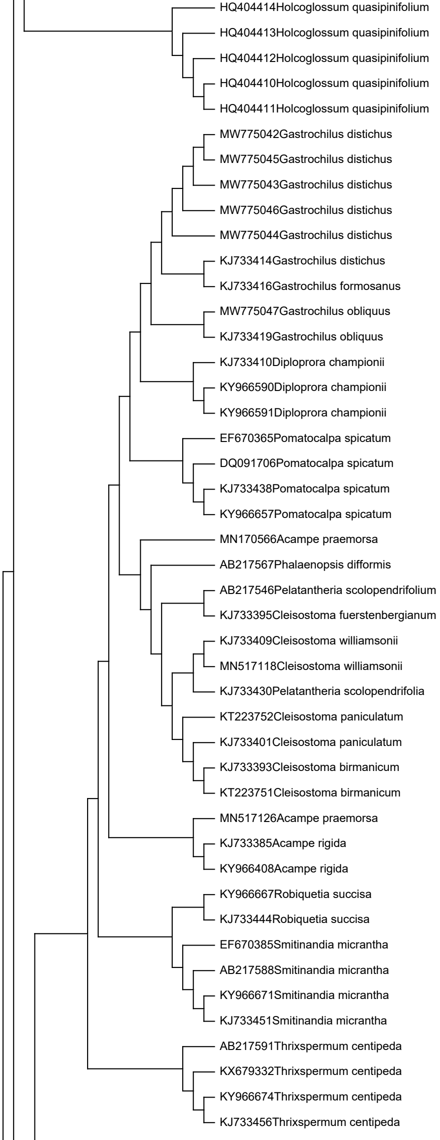
HQ404414Holcoglossum quasipinifolium
HQ404413Holcoglossum quasipinifolium
HQ404412Holcoglossum quasipinifolium
HQ404410Holcoglossum quasipinifolium
HQ404411Holcoglossum quasipinifolium
MW775042Gastrochilus distichus
MW775045Gastrochilus distichus
MW775043Gastrochilus distichus
MW775046Gastrochilus distichus
MW775044Gastrochilus distichus
KJ733414Gastrochilus distichus
KJ733416Gastrochilus formosanus
MW775047Gastrochilus obliquus
KJ733419Gastrochilus obliquus
KJ733410Diploprora championii
KY966590Diploprora championii
KY966591Diploprora championii
EF670365Pomatocalpa spicatum
DQ091706Pomatocalpa spicatum
KJ733438Pomatocalpa spicatum
KY966657Pomatocalpa spicatum
MN170566Acampe praemorsa
AB217567Phalaenopsis difformis
AB217546Pelatantheria scolopendrifolium
KJ733395Cleisostoma fuerstenbergianum
KJ733409Cleisostoma williamsonii
MN517118Cleisostoma williamsonii
KJ733430Pelatantheria scolopendrifolia
KT223752Cleisostoma paniculatum
KJ733401Cleisostoma paniculatum
KJ733393Cleisostoma birmanicum
KT223751Cleisostoma birmanicum
MN517126Acampe praemorsa
KJ733385Acampe rigida
KY966408Acampe rigida
KY966667Robiquetia succisa
KJ733444Robiquetia succisa
EF670385Smitinandia micrantha
AB217588Smitinandia micrantha
KY966671Smitinandia micrantha
KJ733451Smitinandia micrantha
AB217591Thrixspermum centipeda
KX679332Thrixspermum centipeda
KY966674Thrixspermum centipeda
KJ733456Thrixspermum centipeda

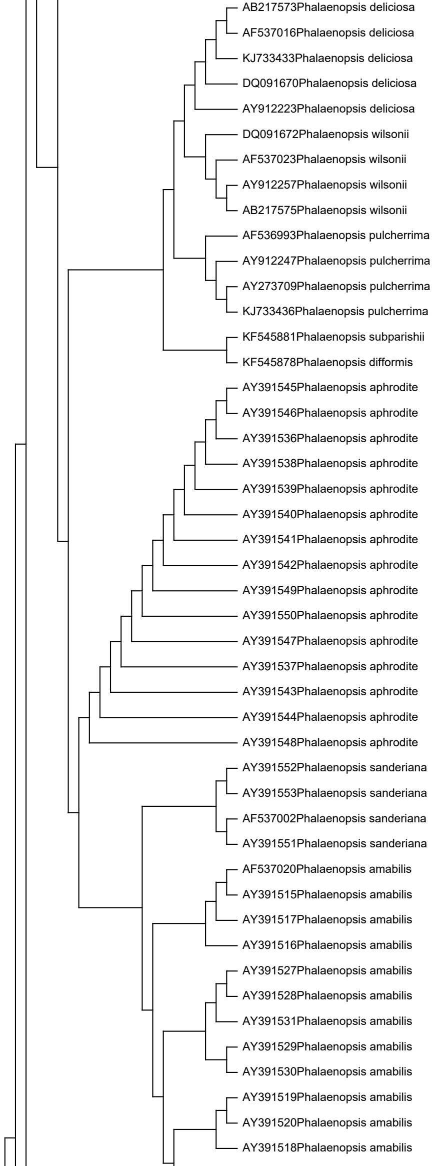
AB217573Phalaenopsis deliciosa
AF537016Phalaenopsis deliciosa
KJ733433Phalaenopsis deliciosa
DQ091670Phalaenopsis deliciosa
AY912223Phalaenopsis deliciosa
DQ091672Phalaenopsis wilsonii
AF537023Phalaenopsis wilsonii
AY912257Phalaenopsis wilsonii
AB217575Phalaenopsis wilsonii
AF536993Phalaenopsis pulcherrima
AY912247Phalaenopsis pulcherrima
AY273709Phalaenopsis pulcherrima
KJ733436Phalaenopsis pulcherrima
KF545881Phalaenopsis subparishii
KF545878Phalaenopsis difformis
AY391545Phalaenopsis aphrodite
AY391546Phalaenopsis aphrodite
AY391536Phalaenopsis aphrodite
AY391538Phalaenopsis aphrodite
AY391539Phalaenopsis aphrodite
AY391540Phalaenopsis aphrodite
AY391541Phalaenopsis aphrodite
AY391542Phalaenopsis aphrodite
AY391549Phalaenopsis aphrodite
AY391550Phalaenopsis aphrodite
AY391547Phalaenopsis aphrodite
AY391537Phalaenopsis aphrodite
AY391543Phalaenopsis aphrodite
AY391544Phalaenopsis aphrodite
AY391548Phalaenopsis aphrodite
AY391552Phalaenopsis sanderiana
AY391553Phalaenopsis sanderiana
AF537002Phalaenopsis sanderiana
AY391551Phalaenopsis sanderiana
AF537020Phalaenopsis amabilis
AY391515Phalaenopsis amabilis
AY391517Phalaenopsis amabilis
AY391516Phalaenopsis amabilis
AY391527Phalaenopsis amabilis
AY391528Phalaenopsis amabilis
AY391531Phalaenopsis amabilis
AY391529Phalaenopsis amabilis
AY391530Phalaenopsis amabilis
AY391519Phalaenopsis amabilis
AY391520Phalaenopsis amabilis
AY391518Phalaenopsis amabilis

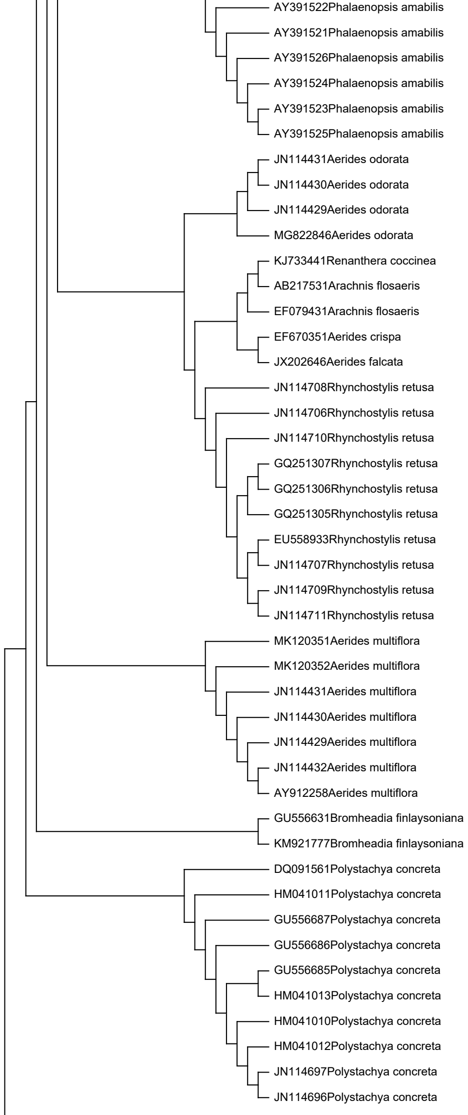
AY391522Phalaenopsis amabilis
AY391521Phalaenopsis amabilis
AY391526Phalaenopsis amabilis
AY391524Phalaenopsis amabilis
AY391523Phalaenopsis amabilis
AY391525Phalaenopsis amabilis
JN114431Aerides odorata
JN114430Aerides odorata
JN114429Aerides odorata
MG822846Aerides odorata
KJ733441Renanthera coccinea
AB217531Arachnis flosaeris
EF079431Arachnis flosaeris
EF670351Aerides crispa
JX202646Aerides falcata
JN114708Rhynchostylis retusa
JN114706Rhynchostylis retusa
JN114710Rhynchostylis retusa
GQ251307Rhynchostylis retusa
GQ251306Rhynchostylis retusa
GQ251305Rhynchostylis retusa
EU558933Rhynchostylis retusa
JN114707Rhynchostylis retusa
JN114709Rhynchostylis retusa
JN114711Rhynchostylis retusa
MK120351Aerides multiflora
MK120352Aerides multiflora
JN114431Aerides multiflora
JN114430Aerides multiflora
JN114429Aerides multiflora
JN114432Aerides multiflora
AY912258Aerides multiflora
GU556631Bromheadia finlaysoniana
KM921777Bromheadia finlaysoniana
DQ091561Polystachya concreta
HM041011Polystachya concreta
GU556687Polystachya concreta
GU556686Polystachya concreta
GU556685Polystachya concreta
HM041013Polystachya concreta
HM041010Polystachya concreta
HM041012Polystachya concreta
JN114697Polystachya concreta
JN114696Polystachya concreta

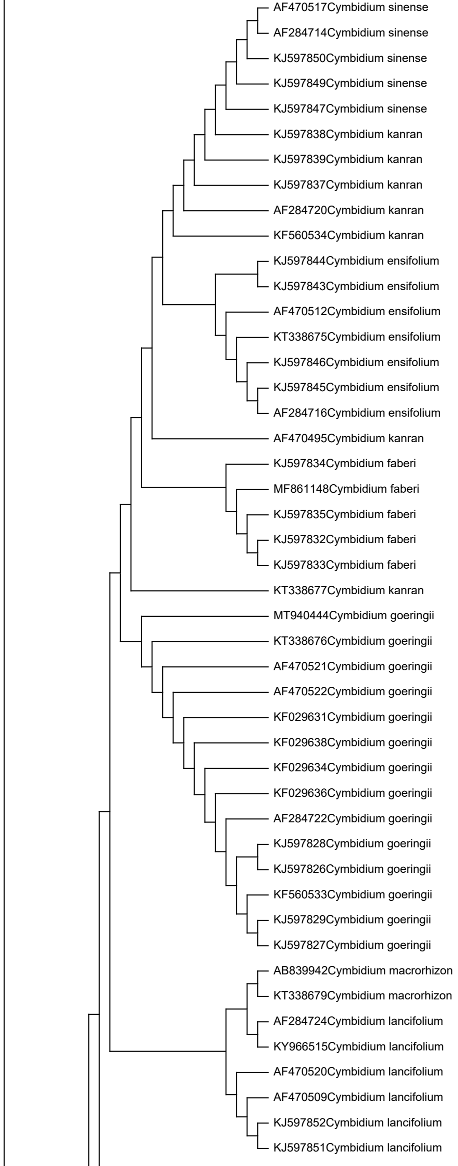
AF470517Cymbidium sinense
AF284714Cymbidium sinense
KJ597850Cymbidium sinense
KJ597849Cymbidium sinense
KJ597847Cymbidium sinense
KJ597838Cymbidium kanran
KJ597839Cymbidium kanran
KJ597837Cymbidium kanran
AF284720Cymbidium kanran
KF560534Cymbidium kanran
KJ597844Cymbidium ensifolium
KJ597843Cymbidium ensifolium
AF470512Cymbidium ensifolium
KT338675Cymbidium ensifolium
KJ597846Cymbidium ensifolium
KJ597845Cymbidium ensifolium
AF284716Cymbidium ensifolium
AF470495Cymbidium kanran
KJ597834Cymbidium faberi
MF861148Cymbidium faberi
KJ597835Cymbidium faberi
KJ597832Cymbidium faberi
KJ597833Cymbidium faberi
KT338677Cymbidium kanran
MT940444Cymbidium goeringii
KT338676Cymbidium goeringii
AF470521Cymbidium goeringii
AF470522Cymbidium goeringii
KF029631Cymbidium goeringii
KF029638Cymbidium goeringii
KF029634Cymbidium goeringii
KF029636Cymbidium goeringii
AF284722Cymbidium goeringii
KJ597828Cymbidium goeringii
KJ597826Cymbidium goeringii
KF560533Cymbidium goeringii
KJ597829Cymbidium goeringii
KJ597827Cymbidium goeringii
AB839942Cymbidium macrorhizon
KT338679Cymbidium macrorhizon
AF284724Cymbidium lancifolium
KY966515Cymbidium lancifolium
AF470520Cymbidium lancifolium
AF470509Cymbidium lancifolium
KJ597852Cymbidium lancifolium
KJ597851Cymbidium lancifolium

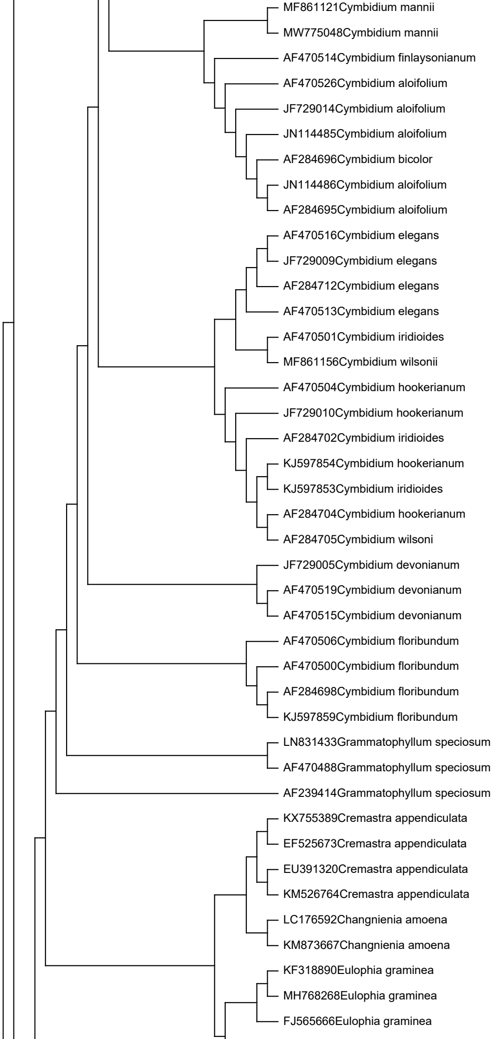
MF861121Cymbidium mannii
MW775048Cymbidium mannii
AF470514Cymbidium finlaysonianum
AF470526Cymbidium aloifolium
JF729014Cymbidium aloifolium
JN114485Cymbidium aloifolium
AF284696Cymbidium bicolor
JN114486Cymbidium aloifolium
AF284695Cymbidium aloifolium
AF470516Cymbidium elegans
JF729009Cymbidium elegans
AF284712Cymbidium elegans
AF470513Cymbidium elegans
AF470501Cymbidium iridioides
MF861156Cymbidium wilsonii
AF470504Cymbidium hookerianum
JF729010Cymbidium hookerianum
AF284702Cymbidium iridioides
KJ597854Cymbidium hookerianum
KJ597853Cymbidium iridioides
AF284704Cymbidium hookerianum
AF284705Cymbidium wilsoni
JF729005Cymbidium devonianum
AF470519Cymbidium devonianum
AF470515Cymbidium devonianum
AF470506Cymbidium floribundum
AF470500Cymbidium floribundum
AF284698Cymbidium floribundum
KJ597859Cymbidium floribundum
LN831433Grammatophyllum speciosum
AF470488Grammatophyllum speciosum
AF239414Grammatophyllum speciosum
KX755389Cremastra appendiculata
EF525673Cremastra appendiculata
EU391320Cremastra appendiculata
KM526764Cremastra appendiculata
LC176592Changnienia amoena
KM873667Changnienia amoena
KF318890Eulophia graminea
MH768268Eulophia graminea
FJ565666Eulophia graminea

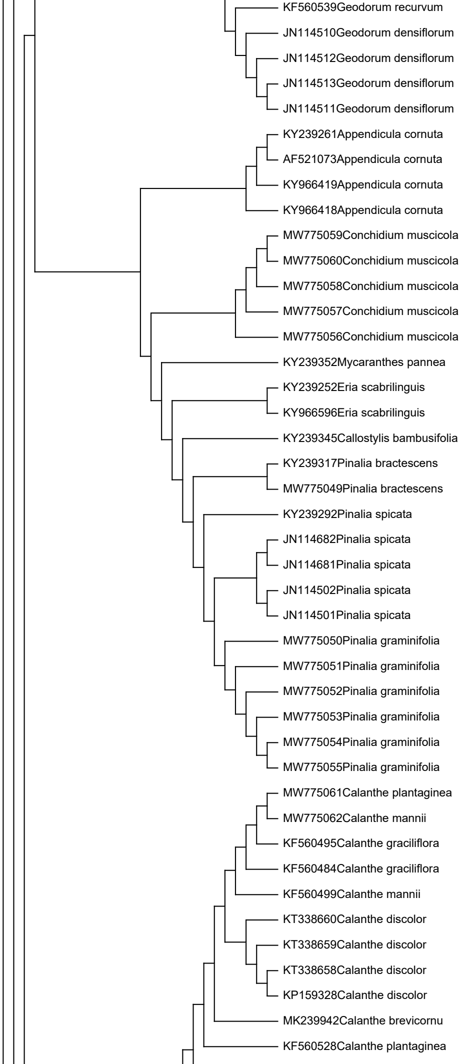
KF560539Geodorum recurvum
JN114510Geodorum densiflorum
JN114512Geodorum densiflorum
JN114513Geodorum densiflorum
JN114511Geodorum densiflorum
KY239261Appendicula cornuta
AF521073Appendicula cornuta
KY966419Appendicula cornuta
KY966418Appendicula cornuta
MW775059Conchidium muscicola
MW775060Conchidium muscicola
MW775058Conchidium muscicola
MW775057Conchidium muscicola
MW775056Conchidium muscicola
KY239352Mycaranthes pannea
KY239252Eria scabrilinguis
KY966596Eria scabrilinguis
KY239345Callostylis bambusifolia
KY239317Pinalia bractescens
MW775049Pinalia bractescens
KY239292Pinalia spicata
JN114682Pinalia spicata
JN114681Pinalia spicata
JN114502Pinalia spicata
JN114501Pinalia spicata
MW775050Pinalia graminifolia
MW775051Pinalia graminifolia
MW775052Pinalia graminifolia
MW775053Pinalia graminifolia
MW775054Pinalia graminifolia
MW775055Pinalia graminifolia
MW775061Calanthe plantaginea
MW775062Calanthe mannii
KF560495Calanthe graciliflora
KF560484Calanthe graciliflora
KF560499Calanthe mannii
KT338660Calanthe discolor
KT338659Calanthe discolor
KT338658Calanthe discolor
KP159328Calanthe discolor
MK239942Calanthe brevicornu
KF560528Calanthe plantaginea

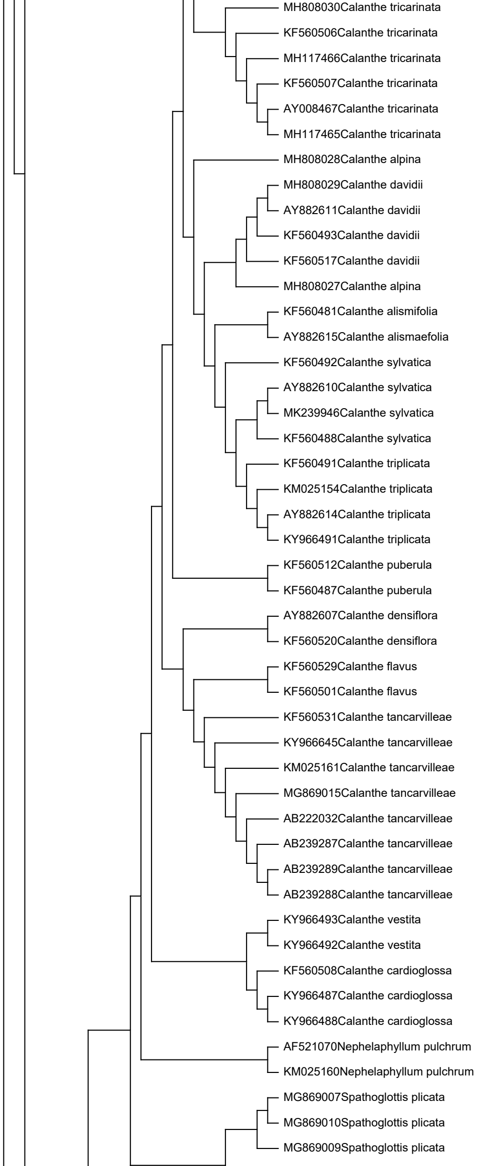
MH808030Calanthe tricarinata
KF560506Calanthe tricarinata
MH117466Calanthe tricarinata
KF560507Calanthe tricarinata
AY008467Calanthe tricarinata
MH117465Calanthe tricarinata
MH808028Calanthe alpina
MH808029Calanthe davidii
AY882611Calanthe davidii
KF560493Calanthe davidii
KF560517Calanthe davidii
MH808027Calanthe alpina
KF560481Calanthe alismifolia
AY882615Calanthe alismaefolia
KF560492Calanthe sylvatica
AY882610Calanthe sylvatica
MK239946Calanthe sylvatica
KF560488Calanthe sylvatica
KF560491Calanthe triplicata
KM025154Calanthe triplicata
AY882614Calanthe triplicata
KY966491Calanthe triplicata
KF560512Calanthe puberula
KF560487Calanthe puberula
AY882607Calanthe densiflora
KF560520Calanthe densiflora
KF560529Calanthe flavus
KF560501Calanthe flavus
KF560531Calanthe tancarvilleae
KY966645Calanthe tancarvilleae
KM025161Calanthe tancarvilleae
MG869015Calanthe tancarvilleae
AB222032Calanthe tancarvilleae
AB239287Calanthe tancarvilleae
AB239289Calanthe tancarvilleae
AB239288Calanthe tancarvilleae
KY966493Calanthe vestita
KY966492Calanthe vestita
KF560508Calanthe cardioglossa
KY966487Calanthe cardioglossa
KY966488Calanthe cardioglossa
AF521070Nephelaphyllum pulchrum
KM025160Nephelaphyllum pulchrum
MG869007Spathoglottis plicata
MG869010Spathoglottis plicata
MG869009Spathoglottis plicata

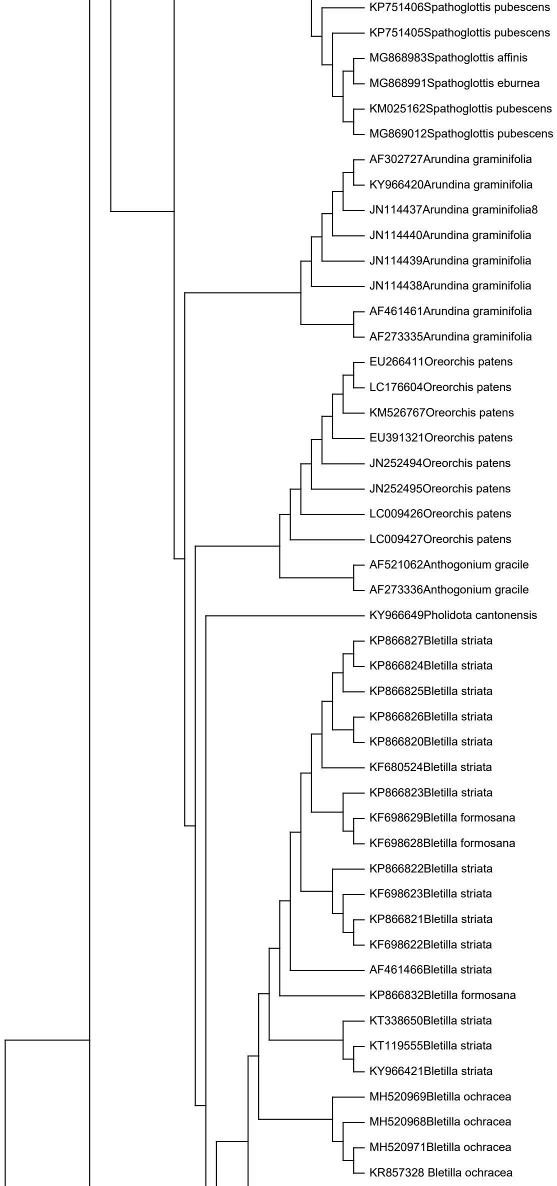
KP751406Spathoglottis pubescens
KP751405Spathoglottis pubescens
MG868983Spathoglottis affinis
MG868991Spathoglottis eburnea
KM025162Spathoglottis pubescens
MG869012Spathoglottis pubescens
AF302727Arundina graminifolia
KY966420Arundina graminifolia
JN114437Arundina graminifolia8
JN114440Arundina graminifolia
JN114439Arundina graminifolia
JN114438Arundina graminifolia
AF461461Arundina graminifolia
AF273335Arundina graminifolia
EU266411Oreorchis patens
LC176604Oreorchis patens
KM526767Oreorchis patens
EU391321Oreorchis patens
JN252494Oreorchis patens
JN252495Oreorchis patens
LC009426Oreorchis patens
LC009427Oreorchis patens
AF521062Anthogonium gracile
AF273336Anthogonium gracile
KY966649Pholidota cantonensis
KP866827Bletilla striata
KP866824Bletilla striata
KP866825Bletilla striata
KP866826Bletilla striata
KP866820Bletilla striata
KF680524Bletilla striata
KP866823Bletilla striata
KF698629Bletilla formosana
KF698628Bletilla formosana
KP866822Bletilla striata
KF698623Bletilla striata
KP866821Bletilla striata
KF698622Bletilla striata
AF461466Bletilla striata
KP866832Bletilla formosana
KT338650Bletilla striata
KT119555Bletilla striata
KY966421Bletilla striata
MH520969Bletilla ochracea
MH520968Bletilla ochracea
MH520971Bletilla ochracea
KR857328 Bletilla ochracea

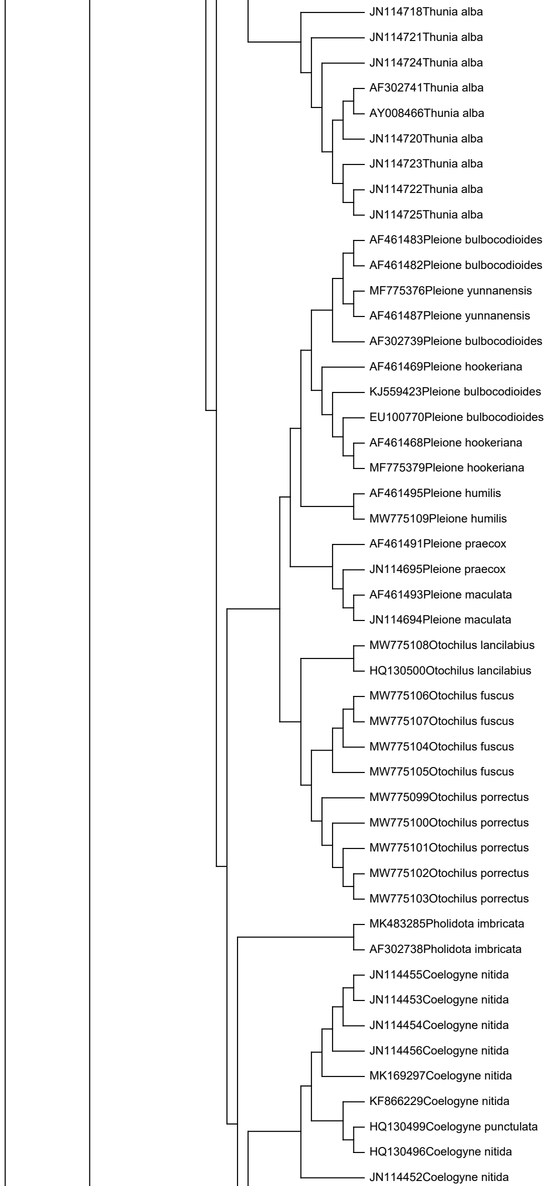
JN114718Thunia alba
JN114721Thunia alba
JN114724Thunia alba
AF302741Thunia alba
AY008466Thunia alba
JN114720Thunia alba
JN114723Thunia alba
JN114722Thunia alba
JN114725Thunia alba
AF461483Pleione bulbocodioides
AF461482Pleione bulbocodioides
MF775376Pleione yunnanensis
AF461487Pleione yunnanensis
AF302739Pleione bulbocodioides
AF461469Pleione hookeriana
KJ559423Pleione bulbocodioides
EU100770Pleione bulbocodioides
AF461468Pleione hookeriana
MF775379Pleione hookeriana
AF461495Pleione humilis
MW775109Pleione humilis
AF461491Pleione praecox
JN114695Pleione praecox
AF461493Pleione maculata
JN114694Pleione maculata
MW775108Otochilus lancilabius
HQ130500Otochilus lancilabius
MW775106Otochilus fuscus
MW775107Otochilus fuscus
MW775104Otochilus fuscus
MW775105Otochilus fuscus
MW775099Otochilus porrectus
MW775100Otochilus porrectus
MW775101Otochilus porrectus
MW775102Otochilus porrectus
MW775103Otochilus porrectus
MK483285Pholidota imbricata
AF302738Pholidota imbricata
JN114455Coelogyne nitida
JN114453Coelogyne nitida
JN114454Coelogyne nitida
JN114456Coelogyne nitida
MK169297Coelogyne nitida
KF866229Coelogyne nitida
HQ130499Coelogyne punctulata
HQ130496Coelogyne nitida
JN114452Coelogyne nitida

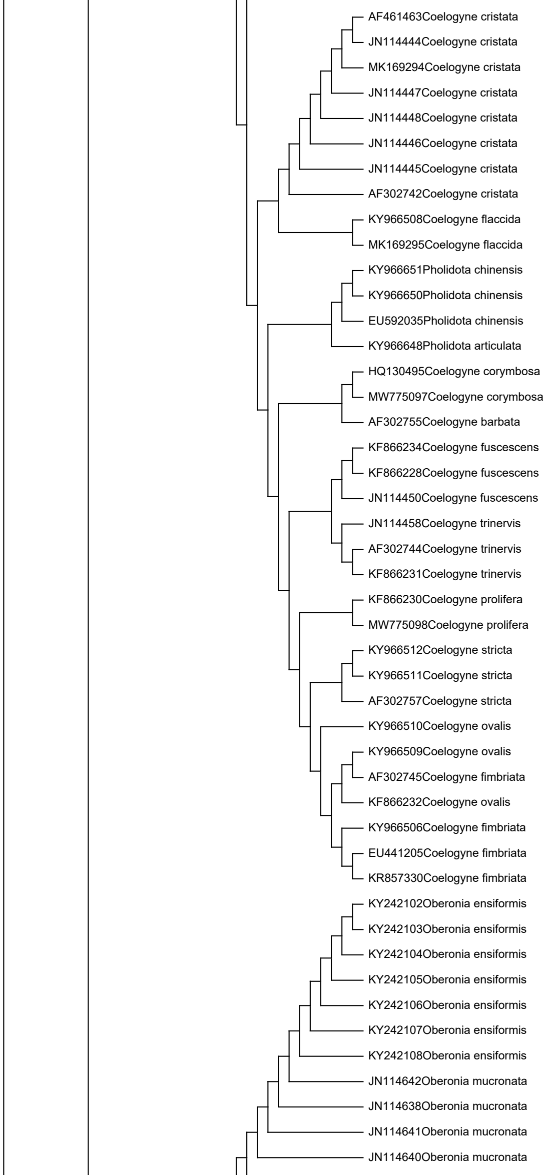
AF461463Coelogyne cristata
JN114444Coelogyne cristata
MK169294Coelogyne cristata
JN114447Coelogyne cristata
JN114448Coelogyne cristata
JN114446Coelogyne cristata
JN114445Coelogyne cristata
AF302742Coelogyne cristata
KY966508Coelogyne flaccida
MK169295Coelogyne flaccida
KY966651Pholidota chinensis
KY966650Pholidota chinensis
EU592035Pholidota chinensis
KY966648Pholidota articulata
HQ130495Coelogyne corymbosa
MW775097Coelogyne corymbosa
AF302755Coelogyne barbata
KF866234Coelogyne fuscescens
KF866228Coelogyne fuscescens
JN114450Coelogyne fuscescens
JN114458Coelogyne trinervis
AF302744Coelogyne trinervis
KF866231Coelogyne trinervis
KF866230Coelogyne prolifera
MW775098Coelogyne prolifera
KY966512Coelogyne stricta
KY966511Coelogyne stricta
AF302757Coelogyne stricta
KY966510Coelogyne ovalis
KY966509Coelogyne ovalis
AF302745Coelogyne fimbriata
KF866232Coelogyne ovalis
KY966506Coelogyne fimbriata
EU441205Coelogyne fimbriata
KR857330Coelogyne fimbriata
KY242102Oberonia ensiformis
KY242103Oberonia ensiformis
KY242104Oberonia ensiformis
KY242105Oberonia ensiformis
KY242106Oberonia ensiformis
KY242107Oberonia ensiformis
KY242108Oberonia ensiformis
JN114642Oberonia mucronata
JN114638Oberonia mucronata
JN114641Oberonia mucronata
JN114640Oberonia mucronata

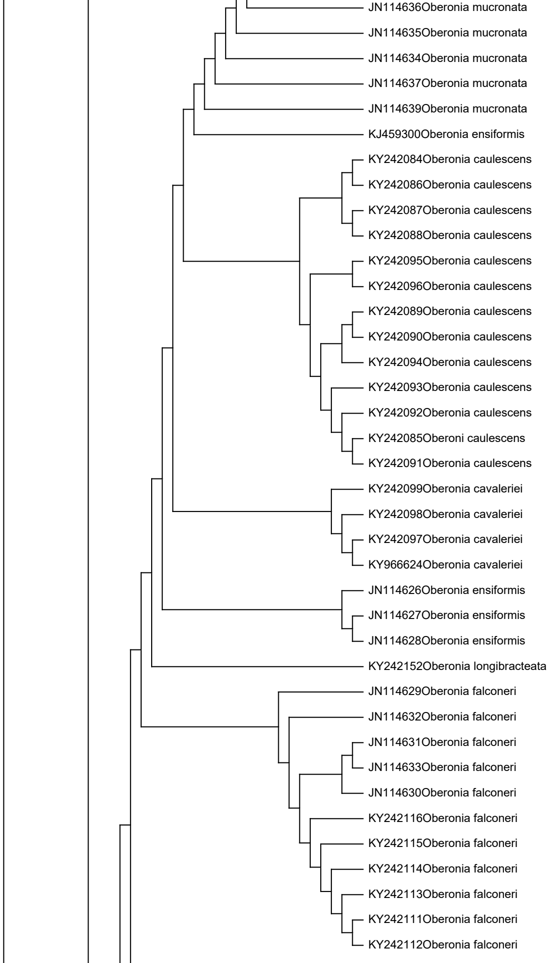
JN114636Oberonia mucronata
JN114635Oberonia mucronata
JN114634Oberonia mucronata
JN114637Oberonia mucronata
JN114639Oberonia mucronata
KJ459300Oberonia ensiformis
KY242084Oberonia caulescens
KY242086Oberonia caulescens
KY242087Oberonia caulescens
KY242088Oberonia caulescens
KY242095Oberonia caulescens
KY242096Oberonia caulescens
KY242089Oberonia caulescens
KY242090Oberonia caulescens
KY242094Oberonia caulescens
KY242093Oberonia caulescens
KY242092Oberonia caulescens
KY242085Oberoni caulescens
KY242091Oberonia caulescens
KY242099Oberonia cavaleriei
KY242098Oberonia cavaleriei
KY242097Oberonia cavaleriei
KY966624Oberonia cavaleriei
JN114626Oberonia ensiformis
JN114627Oberonia ensiformis
JN114628Oberonia ensiformis
KY242152Oberonia longibracteata
JN114629Oberonia falconeri
JN114632Oberonia falconeri
JN114631Oberonia falconeri
JN114633Oberonia falconeri
JN114630Oberonia falconeri
KY242116Oberonia falconeri
KY242115Oberonia falconeri
KY242114Oberonia falconeri
KY242113Oberonia falconeri
KY242111Oberonia falconeri
KY242112Oberonia falconeri

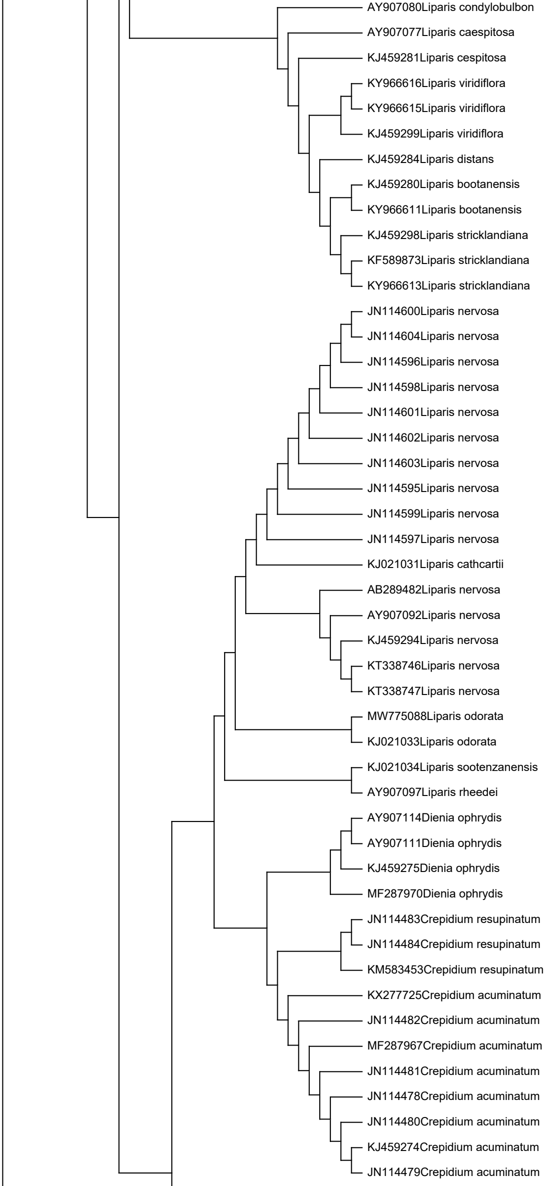
AY907080Liparis condylobulbon
AY907077Liparis caespitosa
KJ459281Liparis cespitosa
KY966616Liparis viridiflora
KY966615Liparis viridiflora
KJ459299Liparis viridiflora
KJ459284Liparis distans
KJ459280Liparis bootanensis
KY966611Liparis bootanensis
KJ459298Liparis stricklandiana
KF589873Liparis stricklandiana
KY966613Liparis stricklandiana
JN114600Liparis nervosa
JN114604Liparis nervosa
JN114596Liparis nervosa
JN114598Liparis nervosa
JN114601Liparis nervosa
JN114602Liparis nervosa
JN114603Liparis nervosa
JN114595Liparis nervosa
JN114599Liparis nervosa
JN114597Liparis nervosa
KJ021031Liparis cathcartii
AB289482Liparis nervosa
AY907092Liparis nervosa
KJ459294Liparis nervosa
KT338746Liparis nervosa
KT338747Liparis nervosa
MW775088Liparis odorata
KJ021033Liparis odorata
KJ021034Liparis sootenzanensis
AY907097Liparis rheedei
AY907114Dienia ophrydis
AY907111Dienia ophrydis
KJ459275Dienia ophrydis
MF287970Dienia ophrydis
JN114483Crepidium resupinatum
JN114484Crepidium resupinatum
KM583453Crepidium resupinatum
KX277725Crepidium acuminatum
JN114482Crepidium acuminatum
MF287967Crepidium acuminatum
JN114481Crepidium acuminatum
JN114478Crepidium acuminatum
JN114480Crepidium acuminatum
KJ459274Crepidium acuminatum
JN114479Crepidium acuminatum

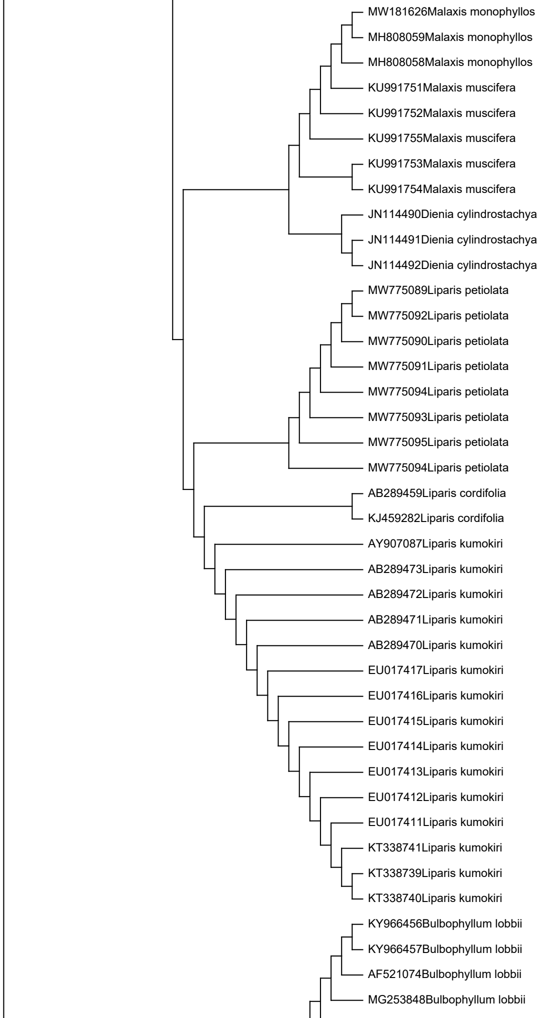
MW181626Malaxis monophyllos
MH808059Malaxis monophyllos
MH808058Malaxis monophyllos
KU991751Malaxis muscifera
KU991752Malaxis muscifera
KU991755Malaxis muscifera
KU991753Malaxis muscifera
KU991754Malaxis muscifera
JN114490Dienia cylindrostachya
JN114491Dienia cylindrostachya
JN114492Dienia cylindrostachya
MW775089Liparis petiolata
MW775092Liparis petiolata
MW775090Liparis petiolata
MW775091Liparis petiolata
MW775094Liparis petiolata
MW775093Liparis petiolata
MW775095Liparis petiolata
MW775094Liparis petiolata
AB289459Liparis cordifolia
KJ459282Liparis cordifolia
AY907087Liparis kumokiri
AB289473Liparis kumokiri
AB289472Liparis kumokiri
AB289471Liparis kumokiri
AB289470Liparis kumokiri
EU017417Liparis kumokiri
EU017416Liparis kumokiri
EU017415Liparis kumokiri
EU017414Liparis kumokiri
EU017413Liparis kumokiri
EU017412Liparis kumokiri
EU017411Liparis kumokiri
KT338741Liparis kumokiri
KT338739Liparis kumokiri
KT338740Liparis kumokiri
KY966456Bulbophyllum lobbii
KY966457Bulbophyllum lobbii
AF521074Bulbophyllum lobbii
MG253848Bulbophyllum lobbii

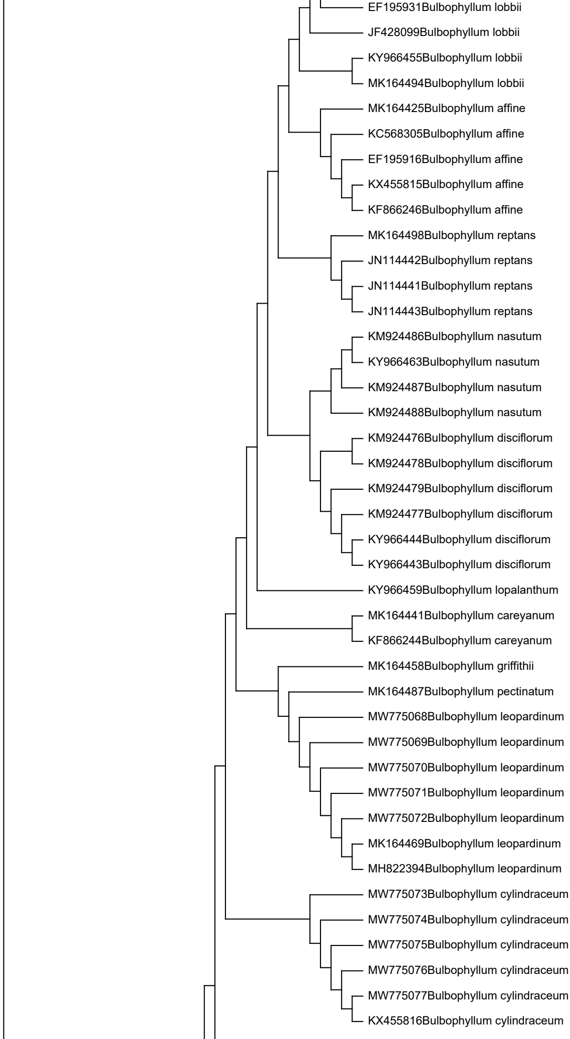
EF195931Bulbophyllum lobbii
JF428099Bulbophyllum lobbii
KY966455Bulbophyllum lobbii
MK164494Bulbophyllum lobbii
MK164425Bulbophyllum affine
KC568305Bulbophyllum affine
EF195916Bulbophyllum affine
KX455815Bulbophyllum affine
KF866246Bulbophyllum affine
MK164498Bulbophyllum reptans
JN114442Bulbophyllum reptans
JN114441Bulbophyllum reptans
JN114443Bulbophyllum reptans
KM924486Bulbophyllum nasutum
KY966463Bulbophyllum nasutum
KM924487Bulbophyllum nasutum
KM924488Bulbophyllum nasutum
KM924476Bulbophyllum disciflorum
KM924478Bulbophyllum disciflorum
KM924479Bulbophyllum disciflorum
KM924477Bulbophyllum disciflorum
KY966444Bulbophyllum disciflorum
KY966443Bulbophyllum disciflorum
KY966459Bulbophyllum lopalanthum
MK164441Bulbophyllum careyanum
KF866244Bulbophyllum careyanum
MK164458Bulbophyllum griffithii
MK164487Bulbophyllum pectinatum
MW775068Bulbophyllum leopardinum
MW775069Bulbophyllum leopardinum
MW775070Bulbophyllum leopardinum
MW775071Bulbophyllum leopardinum
MW775072Bulbophyllum leopardinum
MK164469Bulbophyllum leopardinum
MH822394Bulbophyllum leopardinum
MW775073Bulbophyllum cylindraceum
MW775074Bulbophyllum cylindraceum
MW775075Bulbophyllum cylindraceum
MW775076Bulbophyllum cylindraceum
MW775077Bulbophyllum cylindraceum
KX455816Bulbophyllum cylindraceum

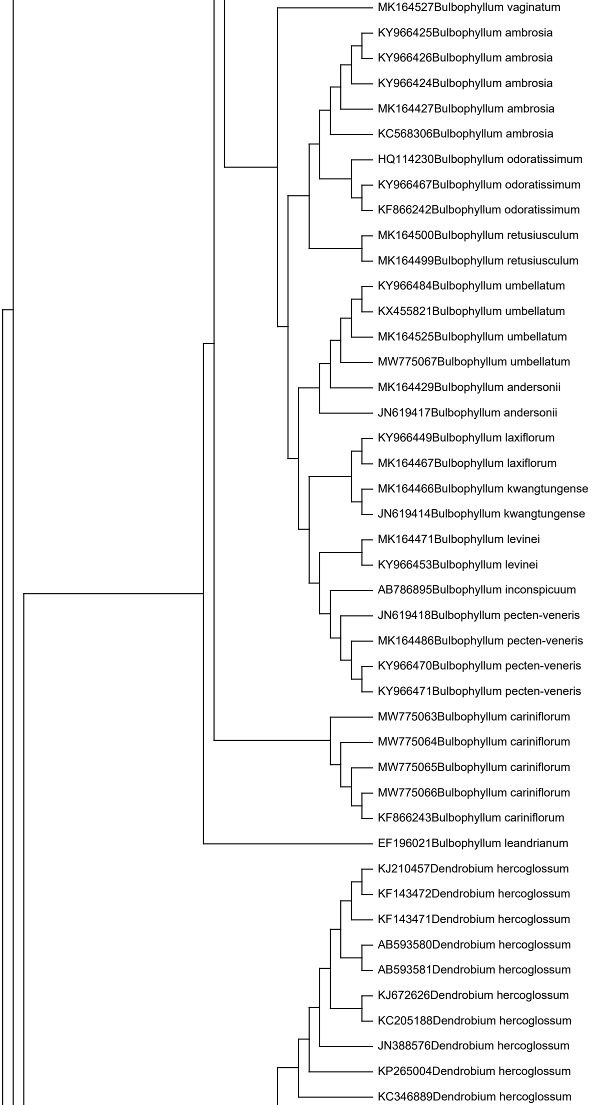
MK164527Bulbophyllum vaginatum
KY966425Bulbophyllum ambrosia
KY966426Bulbophyllum ambrosia
KY966424Bulbophyllum ambrosia
MK164427Bulbophyllum ambrosia
KC568306Bulbophyllum ambrosia
HQ114230Bulbophyllum odoratissimum
KY966467Bulbophyllum odoratissimum
KF866242Bulbophyllum odoratissimum
MK164500Bulbophyllum retusiusculum
MK164499Bulbophyllum retusiusculum
KY966484Bulbophyllum umbellatum
KX455821Bulbophyllum umbellatum
MK164525Bulbophyllum umbellatum
MW775067Bulbophyllum umbellatum
MK164429Bulbophyllum andersonii
JN619417Bulbophyllum andersonii
KY966449Bulbophyllum laxiflorum
MK164467Bulbophyllum laxiflorum
MK164466Bulbophyllum kwangtungense
JN619414Bulbophyllum kwangtungense
MK164471Bulbophyllum levinei
KY966453Bulbophyllum levinei
AB786895Bulbophyllum inconspicuum
JN619418Bulbophyllum pecten-veneris
MK164486Bulbophyllum pecten-veneris
KY966470Bulbophyllum pecten-veneris
KY966471Bulbophyllum pecten-veneris
MW775063Bulbophyllum cariniflorum
MW775064Bulbophyllum cariniflorum
MW775065Bulbophyllum cariniflorum
MW775066Bulbophyllum cariniflorum
KF866243Bulbophyllum cariniflorum
EF196021Bulbophyllum leandrianum
KJ210457Dendrobium hercoglossum
KF143472Dendrobium hercoglossum
KF143471Dendrobium hercoglossum
AB593580Dendrobium hercoglossum
AB593581Dendrobium hercoglossum
KJ672626Dendrobium hercoglossum
KC205188Dendrobium hercoglossum
JN388576Dendrobium hercoglossum
KP265004Dendrobium hercoglossum
KC346889Dendrobium hercoglossum

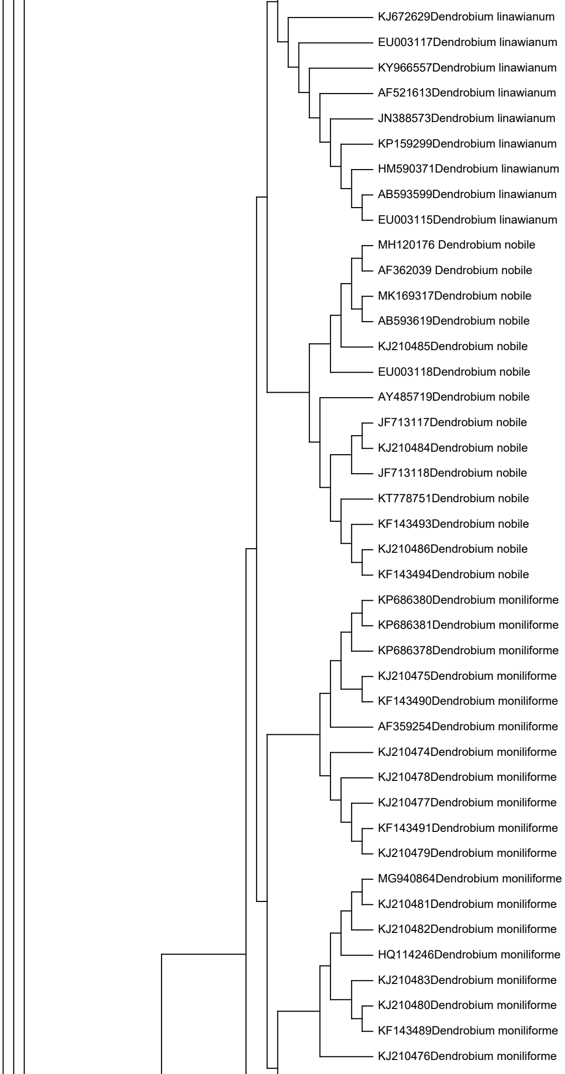

KJ672629Dendrobium linawianum
EU003117Dendrobium linawianum
KY966557Dendrobium linawianum
AF521613Dendrobium linawianum
JN388573Dendrobium linawianum
KP159299Dendrobium linawianum
HM590371Dendrobium linawianum
AB593599Dendrobium linawianum
EU003115Dendrobium linawianum
MH120176 Dendrobium nobile
AF362039 Dendrobium nobile
MK169317Dendrobium nobile
AB593619Dendrobium nobile
KJ210485Dendrobium nobile
EU003118Dendrobium nobile
AY485719Dendrobium nobile
JF713117Dendrobium nobile
KJ210484Dendrobium nobile
JF713118Dendrobium nobile
KT778751Dendrobium nobile
KF143493Dendrobium nobile
KJ210486Dendrobium nobile
KF143494Dendrobium nobile
KP686380Dendrobium moniliforme
KP686381Dendrobium moniliforme
KP686378Dendrobium moniliforme
KJ210475Dendrobium moniliforme
KF143490Dendrobium moniliforme
AF359254Dendrobium moniliforme
KJ210474Dendrobium moniliforme
KJ210478Dendrobium moniliforme
KJ210477Dendrobium moniliforme
KF143491Dendrobium moniliforme
KJ210479Dendrobium moniliforme
MG940864Dendrobium moniliforme
KJ210481Dendrobium moniliforme
KJ210482Dendrobium moniliforme
HQ114246Dendrobium moniliforme
KJ210483Dendrobium moniliforme
KJ210480Dendrobium moniliforme
KF143489Dendrobium moniliforme
KJ210476Dendrobium moniliforme

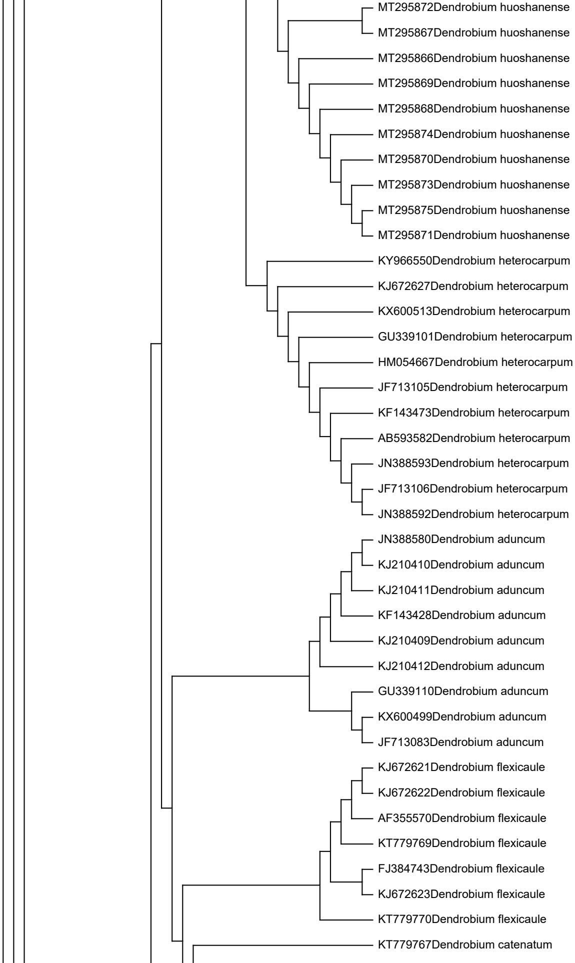
MT295872Dendrobium huoshanense
MT295867Dendrobium huoshanense
MT295866Dendrobium huoshanense
MT295869Dendrobium huoshanense
MT295868Dendrobium huoshanense
MT295874Dendrobium huoshanense
MT295870Dendrobium huoshanense
MT295873Dendrobium huoshanense
MT295875Dendrobium huoshanense
MT295871Dendrobium huoshanense
KY966550Dendrobium heterocarpum
KJ672627Dendrobium heterocarpum
KX600513Dendrobium heterocarpum
GU339101Dendrobium heterocarpum
HM054667Dendrobium heterocarpum
JF713105Dendrobium heterocarpum
KF143473Dendrobium heterocarpum
AB593582Dendrobium heterocarpum
JN388593Dendrobium heterocarpum
JF713106Dendrobium heterocarpum
JN388592Dendrobium heterocarpum
JN388580Dendrobium aduncum
KJ210410Dendrobium aduncum
KJ210411Dendrobium aduncum
KF143428Dendrobium aduncum
KJ210409Dendrobium aduncum
KJ210412Dendrobium aduncum
GU339110Dendrobium aduncum
KX600499Dendrobium aduncum
JF713083Dendrobium aduncum
KJ672621Dendrobium flexicaule
KJ672622Dendrobium flexicaule
AF355570Dendrobium flexicaule
KT779769Dendrobium flexicaule
FJ384743Dendrobium flexicaule
KJ672623Dendrobium flexicaule
KT779770Dendrobium flexicaule
KT779767Dendrobium catenatum

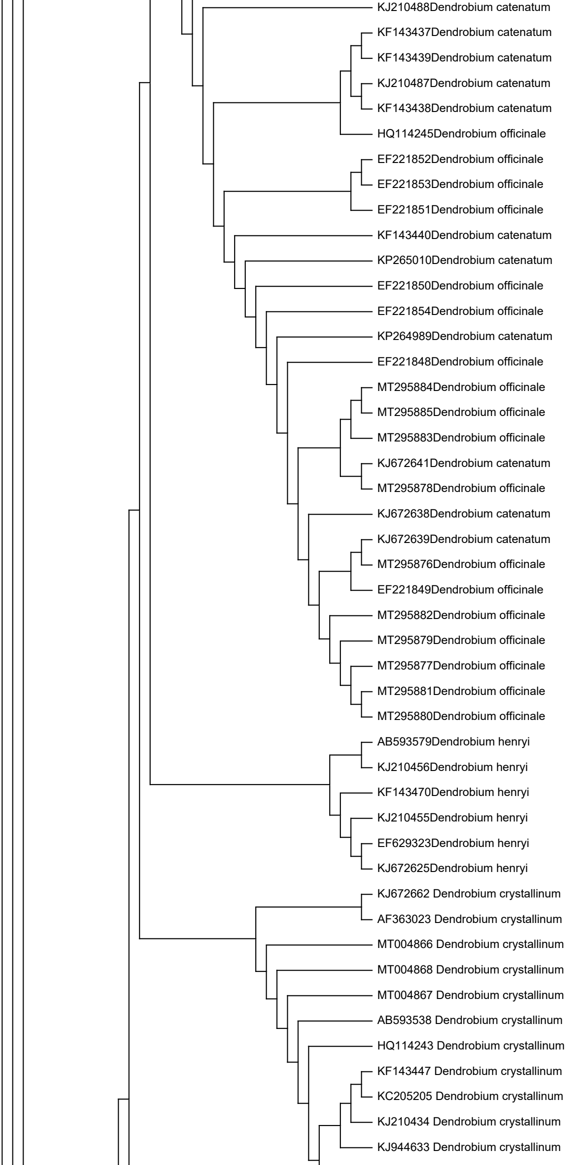
KJ210488Dendrobium catenatum
KF143437Dendrobium catenatum
KF143439Dendrobium catenatum
KJ210487Dendrobium catenatum
KF143438Dendrobium catenatum
HQ114245Dendrobium officinale
EF221852Dendrobium officinale
EF221853Dendrobium officinale
EF221851Dendrobium officinale
KF143440Dendrobium catenatum
KP265010Dendrobium catenatum
EF221850Dendrobium officinale
EF221854Dendrobium officinale
KP264989Dendrobium catenatum
EF221848Dendrobium officinale
MT295884Dendrobium officinale
MT295885Dendrobium officinale
MT295883Dendrobium officinale
KJ672641Dendrobium catenatum
MT295878Dendrobium officinale
KJ672638Dendrobium catenatum
KJ672639Dendrobium catenatum
MT295876Dendrobium officinale
EF221849Dendrobium officinale
MT295882Dendrobium officinale
MT295879Dendrobium officinale
MT295877Dendrobium officinale
MT295881Dendrobium officinale
MT295880Dendrobium officinale
AB593579Dendrobium henryi
KJ210456Dendrobium henryi
KF143470Dendrobium henryi
KJ210455Dendrobium henryi
EF629323Dendrobium henryi
KJ672625Dendrobium henryi
KJ672662 Dendrobium crystallinum
AF363023 Dendrobium crystallinum
MT004866 Dendrobium crystallinum
MT004868 Dendrobium crystallinum
MT004867 Dendrobium crystallinum
AB593538 Dendrobium crystallinum
HQ114243 Dendrobium crystallinum
KF143447 Dendrobium crystallinum
KC205205 Dendrobium crystallinum
KJ210434 Dendrobium crystallinum
KJ944633 Dendrobium crystallinum

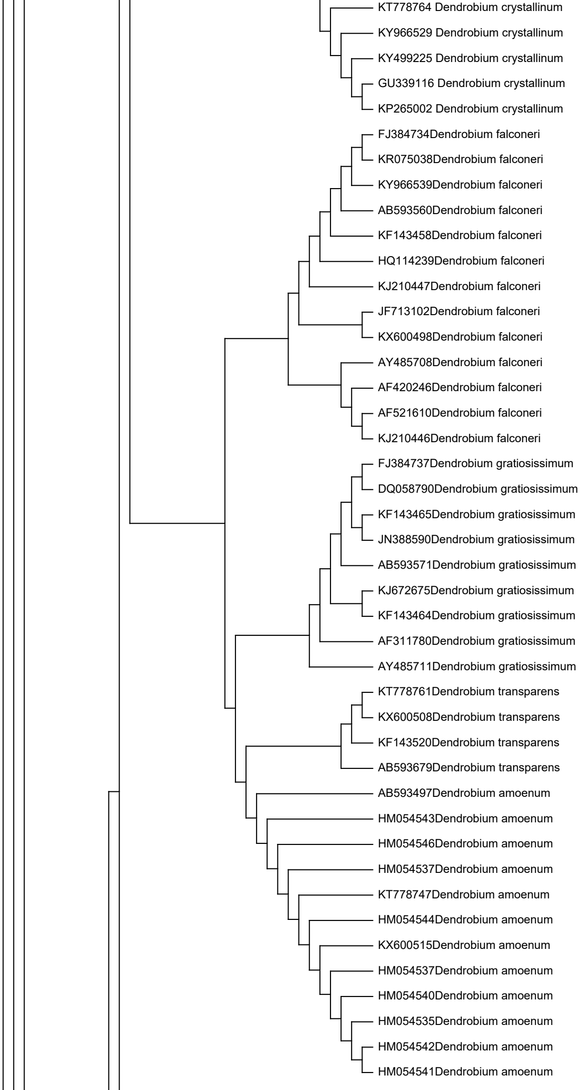
KT778764 Dendrobium crystallinum
KY966529 Dendrobium crystallinum
KY499225 Dendrobium crystallinum
GU339116 Dendrobium crystallinum
KP265002 Dendrobium crystallinum
FJ384734Dendrobium falconeri
KR075038Dendrobium falconeri
KY966539Dendrobium falconeri
AB593560Dendrobium falconeri
KF143458Dendrobium falconeri
HQ114239Dendrobium falconeri
KJ210447Dendrobium falconeri
JF713102Dendrobium falconeri
KX600498Dendrobium falconeri
AY485708Dendrobium falconeri
AF420246Dendrobium falconeri
AF521610Dendrobium falconeri
KJ210446Dendrobium falconeri
FJ384737Dendrobium gratiosissimum
DQ058790Dendrobium gratiosissimum
KF143465Dendrobium gratiosissimum
JN388590Dendrobium gratiosissimum
AB593571Dendrobium gratiosissimum
KJ672675Dendrobium gratiosissimum
KF143464Dendrobium gratiosissimum
AF311780Dendrobium gratiosissimum
AY485711Dendrobium gratiosissimum
KT778761Dendrobium transparens
KX600508Dendrobium transparens
KF143520Dendrobium transparens
AB593679Dendrobium transparens
AB593497Dendrobium amoenum
HM054543Dendrobium amoenum
HM054546Dendrobium amoenum
HM054537Dendrobium amoenum
KT778747Dendrobium amoenum
HM054544Dendrobium amoenum
KX600515Dendrobium amoenum
HM054537Dendrobium amoenum
HM054540Dendrobium amoenum
HM054535Dendrobium amoenum
HM054542Dendrobium amoenum
HM054541Dendrobium amoenum

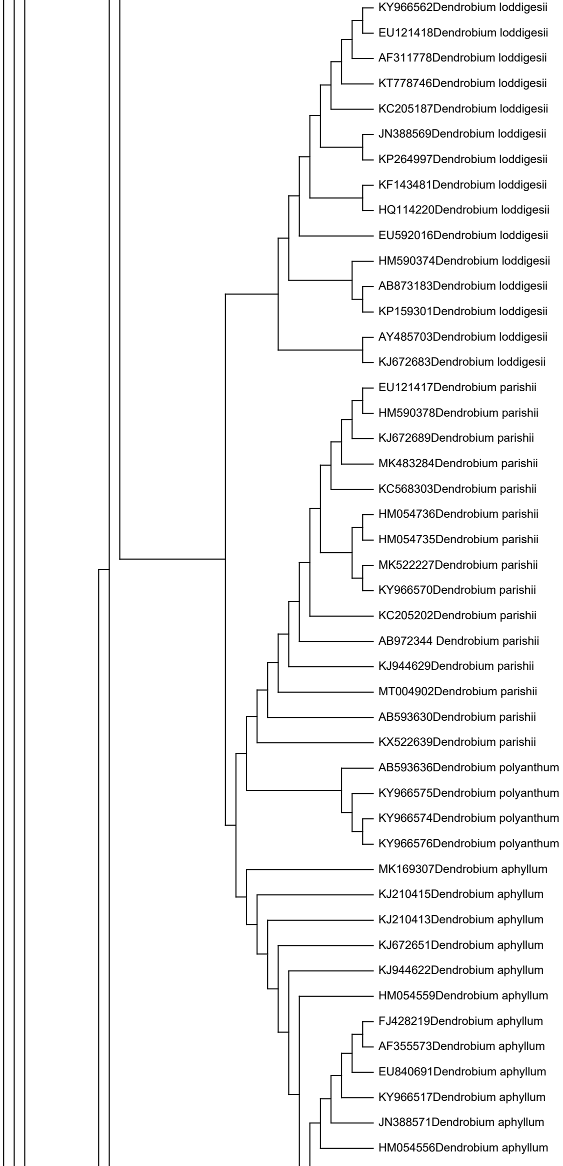

KY966562Dendrobium loddigesii
EU121418Dendrobium loddigesii
AF311778Dendrobium loddigesii
KT778746Dendrobium loddigesii
KC205187Dendrobium loddigesii
JN388569Dendrobium loddigesii
KP264997Dendrobium loddigesii
KF143481Dendrobium loddigesii
HQ114220Dendrobium loddigesii
EU592016Dendrobium loddigesii
HM590374Dendrobium loddigesii
AB873183Dendrobium loddigesii
KP159301Dendrobium loddigesii
AY485703Dendrobium loddigesii
KJ672683Dendrobium loddigesii
EU121417Dendrobium parishii
HM590378Dendrobium parishii
KJ672689Dendrobium parishii
MK483284Dendrobium parishii
KC568303Dendrobium parishii
HM054736Dendrobium parishii
HM054735Dendrobium parishii
MK522227Dendrobium parishii
KY966570Dendrobium parishii
KC205202Dendrobium parishii
AB972344 Dendrobium parishii
KJ944629Dendrobium parishii
MT004902Dendrobium parishii
AB593630Dendrobium parishii
KX522639Dendrobium parishii
AB593636Dendrobium polyanthum
KY966575Dendrobium polyanthum
KY966574Dendrobium polyanthum
KY966576Dendrobium polyanthum
MK169307Dendrobium aphyllum
KJ210415Dendrobium aphyllum
KJ210413Dendrobium aphyllum
KJ672651Dendrobium aphyllum
KJ944622Dendrobium aphyllum
HM054559Dendrobium aphyllum
FJ428219Dendrobium aphyllum
AF355573Dendrobium aphyllum
EU840691Dendrobium aphyllum
KY966517Dendrobium aphyllum
JN388571Dendrobium aphyllum
HM054556Dendrobium aphyllum

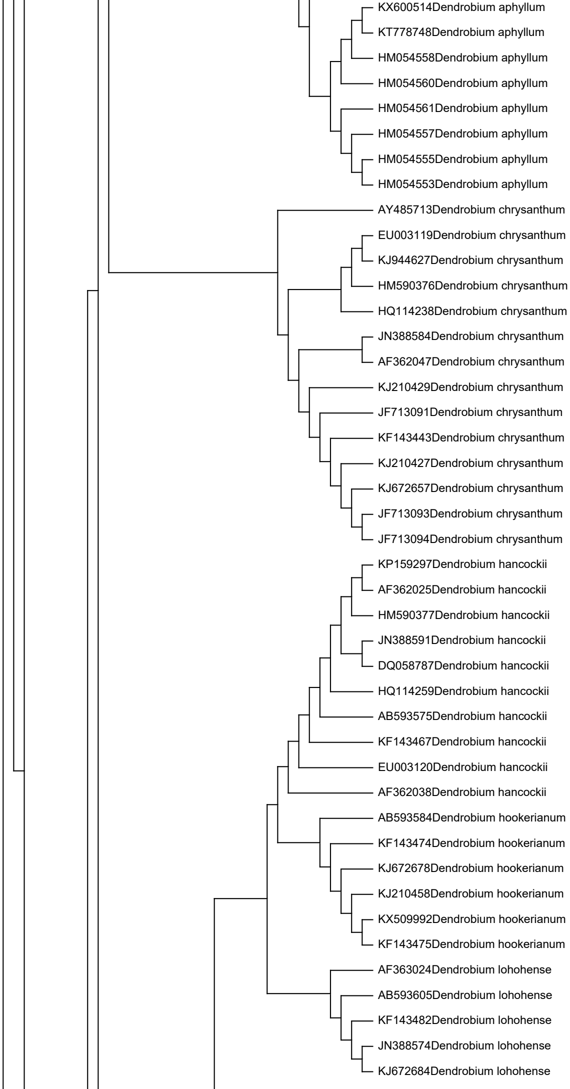
KX600514Dendrobium aphyllum
KT778748Dendrobium aphyllum
HM054558Dendrobium aphyllum
HM054560Dendrobium aphyllum
HM054561Dendrobium aphyllum
HM054557Dendrobium aphyllum
HM054555Dendrobium aphyllum
HM054553Dendrobium aphyllum
AY485713Dendrobium chrysanthum
EU003119Dendrobium chrysanthum
KJ944627Dendrobium chrysanthum
HM590376Dendrobium chrysanthum
HQ114238Dendrobium chrysanthum
JN388584Dendrobium chrysanthum
AF362047Dendrobium chrysanthum
KJ210429Dendrobium chrysanthum
JF713091Dendrobium chrysanthum
KF143443Dendrobium chrysanthum
KJ210427Dendrobium chrysanthum
KJ672657Dendrobium chrysanthum
JF713093Dendrobium chrysanthum
JF713094Dendrobium chrysanthum
KP159297Dendrobium hancockii
AF362025Dendrobium hancockii
HM590377Dendrobium hancockii
JN388591Dendrobium hancockii
DQ058787Dendrobium hancockii
HQ114259Dendrobium hancockii
AB593575Dendrobium hancockii
KF143467Dendrobium hancockii
EU003120Dendrobium hancockii
AF362038Dendrobium hancockii
AB593584Dendrobium hookerianum
KF143474Dendrobium hookerianum
KJ672678Dendrobium hookerianum
KJ210458Dendrobium hookerianum
KX509992Dendrobium hookerianum
KF143475Dendrobium hookerianum
AF363024Dendrobium lohohense
AB593605Dendrobium lohohense
KF143482Dendrobium lohohense
JN388574Dendrobium lohohense
KJ672684Dendrobium lohohense

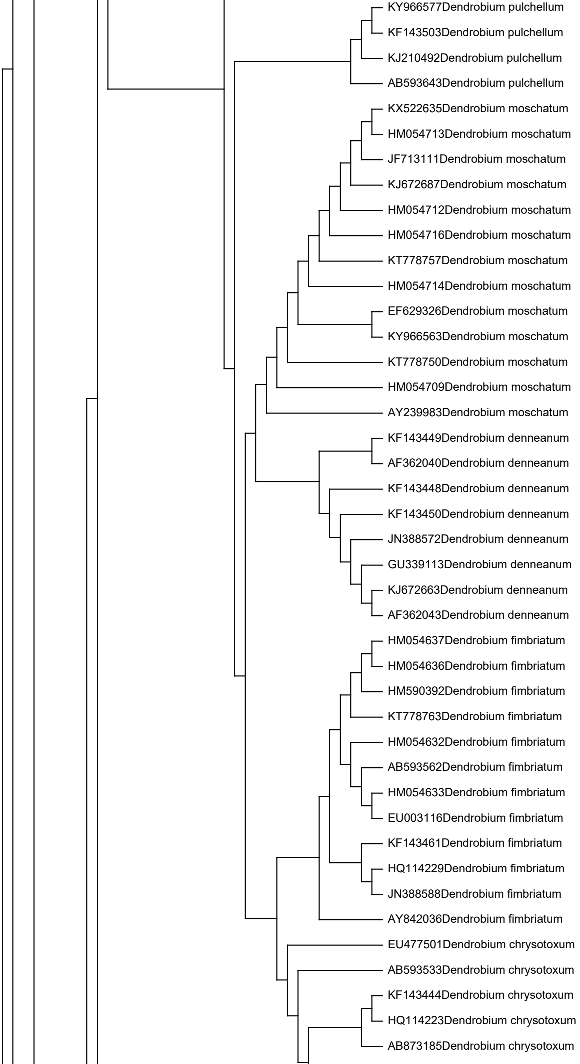
KY966577Dendrobium pulchellum
KF143503Dendrobium pulchellum
KJ210492Dendrobium pulchellum
AB593643Dendrobium pulchellum
KX522635Dendrobium moschatum
HM054713Dendrobium moschatum
JF713111Dendrobium moschatum
KJ672687Dendrobium moschatum
HM054712Dendrobium moschatum
HM054716Dendrobium moschatum
KT778757Dendrobium moschatum
HM054714Dendrobium moschatum
EF629326Dendrobium moschatum
KY966563Dendrobium moschatum
KT778750Dendrobium moschatum
HM054709Dendrobium moschatum
AY239983Dendrobium moschatum
KF143449Dendrobium denneanum
AF362040Dendrobium denneanum
KF143448Dendrobium denneanum
KF143450Dendrobium denneanum
JN388572Dendrobium denneanum
GU339113Dendrobium denneanum
KJ672663Dendrobium denneanum
AF362043Dendrobium denneanum
HM054637Dendrobium fimbriatum
HM054636Dendrobium fimbriatum
HM590392Dendrobium fimbriatum
KT778763Dendrobium fimbriatum
HM054632Dendrobium fimbriatum
AB593562Dendrobium fimbriatum
HM054633Dendrobium fimbriatum
EU003116Dendrobium fimbriatum
KF143461Dendrobium fimbriatum
HQ114229Dendrobium fimbriatum
JN388588Dendrobium fimbriatum
AY842036Dendrobium fimbriatum
EU477501Dendrobium chrysotoxum
AB593533Dendrobium chrysotoxum
KF143444Dendrobium chrysotoxum
HQ114223Dendrobium chrysotoxum
AB873185Dendrobium chrysotoxum

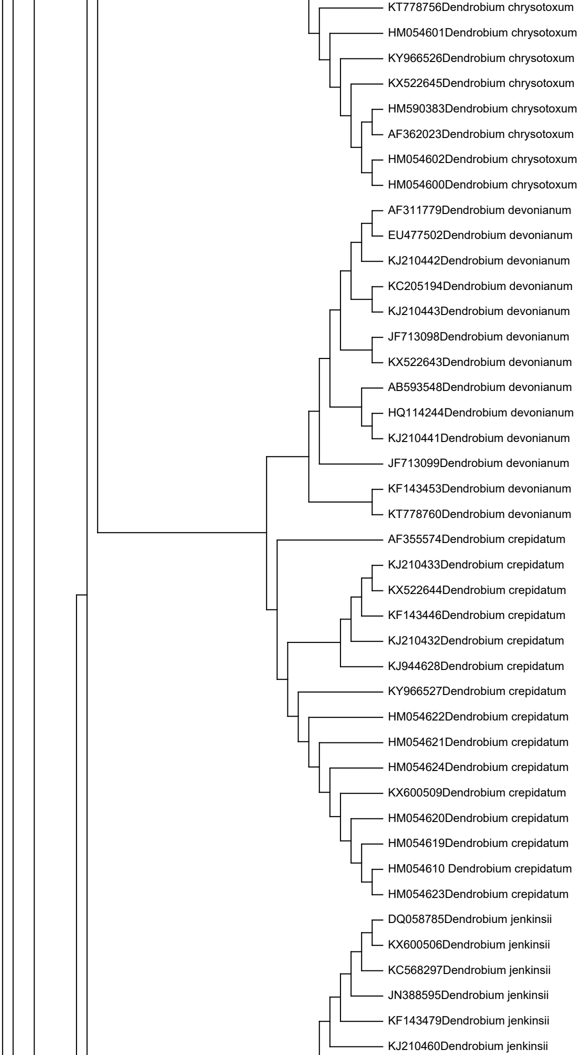
KT778756Dendrobium chrysotoxum
HM054601Dendrobium chrysotoxum
KY966526Dendrobium chrysotoxum
KX522645Dendrobium chrysotoxum
HM590383Dendrobium chrysotoxum
AF362023Dendrobium chrysotoxum
HM054602Dendrobium chrysotoxum
HM054600Dendrobium chrysotoxum
AF311779Dendrobium devonianum
EU477502Dendrobium devonianum
KJ210442Dendrobium devonianum
KC205194Dendrobium devonianum
KJ210443Dendrobium devonianum
JF713098Dendrobium devonianum
KX522643Dendrobium devonianum
AB593548Dendrobium devonianum
HQ114244Dendrobium devonianum
KJ210441Dendrobium devonianum
JF713099Dendrobium devonianum
KF143453Dendrobium devonianum
KT778760Dendrobium devonianum
AF355574Dendrobium crepidatum
KJ210433Dendrobium crepidatum
KX522644Dendrobium crepidatum
KF143446Dendrobium crepidatum
KJ210432Dendrobium crepidatum
KJ944628Dendrobium crepidatum
KY966527Dendrobium crepidatum
HM054622Dendrobium crepidatum
HM054621Dendrobium crepidatum
HM054624Dendrobium crepidatum
KX600509Dendrobium crepidatum
HM054620Dendrobium crepidatum
HM054619Dendrobium crepidatum
HM054610 Dendrobium crepidatum
HM054623Dendrobium crepidatum
DQ058785Dendrobium jenkinsii
KX600506Dendrobium jenkinsii
KC568297Dendrobium jenkinsii
JN388595Dendrobium jenkinsii
KF143479Dendrobium jenkinsii
KJ210460Dendrobium jenkinsii

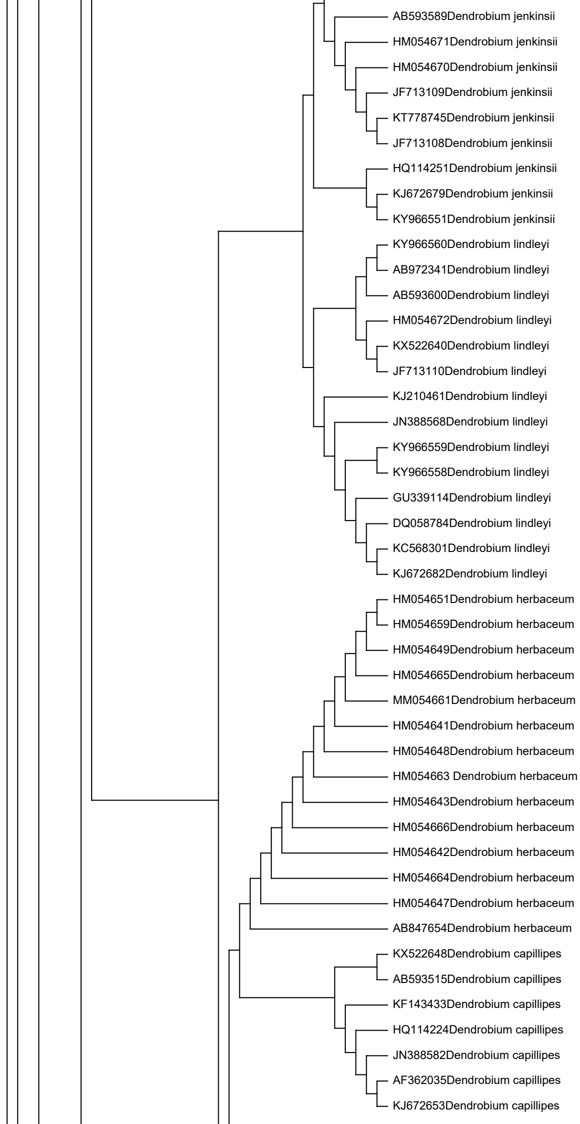
AB593589Dendrobium jenkinsii
HM054671Dendrobium jenkinsii
HM054670Dendrobium jenkinsii
JF713109Dendrobium jenkinsii
KT778745Dendrobium jenkinsii
JF713108Dendrobium jenkinsii
HQ114251Dendrobium jenkinsii
KJ672679Dendrobium jenkinsii
KY966551Dendrobium jenkinsii
KY966560Dendrobium lindleyi
AB972341Dendrobium lindleyi
AB593600Dendrobium lindleyi
HM054672Dendrobium lindleyi
KX522640Dendrobium lindleyi
JF713110Dendrobium lindleyi
KJ210461Dendrobium lindleyi
JN388568Dendrobium lindleyi
KY966559Dendrobium lindleyi
KY966558Dendrobium lindleyi
GU339114Dendrobium lindleyi
DQ058784Dendrobium lindleyi
KC568301Dendrobium lindleyi
KJ672682Dendrobium lindleyi
HM054651Dendrobium herbaceum
HM054659Dendrobium herbaceum
HM054649Dendrobium herbaceum
HM054665Dendrobium herbaceum
MM054661Dendrobium herbaceum
HM054641Dendrobium herbaceum
HM054648Dendrobium herbaceum
HM054663 Dendrobium herbaceum
HM054643Dendrobium herbaceum
HM054666Dendrobium herbaceum
HM054642Dendrobium herbaceum
HM054664Dendrobium herbaceum
HM054647Dendrobium herbaceum
AB847654Dendrobium herbaceum
KX522648Dendrobium capillipes
AB593515Dendrobium capillipes
KF143433Dendrobium capillipes
HQ114224Dendrobium capillipes
JN388582Dendrobium capillipes
AF362035Dendrobium capillipes
KJ672653Dendrobium capillipes

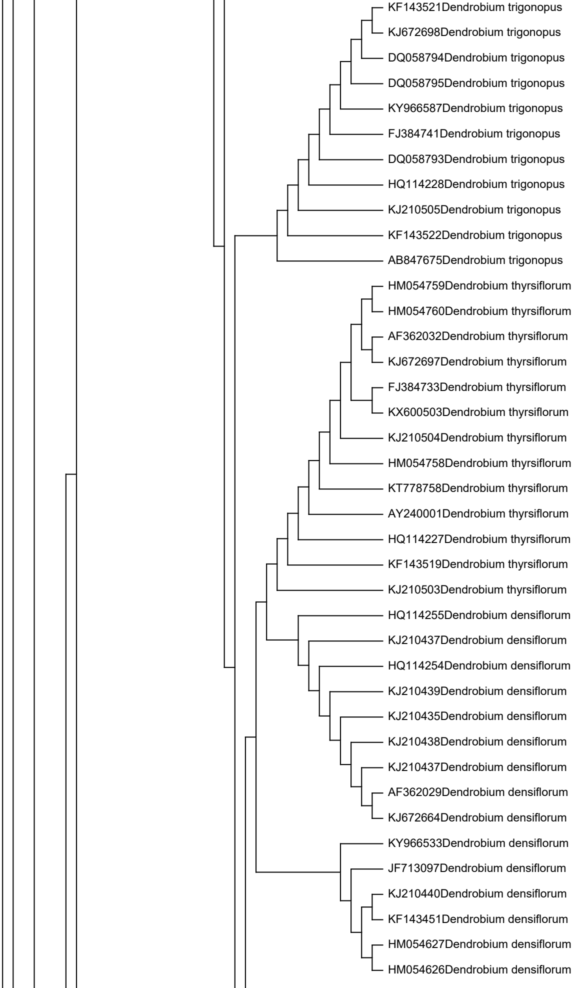

KF143521Dendrobium trigonopus
KJ672698Dendrobium trigonopus
DQ058794Dendrobium trigonopus
DQ058795Dendrobium trigonopus
KY966587Dendrobium trigonopus
FJ384741Dendrobium trigonopus
DQ058793Dendrobium trigonopus
HQ114228Dendrobium trigonopus
KJ210505Dendrobium trigonopus
KF143522Dendrobium trigonopus
AB847675Dendrobium trigonopus
HM054759Dendrobium thyrsiflorum
HM054760Dendrobium thyrsiflorum
AF362032Dendrobium thyrsiflorum
KJ672697Dendrobium thyrsiflorum
FJ384733Dendrobium thyrsiflorum
KX600503Dendrobium thyrsiflorum
KJ210504Dendrobium thyrsiflorum
HM054758Dendrobium thyrsiflorum
KT778758Dendrobium thyrsiflorum
AY240001Dendrobium thyrsiflorum
HQ114227Dendrobium thyrsiflorum
KF143519Dendrobium thyrsiflorum
KJ210503Dendrobium thyrsiflorum
HQ114255Dendrobium densiflorum
KJ210437Dendrobium densiflorum
HQ114254Dendrobium densiflorum
KJ210439Dendrobium densiflorum
KJ210435Dendrobium densiflorum
KJ210438Dendrobium densiflorum
KJ210437Dendrobium densiflorum
AF362029Dendrobium densiflorum
KJ672664Dendrobium densiflorum
KY966533Dendrobium densiflorum
JF713097Dendrobium densiflorum
KJ210440Dendrobium densiflorum
KF143451Dendrobium densiflorum
HM054627Dendrobium densiflorum
HM054626Dendrobium densiflorum

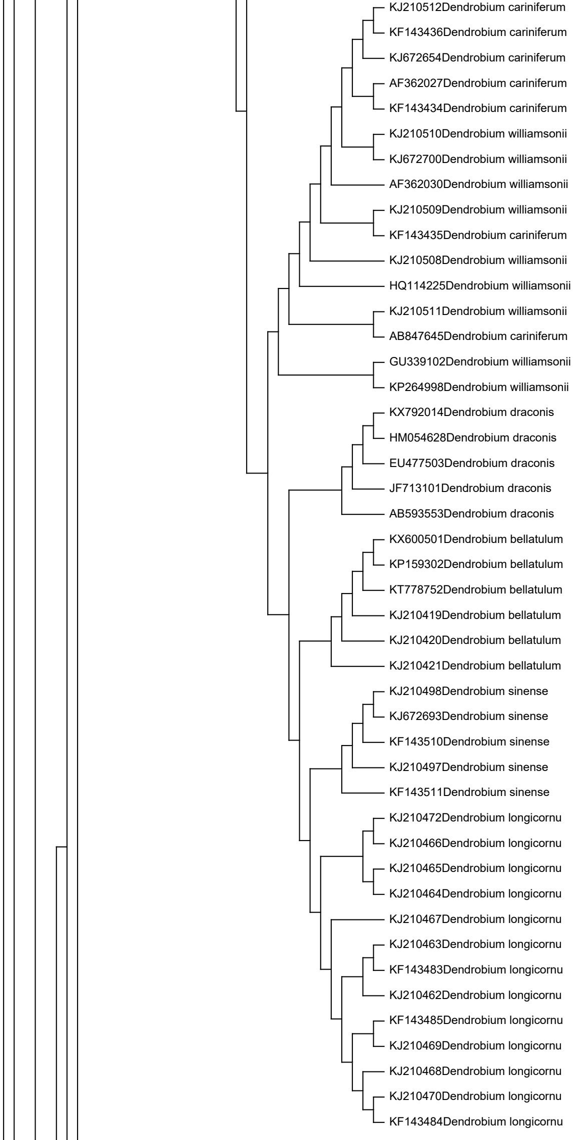
KJ210512Dendrobium cariniferum
KF143436Dendrobium cariniferum
KJ672654Dendrobium cariniferum
AF362027Dendrobium cariniferum
KF143434Dendrobium cariniferum
KJ210510Dendrobium williamsonii
KJ672700Dendrobium williamsonii
AF362030Dendrobium williamsonii
KJ210509Dendrobium williamsonii
KF143435Dendrobium cariniferum
KJ210508Dendrobium williamsonii
HQ114225Dendrobium williamsonii
KJ210511Dendrobium williamsonii
AB847645Dendrobium cariniferum
GU339102Dendrobium williamsonii
KP264998Dendrobium williamsonii
KX792014Dendrobium draconis
HM054628Dendrobium draconis
EU477503Dendrobium draconis
JF713101Dendrobium draconis
AB593553Dendrobium draconis
KX600501Dendrobium bellatulum
KP159302Dendrobium bellatulum
KT778752Dendrobium bellatulum
KJ210419Dendrobium bellatulum
KJ210420Dendrobium bellatulum
KJ210421Dendrobium bellatulum
KJ210498Dendrobium sinense
KJ672693Dendrobium sinense
KF143510Dendrobium sinense
KJ210497Dendrobium sinense
KF143511Dendrobium sinense
KJ210472Dendrobium longicornu
KJ210466Dendrobium longicornu
KJ210465Dendrobium longicornu
KJ210464Dendrobium longicornu
KJ210467Dendrobium longicornu
KJ210463Dendrobium longicornu
KF143483Dendrobium longicornu
KJ210462Dendrobium longicornu
KF143485Dendrobium longicornu
KJ210469Dendrobium longicornu
KJ210468Dendrobium longicornu
KJ210470Dendrobium longicornu
KF143484Dendrobium longicornu

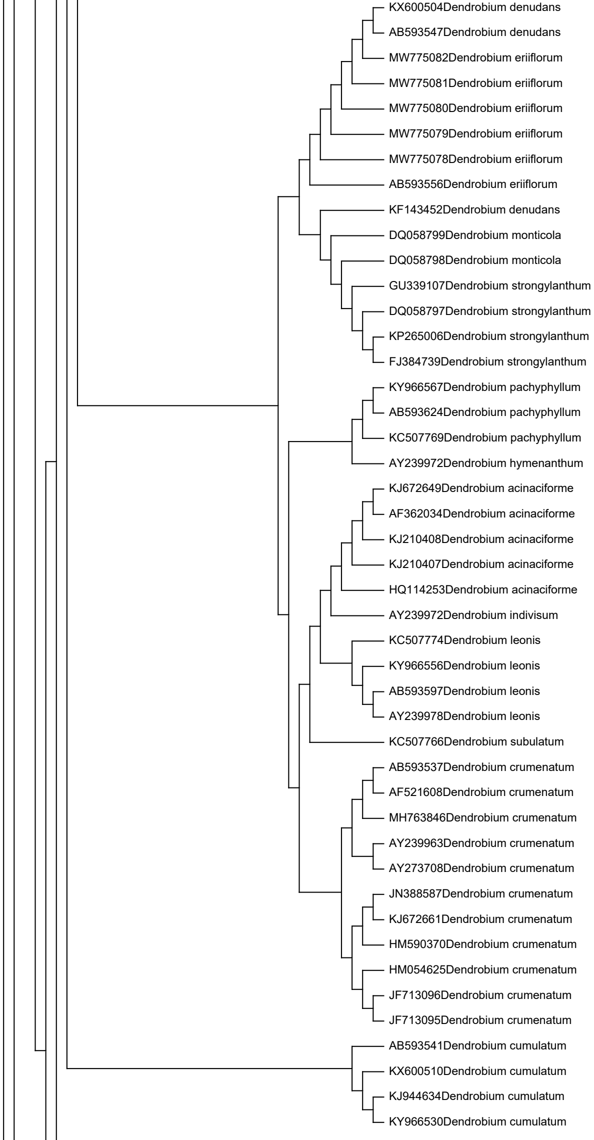

KX600504Dendrobium denudans
AB593547Dendrobium denudans
MW775082Dendrobium eriiflorum
MW775081Dendrobium eriiflorum
MW775080Dendrobium eriiflorum
MW775079Dendrobium eriiflorum
MW775078Dendrobium eriiflorum
AB593556Dendrobium eriiflorum
KF143452Dendrobium denudans
DQ058799Dendrobium monticola
DQ058798Dendrobium monticola
GU339107Dendrobium strongylanthum
DQ058797Dendrobium strongylanthum
KP265006Dendrobium strongylanthum
FJ384739Dendrobium strongylanthum
KY966567Dendrobium pachyphyllum
AB593624Dendrobium pachyphyllum
KC507769Dendrobium pachyphyllum
AY239972Dendrobium hymenanthum
KJ672649Dendrobium acinaciforme
AF362034Dendrobium acinaciforme
KJ210408Dendrobium acinaciforme
KJ210407Dendrobium acinaciforme
HQ114253Dendrobium acinaciforme
AY239972Dendrobium indivisum
KC507774Dendrobium leonis
KY966556Dendrobium leonis
AB593597Dendrobium leonis
AY239978Dendrobium leonis
KC507766Dendrobium subulatum
AB593537Dendrobium crumenatum
AF521608Dendrobium crumenatum
MH763846Dendrobium crumenatum
AY239963Dendrobium crumenatum
AY273708Dendrobium crumenatum
JN388587Dendrobium crumenatum
KJ672661Dendrobium crumenatum
HM590370Dendrobium crumenatum
HM054625Dendrobium crumenatum
JF713096Dendrobium crumenatum
JF713095Dendrobium crumenatum
AB593541Dendrobium cumulatum
KX600510Dendrobium cumulatum
KJ944634Dendrobium cumulatum
KY966530Dendrobium cumulatum

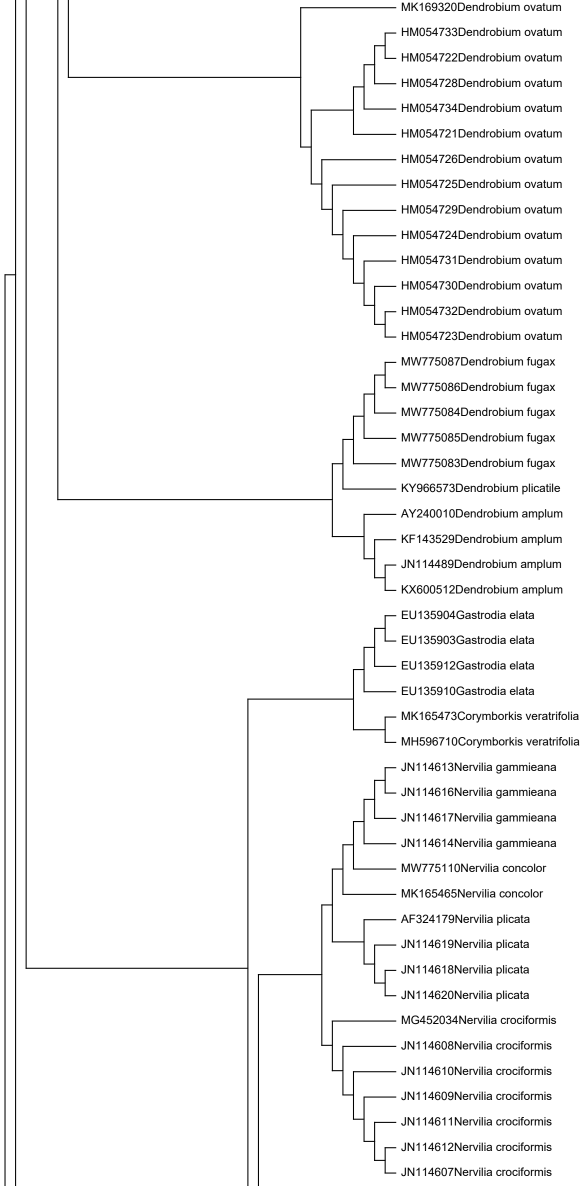
MK169320Dendrobium ovatum
HM054733Dendrobium ovatum
HM054722Dendrobium ovatum
HM054728Dendrobium ovatum
HM054734Dendrobium ovatum
HM054721Dendrobium ovatum
HM054726Dendrobium ovatum
HM054725Dendrobium ovatum
HM054729Dendrobium ovatum
HM054724Dendrobium ovatum
HM054731Dendrobium ovatum
HM054730Dendrobium ovatum
HM054732Dendrobium ovatum
HM054723Dendrobium ovatum
MW775087Dendrobium fugax
MW775086Dendrobium fugax
MW775084Dendrobium fugax
MW775085Dendrobium fugax
MW775083Dendrobium fugax
KY966573Dendrobium plicatile
AY240010Dendrobium amplum
KF143529Dendrobium amplum
JN114489Dendrobium amplum
KX600512Dendrobium amplum
EU135904Gastrodia elata
EU135903Gastrodia elata
EU135912Gastrodia elata
EU135910Gastrodia elata
MK165473Corymborkis veratrifolia
MH596710Corymborkis veratrifolia
JN114613Nervilia gammieana
JN114616Nervilia gammieana
JN114617Nervilia gammieana
JN114614Nervilia gammieana
MW775110Nervilia concolor
MK165465Nervilia concolor
AF324179Nervilia plicata
JN114619Nervilia plicata
JN114618Nervilia plicata
JN114620Nervilia plicata
MG452034Nervilia crociformis
JN114608Nervilia crociformis
JN114610Nervilia crociformis
JN114609Nervilia crociformis
JN114611Nervilia crociformis
JN114612Nervilia crociformis
JN114607Nervilia crociformis

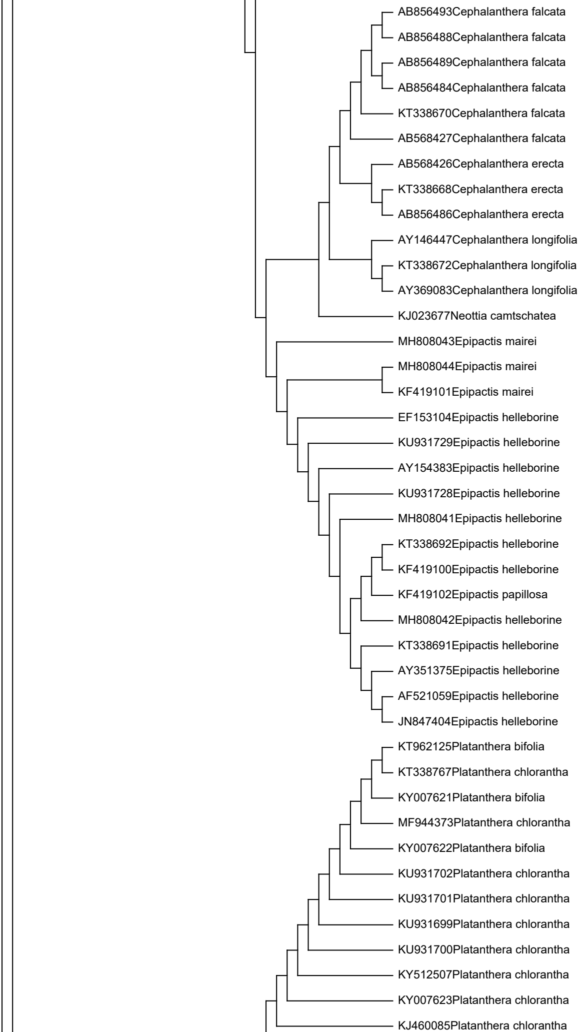
AB856493Cephalanthera falcata
AB856488Cephalanthera falcata
AB856489Cephalanthera falcata
AB856484Cephalanthera falcata
KT338670Cephalanthera falcata
AB568427Cephalanthera falcata
AB568426Cephalanthera erecta
KT338668Cephalanthera erecta
AB856486Cephalanthera erecta
AY146447Cephalanthera longifolia
KT338672Cephalanthera longifolia
AY369083Cephalanthera longifolia
KJ023677Neottia camtschatea
MH808043Epipactis mairei
MH808044Epipactis mairei
KF419101Epipactis mairei
EF153104Epipactis helleborine
KU931729Epipactis helleborine
AY154383Epipactis helleborine
KU931728Epipactis helleborine
MH808041Epipactis helleborine
KT338692Epipactis helleborine
KF419100Epipactis helleborine
KF419102Epipactis papillosa
MH808042Epipactis helleborine
KT338691Epipactis helleborine
AY351375Epipactis helleborine
AF521059Epipactis helleborine
JN847404Epipactis helleborine
KT962125Platanthera bifolia
KT338767Platanthera chlorantha
KY007621Platanthera bifolia
MF944373Platanthera chlorantha
KY007622Platanthera bifolia
KU931702Platanthera chlorantha
KU931701Platanthera chlorantha
KU931699Platanthera chlorantha
KU931700Platanthera chlorantha
KY512507Platanthera chlorantha
KY007623Platanthera chlorantha
KJ460085Platanthera chlorantha

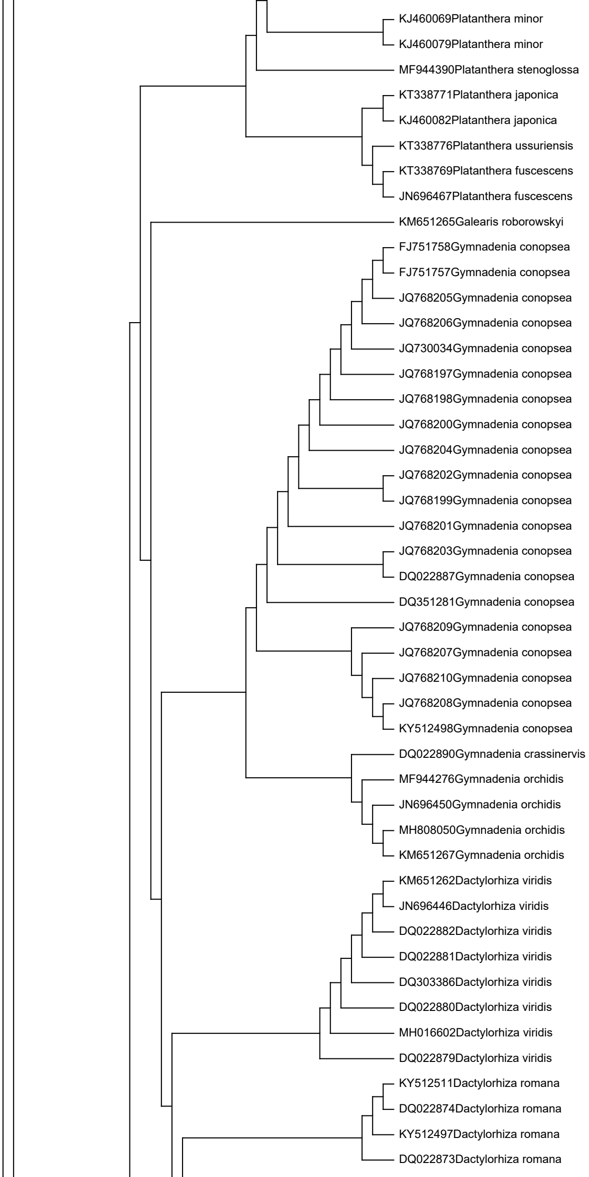
KJ460069Platanthera minor
KJ460079Platanthera minor
MF944390Platanthera stenoglossa
KT338771Platanthera japonica
KJ460082Platanthera japonica
KT338776Platanthera ussuriensis
KT338769Platanthera fuscescens
JN696467Platanthera fuscescens
KM651265Galearis roborowskyi
FJ751758Gymnadenia conopsea
FJ751757Gymnadenia conopsea
JQ768205Gymnadenia conopsea
JQ768206Gymnadenia conopsea
JQ730034Gymnadenia conopsea
JQ768197Gymnadenia conopsea
JQ768198Gymnadenia conopsea
JQ768200Gymnadenia conopsea
JQ768204Gymnadenia conopsea
JQ768202Gymnadenia conopsea
JQ768199Gymnadenia conopsea
JQ768201Gymnadenia conopsea
JQ768203Gymnadenia conopsea
DQ022887Gymnadenia conopsea
DQ351281Gymnadenia conopsea
JQ768209Gymnadenia conopsea
JQ768207Gymnadenia conopsea
JQ768210Gymnadenia conopsea
JQ768208Gymnadenia conopsea
KY512498Gymnadenia conopsea
DQ022890Gymnadenia crassinervis
MF944276Gymnadenia orchidis
JN696450Gymnadenia orchidis
MH808050Gymnadenia orchidis
KM651267Gymnadenia orchidis
KM651262Dactylorhiza viridis
JN696446Dactylorhiza viridis
DQ022882Dactylorhiza viridis
DQ022881Dactylorhiza viridis
DQ303386Dactylorhiza viridis
DQ022880Dactylorhiza viridis
MH016602Dactylorhiza viridis
DQ022879Dactylorhiza viridis
KY512511Dactylorhiza romana
DQ022874Dactylorhiza romana
KY512497Dactylorhiza romana
DQ022873Dactylorhiza romana

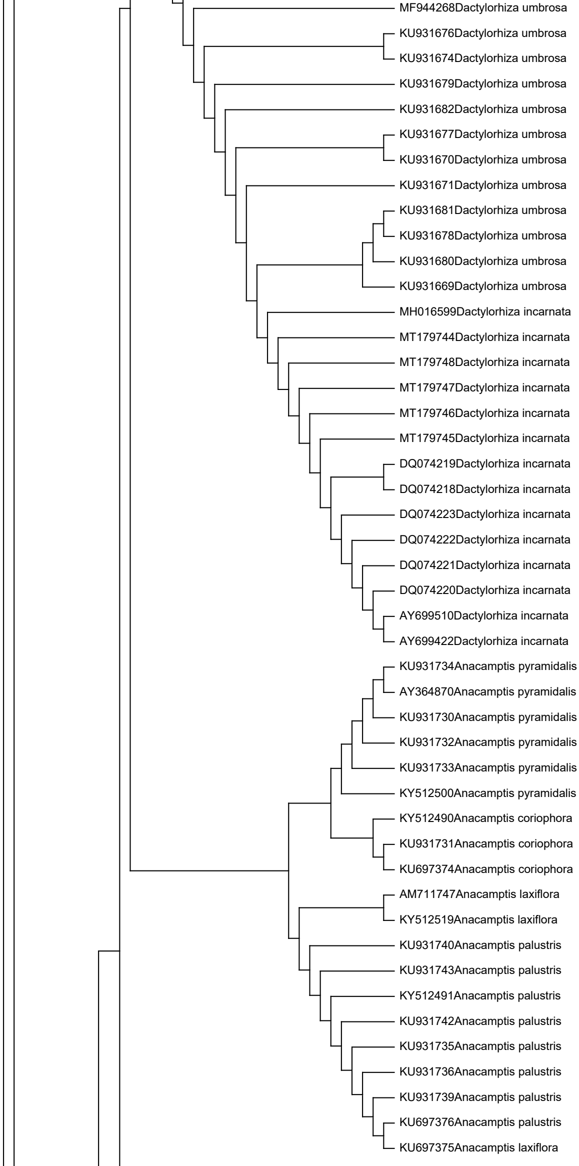
MF944268Dactylorhiza umbrosa
KU931676Dactylorhiza umbrosa
KU931674Dactylorhiza umbrosa
KU931679Dactylorhiza umbrosa
KU931682Dactylorhiza umbrosa
KU931677Dactylorhiza umbrosa
KU931670Dactylorhiza umbrosa
KU931671Dactylorhiza umbrosa
KU931681Dactylorhiza umbrosa
KU931678Dactylorhiza umbrosa
KU931680Dactylorhiza umbrosa
KU931669Dactylorhiza umbrosa
MH016599Dactylorhiza incarnata
MT179744Dactylorhiza incarnata
MT179748Dactylorhiza incarnata
MT179747Dactylorhiza incarnata
MT179746Dactylorhiza incarnata
MT179745Dactylorhiza incarnata
DQ074219Dactylorhiza incarnata
DQ074218Dactylorhiza incarnata
DQ074223Dactylorhiza incarnata
DQ074222Dactylorhiza incarnata
DQ074221Dactylorhiza incarnata
DQ074220Dactylorhiza incarnata
AY699510Dactylorhiza incarnata
AY699422Dactylorhiza incarnata
KU931734Anacamptis pyramidalis
AY364870Anacamptis pyramidalis
KU931730Anacamptis pyramidalis
KU931732Anacamptis pyramidalis
KU931733Anacamptis pyramidalis
KY512500Anacamptis pyramidalis
KY512490Anacamptis coriophora
KU931731Anacamptis coriophora
KU697374Anacamptis coriophora
AM711747Anacamptis laxiflora
KY512519Anacamptis laxiflora
KU931740Anacamptis palustris
KU931743Anacamptis palustris
KY512491Anacamptis palustris
KU931742Anacamptis palustris
KU931735Anacamptis palustris
KU931736Anacamptis palustris
KU931739Anacamptis palustris
KU697376Anacamptis palustris
KU697375Anacamptis laxiflora

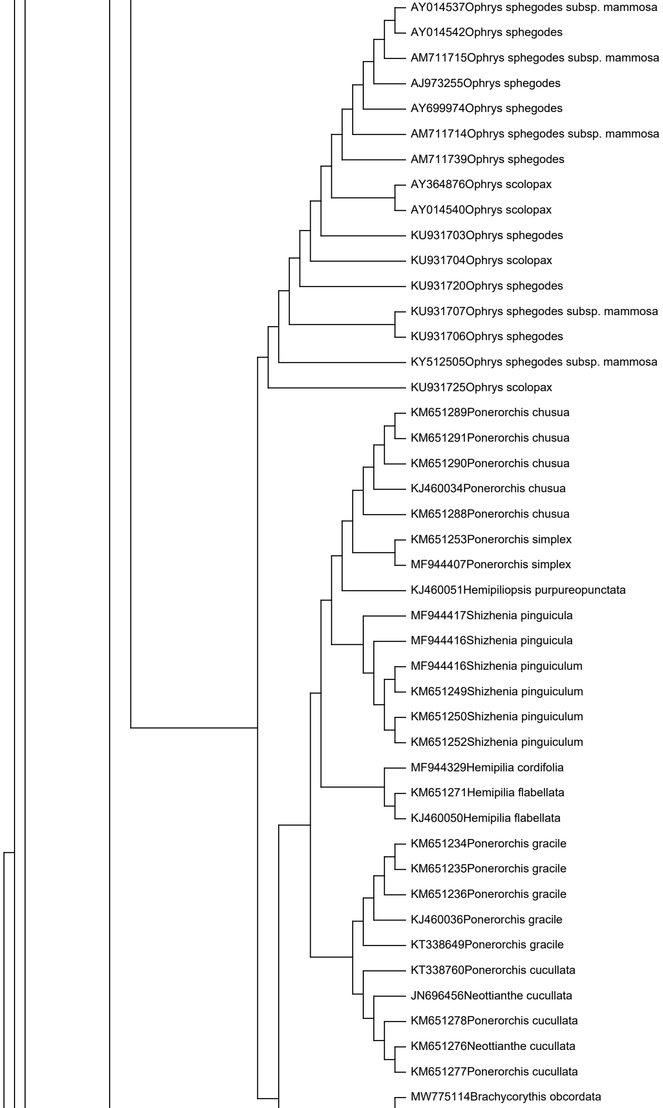
AY014537Ophrys sphegodes subsp. mammosa
AY014542Ophrys sphegodes
AM711715Ophrys sphegodes subsp. mammosa
AJ973255Ophrys sphegodes
AY699974Ophrys sphegodes
AM711714Ophrys sphegodes subsp. mammosa
AM711739Ophrys sphegodes
AY364876Ophrys scolopax
AY014540Ophrys scolopax
KU931703Ophrys sphegodes
KU931704Ophrys scolopax
KU931720Ophrys sphegodes
KU931707Ophrys sphegodes subsp. mammosa
KU931706Ophrys sphegodes
KY512505Ophrys sphegodes subsp. mammosa
KU931725Ophrys scolopax
KM651289Ponerorchis chusua
KM651291Ponerorchis chusua
KM651290Ponerorchis chusua
KJ460034Ponerorchis chusua
KM651288Ponerorchis chusua
KM651253Ponerorchis simplex
MF944407Ponerorchis simplex
KJ460051Hemipiliopsis purpureopunctata
MF944417Shizhenia pinguicula
MF944416Shizhenia pinguicula
MF944416Shizhenia pinguiculum
KM651249Shizhenia pinguiculum
KM651250Shizhenia pinguiculum
KM651252Shizhenia pinguiculum
MF944329Hemipilia cordifolia
KM651271Hemipilia flabellata
KJ460050Hemipilia flabellata
KM651234Ponerorchis gracile
KM651235Ponerorchis gracile
KM651236Ponerorchis gracile
KJ460036Ponerorchis gracile
KT338649Ponerorchis gracile
KT338760Ponerorchis cucullata
JN696456Neottianthe cucullata
KM651278Ponerorchis cucullata
KM651276Neottianthe cucullata
KM651277Ponerorchis cucullata
MW775114Brachycorythis obcordata

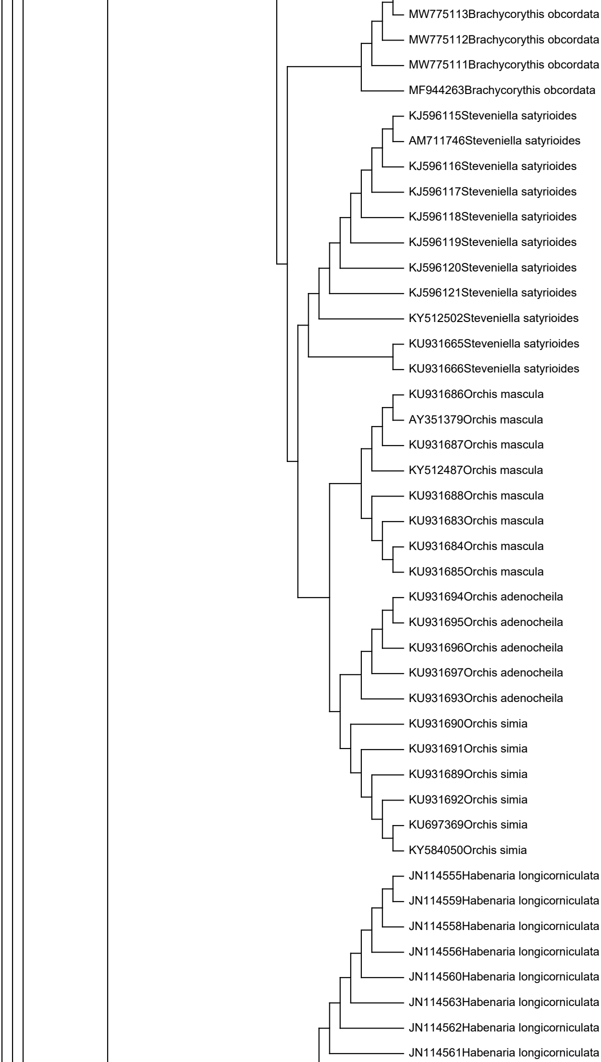
MW775113Brachycorythis obcordata
MW775112Brachycorythis obcordata
MW775111Brachycorythis obcordata
MF944263Brachycorythis obcordata
KJ596115Steveniella satyrioides
AM711746Steveniella satyrioides
KJ596116Steveniella satyrioides
KJ596117Steveniella satyrioides
KJ596118Steveniella satyrioides
KJ596119Steveniella satyrioides
KJ596120Steveniella satyrioides
KJ596121Steveniella satyrioides
KY512502Steveniella satyrioides
KU931665Steveniella satyrioides
KU931666Steveniella satyrioides
KU931686Orchis mascula
AY351379Orchis mascula
KU931687Orchis mascula
KY512487Orchis mascula
KU931688Orchis mascula
KU931683Orchis mascula
KU931684Orchis mascula
KU931685Orchis mascula
KU931694Orchis adenocheila
KU931695Orchis adenocheila
KU931696Orchis adenocheila
KU931697Orchis adenocheila
KU931693Orchis adenocheila
KU931690Orchis simia
KU931691Orchis simia
KU931689Orchis simia
KU931692Orchis simia
KU697369Orchis simia
KY584050Orchis simia
JN114555Habenaria longicorniculata
JN114559Habenaria longicorniculata
JN114558Habenaria longicorniculata
JN114556Habenaria longicorniculata
JN114560Habenaria longicorniculata
JN114563Habenaria longicorniculata
JN114562Habenaria longicorniculata
JN114561Habenaria longicorniculata

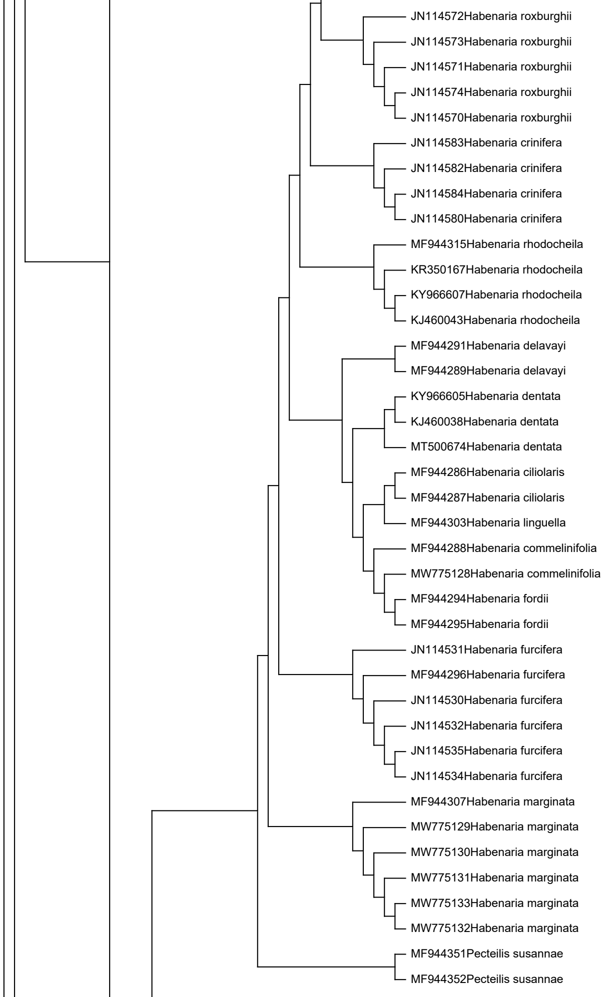
JN114572Habenaria roxburghii
JN114573Habenaria roxburghii
JN114571Habenaria roxburghii
JN114574Habenaria roxburghii
JN114570Habenaria roxburghii
JN114583Habenaria crinifera
JN114582Habenaria crinifera
JN114584Habenaria crinifera
JN114580Habenaria crinifera
MF944315Habenaria rhodocheila
KR350167Habenaria rhodocheila
KY966607Habenaria rhodocheila
KJ460043Habenaria rhodocheila
MF944291Habenaria delavayi
MF944289Habenaria delavayi
KY966605Habenaria dentata
KJ460038Habenaria dentata
MT500674Habenaria dentata
MF944286Habenaria ciliolaris
MF944287Habenaria ciliolaris
MF944303Habenaria linguella
MF944288Habenaria commelinifolia
MW775128Habenaria commelinifolia
MF944294Habenaria fordii
MF944295Habenaria fordii
JN114531Habenaria furcifera
MF944296Habenaria furcifera
JN114530Habenaria furcifera
JN114532Habenaria furcifera
JN114535Habenaria furcifera
JN114534Habenaria furcifera
MF944307Habenaria marginata
MW775129Habenaria marginata
MW775130Habenaria marginata
MW775131Habenaria marginata
MW775133Habenaria marginata
MW775132Habenaria marginata
MF944351Pecteilis susannae
MF944352Pecteilis susannae

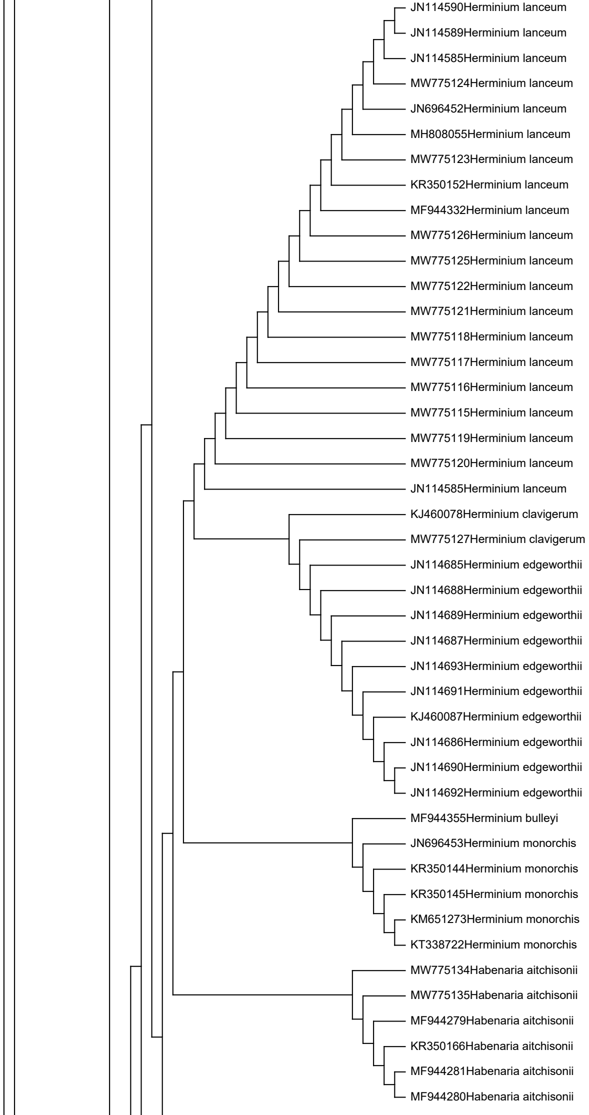
JN114590Herminium lanceum
JN114589Herminium lanceum
JN114585Herminium lanceum
MW775124Herminium lanceum
JN696452Herminium lanceum
MH808055Herminium lanceum
MW775123Herminium lanceum
KR350152Herminium lanceum
MF944332Herminium lanceum
MW775126Herminium lanceum
MW775125Herminium lanceum
MW775122Herminium lanceum
MW775121Herminium lanceum
MW775118Herminium lanceum
MW775117Herminium lanceum
MW775116Herminium lanceum
MW775115Herminium lanceum
MW775119Herminium lanceum
MW775120Herminium lanceum
JN114585Herminium lanceum
KJ460078Herminium clavigerum
MW775127Herminium clavigerum
JN114685Herminium edgeworthii
JN114688Herminium edgeworthii
JN114689Herminium edgeworthii
JN114687Herminium edgeworthii
JN114693Herminium edgeworthii
JN114691Herminium edgeworthii
KJ460087Herminium edgeworthii
JN114686Herminium edgeworthii
JN114690Herminium edgeworthii
JN114692Herminium edgeworthii
MF944355Herminium bulleyi
JN696453Herminium monorchis
KR350144Herminium monorchis
KR350145Herminium monorchis
KM651273Herminium monorchis
KT338722Herminium monorchis
MW775134Habenaria aitchisonii
MW775135Habenaria aitchisonii
MF944279Habenaria aitchisonii
KR350166Habenaria aitchisonii
MF944281Habenaria aitchisonii
MF944280Habenaria aitchisonii

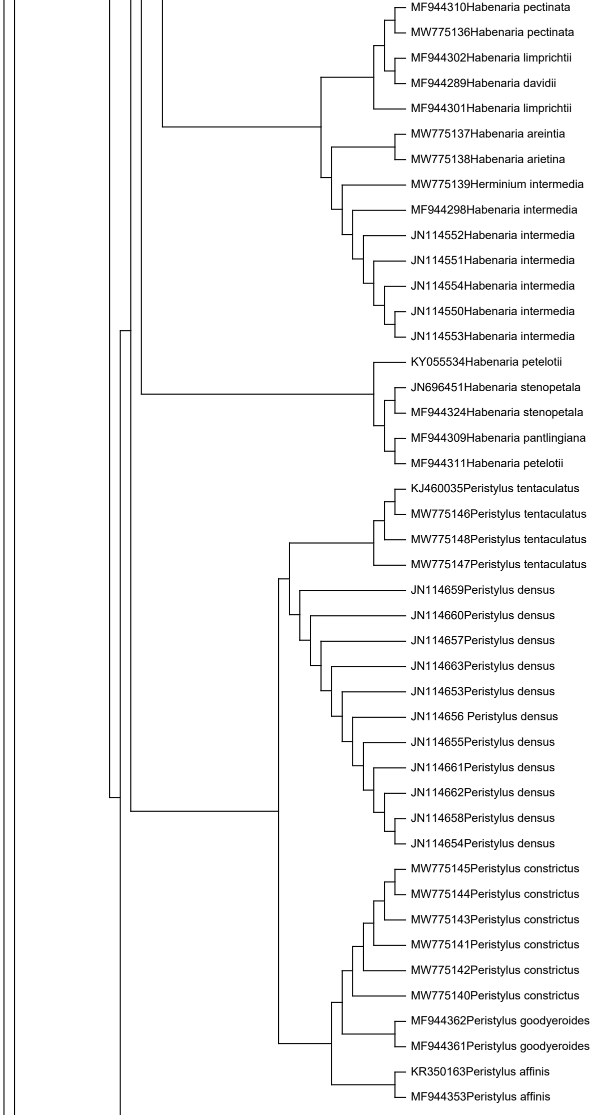
MF944310Habenaria pectinata
MW775136Habenaria pectinata
MF944302Habenaria limprichtii
MF944289Habenaria davidii
MF944301Habenaria limprichtii
MW775137Habenaria areintia
MW775138Habenaria arietina
MW775139Herminium intermedia
MF944298Habenaria intermedia
JN114552Habenaria intermedia
JN114551Habenaria intermedia
JN114554Habenaria intermedia
JN114550Habenaria intermedia
JN114553Habenaria intermedia
KY055534Habenaria petelotii
JN696451Habenaria stenopetala
MF944324Habenaria stenopetala
MF944309Habenaria pantlingiana
MF944311Habenaria petelotii
KJ460035Peristylus tentaculatus
MW775146Peristylus tentaculatus
MW775148Peristylus tentaculatus
MW775147Peristylus tentaculatus
JN114659Peristylus densus
JN114660Peristylus densus
JN114657Peristylus densus
JN114663Peristylus densus
JN114653Peristylus densus
JN114656 Peristylus densus
JN114655Peristylus densus
JN114661Peristylus densus
JN114662Peristylus densus
JN114658Peristylus densus
JN114654Peristylus densus
MW775145Peristylus constrictus
MW775144Peristylus constrictus
MW775143Peristylus constrictus
MW775141Peristylus constrictus
MW775142Peristylus constrictus
MW775140Peristylus constrictus
MF944362Peristylus goodyeroides
MF944361Peristylus goodyeroides
KR350163Peristylus affinis
MF944353Peristylus affinis

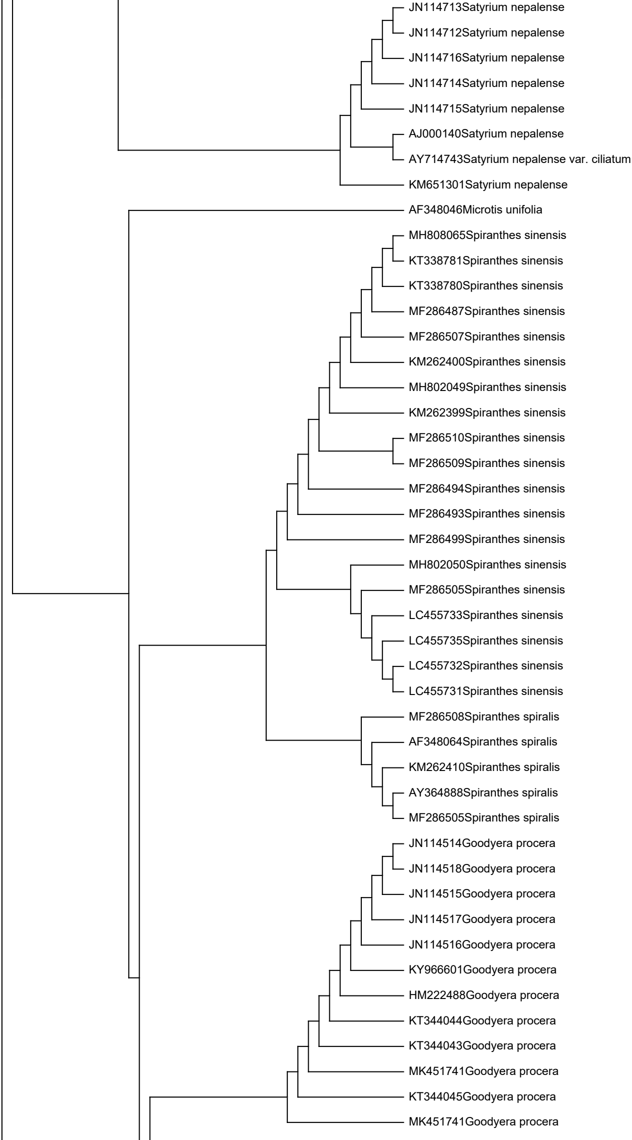
JN114713Satyrium nepalense
JN114712Satyrium nepalense
JN114716Satyrium nepalense
JN114714Satyrium nepalense
JN114715Satyrium nepalense
AJ000140Satyrium nepalense
AY714743Satyrium nepalense var. ciliatum
KM651301Satyrium nepalense
AF348046Microtis unifolia
MH808065Spiranthes sinensis
KT338781Spiranthes sinensis
KT338780Spiranthes sinensis
MF286487Spiranthes sinensis
MF286507Spiranthes sinensis
KM262400Spiranthes sinensis
MH802049Spiranthes sinensis
KM262399Spiranthes sinensis
MF286510Spiranthes sinensis
MF286509Spiranthes sinensis
MF286494Spiranthes sinensis
MF286493Spiranthes sinensis
MF286499Spiranthes sinensis
MH802050Spiranthes sinensis
MF286505Spiranthes sinensis
LC455733Spiranthes sinensis
LC455735Spiranthes sinensis
LC455732Spiranthes sinensis
LC455731Spiranthes sinensis
MF286508Spiranthes spiralis
AF348064Spiranthes spiralis
KM262410Spiranthes spiralis
AY364888Spiranthes spiralis
MF286505Spiranthes spiralis
JN114514Goodyera procera
JN114518Goodyera procera
JN114515Goodyera procera
JN114517Goodyera procera
JN114516Goodyera procera
KY966601Goodyera procera
HM222488Goodyera procera
KT344044Goodyera procera
KT344043Goodyera procera
MK451741Goodyera procera
KT344045Goodyera procera
MK451741Goodyera procera

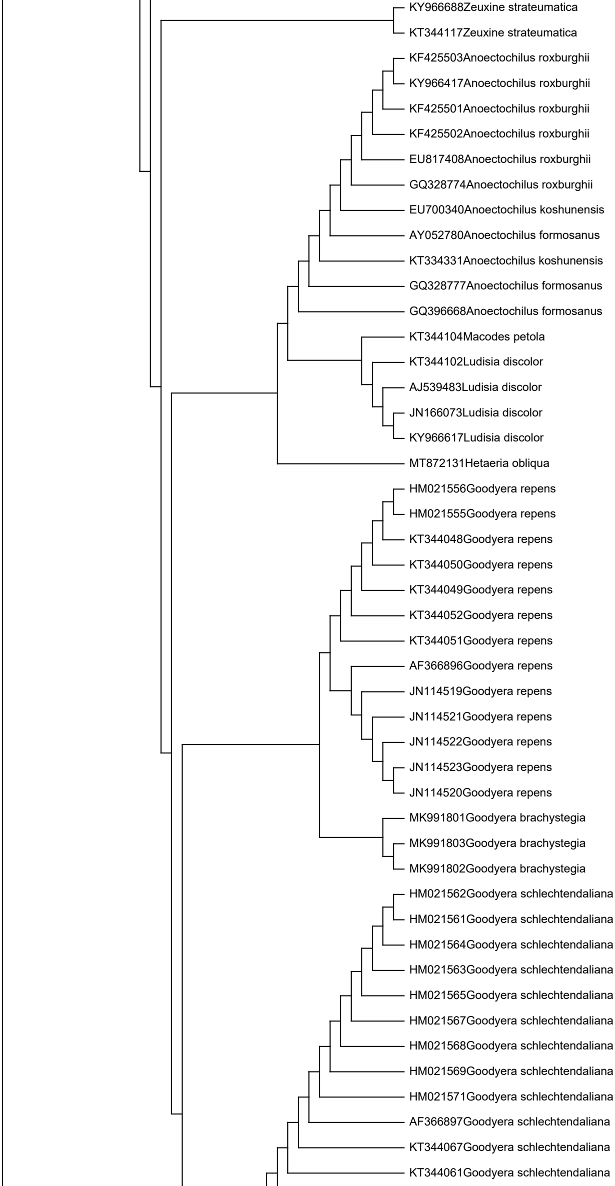
KY966688Zeuxine strateumatica
KT344117Zeuxine strateumatica
KF425503Anoectochilus roxburghii
KY966417Anoectochilus roxburghii
KF425501Anoectochilus roxburghii
KF425502Anoectochilus roxburghii
EU817408Anoectochilus roxburghii
GQ328774Anoectochilus roxburghii
EU700340Anoectochilus koshunensis
AY052780Anoectochilus formosanus
KT334331Anoectochilus koshunensis
GQ328777Anoectochilus formosanus
GQ396668Anoectochilus formosanus
KT344104Macodes petola
KT344102Ludisia discolor
AJ539483Ludisia discolor
JN166073Ludisia discolor
KY966617Ludisia discolor
MT872131Hetaeria obliqua
HM021556Goodyera repens
HM021555Goodyera repens
KT344048Goodyera repens
KT344050Goodyera repens
KT344049Goodyera repens
KT344052Goodyera repens
KT344051Goodyera repens
AF366896Goodyera repens
JN114519Goodyera repens
JN114521Goodyera repens
JN114522Goodyera repens
JN114523Goodyera repens
JN114520Goodyera repens
MK991801Goodyera brachystegia
MK991803Goodyera brachystegia
MK991802Goodyera brachystegia
HM021562Goodyera schlechtendaliana
HM021561Goodyera schlechtendaliana
HM021564Goodyera schlechtendaliana
HM021563Goodyera schlechtendaliana
HM021565Goodyera schlechtendaliana
HM021567Goodyera schlechtendaliana
HM021568Goodyera schlechtendaliana
HM021569Goodyera schlechtendaliana
HM021571Goodyera schlechtendaliana
AF366897Goodyera schlechtendaliana
KT344067Goodyera schlechtendaliana
KT344061Goodyera schlechtendaliana

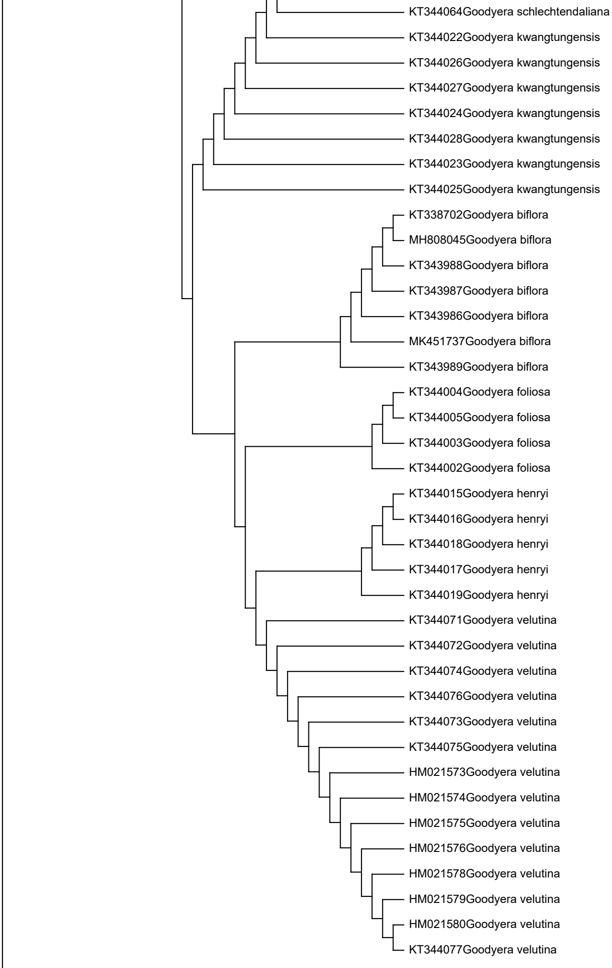
KT344064Goodyera schlechtendaliana
KT344022Goodyera kwangtungensis
KT344026Goodyera kwangtungensis
KT344027Goodyera kwangtungensis
KT344024Goodyera kwangtungensis
KT344028Goodyera kwangtungensis
KT344023Goodyera kwangtungensis
KT344025Goodyera kwangtungensis
KT338702Goodyera biflora
MH808045Goodyera biflora
KT343988Goodyera biflora
KT343987Goodyera biflora
KT343986Goodyera biflora
MK451737Goodyera biflora
KT343989Goodyera biflora
KT344004Goodyera foliosa
KT344005Goodyera foliosa
KT344003Goodyera foliosa
KT344002Goodyera foliosa
KT344015Goodyera henryi
KT344016Goodyera henryi
KT344018Goodyera henryi
KT344017Goodyera henryi
KT344019Goodyera henryi
KT344071Goodyera velutina
KT344072Goodyera velutina
KT344074Goodyera velutina
KT344076Goodyera velutina
KT344073Goodyera velutina
KT344075Goodyera velutina
HM021573Goodyera velutina
HM021574Goodyera velutina
HM021575Goodyera velutina
HM021576Goodyera velutina
HM021578Goodyera velutina
HM021579Goodyera velutina
HM021580Goodyera velutina
KT344077Goodyera velutina

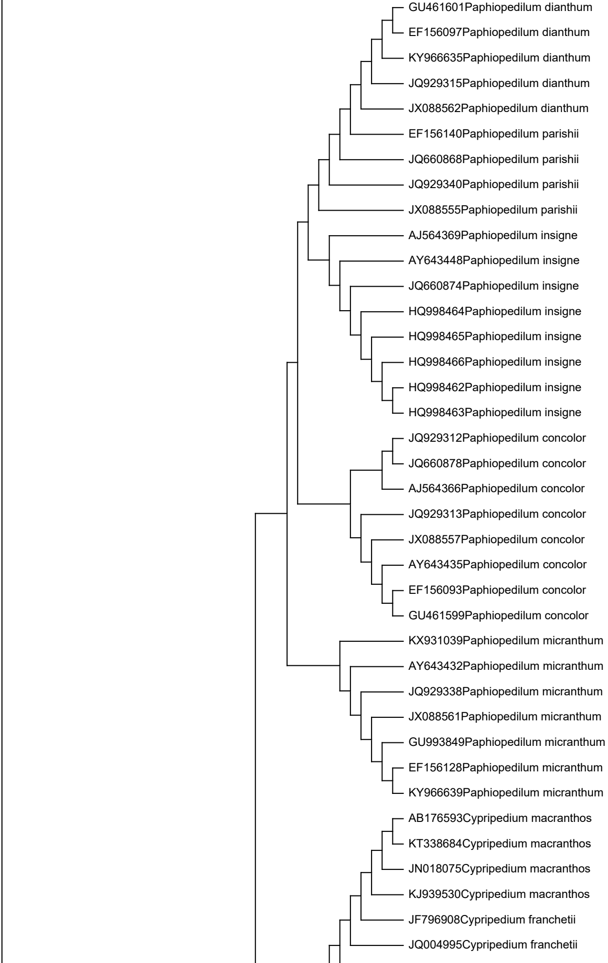
GU461601Paphiopedilum dianthum
EF156097Paphiopedilum dianthum
KY966635Paphiopedilum dianthum
JQ929315Paphiopedilum dianthum
JX088562Paphiopedilum dianthum
EF156140Paphiopedilum parishii
JQ660868Paphiopedilum parishii
JQ929340Paphiopedilum parishii
JX088555Paphiopedilum parishii
AJ564369Paphiopedilum insigne
AY643448Paphiopedilum insigne
JQ660874Paphiopedilum insigne
HQ998464Paphiopedilum insigne
HQ998465Paphiopedilum insigne
HQ998466Paphiopedilum insigne
HQ998462Paphiopedilum insigne
HQ998463Paphiopedilum insigne
JQ929312Paphiopedilum concolor
JQ660878Paphiopedilum concolor
AJ564366Paphiopedilum concolor
JQ929313Paphiopedilum concolor
JX088557Paphiopedilum concolor
AY643435Paphiopedilum concolor
EF156093Paphiopedilum concolor
GU461599Paphiopedilum concolor
KX931039Paphiopedilum micranthum
AY643432Paphiopedilum micranthum
JQ929338Paphiopedilum micranthum
JX088561Paphiopedilum micranthum
GU993849Paphiopedilum micranthum
EF156128Paphiopedilum micranthum
KY966639Paphiopedilum micranthum
AB176593Cypripedium macranthos
KT338684Cypripedium macranthos
JN018075Cypripedium macranthos
KJ939530Cypripedium macranthos
JF796908Cypripedium franchetii
JQ004995Cypripedium franchetii

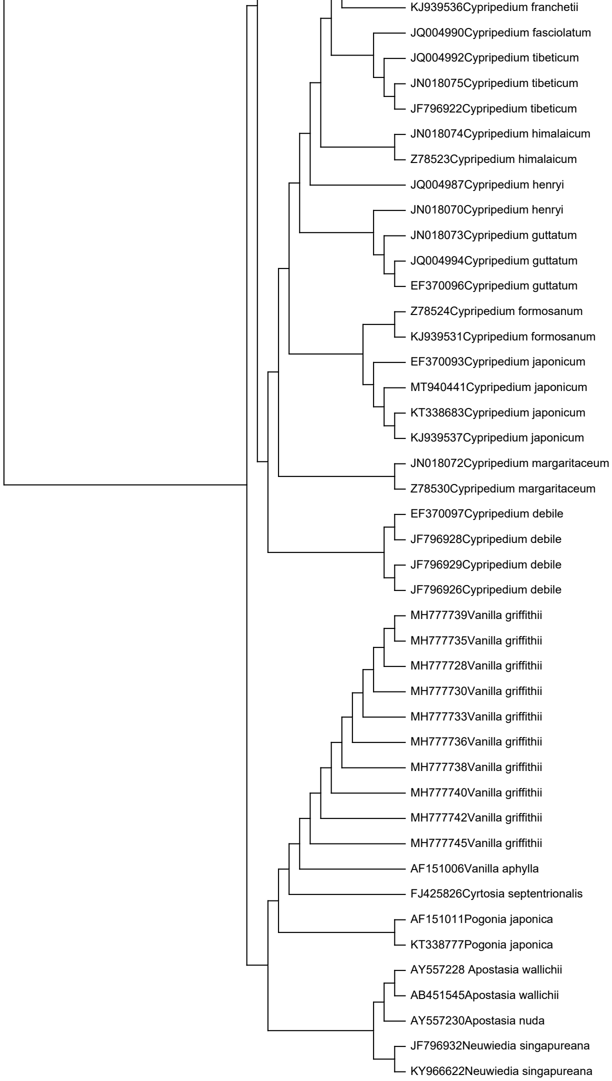

KJ939536Cypripedium franchetii
JQ004990Cypripedium fasciolatum
JQ004992Cypripedium tibeticum
JN018075Cypripedium tibeticum
JF796922Cypripedium tibeticum
JN018074Cypripedium himalaicum
Z78523Cypripedium himalaicum
JQ004987Cypripedium henryi
JN018070Cypripedium henryi
JN018073Cypripedium guttatum
JQ004994Cypripedium guttatum
EF370096Cypripedium guttatum
Z78524Cypripedium formosanum
KJ939531Cypripedium formosanum
EF370093Cypripedium japonicum
MT940441Cypripedium japonicum
KT338683Cypripedium japonicum
KJ939537Cypripedium japonicum
JN018072Cypripedium margaritaceum
Z78530Cypripedium margaritaceum
EF370097Cypripedium debile
JF796928Cypripedium debile
JF796929Cypripedium debile
JF796926Cypripedium debile
MH777739Vanilla griffithii
MH777735Vanilla griffithii
MH777728Vanilla griffithii
MH777730Vanilla griffithii
MH777733Vanilla griffithii
MH777736Vanilla griffithii
MH777738Vanilla griffithii
MH777740Vanilla griffithii
MH777742Vanilla griffithii
MH777745Vanilla griffithii
AF151006Vanilla aphylla
FJ425826Cyrtosia septentrionalis
AF151011Pogonia japonica
KT338777Pogonia japonica
AY557228 Apostasia wallichii
AB451545Apostasia wallichii
AY557230Apostasia nuda
JF796932Neuwiedia singapureana
KY966622Neuwiedia singapureana
