# Supplementary material for: DNA barcoding of medicinal orchids in Asia
Source: Sci Rep. 2021 Dec 8;11:23651. doi: 10.1038/s41598-021-03025-0 (PMC8654824; doi:10.1038/s41598-021-03025-0)
Supplement: Supplementary file 4 — Supplementary Figure S4. [file 41598_2021_3025_MOESM4_ESM.pdf]

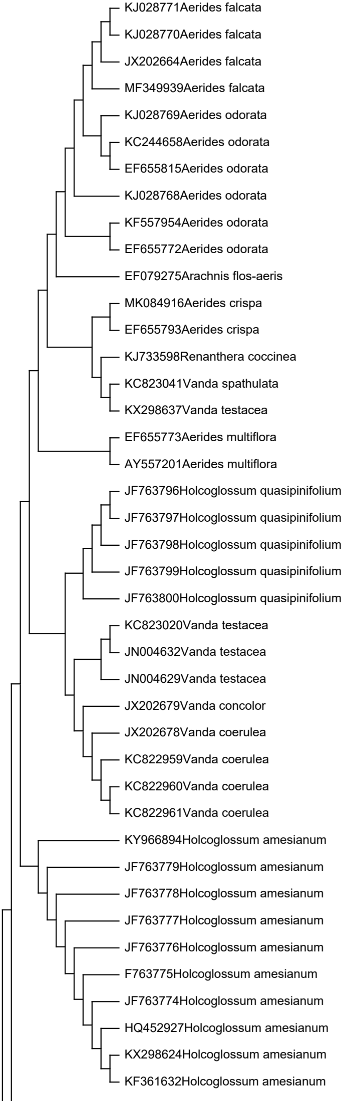
KJ028771Aerides falcata
KJ028770Aerides falcata
JX202664Aerides falcata
MF349939Aerides falcata
KJ028769Aerides odorata
KC244658Aerides odorata
EF655815Aerides odorata
KJ028768Aerides odorata
KF557954Aerides odorata
EF655772Aerides odorata
EF079275Arachnis flos-aeris
MK084916Aerides crispa
EF655793Aerides crispa
KJ733598Renanthera coccinea
KC823041Vanda spathulata
KX298637Vanda testacea
EF655773Aerides multiflora
AY557201Aerides multiflora
JF763796Holcoglossum quasipinifolium
JF763797Holcoglossum quasipinifolium
JF763798Holcoglossum quasipinifolium
JF763799Holcoglossum quasipinifolium
JF763800Holcoglossum quasipinifolium
KC823020Vanda testacea
JN004632Vanda testacea
JN004629Vanda testacea
JX202679Vanda concolor
JX202678Vanda coerulea
KC822959Vanda coerulea
KC822960Vanda coerulea
KC822961Vanda coerulea
KY966894Holcoglossum amesianum
JF763779Holcoglossum amesianum
JF763778Holcoglossum amesianum
JF763777Holcoglossum amesianum
JF763776Holcoglossum amesianum
F763775Holcoglossum amesianum
JF763774Holcoglossum amesianum
HQ452927Holcoglossum amesianum
KX298624Holcoglossum amesianum
KF361632Holcoglossum amesianum

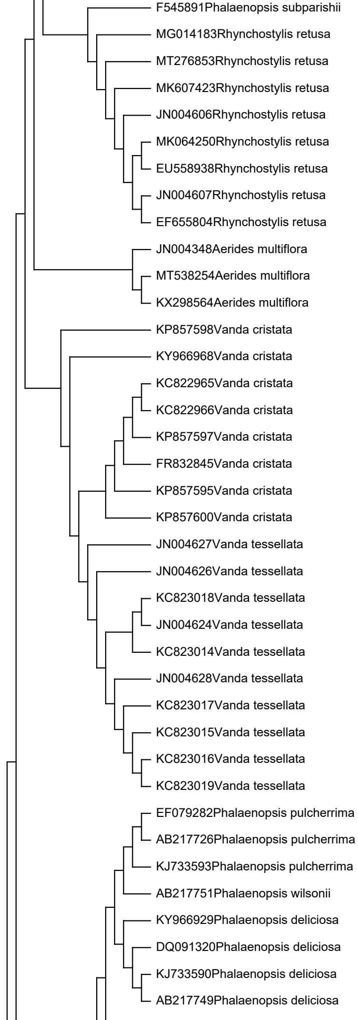
F545891Phalaenopsis subparishii
MG014183Rhynchostylis retusa
MT276853Rhynchostylis retusa
MK607423Rhynchostylis retusa
JN004606Rhynchostylis retusa
MK064250Rhynchostylis retusa
EU558938Rhynchostylis retusa
JN004607Rhynchostylis retusa
EF655804Rhynchostylis retusa
JN004348Aerides multiflora
MT538254Aerides multiflora
KX298564Aerides multiflora
KP857598Vanda cristata
KY966968Vanda cristata
KC822965Vanda cristata
KC822966Vanda cristata
KP857597Vanda cristata
FR832845Vanda cristata
KP857595Vanda cristata
KP857600Vanda cristata
JN004627Vanda tessellata
JN004626Vanda tessellata
KC823018Vanda tessellata
JN004624Vanda tessellata
KC823014Vanda tessellata
JN004628Vanda tessellata
KC823017Vanda tessellata
KC823015Vanda tessellata
KC823016Vanda tessellata
KC823019Vanda tessellata
EF079282Phalaenopsis pulcherrima
AB217726Phalaenopsis pulcherrima
KJ733593Phalaenopsis pulcherrima
AB217751Phalaenopsis wilsonii
KY966929Phalaenopsis deliciosa
DQ091320Phalaenopsis deliciosa
KJ733590Phalaenopsis deliciosa
AB217749Phalaenopsis deliciosa

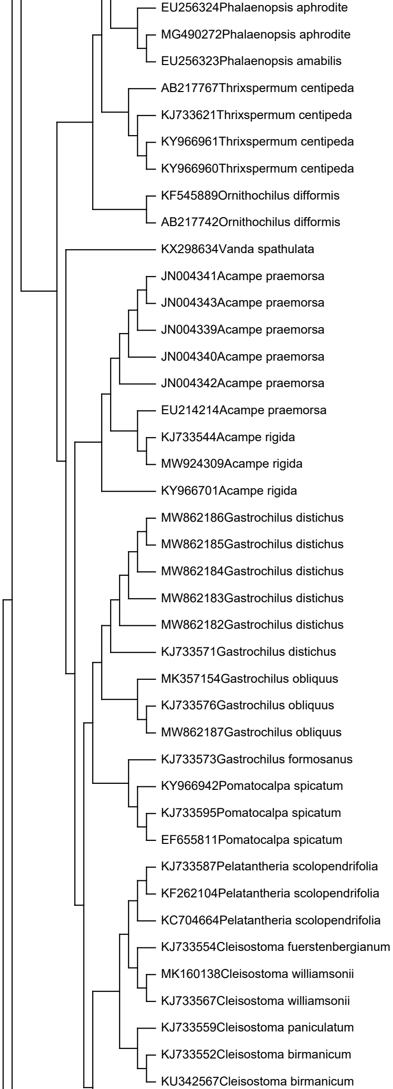
EU256324Phalaenopsis aphrodite
MG490272Phalaenopsis aphrodite
EU256323Phalaenopsis amabilis
AB217767Thrixspermum centipeda
KJ733621Thrixspermum centipeda
KY966961Thrixspermum centipeda
KY966960Thrixspermum centipeda
KF545889Ornithochilus difformis
AB217742Ornithochilus difformis
KX298634Vanda spathulata
JN004341Acampe praemorsa
JN004343Acampe praemorsa
JN004339Acampe praemorsa
JN004340Acampe praemorsa
JN004342Acampe praemorsa
EU214214Acampe praemorsa
KJ733544Acampe rigida
MW924309Acampe rigida
KY966701Acampe rigida
MW862186Gastrochilus distichus
MW862185Gastrochilus distichus
MW862184Gastrochilus distichus
MW862183Gastrochilus distichus
MW862182Gastrochilus distichus
KJ733571Gastrochilus distichus
MK357154Gastrochilus obliquus
KJ733576Gastrochilus obliquus
MW862187Gastrochilus obliquus
KJ733573Gastrochilus formosanus
KY966942Pomatocalpa spicatum
KJ733595Pomatocalpa spicatum
EF655811Pomatocalpa spicatum
KJ733587Pelatantheria scolopendrifolia
KF262104Pelatantheria scolopendrifolia
KC704664Pelatantheria scolopendrifolia
KJ733554Cleisostoma fuerstenbergianum
MK160138Cleisostoma williamsonii
KJ733567Cleisostoma williamsonii
KJ733559Cleisostoma paniculatum
KJ733552Cleisostoma birmanicum
KU342567Cleisostoma birmanicum

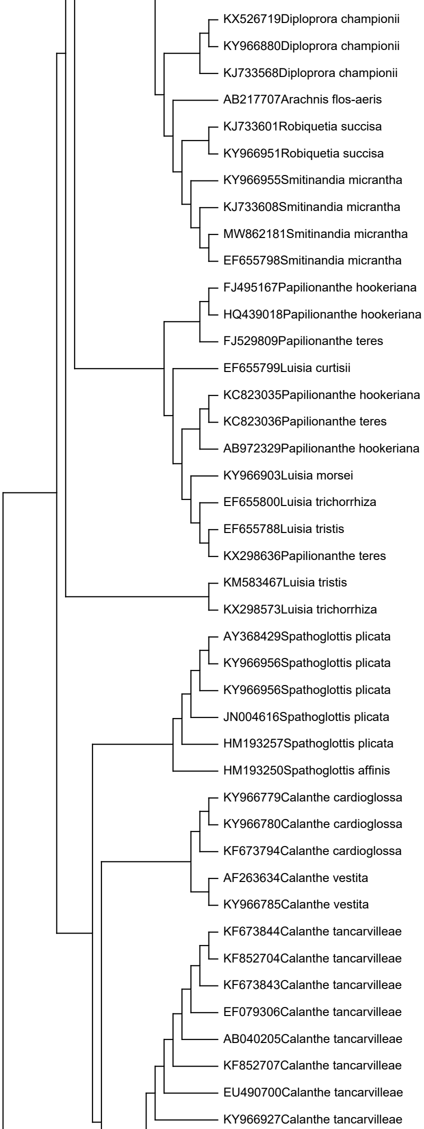

KX526719Diploprora championii
KY966880Diploprora championii
KJ733568Diploprora championii
AB217707Arachnis flos-aeris
KJ733601Robiquetia succisa
KY966951Robiquetia succisa
KY966955Smitinandia micrantha
KJ733608Smitinandia micrantha
MW862181Smitinandia micrantha
EF655798Smitinandia micrantha
FJ495167Papilionanthe hookeriana
HQ439018Papilionanthe hookeriana
FJ529809Papilionanthe teres
EF655799Luisia curtisii
KC823035Papilionanthe hookeriana
KC823036Papilionanthe teres
AB972329Papilionanthe hookeriana
KY966903Luisia morsei
EF655800Luisia trichorrhiza
EF655788Luisia tristis
KX298636Papilionanthe teres
KM583467Luisia tristis
KX298573Luisia trichorrhiza
AY368429Spathoglottis plicata
KY966956Spathoglottis plicata
KY966956Spathoglottis plicata
JN004616Spathoglottis plicata
HM193257Spathoglottis plicata
HM193250Spathoglottis affinis
KY966779Calanthe cardioglossa
KY966780Calanthe cardioglossa
KF673794Calanthe cardioglossa
AF263634Calanthe vestita
KY966785Calanthe vestita
KF673844Calanthe tancarvilleae
KF852704Calanthe tancarvilleae
KF673843Calanthe tancarvilleae
EF079306Calanthe tancarvilleae
AB040205Calanthe tancarvilleae
KF852707Calanthe tancarvilleae
EU490700Calanthe tancarvilleae
KY966927Calanthe tancarvilleae

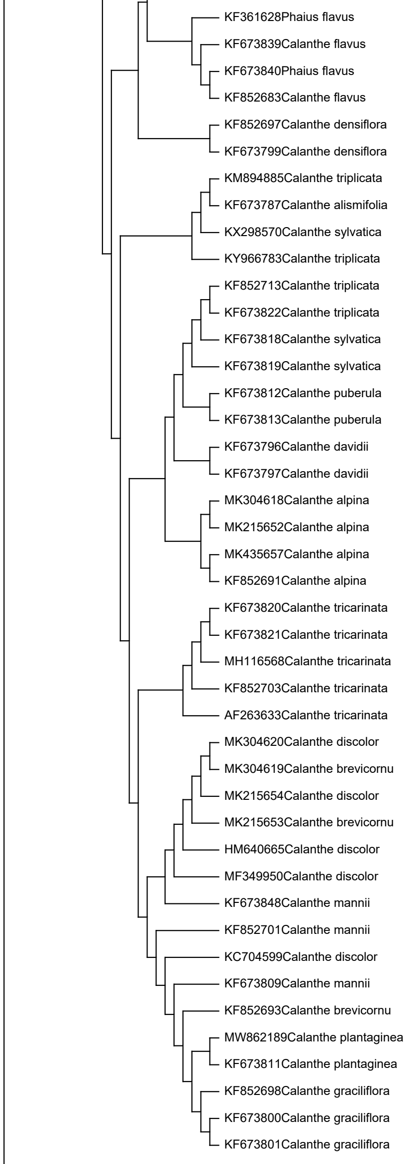
KF361628Phaius flavus
KF673839Calanthe flavus
KF673840Phaius flavus
KF852683Calanthe flavus
KF852697Calanthe densiflora
KF673799Calanthe densiflora
KM894885Calanthe triplicata
KF673787Calanthe alismifolia
KX298570Calanthe sylvatica
KY966783Calanthe triplicata
KF852713Calanthe triplicata
KF673822Calanthe triplicata
KF673818Calanthe sylvatica
KF673819Calanthe sylvatica
KF673812Calanthe puberula
KF673813Calanthe puberula
KF673796Calanthe davidii
KF673797Calanthe davidii
MK304618Calanthe alpina
MK215652Calanthe alpina
MK435657Calanthe alpina
KF852691Calanthe alpina
KF673820Calanthe tricarinata
KF673821Calanthe tricarinata
MH116568Calanthe tricarinata
KF852703Calanthe tricarinata
AF263633Calanthe tricarinata
MK304620Calanthe discolor
MK304619Calanthe brevicornu
MK215654Calanthe discolor
MK215653Calanthe brevicornu
HM640665Calanthe discolor
MF349950Calanthe discolor
KF673848Calanthe mannii
KF852701Calanthe mannii
KC704599Calanthe discolor
KF673809Calanthe mannii
KF852693Calanthe brevicornu
MW862189Calanthe plantaginea
KF673811Calanthe plantaginea
KF852698Calanthe graciliflora
KF673800Calanthe graciliflora
KF673801Calanthe graciliflora

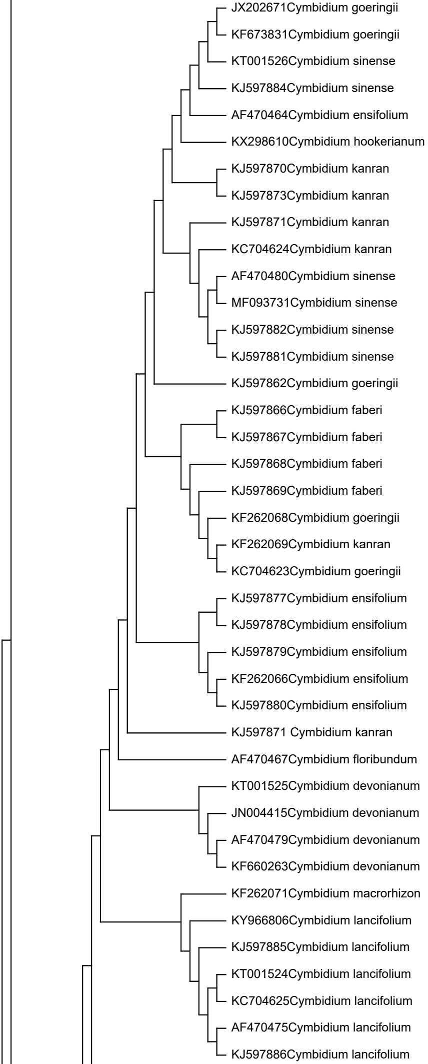
JX202671Cymbidium goeringii
KF673831Cymbidium goeringii
KT001526Cymbidium sinense
KJ597884Cymbidium sinense
AF470464Cymbidium ensifolium
KX298610Cymbidium hookerianum
KJ597870Cymbidium kanran
KJ597873Cymbidium kanran
KJ597871Cymbidium kanran
KC704624Cymbidium kanran
AF470480Cymbidium sinense
MF093731Cymbidium sinense
KJ597882Cymbidium sinense
KJ597881Cymbidium sinense
KJ597862Cymbidium goeringii
KJ597866Cymbidium faberi
KJ597867Cymbidium faberi
KJ597868Cymbidium faberi
KJ597869Cymbidium faberi
KF262068Cymbidium goeringii
KF262069Cymbidium kanran
KC704623Cymbidium goeringii
KJ597877Cymbidium ensifolium
KJ597878Cymbidium ensifolium
KJ597879Cymbidium ensifolium
KF262066Cymbidium ensifolium
KJ597880Cymbidium ensifolium
KJ597871 Cymbidium kanran
AF470467Cymbidium floribundum
KT001525Cymbidium devonianum
JN004415Cymbidium devonianum
AF470479Cymbidium devonianum
KF660263Cymbidium devonianum
KF262071Cymbidium macrorhizon
KY966806Cymbidium lancifolium
KJ597885Cymbidium lancifolium
KT001524Cymbidium lancifolium
KC704625Cymbidium lancifolium
AF470475Cymbidium lancifolium
KJ597886Cymbidium lancifolium

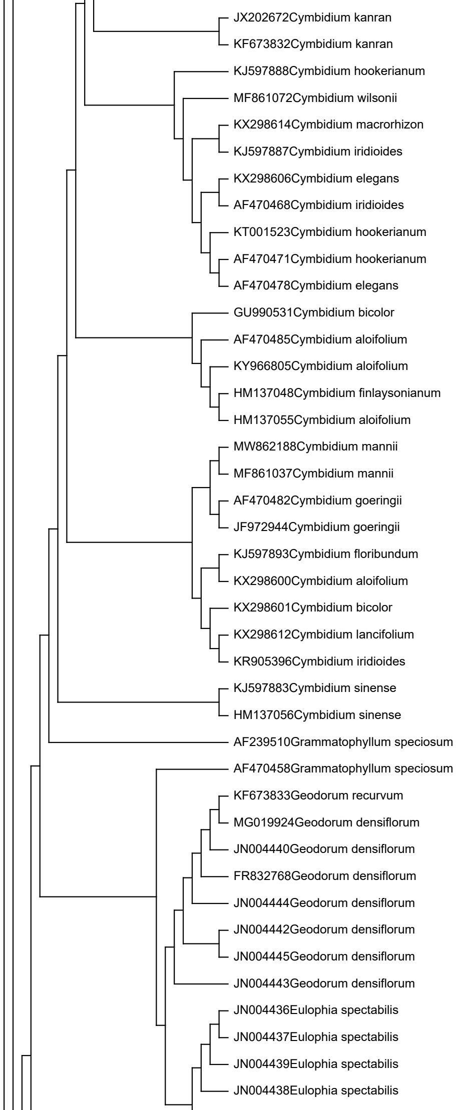
JX202672Cymbidium kanran
KF673832Cymbidium kanran
KJ597888Cymbidium hookerianum
MF861072Cymbidium wilsonii
KX298614Cymbidium macrorhizon
KJ597887Cymbidium iridioides
KX298606Cymbidium elegans
AF470468Cymbidium iridioides
KT001523Cymbidium hookerianum
AF470471Cymbidium hookerianum
AF470478Cymbidium elegans
GU990531Cymbidium bicolor
AF470485Cymbidium aloifolium
KY966805Cymbidium aloifolium
HM137048Cymbidium finlaysonianum
HM137055Cymbidium aloifolium
MW862188Cymbidium mannii
MF861037Cymbidium mannii
AF470482Cymbidium goeringii
JF972944Cymbidium goeringii
KJ597893Cymbidium floribundum
KX298600Cymbidium aloifolium
KX298601Cymbidium bicolor
KX298612Cymbidium lancifolium
KR905396Cymbidium iridioides
KJ597883Cymbidium sinense
HM137056Cymbidium sinense
AF239510Grammatophyllum speciosum
AF470458Grammatophyllum speciosum
KF673833Geodorum recurvum
MG019924Geodorum densiflorum
JN004440Geodorum densiflorum
FR832768Geodorum densiflorum
JN004444Geodorum densiflorum
JN004442Geodorum densiflorum
JN004445Geodorum densiflorum
JN004443Geodorum densiflorum
JN004436Eulophia spectabilis
JN004437Eulophia spectabilis
JN004439Eulophia spectabilis
JN004438Eulophia spectabilis

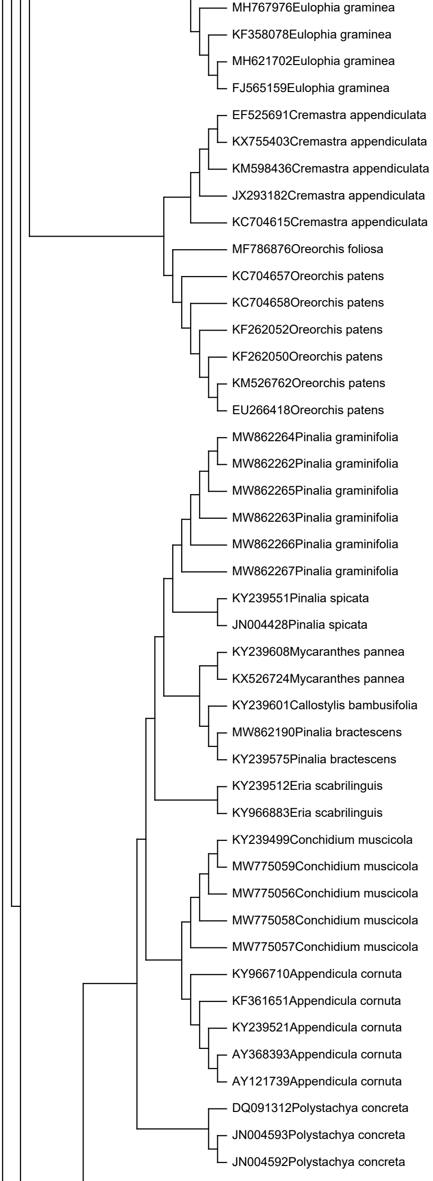
MH767976Eulophia graminea
KF358078Eulophia graminea
MH621702Eulophia graminea
FJ565159Eulophia graminea
EF525691Cremastra appendiculata
KX755403Cremastra appendiculata
KM598436Cremastra appendiculata
JX293182Cremastra appendiculata
KC704615Cremastra appendiculata
MF786876Oreorchis foliosa
KC704657Oreorchis patens
KC704658Oreorchis patens
KF262052Oreorchis patens
KF262050Oreorchis patens
KM526762Oreorchis patens
EU266418Oreorchis patens
MW862264Pinalia graminifolia
MW862262Pinalia graminifolia
MW862265Pinalia graminifolia
MW862263Pinalia graminifolia
MW862266Pinalia graminifolia
MW862267Pinalia graminifolia
KY239551Pinalia spicata
JN004428Pinalia spicata
KY239608Mycaranthes pannea
KX526724Mycaranthes pannea
KY239601Callostylis bambusifolia
MW862190Pinalia bractescens
KY239575Pinalia bractescens
KY239512Eria scabrilinguis
KY966883Eria scabrilinguis
KY239499Conchidium muscicola
MW775059Conchidium muscicola
MW775056Conchidium muscicola
MW775058Conchidium muscicola
MW775057Conchidium muscicola
KY966710Appendicula cornuta
KF361651Appendicula cornuta
KY239521Appendicula cornuta
AY368393Appendicula cornuta
AY121739Appendicula cornuta
DQ091312Polystachya concreta
JN004593Polystachya concreta
JN004592Polystachya concreta

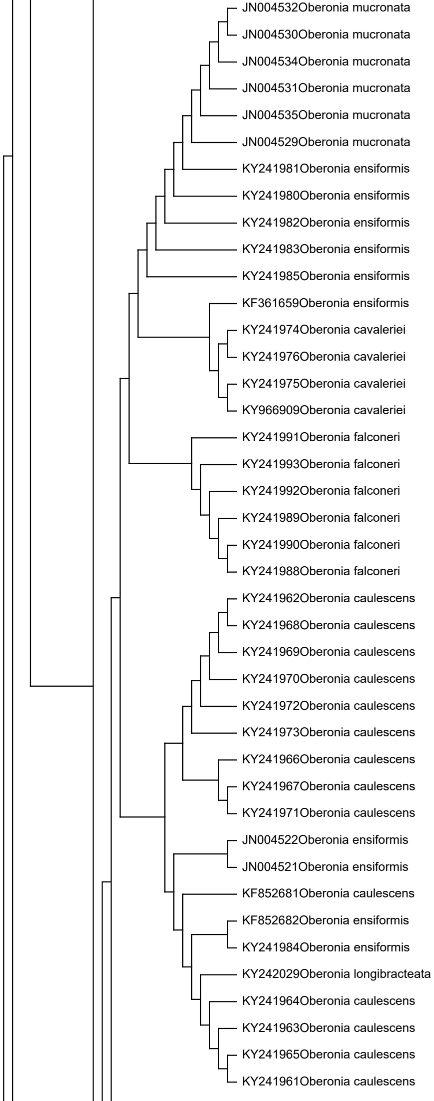
JN004532Oberonia mucronata
JN004530Oberonia mucronata
JN004534Oberonia mucronata
JN004531Oberonia mucronata
JN004535Oberonia mucronata
JN004529Oberonia mucronata
KY241981Oberonia ensiformis
KY241980Oberonia ensiformis
KY241982Oberonia ensiformis
KY241983Oberonia ensiformis
KY241985Oberonia ensiformis
KF361659Oberonia ensiformis
KY241974Oberonia cavaleriei
KY241976Oberonia cavaleriei
KY241975Oberonia cavaleriei
KY966909Oberonia cavaleriei
KY241991Oberonia falconeri
KY241993Oberonia falconeri
KY241992Oberonia falconeri
KY241989Oberonia falconeri
KY241990Oberonia falconeri
KY241988Oberonia falconeri
KY241962Oberonia caulescens
KY241968Oberonia caulescens
KY241969Oberonia caulescens
KY241970Oberonia caulescens
KY241972Oberonia caulescens
KY241973Oberonia caulescens
KY241966Oberonia caulescens
KY241967Oberonia caulescens
KY241971Oberonia caulescens
JN004522Oberonia ensiformis
JN004521Oberonia ensiformis
KF852681Oberonia caulescens
KF852682Oberonia ensiformis
KY241984Oberonia ensiformis
KY242029Oberonia longibracteata
KY241964Oberonia caulescens
KY241963Oberonia caulescens
KY241965Oberonia caulescens
KY241961Oberonia caulescens

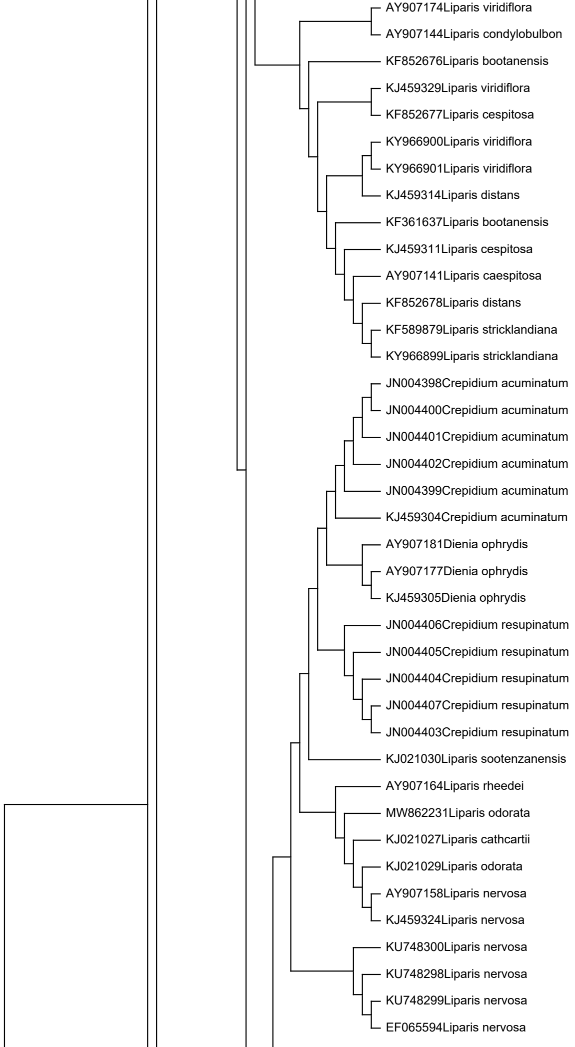
AY907174Liparis viridiflora
AY907144Liparis condylobulbon
KF852676Liparis bootanensis
KJ459329Liparis viridiflora
KF852677Liparis cespitosa
KY966900Liparis viridiflora
KY966901Liparis viridiflora
KJ459314Liparis distans
KF361637Liparis bootanensis
KJ459311Liparis cespitosa
AY907141Liparis caespitosa
KF852678Liparis distans
KF589879Liparis stricklandiana
KY966899Liparis stricklandiana
JN004398Crepidium acuminatum
JN004400Crepidium acuminatum
JN004401Crepidium acuminatum
JN004402Crepidium acuminatum
JN004399Crepidium acuminatum
KJ459304Crepidium acuminatum
AY907181Dienia ophrydis
AY907177Dienia ophrydis
KJ459305Dienia ophrydis
JN004406Crepidium resupinatum
JN004405Crepidium resupinatum
JN004404Crepidium resupinatum
JN004407Crepidium resupinatum
JN004403Crepidium resupinatum
KJ021030Liparis sootenzanensis
AY907164Liparis rheedei
MW862231Liparis odorata
KJ021027Liparis cathcartii
KJ021029Liparis odorata
AY907158Liparis nervosa
KJ459324Liparis nervosa
KU748300Liparis nervosa
KU748298Liparis nervosa
KU748299Liparis nervosa
EF065594Liparis nervosa

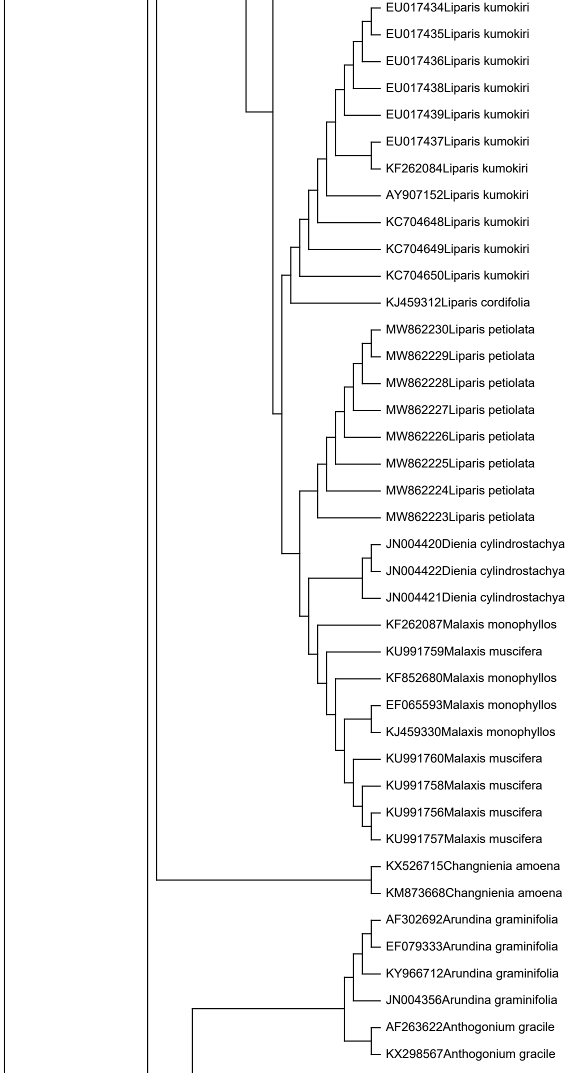
EU017434Liparis kumokiri
EU017435Liparis kumokiri
EU017436Liparis kumokiri
EU017438Liparis kumokiri
EU017439Liparis kumokiri
EU017437Liparis kumokiri
KF262084Liparis kumokiri
AY907152Liparis kumokiri
KC704648Liparis kumokiri
KC704649Liparis kumokiri
KC704650Liparis kumokiri
KJ459312Liparis cordifolia
MW862230Liparis petiolata
MW862229Liparis petiolata
MW862228Liparis petiolata
MW862227Liparis petiolata
MW862226Liparis petiolata
MW862225Liparis petiolata
MW862224Liparis petiolata
MW862223Liparis petiolata
JN004420Dienia cylindrostachya
JN004422Dienia cylindrostachya
JN004421Dienia cylindrostachya
KF262087Malaxis monophyllos
KU991759Malaxis muscifera
KF852680Malaxis monophyllos
EF065593Malaxis monophyllos
KJ459330Malaxis monophyllos
KU991760Malaxis muscifera
KU991758Malaxis muscifera
KU991756Malaxis muscifera
KU991757Malaxis muscifera
KX526715Changnienia amoena
KM873668Changnienia amoena
AF302692Arundina graminifolia
EF079333Arundina graminifolia
KY966712Arundina graminifolia
JN004356Arundina graminifolia
AF263622Anthogonium gracile
KX298567Anthogonium gracile

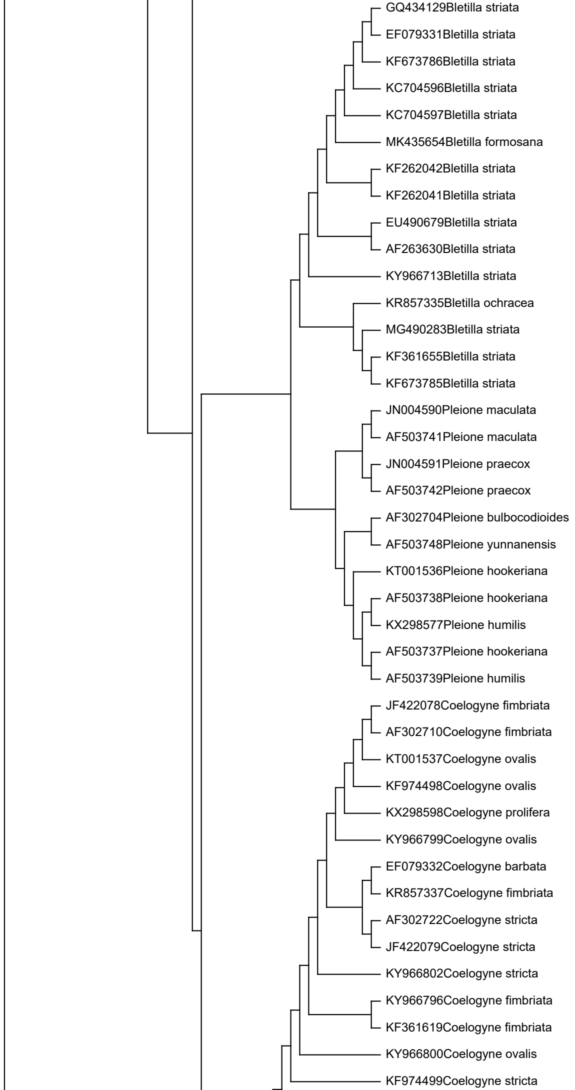

GQ434129Bletilla striata
EF079331Bletilla striata
KF673786Bletilla striata
KC704596Bletilla striata
KC704597Bletilla striata
MK435654Bletilla formosana
KF262042Bletilla striata
KF262041Bletilla striata
EU490679Bletilla striata
AF263630Bletilla striata
KY966713Bletilla striata
KR857335Bletilla ochracea
MG490283Bletilla striata
KF361655Bletilla striata
KF673785Bletilla striata
JN004590Pleione maculata
AF503741Pleione maculata
JN004591Pleione praecox
AF503742Pleione praecox
AF302704Pleione bulbocodioides
AF503748Pleione yunnanensis
KT001536Pleione hookeriana
AF503738Pleione hookeriana
KX298577Pleione humilis
AF503737Pleione hookeriana
AF503739Pleione humilis
JF422078Coelogyne fimbriata
AF302710Coelogyne fimbriata
KT001537Coelogyne ovalis
KF974498Coelogyne ovalis
KX298598Coelogyne prolifera
KY966799Coelogyne ovalis
EF079332Coelogyne barbata
KR857337Coelogyne fimbriata
AF302722Coelogyne stricta
JF422079Coelogyne stricta
KY966802Coelogyne stricta
KY966796Coelogyne fimbriata
KF361619Coelogyne fimbriata
KY966800Coelogyne ovalis
KF974499Coelogyne stricta

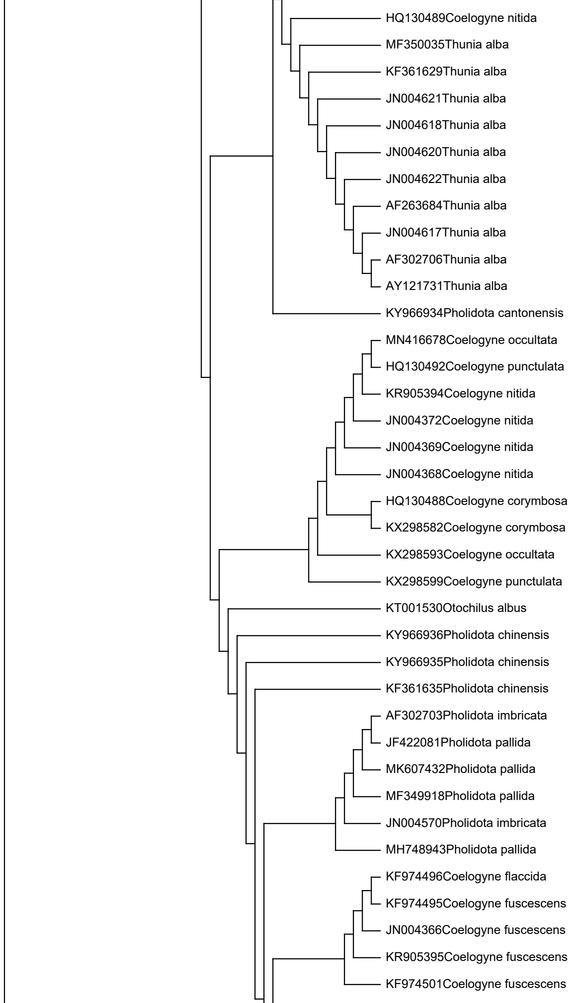
HQ130489Coelogyne nitida
MF350035Thunia alba
KF361629Thunia alba
JN004621Thunia alba
JN004618Thunia alba
JN004620Thunia alba
JN004622Thunia alba
AF263684Thunia alba
JN004617Thunia alba
AF302706Thunia alba
AY121731Thunia alba
KY966934Pholidota cantonensis
MN416678Coelogyne occultata
HQ130492Coelogyne punctulata
KR905394Coelogyne nitida
JN004372Coelogyne nitida
JN004369Coelogyne nitida
JN004368Coelogyne nitida
HQ130488Coelogyne corymbosa
KX298582Coelogyne corymbosa
KX298593Coelogyne occultata
KX298599Coelogyne punctulata
KT001530Otochilus albus
KY966936Pholidota chinensis
KY966935Pholidota chinensis
KF361635Pholidota chinensis
AF302703Pholidota imbricata
JF422081Pholidota pallida
MK607432Pholidota pallida
MF349918Pholidota pallida
JN004570Pholidota imbricata
MH748943Pholidota pallida
KF974496Coelogyne flaccida
KF974495Coelogyne fuscescens
JN004366Coelogyne fuscescens
KR905395Coelogyne fuscescens
KF974501Coelogyne fuscescens

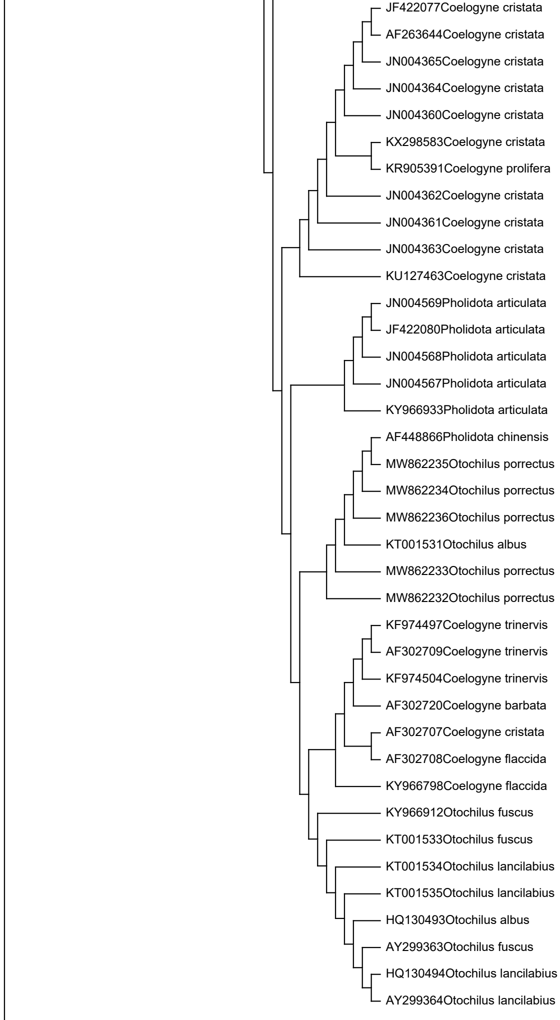
JF422077Coelogyne cristata
AF263644Coelogyne cristata
JN004365Coelogyne cristata
JN004364Coelogyne cristata
JN004360Coelogyne cristata
KX298583Coelogyne cristata
KR905391Coelogyne prolifera
JN004362Coelogyne cristata
JN004361Coelogyne cristata
JN004363Coelogyne cristata
KU127463Coelogyne cristata
JN004569Pholidota articulata
JF422080Pholidota articulata
JN004568Pholidota articulata
JN004567Pholidota articulata
KY966933Pholidota articulata
AF448866Pholidota chinensis
MW862235Otochilus porrectus
MW862234Otochilus porrectus
MW862236Otochilus porrectus
KT001531Otochilus albus
MW862233Otochilus porrectus
MW862232Otochilus porrectus
KF974497Coelogyne trinervis
AF302709Coelogyne trinervis
KF974504Coelogyne trinervis
AF302720Coelogyne barbata
AF302707Coelogyne cristata
AF302708Coelogyne flaccida
KY966798Coelogyne flaccida
KY966912Otochilus fuscus
KT001533Otochilus fuscus
KT001534Otochilus lancilabius
KT001535Otochilus lancilabius
HQ130493Otochilus albus
AY299363Otochilus fuscus
HQ130494Otochilus lancilabius
AY299364Otochilus lancilabius

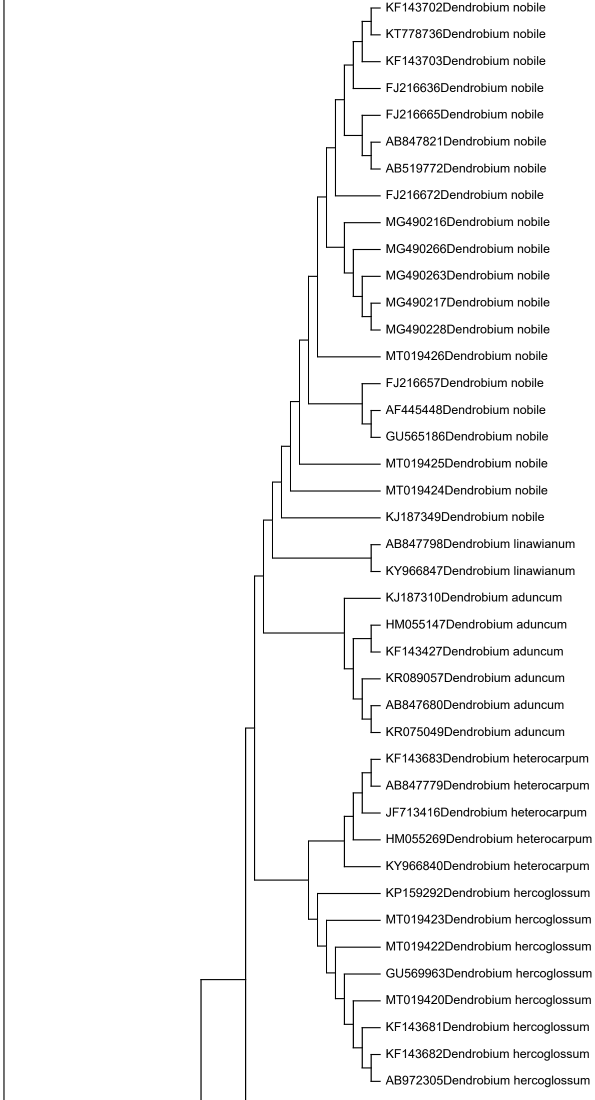
KF143702Dendrobium nobile
KT778736Dendrobium nobile
KF143703Dendrobium nobile
FJ216636Dendrobium nobile
FJ216665Dendrobium nobile
AB847821Dendrobium nobile
AB519772Dendrobium nobile
FJ216672Dendrobium nobile
MG490216Dendrobium nobile
MG490266Dendrobium nobile
MG490263Dendrobium nobile
MG490217Dendrobium nobile
MG490228Dendrobium nobile
MT019426Dendrobium nobile
FJ216657Dendrobium nobile
AF445448Dendrobium nobile
GU565186Dendrobium nobile
MT019425Dendrobium nobile
MT019424Dendrobium nobile
KJ187349Dendrobium nobile
AB847798Dendrobium linawianum
KY966847Dendrobium linawianum
KJ187310Dendrobium aduncum
HM055147Dendrobium aduncum
KF143427Dendrobium aduncum
KR089057Dendrobium aduncum
AB847680Dendrobium aduncum
KR075049Dendrobium aduncum
KF143683Dendrobium heterocarpum
AB847779Dendrobium heterocarpum
JF713416Dendrobium heterocarpum
HM055269Dendrobium heterocarpum
KY966840Dendrobium heterocarpum
KP159292Dendrobium hercoglossum
MT019423Dendrobium hercoglossum
MT019422Dendrobium hercoglossum
GU569963Dendrobium hercoglossum
MT019420Dendrobium hercoglossum
KF143681Dendrobium hercoglossum
KF143682Dendrobium hercoglossum
AB972305Dendrobium hercoglossum

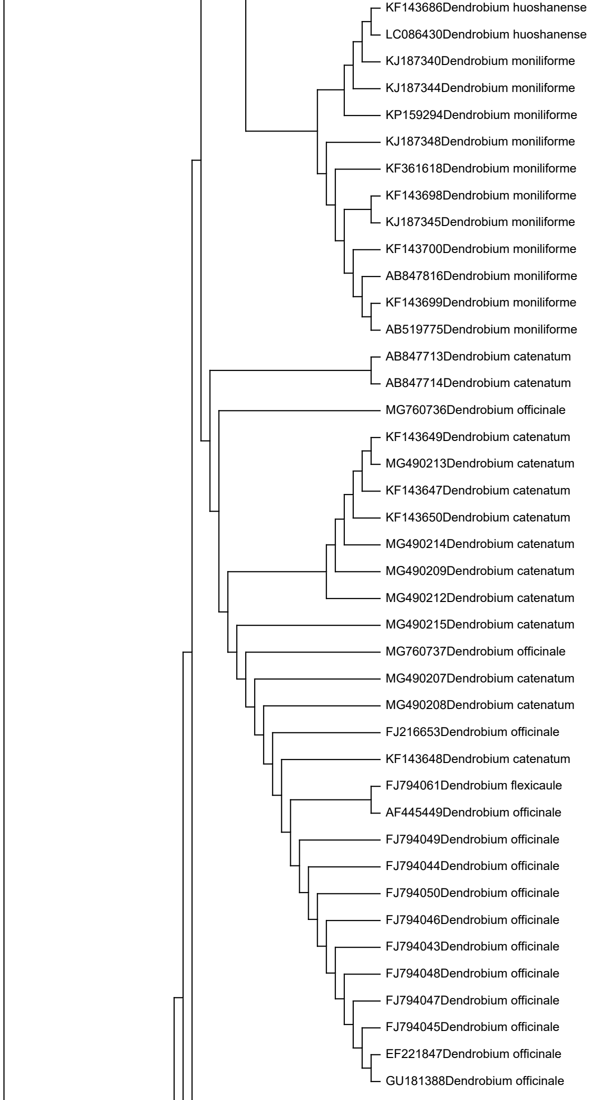
KF143686Dendrobium huoshanense
LC086430Dendrobium huoshanense
KJ187340Dendrobium moniliforme
KJ187344Dendrobium moniliforme
KP159294Dendrobium moniliforme
KJ187348Dendrobium moniliforme
KF361618Dendrobium moniliforme
KF143698Dendrobium moniliforme
KJ187345Dendrobium moniliforme
KF143700Dendrobium moniliforme
AB847816Dendrobium moniliforme
KF143699Dendrobium moniliforme
AB519775Dendrobium moniliforme
AB847713Dendrobium catenatum
AB847714Dendrobium catenatum
MG760736Dendrobium officinale
KF143649Dendrobium catenatum
MG490213Dendrobium catenatum
KF143647Dendrobium catenatum
KF143650Dendrobium catenatum
MG490214Dendrobium catenatum
MG490209Dendrobium catenatum
MG490212Dendrobium catenatum
MG490215Dendrobium catenatum
MG760737Dendrobium officinale
MG490207Dendrobium catenatum
MG490208Dendrobium catenatum
FJ216653Dendrobium officinale
KF143648Dendrobium catenatum
FJ794061Dendrobium flexicaule
AF445449Dendrobium officinale
FJ794049Dendrobium officinale
FJ794044Dendrobium officinale
FJ794050Dendrobium officinale
FJ794046Dendrobium officinale
FJ794043Dendrobium officinale
FJ794048Dendrobium officinale
FJ794047Dendrobium officinale
FJ794045Dendrobium officinale
EF221847Dendrobium officinale
GU181388Dendrobium officinale

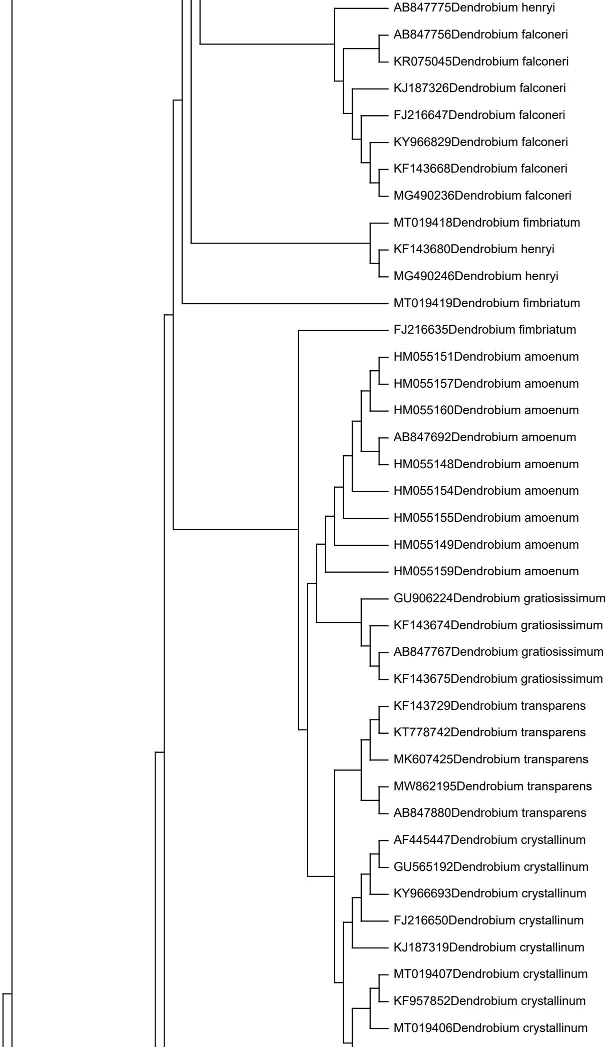
AB847775Dendrobium henryi
AB847756Dendrobium falconeri
KR075045Dendrobium falconeri
KJ187326Dendrobium falconeri
FJ216647Dendrobium falconeri
KY966829Dendrobium falconeri
KF143668Dendrobium falconeri
MG490236Dendrobium falconeri
MT019418Dendrobium fimbriatum
KF143680Dendrobium henryi
MG490246Dendrobium henryi
MT019419Dendrobium fimbriatum
FJ216635Dendrobium fimbriatum
HM055151Dendrobium amoenum
HM055157Dendrobium amoenum
HM055160Dendrobium amoenum
AB847692Dendrobium amoenum
HM055148Dendrobium amoenum
HM055154Dendrobium amoenum
HM055155Dendrobium amoenum
HM055149Dendrobium amoenum
HM055159Dendrobium amoenum
GU906224Dendrobium gratiosissimum
KF143674Dendrobium gratiosissimum
AB847767Dendrobium gratiosissimum
KF143675Dendrobium gratiosissimum
KF143729Dendrobium transparens
KT778742Dendrobium transparens
MK607425Dendrobium transparens
MW862195Dendrobium transparens
AB847880Dendrobium transparens
AF445447Dendrobium crystallinum
GU565192Dendrobium crystallinum
KY966693Dendrobium crystallinum
FJ216650Dendrobium crystallinum
KJ187319Dendrobium crystallinum
MT019407Dendrobium crystallinum
KF957852Dendrobium crystallinum
MT019406Dendrobium crystallinum

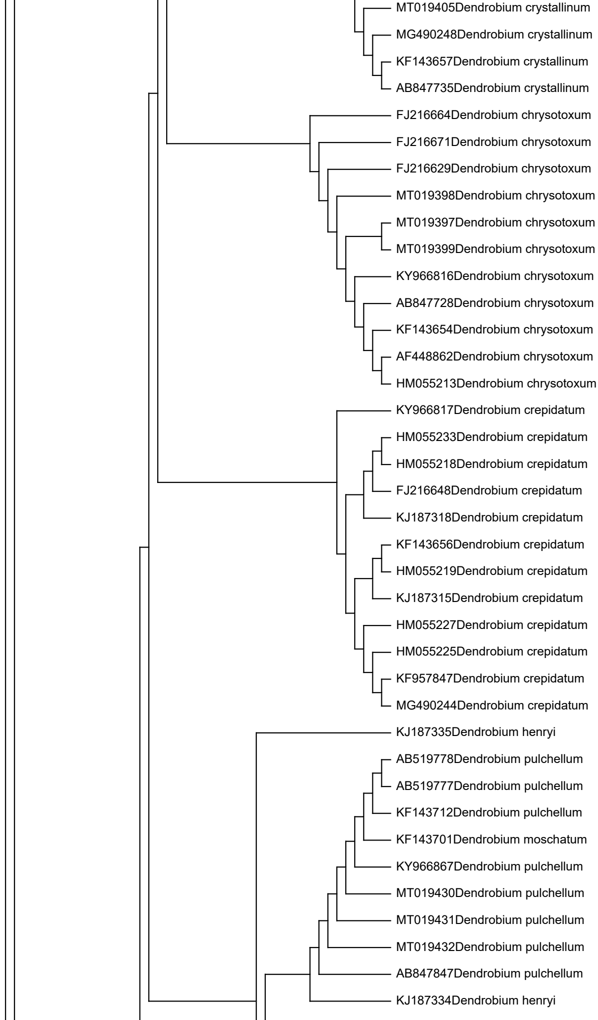
MT019405Dendrobium crystallinum
MG490248Dendrobium crystallinum
KF143657Dendrobium crystallinum
AB847735Dendrobium crystallinum
FJ216664Dendrobium chrysotoxum
FJ216671Dendrobium chrysotoxum
FJ216629Dendrobium chrysotoxum
MT019398Dendrobium chrysotoxum
MT019397Dendrobium chrysotoxum
MT019399Dendrobium chrysotoxum
KY966816Dendrobium chrysotoxum
AB847728Dendrobium chrysotoxum
KF143654Dendrobium chrysotoxum
AF448862Dendrobium chrysotoxum
HM055213Dendrobium chrysotoxum
KY966817Dendrobium crepidatum
HM055233Dendrobium crepidatum
HM055218Dendrobium crepidatum
FJ216648Dendrobium crepidatum
KJ187318Dendrobium crepidatum
KF143656Dendrobium crepidatum
HM055219Dendrobium crepidatum
KJ187315Dendrobium crepidatum
HM055227Dendrobium crepidatum
HM055225Dendrobium crepidatum
KF957847Dendrobium crepidatum
MG490244Dendrobium crepidatum
KJ187335Dendrobium henryi
AB519778Dendrobium pulchellum
AB519777Dendrobium pulchellum
KF143712Dendrobium pulchellum
KF143701Dendrobium moschatum
KY966867Dendrobium pulchellum
MT019430Dendrobium pulchellum
MT019431Dendrobium pulchellum
MT019432Dendrobium pulchellum
AB847847Dendrobium pulchellum
KJ187334Dendrobium henryi

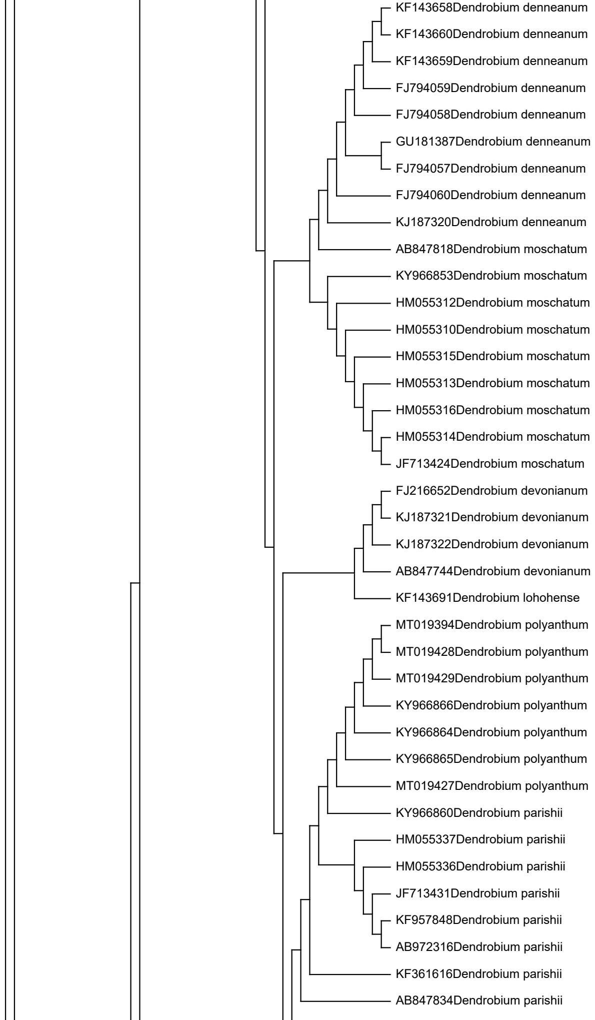
KF143658Dendrobium denneanum
KF143660Dendrobium denneanum
KF143659Dendrobium denneanum
FJ794059Dendrobium denneanum
FJ794058Dendrobium denneanum
GU181387Dendrobium denneanum
FJ794057Dendrobium denneanum
FJ794060Dendrobium denneanum
KJ187320Dendrobium denneanum
AB847818Dendrobium moschatum
KY966853Dendrobium moschatum
HM055312Dendrobium moschatum
HM055310Dendrobium moschatum
HM055315Dendrobium moschatum
HM055313Dendrobium moschatum
HM055316Dendrobium moschatum
HM055314Dendrobium moschatum
JF713424Dendrobium moschatum
FJ216652Dendrobium devonianum
KJ187321Dendrobium devonianum
KJ187322Dendrobium devonianum
AB847744Dendrobium devonianum
KF143691Dendrobium lohohense
MT019394Dendrobium polyanthum
MT019428Dendrobium polyanthum
MT019429Dendrobium polyanthum
KY966866Dendrobium polyanthum
KY966864Dendrobium polyanthum
KY966865Dendrobium polyanthum
MT019427Dendrobium polyanthum
KY966860Dendrobium parishii
HM055337Dendrobium parishii
HM055336Dendrobium parishii
JF713431Dendrobium parishii
KF957848Dendrobium parishii
AB972316Dendrobium parishii
KF361616Dendrobium parishii
AB847834Dendrobium parishii

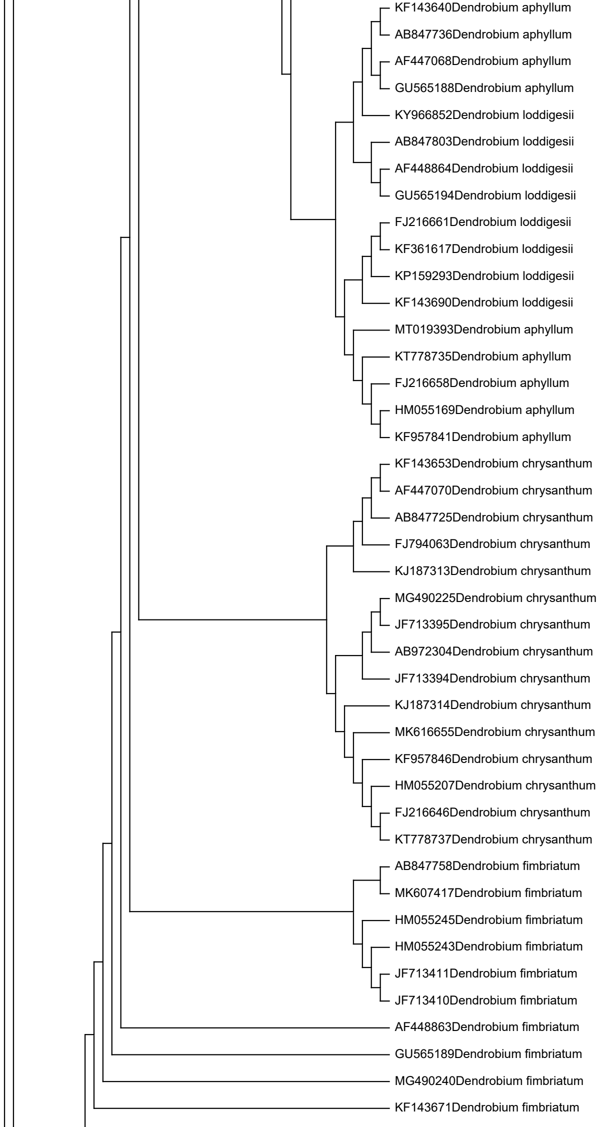
KF143640Dendrobium aphyllum
AB847736Dendrobium aphyllum
AF447068Dendrobium aphyllum
GU565188Dendrobium aphyllum
KY966852Dendrobium loddigesii
AB847803Dendrobium loddigesii
AF448864Dendrobium loddigesii
GU565194Dendrobium loddigesii
FJ216661Dendrobium loddigesii
KF361617Dendrobium loddigesii
KP159293Dendrobium loddigesii
KF143690Dendrobium loddigesii
MT019393Dendrobium aphyllum
KT778735Dendrobium aphyllum
FJ216658Dendrobium aphyllum
HM055169Dendrobium aphyllum
KF957841Dendrobium aphyllum
KF143653Dendrobium chrysanthum
AF447070Dendrobium chrysanthum
AB847725Dendrobium chrysanthum
FJ794063Dendrobium chrysanthum
KJ187313Dendrobium chrysanthum
MG490225Dendrobium chrysanthum
JF713395Dendrobium chrysanthum
AB972304Dendrobium chrysanthum
JF713394Dendrobium chrysanthum
KJ187314Dendrobium chrysanthum
MK616655Dendrobium chrysanthum
KF957846Dendrobium chrysanthum
HM055207Dendrobium chrysanthum
FJ216646Dendrobium chrysanthum
KT778737Dendrobium chrysanthum
AB847758Dendrobium fimbriatum
MK607417Dendrobium fimbriatum
HM055245Dendrobium fimbriatum
HM055243Dendrobium fimbriatum
JF713411Dendrobium fimbriatum
JF713410Dendrobium fimbriatum
AF448863Dendrobium fimbriatum
GU565189Dendrobium fimbriatum
MG490240Dendrobium fimbriatum
KF143671Dendrobium fimbriatum

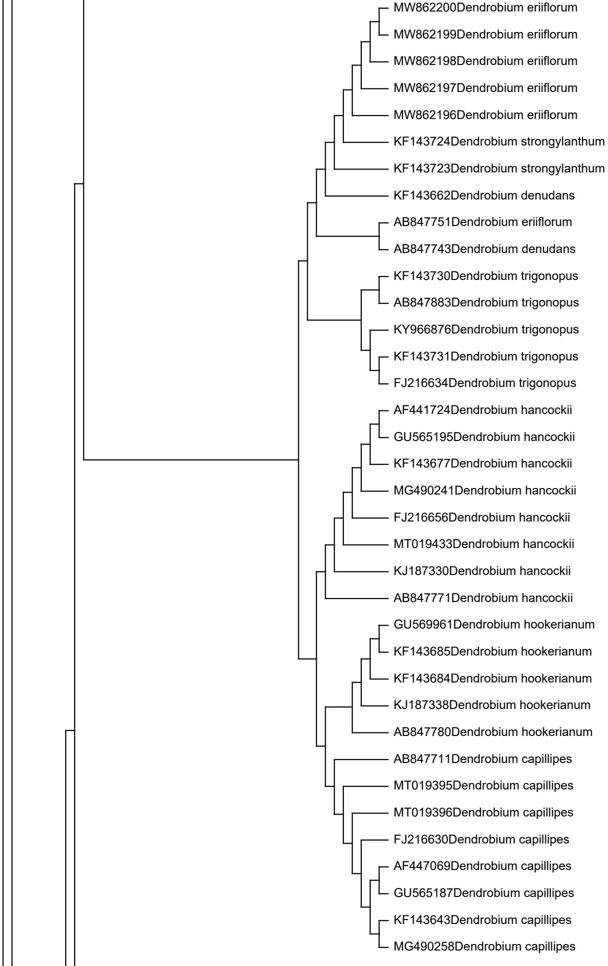

MW862200Dendrobium eriiflorum
MW862199Dendrobium eriiflorum
MW862198Dendrobium eriiflorum
MW862197Dendrobium eriiflorum
MW862196Dendrobium eriiflorum
KF143724Dendrobium strongylanthum
KF143723Dendrobium strongylanthum
KF143662Dendrobium denudans
AB847751Dendrobium eriiflorum
AB847743Dendrobium denudans
KF143730Dendrobium trigonopus
AB847883Dendrobium trigonopus
KY966876Dendrobium trigonopus
KF143731Dendrobium trigonopus
FJ216634Dendrobium trigonopus
AF441724Dendrobium hancockii
GU565195Dendrobium hancockii
KF143677Dendrobium hancockii
MG490241Dendrobium hancockii
FJ216656Dendrobium hancockii
MT019433Dendrobium hancockii
KJ187330Dendrobium hancockii
AB847771Dendrobium hancockii
GU569961Dendrobium hookerianum
KF143685Dendrobium hookerianum
KF143684Dendrobium hookerianum
KJ187338Dendrobium hookerianum
AB847780Dendrobium hookerianum
AB847711Dendrobium capillipes
MT019395Dendrobium capillipes
MT019396Dendrobium capillipes
FJ216630Dendrobium capillipes
AF447069Dendrobium capillipes
GU565187Dendrobium capillipes
KF143643Dendrobium capillipes
MG490258Dendrobium capillipes

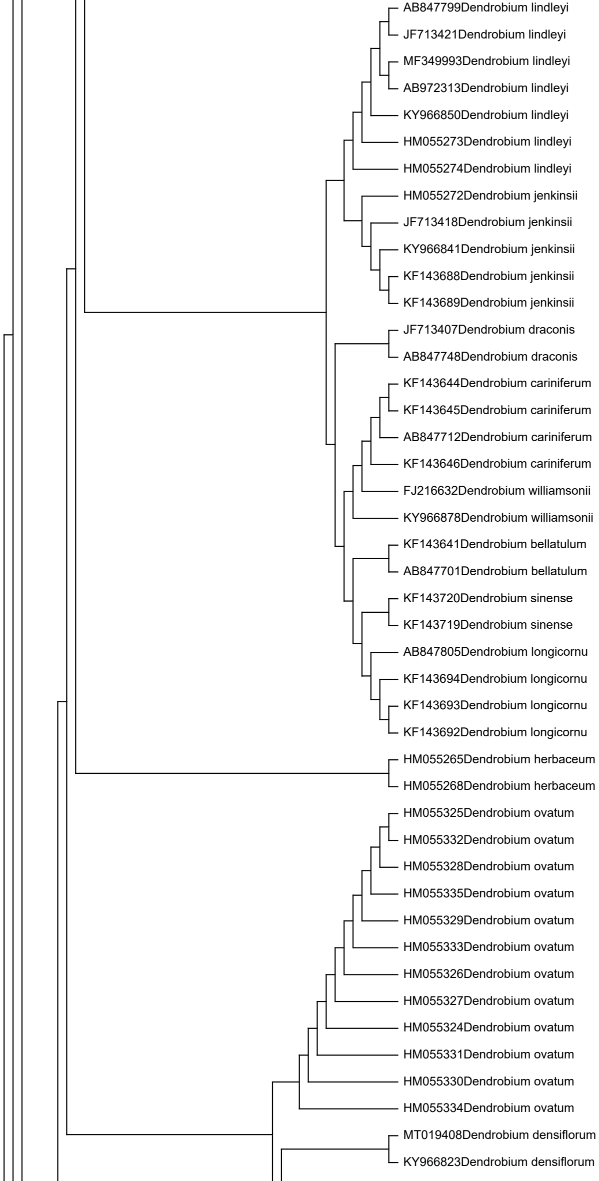

AB847799Dendrobium lindleyi
JF713421Dendrobium lindleyi
MF349993Dendrobium lindleyi
AB972313Dendrobium lindleyi
KY966850Dendrobium lindleyi
HM055273Dendrobium lindleyi
HM055274Dendrobium lindleyi
HM055272Dendrobium jenkinsii
JF713418Dendrobium jenkinsii
KY966841Dendrobium jenkinsii
KF143688Dendrobium jenkinsii
KF143689Dendrobium jenkinsii
JF713407Dendrobium draconis
AB847748Dendrobium draconis
KF143644Dendrobium cariniferum
KF143645Dendrobium cariniferum
AB847712Dendrobium cariniferum
KF143646Dendrobium cariniferum
FJ216632Dendrobium williamsonii
KY966878Dendrobium williamsonii
KF143641Dendrobium bellatulum
AB847701Dendrobium bellatulum
KF143720Dendrobium sinense
KF143719Dendrobium sinense
AB847805Dendrobium longicornu
KF143694Dendrobium longicornu
KF143693Dendrobium longicornu
KF143692Dendrobium longicornu
HM055265Dendrobium herbaceum
HM055268Dendrobium herbaceum
HM055325Dendrobium ovatum
HM055332Dendrobium ovatum
HM055328Dendrobium ovatum
HM055335Dendrobium ovatum
HM055329Dendrobium ovatum
HM055333Dendrobium ovatum
HM055326Dendrobium ovatum
HM055327Dendrobium ovatum
HM055324Dendrobium ovatum
HM055331Dendrobium ovatum
HM055330Dendrobium ovatum
HM055334Dendrobium ovatum
MT019408Dendrobium densiflorum
KY966823Dendrobium densiflorum

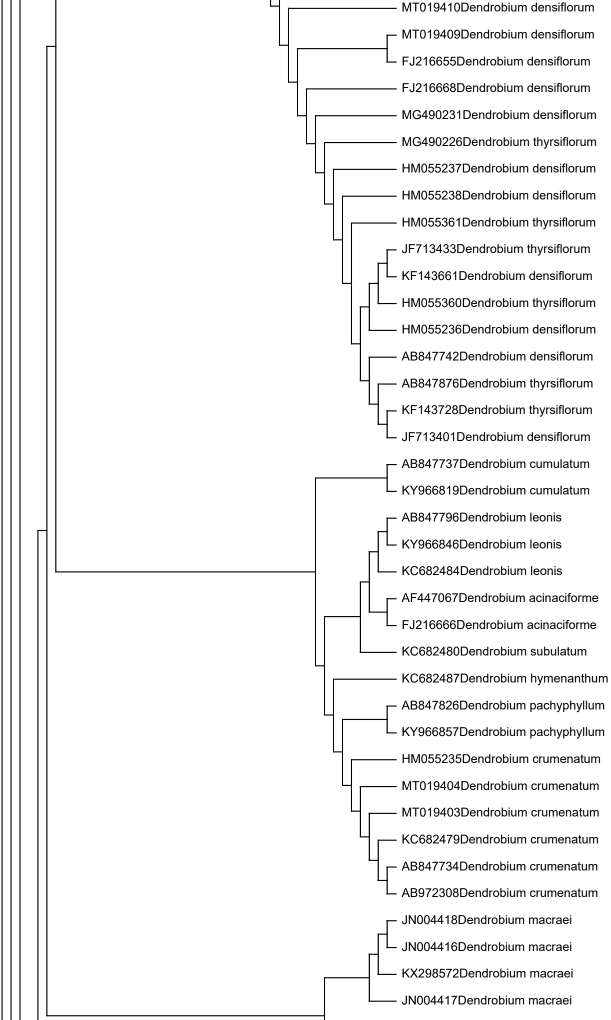
MT019410Dendrobium densiflorum
MT019409Dendrobium densiflorum
FJ216655Dendrobium densiflorum
FJ216668Dendrobium densiflorum
MG490231Dendrobium densiflorum
MG490226Dendrobium thyrsiflorum
HM055237Dendrobium densiflorum
HM055238Dendrobium densiflorum
HM055361Dendrobium thyrsiflorum
JF713433Dendrobium thyrsiflorum
KF143661Dendrobium densiflorum
HM055360Dendrobium thyrsiflorum
HM055236Dendrobium densiflorum
AB847742Dendrobium densiflorum
AB847876Dendrobium thyrsiflorum
KF143728Dendrobium thyrsiflorum
JF713401Dendrobium densiflorum
AB847737Dendrobium cumulatum
KY966819Dendrobium cumulatum
AB847796Dendrobium leonis
KY966846Dendrobium leonis
KC682484Dendrobium leonis
AF447067Dendrobium acinaciforme
FJ216666Dendrobium acinaciforme
KC682480Dendrobium subulatum
KC682487Dendrobium hymenanthum
AB847826Dendrobium pachyphyllum
KY966857Dendrobium pachyphyllum
HM055235Dendrobium crumenatum
MT019404Dendrobium crumenatum
MT019403Dendrobium crumenatum
KC682479Dendrobium crumenatum
AB847734Dendrobium crumenatum
AB972308Dendrobium crumenatum
JN004418Dendrobium macraei
JN004416Dendrobium macraei
KX298572Dendrobium macraei
JN004417Dendrobium macraei

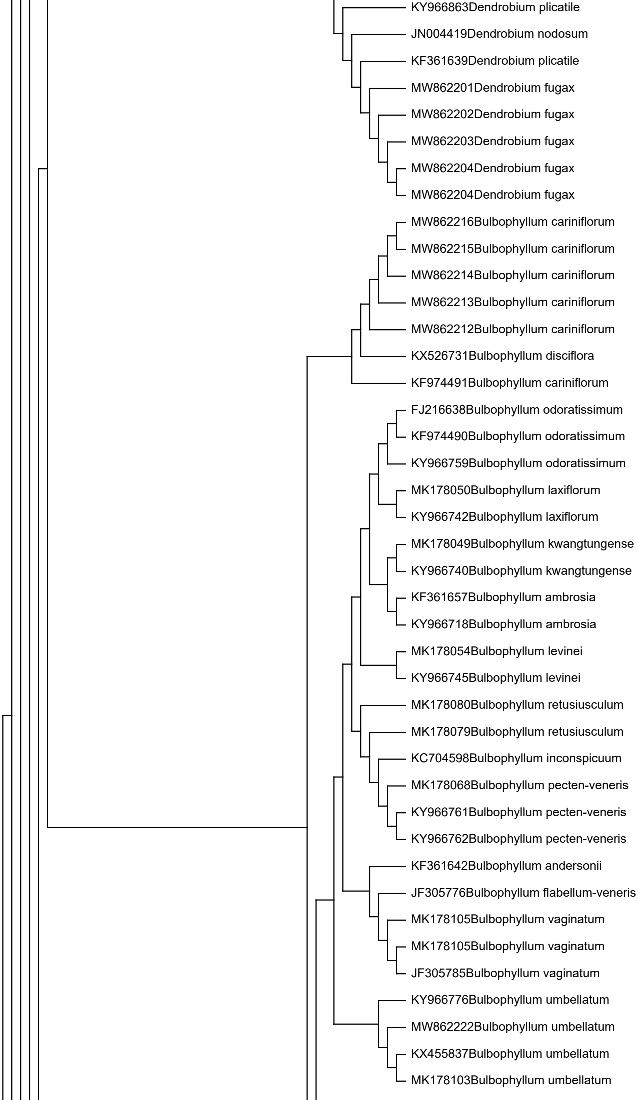
KY966863Dendrobium plicatile
JN004419Dendrobium nodosum
KF361639Dendrobium plicatile
MW862201Dendrobium fugax
MW862202Dendrobium fugax
MW862203Dendrobium fugax
MW862204Dendrobium fugax
MW862204Dendrobium fugax
MW862216Bulbophyllum cariniflorum
MW862215Bulbophyllum cariniflorum
MW862214Bulbophyllum cariniflorum
MW862213Bulbophyllum cariniflorum
MW862212Bulbophyllum cariniflorum
KX526731Bulbophyllum disciflora
KF974491Bulbophyllum cariniflorum
FJ216638Bulbophyllum odoratissimum
KF974490Bulbophyllum odoratissimum
KY966759Bulbophyllum odoratissimum
MK178050Bulbophyllum laxiflorum
KY966742Bulbophyllum laxiflorum
MK178049Bulbophyllum kwangtungense
KY966740Bulbophyllum kwangtungense
KF361657Bulbophyllum ambrosia
KY966718Bulbophyllum ambrosia
MK178054Bulbophyllum levinei
KY966745Bulbophyllum levinei
MK178080Bulbophyllum retusiusculum
MK178079Bulbophyllum retusiusculum
KC704598Bulbophyllum inconspicuum
MK178068Bulbophyllum pecten-veneris
KY966761Bulbophyllum pecten-veneris
KY966762Bulbophyllum pecten-veneris
KF361642Bulbophyllum andersonii
JF305776Bulbophyllum flabellum-veneris
MK178105Bulbophyllum vaginatum
MK178105Bulbophyllum vaginatum
JF305785Bulbophyllum vaginatum
KY966776Bulbophyllum umbellatum
MW862222Bulbophyllum umbellatum
KX455837Bulbophyllum umbellatum
MK178103Bulbophyllum umbellatum

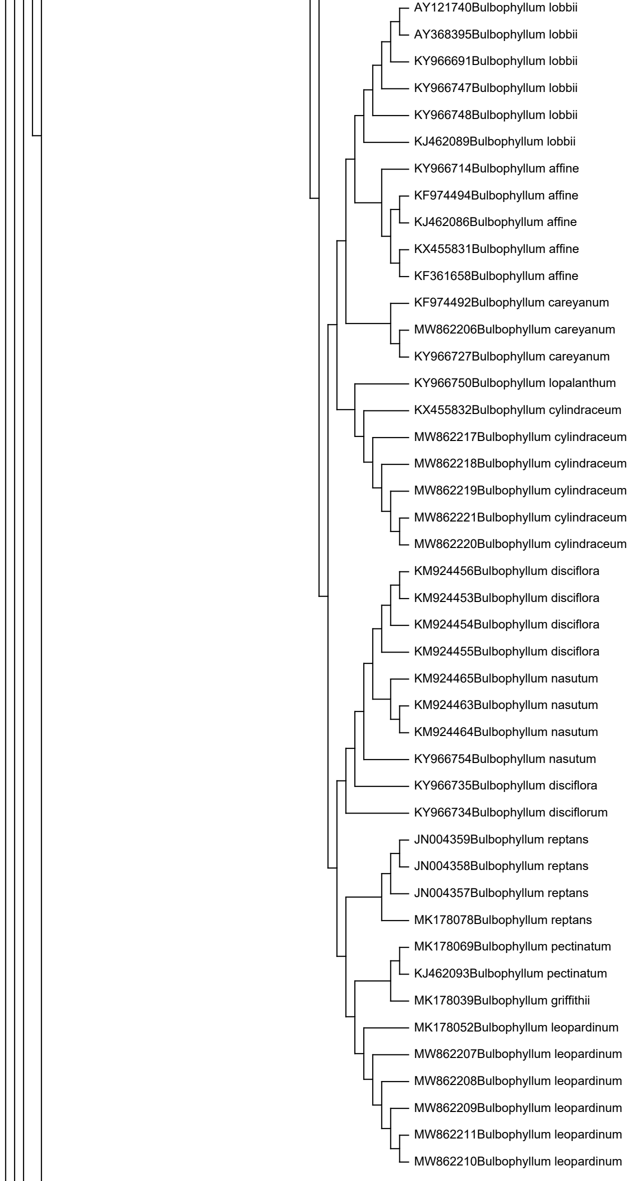
AY121740Bulbophyllum lobbii
AY368395Bulbophyllum lobbii
KY966691Bulbophyllum lobbii
KY966747Bulbophyllum lobbii
KY966748Bulbophyllum lobbii
KJ462089Bulbophyllum lobbii
KY966714Bulbophyllum affine
KF974494Bulbophyllum affine
KJ462086Bulbophyllum affine
KX455831Bulbophyllum affine
KF361658Bulbophyllum affine
KF974492Bulbophyllum careyanum
MW862206Bulbophyllum careyanum
KY966727Bulbophyllum careyanum
KY966750Bulbophyllum lopalanthum
KX455832Bulbophyllum cylindraceum
MW862217Bulbophyllum cylindraceum
MW862218Bulbophyllum cylindraceum
MW862219Bulbophyllum cylindraceum
MW862221Bulbophyllum cylindraceum
MW862220Bulbophyllum cylindraceum
KM924456Bulbophyllum disciflora
KM924453Bulbophyllum disciflora
KM924454Bulbophyllum disciflora
KM924455Bulbophyllum disciflora
KM924465Bulbophyllum nasutum
KM924463Bulbophyllum nasutum
KM924464Bulbophyllum nasutum
KY966754Bulbophyllum nasutum
KY966735Bulbophyllum disciflora
KY966734Bulbophyllum disciflorum
JN004359Bulbophyllum reptans
JN004358Bulbophyllum reptans
JN004357Bulbophyllum reptans
MK178078Bulbophyllum reptans
MK178069Bulbophyllum pectinatum
KJ462093Bulbophyllum pectinatum
MK178039Bulbophyllum griffithii
MK178052Bulbophyllum leopardinum
MW862207Bulbophyllum leopardinum
MW862208Bulbophyllum leopardinum
MW862209Bulbophyllum leopardinum
MW862211Bulbophyllum leopardinum
MW862210Bulbophyllum leopardinum

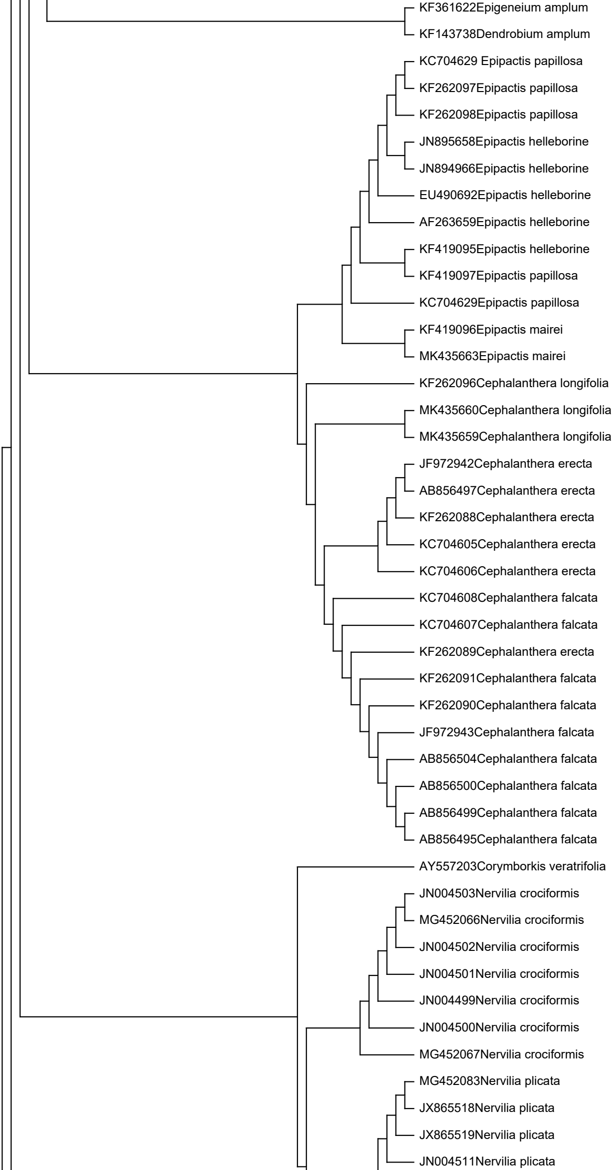
KF361622Epigeneium amplum
KF143738Dendrobium amplum
KC704629 Epipactis papillosa
KF262097Epipactis papillosa
KF262098Epipactis papillosa
JN895658Epipactis helleborine
JN894966Epipactis helleborine
EU490692Epipactis helleborine
AF263659Epipactis helleborine
KF419095Epipactis helleborine
KF419097Epipactis papillosa
KC704629Epipactis papillosa
KF419096Epipactis mairei
MK435663Epipactis mairei
KF262096Cephalanthera longifolia
MK435660Cephalanthera longifolia
MK435659Cephalanthera longifolia
JF972942Cephalanthera erecta
AB856497Cephalanthera erecta
KF262088Cephalanthera erecta
KC704605Cephalanthera erecta
KC704606Cephalanthera erecta
KC704608Cephalanthera falcata
KC704607Cephalanthera falcata
KF262089Cephalanthera erecta
KF262091Cephalanthera falcata
KF262090Cephalanthera falcata
JF972943Cephalanthera falcata
AB856504Cephalanthera falcata
AB856500Cephalanthera falcata
AB856499Cephalanthera falcata
AB856495Cephalanthera falcata
AY557203Corymborkis veratrifolia
JN004503Nervilia crociformis
MG452066Nervilia crociformis
JN004502Nervilia crociformis
JN004501Nervilia crociformis
JN004499Nervilia crociformis
JN004500Nervilia crociformis
MG452067Nervilia crociformis
MG452083Nervilia plicata
JX865518Nervilia plicata
JX865519Nervilia plicata
JN004511Nervilia plicata

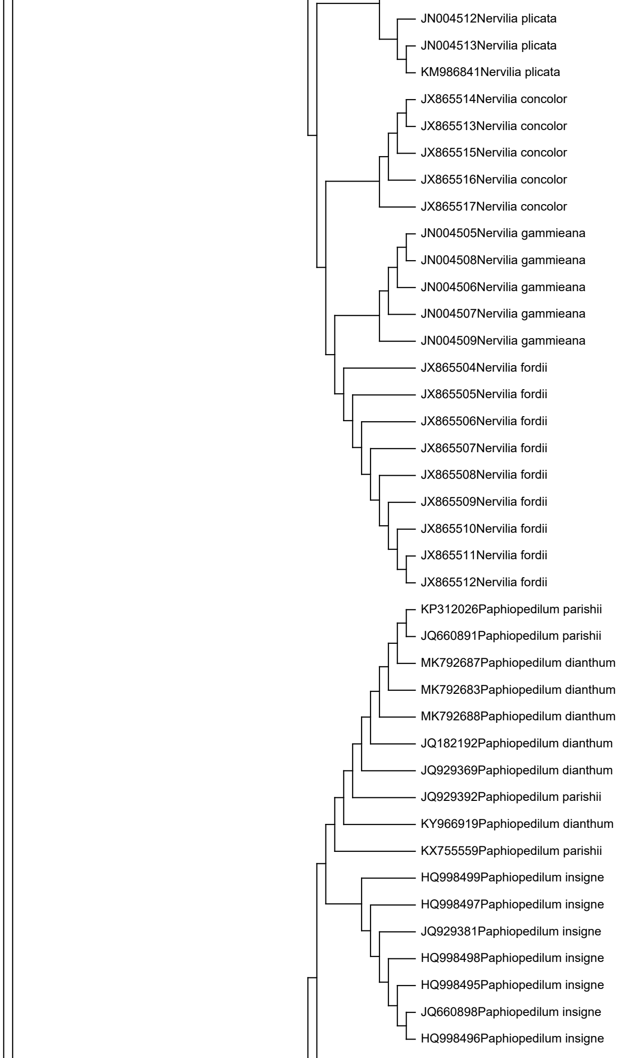
JN004512Nervilia plicata
JN004513Nervilia plicata
KM986841Nervilia plicata
JX865514Nervilia concolor
JX865513Nervilia concolor
JX865515Nervilia concolor
JX865516Nervilia concolor
JX865517Nervilia concolor
JN004505Nervilia gammieana
JN004508Nervilia gammieana
JN004506Nervilia gammieana
JN004507Nervilia gammieana
JN004509Nervilia gammieana
JX865504Nervilia fordii
JX865505Nervilia fordii
JX865506Nervilia fordii
JX865507Nervilia fordii
JX865508Nervilia fordii
JX865509Nervilia fordii
JX865510Nervilia fordii
JX865511Nervilia fordii
JX865512Nervilia fordii
KP312026Paphiopedilum parishii
JQ660891Paphiopedilum parishii
MK792687Paphiopedilum dianthum
MK792683Paphiopedilum dianthum
MK792688Paphiopedilum dianthum
JQ182192Paphiopedilum dianthum
JQ929369Paphiopedilum dianthum
JQ929392Paphiopedilum parishii
KY966919Paphiopedilum dianthum
KX755559Paphiopedilum parishii
HQ998499Paphiopedilum insigne
HQ998497Paphiopedilum insigne
JQ929381Paphiopedilum insigne
HQ998498Paphiopedilum insigne
HQ998495Paphiopedilum insigne
JQ660898Paphiopedilum insigne
HQ998496Paphiopedilum insigne

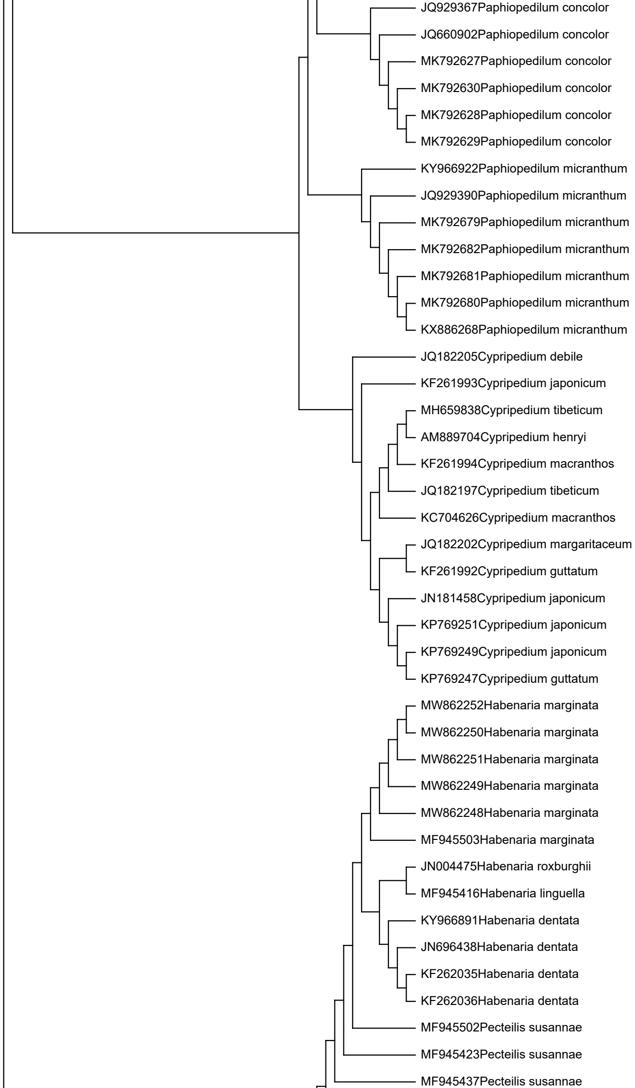
JQ929367Paphiopedilum concolor
JQ660902Paphiopedilum concolor
MK792627Paphiopedilum concolor
MK792630Paphiopedilum concolor
MK792628Paphiopedilum concolor
MK792629Paphiopedilum concolor
KY966922Paphiopedilum micranthum
JQ929390Paphiopedilum micranthum
MK792679Paphiopedilum micranthum
MK792682Paphiopedilum micranthum
MK792681Paphiopedilum micranthum
MK792680Paphiopedilum micranthum
KX886268Paphiopedilum micranthum
JQ182205Cypripedium debile
KF261993Cypripedium japonicum
MH659838Cypripedium tibeticum
AM889704Cypripedium henryi
KF261994Cypripedium macranthos
JQ182197Cypripedium tibeticum
KC704626Cypripedium macranthos
JQ182202Cypripedium margaritaceum
KF261992Cypripedium guttatum
JN181458Cypripedium japonicum
KP769251Cypripedium japonicum
KP769249Cypripedium japonicum
KP769247Cypripedium guttatum
MW862252Habenaria marginata
MW862250Habenaria marginata
MW862251Habenaria marginata
MW862249Habenaria marginata
MW862248Habenaria marginata
MF945503Habenaria marginata
JN004475Habenaria roxburghii
MF945416Habenaria linguella
KY966891Habenaria dentata
JN696438Habenaria dentata
KF262035Habenaria dentata
KF262036Habenaria dentata
MF945502Pecteilis susannae
MF945423Pecteilis susannae
MF945437Pecteilis susannae

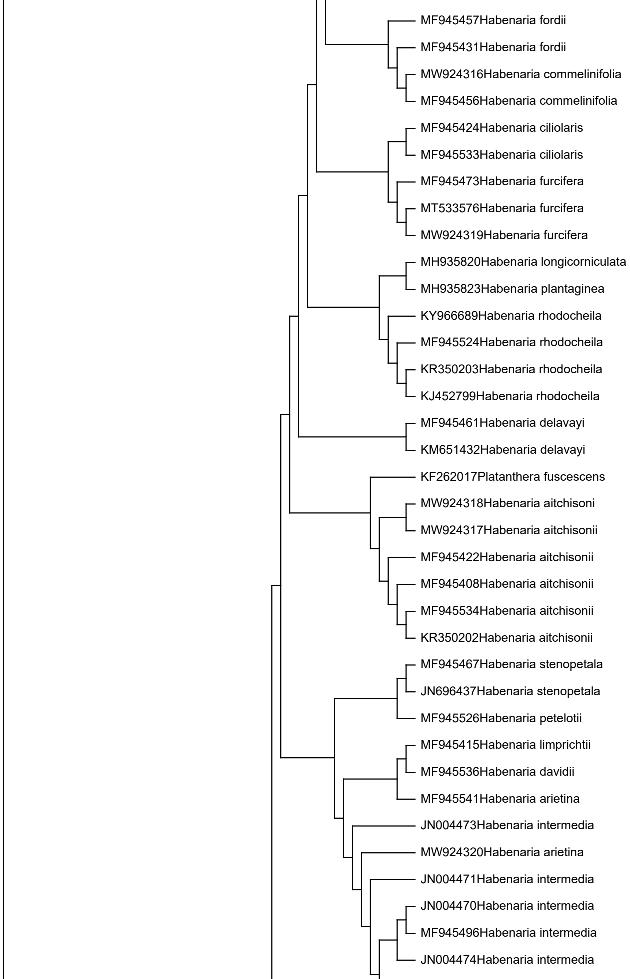
MF945457Habenaria fordii
MF945431Habenaria fordii
MW924316Habenaria commelinifolia
MF945456Habenaria commelinifolia
MF945424Habenaria ciliolaris
MF945533Habenaria ciliolaris
MF945473Habenaria furcifera
MT533576Habenaria furcifera
MW924319Habenaria furcifera
MH935820Habenaria longicorniculata
MH935823Habenaria plantaginea
KY966689Habenaria rhodocheila
MF945524Habenaria rhodocheila
KR350203Habenaria rhodocheila
KJ452799Habenaria rhodocheila
MF945461Habenaria delavayi
KM651432Habenaria delavayi
KF262017Platanthera fuscescens
MW924318Habenaria aitchisoni
MW924317Habenaria aitchisonii
MF945422Habenaria aitchisonii
MF945408Habenaria aitchisonii
MF945534Habenaria aitchisonii
KR350202Habenaria aitchisonii
MF945467Habenaria stenopetala
JN696437Habenaria stenopetala
MF945526Habenaria petelotii
MF945415Habenaria limprichtii
MF945536Habenaria davidii
MF945541Habenaria arietina
JN004473Habenaria intermedia
MW924320Habenaria arietina
JN004471Habenaria intermedia
JN004470Habenaria intermedia
MF945496Habenaria intermedia
JN004474Habenaria intermedia

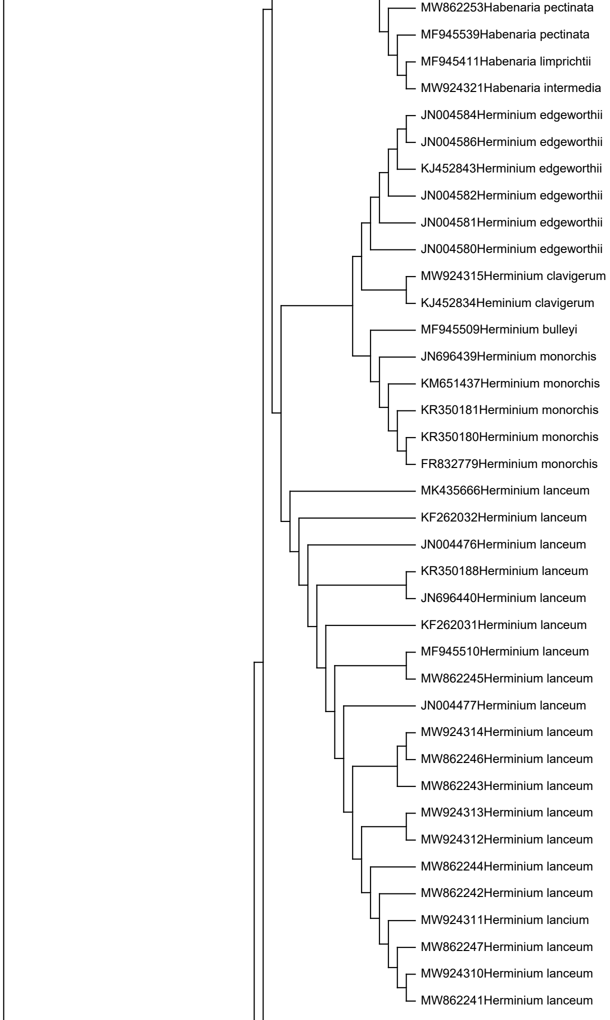
MW862253Habenaria pectinata
MF945539Habenaria pectinata
MF945411Habenaria limprichtii
MW924321Habenaria intermedia
JN004584Herminium edgeworthii
JN004586Herminium edgeworthii
KJ452843Herminium edgeworthii
JN004582Herminium edgeworthii
JN004581Herminium edgeworthii
JN004580Herminium edgeworthii
MW924315Herminium clavigerum
KJ452834Heminium clavigerum
MF945509Herminium bulleyi
JN696439Herminium monorchis
KM651437Herminium monorchis
KR350181Herminium monorchis
KR350180Herminium monorchis
FR832779Herminium monorchis
MK435666Herminium lanceum
KF262032Herminium lanceum
JN004476Herminium lanceum
KR350188Herminium lanceum
JN696440Herminium lanceum
KF262031Herminium lanceum
MF945510Herminium lanceum
MW862245Herminium lanceum
JN004477Herminium lanceum
MW924314Herminium lanceum
MW862246Herminium lanceum
MW862243Herminium lanceum
MW924313Herminium lanceum
MW924312Herminium lanceum
MW862244Herminium lanceum
MW862242Herminium lanceum
MW924311Herminium lancium
MW862247Herminium lanceum
MW924310Herminium lanceum
MW862241Herminium lanceum

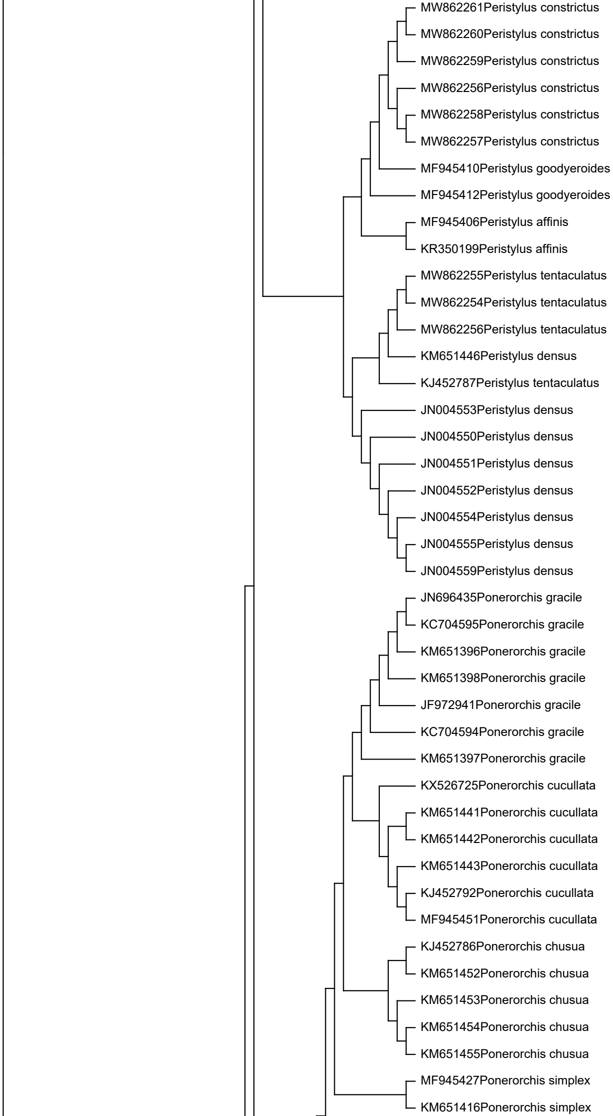
MW862261Peristylus constrictus
MW862260Peristylus constrictus
MW862259Peristylus constrictus
MW862256Peristylus constrictus
MW862258Peristylus constrictus
MW862257Peristylus constrictus
MF945410Peristylus goodyeroides
MF945412Peristylus goodyeroides
MF945406Peristylus affinis
KR350199Peristylus affinis
MW862255Peristylus tentaculatus
MW862254Peristylus tentaculatus
MW862256Peristylus tentaculatus
KM651446Peristylus densus
KJ452787Peristylus tentaculatus
JN004553Peristylus densus
JN004550Peristylus densus
JN004551Peristylus densus
JN004552Peristylus densus
JN004554Peristylus densus
JN004555Peristylus densus
JN004559Peristylus densus
JN696435Ponerorchis gracile
KC704595Ponerorchis gracile
KM651396Ponerorchis gracile
KM651398Ponerorchis gracile
JF972941Ponerorchis gracile
KC704594Ponerorchis gracile
KM651397Ponerorchis gracile
KX526725Ponerorchis cucullata
KM651441Ponerorchis cucullata
KM651442Ponerorchis cucullata
KM651443Ponerorchis cucullata
KJ452792Ponerorchis cucullata
MF945451Ponerorchis cucullata
KJ452786Ponerorchis chusua
KM651452Ponerorchis chusua
KM651453Ponerorchis chusua
KM651454Ponerorchis chusua
KM651455Ponerorchis chusua
MF945427Ponerorchis simplex
KM651416Ponerorchis simplex

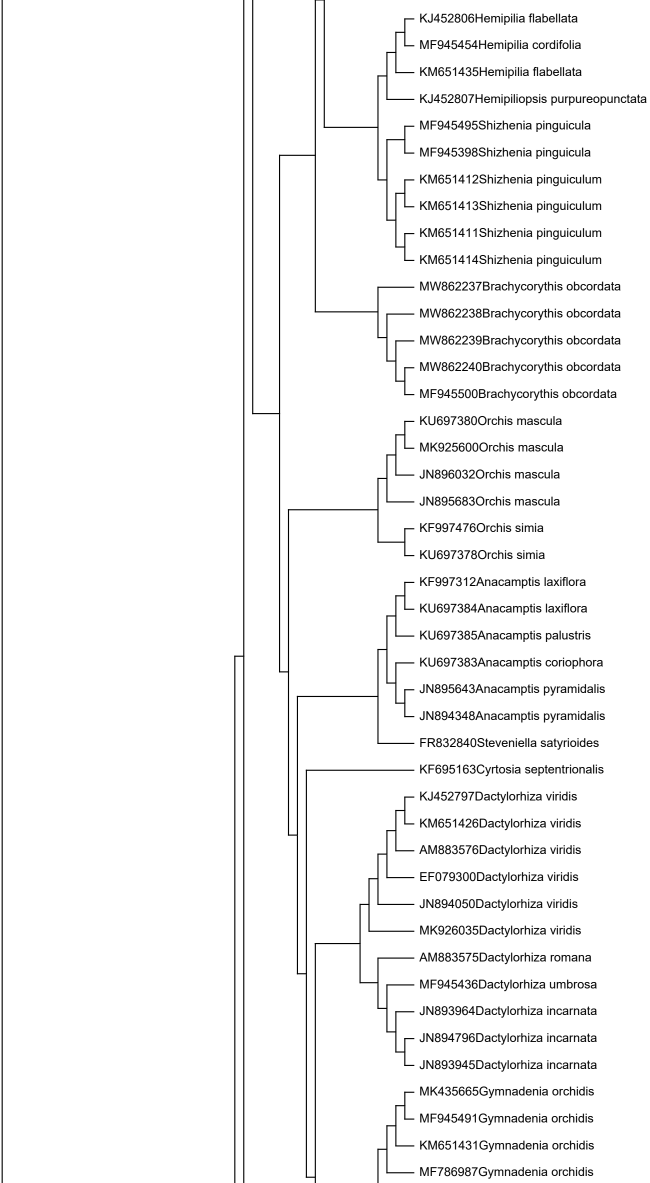
KJ452806Hemipilia flabellata
MF945454Hemipilia cordifolia
KM651435Hemipilia flabellata
KJ452807Hemipiliopsis purpureopunctata
MF945495Shizhenia pinguicula
MF945398Shizhenia pinguicula
KM651412Shizhenia pinguiculum
KM651413Shizhenia pinguiculum
KM651411Shizhenia pinguiculum
KM651414Shizhenia pinguiculum
MW862237Brachycorythis obcordata
MW862238Brachycorythis obcordata
MW862239Brachycorythis obcordata
MW862240Brachycorythis obcordata
MF945500Brachycorythis obcordata
KU697380Orchis mascula
MK925600Orchis mascula
JN896032Orchis mascula
JN895683Orchis mascula
KF997476Orchis simia
KU697378Orchis simia
KF997312Anacamptis laxiflora
KU697384Anacamptis laxiflora
KU697385Anacamptis palustris
KU697383Anacamptis coriophora
JN895643Anacamptis pyramidalis
JN894348Anacamptis pyramidalis
FR832840Steveniella satyrioides
KF695163Cyrtosia septentrionalis
KJ452797Dactylorhiza viridis
KM651426Dactylorhiza viridis
AM883576Dactylorhiza viridis
EF079300Dactylorhiza viridis
JN894050Dactylorhiza viridis
MK926035Dactylorhiza viridis
AM883575Dactylorhiza romana
MF945436Dactylorhiza umbrosa
JN893964Dactylorhiza incarnata
JN894796Dactylorhiza incarnata
JN893945Dactylorhiza incarnata
MK435665Gymnadenia orchidis
MF945491Gymnadenia orchidis
KM651431Gymnadenia orchidis
MF786987Gymnadenia orchidis

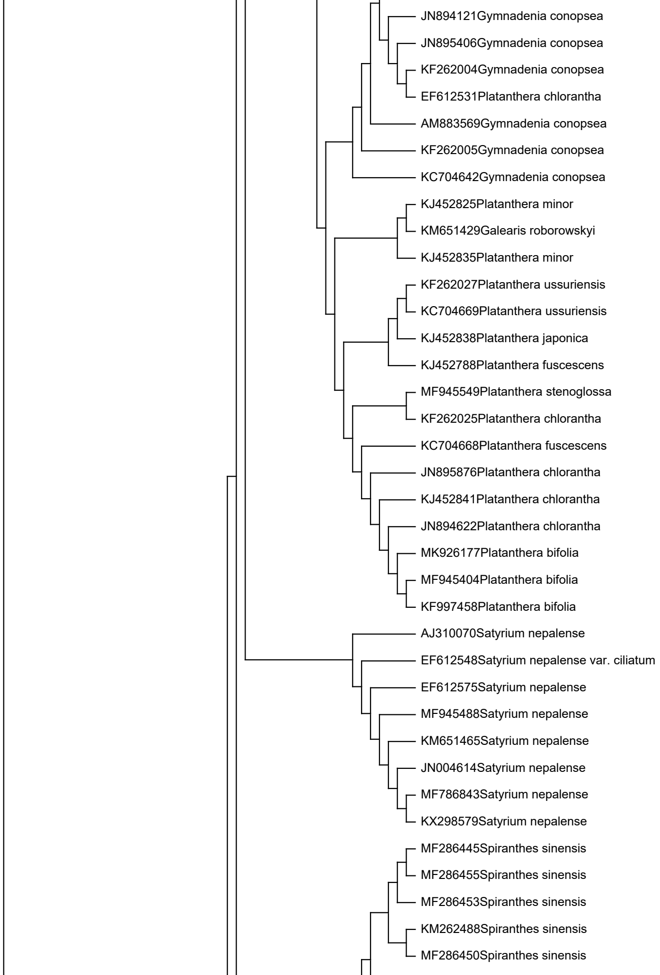
JN894121Gymnadenia conopsea
JN895406Gymnadenia conopsea
KF262004Gymnadenia conopsea
EF612531Platanthera chlorantha
AM883569Gymnadenia conopsea
KF262005Gymnadenia conopsea
KC704642Gymnadenia conopsea
KJ452825Platanthera minor
KM651429Galearis roborowskyi
KJ452835Platanthera minor
KF262027Platanthera ussuriensis
KC704669Platanthera ussuriensis
KJ452838Platanthera japonica
KJ452788Platanthera fuscescens
MF945549Platanthera stenoglossa
KF262025Platanthera chlorantha
KC704668Platanthera fuscescens
JN895876Platanthera chlorantha
KJ452841Platanthera chlorantha
JN894622Platanthera chlorantha
MK926177Platanthera bifolia
MF945404Platanthera bifolia
KF997458Platanthera bifolia
AJ310070Satyrium nepalense
EF612548Satyrium nepalense var. ciliatum
EF612575Satyrium nepalense
MF945488Satyrium nepalense
KM651465Satyrium nepalense
JN004614Satyrium nepalense
MF786843Satyrium nepalense
KX298579Satyrium nepalense
MF286445Spiranthes sinensis
MF286455Spiranthes sinensis
MF286453Spiranthes sinensis
KM262488Spiranthes sinensis
MF286450Spiranthes sinensis

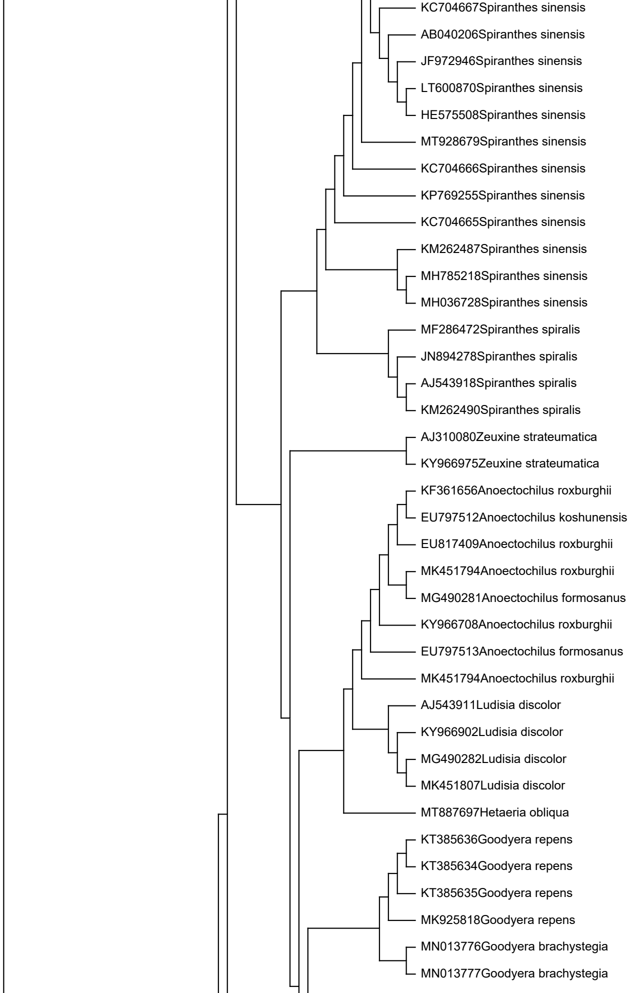
KC704667Spiranthes sinensis
AB040206Spiranthes sinensis
JF972946Spiranthes sinensis
LT600870Spiranthes sinensis
HE575508Spiranthes sinensis
MT928679Spiranthes sinensis
KC704666Spiranthes sinensis
KP769255Spiranthes sinensis
KC704665Spiranthes sinensis
KM262487Spiranthes sinensis
MH785218Spiranthes sinensis
MH036728Spiranthes sinensis
MF286472Spiranthes spiralis
JN894278Spiranthes spiralis
AJ543918Spiranthes spiralis
KM262490Spiranthes spiralis
AJ310080Zeuxine strateumatica
KY966975Zeuxine strateumatica
KF361656Anoectochilus roxburghii
EU797512Anoectochilus koshunensis
EU817409Anoectochilus roxburghii
MK451794Anoectochilus roxburghii
MG490281Anoectochilus formosanus
KY966708Anoectochilus roxburghii
EU797513Anoectochilus formosanus
MK451794Anoectochilus roxburghii
AJ543911Ludisia discolor
KY966902Ludisia discolor
MG490282Ludisia discolor
MK451807Ludisia discolor
MT887697Hetaeria obliqua
KT385636Goodyera repens
KT385634Goodyera repens
KT385635Goodyera repens
MK925818Goodyera repens
MN013776Goodyera brachystegia
MN013777Goodyera brachystegia

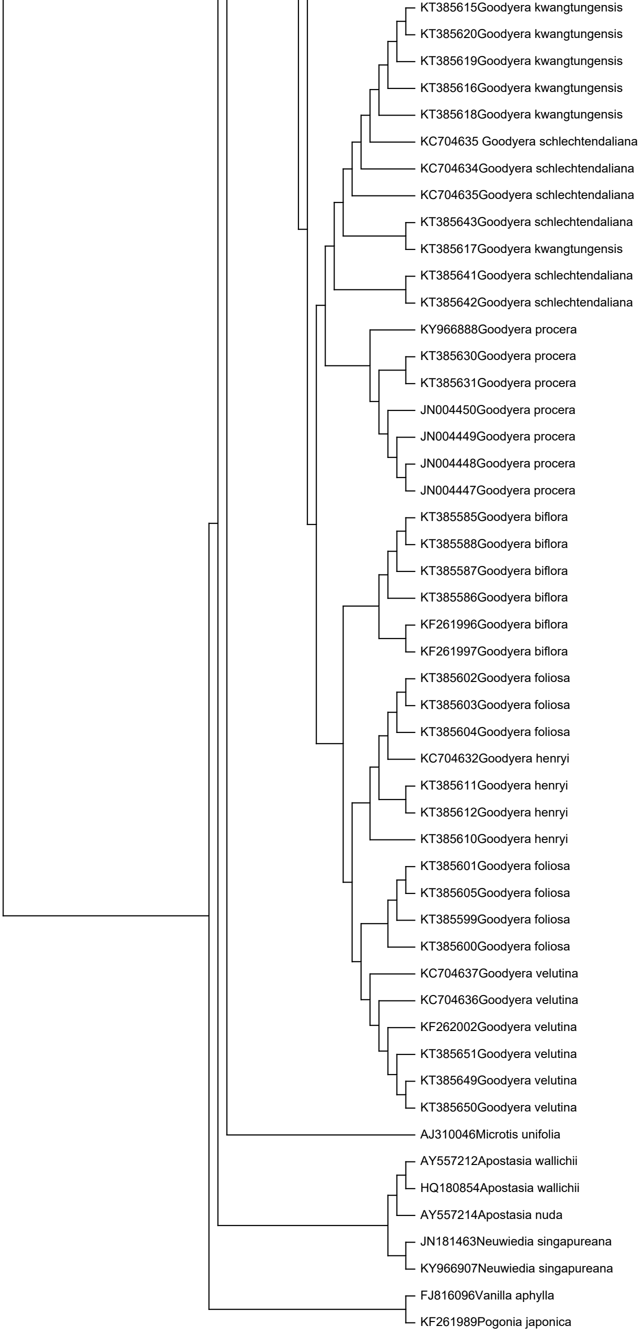
KT385615Goodyera kwangtungensis
KT385620Goodyera kwangtungensis
KT385619Goodyera kwangtungensis
KT385616Goodyera kwangtungensis
KT385618Goodyera kwangtungensis
KC704635 Goodyera schlechtendaliana
KC704634Goodyera schlechtendaliana
KC704635Goodyera schlechtendaliana
KT385643Goodyera schlechtendaliana
KT385617Goodyera kwangtungensis
KT385641Goodyera schlechtendaliana
KT385642Goodyera schlechtendaliana
KY966888Goodyera procera
KT385630Goodyera procera
KT385631Goodyera procera
JN004450Goodyera procera
JN004449Goodyera procera
JN004448Goodyera procera
JN004447Goodyera procera
KT385585Goodyera biflora
KT385588Goodyera biflora
KT385587Goodyera biflora
KT385586Goodyera biflora
KF261996Goodyera biflora
KF261997Goodyera biflora
KT385602Goodyera foliosa
KT385603Goodyera foliosa
KT385604Goodyera foliosa
KC704632Goodyera henryi
KT385611Goodyera henryi
KT385612Goodyera henryi
KT385610Goodyera henryi
KT385601Goodyera foliosa
KT385605Goodyera foliosa
KT385599Goodyera foliosa
KT385600Goodyera foliosa
KC704637Goodyera velutina
KC704636Goodyera velutina
KF262002Goodyera velutina
KT385651Goodyera velutina
KT385649Goodyera velutina
KT385650Goodyera velutina
AJ310046Microtis unifolia
AY557212Apostasia wallichii
HQ180854Apostasia wallichii
AY557214Apostasia nuda
JN181463Neuwiedia singapureana
KY966907Neuwiedia singapureana
FJ816096Vanilla aphylla
KF261989Pogonia japonica
