# Supplementary material for: DNA barcoding of medicinal orchids in Asia
Source: Sci Rep. 2021 Dec 8;11:23651. doi: 10.1038/s41598-021-03025-0 (PMC8654824; doi:10.1038/s41598-021-03025-0)
Supplement: Supplementary file 5 — Supplementary Figure S5. [file 41598_2021_3025_MOESM5_ESM.pdf]

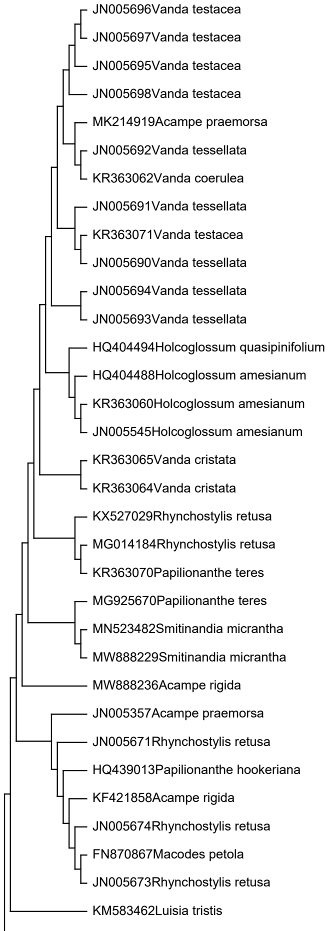

JN005696Vanda testacea
JN005697Vanda testacea
JN005695Vanda testacea
JN005698Vanda testacea
MK214919Acampe praemorsa
JN005692Vanda tessellata
KR363062Vanda coerulea
JN005691Vanda tessellata
KR363071Vanda testacea
JN005690Vanda tessellata
JN005694Vanda tessellata
JN005693Vanda tessellata
HQ404494Holcoglossum quasipinifolium
HQ404488Holcoglossum amesianum
KR363060Holcoglossum amesianum
JN005545Holcoglossum amesianum
KR363065Vanda cristata
KR363064Vanda cristata
KX527029Rhynchostylis retusa
MG014184Rhynchostylis retusa
KR363070Papilionanthe teres
MG925670Papilionanthe teres
MN523482Smitinandia micrantha
MW888229Smitinandia micrantha
MW888236Acampe rigida
JN005357Acampe praemorsa
JN005671Rhynchostylis retusa
HQ439013Papilionanthe hookeriana
KF421858Acampe rigida
JN005674Rhynchostylis retusa
FN870867Macodes petola
JN005673Rhynchostylis retusa
KM583462Luisia tristis

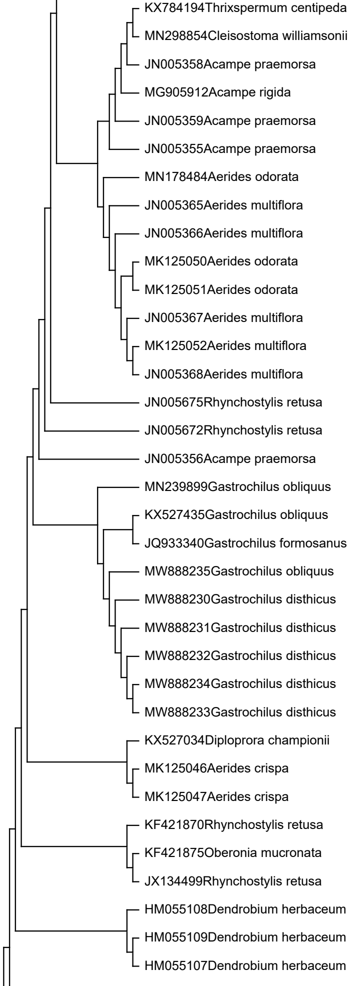
KX784194Thrixspermum centipeda
MN298854Cleisostoma williamsonii
JN005358Acampe praemorsa
MG905912Acampe rigida
JN005359Acampe praemorsa
JN005355Acampe praemorsa
MN178484Aerides odorata
JN005365Aerides multiflora
JN005366Aerides multiflora
MK125050Aerides odorata
MK125051Aerides odorata
JN005367Aerides multiflora
MK125052Aerides multiflora
JN005368Aerides multiflora
JN005675Rhynchostylis retusa
JN005672Rhynchostylis retusa
JN005356Acampe praemorsa
MN239899Gastrochilus obliquus
KX527435Gastrochilus obliquus
JQ933340Gastrochilus formosanus
MW888235Gastrochilus obliquus
MW888230Gastrochilus disthicus
MW888231Gastrochilus disthicus
MW888232Gastrochilus disthicus
MW888234Gastrochilus disthicus
MW888233Gastrochilus disthicus
KX527034Diploprora championii
MK125046Aerides crispa
MK125047Aerides crispa
KF421870Rhynchostylis retusa
KF421875Oberonia mucronata
JX134499Rhynchostylis retusa
HM055108Dendrobium herbaceum
HM055109Dendrobium herbaceum
HM055107Dendrobium herbaceum

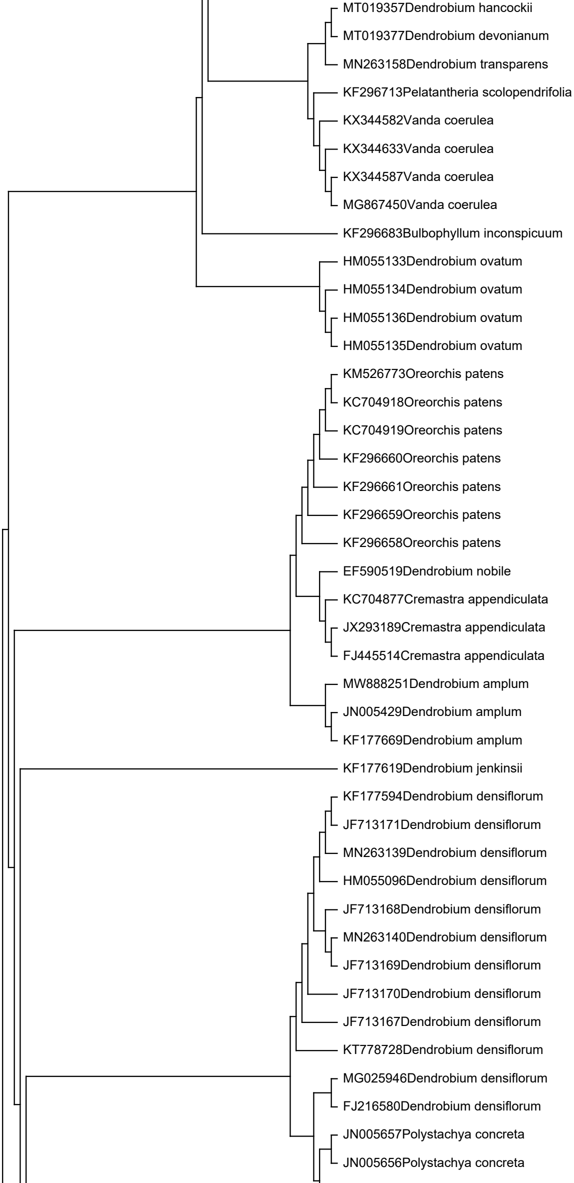
MT019357Dendrobium hancockii
MT019377Dendrobium devonianum
MN263158Dendrobium transparens
KF296713Pelatantheria scolopendrifolia
KX344582Vanda coerulea
KX344633Vanda coerulea
KX344587Vanda coerulea
MG867450Vanda coerulea
KF296683Bulbophyllum inconspicuum
HM055133Dendrobium ovatum
HM055134Dendrobium ovatum
HM055136Dendrobium ovatum
HM055135Dendrobium ovatum
KM526773Oreorchis patens
KC704918Oreorchis patens
KC704919Oreorchis patens
KF296660Oreorchis patens
KF296661Oreorchis patens
KF296659Oreorchis patens
KF296658Oreorchis patens
EF590519Dendrobium nobile
KC704877Cremastra appendiculata
JX293189Cremastra appendiculata
FJ445514Cremastra appendiculata
MW888251Dendrobium amplum
JN005429Dendrobium amplum
KF177669Dendrobium amplum
KF177619Dendrobium jenkinsii
KF177594Dendrobium densiflorum
JF713171Dendrobium densiflorum
MN263139Dendrobium densiflorum
HM055096Dendrobium densiflorum
JF713168Dendrobium densiflorum
MN263140Dendrobium densiflorum
JF713169Dendrobium densiflorum
JF713170Dendrobium densiflorum
JF713167Dendrobium densiflorum
KT778728Dendrobium densiflorum
MG025946Dendrobium densiflorum
FJ216580Dendrobium densiflorum
JN005657Polystachya concreta
JN005656Polystachya concreta

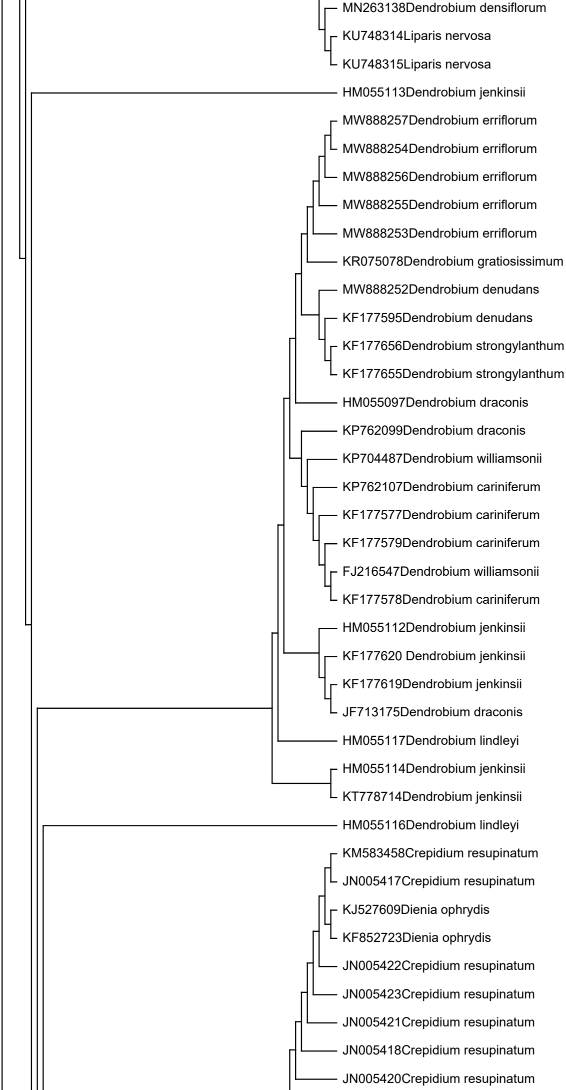
MN263138Dendrobium densiflorum
KU748314Liparis nervosa
KU748315Liparis nervosa
HM055113Dendrobium jenkinsii
MW888257Dendrobium erriflorum
MW888254Dendrobium erriflorum
MW888256Dendrobium erriflorum
MW888255Dendrobium erriflorum
MW888253Dendrobium erriflorum
KR075078Dendrobium gratiosissimum
MW888252Dendrobium denudans
KF177595Dendrobium denudans
KF177656Dendrobium strongylanthum
KF177655Dendrobium strongylanthum
HM055097Dendrobium draconis
KP762099Dendrobium draconis
KP704487Dendrobium williamsonii
KP762107Dendrobium cariniferum
KF177577Dendrobium cariniferum
KF177579Dendrobium cariniferum
FJ216547Dendrobium williamsonii
KF177578Dendrobium cariniferum
HM055112Dendrobium jenkinsii
KF177620 Dendrobium jenkinsii
KF177619Dendrobium jenkinsii
JF713175Dendrobium draconis
HM055117Dendrobium lindleyi
HM055114Dendrobium jenkinsii
KT778714Dendrobium jenkinsii
HM055116Dendrobium lindleyi
KM583458Crepidium resupinatum
JN005417Crepidium resupinatum
KJ527609Dienia ophrydis
KF852723Dienia ophrydis
JN005422Crepidium resupinatum
JN005423Crepidium resupinatum
JN005421Crepidium resupinatum
JN005418Crepidium resupinatum
JN005420Crepidium resupinatum

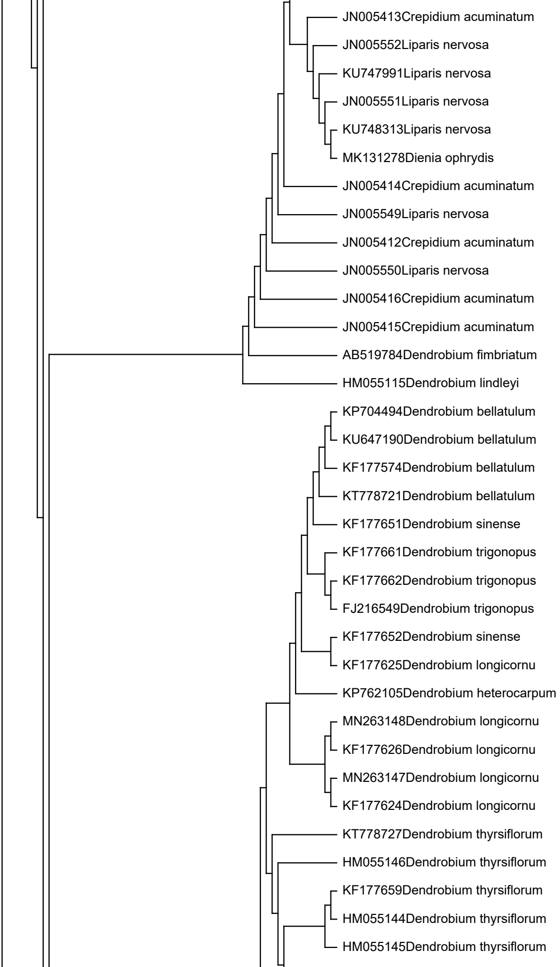

JN005413Crepidium acuminatum
JN005552Liparis nervosa
KU747991Liparis nervosa
JN005551Liparis nervosa
KU748313Liparis nervosa
MK131278Dienia ophrydis
JN005414Crepidium acuminatum
JN005549Liparis nervosa
JN005412Crepidium acuminatum
JN005550Liparis nervosa
JN005416Crepidium acuminatum
JN005415Crepidium acuminatum
AB519784Dendrobium fimbriatum
HM055115Dendrobium lindleyi
KP704494Dendrobium bellatulum
KU647190Dendrobium bellatulum
KF177574Dendrobium bellatulum
KT778721Dendrobium bellatulum
KF177651Dendrobium sinense
KF177661Dendrobium trigonopus
KF177662Dendrobium trigonopus
FJ216549Dendrobium trigonopus
KF177652Dendrobium sinense
KF177625Dendrobium longicornu
KP762105Dendrobium heterocarpum
MN263148Dendrobium longicornu
KF177626Dendrobium longicornu
MN263147Dendrobium longicornu
KF177624Dendrobium longicornu
KT778727Dendrobium thyrsiflorum
HM055146Dendrobium thyrsiflorum
KF177659Dendrobium thyrsiflorum
HM055144Dendrobium thyrsiflorum
HM055145Dendrobium thyrsiflorum

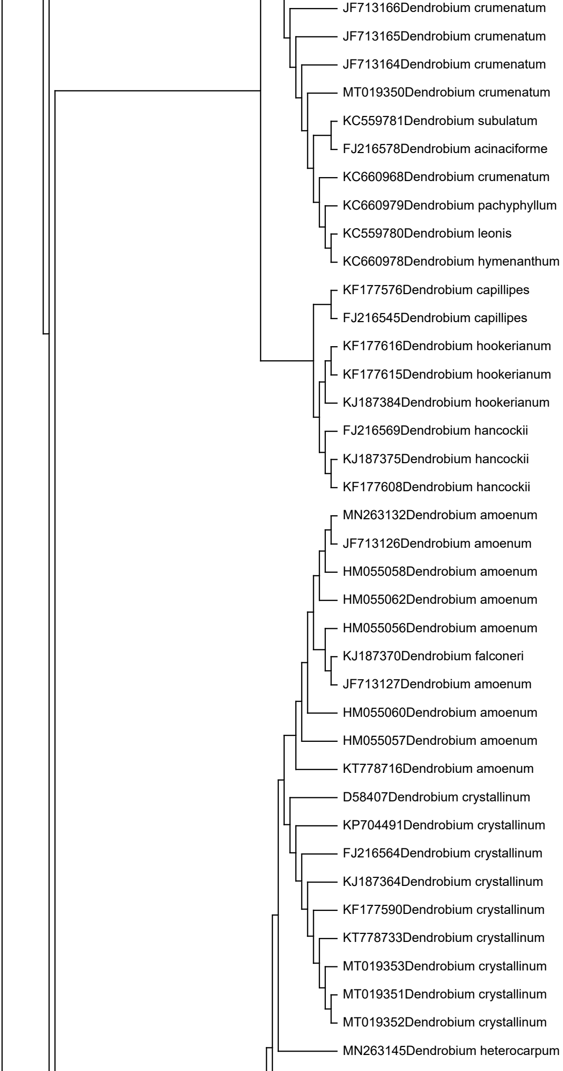
JF713166Dendrobium crumenatum
JF713165Dendrobium crumenatum
JF713164Dendrobium crumenatum
MT019350Dendrobium crumenatum
KC559781Dendrobium subulatum
FJ216578Dendrobium acinaciforme
KC660968Dendrobium crumenatum
KC660979Dendrobium pachyphyllum
KC559780Dendrobium leonis
KC660978Dendrobium hymenanthum
KF177576Dendrobium capillipes
FJ216545Dendrobium capillipes
KF177616Dendrobium hookerianum
KF177615Dendrobium hookerianum
KJ187384Dendrobium hookerianum
FJ216569Dendrobium hancockii
KJ187375Dendrobium hancockii
KF177608Dendrobium hancockii
MN263132Dendrobium amoenum
JF713126Dendrobium amoenum
HM055058Dendrobium amoenum
HM055062Dendrobium amoenum
HM055056Dendrobium amoenum
KJ187370Dendrobium falconeri
JF713127Dendrobium amoenum
HM055060Dendrobium amoenum
HM055057Dendrobium amoenum
KT778716Dendrobium amoenum
D58407Dendrobium crystallinum
KP704491Dendrobium crystallinum
FJ216564Dendrobium crystallinum
KJ187364Dendrobium crystallinum
KF177590Dendrobium crystallinum
KT778733Dendrobium crystallinum
MT019353Dendrobium crystallinum
MT019351Dendrobium crystallinum
MT019352Dendrobium crystallinum
MN263145Dendrobium heterocarpum

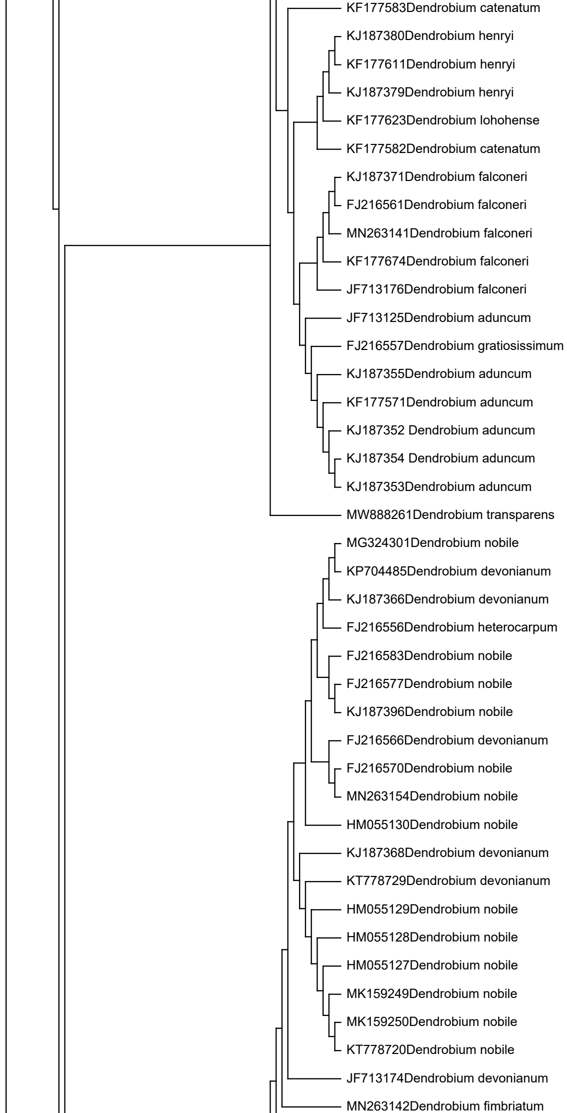
KF177583Dendrobium catenatum
KJ187380Dendrobium henryi
KF177611Dendrobium henryi
KJ187379Dendrobium henryi
KF177623Dendrobium lohohense
KF177582Dendrobium catenatum
KJ187371Dendrobium falconeri
FJ216561Dendrobium falconeri
MN263141Dendrobium falconeri
KF177674Dendrobium falconeri
JF713176Dendrobium falconeri
JF713125Dendrobium aduncum
FJ216557Dendrobium gratiosissimum
KJ187355Dendrobium aduncum
KF177571Dendrobium aduncum
KJ187352 Dendrobium aduncum
KJ187354 Dendrobium aduncum
KJ187353Dendrobium aduncum
MW888261Dendrobium transparens
MG324301Dendrobium nobile
KP704485Dendrobium devonianum
KJ187366Dendrobium devonianum
FJ216556Dendrobium heterocarpum
FJ216583Dendrobium nobile
FJ216577Dendrobium nobile
KJ187396Dendrobium nobile
FJ216566Dendrobium devonianum
FJ216570Dendrobium nobile
MN263154Dendrobium nobile
HM055130Dendrobium nobile
KJ187368Dendrobium devonianum
KT778729Dendrobium devonianum
HM055129Dendrobium nobile
HM055128Dendrobium nobile
HM055127Dendrobium nobile
MK159249Dendrobium nobile
MK159250Dendrobium nobile
KT778720Dendrobium nobile
JF713174Dendrobium devonianum
MN263142Dendrobium fimbriatum

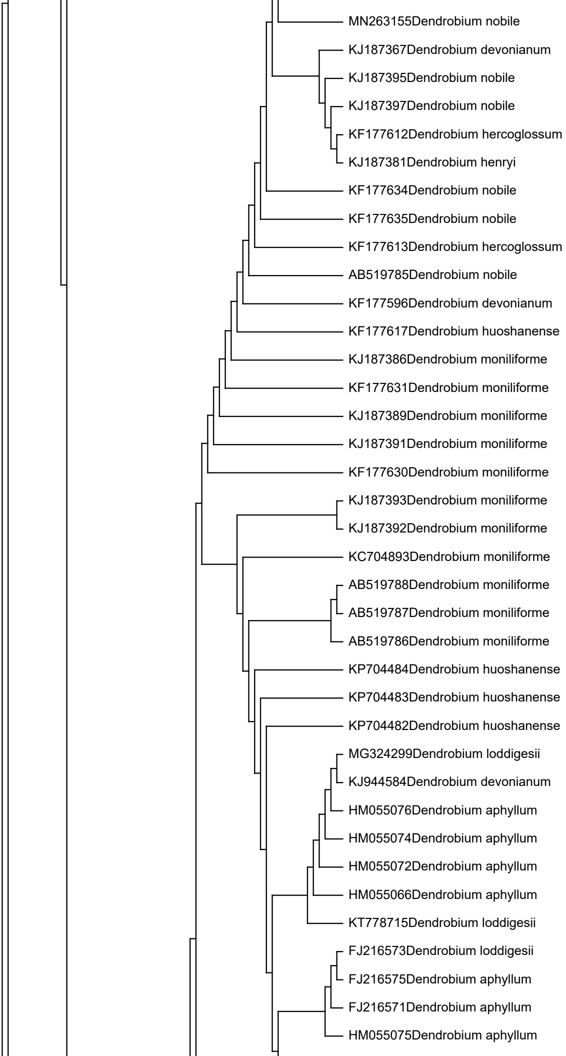
MN263155Dendrobium nobile
KJ187367Dendrobium devonianum
KJ187395Dendrobium nobile
KJ187397Dendrobium nobile
KF177612Dendrobium hercoglossum
KJ187381Dendrobium henryi
KF177634Dendrobium nobile
KF177635Dendrobium nobile
KF177613Dendrobium hercoglossum
AB519785Dendrobium nobile
KF177596Dendrobium devonianum
KF177617Dendrobium huoshanense
KJ187386Dendrobium moniliforme
KF177631Dendrobium moniliforme
KJ187389Dendrobium moniliforme
KJ187391Dendrobium moniliforme
KF177630Dendrobium moniliforme
KJ187393Dendrobium moniliforme
KJ187392Dendrobium moniliforme
KC704893Dendrobium moniliforme
AB519788Dendrobium moniliforme
AB519787Dendrobium moniliforme
AB519786Dendrobium moniliforme
KP704484Dendrobium huoshanense
KP704483Dendrobium huoshanense
KP704482Dendrobium huoshanense
MG324299Dendrobium loddigesii
KJ944584Dendrobium devonianum
HM055076Dendrobium aphyllum
HM055074Dendrobium aphyllum
HM055072Dendrobium aphyllum
HM055066Dendrobium aphyllum
KT778715Dendrobium loddigesii
FJ216573Dendrobium loddigesii
FJ216575Dendrobium aphyllum
FJ216571Dendrobium aphyllum
HM055075Dendrobium aphyllum

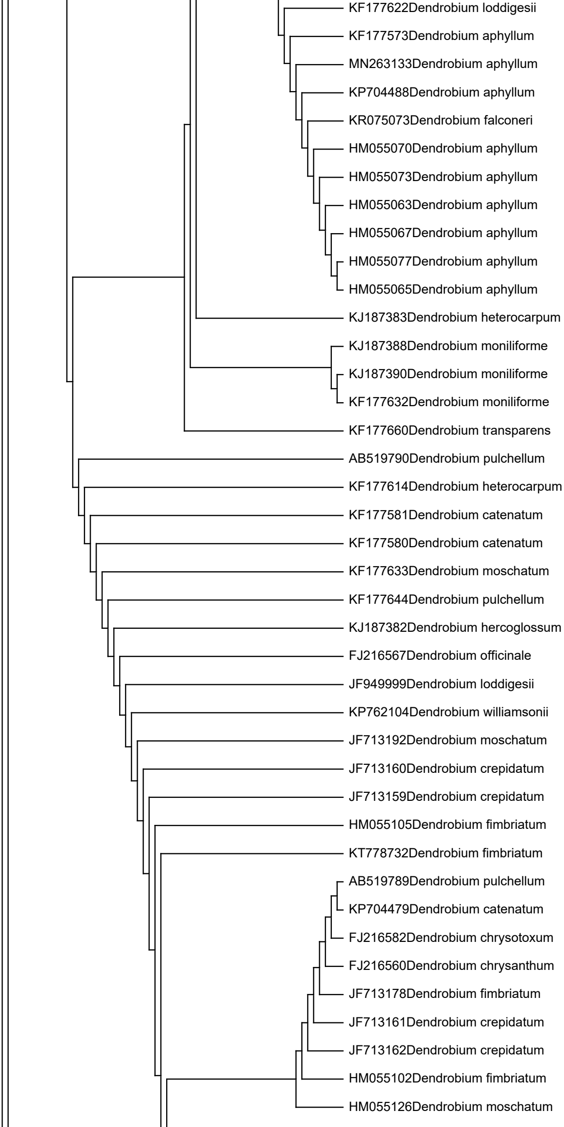
KF177622Dendrobium loddigesii
KF177573Dendrobium aphyllum
MN263133Dendrobium aphyllum
KP704488Dendrobium aphyllum
KR075073Dendrobium falconeri
HM055070Dendrobium aphyllum
HM055073Dendrobium aphyllum
HM055063Dendrobium aphyllum
HM055067Dendrobium aphyllum
HM055077Dendrobium aphyllum
HM055065Dendrobium aphyllum
KJ187383Dendrobium heterocarpum
KJ187388Dendrobium moniliforme
KJ187390Dendrobium moniliforme
KF177632Dendrobium moniliforme
KF177660Dendrobium transparens
AB519790Dendrobium pulchellum
KF177614Dendrobium heterocarpum
KF177581Dendrobium catenatum
KF177580Dendrobium catenatum
KF177633Dendrobium moschatum
KF177644Dendrobium pulchellum
KJ187382Dendrobium hercoglossum
FJ216567Dendrobium officinale
JF949999Dendrobium loddigesii
KP762104Dendrobium williamsonii
JF713192Dendrobium moschatum
JF713160Dendrobium crepidatum
JF713159Dendrobium crepidatum
HM055105Dendrobium fimbriatum
KT778732Dendrobium fimbriatum
AB519789Dendrobium pulchellum
KP704479Dendrobium catenatum
FJ216582Dendrobium chrysotoxum
FJ216560Dendrobium chrysanthum
JF713178Dendrobium fimbriatum
JF713161Dendrobium crepidatum
JF713162Dendrobium crepidatum
HM055102Dendrobium fimbriatum
HM055126Dendrobium moschatum

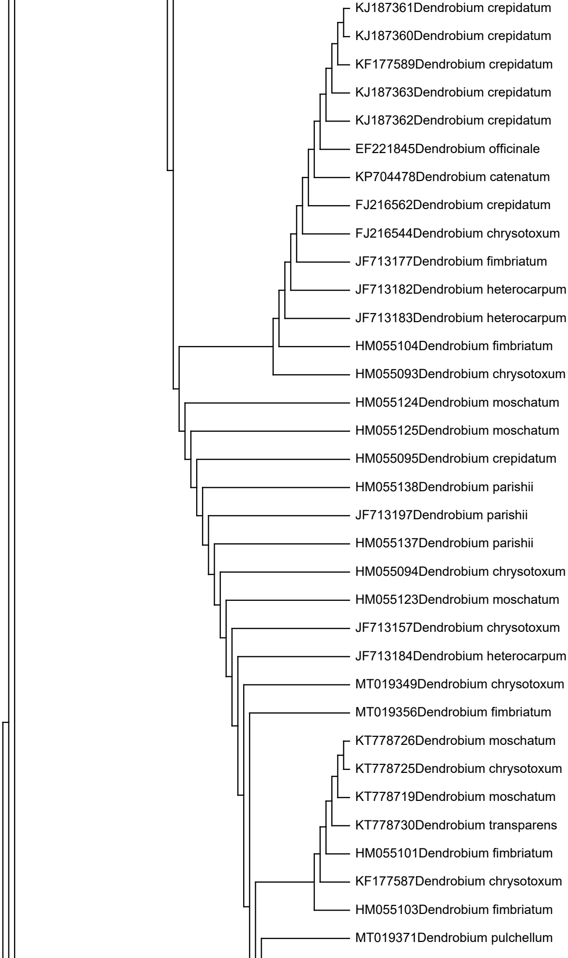
KJ187361Dendrobium crepidatum
KJ187360Dendrobium crepidatum
KF177589Dendrobium crepidatum
KJ187363Dendrobium crepidatum
KJ187362Dendrobium crepidatum
EF221845Dendrobium officinale
KP704478Dendrobium catenatum
FJ216562Dendrobium crepidatum
FJ216544Dendrobium chrysotoxum
JF713177Dendrobium fimbriatum
JF713182Dendrobium heterocarpum
JF713183Dendrobium heterocarpum
HM055104Dendrobium fimbriatum
HM055093Dendrobium chrysotoxum
HM055124Dendrobium moschatum
HM055125Dendrobium moschatum
HM055095Dendrobium crepidatum
HM055138Dendrobium parishii
JF713197Dendrobium parishii
HM055137Dendrobium parishii
HM055094Dendrobium chrysotoxum
HM055123Dendrobium moschatum
JF713157Dendrobium chrysotoxum
JF713184Dendrobium heterocarpum
MT019349Dendrobium chrysotoxum
MT019356Dendrobium fimbriatum
KT778726Dendrobium moschatum
KT778725Dendrobium chrysotoxum
KT778719Dendrobium moschatum
KT778730Dendrobium transparens
HM055101Dendrobium fimbriatum
KF177587Dendrobium chrysotoxum
HM055103Dendrobium fimbriatum
MT019371Dendrobium pulchellum

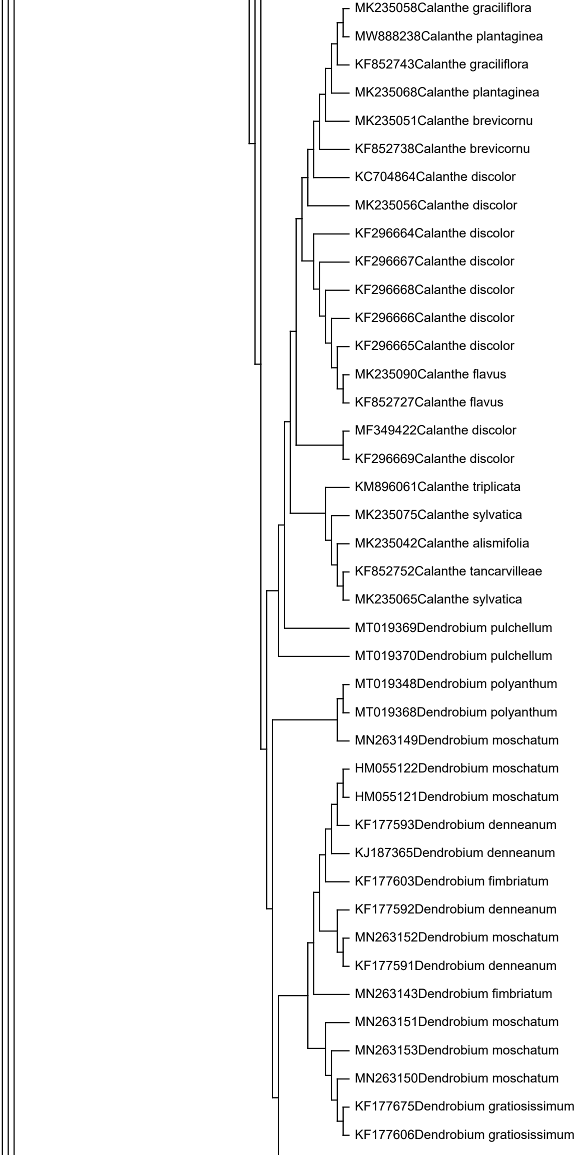
MK235058Calanthe graciliflora
MW888238Calanthe plantaginea
KF852743Calanthe graciliflora
MK235068Calanthe plantaginea
MK235051Calanthe brevicornu
KF852738Calanthe brevicornu
KC704864Calanthe discolor
MK235056Calanthe discolor
KF296664Calanthe discolor
KF296667Calanthe discolor
KF296668Calanthe discolor
KF296666Calanthe discolor
KF296665Calanthe discolor
MK235090Calanthe flavus
KF852727Calanthe flavus
MF349422Calanthe discolor
KF296669Calanthe discolor
KM896061Calanthe triplicata
MK235075Calanthe sylvatica
MK235042Calanthe alismifolia
KF852752Calanthe tancarvilleae
MK235065Calanthe sylvatica
MT019369Dendrobium pulchellum
MT019370Dendrobium pulchellum
MT019348Dendrobium polyanthum
MT019368Dendrobium polyanthum
MN263149Dendrobium moschatum
HM055122Dendrobium moschatum
HM055121Dendrobium moschatum
KF177593Dendrobium denneanum
KJ187365Dendrobium denneanum
KF177603Dendrobium fimbriatum
KF177592Dendrobium denneanum
MN263152Dendrobium moschatum
KF177591Dendrobium denneanum
MN263143Dendrobium fimbriatum
MN263151Dendrobium moschatum
MN263153Dendrobium moschatum
MN263150Dendrobium moschatum
KF177675Dendrobium gratiosissimum
KF177606Dendrobium gratiosissimum

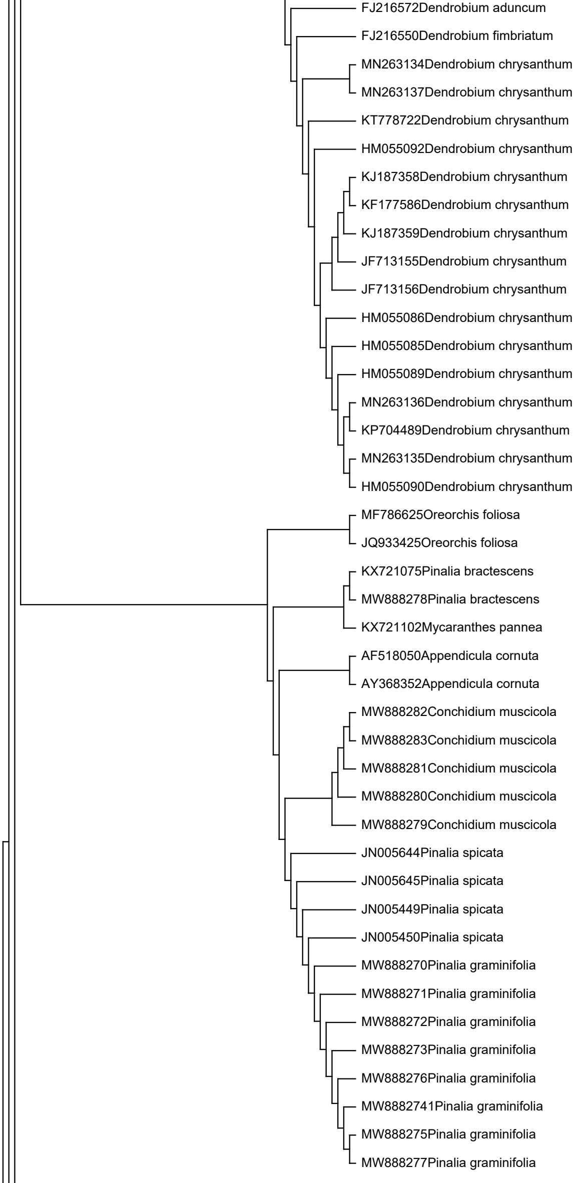

FJ216572Dendrobium aduncum
FJ216550Dendrobium fimbriatum
MN263134Dendrobium chrysanthum
MN263137Dendrobium chrysanthum
KT778722Dendrobium chrysanthum
HM055092Dendrobium chrysanthum
KJ187358Dendrobium chrysanthum
KF177586Dendrobium chrysanthum
KJ187359Dendrobium chrysanthum
JF713155Dendrobium chrysanthum
JF713156Dendrobium chrysanthum
HM055086Dendrobium chrysanthum
HM055085Dendrobium chrysanthum
HM055089Dendrobium chrysanthum
MN263136Dendrobium chrysanthum
KP704489Dendrobium chrysanthum
MN263135Dendrobium chrysanthum
HM055090Dendrobium chrysanthum
MF786625Oreorchis foliosa
JQ933425Oreorchis foliosa
KX721075Pinalia bractescens
MW888278Pinalia bractescens
KX721102Mycaranthes pannea
AF518050Appendicula cornuta
AY368352Appendicula cornuta
MW888282Conchidium muscicola
MW888283Conchidium muscicola
MW888281Conchidium muscicola
MW888280Conchidium muscicola
MW888279Conchidium muscicola
JN005644Pinalia spicata
JN005645Pinalia spicata
JN005449Pinalia spicata
JN005450Pinalia spicata
MW888270Pinalia graminifolia
MW888271Pinalia graminifolia
MW888272Pinalia graminifolia
MW888273Pinalia graminifolia
MW888276Pinalia graminifolia
MW8882741Pinalia graminifolia
MW888275Pinalia graminifolia
MW888277Pinalia graminifolia

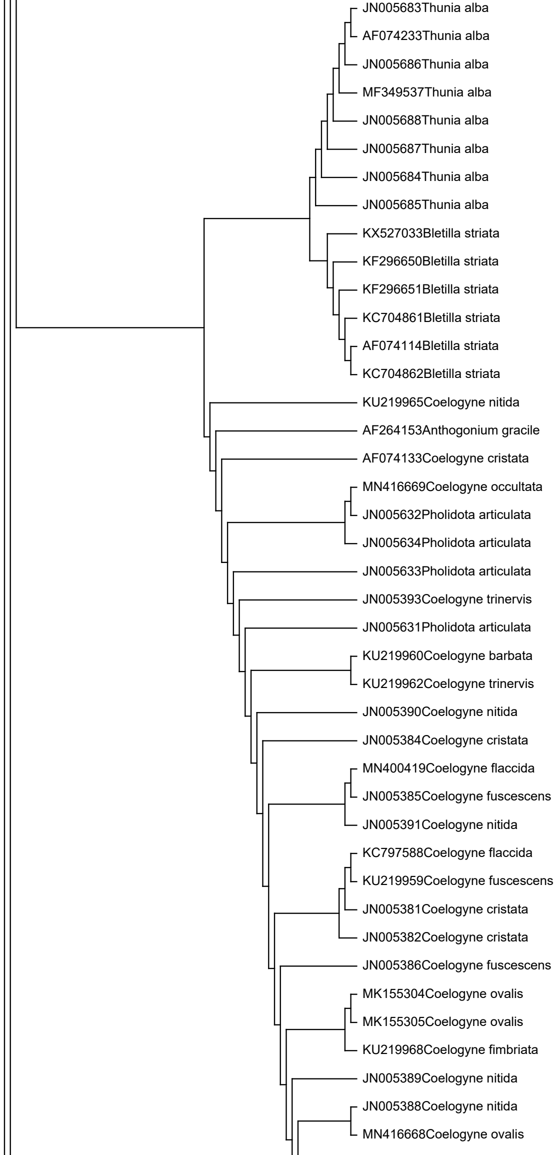
JN005683Thunia alba
AF074233Thunia alba
JN005686Thunia alba
MF349537Thunia alba
JN005688Thunia alba
JN005687Thunia alba
JN005684Thunia alba
JN005685Thunia alba
KX527033Bletilla striata
KF296650Bletilla striata
KF296651Bletilla striata
KC704861Bletilla striata
AF074114Bletilla striata
KC704862Bletilla striata
KU219965Coelogyne nitida
AF264153Anthogonium gracile
AF074133Coelogyne cristata
MN416669Coelogyne occultata
JN005632Pholidota articulata
JN005634Pholidota articulata
JN005633Pholidota articulata
JN005393Coelogyne trinervis
JN005631Pholidota articulata
KU219960Coelogyne barbata
KU219962Coelogyne trinervis
JN005390Coelogyne nitida
JN005384Coelogyne cristata
MN400419Coelogyne flaccida
JN005385Coelogyne fuscescens
JN005391Coelogyne nitida
KC797588Coelogyne flaccida
KU219959Coelogyne fuscescens
JN005381Coelogyne cristata
JN005382Coelogyne cristata
JN005386Coelogyne fuscescens
MK155304Coelogyne ovalis
MK155305Coelogyne ovalis
KU219968Coelogyne fimbriata
JN005389Coelogyne nitida
JN005388Coelogyne nitida
MN416668Coelogyne ovalis

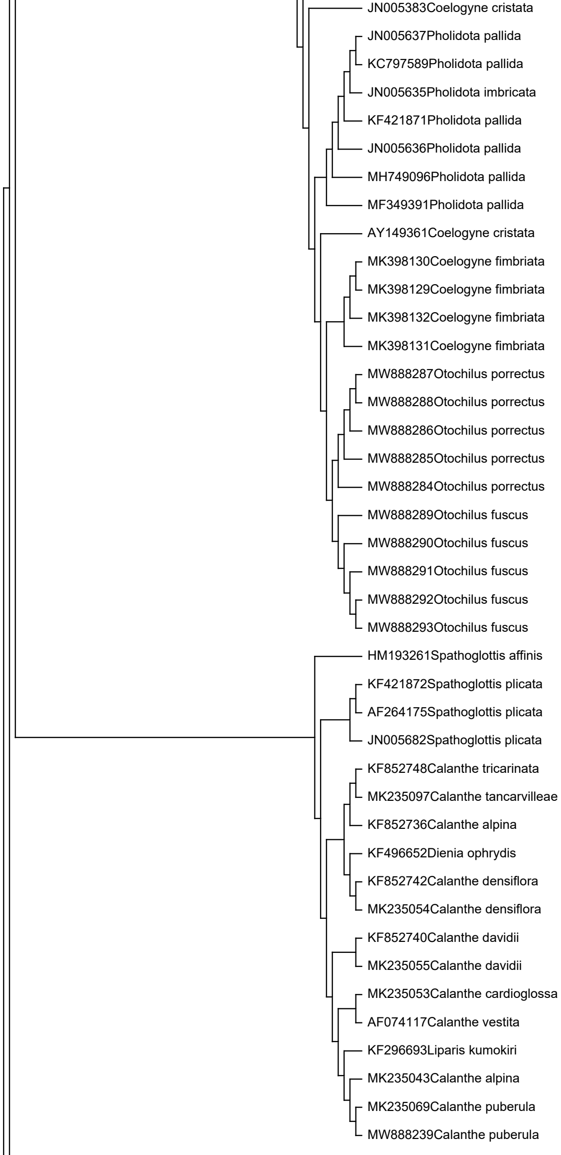

JN005383Coelogyne cristata
JN005637Pholidota pallida
KC797589Pholidota pallida
JN005635Pholidota imbricata
KF421871Pholidota pallida
JN005636Pholidota pallida
MH749096Pholidota pallida
MF349391Pholidota pallida
AY149361Coelogyne cristata
MK398130Coelogyne fimbriata
MK398129Coelogyne fimbriata
MK398132Coelogyne fimbriata
MK398131Coelogyne fimbriata
MW888287Otochilus porrectus
MW888288Otochilus porrectus
MW888286Otochilus porrectus
MW888285Otochilus porrectus
MW888284Otochilus porrectus
MW888289Otochilus fuscus
MW888290Otochilus fuscus
MW888291Otochilus fuscus
MW888292Otochilus fuscus
MW888293Otochilus fuscus
HM193261Spathoglottis affinis
KF421872Spathoglottis plicata
AF264175Spathoglottis plicata
JN005682Spathoglottis plicata
KF852748Calanthe tricarinata
MK235097Calanthe tancarvilleae
KF852736Calanthe alpina
KF496652Dienia ophrydis
KF852742Calanthe densiflora
MK235054Calanthe densiflora
KF852740Calanthe davidii
MK235055Calanthe davidii
MK235053Calanthe cardioglossa
AF074117Calanthe vestita
KF296693Liparis kumokiri
MK235043Calanthe alpina
MK235069Calanthe puberula
MW888239Calanthe puberula

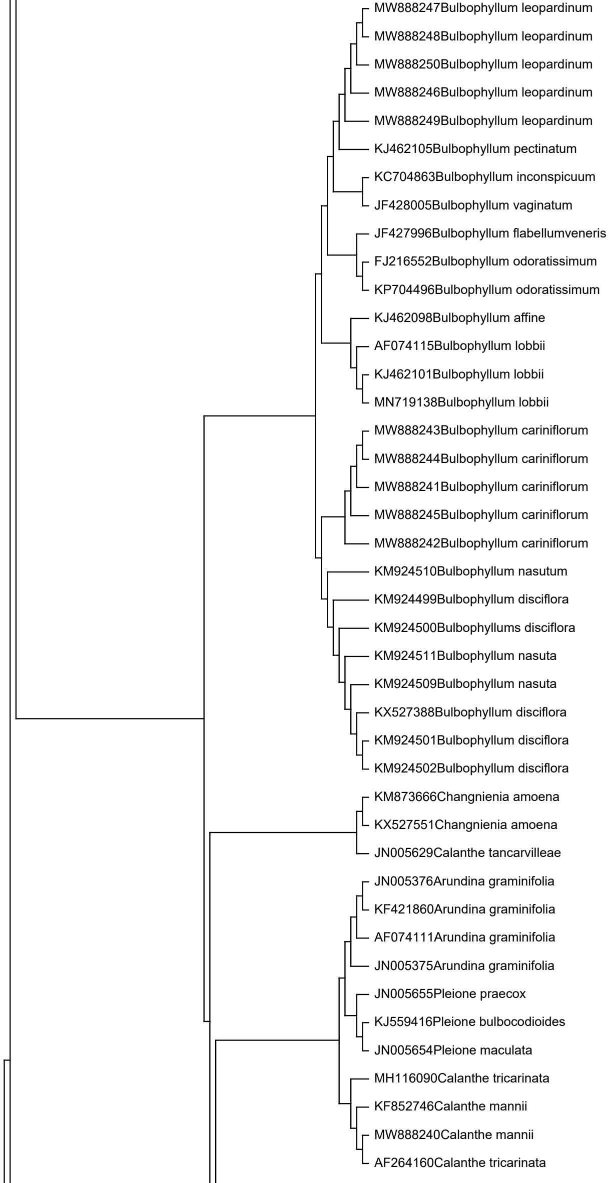

MW888247Bulbophyllum leopardinum
MW888248Bulbophyllum leopardinum
MW888250Bulbophyllum leopardinum
MW888246Bulbophyllum leopardinum
MW888249Bulbophyllum leopardinum
KJ462105Bulbophyllum pectinatum
KC704863Bulbophyllum inconspicuum
JF428005Bulbophyllum vaginatum
JF427996Bulbophyllum flabellumveneris
FJ216552Bulbophyllum odoratissimum
KP704496Bulbophyllum odoratissimum
KJ462098Bulbophyllum affine
AF074115Bulbophyllum lobbii
KJ462101Bulbophyllum lobbii
MN719138Bulbophyllum lobbii
MW888243Bulbophyllum cariniflorum
MW888244Bulbophyllum cariniflorum
MW888241Bulbophyllum cariniflorum
MW888245Bulbophyllum cariniflorum
MW888242Bulbophyllum cariniflorum
KM924510Bulbophyllum nasutum
KM924499Bulbophyllum disciflora
KM924500Bulbophyllums disciflora
KM924511Bulbophyllum nasuta
KM924509Bulbophyllum nasuta
KX527388Bulbophyllum disciflora
KM924501Bulbophyllum disciflora
KM924502Bulbophyllum disciflora
KM873666Changnienia amoena
KX527551Changnienia amoena
JN005629Calanthe tancarvilleae
JN005376Arundina graminifolia
KF421860Arundina graminifolia
AF074111Arundina graminifolia
JN005375Arundina graminifolia
JN005655Pleione praecox
KJ559416Pleione bulbocodioides
JN005654Pleione maculata
MH116090Calanthe tricarinata
KF852746Calanthe mannii
MW888240Calanthe mannii
AF264160Calanthe tricarinata

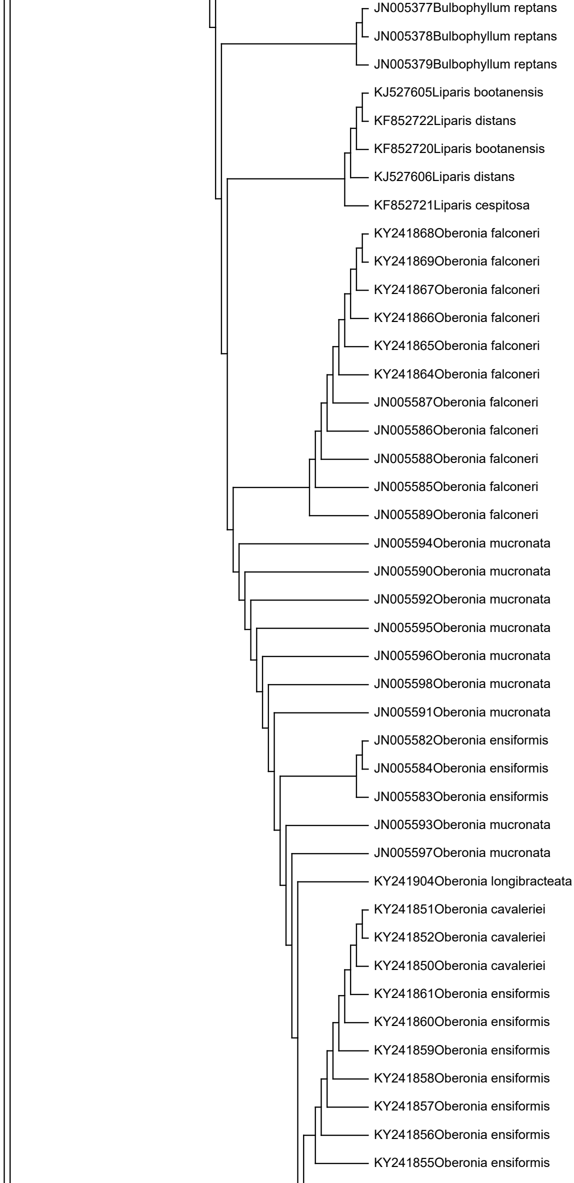
JN005377Bulbophyllum reptans
JN005378Bulbophyllum reptans
JN005379Bulbophyllum reptans
KJ527605Liparis bootanensis
KF852722Liparis distans
KF852720Liparis bootanensis
KJ527606Liparis distans
KF852721Liparis cespitosa
KY241868Oberonia falconeri
KY241869Oberonia falconeri
KY241867Oberonia falconeri
KY241866Oberonia falconeri
KY241865Oberonia falconeri
KY241864Oberonia falconeri
JN005587Oberonia falconeri
JN005586Oberonia falconeri
JN005588Oberonia falconeri
JN005585Oberonia falconeri
JN005589Oberonia falconeri
JN005594Oberonia mucronata
JN005590Oberonia mucronata
JN005592Oberonia mucronata
JN005595Oberonia mucronata
JN005596Oberonia mucronata
JN005598Oberonia mucronata
JN005591Oberonia mucronata
JN005582Oberonia ensiformis
JN005584Oberonia ensiformis
JN005583Oberonia ensiformis
JN005593Oberonia mucronata
JN005597Oberonia mucronata
KY241904Oberonia longibracteata
KY241851Oberonia cavaleriei
KY241852Oberonia cavaleriei
KY241850Oberonia cavaleriei
KY241861Oberonia ensiformis
KY241860Oberonia ensiformis
KY241859Oberonia ensiformis
KY241858Oberonia ensiformis
KY241857Oberonia ensiformis
KY241856Oberonia ensiformis
KY241855Oberonia ensiformis

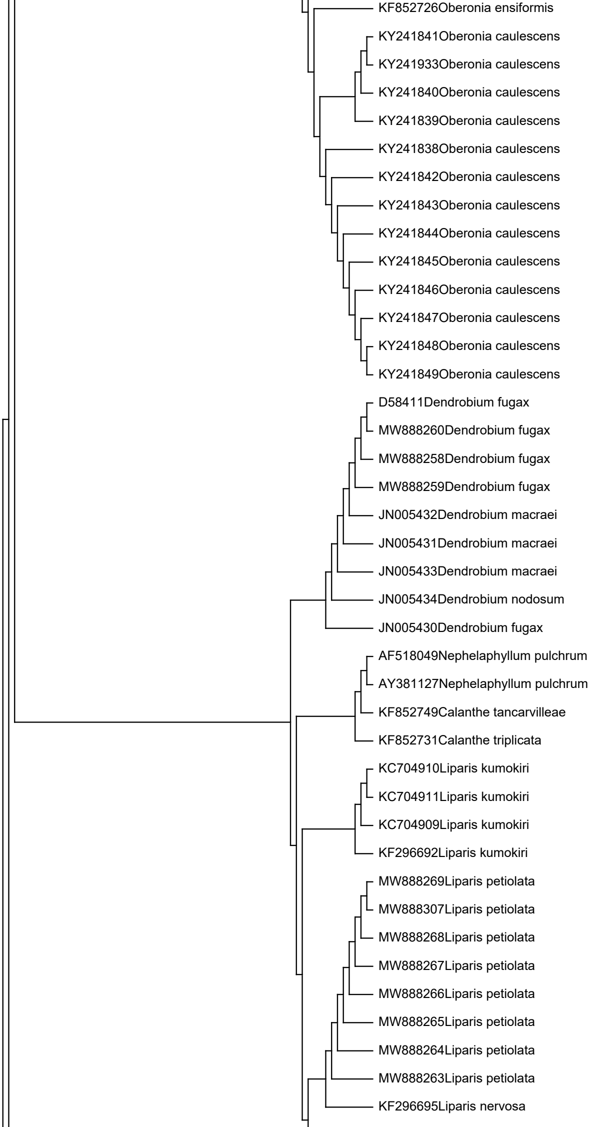

KF852726Oberonia ensiformis
KY241841Oberonia caulescens
KY241933Oberonia caulescens
KY241840Oberonia caulescens
KY241839Oberonia caulescens
KY241838Oberonia caulescens
KY241842Oberonia caulescens
KY241843Oberonia caulescens
KY241844Oberonia caulescens
KY241845Oberonia caulescens
KY241846Oberonia caulescens
KY241847Oberonia caulescens
KY241848Oberonia caulescens
KY241849Oberonia caulescens
D58411Dendrobium fugax
MW888260Dendrobium fugax
MW888258Dendrobium fugax
MW888259Dendrobium fugax
JN005432Dendrobium macraei
JN005431Dendrobium macraei
JN005433Dendrobium macraei
JN005434Dendrobium nodosum
JN005430Dendrobium fugax
AF518049Nephelaphyllum pulchrum
AY381127Nephelaphyllum pulchrum
KF852749Calanthe tancarvilleae
KF852731Calanthe triplicata
KC704910Liparis kumokiri
KC704911Liparis kumokiri
KC704909Liparis kumokiri
KF296692Liparis kumokiri
MW888269Liparis petiolata
MW888307Liparis petiolata
MW888268Liparis petiolata
MW888267Liparis petiolata
MW888266Liparis petiolata
MW888265Liparis petiolata
MW888264Liparis petiolata
MW888263Liparis petiolata
KF296695Liparis nervosa

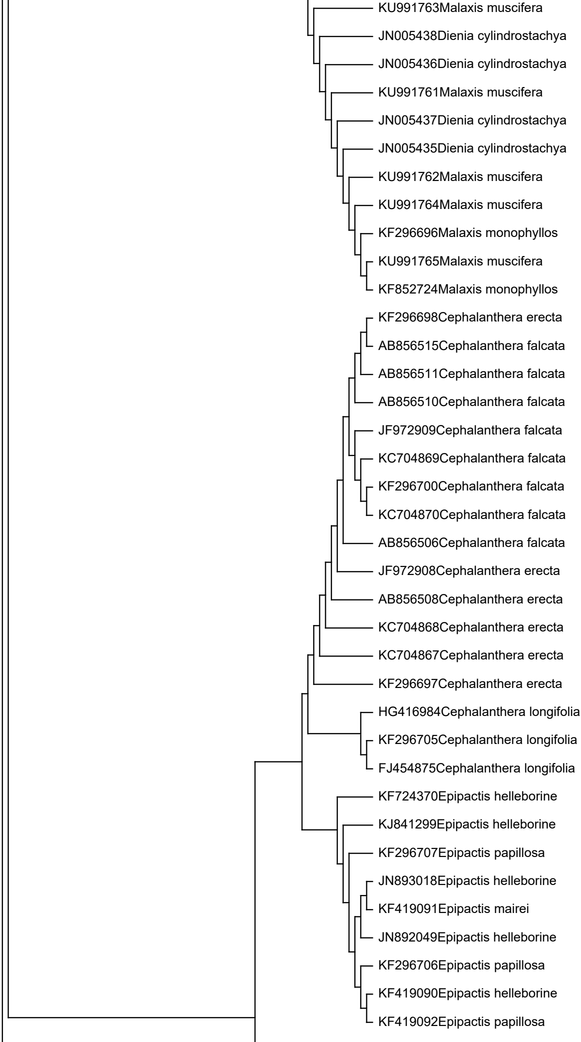

KU991763Malaxis muscifera
JN005438Dienia cylindrostachya
JN005436Dienia cylindrostachya
KU991761Malaxis muscifera
JN005437Dienia cylindrostachya
JN005435Dienia cylindrostachya
KU991762Malaxis muscifera
KU991764Malaxis muscifera
KF296696Malaxis monophyllos
KU991765Malaxis muscifera
KF852724Malaxis monophyllos
KF296698Cephalanthera erecta
AB856515Cephalanthera falcata
AB856511Cephalanthera falcata
AB856510Cephalanthera falcata
JF972909Cephalanthera falcata
KC704869Cephalanthera falcata
KF296700Cephalanthera falcata
KC704870Cephalanthera falcata
AB856506Cephalanthera falcata
JF972908Cephalanthera erecta
AB856508Cephalanthera erecta
KC704868Cephalanthera erecta
KC704867Cephalanthera erecta
KF296697Cephalanthera erecta
HG416984Cephalanthera longifolia
KF296705Cephalanthera longifolia
FJ454875Cephalanthera longifolia
KF724370Epipactis helleborine
KJ841299Epipactis helleborine
KF296707Epipactis papillosa
JN893018Epipactis helleborine
KF419091Epipactis mairei
JN892049Epipactis helleborine
KF296706Epipactis papillosa
KF419090Epipactis helleborine
KF419092Epipactis papillosa

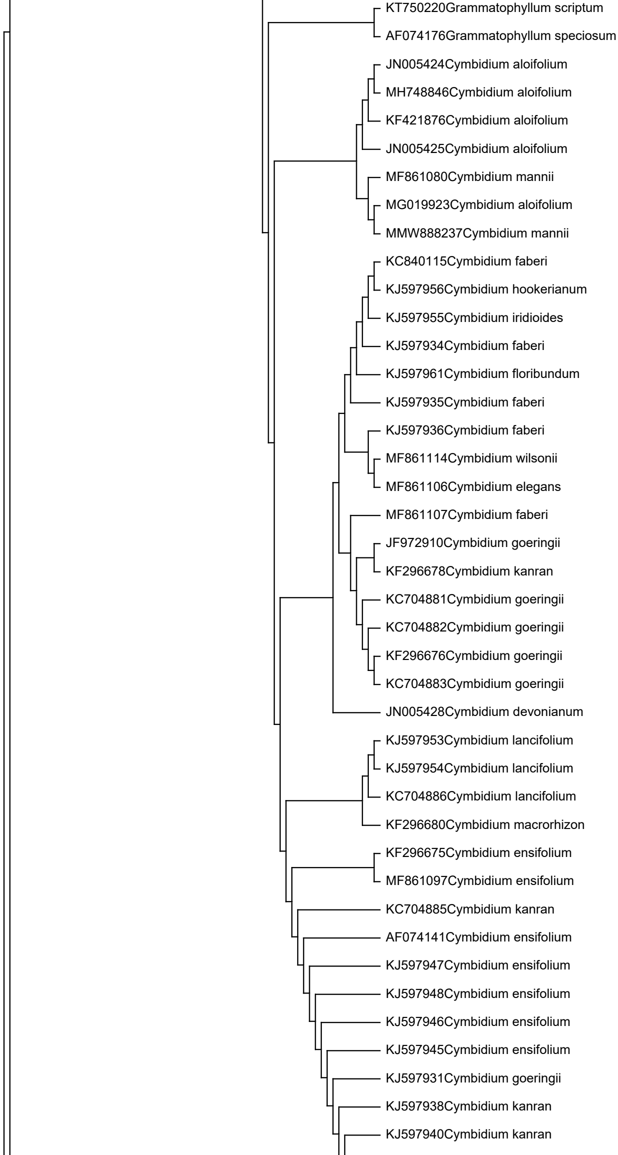
KT750220Grammatophyllum scriptum
AF074176Grammatophyllum speciosum
JN005424Cymbidium aloifolium
MH748846Cymbidium aloifolium
KF421876Cymbidium aloifolium
JN005425Cymbidium aloifolium
MF861080Cymbidium mannii
MG019923Cymbidium aloifolium
MMW888237Cymbidium mannii
KC840115Cymbidium faberi
KJ597956Cymbidium hookerianum
KJ597955Cymbidium iridioides
KJ597934Cymbidium faberi
KJ597961Cymbidium floribundum
KJ597935Cymbidium faberi
KJ597936Cymbidium faberi
MF861114Cymbidium wilsonii
MF861106Cymbidium elegans
MF861107Cymbidium faberi
JF972910Cymbidium goeringii
KF296678Cymbidium kanran
KC704881Cymbidium goeringii
KC704882Cymbidium goeringii
KF296676Cymbidium goeringii
KC704883Cymbidium goeringii
JN005428Cymbidium devonianum
KJ597953Cymbidium lancifolium
KJ597954Cymbidium lancifolium
KC704886Cymbidium lancifolium
KF296680Cymbidium macrorhizon
KF296675Cymbidium ensifolium
MF861097Cymbidium ensifolium
KC704885Cymbidium kanran
AF074141Cymbidium ensifolium
KJ597947Cymbidium ensifolium
KJ597948Cymbidium ensifolium
KJ597946Cymbidium ensifolium
KJ597945Cymbidium ensifolium
KJ597931Cymbidium goeringii
KJ597938Cymbidium kanran
KJ597940Cymbidium kanran

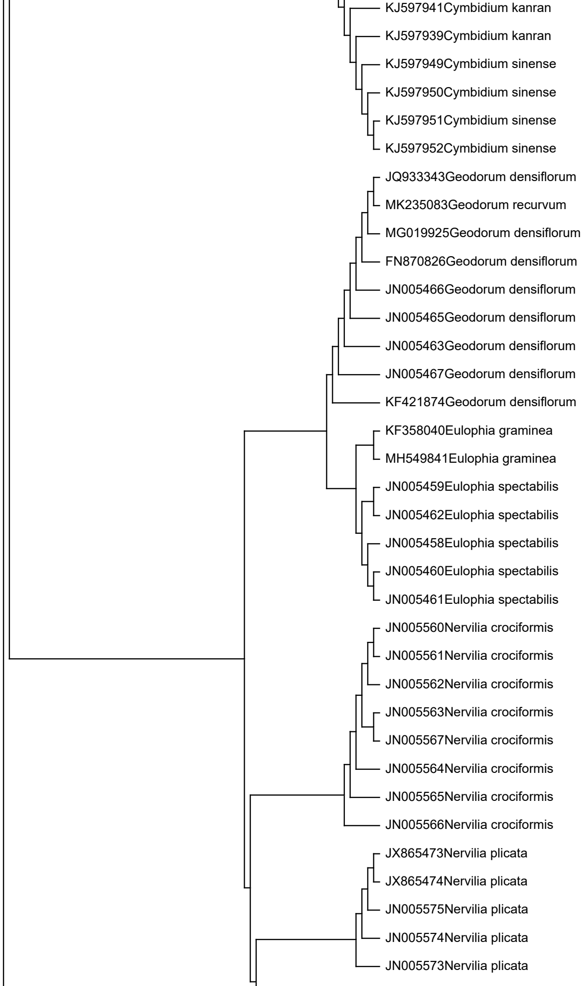
KJ597941Cymbidium kanran
KJ597939Cymbidium kanran
KJ597949Cymbidium sinense
KJ597950Cymbidium sinense
KJ597951Cymbidium sinense
KJ597952Cymbidium sinense
JQ933343Geodorum densiflorum
MK235083Geodorum recurvum
MG019925Geodorum densiflorum
FN870826Geodorum densiflorum
JN005466Geodorum densiflorum
JN005465Geodorum densiflorum
JN005463Geodorum densiflorum
JN005467Geodorum densiflorum
KF421874Geodorum densiflorum
KF358040Eulophia graminea
MH549841Eulophia graminea
JN005459Eulophia spectabilis
JN005462Eulophia spectabilis
JN005458Eulophia spectabilis
JN005460Eulophia spectabilis
JN005461Eulophia spectabilis
JN005560Nervilia crociformis
JN005561Nervilia crociformis
JN005562Nervilia crociformis
JN005563Nervilia crociformis
JN005567Nervilia crociformis
JN005564Nervilia crociformis
JN005565Nervilia crociformis
JN005566Nervilia crociformis
JX865473Nervilia plicata
JX865474Nervilia plicata
JN005575Nervilia plicata
JN005574Nervilia plicata
JN005573Nervilia plicata

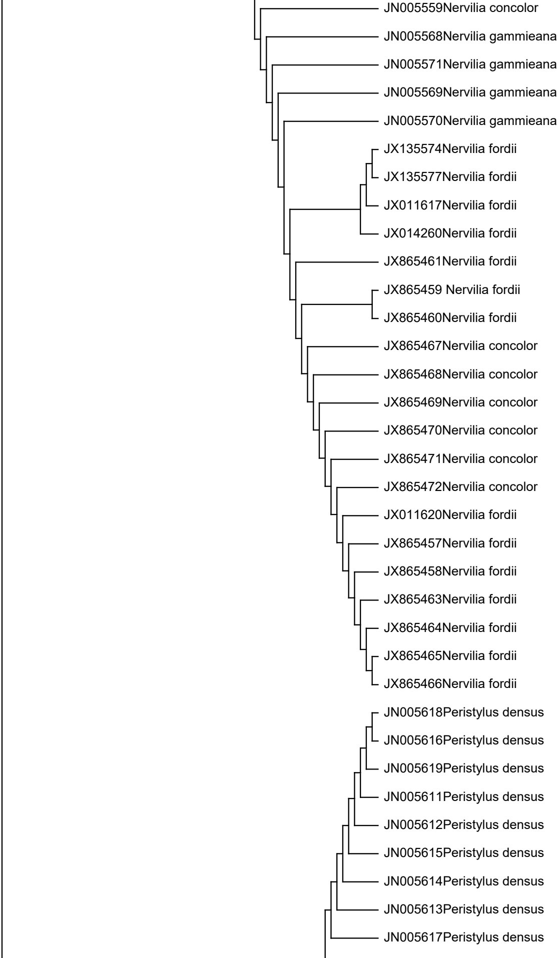
JN005559Nervilia concolor
JN005568Nervilia gammieana
JN005571Nervilia gammieana
JN005569Nervilia gammieana
JN005570Nervilia gammieana
JX135574Nervilia fordii
JX135577Nervilia fordii
JX011617Nervilia fordii
JX014260Nervilia fordii
JX865461Nervilia fordii
JX865459 Nervilia fordii
JX865460Nervilia fordii
JX865467Nervilia concolor
JX865468Nervilia concolor
JX865469Nervilia concolor
JX865470Nervilia concolor
JX865471Nervilia concolor
JX865472Nervilia concolor
JX011620Nervilia fordii
JX865457Nervilia fordii
JX865458Nervilia fordii
JX865463Nervilia fordii
JX865464Nervilia fordii
JX865465Nervilia fordii
JX865466Nervilia fordii
JN005618Peristylus densus
JN005616Peristylus densus
JN005619Peristylus densus
JN005611Peristylus densus
JN005612Peristylus densus
JN005615Peristylus densus
JN005614Peristylus densus
JN005613Peristylus densus
JN005617Peristylus densus

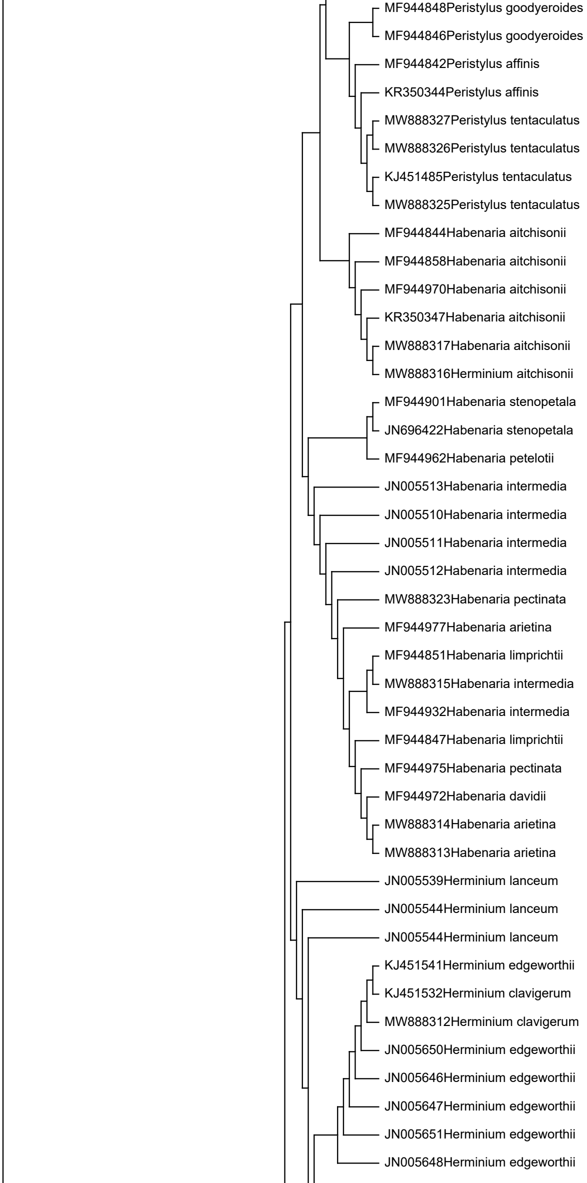
MF944848Peristylus goodyeroides
MF944846Peristylus goodyeroides
MF944842Peristylus affinis
KR350344Peristylus affinis
MW888327Peristylus tentaculatus
MW888326Peristylus tentaculatus
KJ451485Peristylus tentaculatus
MW888325Peristylus tentaculatus
MF944844Habenaria aitchisonii
MF944858Habenaria aitchisonii
MF944970Habenaria aitchisonii
KR350347Habenaria aitchisonii
MW888317Habenaria aitchisonii
MW888316Herminium aitchisonii
MF944901Habenaria stenopetala
JN696422Habenaria stenopetala
MF944962Habenaria petelotii
JN005513Habenaria intermedia
JN005510Habenaria intermedia
JN005511Habenaria intermedia
JN005512Habenaria intermedia
MW888323Habenaria pectinata
MF944977Habenaria arietina
MF944851Habenaria limprichtii
MW888315Habenaria intermedia
MF944932Habenaria intermedia
MF944847Habenaria limprichtii
MF944975Habenaria pectinata
MF944972Habenaria davidii
MW888314Habenaria arietina
MW888313Habenaria arietina
JN005539Herminium lanceum
JN005544Herminium lanceum
JN005544Herminium lanceum
KJ451541Herminium edgeworthii
KJ451532Herminium clavigerum
MW888312Herminium clavigerum
JN005650Herminium edgeworthii
JN005646Herminium edgeworthii
JN005647Herminium edgeworthii
JN005651Herminium edgeworthii
JN005648Herminium edgeworthii

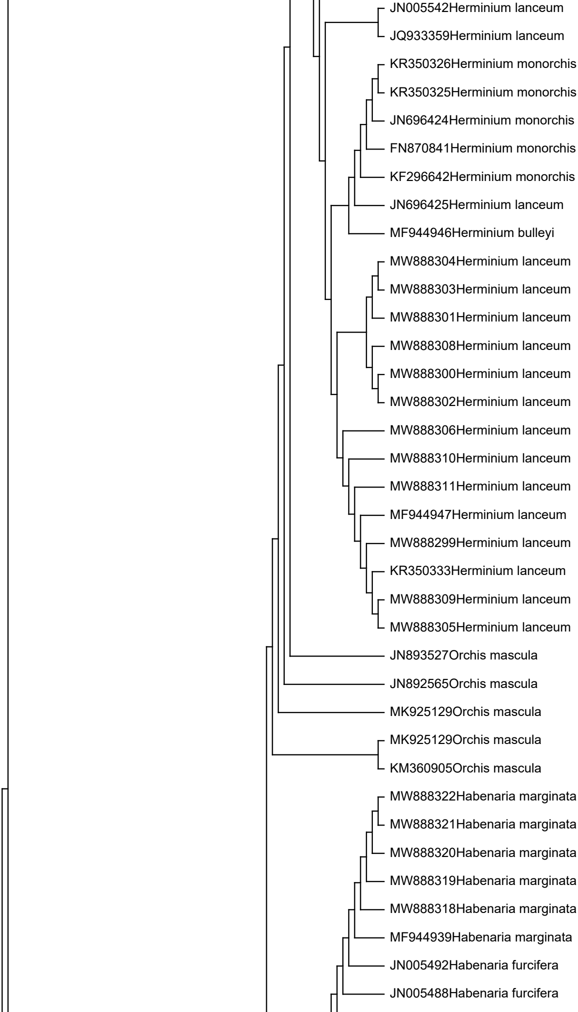
JN005542Herminium lanceum
JQ933359Herminium lanceum
KR350326Herminium monorchis
KR350325Herminium monorchis
JN696424Herminium monorchis
FN870841Herminium monorchis
KF296642Herminium monorchis
JN696425Herminium lanceum
MF944946Herminium bulleyi
MW888304Herminium lanceum
MW888303Herminium lanceum
MW888301Herminium lanceum
MW888308Herminium lanceum
MW888300Herminium lanceum
MW888302Herminium lanceum
MW888306Herminium lanceum
MW888310Herminium lanceum
MW888311Herminium lanceum
MF944947Herminium lanceum
MW888299Herminium lanceum
KR350333Herminium lanceum
MW888309Herminium lanceum
MW888305Herminium lanceum
JN893527Orchis mascula
JN892565Orchis mascula
MK925129Orchis mascula
MK925129Orchis mascula
KM360905Orchis mascula
MW888322Habenaria marginata
MW888321Habenaria marginata
MW888320Habenaria marginata
MW888319Habenaria marginata
MW888318Habenaria marginata
MF944939Habenaria marginata
JN005492Habenaria furcifera
JN005488Habenaria furcifera

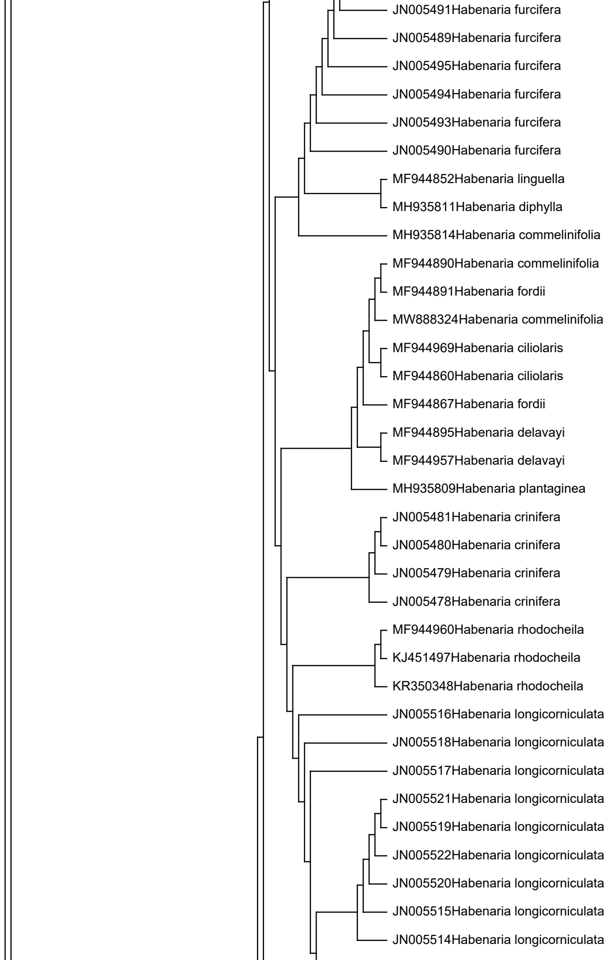

JN005491Habenaria furcifera
JN005489Habenaria furcifera
JN005495Habenaria furcifera
JN005494Habenaria furcifera
JN005493Habenaria furcifera
JN005490Habenaria furcifera
MF944852Habenaria linguella
MH935811Habenaria diphylla
MH935814Habenaria commelinifolia
MF944890Habenaria commelinifolia
MF944891Habenaria fordii
MW888324Habenaria commelinifolia
MF944969Habenaria ciliolaris
MF944860Habenaria ciliolaris
MF944867Habenaria fordii
MF944895Habenaria delavayi
MF944957Habenaria delavayi
MH935809Habenaria plantaginea
JN005481Habenaria crinifera
JN005480Habenaria crinifera
JN005479Habenaria crinifera
JN005478Habenaria crinifera
MF944960Habenaria rhodocheila
KJ451497Habenaria rhodocheila
KR350348Habenaria rhodocheila
JN005516Habenaria longicorniculata
JN005518Habenaria longicorniculata
JN005517Habenaria longicorniculata
JN005521Habenaria longicorniculata
JN005519Habenaria longicorniculata
JN005522Habenaria longicorniculata
JN005520Habenaria longicorniculata
JN005515Habenaria longicorniculata
JN005514Habenaria longicorniculata

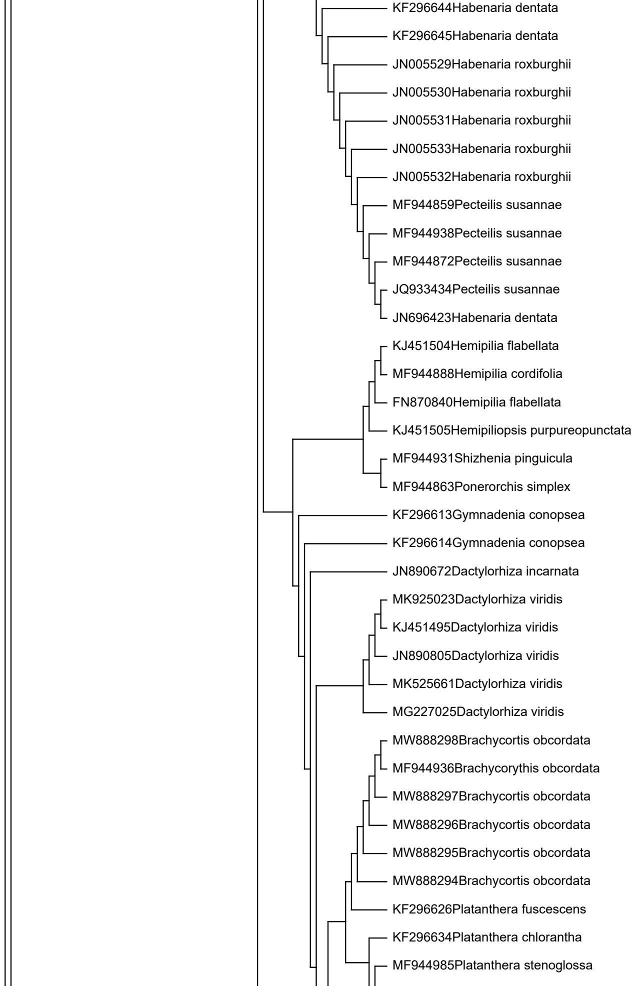
KF296644Habenaria dentata
KF296645Habenaria dentata
JN005529Habenaria roxburghii
JN005530Habenaria roxburghii
JN005531Habenaria roxburghii
JN005533Habenaria roxburghii
JN005532Habenaria roxburghii
MF944859Pecteilis susannae
MF944938Pecteilis susannae
MF944872Pecteilis susannae
JQ933434Pecteilis susannae
JN696423Habenaria dentata
KJ451504Hemipilia flabellata
MF944888Hemipilia cordifolia
FN870840Hemipilia flabellata
KJ451505Hemipiliopsis purpureopunctata
MF944931Shizhenia pinguicula
MF944863Ponerorchis simplex
KF296613Gymnadenia conopsea
KF296614Gymnadenia conopsea
JN890672Dactylorhiza incarnata
MK925023Dactylorhiza viridis
KJ451495Dactylorhiza viridis
JN890805Dactylorhiza viridis
MK525661Dactylorhiza viridis
MG227025Dactylorhiza viridis
MW888298Brachycortis obcordata
MF944936Brachycorythis obcordata
MW888297Brachycortis obcordata
MW888296Brachycortis obcordata
MW888295Brachycortis obcordata
MW888294Brachycortis obcordata
KF296626Platanthera fuscescens
KF296634Platanthera chlorantha
MF944985Platanthera stenoglossa

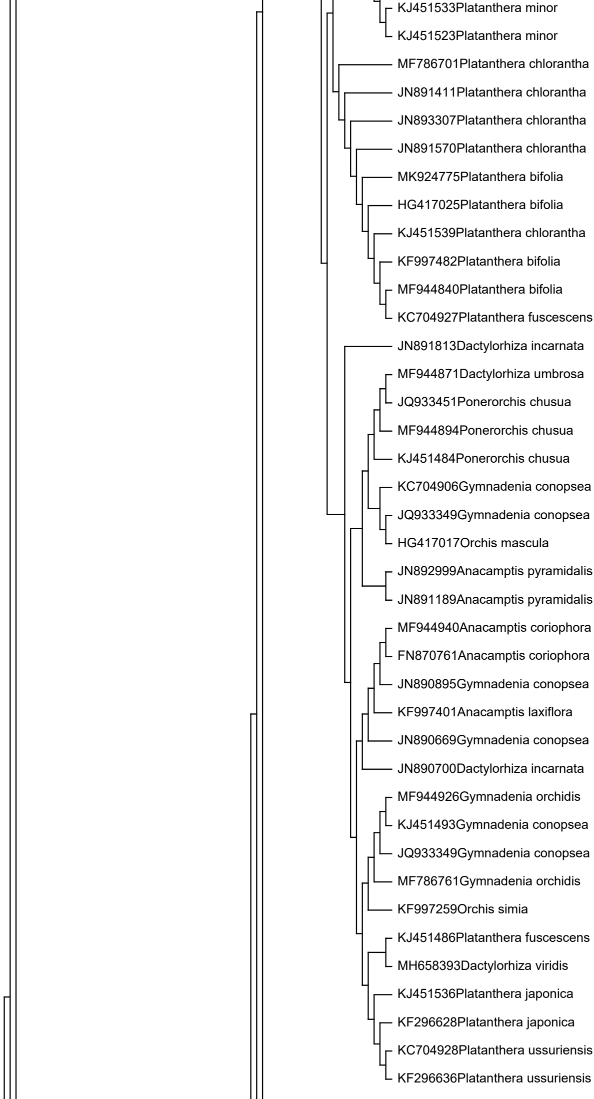
KJ451533Platanthera minor
KJ451523Platanthera minor
MF786701Platanthera chlorantha
JN891411Platanthera chlorantha
JN893307Platanthera chlorantha
JN891570Platanthera chlorantha
MK924775Platanthera bifolia
HG417025Platanthera bifolia
KJ451539Platanthera chlorantha
KF997482Platanthera bifolia
MF944840Platanthera bifolia
KC704927Platanthera fuscescens
JN891813Dactylorhiza incarnata
MF944871Dactylorhiza umbrosa
JQ933451Ponerorchis chusua
MF944894Ponerorchis chusua
KJ451484Ponerorchis chusua
KC704906Gymnadenia conopsea
JQ933349Gymnadenia conopsea
HG417017Orchis mascula
JN892999Anacamptis pyramidalis
JN891189Anacamptis pyramidalis
MF944940Anacamptis coriophora
FN870761Anacamptis coriophora
JN890895Gymnadenia conopsea
KF997401Anacamptis laxiflora
JN890669Gymnadenia conopsea
JN890700Dactylorhiza incarnata
MF944926Gymnadenia orchidis
KJ451493Gymnadenia conopsea
JQ933349Gymnadenia conopsea
MF786761Gymnadenia orchidis
KF997259Orchis simia
KJ451486Platanthera fuscescens
MH658393Dactylorhiza viridis
KJ451536Platanthera japonica
KF296628Platanthera japonica
KC704928Platanthera ussuriensis
KF296636Platanthera ussuriensis

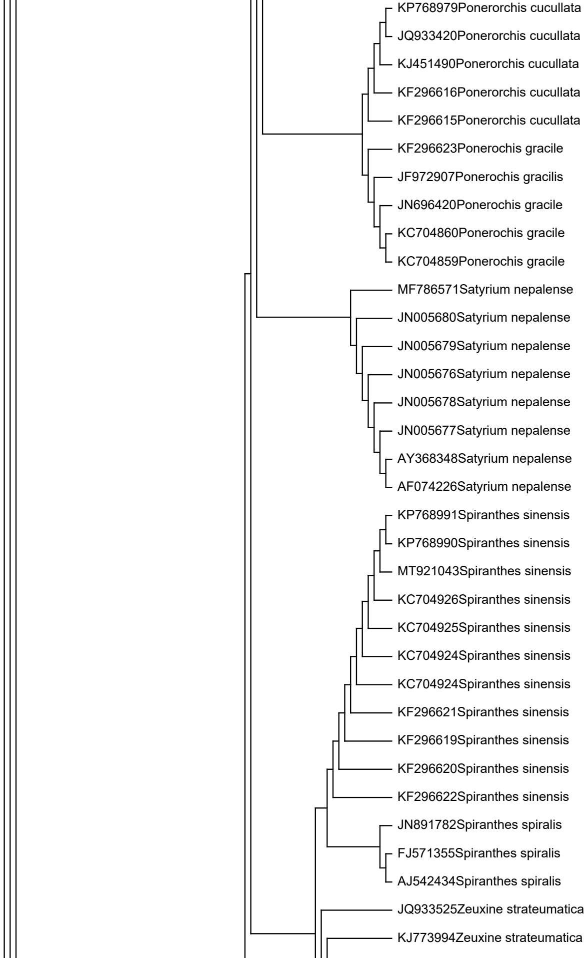
KP768979Ponerorchis cucullata
JQ933420Ponerorchis cucullata
KJ451490Ponerorchis cucullata
KF296616Ponerorchis cucullata
KF296615Ponerorchis cucullata
KF296623Ponerochis gracile
JF972907Ponerochis gracilis
JN696420Ponerochis gracile
KC704860Ponerochis gracile
KC704859Ponerochis gracile
MF786571Satyrium nepalense
JN005680Satyrium nepalense
JN005679Satyrium nepalense
JN005676Satyrium nepalense
JN005678Satyrium nepalense
JN005677Satyrium nepalense
AY368348Satyrium nepalense
AF074226Satyrium nepalense
KP768991Spiranthes sinensis
KP768990Spiranthes sinensis
MT921043Spiranthes sinensis
KC704926Spiranthes sinensis
KC704925Spiranthes sinensis
KC704924Spiranthes sinensis
KC704924Spiranthes sinensis
KF296621Spiranthes sinensis
KF296619Spiranthes sinensis
KF296620Spiranthes sinensis
KF296622Spiranthes sinensis
JN891782Spiranthes spiralis
FJ571355Spiranthes spiralis
AJ542434Spiranthes spiralis
JQ933525Zeuxine strateumatica
KJ773994Zeuxine strateumatica

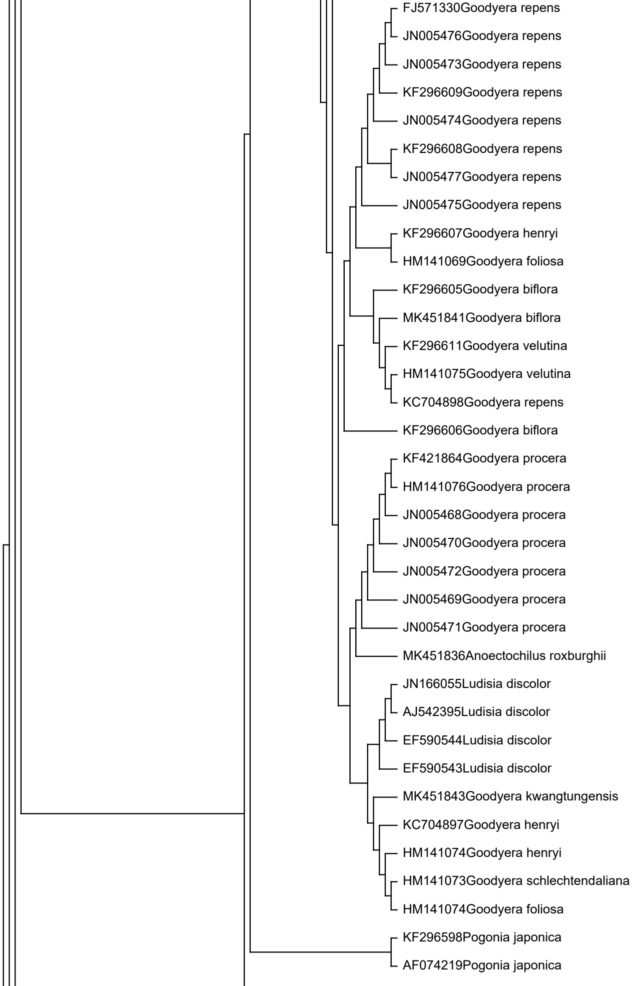
FJ571330Goodyera repens
JN005476Goodyera repens
JN005473Goodyera repens
KF296609Goodyera repens
JN005474Goodyera repens
KF296608Goodyera repens
JN005477Goodyera repens
JN005475Goodyera repens
KF296607Goodyera henryi
HM141069Goodyera foliosa
KF296605Goodyera biflora
MK451841Goodyera biflora
KF296611Goodyera velutina
HM141075Goodyera velutina
KC704898Goodyera repens
KF296606Goodyera biflora
KF421864Goodyera procera
HM141076Goodyera procera
JN005468Goodyera procera
JN005470Goodyera procera
JN005472Goodyera procera
JN005469Goodyera procera
JN005471Goodyera procera
MK451836Anoectochilus roxburghii
JN166055Ludisia discolor
AJ542395Ludisia discolor
EF590544Ludisia discolor
EF590543Ludisia discolor
MK451843Goodyera kwangtungensis
KC704897Goodyera henryi
HM141074Goodyera henryi
HM141073Goodyera schlechtendaliana
HM141074Goodyera foliosa
KF296598Pogonia japonica
AF074219Pogonia japonica

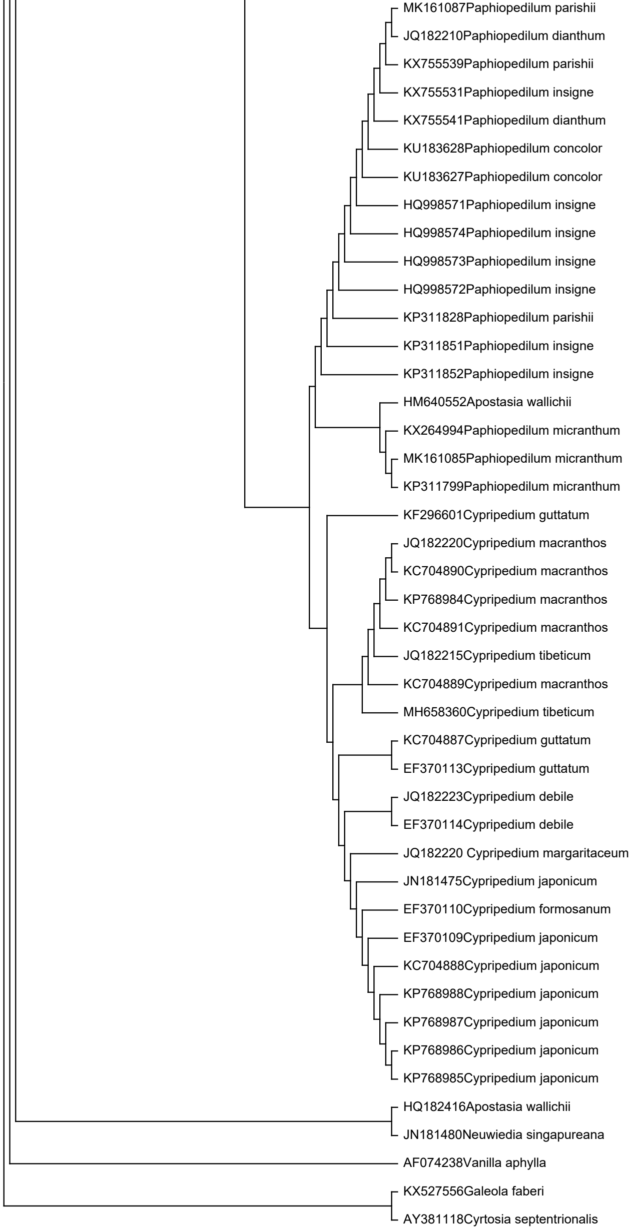
MK161087Paphiopedilum parishii
JQ182210Paphiopedilum dianthum
KX755539Paphiopedilum parishii
KX755531Paphiopedilum insigne
KX755541Paphiopedilum dianthum
KU183628Paphiopedilum concolor
KU183627Paphiopedilum concolor
HQ998571Paphiopedilum insigne
HQ998574Paphiopedilum insigne
HQ998573Paphiopedilum insigne
HQ998572Paphiopedilum insigne
KP311828Paphiopedilum parishii
KP311851Paphiopedilum insigne
KP311852Paphiopedilum insigne
HM640552Apostasia wallichii
KX264994Paphiopedilum micranthum
MK161085Paphiopedilum micranthum
KP311799Paphiopedilum micranthum
KF296601Cypripedium guttatum
JQ182220Cypripedium macranthos
KC704890Cypripedium macranthos
KP768984Cypripedium macranthos
KC704891Cypripedium macranthos
JQ182215Cypripedium tibeticum
KC704889Cypripedium macranthos
MH658360Cypripedium tibeticum
KC704887Cypripedium guttatum
EF370113Cypripedium guttatum
JQ182223Cypripedium debile
EF370114Cypripedium debile
JQ182220 Cypripedium margaritaceum
JN181475Cypripedium japonicum
EF370110Cypripedium formosanum
EF370109Cypripedium japonicum
KC704888Cypripedium japonicum
KP768988Cypripedium japonicum
KP768987Cypripedium japonicum
KP768986Cypripedium japonicum
KP768985Cypripedium japonicum
HQ182416Apostasia wallichii
JN181480Neuwiedia singapureana
AF074238Vanilla aphylla
KX527556Galeola faberi
AY381118Cyrtosia septentrionalis
