# Supplementary material for: DNA barcoding of medicinal orchids in Asia
Source: Sci Rep. 2021 Dec 8;11:23651. doi: 10.1038/s41598-021-03025-0 (PMC8654824; doi:10.1038/s41598-021-03025-0)

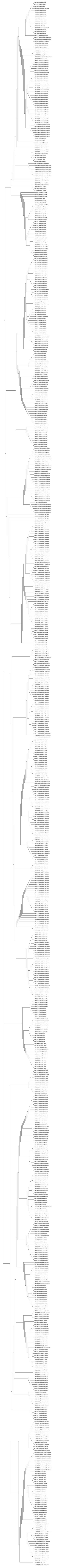

Supplement: Supplementary file 6 — Supplementary Figure S6. [file 41598_2021_3025_MOESM6_ESM.pdf]
