# Supplementary material for: DNA barcoding of medicinal orchids in Asia
Source: Sci Rep. 2021 Dec 8;11:23651. doi: 10.1038/s41598-021-03025-0 (PMC8654824; doi:10.1038/s41598-021-03025-0)
Supplement: Supplementary file 7 — Supplementary Figure S7. [file 41598_2021_3025_MOESM7_ESM.pdf]

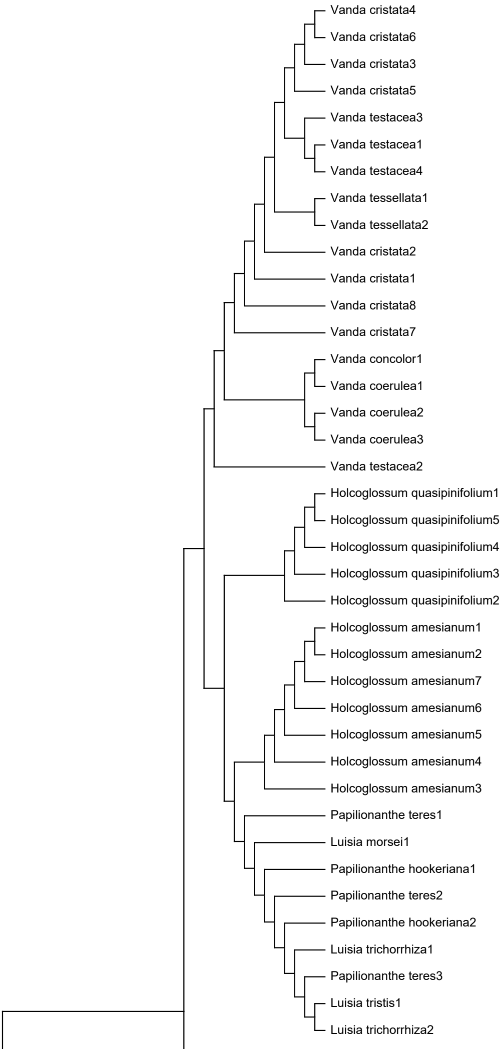
Vanda cristata4
Vanda cristata6
Vanda cristata3
Vanda cristata5
Vanda testacea3
Vanda testacea1
Vanda testacea4
Vanda tessellata1
Vanda tessellata2
Vanda cristata2
Vanda cristata1
Vanda cristata8
Vanda cristata7
Vanda concolor1
Vanda coerulea1
Vanda coerulea2
Vanda coerulea3
Vanda testacea2
Holcoglossum quasipinifolium1
Holcoglossum quasipinifolium5
Holcoglossum quasipinifolium4
Holcoglossum quasipinifolium3
Holcoglossum quasipinifolium2
Holcoglossum amesianum1
Holcoglossum amesianum2
Holcoglossum amesianum7
Holcoglossum amesianum6
Holcoglossum amesianum5
Holcoglossum amesianum4
Holcoglossum amesianum3
Papilionanthe teres1
Luisia morsei1
Papilionanthe hookeriana1
Papilionanthe teres2
Papilionanthe hookeriana2
Luisia trichorrhiza1
Papilionanthe teres3
Luisia tristis1
Luisia trichorrhiza2

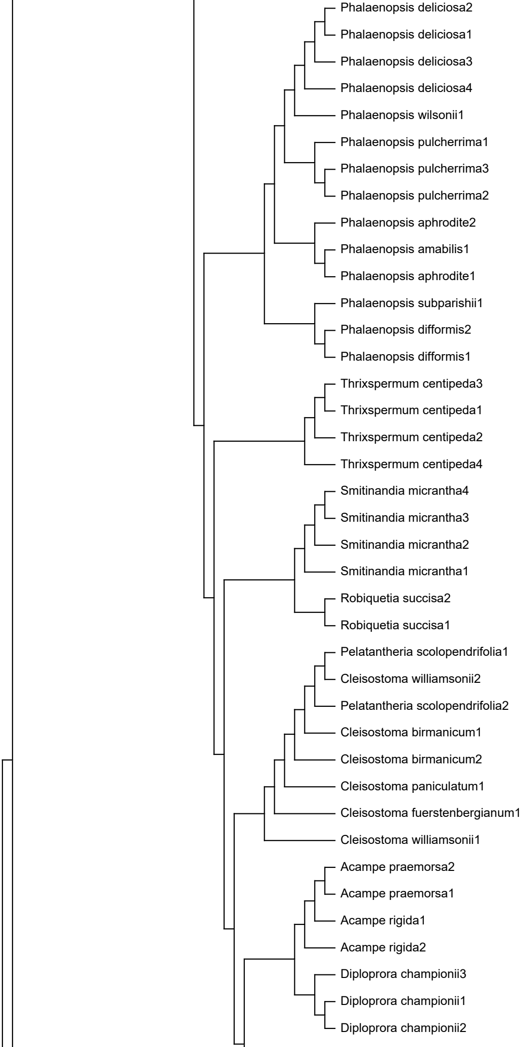
Phalaenopsis deliciosa2
Phalaenopsis deliciosa1
Phalaenopsis deliciosa3
Phalaenopsis deliciosa4
Phalaenopsis wilsonii1
Phalaenopsis pulcherrima1
Phalaenopsis pulcherrima3
Phalaenopsis pulcherrima2
Phalaenopsis aphrodite2
Phalaenopsis amabilis1
Phalaenopsis aphrodite1
Phalaenopsis subparishii1
Phalaenopsis difformis2
Phalaenopsis difformis1
Thrixspermum centipeda3
Thrixspermum centipeda1
Thrixspermum centipeda2
Thrixspermum centipeda4
Smitinandia micrantha4
Smitinandia micrantha3
Smitinandia micrantha2
Smitinandia micrantha1
Robiquetia succisa2
Robiquetia succisa1
Pelatantheria scolopendrifolia1
Cleisostoma williamsonii2
Pelatantheria scolopendrifolia2
Cleisostoma birmanicum1
Cleisostoma birmanicum2
Cleisostoma paniculatum1
Cleisostoma fuerstenbergianum1
Cleisostoma williamsonii1
Acampe praemorsa2
Acampe praemorsa1
Acampe rigida1
Acampe rigida2
Diploprora championii3
Diploprora championii1
Diploprora championii2

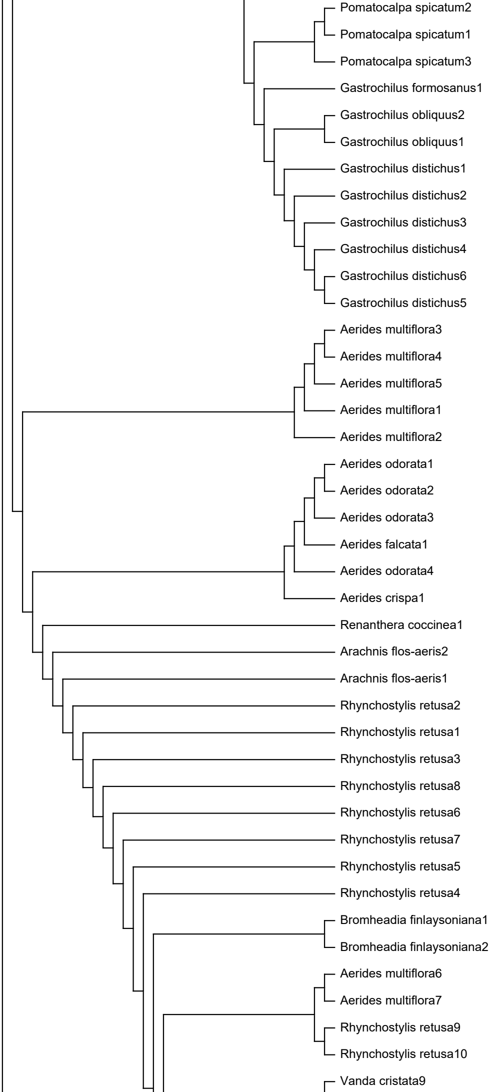

Pomatocalpa spicatum2
Pomatocalpa spicatum1
Pomatocalpa spicatum3
Gastrochilus formosanus1
Gastrochilus obliquus2
Gastrochilus obliquus1
Gastrochilus distichus1
Gastrochilus distichus2
Gastrochilus distichus3
Gastrochilus distichus4
Gastrochilus distichus6
Gastrochilus distichus5
Aerides multiflora3
Aerides multiflora4
Aerides multiflora5
Aerides multiflora1
Aerides multiflora2
Aerides odorata1
Aerides odorata2
Aerides odorata3
Aerides falcata1
Aerides odorata4
Aerides crispa1
Renanthera coccinea1
Arachnis flos-aeris2
Arachnis flos-aeris1
Rhynchostylis retusa2
Rhynchostylis retusa1
Rhynchostylis retusa3
Rhynchostylis retusa8
Rhynchostylis retusa6
Rhynchostylis retusa7
Rhynchostylis retusa5
Rhynchostylis retusa4
Bromheadia finlaysoniana1
Bromheadia finlaysoniana2
Aerides multiflora6
Aerides multiflora7
Rhynchostylis retusa9
Rhynchostylis retusa10
Vanda cristata9

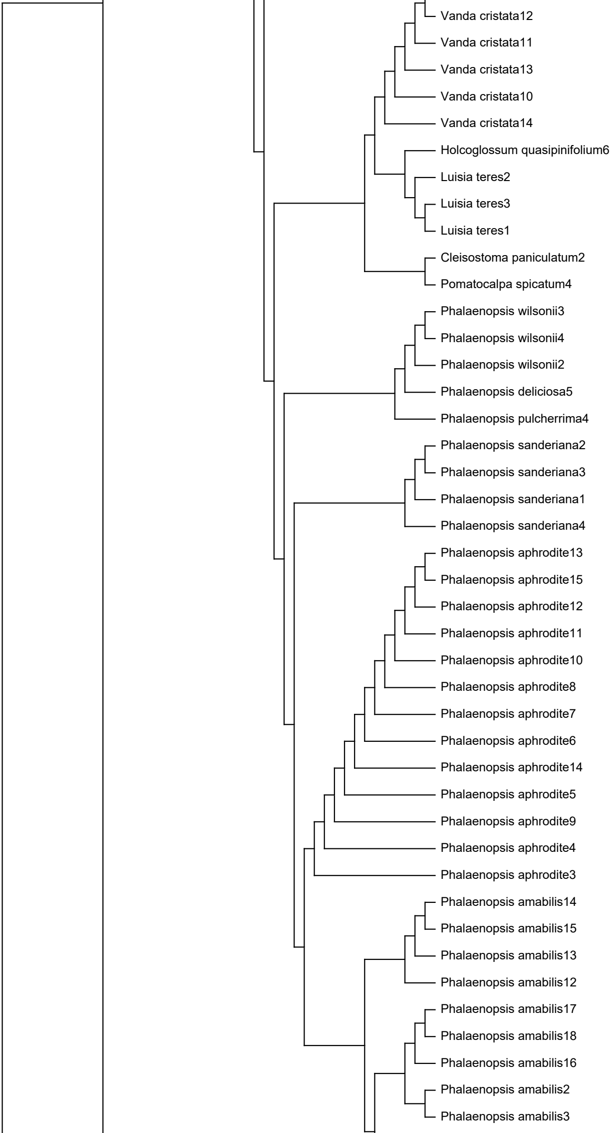
Vanda cristata12
Vanda cristata11
Vanda cristata13
Vanda cristata10
Vanda cristata14
Holcoglossum quasipinifolium6
Luisia teres2
Luisia teres3
Luisia teres1
Cleisostoma paniculatum2
Pomatocalpa spicatum4
Phalaenopsis wilsonii3
Phalaenopsis wilsonii4
Phalaenopsis wilsonii2
Phalaenopsis deliciosa5
Phalaenopsis pulcherrima4
Phalaenopsis sanderiana2
Phalaenopsis sanderiana3
Phalaenopsis sanderiana1
Phalaenopsis sanderiana4
Phalaenopsis aphrodite13
Phalaenopsis aphrodite15
Phalaenopsis aphrodite12
Phalaenopsis aphrodite11
Phalaenopsis aphrodite10
Phalaenopsis aphrodite8
Phalaenopsis aphrodite7
Phalaenopsis aphrodite6
Phalaenopsis aphrodite14
Phalaenopsis aphrodite5
Phalaenopsis aphrodite9
Phalaenopsis aphrodite4
Phalaenopsis aphrodite3
Phalaenopsis amabilis14
Phalaenopsis amabilis15
Phalaenopsis amabilis13
Phalaenopsis amabilis12
Phalaenopsis amabilis17
Phalaenopsis amabilis18
Phalaenopsis amabilis16
Phalaenopsis amabilis2
Phalaenopsis amabilis3

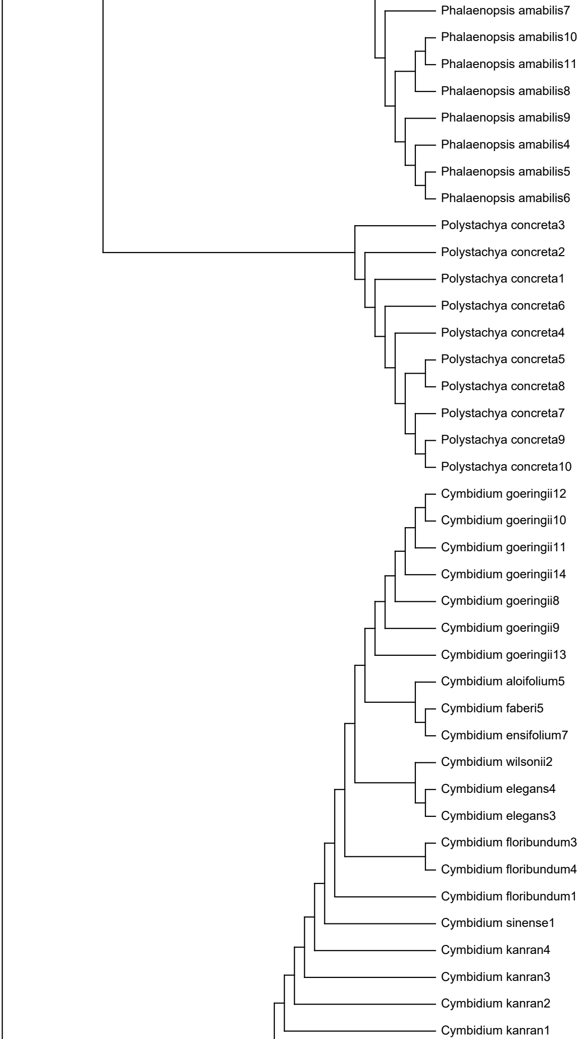
Phalaenopsis amabilis7
Phalaenopsis amabilis10
Phalaenopsis amabilis11
Phalaenopsis amabilis8
Phalaenopsis amabilis9
Phalaenopsis amabilis4
Phalaenopsis amabilis5
Phalaenopsis amabilis6
Polystachya concreta3
Polystachya concreta2
Polystachya concreta1
Polystachya concreta6
Polystachya concreta4
Polystachya concreta5
Polystachya concreta8
Polystachya concreta7
Polystachya concreta9
Polystachya concreta10
Cymbidium goeringii12
Cymbidium goeringii10
Cymbidium goeringii11
Cymbidium goeringii14
Cymbidium goeringii8
Cymbidium goeringii9
Cymbidium goeringii13
Cymbidium aloifolium5
Cymbidium faberi5
Cymbidium ensifolium7
Cymbidium wilsonii2
Cymbidium elegans4
Cymbidium elegans3
Cymbidium floribundum3
Cymbidium floribundum4
Cymbidium floribundum1
Cymbidium sinense1
Cymbidium kanran4
Cymbidium kanran3
Cymbidium kanran2
Cymbidium kanran1

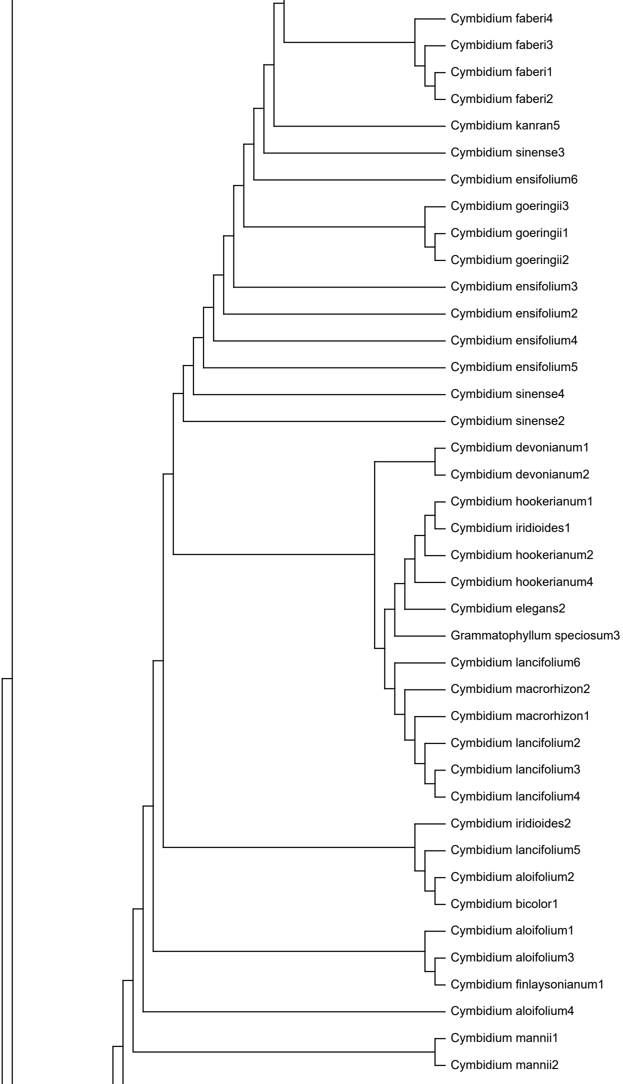
Cymbidium faberi4
Cymbidium faberi3
Cymbidium faberi1
Cymbidium faberi2
Cymbidium kanran5
Cymbidium sinense3
Cymbidium ensifolium6
Cymbidium goeringii3
Cymbidium goeringii1
Cymbidium goeringii2
Cymbidium ensifolium3
Cymbidium ensifolium2
Cymbidium ensifolium4
Cymbidium ensifolium5
Cymbidium sinense4
Cymbidium sinense2
Cymbidium devonianum1
Cymbidium devonianum2
Cymbidium hookerianum1
Cymbidium iridioides1
Cymbidium hookerianum2
Cymbidium hookerianum4
Cymbidium elegans2
Grammatophyllum speciosum3
Cymbidium lancifolium6
Cymbidium macrorhizon2
Cymbidium macrorhizon1
Cymbidium lancifolium2
Cymbidium lancifolium3
Cymbidium lancifolium4
Cymbidium iridioides2
Cymbidium lancifolium5
Cymbidium aloifolium2
Cymbidium bicolor1
Cymbidium aloifolium1
Cymbidium aloifolium3
Cymbidium finlaysonianum1
Cymbidium aloifolium4
Cymbidium mannii1
Cymbidium mannii2

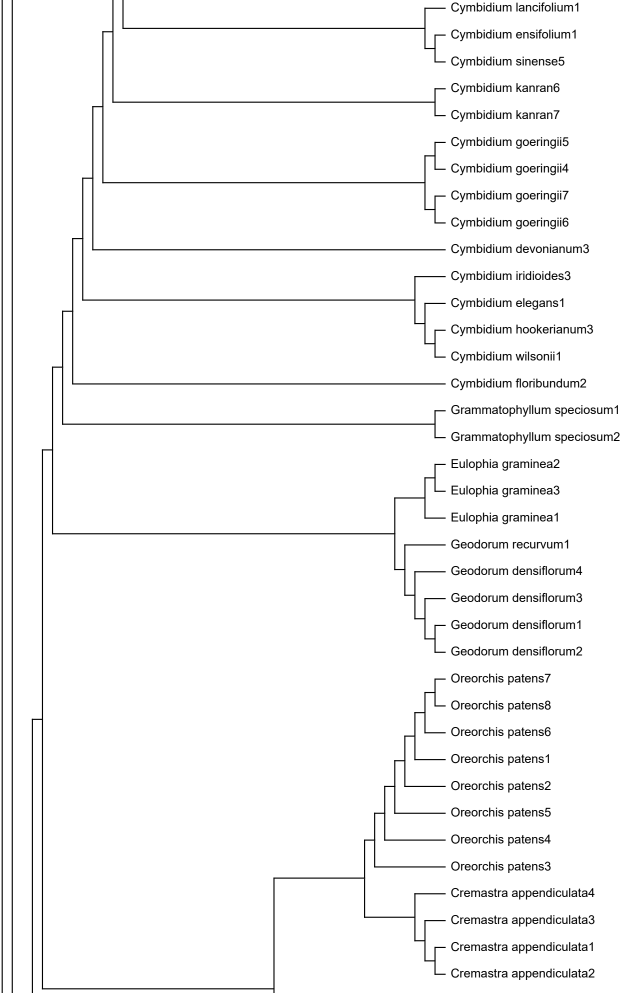
Cymbidium lancifolium1
Cymbidium ensifolium1
Cymbidium sinense5
Cymbidium kanran6
Cymbidium kanran7
Cymbidium goeringii5
Cymbidium goeringii4
Cymbidium goeringii7
Cymbidium goeringii6
Cymbidium devonianum3
Cymbidium iridioides3
Cymbidium elegans1
Cymbidium hookerianum3
Cymbidium wilsonii1
Cymbidium floribundum2
Grammatophyllum speciosum1
Grammatophyllum speciosum2
Eulophia graminea2
Eulophia graminea3
Eulophia graminea1
Geodorum recurvum1
Geodorum densiflorum4
Geodorum densiflorum3
Geodorum densiflorum1
Geodorum densiflorum2
Oreorchis patens7
Oreorchis patens8
Oreorchis patens6
Oreorchis patens1
Oreorchis patens2
Oreorchis patens5
Oreorchis patens4
Oreorchis patens3
Cremastra appendiculata4
Cremastra appendiculata3
Cremastra appendiculata1
Cremastra appendiculata2

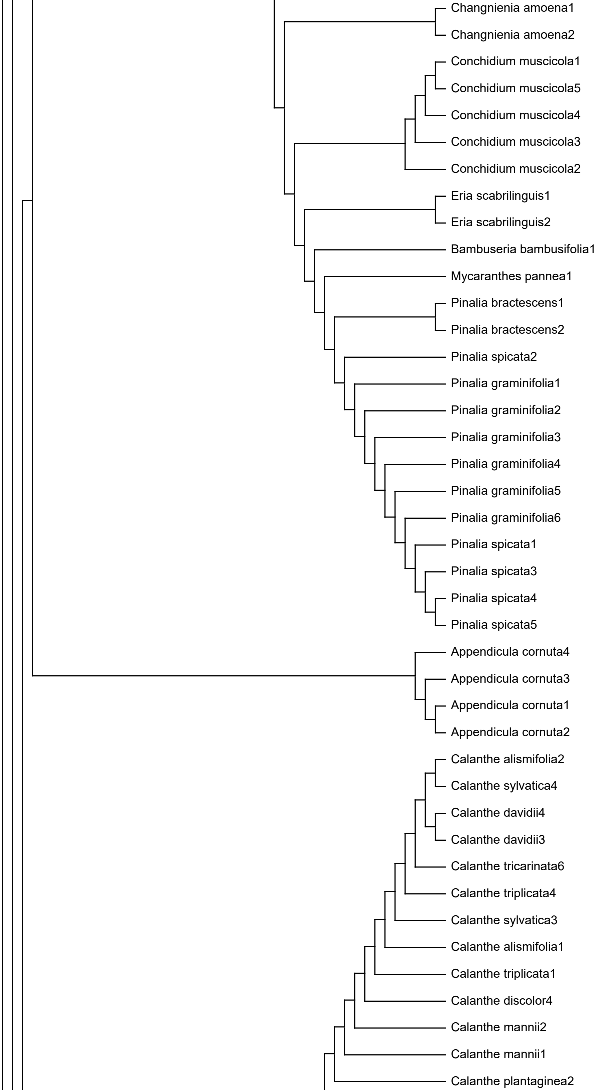
Changnienia amoena1
Changnienia amoena2
Conchidium muscicola1
Conchidium muscicola5
Conchidium muscicola4
Conchidium muscicola3
Conchidium muscicola2
Eria scabrilinguis1
Eria scabrilinguis2
Bambuseria bambusifolia1
Mycaranthes pannea1
Pinalia bractescens1
Pinalia bractescens2
Pinalia spicata2
Pinalia graminifolia1
Pinalia graminifolia2
Pinalia graminifolia3
Pinalia graminifolia4
Pinalia graminifolia5
Pinalia graminifolia6
Pinalia spicata1
Pinalia spicata3
Pinalia spicata4
Pinalia spicata5
Appendicula cornuta4
Appendicula cornuta3
Appendicula cornuta1
Appendicula cornuta2
Calanthe alismifolia2
Calanthe sylvatica4
Calanthe davidii4
Calanthe davidii3
Calanthe tricarinata6
Calanthe triplicata4
Calanthe sylvatica3
Calanthe alismifolia1
Calanthe triplicata1
Calanthe discolor4
Calanthe mannii2
Calanthe mannii1
Calanthe plantaginea2

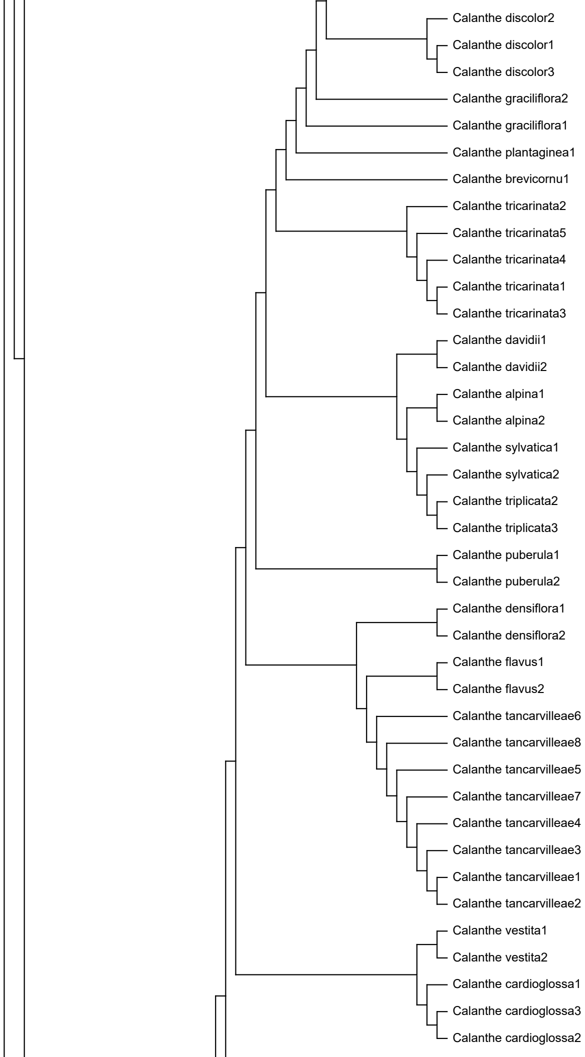
Calanthe discolor2
Calanthe discolor1
Calanthe discolor3
Calanthe graciliflora2
Calanthe graciliflora1
Calanthe plantaginea1
Calanthe brevicornu1
Calanthe tricarinata2
Calanthe tricarinata5
Calanthe tricarinata4
Calanthe tricarinata1
Calanthe tricarinata3
Calanthe davidii1
Calanthe davidii2
Calanthe alpina1
Calanthe alpina2
Calanthe sylvatica1
Calanthe sylvatica2
Calanthe triplicata2
Calanthe triplicata3
Calanthe puberula1
Calanthe puberula2
Calanthe densiflora1
Calanthe densiflora2
Calanthe flavus1
Calanthe flavus2
Calanthe tancarvilleae6
Calanthe tancarvilleae8
Calanthe tancarvilleae5
Calanthe tancarvilleae7
Calanthe tancarvilleae4
Calanthe tancarvilleae3
Calanthe tancarvilleae1
Calanthe tancarvilleae2
Calanthe vestita1
Calanthe vestita2
Calanthe cardioglossa1
Calanthe cardioglossa3
Calanthe cardioglossa2

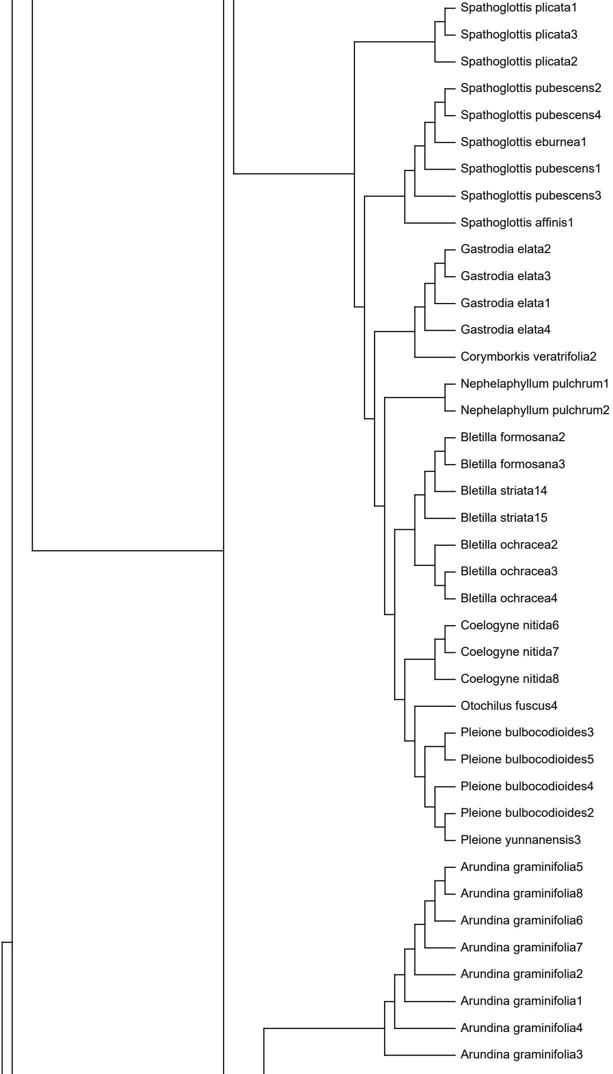
Spathoglottis plicata1
Spathoglottis plicata3
Spathoglottis plicata2
Spathoglottis pubescens2
Spathoglottis pubescens4
Spathoglottis eburnea1
Spathoglottis pubescens1
Spathoglottis pubescens3
Spathoglottis affinis1
Gastrodia elata2
Gastrodia elata3
Gastrodia elata1
Gastrodia elata4
Corymborkis veratrifolia2
Nephelaphyllum pulchrum1
Nephelaphyllum pulchrum2
Bletilla formosana2
Bletilla formosana3
Bletilla striata14
Bletilla striata15
Bletilla ochracea2
Bletilla ochracea3
Bletilla ochracea4
Coelogyne nitida6
Coelogyne nitida7
Coelogyne nitida8
Otochilus fuscus4
Pleione bulbocodioides3
Pleione bulbocodioides5
Pleione bulbocodioides4
Pleione bulbocodioides2
Pleione yunnanensis3
Arundina graminifolia5
Arundina graminifolia8
Arundina graminifolia6
Arundina graminifolia7
Arundina graminifolia2
Arundina graminifolia1
Arundina graminifolia4
Arundina graminifolia3

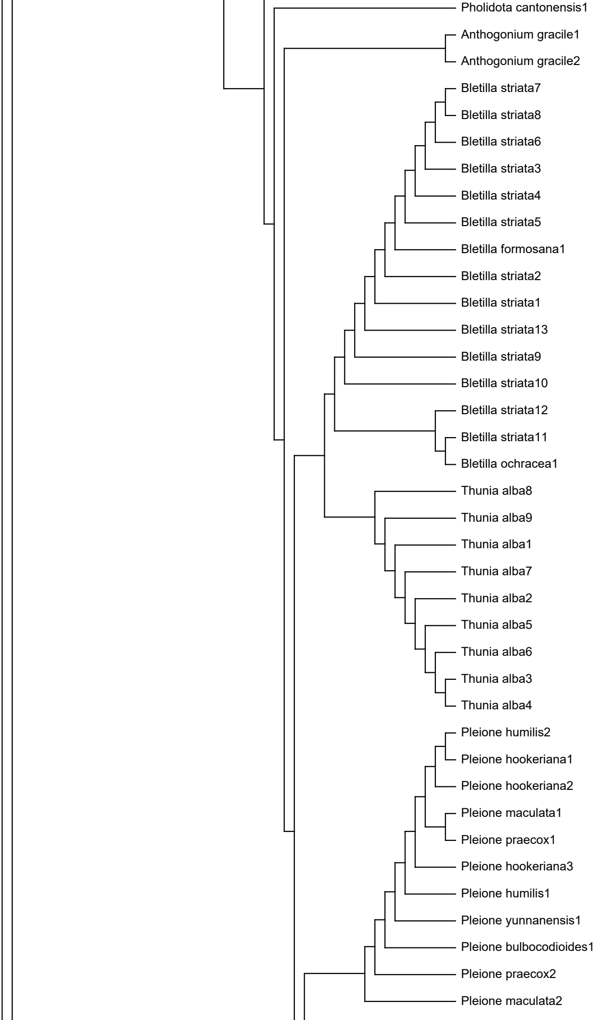
Pholidota cantonensis1
Anthogonium gracile1
Anthogonium gracile2
Bletilla striata7
Bletilla striata8
Bletilla striata6
Bletilla striata3
Bletilla striata4
Bletilla striata5
Bletilla formosana1
Bletilla striata2
Bletilla striata1
Bletilla striata13
Bletilla striata9
Bletilla striata10
Bletilla striata12
Bletilla striata11
Bletilla ochracea1
Thunia alba8
Thunia alba9
Thunia alba1
Thunia alba7
Thunia alba2
Thunia alba5
Thunia alba6
Thunia alba3
Thunia alba4
Pleione humilis2
Pleione hookeriana1
Pleione hookeriana2
Pleione maculata1
Pleione praecox1
Pleione hookeriana3
Pleione humilis1
Pleione yunnanensis1
Pleione bulbocodioides1
Pleione praecox2
Pleione maculata2

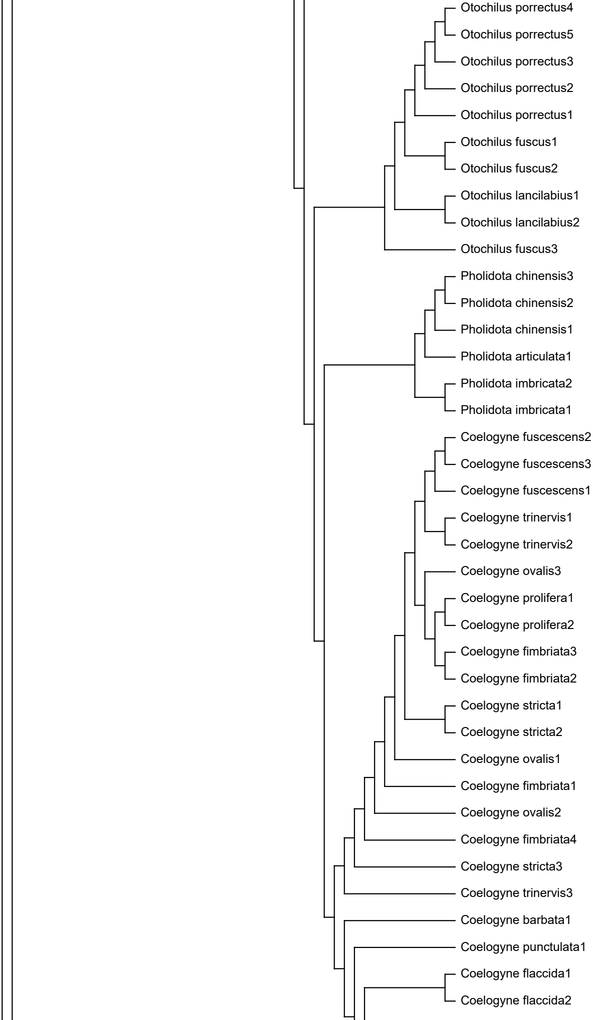
Otochilus porrectus4
Otochilus porrectus5
Otochilus porrectus3
Otochilus porrectus2
Otochilus porrectus1
Otochilus fuscus1
Otochilus fuscus2
Otochilus lancilabius1
Otochilus lancilabius2
Otochilus fuscus3
Pholidota chinensis3
Pholidota chinensis2
Pholidota chinensis1
Pholidota articulata1
Pholidota imbricata2
Pholidota imbricata1
Coelogyne fuscescens2
Coelogyne fuscescens3
Coelogyne fuscescens1
Coelogyne trinervis1
Coelogyne trinervis2
Coelogyne ovalis3
Coelogyne prolifera1
Coelogyne prolifera2
Coelogyne fimbriata3
Coelogyne fimbriata2
Coelogyne stricta1
Coelogyne stricta2
Coelogyne ovalis1
Coelogyne fimbriata1
Coelogyne ovalis2
Coelogyne fimbriata4
Coelogyne stricta3
Coelogyne trinervis3
Coelogyne barbata1
Coelogyne punctulata1
Coelogyne flaccida1
Coelogyne flaccida2

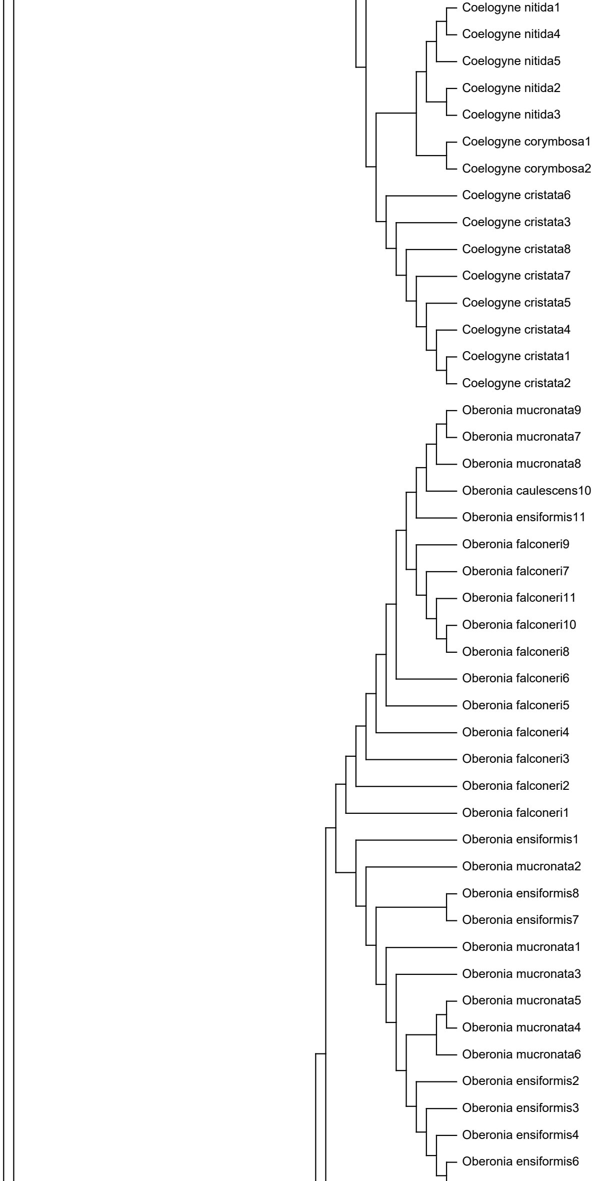
Coelogyne nitida1
Coelogyne nitida4
Coelogyne nitida5
Coelogyne nitida2
Coelogyne nitida3
Coelogyne corymbosa1
Coelogyne corymbosa2
Coelogyne cristata6
Coelogyne cristata3
Coelogyne cristata8
Coelogyne cristata7
Coelogyne cristata5
Coelogyne cristata4
Coelogyne cristata1
Coelogyne cristata2
Oberonia mucronata9
Oberonia mucronata7
Oberonia mucronata8
Oberonia caulescens10
Oberonia ensiformis11
Oberonia falconeri9
Oberonia falconeri7
Oberonia falconeri11
Oberonia falconeri10
Oberonia falconeri8
Oberonia falconeri6
Oberonia falconeri5
Oberonia falconeri4
Oberonia falconeri3
Oberonia falconeri2
Oberonia falconeri1
Oberonia ensiformis1
Oberonia mucronata2
Oberonia ensiformis8
Oberonia ensiformis7
Oberonia mucronata1
Oberonia mucronata3
Oberonia mucronata5
Oberonia mucronata4
Oberonia mucronata6
Oberonia ensiformis2
Oberonia ensiformis3
Oberonia ensiformis4
Oberonia ensiformis6

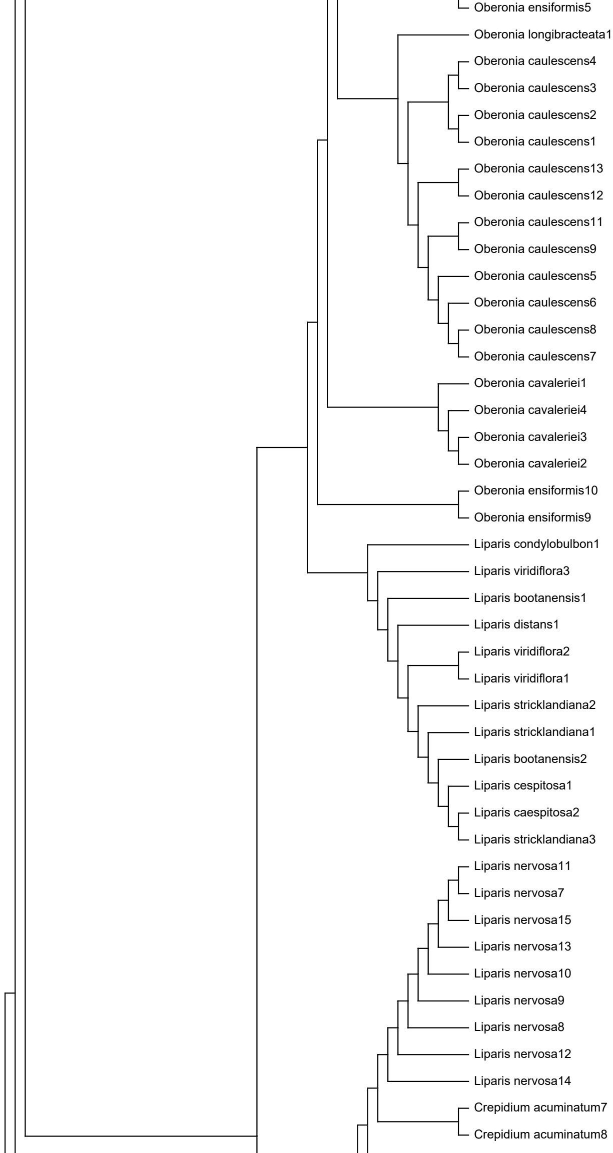
Oberonia ensiformis5
Oberonia longibracteata1
Oberonia caulescens4
Oberonia caulescens3
Oberonia caulescens2
Oberonia caulescens1
Oberonia caulescens13
Oberonia caulescens12
Oberonia caulescens11
Oberonia caulescens9
Oberonia caulescens5
Oberonia caulescens6
Oberonia caulescens8
Oberonia caulescens7
Oberonia cavaleriei1
Oberonia cavaleriei4
Oberonia cavaleriei3
Oberonia cavaleriei2
Oberonia ensiformis10
Oberonia ensiformis9
Liparis condylobulbon1
Liparis viridiflora3
Liparis bootanensis1
Liparis distans1
Liparis viridiflora2
Liparis viridiflora1
Liparis stricklandiana2
Liparis stricklandiana1
Liparis bootanensis2
Liparis cespitosa1
Liparis caespitosa2
Liparis stricklandiana3
Liparis nervosa11
Liparis nervosa7
Liparis nervosa15
Liparis nervosa13
Liparis nervosa10
Liparis nervosa9
Liparis nervosa8
Liparis nervosa12
Liparis nervosa14
Crepidium acuminatum7
Crepidium acuminatum8

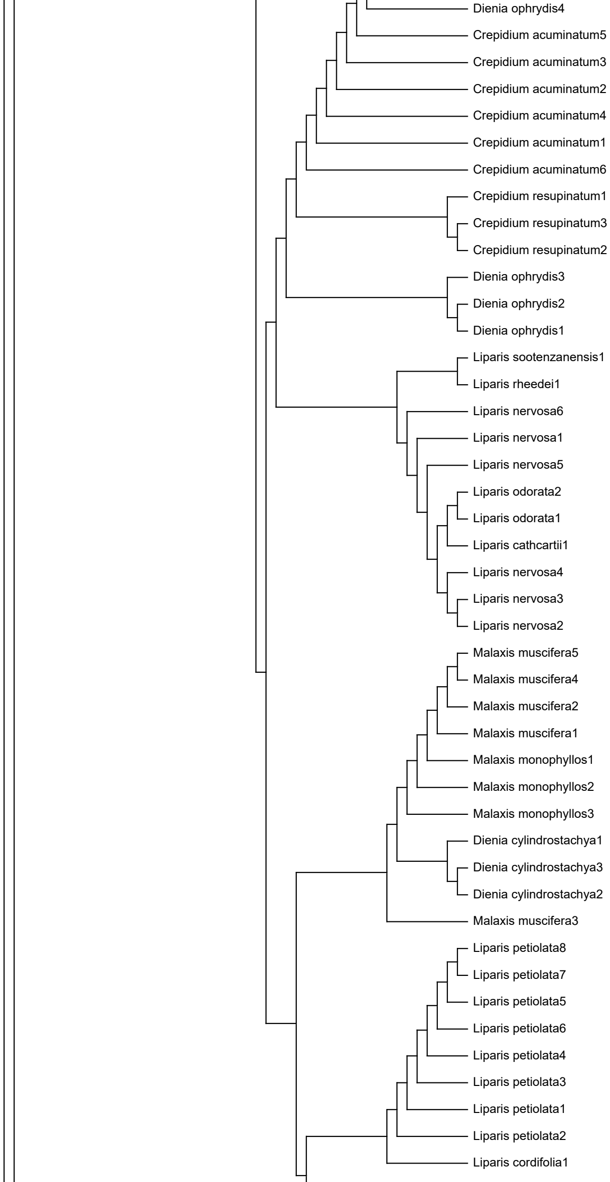
Dienia ophrydis4
Crepidium acuminatum5
Crepidium acuminatum3
Crepidium acuminatum2
Crepidium acuminatum4
Crepidium acuminatum1
Crepidium acuminatum6
Crepidium resupinatum1
Crepidium resupinatum3
Crepidium resupinatum2
Dienia ophrydis3
Dienia ophrydis2
Dienia ophrydis1
Liparis sootenzanensis1
Liparis rheedei1
Liparis nervosa6
Liparis nervosa1
Liparis nervosa5
Liparis odorata2
Liparis odorata1
Liparis cathcartii1
Liparis nervosa4
Liparis nervosa3
Liparis nervosa2
Malaxis muscifera5
Malaxis muscifera4
Malaxis muscifera2
Malaxis muscifera1
Malaxis monophyllos1
Malaxis monophyllos2
Malaxis monophyllos3
Dienia cylindrostachya1
Dienia cylindrostachya3
Dienia cylindrostachya2
Malaxis muscifera3
Liparis petiolata8
Liparis petiolata7
Liparis petiolata5
Liparis petiolata6
Liparis petiolata4
Liparis petiolata3
Liparis petiolata1
Liparis petiolata2
Liparis cordifolia1

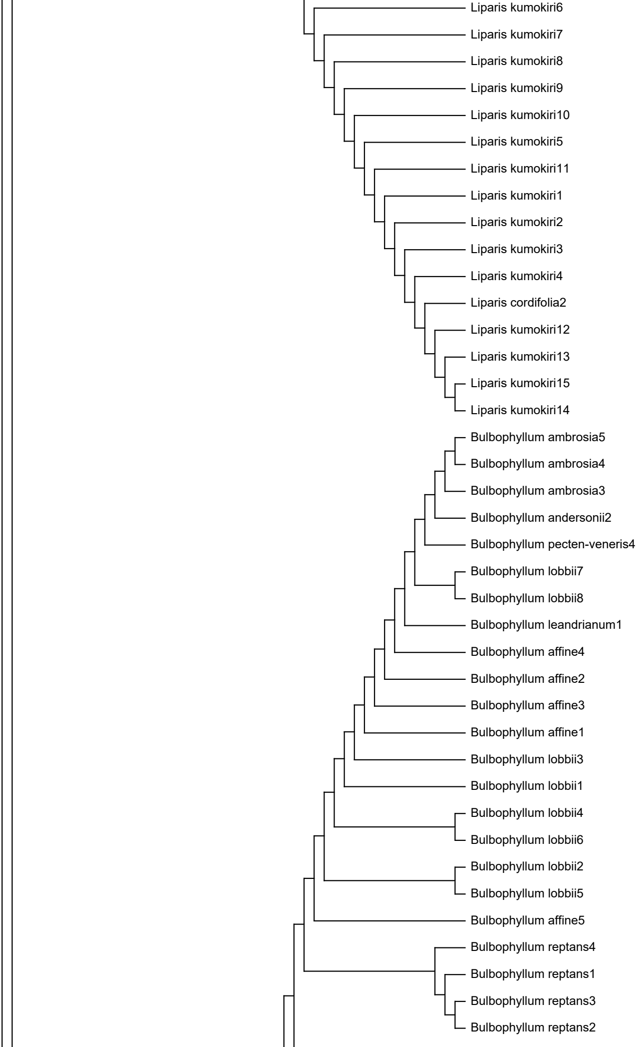
Liparis kumokiri6
Liparis kumokiri7
Liparis kumokiri8
Liparis kumokiri9
Liparis kumokiri10
Liparis kumokiri5
Liparis kumokiri11
Liparis kumokiri1
Liparis kumokiri2
Liparis kumokiri3
Liparis kumokiri4
Liparis cordifolia2
Liparis kumokiri12
Liparis kumokiri13
Liparis kumokiri15
Liparis kumokiri14
Bulbophyllum ambrosia5
Bulbophyllum ambrosia4
Bulbophyllum ambrosia3
Bulbophyllum andersonii2
Bulbophyllum pecten-veneris4
Bulbophyllum lobbii7
Bulbophyllum lobbii8
Bulbophyllum leandrianum1
Bulbophyllum affine4
Bulbophyllum affine2
Bulbophyllum affine3
Bulbophyllum affine1
Bulbophyllum lobbii3
Bulbophyllum lobbii1
Bulbophyllum lobbii4
Bulbophyllum lobbii6
Bulbophyllum lobbii2
Bulbophyllum lobbii5
Bulbophyllum affine5
Bulbophyllum reptans4
Bulbophyllum reptans1
Bulbophyllum reptans3
Bulbophyllum reptans2

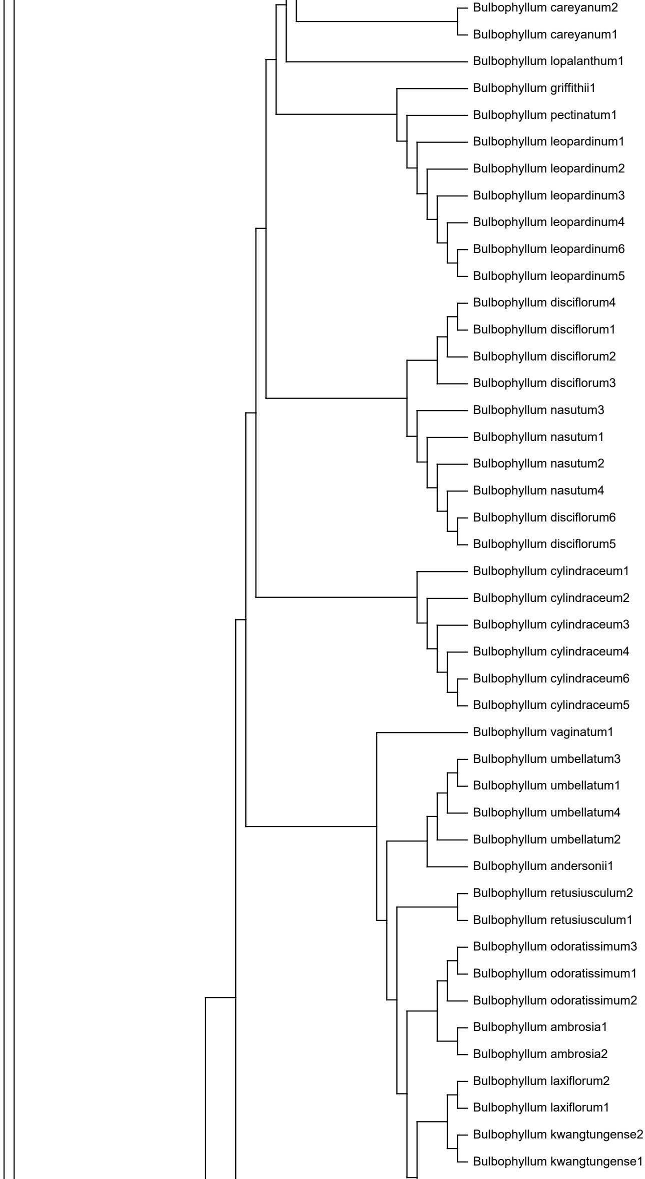
Bulbophyllum careyanum2
Bulbophyllum careyanum1
Bulbophyllum lopalanthum1
Bulbophyllum griffithii1
Bulbophyllum pectinatum1
Bulbophyllum leopardinum1
Bulbophyllum leopardinum2
Bulbophyllum leopardinum3
Bulbophyllum leopardinum4
Bulbophyllum leopardinum6
Bulbophyllum leopardinum5
Bulbophyllum disciflorum4
Bulbophyllum disciflorum1
Bulbophyllum disciflorum2
Bulbophyllum disciflorum3
Bulbophyllum nasutum3
Bulbophyllum nasutum1
Bulbophyllum nasutum2
Bulbophyllum nasutum4
Bulbophyllum disciflorum6
Bulbophyllum disciflorum5
Bulbophyllum cylindraceum1
Bulbophyllum cylindraceum2
Bulbophyllum cylindraceum3
Bulbophyllum cylindraceum4
Bulbophyllum cylindraceum6
Bulbophyllum cylindraceum5
Bulbophyllum vaginatum1
Bulbophyllum umbellatum3
Bulbophyllum umbellatum1
Bulbophyllum umbellatum4
Bulbophyllum umbellatum2
Bulbophyllum andersonii1
Bulbophyllum retusiusculum2
Bulbophyllum retusiusculum1
Bulbophyllum odoratissimum3
Bulbophyllum odoratissimum1
Bulbophyllum odoratissimum2
Bulbophyllum ambrosia1
Bulbophyllum ambrosia2
Bulbophyllum laxiflorum2
Bulbophyllum laxiflorum1
Bulbophyllum kwangtungense2
Bulbophyllum kwangtungense1

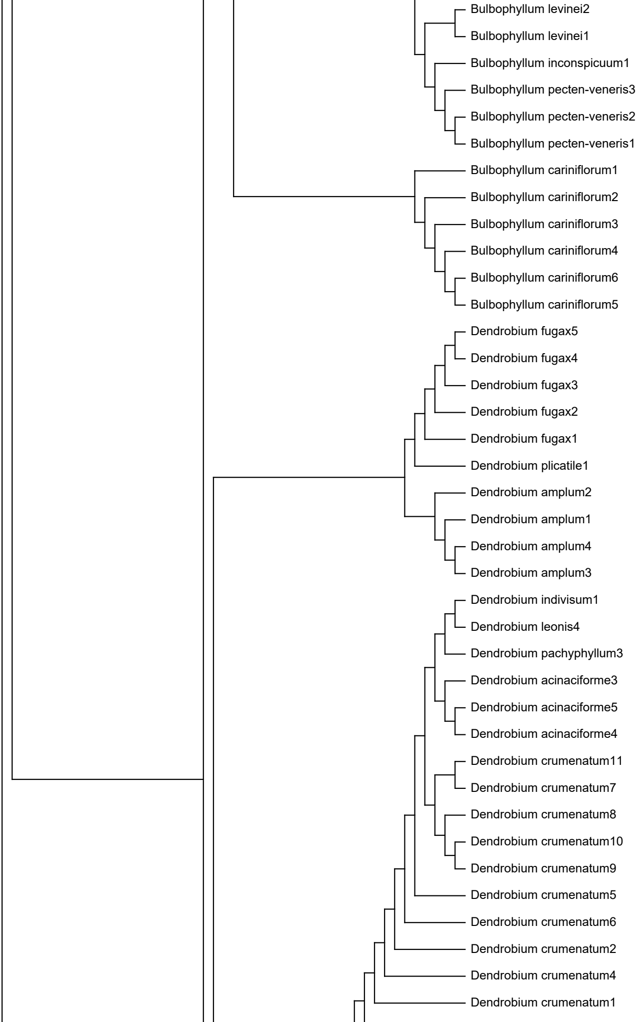
Bulbophyllum levinei2
Bulbophyllum levinei1
Bulbophyllum inconspicuum1
Bulbophyllum pecten-veneris3
Bulbophyllum pecten-veneris2
Bulbophyllum pecten-veneris1
Bulbophyllum cariniflorum1
Bulbophyllum cariniflorum2
Bulbophyllum cariniflorum3
Bulbophyllum cariniflorum4
Bulbophyllum cariniflorum6
Bulbophyllum cariniflorum5
Dendrobium fugax5
Dendrobium fugax4
Dendrobium fugax3
Dendrobium fugax2
Dendrobium fugax1
Dendrobium plicatile1
Dendrobium amplum2
Dendrobium amplum1
Dendrobium amplum4
Dendrobium amplum3
Dendrobium indivisum1
Dendrobium leonis4
Dendrobium pachyphyllum3
Dendrobium acinaciforme3
Dendrobium acinaciforme5
Dendrobium acinaciforme4
Dendrobium crumenatum11
Dendrobium crumenatum7
Dendrobium crumenatum8
Dendrobium crumenatum10
Dendrobium crumenatum9
Dendrobium crumenatum5
Dendrobium crumenatum6
Dendrobium crumenatum2
Dendrobium crumenatum4
Dendrobium crumenatum1

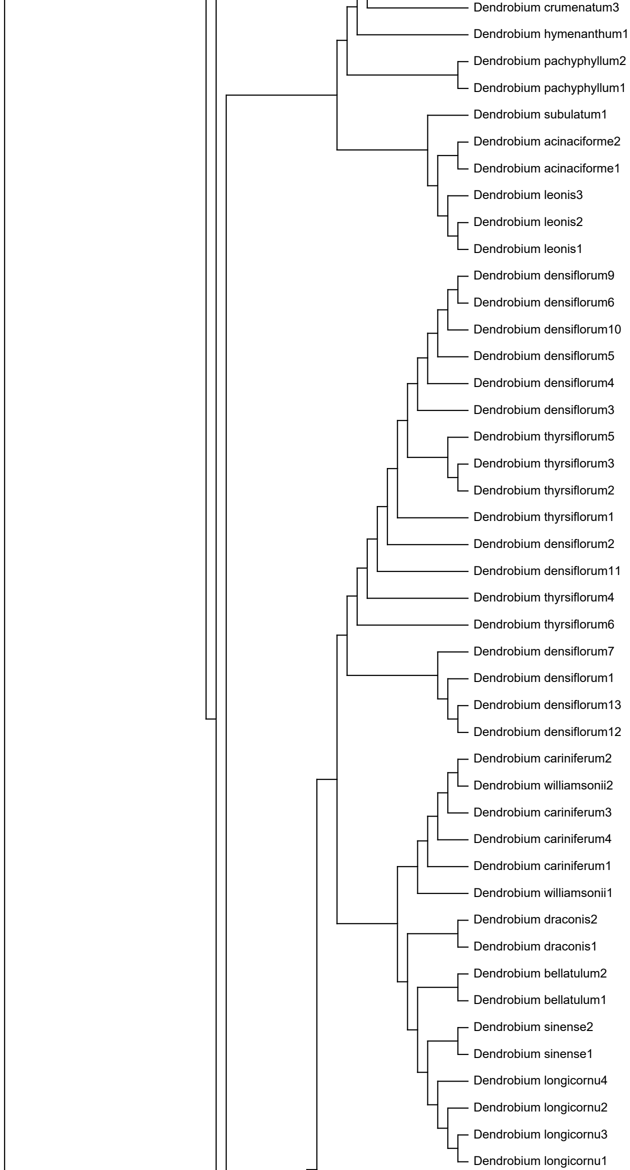
Dendrobium crumenatum3
Dendrobium hymenanthum1
Dendrobium pachyphyllum2
Dendrobium pachyphyllum1
Dendrobium subulatum1
Dendrobium acinaciforme2
Dendrobium acinaciforme1
Dendrobium leonis3
Dendrobium leonis2
Dendrobium leonis1
Dendrobium densiflorum9
Dendrobium densiflorum6
Dendrobium densiflorum10
Dendrobium densiflorum5
Dendrobium densiflorum4
Dendrobium densiflorum3
Dendrobium thyrsiflorum5
Dendrobium thyrsiflorum3
Dendrobium thyrsiflorum2
Dendrobium thyrsiflorum1
Dendrobium densiflorum2
Dendrobium densiflorum11
Dendrobium thyrsiflorum4
Dendrobium thyrsiflorum6
Dendrobium densiflorum7
Dendrobium densiflorum1
Dendrobium densiflorum13
Dendrobium densiflorum12
Dendrobium cariniferum2
Dendrobium williamsonii2
Dendrobium cariniferum3
Dendrobium cariniferum4
Dendrobium cariniferum1
Dendrobium williamsonii1
Dendrobium draconis2
Dendrobium draconis1
Dendrobium bellatulum2
Dendrobium bellatulum1
Dendrobium sinense2
Dendrobium sinense1
Dendrobium longicornu4
Dendrobium longicornu2
Dendrobium longicornu3
Dendrobium longicornu1

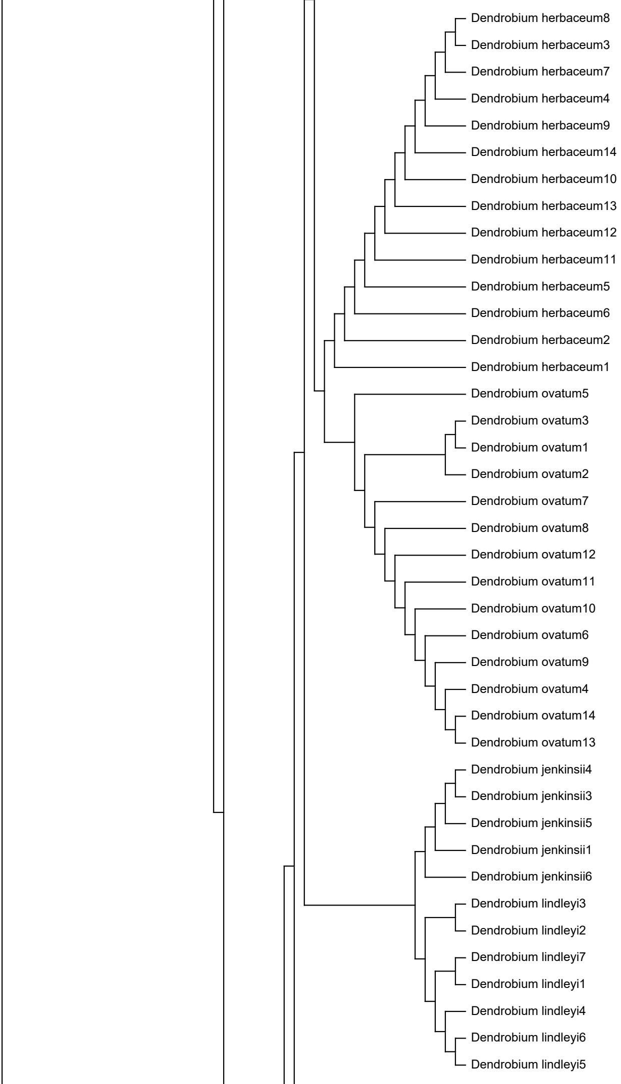
Dendrobium herbaceum8
Dendrobium herbaceum3
Dendrobium herbaceum7
Dendrobium herbaceum4
Dendrobium herbaceum9
Dendrobium herbaceum14
Dendrobium herbaceum10
Dendrobium herbaceum13
Dendrobium herbaceum12
Dendrobium herbaceum11
Dendrobium herbaceum5
Dendrobium herbaceum6
Dendrobium herbaceum2
Dendrobium herbaceum1
Dendrobium ovatum5
Dendrobium ovatum3
Dendrobium ovatum1
Dendrobium ovatum2
Dendrobium ovatum7
Dendrobium ovatum8
Dendrobium ovatum12
Dendrobium ovatum11
Dendrobium ovatum10
Dendrobium ovatum6
Dendrobium ovatum9
Dendrobium ovatum4
Dendrobium ovatum14
Dendrobium ovatum13
Dendrobium jenkinsii4
Dendrobium jenkinsii3
Dendrobium jenkinsii5
Dendrobium jenkinsii1
Dendrobium jenkinsii6
Dendrobium lindleyi3
Dendrobium lindleyi2
Dendrobium lindleyi7
Dendrobium lindleyi1
Dendrobium lindleyi4
Dendrobium lindleyi6
Dendrobium lindleyi5

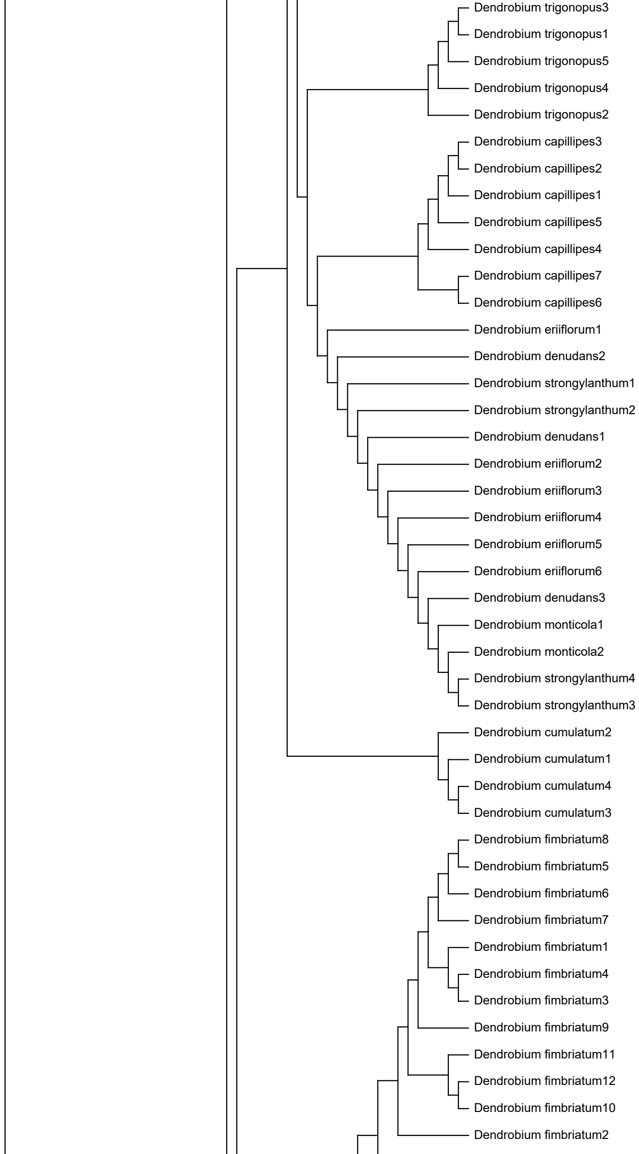

Dendrobium trigonopus3
Dendrobium trigonopus1
Dendrobium trigonopus5
Dendrobium trigonopus4
Dendrobium trigonopus2
Dendrobium capillipes3
Dendrobium capillipes2
Dendrobium capillipes1
Dendrobium capillipes5
Dendrobium capillipes4
Dendrobium capillipes7
Dendrobium capillipes6
Dendrobium eriiflorum1
Dendrobium denudans2
Dendrobium strongylanthum1
Dendrobium strongylanthum2
Dendrobium denudans1
Dendrobium eriiflorum2
Dendrobium eriiflorum3
Dendrobium eriiflorum4
Dendrobium eriiflorum5
Dendrobium eriiflorum6
Dendrobium denudans3
Dendrobium monticola1
Dendrobium monticola2
Dendrobium strongylanthum4
Dendrobium strongylanthum3
Dendrobium cumulatum2
Dendrobium cumulatum1
Dendrobium cumulatum4
Dendrobium cumulatum3
Dendrobium fimbriatum8
Dendrobium fimbriatum5
Dendrobium fimbriatum6
Dendrobium fimbriatum7
Dendrobium fimbriatum1
Dendrobium fimbriatum4
Dendrobium fimbriatum3
Dendrobium fimbriatum9
Dendrobium fimbriatum11
Dendrobium fimbriatum12
Dendrobium fimbriatum10
Dendrobium fimbriatum2

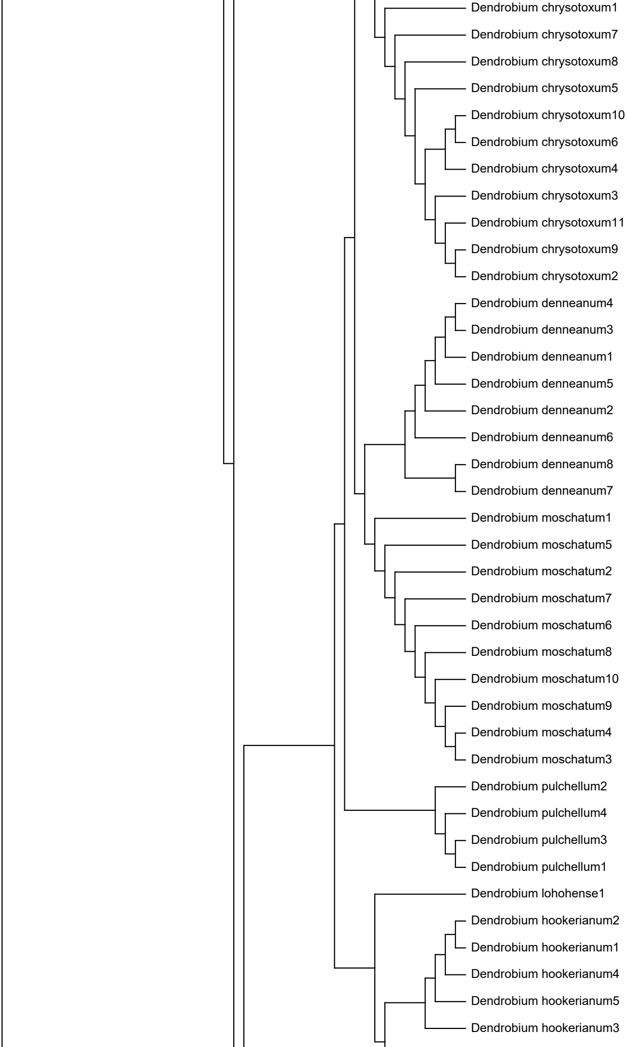
Dendrobium chrysotoxum1
Dendrobium chrysotoxum7
Dendrobium chrysotoxum8
Dendrobium chrysotoxum5
Dendrobium chrysotoxum10
Dendrobium chrysotoxum6
Dendrobium chrysotoxum4
Dendrobium chrysotoxum3
Dendrobium chrysotoxum11
Dendrobium chrysotoxum9
Dendrobium chrysotoxum2
Dendrobium denneanum4
Dendrobium denneanum3
Dendrobium denneanum1
Dendrobium denneanum5
Dendrobium denneanum2
Dendrobium denneanum6
Dendrobium denneanum8
Dendrobium denneanum7
Dendrobium moschatum1
Dendrobium moschatum5
Dendrobium moschatum2
Dendrobium moschatum7
Dendrobium moschatum6
Dendrobium moschatum8
Dendrobium moschatum10
Dendrobium moschatum9
Dendrobium moschatum4
Dendrobium moschatum3
Dendrobium pulchellum2
Dendrobium pulchellum4
Dendrobium pulchellum3
Dendrobium pulchellum1
Dendrobium lohohense1
Dendrobium hookerianum2
Dendrobium hookerianum1
Dendrobium hookerianum4
Dendrobium hookerianum5
Dendrobium hookerianum3

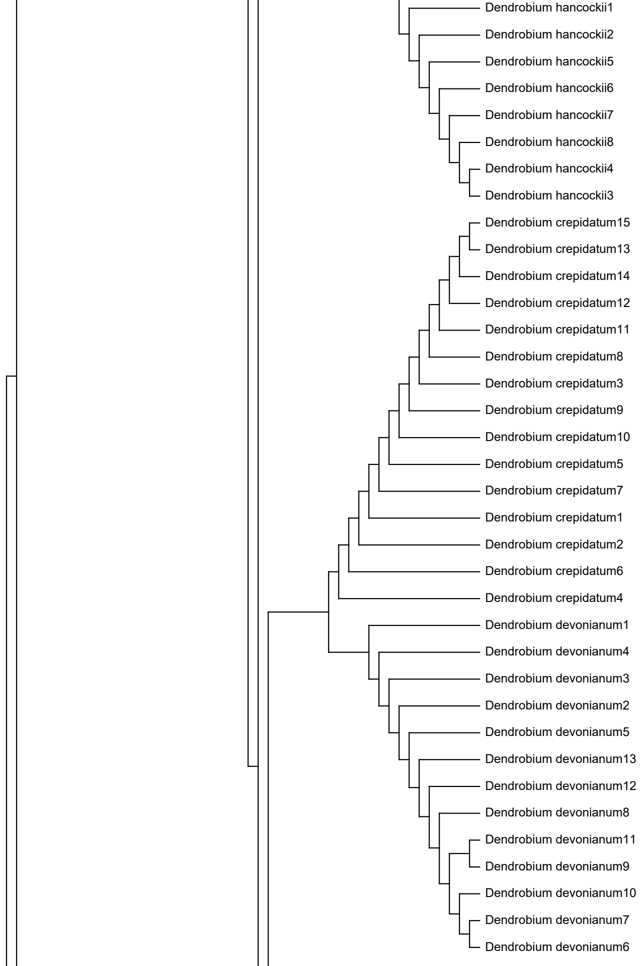
Dendrobium hancockii1
Dendrobium hancockii2
Dendrobium hancockii5
Dendrobium hancockii6
Dendrobium hancockii7
Dendrobium hancockii8
Dendrobium hancockii4
Dendrobium hancockii3
Dendrobium crepidatum15
Dendrobium crepidatum13
Dendrobium crepidatum14
Dendrobium crepidatum12
Dendrobium crepidatum11
Dendrobium crepidatum8
Dendrobium crepidatum3
Dendrobium crepidatum9
Dendrobium crepidatum10
Dendrobium crepidatum5
Dendrobium crepidatum7
Dendrobium crepidatum1
Dendrobium crepidatum2
Dendrobium crepidatum6
Dendrobium crepidatum4
Dendrobium devonianum1
Dendrobium devonianum4
Dendrobium devonianum3
Dendrobium devonianum2
Dendrobium devonianum5
Dendrobium devonianum13
Dendrobium devonianum12
Dendrobium devonianum8
Dendrobium devonianum11
Dendrobium devonianum9
Dendrobium devonianum10
Dendrobium devonianum7
Dendrobium devonianum6

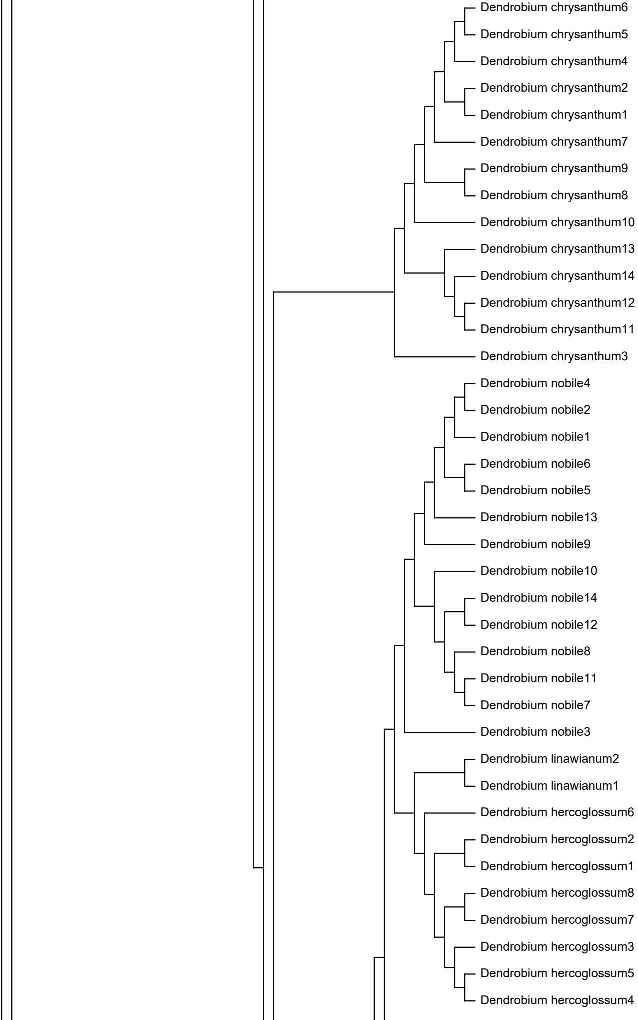
Dendrobium chrysanthum6
Dendrobium chrysanthum5
Dendrobium chrysanthum4
Dendrobium chrysanthum2
Dendrobium chrysanthum1
Dendrobium chrysanthum7
Dendrobium chrysanthum9
Dendrobium chrysanthum8
Dendrobium chrysanthum10
Dendrobium chrysanthum13
Dendrobium chrysanthum14
Dendrobium chrysanthum12
Dendrobium chrysanthum11
Dendrobium chrysanthum3
Dendrobium nobile4
Dendrobium nobile2
Dendrobium nobile1
Dendrobium nobile6
Dendrobium nobile5
Dendrobium nobile13
Dendrobium nobile9
Dendrobium nobile10
Dendrobium nobile14
Dendrobium nobile12
Dendrobium nobile8
Dendrobium nobile11
Dendrobium nobile7
Dendrobium nobile3
Dendrobium linawianum2
Dendrobium linawianum1
Dendrobium hercoglossum6
Dendrobium hercoglossum2
Dendrobium hercoglossum1
Dendrobium hercoglossum8
Dendrobium hercoglossum7
Dendrobium hercoglossum3
Dendrobium hercoglossum5
Dendrobium hercoglossum4

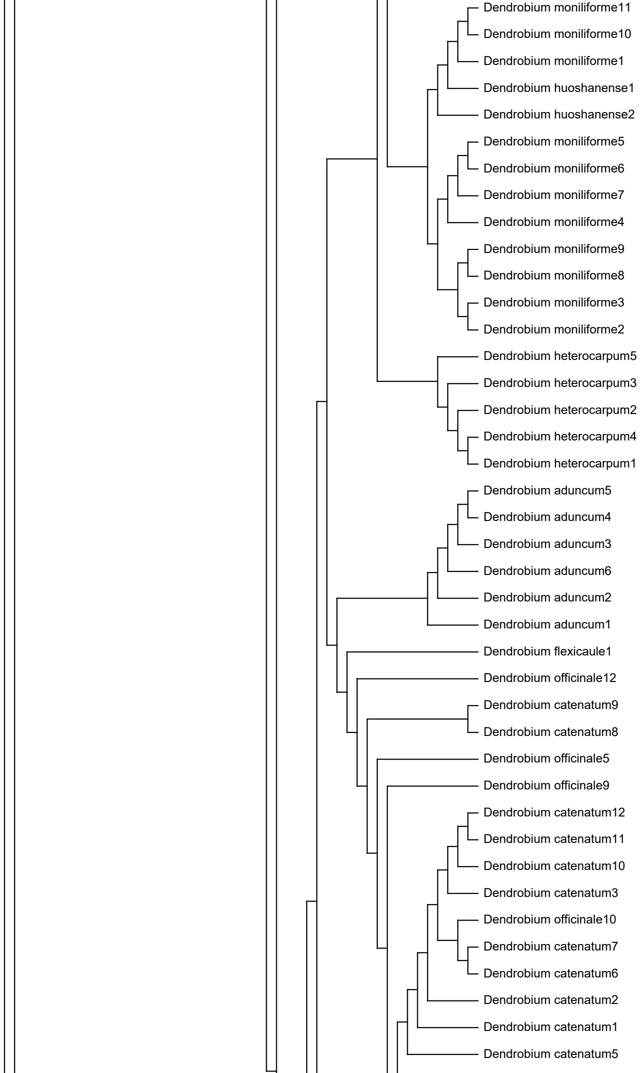
Dendrobium moniliforme11
Dendrobium moniliforme10
Dendrobium moniliforme1
Dendrobium huoshanense1
Dendrobium huoshanense2
Dendrobium moniliforme5
Dendrobium moniliforme6
Dendrobium moniliforme7
Dendrobium moniliforme4
Dendrobium moniliforme9
Dendrobium moniliforme8
Dendrobium moniliforme3
Dendrobium moniliforme2
Dendrobium heterocarpum5
Dendrobium heterocarpum3
Dendrobium heterocarpum2
Dendrobium heterocarpum4
Dendrobium heterocarpum1
Dendrobium aduncum5
Dendrobium aduncum4
Dendrobium aduncum3
Dendrobium aduncum6
Dendrobium aduncum2
Dendrobium aduncum1
Dendrobium flexicaule1
Dendrobium officinale12
Dendrobium catenatum9
Dendrobium catenatum8
Dendrobium officinale5
Dendrobium officinale9
Dendrobium catenatum12
Dendrobium catenatum11
Dendrobium catenatum10
Dendrobium catenatum3
Dendrobium officinale10
Dendrobium catenatum7
Dendrobium catenatum6
Dendrobium catenatum2
Dendrobium catenatum1
Dendrobium catenatum5

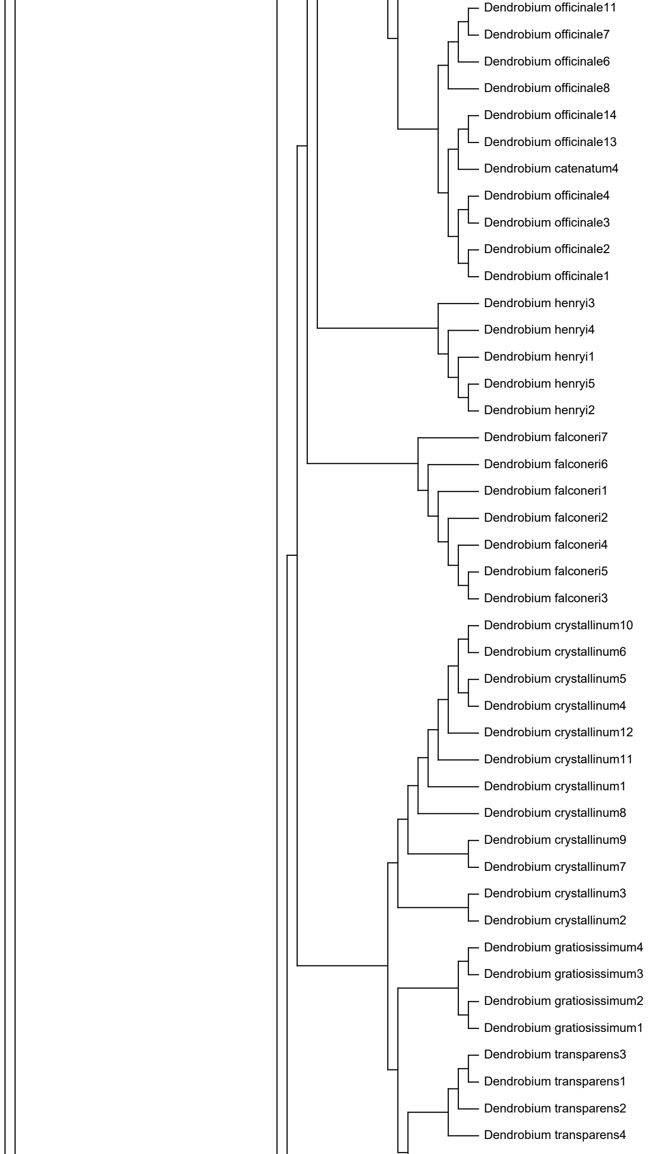
Dendrobium officinale11
Dendrobium officinale7
Dendrobium officinale6
Dendrobium officinale8
Dendrobium officinale14
Dendrobium officinale13
Dendrobium catenatum4
Dendrobium officinale4
Dendrobium officinale3
Dendrobium officinale2
Dendrobium officinale1
Dendrobium henryi3
Dendrobium henryi4
Dendrobium henryi1
Dendrobium henryi5
Dendrobium henryi2
Dendrobium falconeri7
Dendrobium falconeri6
Dendrobium falconeri1
Dendrobium falconeri2
Dendrobium falconeri4
Dendrobium falconeri5
Dendrobium falconeri3
Dendrobium crystallinum10
Dendrobium crystallinum6
Dendrobium crystallinum5
Dendrobium crystallinum4
Dendrobium crystallinum12
Dendrobium crystallinum11
Dendrobium crystallinum1
Dendrobium crystallinum8
Dendrobium crystallinum9
Dendrobium crystallinum7
Dendrobium crystallinum3
Dendrobium crystallinum2
Dendrobium gratiosissimum4
Dendrobium gratiosissimum3
Dendrobium gratiosissimum2
Dendrobium gratiosissimum1
Dendrobium transparens3
Dendrobium transparens1
Dendrobium transparens2
Dendrobium transparens4

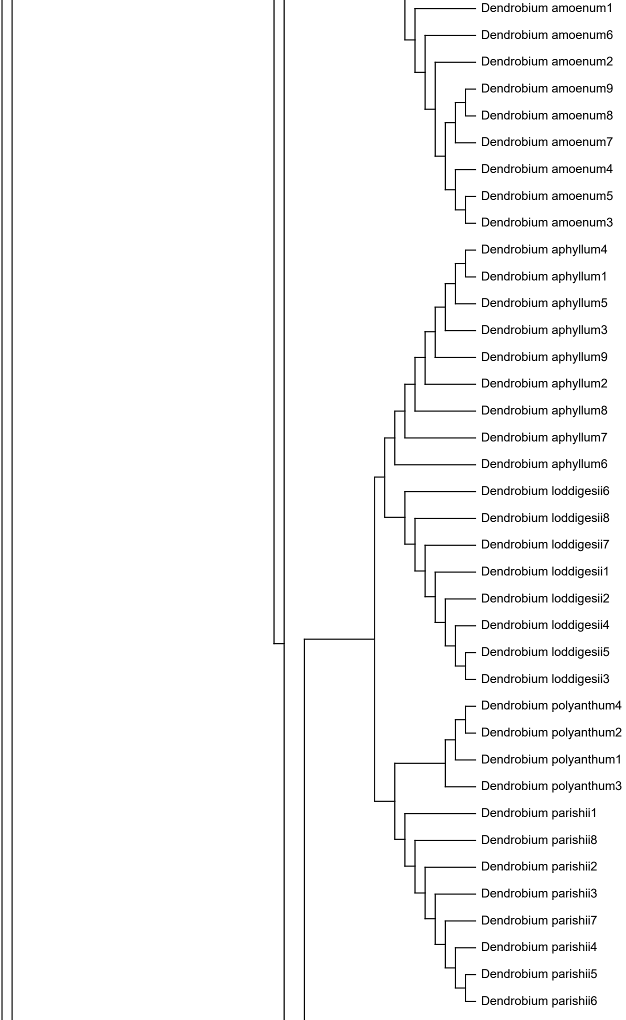
Dendrobium amoenum1
Dendrobium amoenum6
Dendrobium amoenum2
Dendrobium amoenum9
Dendrobium amoenum8
Dendrobium amoenum7
Dendrobium amoenum4
Dendrobium amoenum5
Dendrobium amoenum3
Dendrobium aphyllum4
Dendrobium aphyllum1
Dendrobium aphyllum5
Dendrobium aphyllum3
Dendrobium aphyllum9
Dendrobium aphyllum2
Dendrobium aphyllum8
Dendrobium aphyllum7
Dendrobium aphyllum6
Dendrobium loddigesii6
Dendrobium loddigesii8
Dendrobium loddigesii7
Dendrobium loddigesii1
Dendrobium loddigesii2
Dendrobium loddigesii4
Dendrobium loddigesii5
Dendrobium loddigesii3
Dendrobium polyanthum4
Dendrobium polyanthum2
Dendrobium polyanthum1
Dendrobium polyanthum3
Dendrobium parishii1
Dendrobium parishii8
Dendrobium parishii2
Dendrobium parishii3
Dendrobium parishii7
Dendrobium parishii4
Dendrobium parishii5
Dendrobium parishii6

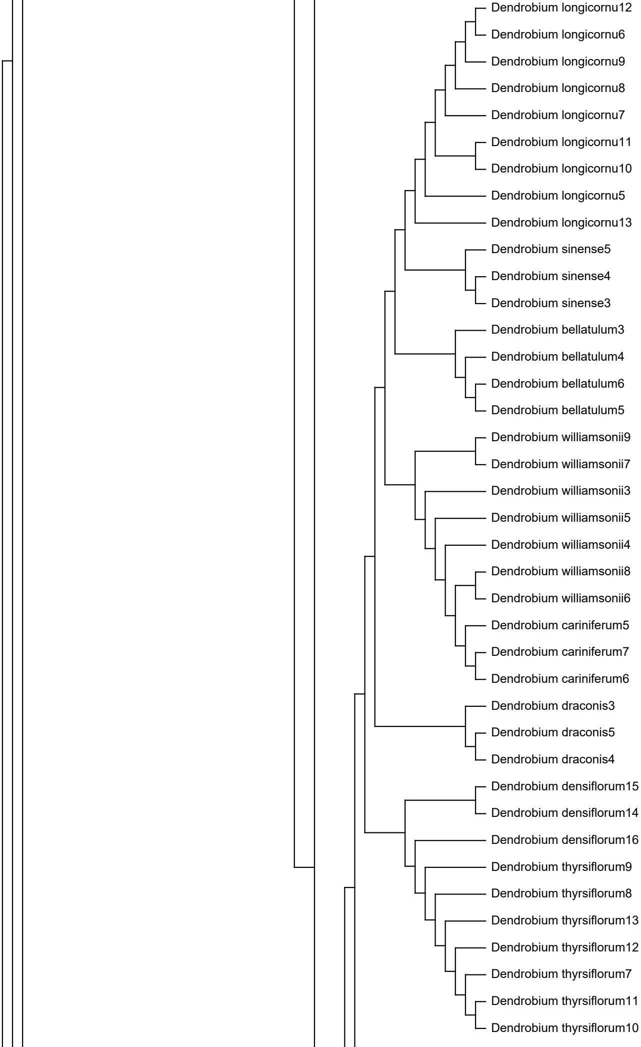

Dendrobium longicornu12
Dendrobium longicornu6
Dendrobium longicornu9
Dendrobium longicornu8
Dendrobium longicornu7
Dendrobium longicornu11
Dendrobium longicornu10
Dendrobium longicornu5
Dendrobium longicornu13
Dendrobium sinense5
Dendrobium sinense4
Dendrobium sinense3
Dendrobium bellatulum3
Dendrobium bellatulum4
Dendrobium bellatulum6
Dendrobium bellatulum5
Dendrobium williamsonii9
Dendrobium williamsonii7
Dendrobium williamsonii3
Dendrobium williamsonii5
Dendrobium williamsonii4
Dendrobium williamsonii8
Dendrobium williamsonii6
Dendrobium cariniferum5
Dendrobium cariniferum7
Dendrobium cariniferum6
Dendrobium draconis3
Dendrobium draconis5
Dendrobium draconis4
Dendrobium densiflorum15
Dendrobium densiflorum14
Dendrobium densiflorum16
Dendrobium thyrsiflorum9
Dendrobium thyrsiflorum8
Dendrobium thyrsiflorum13
Dendrobium thyrsiflorum12
Dendrobium thyrsiflorum7
Dendrobium thyrsiflorum11
Dendrobium thyrsiflorum10

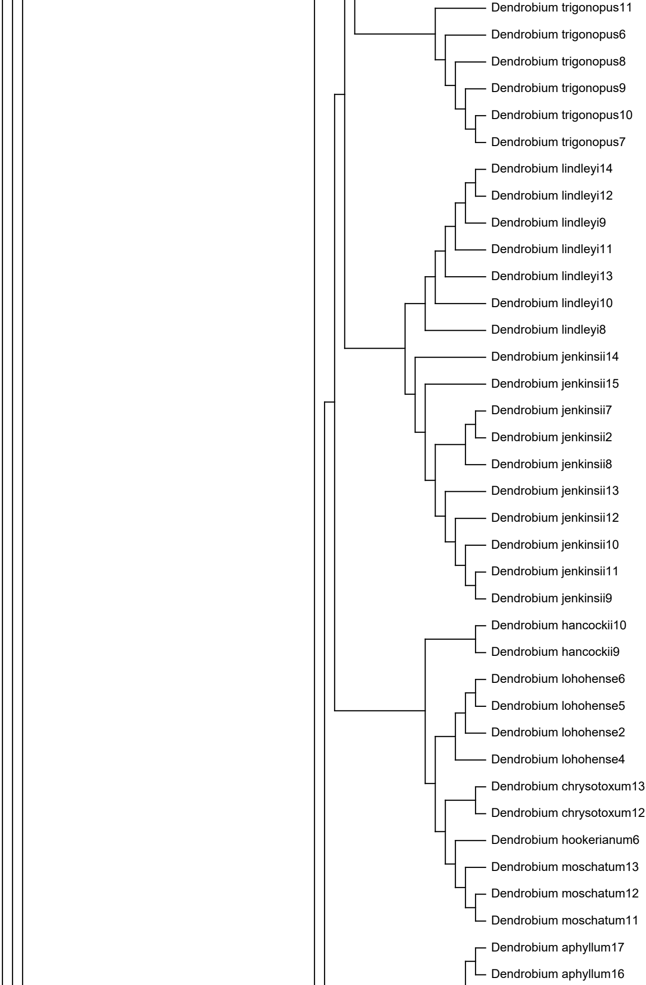
Dendrobium trigonopus11
Dendrobium trigonopus6
Dendrobium trigonopus8
Dendrobium trigonopus9
Dendrobium trigonopus10
Dendrobium trigonopus7
Dendrobium lindleyi14
Dendrobium lindleyi12
Dendrobium lindleyi9
Dendrobium lindleyi11
Dendrobium lindleyi13
Dendrobium lindleyi10
Dendrobium lindleyi8
Dendrobium jenkinsii14
Dendrobium jenkinsii15
Dendrobium jenkinsii7
Dendrobium jenkinsii2
Dendrobium jenkinsii8
Dendrobium jenkinsii13
Dendrobium jenkinsii12
Dendrobium jenkinsii10
Dendrobium jenkinsii11
Dendrobium jenkinsii9
Dendrobium hancockii10
Dendrobium hancockii9
Dendrobium lohohense6
Dendrobium lohohense5
Dendrobium lohohense2
Dendrobium lohohense4
Dendrobium chrysotoxum13
Dendrobium chrysotoxum12
Dendrobium hookerianum6
Dendrobium moschatum13
Dendrobium moschatum12
Dendrobium moschatum11
Dendrobium aphyllum17
Dendrobium aphyllum16

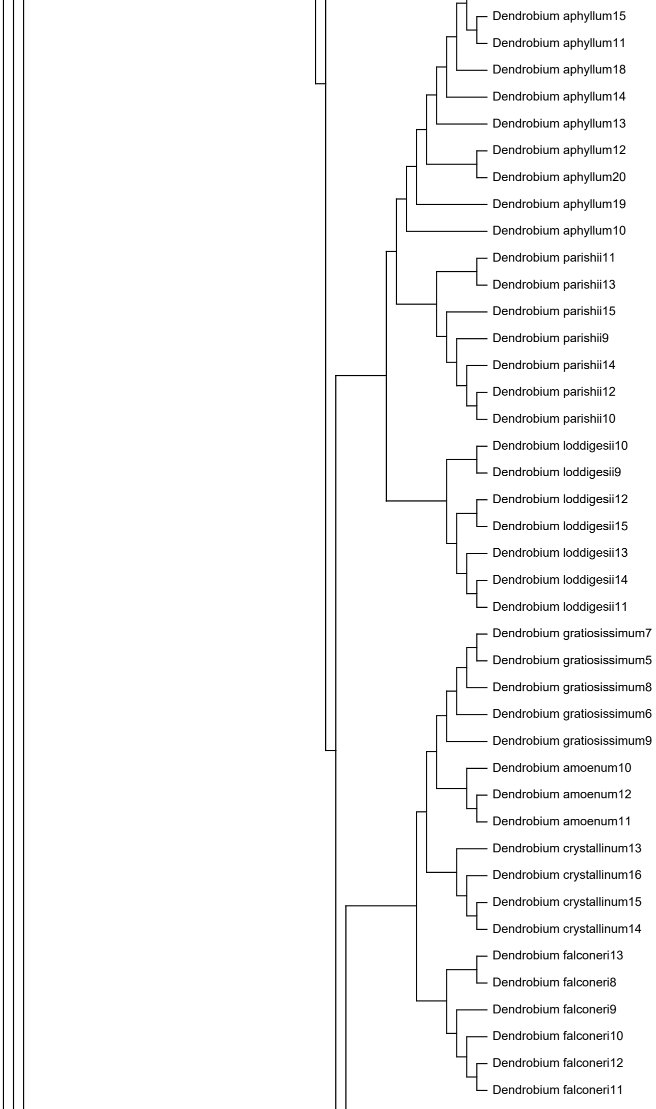
Dendrobium aphyllum15
Dendrobium aphyllum11
Dendrobium aphyllum18
Dendrobium aphyllum14
Dendrobium aphyllum13
Dendrobium aphyllum12
Dendrobium aphyllum20
Dendrobium aphyllum19
Dendrobium aphyllum10
Dendrobium parishii11
Dendrobium parishii13
Dendrobium parishii15
Dendrobium parishii9
Dendrobium parishii14
Dendrobium parishii12
Dendrobium parishii10
Dendrobium loddigesii10
Dendrobium loddigesii9
Dendrobium loddigesii12
Dendrobium loddigesii15
Dendrobium loddigesii13
Dendrobium loddigesii14
Dendrobium loddigesii11
Dendrobium gratiosissimum7
Dendrobium gratiosissimum5
Dendrobium gratiosissimum8
Dendrobium gratiosissimum6
Dendrobium gratiosissimum9
Dendrobium amoenum10
Dendrobium amoenum12
Dendrobium amoenum11
Dendrobium crystallinum13
Dendrobium crystallinum16
Dendrobium crystallinum15
Dendrobium crystallinum14
Dendrobium falconeri13
Dendrobium falconeri8
Dendrobium falconeri9
Dendrobium falconeri10
Dendrobium falconeri12
Dendrobium falconeri11

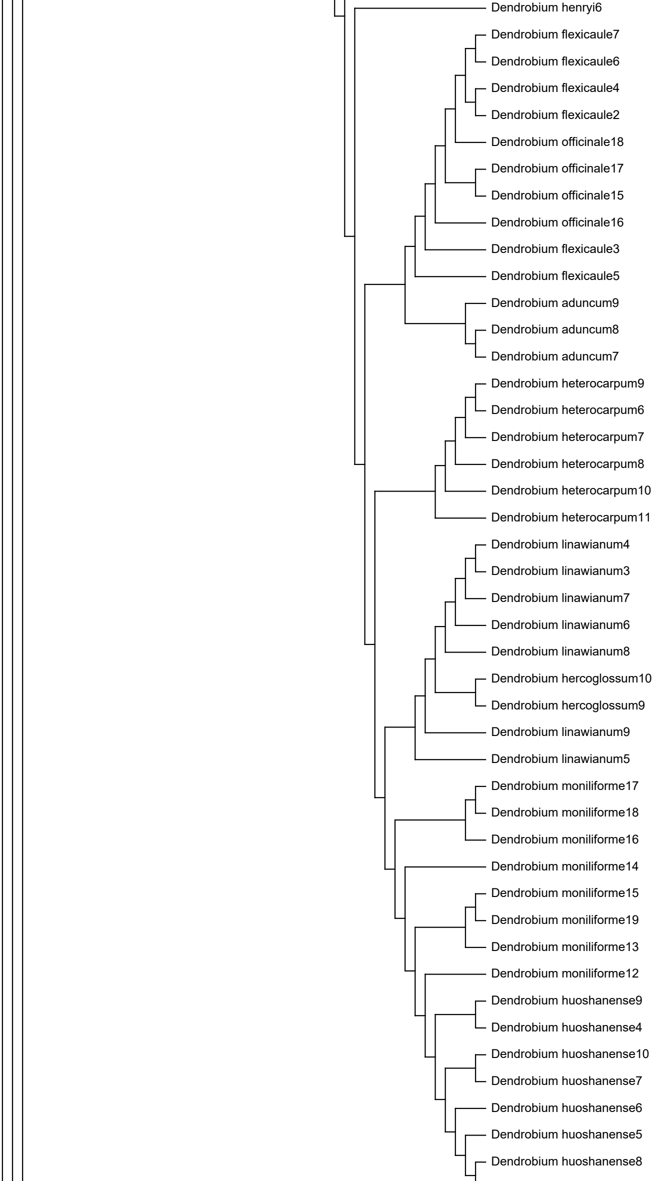
Dendrobium henryi6
Dendrobium flexicaule7
Dendrobium flexicaule6
Dendrobium flexicaule4
Dendrobium flexicaule2
Dendrobium officinale18
Dendrobium officinale17
Dendrobium officinale15
Dendrobium officinale16
Dendrobium flexicaule3
Dendrobium flexicaule5
Dendrobium aduncum9
Dendrobium aduncum8
Dendrobium aduncum7
Dendrobium heterocarpum9
Dendrobium heterocarpum6
Dendrobium heterocarpum7
Dendrobium heterocarpum8
Dendrobium heterocarpum10
Dendrobium heterocarpum11
Dendrobium linawianum4
Dendrobium linawianum3
Dendrobium linawianum7
Dendrobium linawianum6
Dendrobium linawianum8
Dendrobium hercoglossum10
Dendrobium hercoglossum9
Dendrobium linawianum9
Dendrobium linawianum5
Dendrobium moniliforme17
Dendrobium moniliforme18
Dendrobium moniliforme16
Dendrobium moniliforme14
Dendrobium moniliforme15
Dendrobium moniliforme19
Dendrobium moniliforme13
Dendrobium moniliforme12
Dendrobium huoshanense9
Dendrobium huoshanense4
Dendrobium huoshanense10
Dendrobium huoshanense7
Dendrobium huoshanense6
Dendrobium huoshanense5
Dendrobium huoshanense8

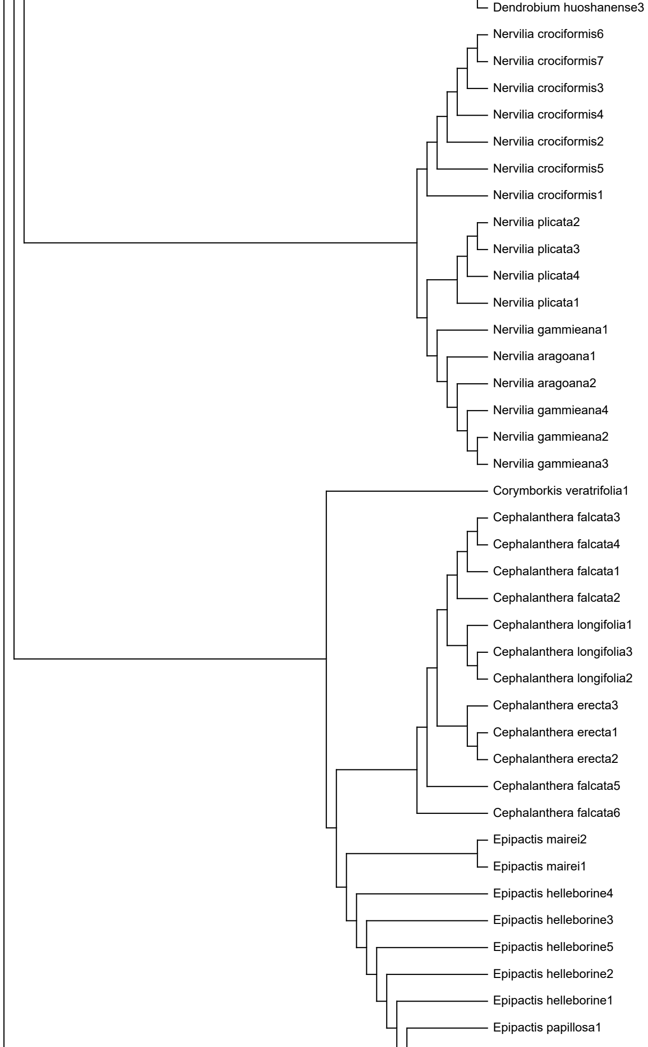
Dendrobium huoshanense3
Nervilia crociformis6
Nervilia crociformis7
Nervilia crociformis3
Nervilia crociformis4
Nervilia crociformis2
Nervilia crociformis5
Nervilia crociformis1
Nervilia plicata2
Nervilia plicata3
Nervilia plicata4
Nervilia plicata1
Nervilia gammieana1
Nervilia aragoana1
Nervilia aragoana2
Nervilia gammieana4
Nervilia gammieana2
Nervilia gammieana3
Corymborkis veratrifolia1
Cephalanthera falcata3
Cephalanthera falcata4
Cephalanthera falcata1
Cephalanthera falcata2
Cephalanthera longifolia1
Cephalanthera longifolia3
Cephalanthera longifolia2
Cephalanthera erecta3
Cephalanthera erecta1
Cephalanthera erecta2
Cephalanthera falcata5
Cephalanthera falcata6
Epipactis mairei2
Epipactis mairei1
Epipactis helleborine4
Epipactis helleborine3
Epipactis helleborine5
Epipactis helleborine2
Epipactis helleborine1
Epipactis papillosa1

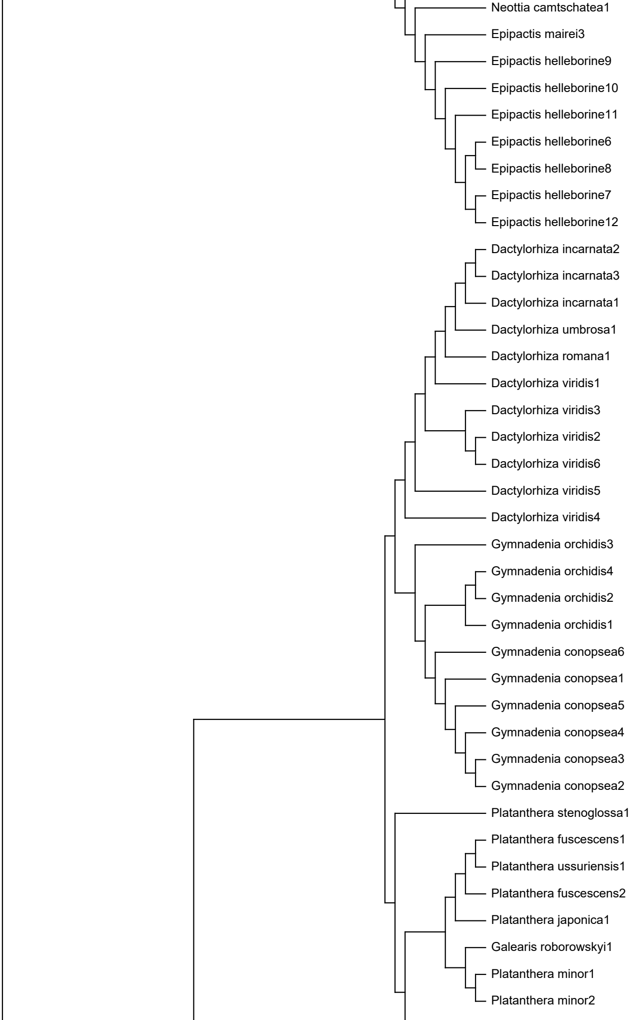
Neottia camtschatea1
Epipactis mairei3
Epipactis helleborine9
Epipactis helleborine10
Epipactis helleborine11
Epipactis helleborine6
Epipactis helleborine8
Epipactis helleborine7
Epipactis helleborine12
Dactylorhiza incarnata2
Dactylorhiza incarnata3
Dactylorhiza incarnata1
Dactylorhiza umbrosa1
Dactylorhiza romana1
Dactylorhiza viridis1
Dactylorhiza viridis3
Dactylorhiza viridis2
Dactylorhiza viridis6
Dactylorhiza viridis5
Dactylorhiza viridis4
Gymnadenia orchidis3
Gymnadenia orchidis4
Gymnadenia orchidis2
Gymnadenia orchidis1
Gymnadenia conopsea6
Gymnadenia conopsea1
Gymnadenia conopsea5
Gymnadenia conopsea4
Gymnadenia conopsea3
Gymnadenia conopsea2
Platanthera stenoglossa1
Platanthera fuscescens1
Platanthera ussuriensis1
Platanthera fuscescens2
Platanthera japonica1
Galearis roborowskyi1
Platanthera minor1
Platanthera minor2

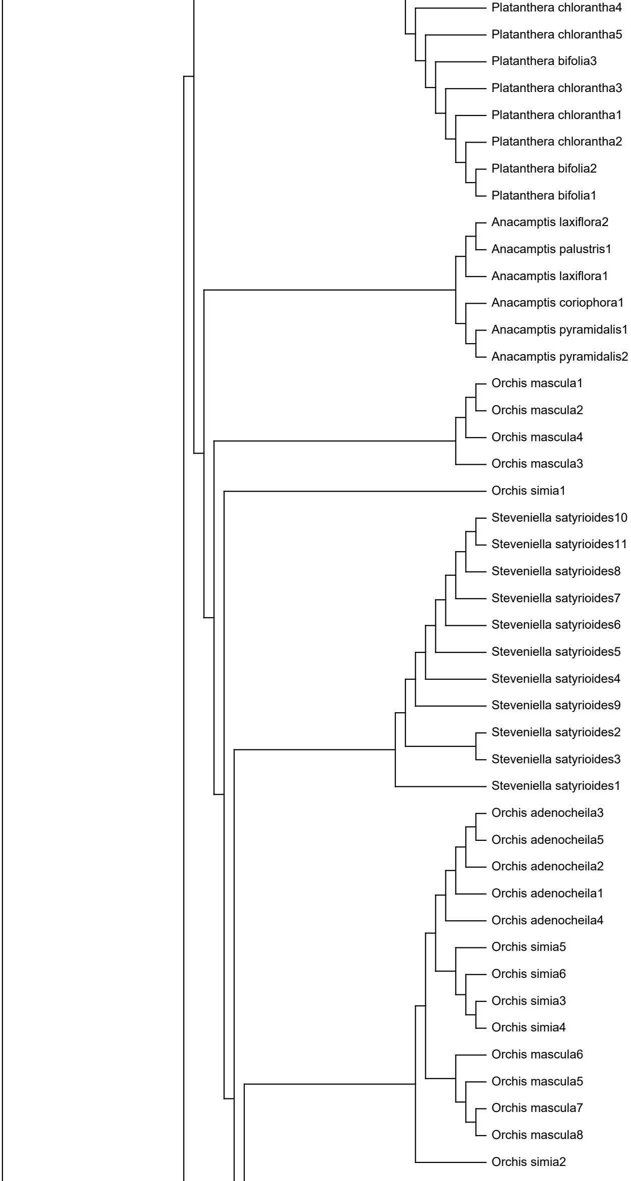

Platanthera chlorantha4
Platanthera chlorantha5
Platanthera bifolia3
Platanthera chlorantha3
Platanthera chlorantha1
Platanthera chlorantha2
Platanthera bifolia2
Platanthera bifolia1
Anacamptis laxiflora2
Anacamptis palustris1
Anacamptis laxiflora1
Anacamptis coriophora1
Anacamptis pyramidalis1
Anacamptis pyramidalis2
Orchis mascula1
Orchis mascula2
Orchis mascula4
Orchis mascula3
Orchis simia1
Steveniella satyrioides10
Steveniella satyrioides11
Steveniella satyrioides8
Steveniella satyrioides7
Steveniella satyrioides6
Steveniella satyrioides5
Steveniella satyrioides4
Steveniella satyrioides9
Steveniella satyrioides2
Steveniella satyrioides3
Steveniella satyrioides1
Orchis adenocheila3
Orchis adenocheila5
Orchis adenocheila2
Orchis adenocheila1
Orchis adenocheila4
Orchis simia5
Orchis simia6
Orchis simia3
Orchis simia4
Orchis mascula6
Orchis mascula5
Orchis mascula7
Orchis mascula8
Orchis simia2

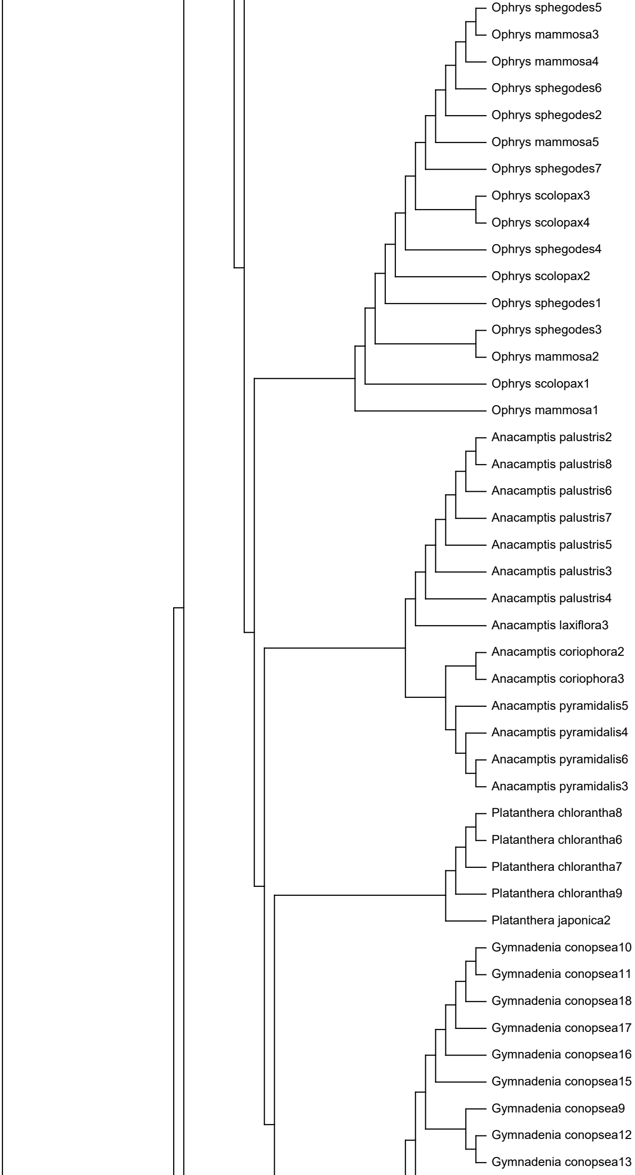
Ophrys sphegodes5
Ophrys mammosa3
Ophrys mammosa4
Ophrys sphegodes6
Ophrys sphegodes2
Ophrys mammosa5
Ophrys sphegodes7
Ophrys scolopax3
Ophrys scolopax4
Ophrys sphegodes4
Ophrys scolopax2
Ophrys sphegodes1
Ophrys sphegodes3
Ophrys mammosa2
Ophrys scolopax1
Ophrys mammosa1
Anacamptis palustris2
Anacamptis palustris8
Anacamptis palustris6
Anacamptis palustris7
Anacamptis palustris5
Anacamptis palustris3
Anacamptis palustris4
Anacamptis laxiflora3
Anacamptis coriophora2
Anacamptis coriophora3
Anacamptis pyramidalis5
Anacamptis pyramidalis4
Anacamptis pyramidalis6
Anacamptis pyramidalis3
Platanthera chlorantha8
Platanthera chlorantha6
Platanthera chlorantha7
Platanthera chlorantha9
Platanthera japonica2
Gymnadenia conopsea10
Gymnadenia conopsea11
Gymnadenia conopsea18
Gymnadenia conopsea17
Gymnadenia conopsea16
Gymnadenia conopsea15
Gymnadenia conopsea9
Gymnadenia conopsea12
Gymnadenia conopsea13

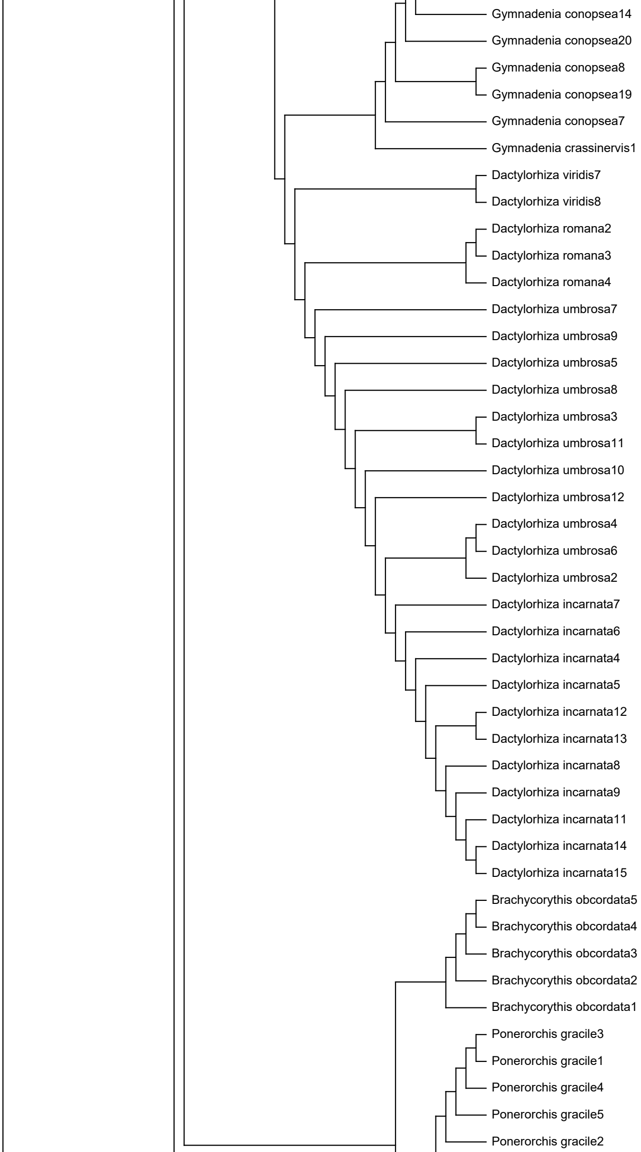
Gymnadenia conopsea14
Gymnadenia conopsea20
Gymnadenia conopsea8
Gymnadenia conopsea19
Gymnadenia conopsea7
Gymnadenia crassinervis1
Dactylorhiza viridis7
Dactylorhiza viridis8
Dactylorhiza romana2
Dactylorhiza romana3
Dactylorhiza romana4
Dactylorhiza umbrosa7
Dactylorhiza umbrosa9
Dactylorhiza umbrosa5
Dactylorhiza umbrosa8
Dactylorhiza umbrosa3
Dactylorhiza umbrosa11
Dactylorhiza umbrosa10
Dactylorhiza umbrosa12
Dactylorhiza umbrosa4
Dactylorhiza umbrosa6
Dactylorhiza umbrosa2
Dactylorhiza incarnata7
Dactylorhiza incarnata6
Dactylorhiza incarnata4
Dactylorhiza incarnata5
Dactylorhiza incarnata12
Dactylorhiza incarnata13
Dactylorhiza incarnata8
Dactylorhiza incarnata9
Dactylorhiza incarnata11
Dactylorhiza incarnata14
Dactylorhiza incarnata15
Brachycorythis obcordata5
Brachycorythis obcordata4
Brachycorythis obcordata3
Brachycorythis obcordata2
Brachycorythis obcordata1
Ponerorchis gracile3
Ponerorchis gracile1
Ponerorchis gracile4
Ponerorchis gracile5
Ponerorchis gracile2

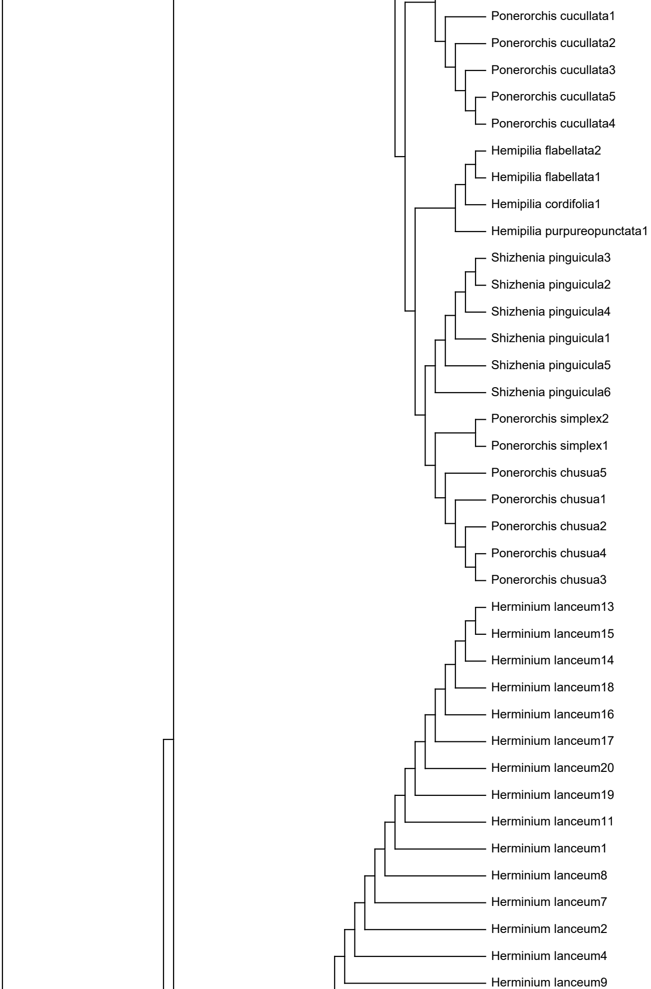
Ponerorchis cucullata1
Ponerorchis cucullata2
Ponerorchis cucullata3
Ponerorchis cucullata5
Ponerorchis cucullata4
Hemipilia flabellata2
Hemipilia flabellata1
Hemipilia cordifolia1
Hemipilia purpureopunctata1
Shizhenia pinguicula3
Shizhenia pinguicula2
Shizhenia pinguicula4
Shizhenia pinguicula1
Shizhenia pinguicula5
Shizhenia pinguicula6
Ponerorchis simplex2
Ponerorchis simplex1
Ponerorchis chusua5
Ponerorchis chusua1
Ponerorchis chusua2
Ponerorchis chusua4
Ponerorchis chusua3
Herminium lanceum13
Herminium lanceum15
Herminium lanceum14
Herminium lanceum18
Herminium lanceum16
Herminium lanceum17
Herminium lanceum20
Herminium lanceum19
Herminium lanceum11
Herminium lanceum1
Herminium lanceum8
Herminium lanceum7
Herminium lanceum2
Herminium lanceum4
Herminium lanceum9

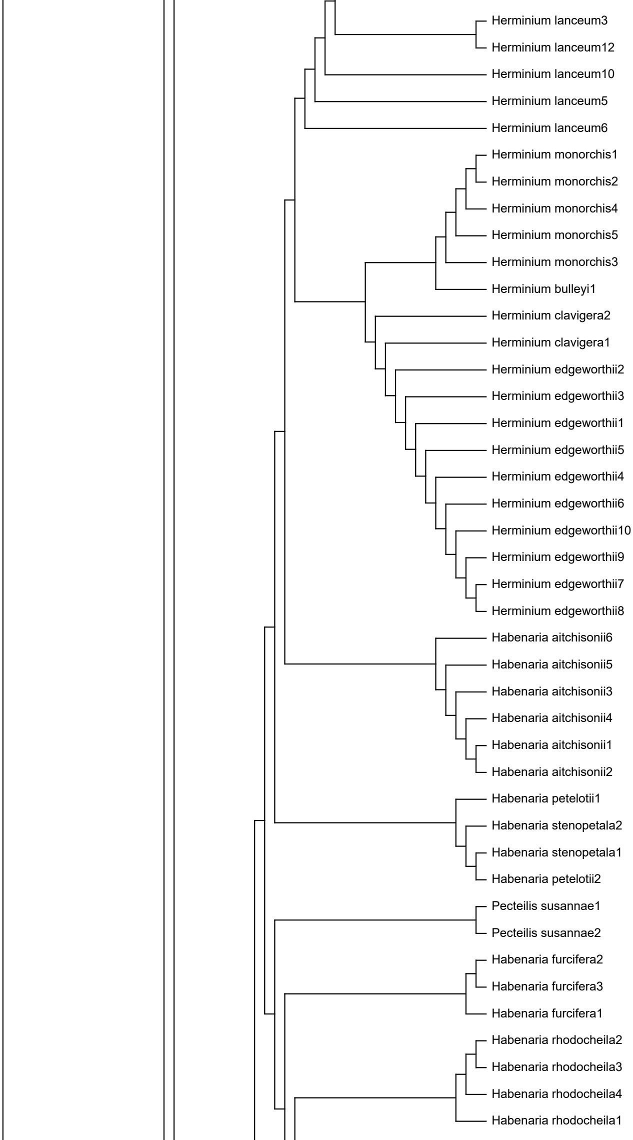
Herminium lanceum3
Herminium lanceum12
Herminium lanceum10
Herminium lanceum5
Herminium lanceum6
Herminium monorchis1
Herminium monorchis2
Herminium monorchis4
Herminium monorchis5
Herminium monorchis3
Herminium bulleyi1
Herminium clavigera2
Herminium clavigera1
Herminium edgeworthii2
Herminium edgeworthii3
Herminium edgeworthii1
Herminium edgeworthii5
Herminium edgeworthii4
Herminium edgeworthii6
Herminium edgeworthii10
Herminium edgeworthii9
Herminium edgeworthii7
Herminium edgeworthii8
Habenaria aitchisonii6
Habenaria aitchisonii5
Habenaria aitchisonii3
Habenaria aitchisonii4
Habenaria aitchisonii1
Habenaria aitchisonii2
Habenaria petelotii1
Habenaria stenopetala2
Habenaria stenopetala1
Habenaria petelotii2
Pecteilis susannae1
Pecteilis susannae2
Habenaria furcifera2
Habenaria furcifera3
Habenaria furcifera1
Habenaria rhodocheila2
Habenaria rhodocheila3
Habenaria rhodocheila4
Habenaria rhodocheila1

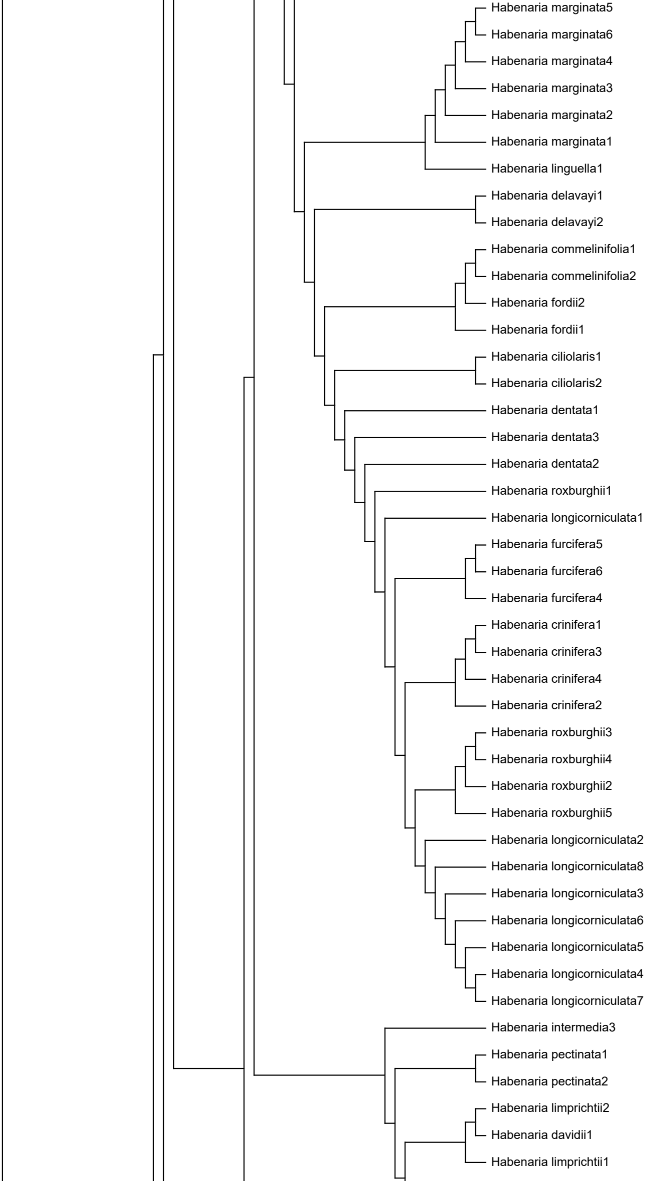
Habenaria marginata5
Habenaria marginata6
Habenaria marginata4
Habenaria marginata3
Habenaria marginata2
Habenaria marginata1
Habenaria linguella1
Habenaria delavayi1
Habenaria delavayi2
Habenaria commelinifolia1
Habenaria commelinifolia2
Habenaria fordii2
Habenaria fordii1
Habenaria ciliolaris1
Habenaria ciliolaris2
Habenaria dentata1
Habenaria dentata3
Habenaria dentata2
Habenaria roxburghii1
Habenaria longicorniculata1
Habenaria furcifera5
Habenaria furcifera6
Habenaria furcifera4
Habenaria crinifera1
Habenaria crinifera3
Habenaria crinifera4
Habenaria crinifera2
Habenaria roxburghii3
Habenaria roxburghii4
Habenaria roxburghii2
Habenaria roxburghii5
Habenaria longicorniculata2
Habenaria longicorniculata8
Habenaria longicorniculata3
Habenaria longicorniculata6
Habenaria longicorniculata5
Habenaria longicorniculata4
Habenaria longicorniculata7
Habenaria intermedia3
Habenaria pectinata1
Habenaria pectinata2
Habenaria limprichtii2
Habenaria davidii1
Habenaria limprichtii1

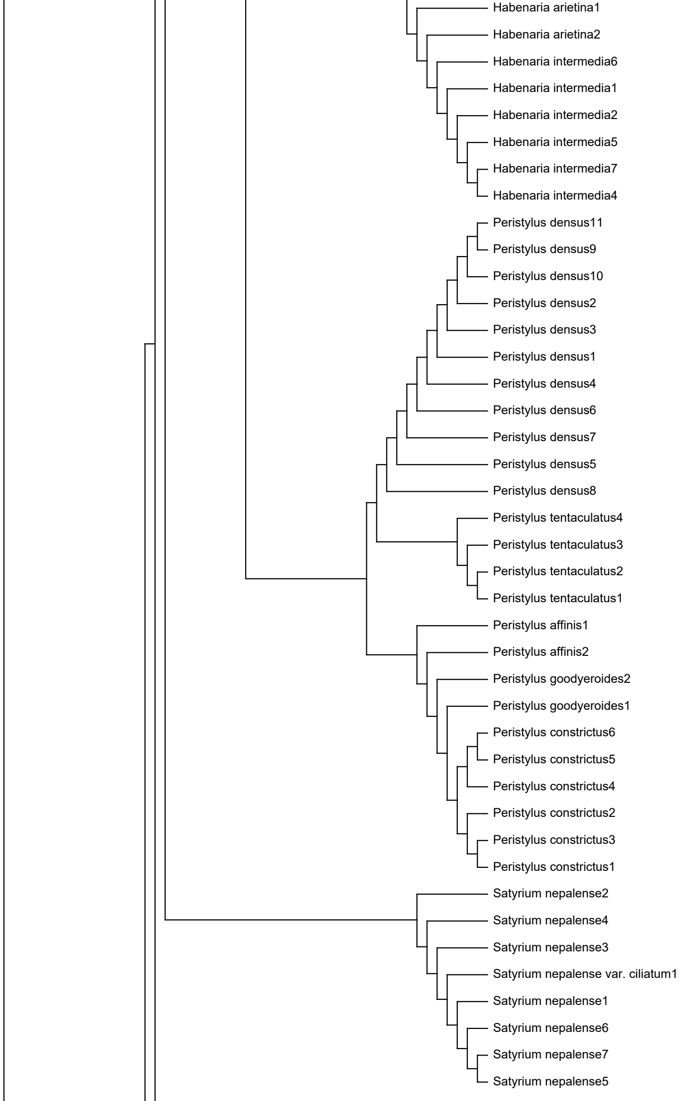
Habenaria arietina1
Habenaria arietina2
Habenaria intermedia6
Habenaria intermedia1
Habenaria intermedia2
Habenaria intermedia5
Habenaria intermedia7
Habenaria intermedia4
Peristylus densus11
Peristylus densus9
Peristylus densus10
Peristylus densus2
Peristylus densus3
Peristylus densus1
Peristylus densus4
Peristylus densus6
Peristylus densus7
Peristylus densus5
Peristylus densus8
Peristylus tentaculatus4
Peristylus tentaculatus3
Peristylus tentaculatus2
Peristylus tentaculatus1
Peristylus affinis1
Peristylus affinis2
Peristylus goodyeroides2
Peristylus goodyeroides1
Peristylus constrictus6
Peristylus constrictus5
Peristylus constrictus4
Peristylus constrictus2
Peristylus constrictus3
Peristylus constrictus1
Satyrium nepalense2
Satyrium nepalense4
Satyrium nepalense3
Satyrium nepalense var. ciliatum1
Satyrium nepalense1
Satyrium nepalense6
Satyrium nepalense7
Satyrium nepalense5

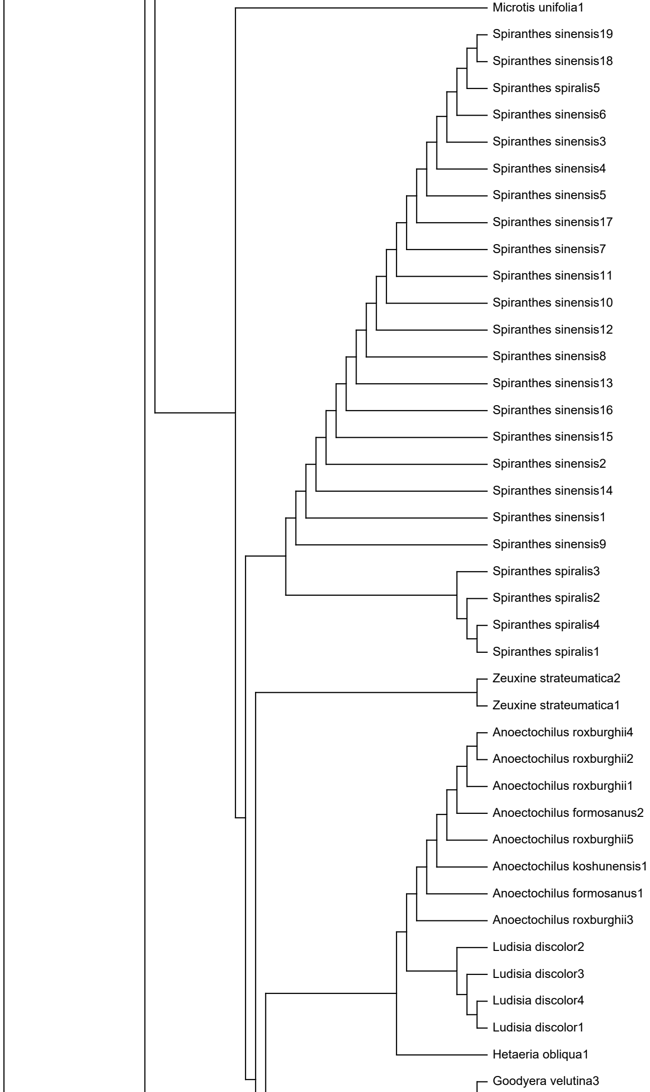

Microtis unifolia1
Spiranthes sinensis19
Spiranthes sinensis18
Spiranthes spiralis5
Spiranthes sinensis6
Spiranthes sinensis3
Spiranthes sinensis4
Spiranthes sinensis5
Spiranthes sinensis17
Spiranthes sinensis7
Spiranthes sinensis11
Spiranthes sinensis10
Spiranthes sinensis12
Spiranthes sinensis8
Spiranthes sinensis13
Spiranthes sinensis16
Spiranthes sinensis15
Spiranthes sinensis2
Spiranthes sinensis14
Spiranthes sinensis1
Spiranthes sinensis9
Spiranthes spiralis3
Spiranthes spiralis2
Spiranthes spiralis4
Spiranthes spiralis1
Zeuxine strateumatica2
Zeuxine strateumatica1
Anoectochilus roxburghii4
Anoectochilus roxburghii2
Anoectochilus roxburghii1
Anoectochilus formosanus2
Anoectochilus roxburghii5
Anoectochilus koshunensis1
Anoectochilus formosanus1
Anoectochilus roxburghii3
Ludisia discolor2
Ludisia discolor3
Ludisia discolor4
Ludisia discolor1
Hetaeria obliqua1
Goodyera velutina3

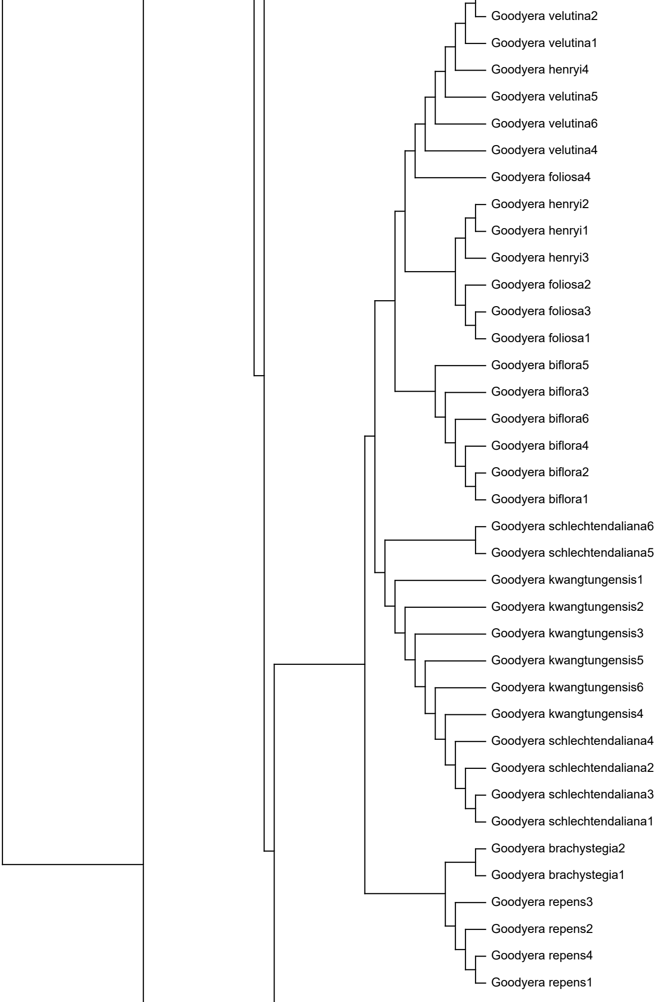
Goodyera velutina2
Goodyera velutina1
Goodyera henryi4
Goodyera velutina5
Goodyera velutina6
Goodyera velutina4
Goodyera foliosa4
Goodyera henryi2
Goodyera henryi1
Goodyera henryi3
Goodyera foliosa2
Goodyera foliosa3
Goodyera foliosa1
Goodyera biflora5
Goodyera biflora3
Goodyera biflora6
Goodyera biflora4
Goodyera biflora2
Goodyera biflora1
Goodyera schlechtendaliana6
Goodyera schlechtendaliana5
Goodyera kwangtungensis1
Goodyera kwangtungensis2
Goodyera kwangtungensis3
Goodyera kwangtungensis5
Goodyera kwangtungensis6
Goodyera kwangtungensis4
Goodyera schlechtendaliana4
Goodyera schlechtendaliana2
Goodyera schlechtendaliana3
Goodyera schlechtendaliana1
Goodyera brachystegia2
Goodyera brachystegia1
Goodyera repens3
Goodyera repens2
Goodyera repens4
Goodyera repens1

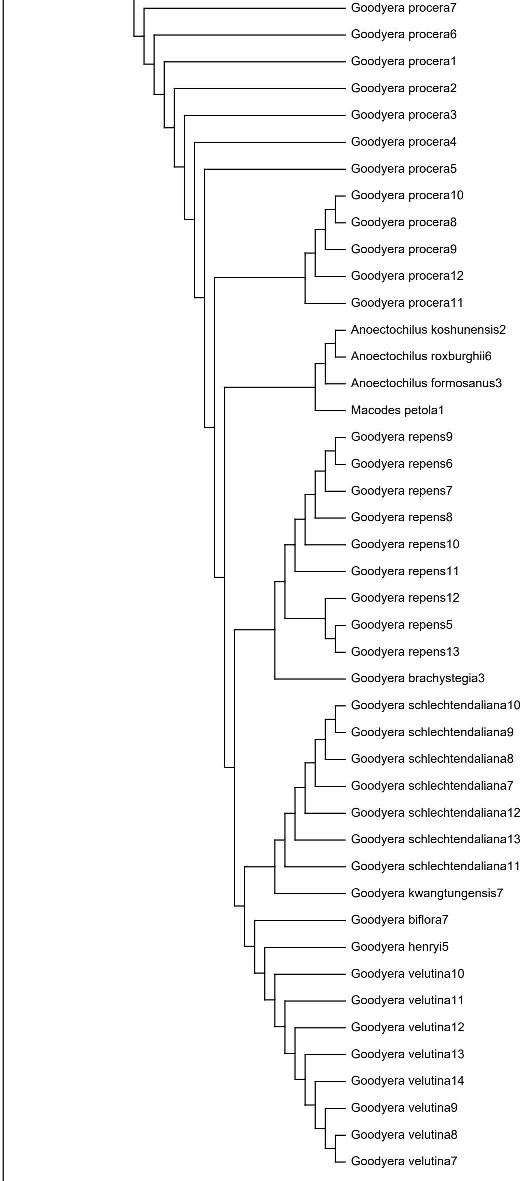

Goodyera procera7
Goodyera procera6
Goodyera procera1
Goodyera procera2
Goodyera procera3
Goodyera procera4
Goodyera procera5
Goodyera procera10
Goodyera procera8
Goodyera procera9
Goodyera procera12
Goodyera procera11
Anoectochilus koshunensis2
Anoectochilus roxburghii6
Anoectochilus formosanus3
Macodes petola1
Goodyera repens9
Goodyera repens6
Goodyera repens7
Goodyera repens8
Goodyera repens10
Goodyera repens11
Goodyera repens12
Goodyera repens5
Goodyera repens13
Goodyera brachystegia3
Goodyera schlechtendaliana10
Goodyera schlechtendaliana9
Goodyera schlechtendaliana8
Goodyera schlechtendaliana7
Goodyera schlechtendaliana12
Goodyera schlechtendaliana13
Goodyera schlechtendaliana11
Goodyera kwangtungensis7
Goodyera biflora7
Goodyera henryi5
Goodyera velutina10
Goodyera velutina11
Goodyera velutina12
Goodyera velutina13
Goodyera velutina14
Goodyera velutina9
Goodyera velutina8
Goodyera velutina7

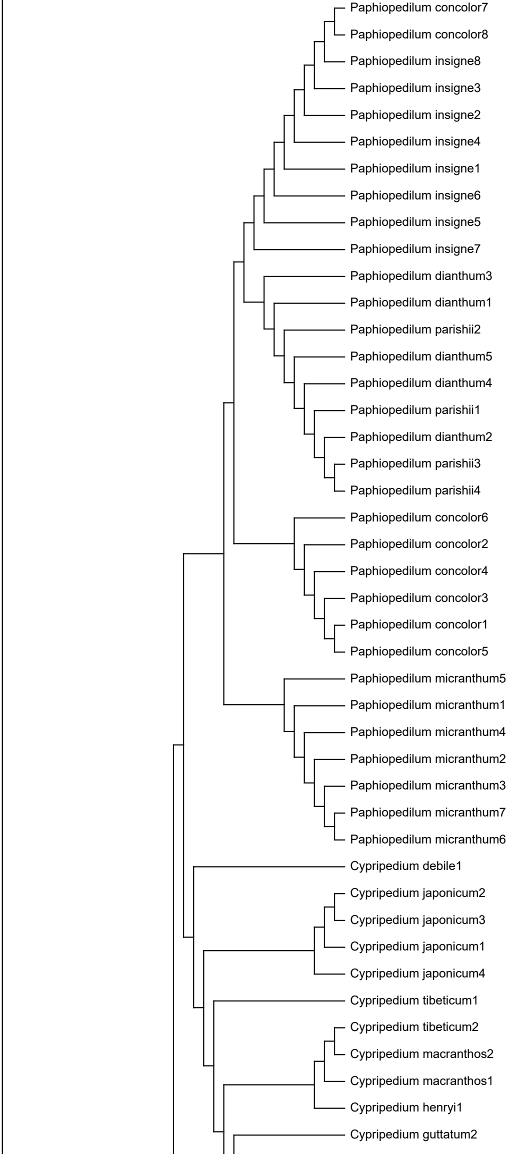
Paphiopedilum concolor7
Paphiopedilum concolor8
Paphiopedilum insigne8
Paphiopedilum insigne3
Paphiopedilum insigne2
Paphiopedilum insigne4
Paphiopedilum insigne1
Paphiopedilum insigne6
Paphiopedilum insigne5
Paphiopedilum insigne7
Paphiopedilum dianthum3
Paphiopedilum dianthum1
Paphiopedilum parishii2
Paphiopedilum dianthum5
Paphiopedilum dianthum4
Paphiopedilum parishii1
Paphiopedilum dianthum2
Paphiopedilum parishii3
Paphiopedilum parishii4
Paphiopedilum concolor6
Paphiopedilum concolor2
Paphiopedilum concolor4
Paphiopedilum concolor3
Paphiopedilum concolor1
Paphiopedilum concolor5
Paphiopedilum micranthum5
Paphiopedilum micranthum1
Paphiopedilum micranthum4
Paphiopedilum micranthum2
Paphiopedilum micranthum3
Paphiopedilum micranthum7
Paphiopedilum micranthum6
Cypripedium debile1
Cypripedium japonicum2
Cypripedium japonicum3
Cypripedium japonicum1
Cypripedium japonicum4
Cypripedium tibeticum1
Cypripedium tibeticum2
Cypripedium macranthos2
Cypripedium macranthos1
Cypripedium henryi1
Cypripedium guttatum2

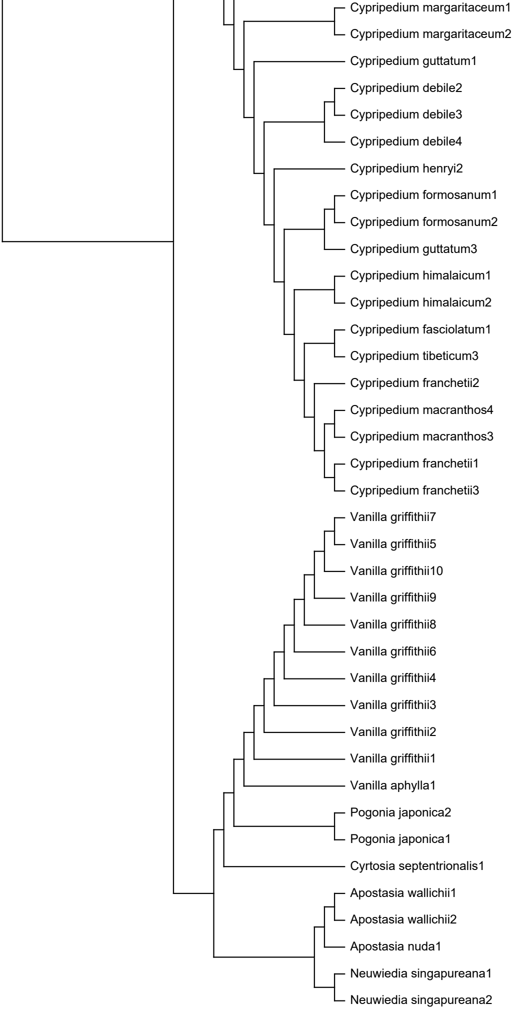
Cypripedium margaritaceum1
Cypripedium margaritaceum2
Cypripedium guttatum1
Cypripedium debile2
Cypripedium debile3
Cypripedium debile4
Cypripedium henryi2
Cypripedium formosanum1
Cypripedium formosanum2
Cypripedium guttatum3
Cypripedium himalaicum1
Cypripedium himalaicum2
Cypripedium fasciolatum1
Cypripedium tibeticum3
Cypripedium franchetii2
Cypripedium macranthos4
Cypripedium macranthos3
Cypripedium franchetii1
Cypripedium franchetii3
Vanilla griffithii7
Vanilla griffithii5
Vanilla griffithii10
Vanilla griffithii9
Vanilla griffithii8
Vanilla griffithii6
Vanilla griffithii4
Vanilla griffithii3
Vanilla griffithii2
Vanilla griffithii1
Vanilla aphylla1
Pogonia japonica2
Pogonia japonica1
Cyrtosia septentrionalis1
Apostasia wallichii1
Apostasia wallichii2
Apostasia nuda1
Neuwiedia singapureana1
Neuwiedia singapureana2
