# Supplementary material for: DNA barcoding of medicinal orchids in Asia
Source: Sci Rep. 2021 Dec 8;11:23651. doi: 10.1038/s41598-021-03025-0 (PMC8654824; doi:10.1038/s41598-021-03025-0)
Supplement: Supplementary file 8 — Supplementary Figure S8. [file 41598_2021_3025_MOESM8_ESM.pdf]

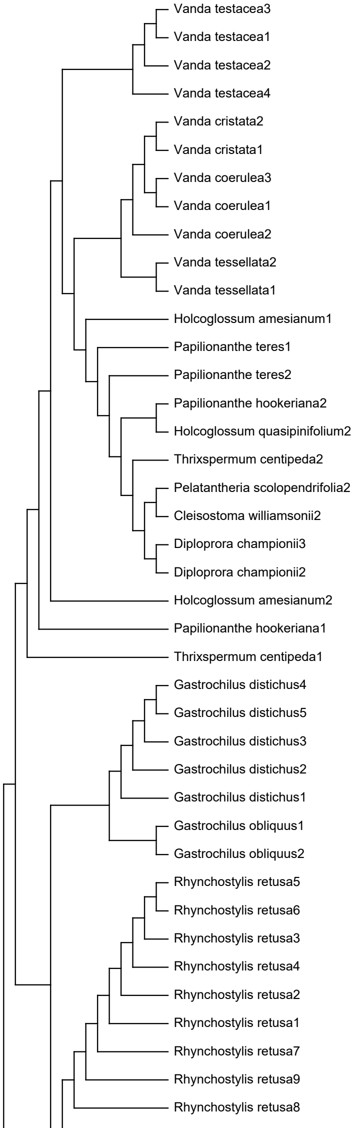
Vanda testacea3
Vanda testacea1
Vanda testacea2
Vanda testacea4
Vanda cristata2
Vanda cristata1
Vanda coerulea3
Vanda coerulea1
Vanda coerulea2
Vanda tessellata2
Vanda tessellata1
Holcoglossum amesianum1
Papilionanthe teres1
Papilionanthe teres2
Papilionanthe hookeriana2
Holcoglossum quasipinifolium2
Thrixspermum centipeda2
Pelatantheria scolopendrifolia2
Cleisostoma williamsonii2
Diploprora championii3
Diploprora championii2
Holcoglossum amesianum2
Papilionanthe hookeriana1
Thrixspermum centipeda1
Gastrochilus distichus4
Gastrochilus distichus5
Gastrochilus distichus3
Gastrochilus distichus2
Gastrochilus distichus1
Gastrochilus obliquus1
Gastrochilus obliquus2
Rhynchostylis retusa5
Rhynchostylis retusa6
Rhynchostylis retusa3
Rhynchostylis retusa4
Rhynchostylis retusa2
Rhynchostylis retusa1
Rhynchostylis retusa7
Rhynchostylis retusa9
Rhynchostylis retusa8

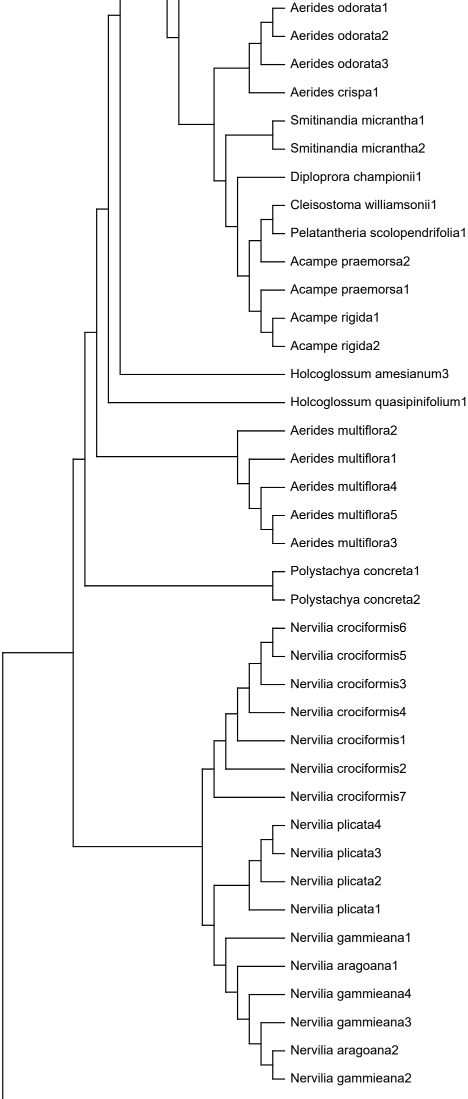
Aerides odorata1
Aerides odorata2
Aerides odorata3
Aerides crispa1
Smitinandia micrantha1
Smitinandia micrantha2
Diploprora championii1
Cleisostoma williamsonii1
Pelatantheria scolopendrifolia1
Acampe praemorsa2
Acampe praemorsa1
Acampe rigida1
Acampe rigida2
Holcoglossum amesianum3
Holcoglossum quasipinifolium1
Aerides multiflora2
Aerides multiflora1
Aerides multiflora4
Aerides multiflora5
Aerides multiflora3
Polystachya concreta1
Polystachya concreta2
Nervilia crociformis6
Nervilia crociformis5
Nervilia crociformis3
Nervilia crociformis4
Nervilia crociformis1
Nervilia crociformis2
Nervilia crociformis7
Nervilia plicata4
Nervilia plicata3
Nervilia plicata2
Nervilia plicata1
Nervilia gammieana1
Nervilia aragoana1
Nervilia gammieana4
Nervilia gammieana3
Nervilia aragoana2
Nervilia gammieana2

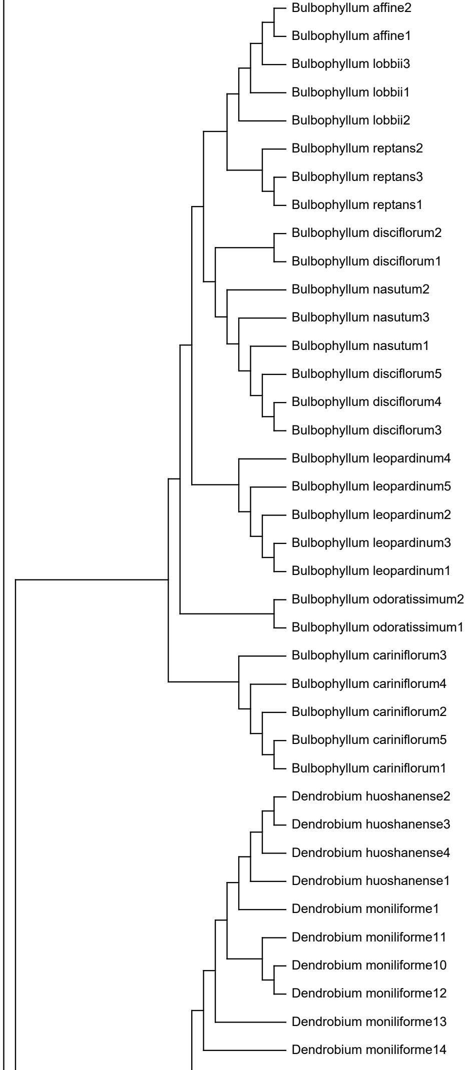

Bulbophyllum affine2
Bulbophyllum affine1
Bulbophyllum lobbii3
Bulbophyllum lobbii1
Bulbophyllum lobbii2
Bulbophyllum reptans2
Bulbophyllum reptans3
Bulbophyllum reptans1
Bulbophyllum disciflorum2
Bulbophyllum disciflorum1
Bulbophyllum nasutum2
Bulbophyllum nasutum3
Bulbophyllum nasutum1
Bulbophyllum disciflorum5
Bulbophyllum disciflorum4
Bulbophyllum disciflorum3
Bulbophyllum leopardinum4
Bulbophyllum leopardinum5
Bulbophyllum leopardinum2
Bulbophyllum leopardinum3
Bulbophyllum leopardinum1
Bulbophyllum odoratissimum2
Bulbophyllum odoratissimum1
Bulbophyllum cariniflorum3
Bulbophyllum cariniflorum4
Bulbophyllum cariniflorum2
Bulbophyllum cariniflorum5
Bulbophyllum cariniflorum1
Dendrobium huoshanense2
Dendrobium huoshanense3
Dendrobium huoshanense4
Dendrobium huoshanense1
Dendrobium moniliforme1
Dendrobium moniliforme11
Dendrobium moniliforme10
Dendrobium moniliforme12
Dendrobium moniliforme13
Dendrobium moniliforme14

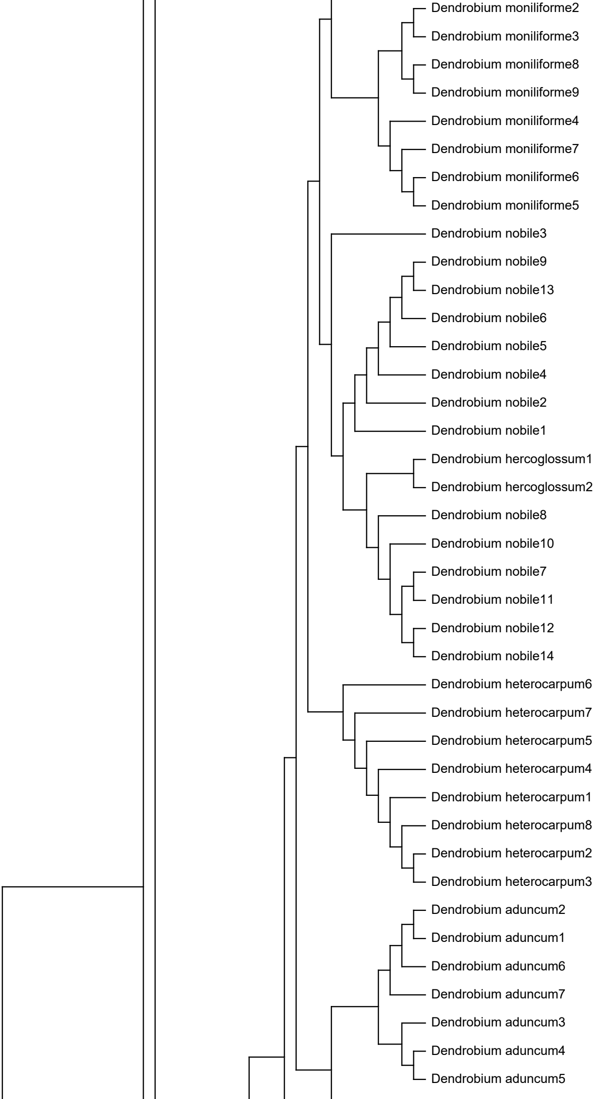

Dendrobium moniliforme2
Dendrobium moniliforme3
Dendrobium moniliforme8
Dendrobium moniliforme9
Dendrobium moniliforme4
Dendrobium moniliforme7
Dendrobium moniliforme6
Dendrobium moniliforme5
Dendrobium nobile3
Dendrobium nobile9
Dendrobium nobile13
Dendrobium nobile6
Dendrobium nobile5
Dendrobium nobile4
Dendrobium nobile2
Dendrobium nobile1
Dendrobium hercoglossum1
Dendrobium hercoglossum2
Dendrobium nobile8
Dendrobium nobile10
Dendrobium nobile7
Dendrobium nobile11
Dendrobium nobile12
Dendrobium nobile14
Dendrobium heterocarpum6
Dendrobium heterocarpum7
Dendrobium heterocarpum5
Dendrobium heterocarpum4
Dendrobium heterocarpum1
Dendrobium heterocarpum8
Dendrobium heterocarpum2
Dendrobium heterocarpum3
Dendrobium aduncum2
Dendrobium aduncum1
Dendrobium aduncum6
Dendrobium aduncum7
Dendrobium aduncum3
Dendrobium aduncum4
Dendrobium aduncum5

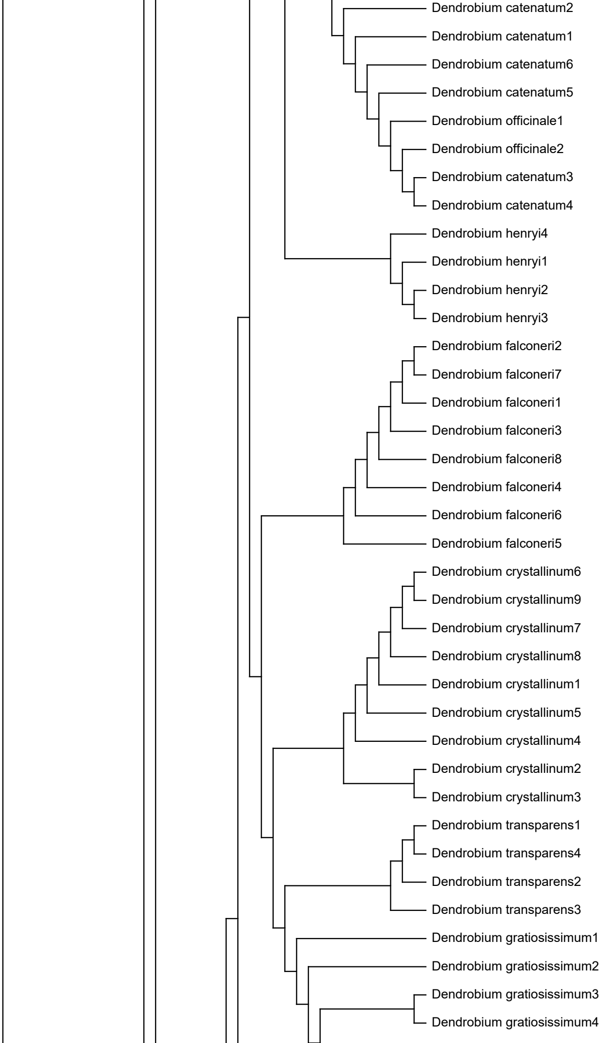
Dendrobium catenatum2
Dendrobium catenatum1
Dendrobium catenatum6
Dendrobium catenatum5
Dendrobium officinale1
Dendrobium officinale2
Dendrobium catenatum3
Dendrobium catenatum4
Dendrobium henryi4
Dendrobium henryi1
Dendrobium henryi2
Dendrobium henryi3
Dendrobium falconeri2
Dendrobium falconeri7
Dendrobium falconeri1
Dendrobium falconeri3
Dendrobium falconeri8
Dendrobium falconeri4
Dendrobium falconeri6
Dendrobium falconeri5
Dendrobium crystallinum6
Dendrobium crystallinum9
Dendrobium crystallinum7
Dendrobium crystallinum8
Dendrobium crystallinum1
Dendrobium crystallinum5
Dendrobium crystallinum4
Dendrobium crystallinum2
Dendrobium crystallinum3
Dendrobium transparens1
Dendrobium transparens4
Dendrobium transparens2
Dendrobium transparens3
Dendrobium gratiosissimum1
Dendrobium gratiosissimum2
Dendrobium gratiosissimum3
Dendrobium gratiosissimum4

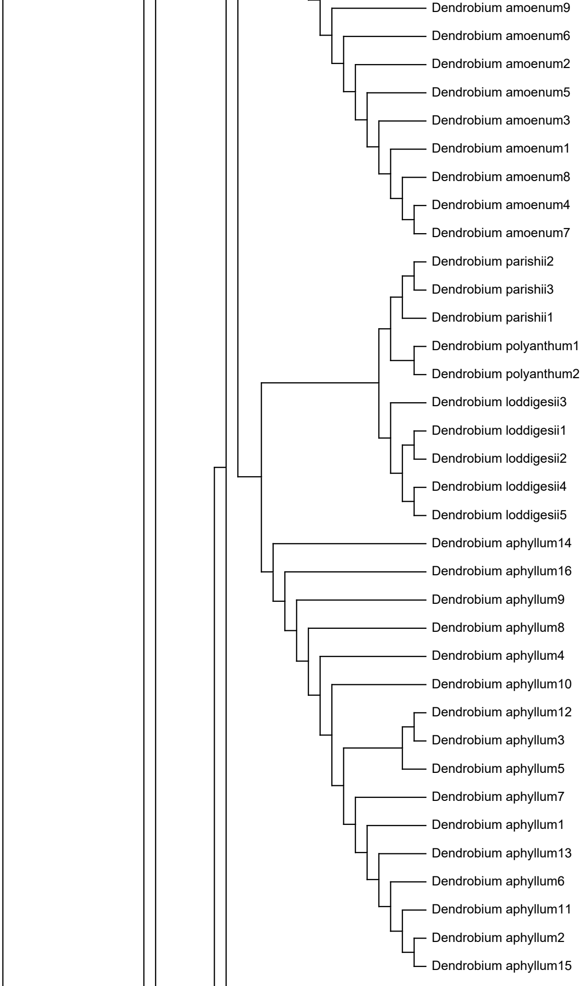

Dendrobium amoenum9
Dendrobium amoenum6
Dendrobium amoenum2
Dendrobium amoenum5
Dendrobium amoenum3
Dendrobium amoenum1
Dendrobium amoenum8
Dendrobium amoenum4
Dendrobium amoenum7
Dendrobium parishii2
Dendrobium parishii3
Dendrobium parishii1
Dendrobium polyanthum1
Dendrobium polyanthum2
Dendrobium loddigesii3
Dendrobium loddigesii1
Dendrobium loddigesii2
Dendrobium loddigesii4
Dendrobium loddigesii5
Dendrobium aphyllum14
Dendrobium aphyllum16
Dendrobium aphyllum9
Dendrobium aphyllum8
Dendrobium aphyllum4
Dendrobium aphyllum10
Dendrobium aphyllum12
Dendrobium aphyllum3
Dendrobium aphyllum5
Dendrobium aphyllum7
Dendrobium aphyllum1
Dendrobium aphyllum13
Dendrobium aphyllum6
Dendrobium aphyllum11
Dendrobium aphyllum2
Dendrobium aphyllum15

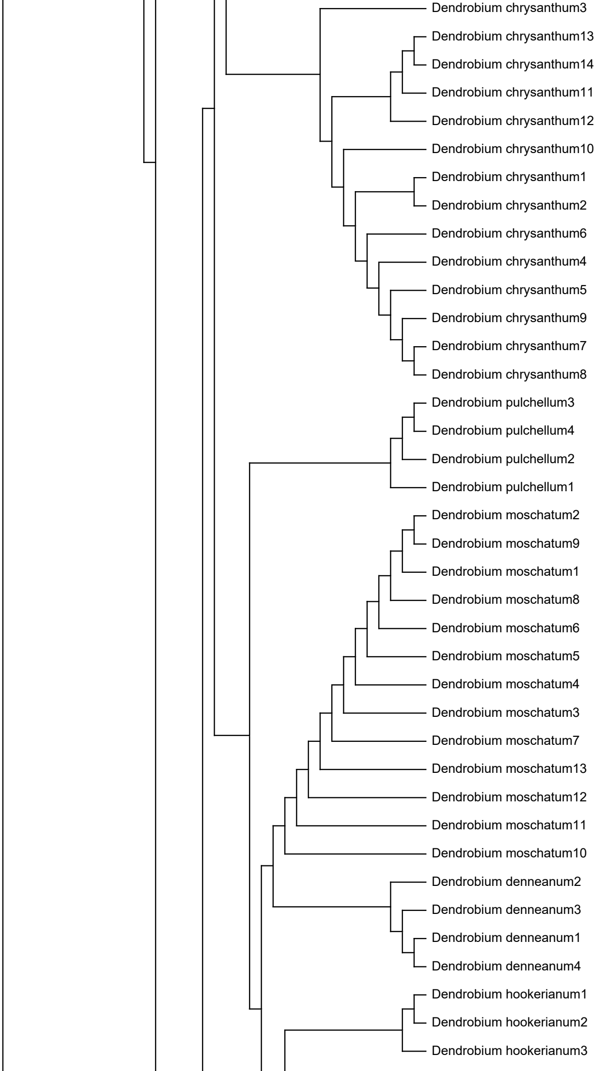

Dendrobium chrysanthum3
Dendrobium chrysanthum13
Dendrobium chrysanthum14
Dendrobium chrysanthum11
Dendrobium chrysanthum12
Dendrobium chrysanthum10
Dendrobium chrysanthum1
Dendrobium chrysanthum2
Dendrobium chrysanthum6
Dendrobium chrysanthum4
Dendrobium chrysanthum5
Dendrobium chrysanthum9
Dendrobium chrysanthum7
Dendrobium chrysanthum8
Dendrobium pulchellum3
Dendrobium pulchellum4
Dendrobium pulchellum2
Dendrobium pulchellum1
Dendrobium moschatum2
Dendrobium moschatum9
Dendrobium moschatum1
Dendrobium moschatum8
Dendrobium moschatum6
Dendrobium moschatum5
Dendrobium moschatum4
Dendrobium moschatum3
Dendrobium moschatum7
Dendrobium moschatum13
Dendrobium moschatum12
Dendrobium moschatum11
Dendrobium moschatum10
Dendrobium denneanum2
Dendrobium denneanum3
Dendrobium denneanum1
Dendrobium denneanum4
Dendrobium hookerianum1
Dendrobium hookerianum2
Dendrobium hookerianum3

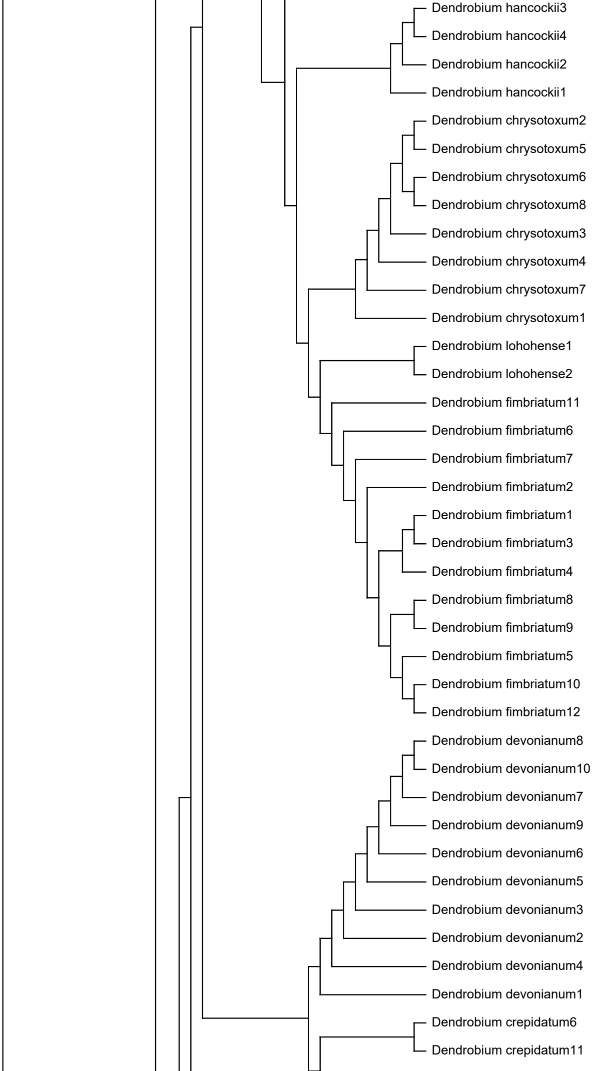
Dendrobium hancockii3
Dendrobium hancockii4
Dendrobium hancockii2
Dendrobium hancockii1
Dendrobium chrysotoxum2
Dendrobium chrysotoxum5
Dendrobium chrysotoxum6
Dendrobium chrysotoxum8
Dendrobium chrysotoxum3
Dendrobium chrysotoxum4
Dendrobium chrysotoxum7
Dendrobium chrysotoxum1
Dendrobium lohohense1
Dendrobium lohohense2
Dendrobium fimbriatum11
Dendrobium fimbriatum6
Dendrobium fimbriatum7
Dendrobium fimbriatum2
Dendrobium fimbriatum1
Dendrobium fimbriatum3
Dendrobium fimbriatum4
Dendrobium fimbriatum8
Dendrobium fimbriatum9
Dendrobium fimbriatum5
Dendrobium fimbriatum10
Dendrobium fimbriatum12
Dendrobium devonianum8
Dendrobium devonianum10
Dendrobium devonianum7
Dendrobium devonianum9
Dendrobium devonianum6
Dendrobium devonianum5
Dendrobium devonianum3
Dendrobium devonianum2
Dendrobium devonianum4
Dendrobium devonianum1
Dendrobium crepidatum6
Dendrobium crepidatum11

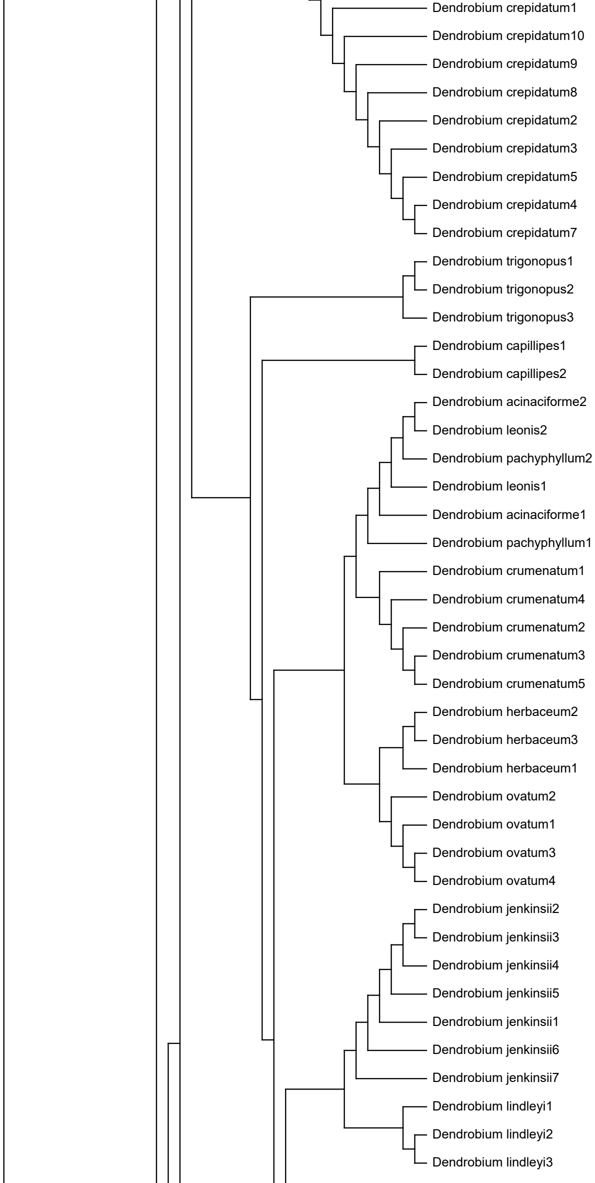
Dendrobium crepidatum1
Dendrobium crepidatum10
Dendrobium crepidatum9
Dendrobium crepidatum8
Dendrobium crepidatum2
Dendrobium crepidatum3
Dendrobium crepidatum5
Dendrobium crepidatum4
Dendrobium crepidatum7
Dendrobium trigonopus1
Dendrobium trigonopus2
Dendrobium trigonopus3
Dendrobium capillipes1
Dendrobium capillipes2
Dendrobium acinaciforme2
Dendrobium leonis2
Dendrobium pachyphyllum2
Dendrobium leonis1
Dendrobium acinaciforme1
Dendrobium pachyphyllum1
Dendrobium crumenatum1
Dendrobium crumenatum4
Dendrobium crumenatum2
Dendrobium crumenatum3
Dendrobium crumenatum5
Dendrobium herbaceum2
Dendrobium herbaceum3
Dendrobium herbaceum1
Dendrobium ovatum2
Dendrobium ovatum1
Dendrobium ovatum3
Dendrobium ovatum4
Dendrobium jenkinsii2
Dendrobium jenkinsii3
Dendrobium jenkinsii4
Dendrobium jenkinsii5
Dendrobium jenkinsii1
Dendrobium jenkinsii6
Dendrobium jenkinsii7
Dendrobium lindleyi1
Dendrobium lindleyi2
Dendrobium lindleyi3

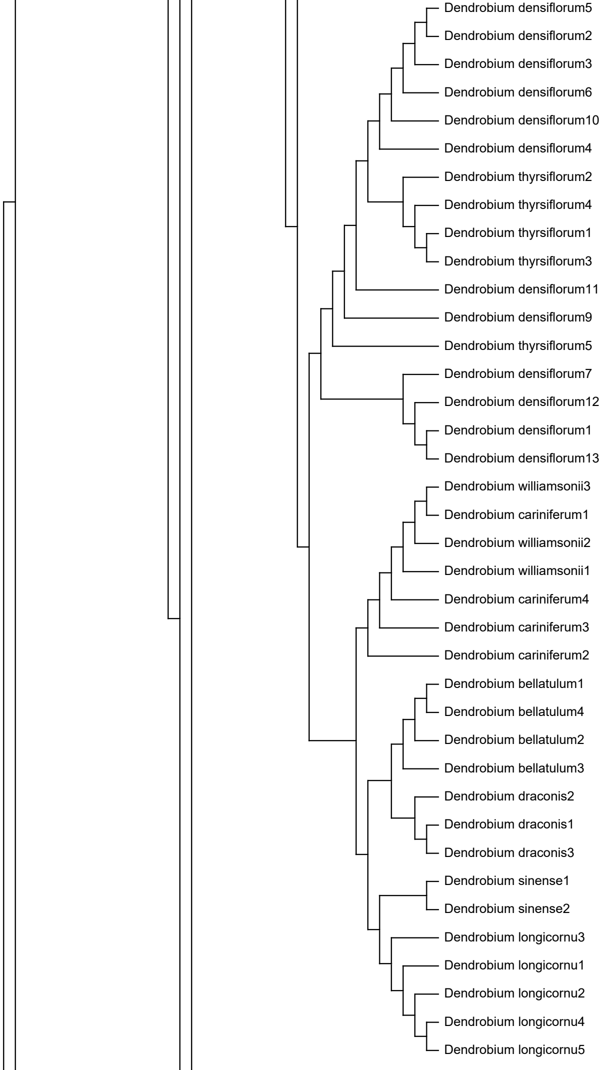

Dendrobium densiflorum5
Dendrobium densiflorum2
Dendrobium densiflorum3
Dendrobium densiflorum6
Dendrobium densiflorum10
Dendrobium densiflorum4
Dendrobium thyrsiflorum2
Dendrobium thyrsiflorum4
Dendrobium thyrsiflorum1
Dendrobium thyrsiflorum3
Dendrobium densiflorum11
Dendrobium densiflorum9
Dendrobium thyrsiflorum5
Dendrobium densiflorum7
Dendrobium densiflorum12
Dendrobium densiflorum1
Dendrobium densiflorum13
Dendrobium williamsonii3
Dendrobium cariniferum1
Dendrobium williamsonii2
Dendrobium williamsonii1
Dendrobium cariniferum4
Dendrobium cariniferum3
Dendrobium cariniferum2
Dendrobium bellatulum1
Dendrobium bellatulum4
Dendrobium bellatulum2
Dendrobium bellatulum3
Dendrobium draconis2
Dendrobium draconis1
Dendrobium draconis3
Dendrobium sinense1
Dendrobium sinense2
Dendrobium longicornu3
Dendrobium longicornu1
Dendrobium longicornu2
Dendrobium longicornu4
Dendrobium longicornu5

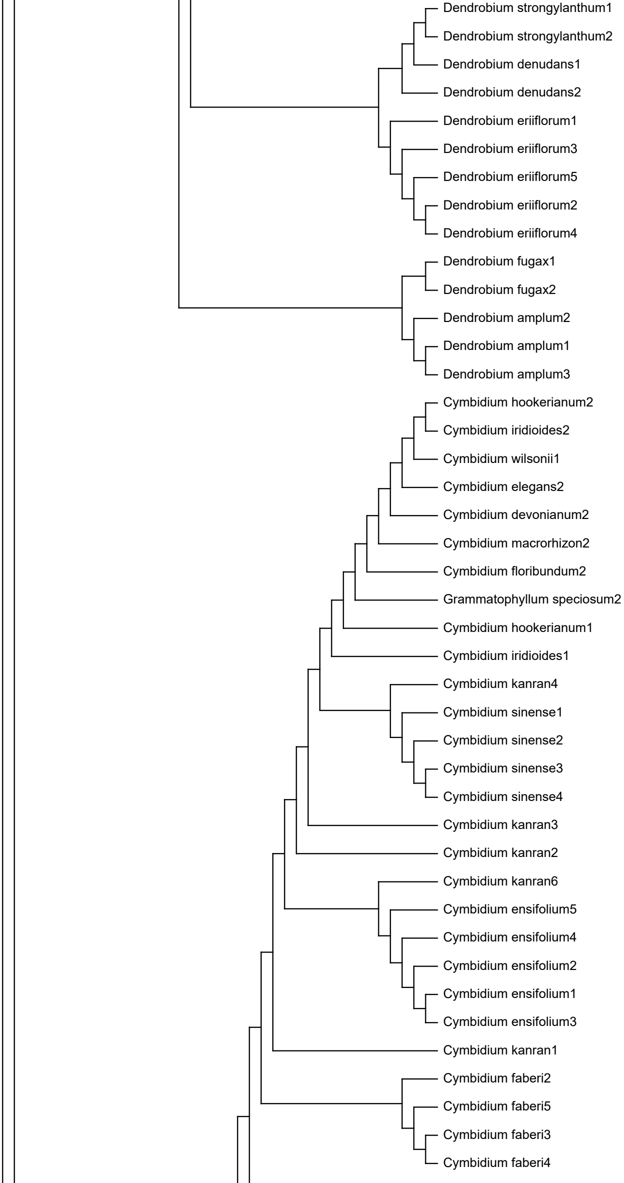
Dendrobium strongylanthum1
Dendrobium strongylanthum2
Dendrobium denudans1
Dendrobium denudans2
Dendrobium eriiflorum1
Dendrobium eriiflorum3
Dendrobium eriiflorum5
Dendrobium eriiflorum2
Dendrobium eriiflorum4
Dendrobium fugax1
Dendrobium fugax2
Dendrobium amplum2
Dendrobium amplum1
Dendrobium amplum3
Cymbidium hookerianum2
Cymbidium iridioides2
Cymbidium wilsonii1
Cymbidium elegans2
Cymbidium devonianum2
Cymbidium macrorhizon2
Cymbidium floribundum2
Grammatophyllum speciosum2
Cymbidium hookerianum1
Cymbidium iridioides1
Cymbidium kanran4
Cymbidium sinense1
Cymbidium sinense2
Cymbidium sinense3
Cymbidium sinense4
Cymbidium kanran3
Cymbidium kanran2
Cymbidium kanran6
Cymbidium ensifolium5
Cymbidium ensifolium4
Cymbidium ensifolium2
Cymbidium ensifolium1
Cymbidium ensifolium3
Cymbidium kanran1
Cymbidium faberi2
Cymbidium faberi5
Cymbidium faberi3
Cymbidium faberi4

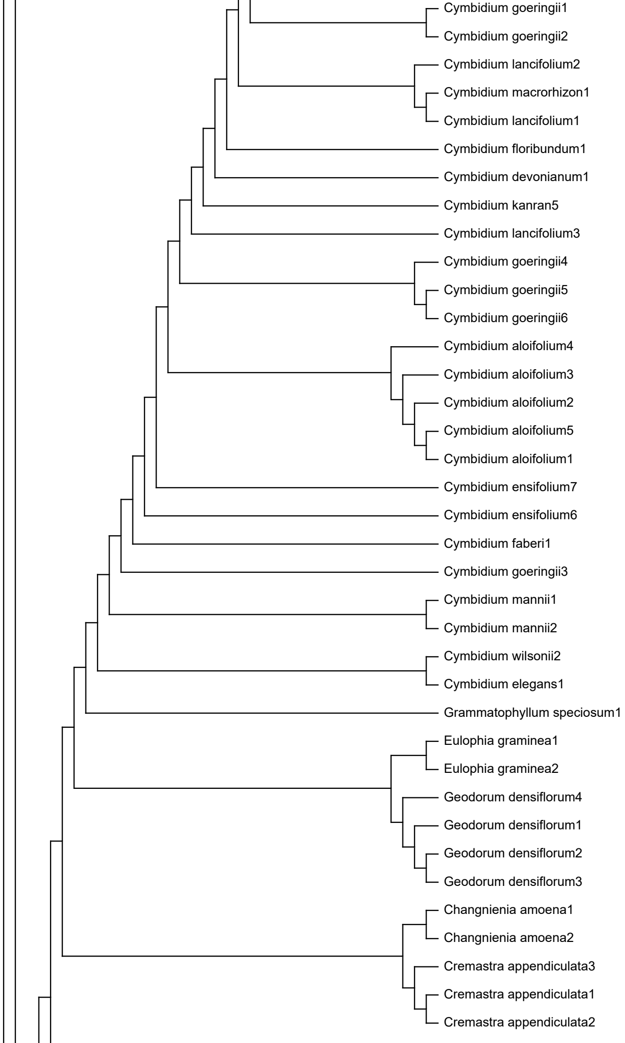

Cymbidium goeringii1
Cymbidium goeringii2
Cymbidium lancifolium2
Cymbidium macrorhizon1
Cymbidium lancifolium1
Cymbidium floribundum1
Cymbidium devonianum1
Cymbidium kanran5
Cymbidium lancifolium3
Cymbidium goeringii4
Cymbidium goeringii5
Cymbidium goeringii6
Cymbidium aloifolium4
Cymbidium aloifolium3
Cymbidium aloifolium2
Cymbidium aloifolium5
Cymbidium aloifolium1
Cymbidium ensifolium7
Cymbidium ensifolium6
Cymbidium faberi1
Cymbidium goeringii3
Cymbidium mannii1
Cymbidium mannii2
Cymbidium wilsonii2
Cymbidium elegans1
Grammatophyllum speciosum1
Eulophia graminea1
Eulophia graminea2
Geodorum densiflorum4
Geodorum densiflorum1
Geodorum densiflorum2
Geodorum densiflorum3
Changnienia amoena1
Changnienia amoena2
Cremastra appendiculata3
Cremastra appendiculata1
Cremastra appendiculata2

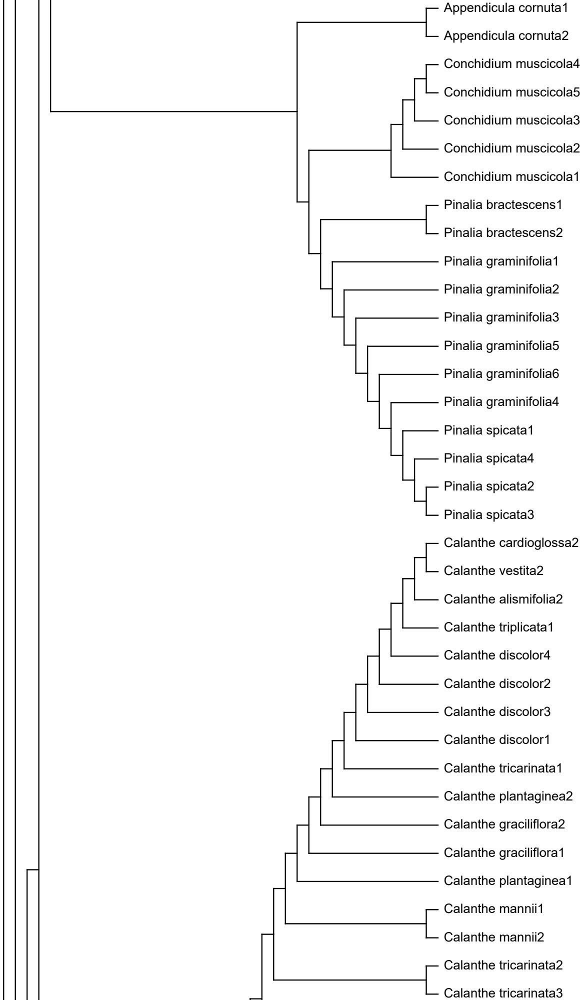
Appendicula cornuta1
Appendicula cornuta2
Conchidium muscicola4
Conchidium muscicola5
Conchidium muscicola3
Conchidium muscicola2
Conchidium muscicola1
Pinalia bractescens1
Pinalia bractescens2
Pinalia graminifolia1
Pinalia graminifolia2
Pinalia graminifolia3
Pinalia graminifolia5
Pinalia graminifolia6
Pinalia graminifolia4
Pinalia spicata1
Pinalia spicata4
Pinalia spicata2
Pinalia spicata3
Calanthe cardioglossa2
Calanthe vestita2
Calanthe alismifolia2
Calanthe triplicata1
Calanthe discolor4
Calanthe discolor2
Calanthe discolor3
Calanthe discolor1
Calanthe tricarinata1
Calanthe plantaginea2
Calanthe graciliflora2
Calanthe graciliflora1
Calanthe plantaginea1
Calanthe mannii1
Calanthe mannii2
Calanthe tricarinata2
Calanthe tricarinata3

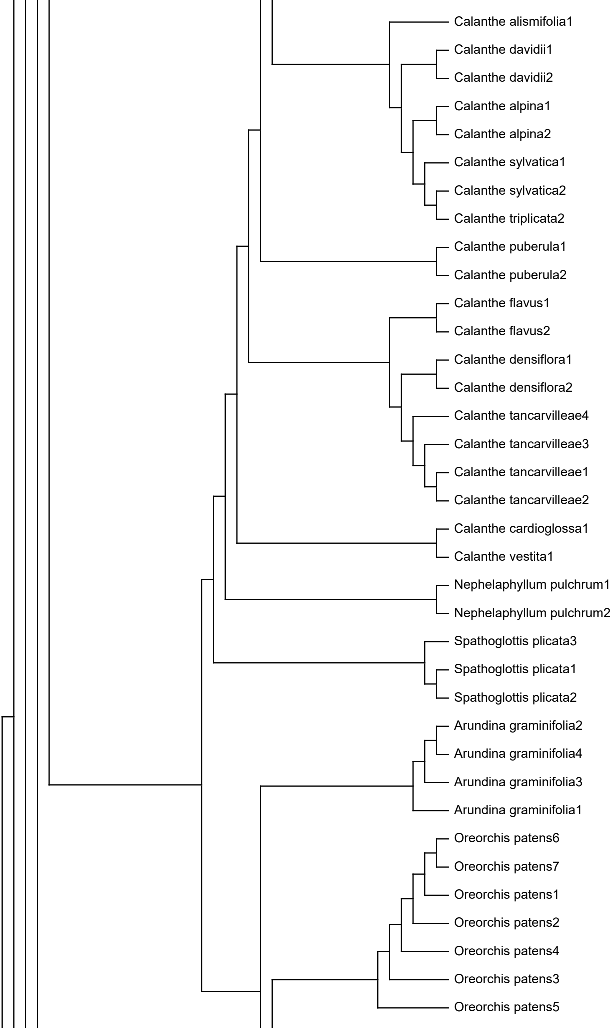
Calanthe alismifolia1
Calanthe davidii1
Calanthe davidii2
Calanthe alpina1
Calanthe alpina2
Calanthe sylvatica1
Calanthe sylvatica2
Calanthe triplicata2
Calanthe puberula1
Calanthe puberula2
Calanthe flavus1
Calanthe flavus2
Calanthe densiflora1
Calanthe densiflora2
Calanthe tancarvilleae4
Calanthe tancarvilleae3
Calanthe tancarvilleae1
Calanthe tancarvilleae2
Calanthe cardioglossa1
Calanthe vestita1
Nephelaphyllum pulchrum1
Nephelaphyllum pulchrum2
Spathoglottis plicata3
Spathoglottis plicata1
Spathoglottis plicata2
Arundina graminifolia2
Arundina graminifolia4
Arundina graminifolia3
Arundina graminifolia1
Oreorchis patens6
Oreorchis patens7
Oreorchis patens1
Oreorchis patens2
Oreorchis patens4
Oreorchis patens3
Oreorchis patens5

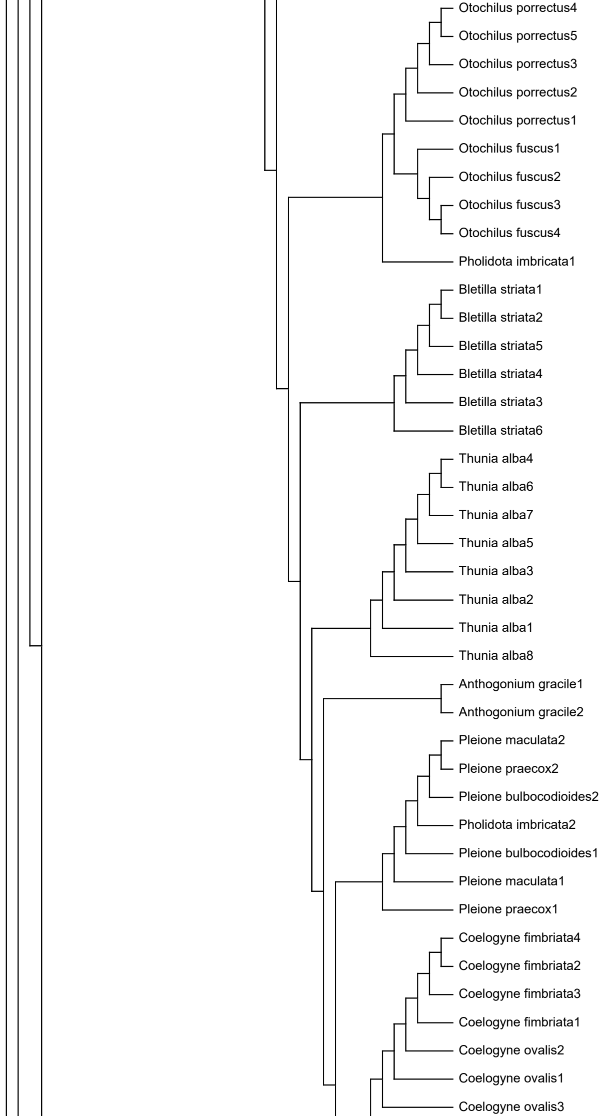
Otochilus porrectus4
Otochilus porrectus5
Otochilus porrectus3
Otochilus porrectus2
Otochilus porrectus1
Otochilus fuscus1
Otochilus fuscus2
Otochilus fuscus3
Otochilus fuscus4
Pholidota imbricata1
Bletilla striata1
Bletilla striata2
Bletilla striata5
Bletilla striata4
Bletilla striata3
Bletilla striata6
Thunia alba4
Thunia alba6
Thunia alba7
Thunia alba5
Thunia alba3
Thunia alba2
Thunia alba1
Thunia alba8
Anthogonium gracile1
Anthogonium gracile2
Pleione maculata2
Pleione praecox2
Pleione bulbocodioides2
Pholidota imbricata2
Pleione bulbocodioides1
Pleione maculata1
Pleione praecox1
Coelogyne fimbriata4
Coelogyne fimbriata2
Coelogyne fimbriata3
Coelogyne fimbriata1
Coelogyne ovalis2
Coelogyne ovalis1
Coelogyne ovalis3

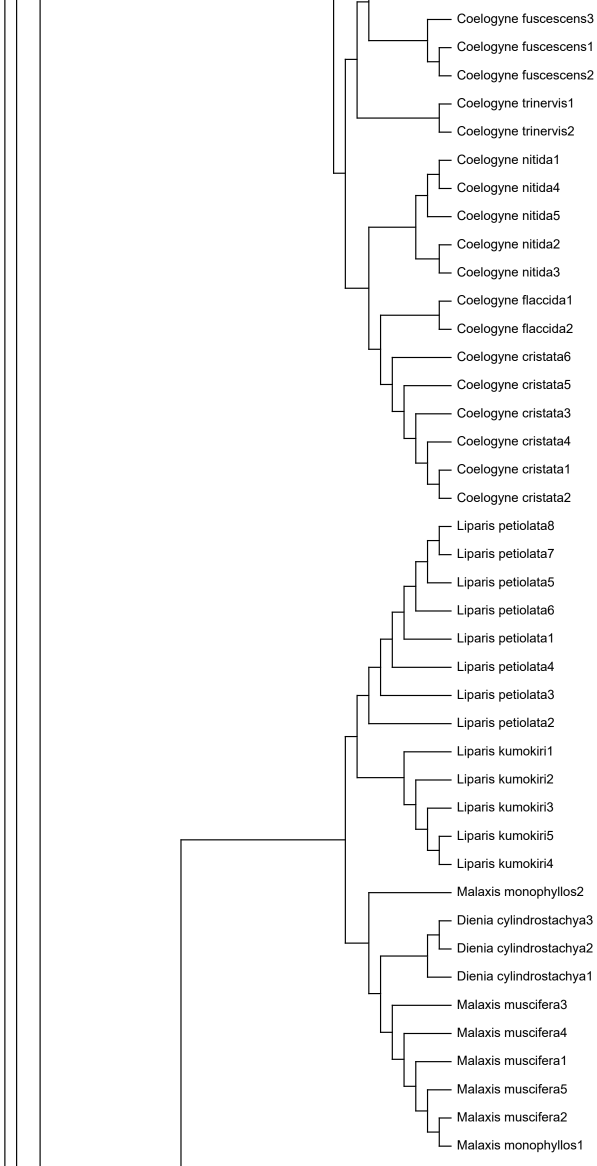

Coelogyne fuscescens3
Coelogyne fuscescens1
Coelogyne fuscescens2
Coelogyne trinervis1
Coelogyne trinervis2
Coelogyne nitida1
Coelogyne nitida4
Coelogyne nitida5
Coelogyne nitida2
Coelogyne nitida3
Coelogyne flaccida1
Coelogyne flaccida2
Coelogyne cristata6
Coelogyne cristata5
Coelogyne cristata3
Coelogyne cristata4
Coelogyne cristata1
Coelogyne cristata2
Liparis petiolata8
Liparis petiolata7
Liparis petiolata5
Liparis petiolata6
Liparis petiolata1
Liparis petiolata4
Liparis petiolata3
Liparis petiolata2
Liparis kumokiri1
Liparis kumokiri2
Liparis kumokiri3
Liparis kumokiri5
Liparis kumokiri4
Malaxis monophyllos2
Dienia cylindrostachya3
Dienia cylindrostachya2
Dienia cylindrostachya1
Malaxis muscifera3
Malaxis muscifera4
Malaxis muscifera1
Malaxis muscifera5
Malaxis muscifera2
Malaxis monophyllos1

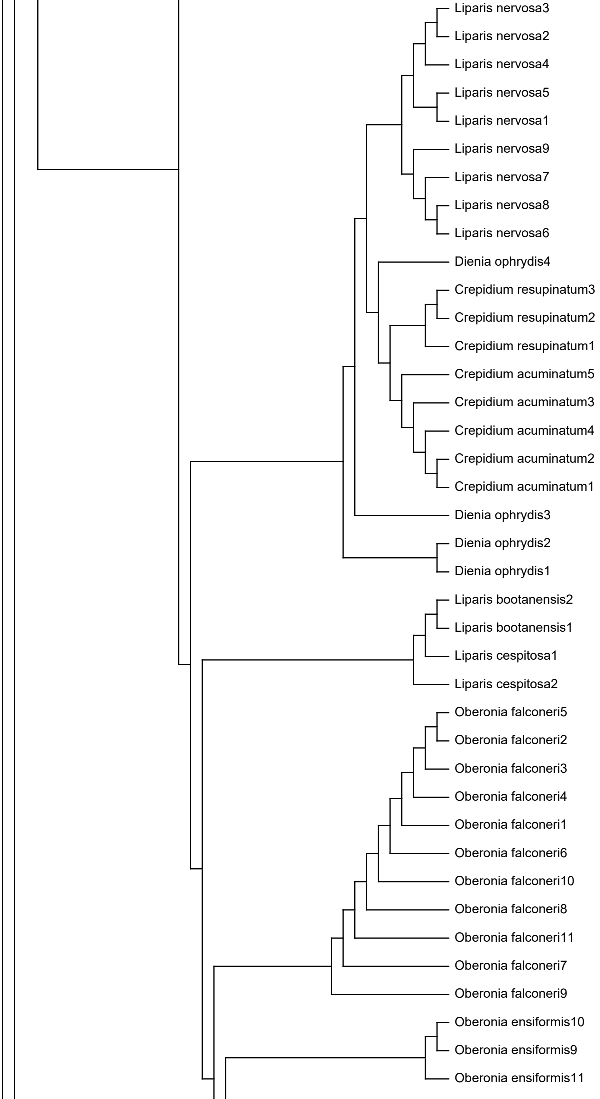
Liparis nervosa3
Liparis nervosa2
Liparis nervosa4
Liparis nervosa5
Liparis nervosa1
Liparis nervosa9
Liparis nervosa7
Liparis nervosa8
Liparis nervosa6
Dienia ophrydis4
Crepidium resupinatum3
Crepidium resupinatum2
Crepidium resupinatum1
Crepidium acuminatum5
Crepidium acuminatum3
Crepidium acuminatum4
Crepidium acuminatum2
Crepidium acuminatum1
Dienia ophrydis3
Dienia ophrydis2
Dienia ophrydis1
Liparis bootanensis2
Liparis bootanensis1
Liparis cespitosa1
Liparis cespitosa2
Oberonia falconeri5
Oberonia falconeri2
Oberonia falconeri3
Oberonia falconeri4
Oberonia falconeri1
Oberonia falconeri6
Oberonia falconeri10
Oberonia falconeri8
Oberonia falconeri11
Oberonia falconeri7
Oberonia falconeri9
Oberonia ensiformis10
Oberonia ensiformis9
Oberonia ensiformis11

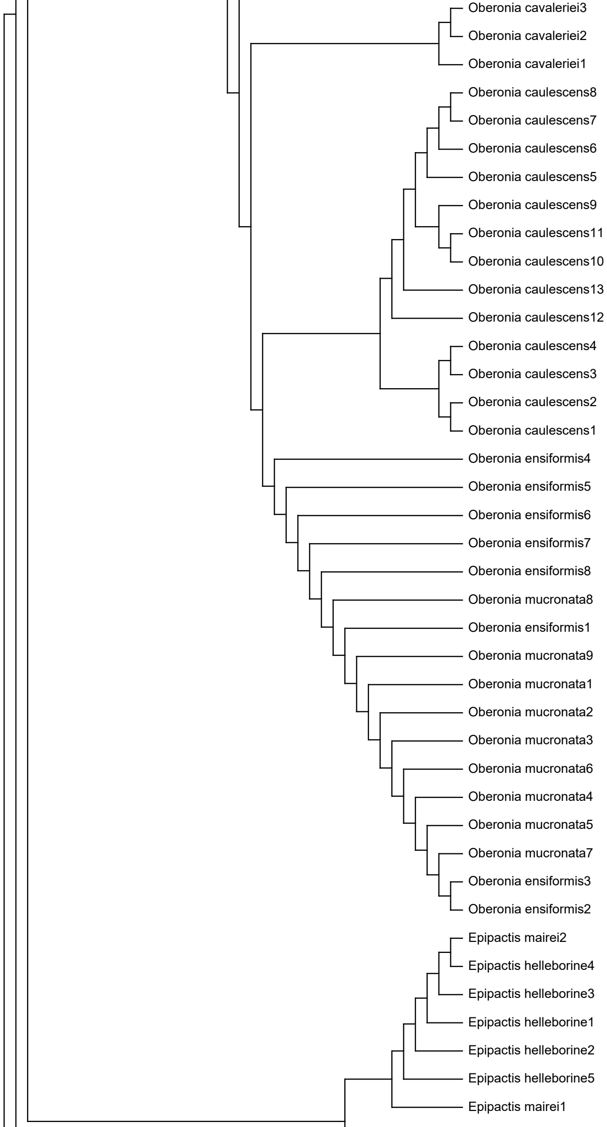
Oberonia cavaleriei3
Oberonia cavaleriei2
Oberonia cavaleriei1
Oberonia caulescens8
Oberonia caulescens7
Oberonia caulescens6
Oberonia caulescens5
Oberonia caulescens9
Oberonia caulescens11
Oberonia caulescens10
Oberonia caulescens13
Oberonia caulescens12
Oberonia caulescens4
Oberonia caulescens3
Oberonia caulescens2
Oberonia caulescens1
Oberonia ensiformis4
Oberonia ensiformis5
Oberonia ensiformis6
Oberonia ensiformis7
Oberonia ensiformis8
Oberonia mucronata8
Oberonia ensiformis1
Oberonia mucronata9
Oberonia mucronata1
Oberonia mucronata2
Oberonia mucronata3
Oberonia mucronata6
Oberonia mucronata4
Oberonia mucronata5
Oberonia mucronata7
Oberonia ensiformis3
Oberonia ensiformis2
Epipactis mairei2
Epipactis helleborine4
Epipactis helleborine3
Epipactis helleborine1
Epipactis helleborine2
Epipactis helleborine5
Epipactis mairei1

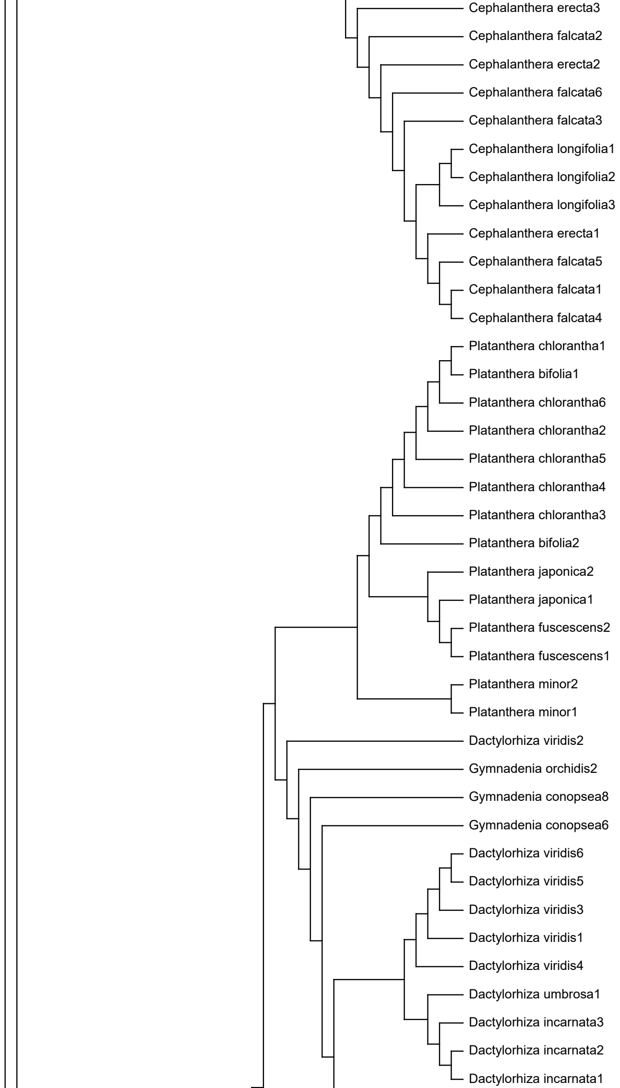
Cephalanthera erecta3
Cephalanthera falcata2
Cephalanthera erecta2
Cephalanthera falcata6
Cephalanthera falcata3
Cephalanthera longifolia1
Cephalanthera longifolia2
Cephalanthera longifolia3
Cephalanthera erecta1
Cephalanthera falcata5
Cephalanthera falcata1
Cephalanthera falcata4
Platanthera chlorantha1
Platanthera bifolia1
Platanthera chlorantha6
Platanthera chlorantha2
Platanthera chlorantha5
Platanthera chlorantha4
Platanthera chlorantha3
Platanthera bifolia2
Platanthera japonica2
Platanthera japonica1
Platanthera fuscescens2
Platanthera fuscescens1
Platanthera minor2
Platanthera minor1
Dactylorhiza viridis2
Gymnadenia orchidis2
Gymnadenia conopsea8
Gymnadenia conopsea6
Dactylorhiza viridis6
Dactylorhiza viridis5
Dactylorhiza viridis3
Dactylorhiza viridis1
Dactylorhiza viridis4
Dactylorhiza umbrosa1
Dactylorhiza incarnata3
Dactylorhiza incarnata2
Dactylorhiza incarnata1

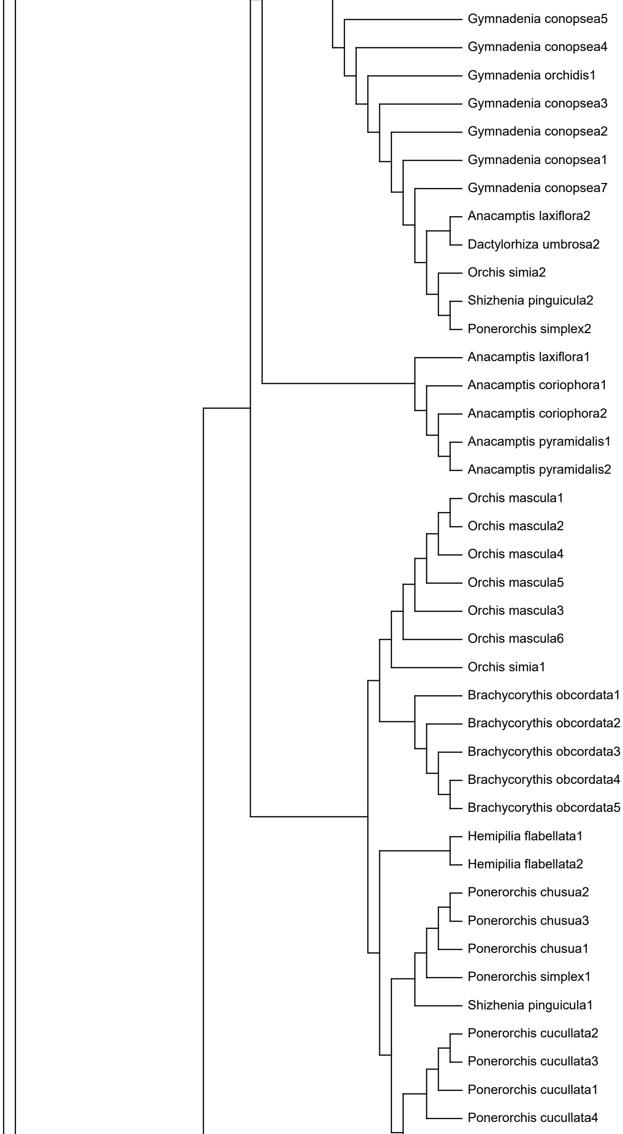
Gymnadenia conopsea5
Gymnadenia conopsea4
Gymnadenia orchidis1
Gymnadenia conopsea3
Gymnadenia conopsea2
Gymnadenia conopsea1
Gymnadenia conopsea7
Anacamptis laxiflora2
Dactylorhiza umbrosa2
Orchis simia2
Shizhenia pinguicula2
Ponerorchis simplex2
Anacamptis laxiflora1
Anacamptis coriophora1
Anacamptis coriophora2
Anacamptis pyramidalis1
Anacamptis pyramidalis2
Orchis mascula1
Orchis mascula2
Orchis mascula4
Orchis mascula5
Orchis mascula3
Orchis mascula6
Orchis simia1
Brachycorythis obcordata1
Brachycorythis obcordata2
Brachycorythis obcordata3
Brachycorythis obcordata4
Brachycorythis obcordata5
Hemipilia flabellata1
Hemipilia flabellata2
Ponerorchis chusua2
Ponerorchis chusua3
Ponerorchis chusua1
Ponerorchis simplex1
Shizhenia pinguicula1
Ponerorchis cucullata2
Ponerorchis cucullata3
Ponerorchis cucullata1
Ponerorchis cucullata4

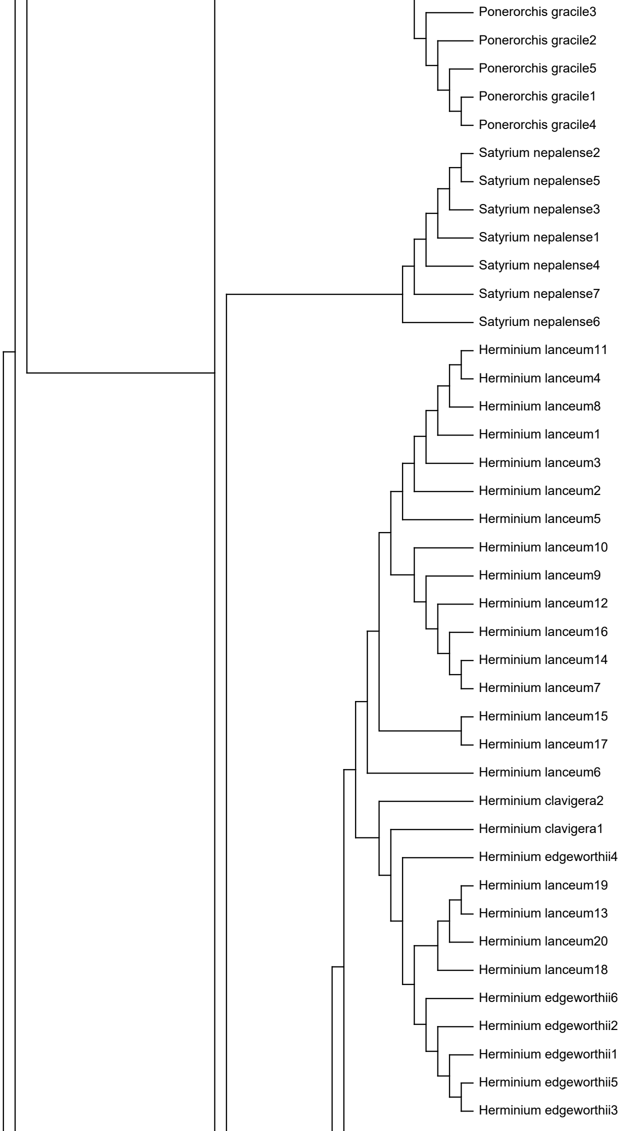
Ponerorchis gracile3
Ponerorchis gracile2
Ponerorchis gracile5
Ponerorchis gracile1
Ponerorchis gracile4
Satyrium nepalense2
Satyrium nepalense5
Satyrium nepalense3
Satyrium nepalense1
Satyrium nepalense4
Satyrium nepalense7
Satyrium nepalense6
Herminium lanceum11
Herminium lanceum4
Herminium lanceum8
Herminium lanceum1
Herminium lanceum3
Herminium lanceum2
Herminium lanceum5
Herminium lanceum10
Herminium lanceum9
Herminium lanceum12
Herminium lanceum16
Herminium lanceum14
Herminium lanceum7
Herminium lanceum15
Herminium lanceum17
Herminium lanceum6
Herminium clavigera2
Herminium clavigera1
Herminium edgeworthii4
Herminium lanceum19
Herminium lanceum13
Herminium lanceum20
Herminium lanceum18
Herminium edgeworthii6
Herminium edgeworthii2
Herminium edgeworthii1
Herminium edgeworthii5
Herminium edgeworthii3

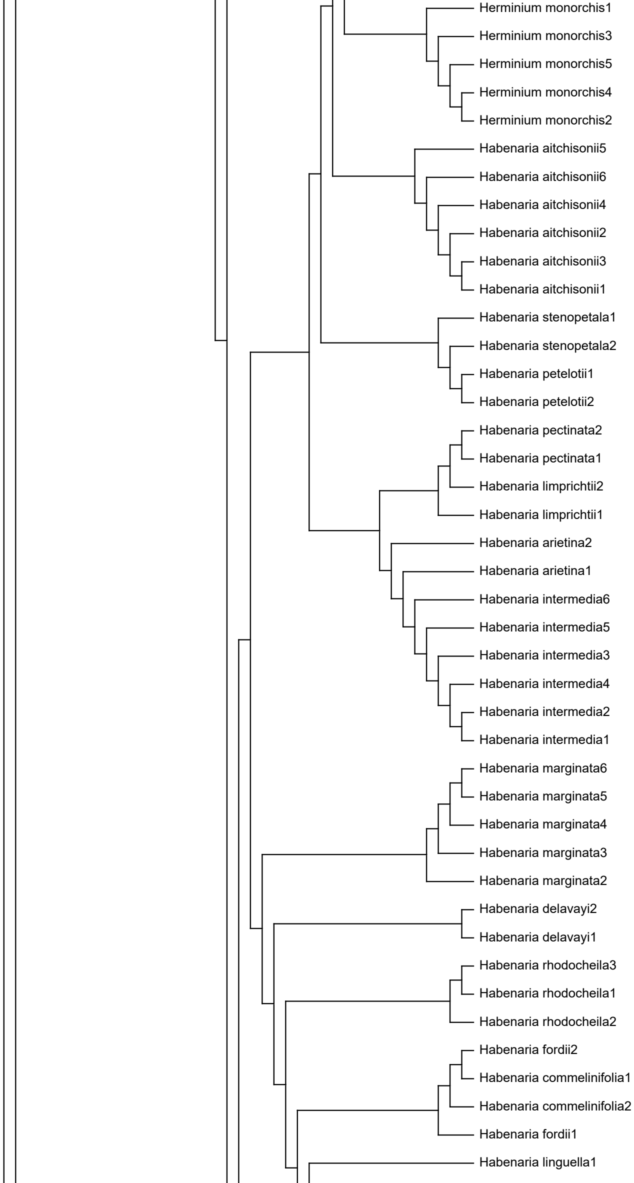

Herminium monorchis1
Herminium monorchis3
Herminium monorchis5
Herminium monorchis4
Herminium monorchis2
Habenaria aitchisonii5
Habenaria aitchisonii6
Habenaria aitchisonii4
Habenaria aitchisonii2
Habenaria aitchisonii3
Habenaria aitchisonii1
Habenaria stenopetala1
Habenaria stenopetala2
Habenaria petelotii1
Habenaria petelotii2
Habenaria pectinata2
Habenaria pectinata1
Habenaria limprichtii2
Habenaria limprichtii1
Habenaria arietina2
Habenaria arietina1
Habenaria intermedia6
Habenaria intermedia5
Habenaria intermedia3
Habenaria intermedia4
Habenaria intermedia2
Habenaria intermedia1
Habenaria marginata6
Habenaria marginata5
Habenaria marginata4
Habenaria marginata3
Habenaria marginata2
Habenaria delavayi2
Habenaria delavayi1
Habenaria rhodocheila3
Habenaria rhodocheila1
Habenaria rhodocheila2
Habenaria fordii2
Habenaria commelinifolia1
Habenaria commelinifolia2
Habenaria fordii1
Habenaria linguella1

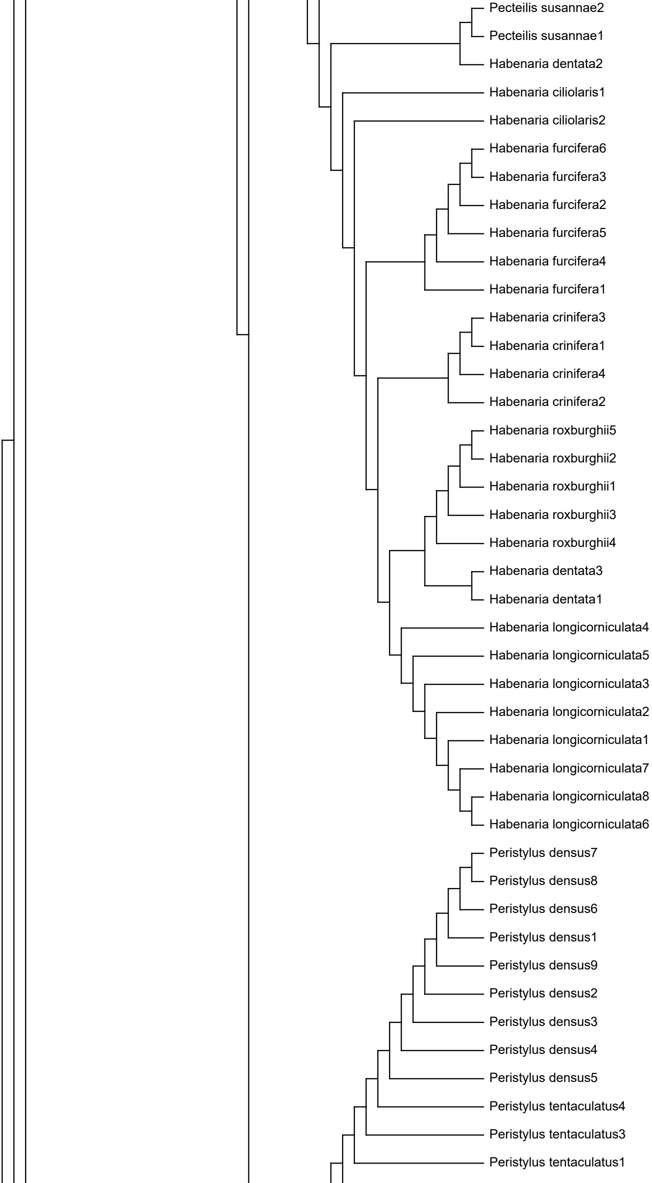

Pecteilis susannae2
Pecteilis susannae1
Habenaria dentata2
Habenaria ciliolaris1
Habenaria ciliolaris2
Habenaria furcifera6
Habenaria furcifera3
Habenaria furcifera2
Habenaria furcifera5
Habenaria furcifera4
Habenaria furcifera1
Habenaria crinifera3
Habenaria crinifera1
Habenaria crinifera4
Habenaria crinifera2
Habenaria roxburghii5
Habenaria roxburghii2
Habenaria roxburghii1
Habenaria roxburghii3
Habenaria roxburghii4
Habenaria dentata3
Habenaria dentata1
Habenaria longicorniculata4
Habenaria longicorniculata5
Habenaria longicorniculata3
Habenaria longicorniculata2
Habenaria longicorniculata1
Habenaria longicorniculata7
Habenaria longicorniculata8
Habenaria longicorniculata6
Peristylus densus7
Peristylus densus8
Peristylus densus6
Peristylus densus1
Peristylus densus9
Peristylus densus2
Peristylus densus3
Peristylus densus4
Peristylus densus5
Peristylus tentaculatus4
Peristylus tentaculatus3
Peristylus tentaculatus1

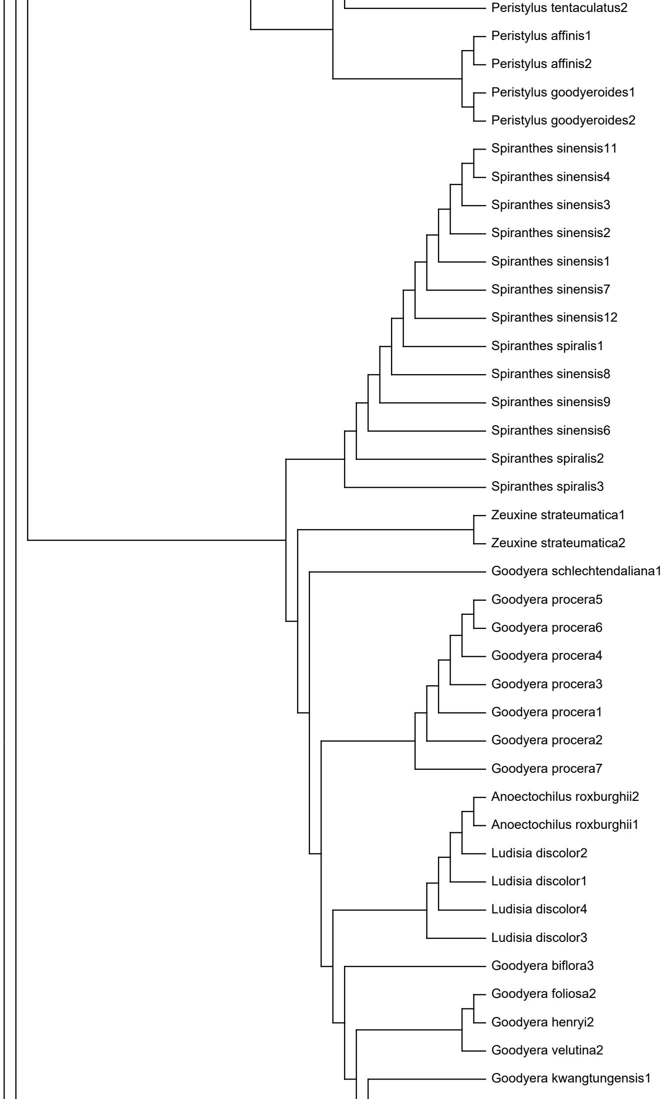
Peristylus tentaculatus2
Peristylus affinis1
Peristylus affinis2
Peristylus goodyeroides1
Peristylus goodyeroides2
Spiranthes sinensis11
Spiranthes sinensis4
Spiranthes sinensis3
Spiranthes sinensis2
Spiranthes sinensis1
Spiranthes sinensis7
Spiranthes sinensis12
Spiranthes spiralis1
Spiranthes sinensis8
Spiranthes sinensis9
Spiranthes sinensis6
Spiranthes spiralis2
Spiranthes spiralis3
Zeuxine strateumatica1
Zeuxine strateumatica2
Goodyera schlechtendaliana1
Goodyera procera5
Goodyera procera6
Goodyera procera4
Goodyera procera3
Goodyera procera1
Goodyera procera2
Goodyera procera7
Anoectochilus roxburghii2
Anoectochilus roxburghii1
Ludisia discolor2
Ludisia discolor1
Ludisia discolor4
Ludisia discolor3
Goodyera biflora3
Goodyera foliosa2
Goodyera henryi2
Goodyera velutina2
Goodyera kwangtungensis1

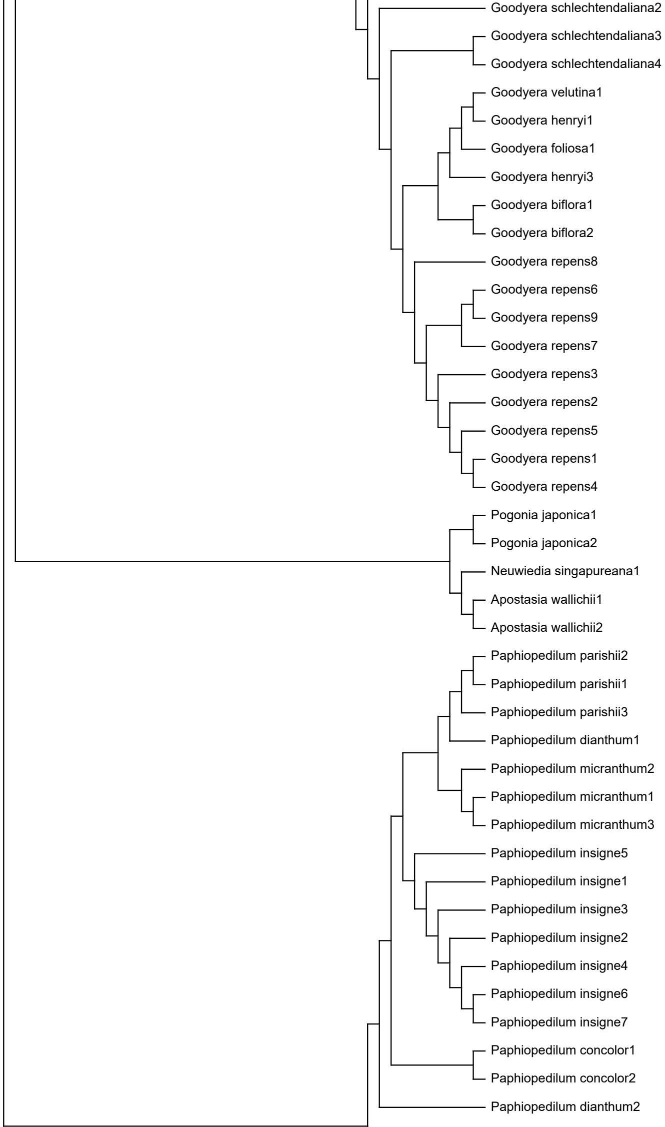
Goodyera schlechtendaliana2
Goodyera schlechtendaliana3
Goodyera schlechtendaliana4
Goodyera velutina1
Goodyera henryi1
Goodyera foliosa1
Goodyera henryi3
Goodyera biflora1
Goodyera biflora2
Goodyera repens8
Goodyera repens6
Goodyera repens9
Goodyera repens7
Goodyera repens3
Goodyera repens2
Goodyera repens5
Goodyera repens1
Goodyera repens4
Pogonia japonica1
Pogonia japonica2
Neuwiedia singapureana1
Apostasia wallichii1
Apostasia wallichii2
Paphiopedilum parishii2
Paphiopedilum parishii1
Paphiopedilum parishii3
Paphiopedilum dianthum1
Paphiopedilum micranthum2
Paphiopedilum micranthum1
Paphiopedilum micranthum3
Paphiopedilum insigne5
Paphiopedilum insigne1
Paphiopedilum insigne3
Paphiopedilum insigne2
Paphiopedilum insigne4
Paphiopedilum insigne6
Paphiopedilum insigne7
Paphiopedilum concolor1
Paphiopedilum concolor2
Paphiopedilum dianthum2

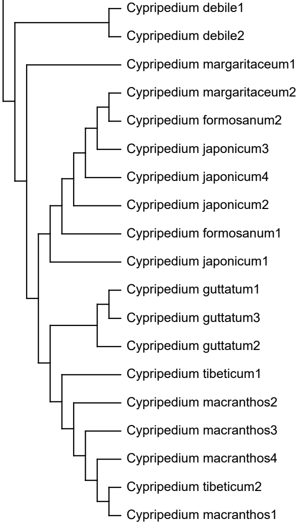
Cypripedium debile1
Cypripedium debile2
Cypripedium margaritaceum1
Cypripedium margaritaceum2
Cypripedium formosanum2
Cypripedium japonicum3
Cypripedium japonicum4
Cypripedium japonicum2
Cypripedium formosanum1
Cypripedium japonicum1
Cypripedium guttatum1
Cypripedium guttatum3
Cypripedium guttatum2
Cypripedium tibeticum1
Cypripedium macranthos2
Cypripedium macranthos3
Cypripedium macranthos4
Cypripedium tibeticum2
Cypripedium macranthos1
