# Supplementary material for: DNA barcoding of medicinal orchids in Asia
Source: Sci Rep. 2021 Dec 8;11:23651. doi: 10.1038/s41598-021-03025-0 (PMC8654824; doi:10.1038/s41598-021-03025-0)
Supplement: Supplementary file 9 — Supplementary Figure S9. [file 41598_2021_3025_MOESM9_ESM.pdf]

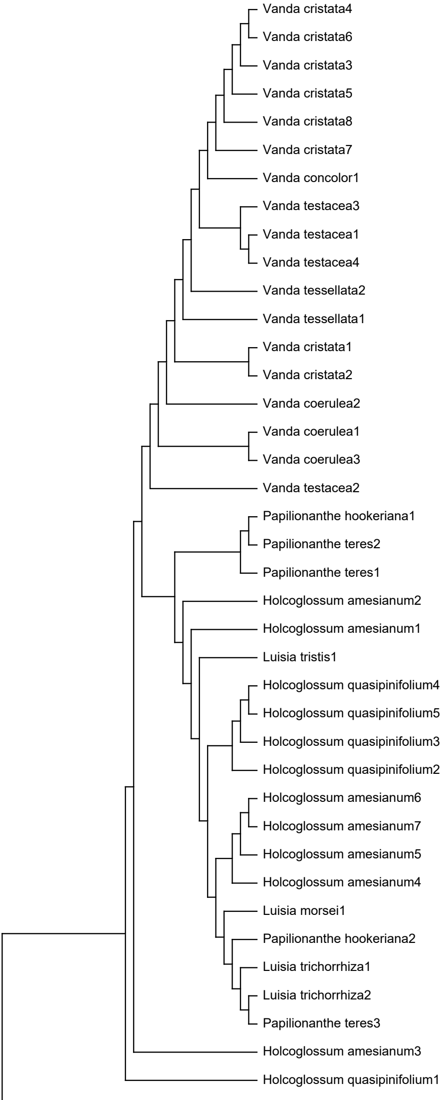
Vanda cristata4
Vanda cristata6
Vanda cristata3
Vanda cristata5
Vanda cristata8
Vanda cristata7
Vanda concolor1
Vanda testacea3
Vanda testacea1
Vanda testacea4
Vanda tessellata2
Vanda tessellata1
Vanda cristata1
Vanda cristata2
Vanda coerulea2
Vanda coerulea1
Vanda coerulea3
Vanda testacea2
Papilionanthe hookeriana1
Papilionanthe teres2
Papilionanthe teres1
Holcoglossum amesianum2
Holcoglossum amesianum1
Luisia tristis1
Holcoglossum quasipinifolium4
Holcoglossum quasipinifolium5
Holcoglossum quasipinifolium3
Holcoglossum quasipinifolium2
Holcoglossum amesianum6
Holcoglossum amesianum7
Holcoglossum amesianum5
Holcoglossum amesianum4
Luisia morsei1
Papilionanthe hookeriana2
Luisia trichorrhiza1
Luisia trichorrhiza2
Papilionanthe teres3
Holcoglossum amesianum3
Holcoglossum quasipinifolium1

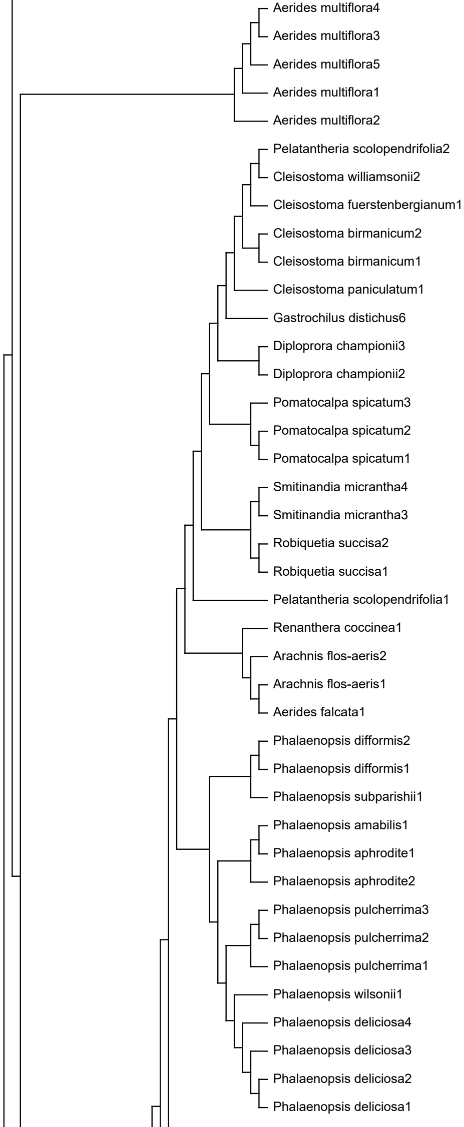
Aerides multiflora4
Aerides multiflora3
Aerides multiflora5
Aerides multiflora1
Aerides multiflora2
Pelatantheria scolopendrifolia2
Cleisostoma williamsonii2
Cleisostoma fuerstenbergianum1
Cleisostoma birmanicum2
Cleisostoma birmanicum1
Cleisostoma paniculatum1
Gastrochilus distichus6
Diploprora championii3
Diploprora championii2
Pomatocalpa spicatum3
Pomatocalpa spicatum2
Pomatocalpa spicatum1
Smitinandia micrantha4
Smitinandia micrantha3
Robiquetia succisa2
Robiquetia succisa1
Pelatantheria scolopendrifolia1
Renanthera coccinea1
Arachnis flos-aeris2
Arachnis flos-aeris1
Aerides falcata1
Phalaenopsis difformis2
Phalaenopsis difformis1
Phalaenopsis subparishii1
Phalaenopsis amabilis1
Phalaenopsis aphrodite1
Phalaenopsis aphrodite2
Phalaenopsis pulcherrima3
Phalaenopsis pulcherrima2
Phalaenopsis pulcherrima1
Phalaenopsis wilsonii1
Phalaenopsis deliciosa4
Phalaenopsis deliciosa3
Phalaenopsis deliciosa2
Phalaenopsis deliciosa1

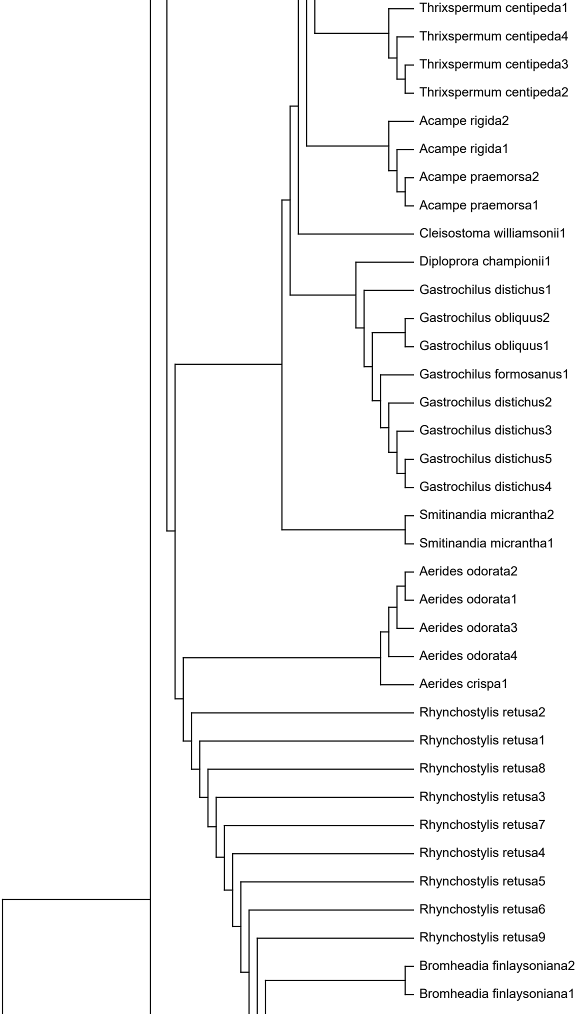
Thrixspermum centipeda1
Thrixspermum centipeda4
Thrixspermum centipeda3
Thrixspermum centipeda2
Acampe rigida2
Acampe rigida1
Acampe praemorsa2
Acampe praemorsa1
Cleisostoma williamsonii1
Diploprora championii1
Gastrochilus distichus1
Gastrochilus obliquus2
Gastrochilus obliquus1
Gastrochilus formosanus1
Gastrochilus distichus2
Gastrochilus distichus3
Gastrochilus distichus5
Gastrochilus distichus4
Smitinandia micrantha2
Smitinandia micrantha1
Aerides odorata2
Aerides odorata1
Aerides odorata3
Aerides odorata4
Aerides crispa1
Rhynchostylis retusa2
Rhynchostylis retusa1
Rhynchostylis retusa8
Rhynchostylis retusa3
Rhynchostylis retusa7
Rhynchostylis retusa4
Rhynchostylis retusa5
Rhynchostylis retusa6
Rhynchostylis retusa9
Bromheadia finlaysoniana2
Bromheadia finlaysoniana1

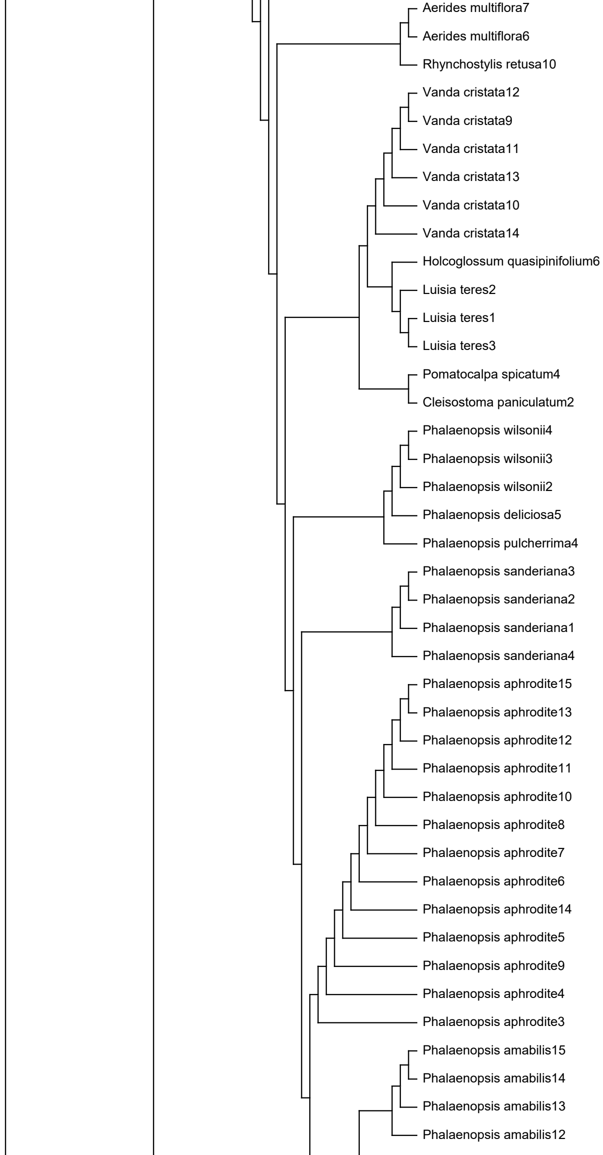
Aerides multiflora7
Aerides multiflora6
Rhynchostylis retusa10
Vanda cristata12
Vanda cristata9
Vanda cristata11
Vanda cristata13
Vanda cristata10
Vanda cristata14
Holcoglossum quasipinifolium6
Luisia teres2
Luisia teres1
Luisia teres3
Pomatocalpa spicatum4
Cleisostoma paniculatum2
Phalaenopsis wilsonii4
Phalaenopsis wilsonii3
Phalaenopsis wilsonii2
Phalaenopsis deliciosa5
Phalaenopsis pulcherrima4
Phalaenopsis sanderiana3
Phalaenopsis sanderiana2
Phalaenopsis sanderiana1
Phalaenopsis sanderiana4
Phalaenopsis aphrodite15
Phalaenopsis aphrodite13
Phalaenopsis aphrodite12
Phalaenopsis aphrodite11
Phalaenopsis aphrodite10
Phalaenopsis aphrodite8
Phalaenopsis aphrodite7
Phalaenopsis aphrodite6
Phalaenopsis aphrodite14
Phalaenopsis aphrodite5
Phalaenopsis aphrodite9
Phalaenopsis aphrodite4
Phalaenopsis aphrodite3
Phalaenopsis amabilis15
Phalaenopsis amabilis14
Phalaenopsis amabilis13
Phalaenopsis amabilis12

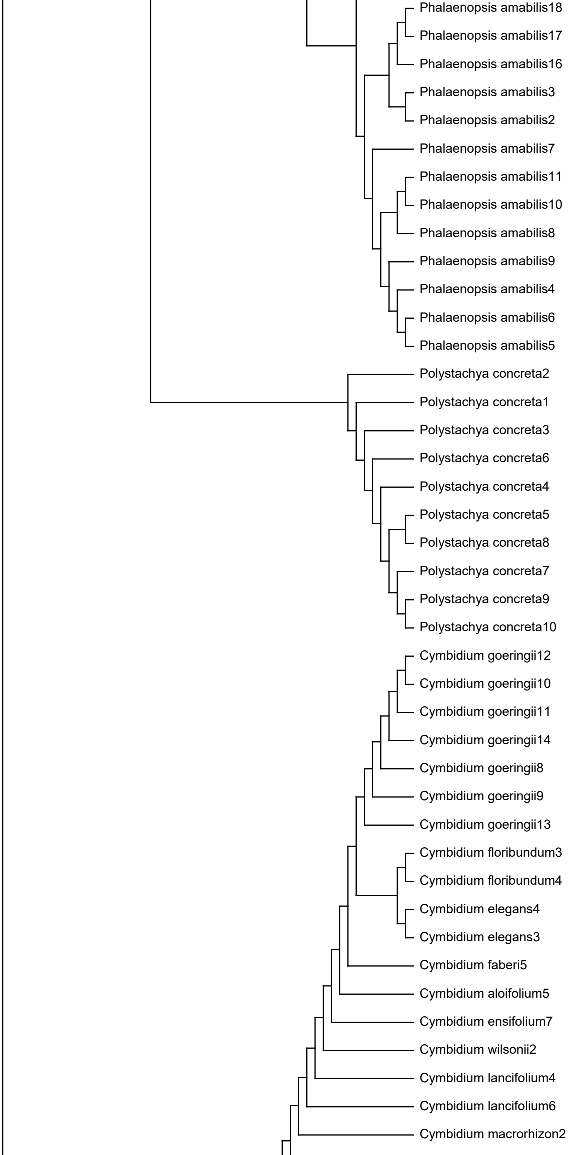
Phalaenopsis amabilis18
Phalaenopsis amabilis17
Phalaenopsis amabilis16
Phalaenopsis amabilis3
Phalaenopsis amabilis2
Phalaenopsis amabilis7
Phalaenopsis amabilis11
Phalaenopsis amabilis10
Phalaenopsis amabilis8
Phalaenopsis amabilis9
Phalaenopsis amabilis4
Phalaenopsis amabilis6
Phalaenopsis amabilis5
Polystachya concreta2
Polystachya concreta1
Polystachya concreta3
Polystachya concreta6
Polystachya concreta4
Polystachya concreta5
Polystachya concreta8
Polystachya concreta7
Polystachya concreta9
Polystachya concreta10
Cymbidium goeringii12
Cymbidium goeringii10
Cymbidium goeringii11
Cymbidium goeringii14
Cymbidium goeringii8
Cymbidium goeringii9
Cymbidium goeringii13
Cymbidium floribundum3
Cymbidium floribundum4
Cymbidium elegans4
Cymbidium elegans3
Cymbidium faberi5
Cymbidium aloifolium5
Cymbidium ensifolium7
Cymbidium wilsonii2
Cymbidium lancifolium4
Cymbidium lancifolium6
Cymbidium macrorhizon2

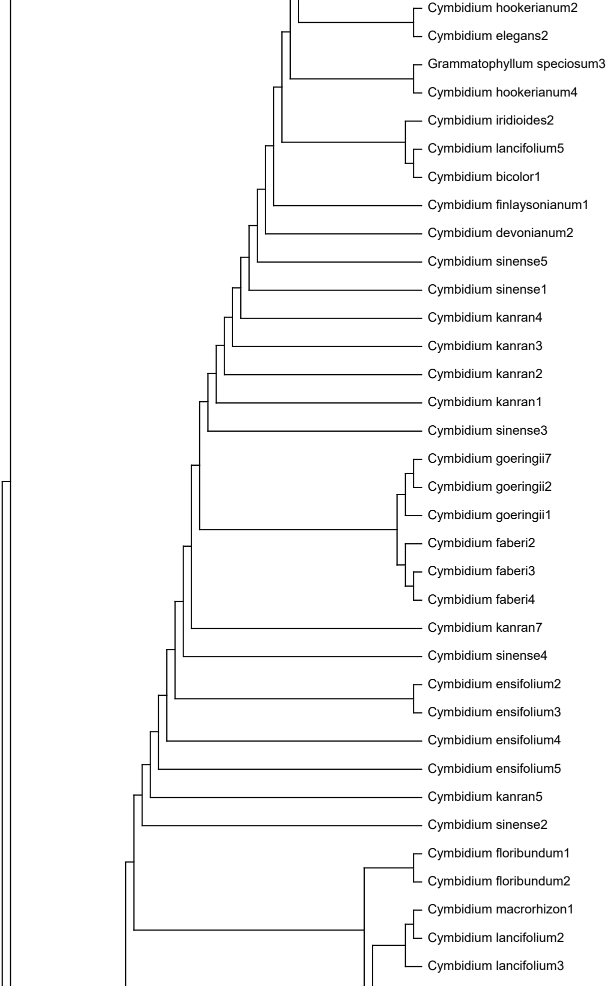

Cymbidium hookerianum2
Cymbidium elegans2
Grammatophyllum speciosum3
Cymbidium hookerianum4
Cymbidium iridioides2
Cymbidium lancifolium5
Cymbidium bicolor1
Cymbidium finlaysonianum1
Cymbidium devonianum2
Cymbidium sinense5
Cymbidium sinense1
Cymbidium kanran4
Cymbidium kanran3
Cymbidium kanran2
Cymbidium kanran1
Cymbidium sinense3
Cymbidium goeringii7
Cymbidium goeringii2
Cymbidium goeringii1
Cymbidium faberi2
Cymbidium faberi3
Cymbidium faberi4
Cymbidium kanran7
Cymbidium sinense4
Cymbidium ensifolium2
Cymbidium ensifolium3
Cymbidium ensifolium4
Cymbidium ensifolium5
Cymbidium kanran5
Cymbidium sinense2
Cymbidium floribundum1
Cymbidium floribundum2
Cymbidium macrorhizon1
Cymbidium lancifolium2
Cymbidium lancifolium3

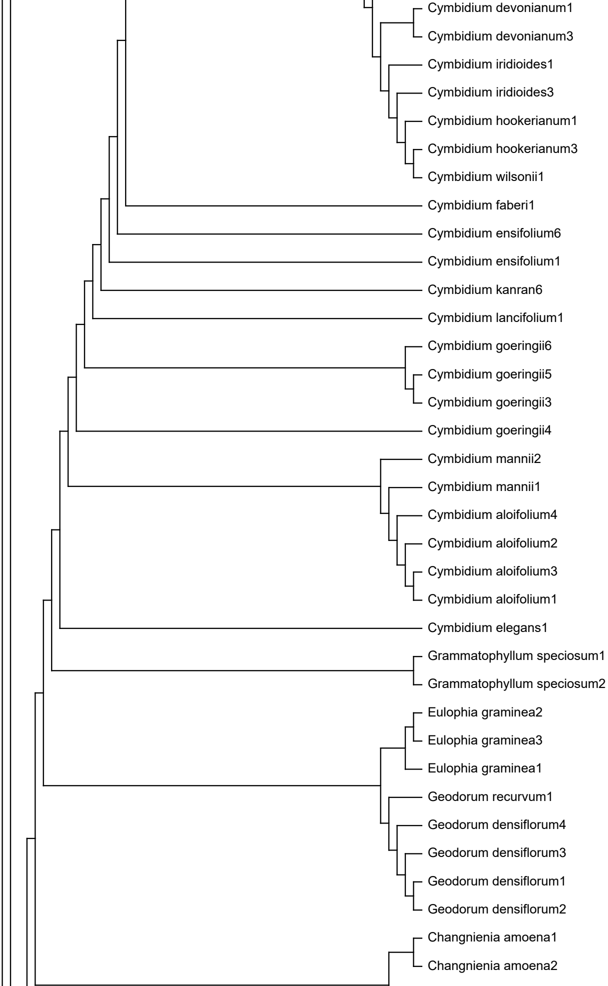
Cymbidium devonianum1
Cymbidium devonianum3
Cymbidium iridioides1
Cymbidium iridioides3
Cymbidium hookerianum1
Cymbidium hookerianum3
Cymbidium wilsonii1
Cymbidium faberi1
Cymbidium ensifolium6
Cymbidium ensifolium1
Cymbidium kanran6
Cymbidium lancifolium1
Cymbidium goeringii6
Cymbidium goeringii5
Cymbidium goeringii3
Cymbidium goeringii4
Cymbidium mannii2
Cymbidium mannii1
Cymbidium aloifolium4
Cymbidium aloifolium2
Cymbidium aloifolium3
Cymbidium aloifolium1
Cymbidium elegans1
Grammatophyllum speciosum1
Grammatophyllum speciosum2
Eulophia graminea2
Eulophia graminea3
Eulophia graminea1
Geodorum recurvum1
Geodorum densiflorum4
Geodorum densiflorum3
Geodorum densiflorum1
Geodorum densiflorum2
Changnienia amoena1
Changnienia amoena2

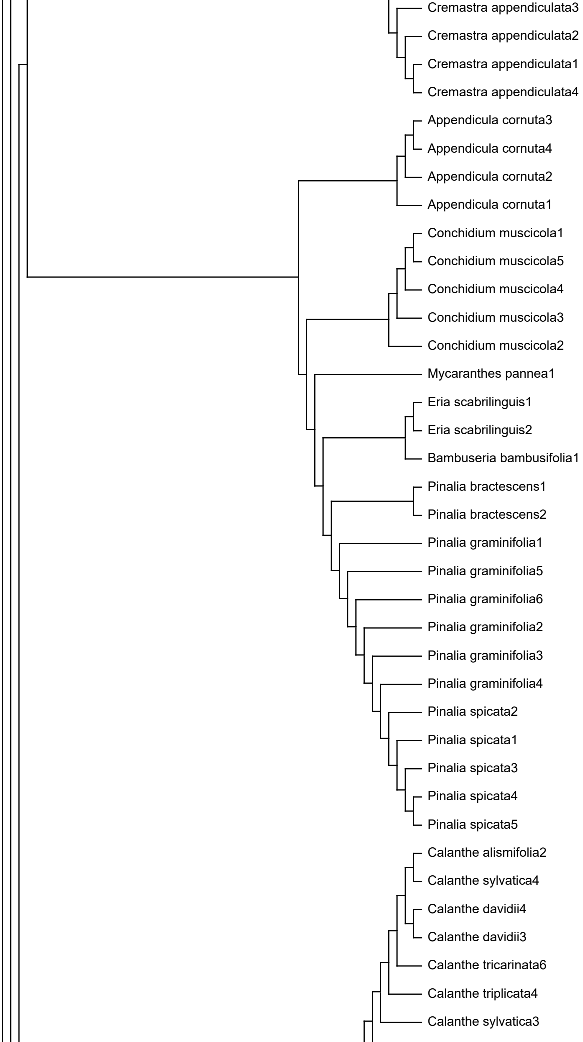
Cremastra appendiculata3
Cremastra appendiculata2
Cremastra appendiculata1
Cremastra appendiculata4
Appendicula cornuta3
Appendicula cornuta4
Appendicula cornuta2
Appendicula cornuta1
Conchidium muscicola1
Conchidium muscicola5
Conchidium muscicola4
Conchidium muscicola3
Conchidium muscicola2
Mycaranthes pannea1
Eria scabrilinguis1
Eria scabrilinguis2
Bambuseria bambusifolia1
Pinalia bractescens1
Pinalia bractescens2
Pinalia graminifolia1
Pinalia graminifolia5
Pinalia graminifolia6
Pinalia graminifolia2
Pinalia graminifolia3
Pinalia graminifolia4
Pinalia spicata2
Pinalia spicata1
Pinalia spicata3
Pinalia spicata4
Pinalia spicata5
Calanthe alismifolia2
Calanthe sylvatica4
Calanthe davidii4
Calanthe davidii3
Calanthe tricarinata6
Calanthe triplicata4
Calanthe sylvatica3

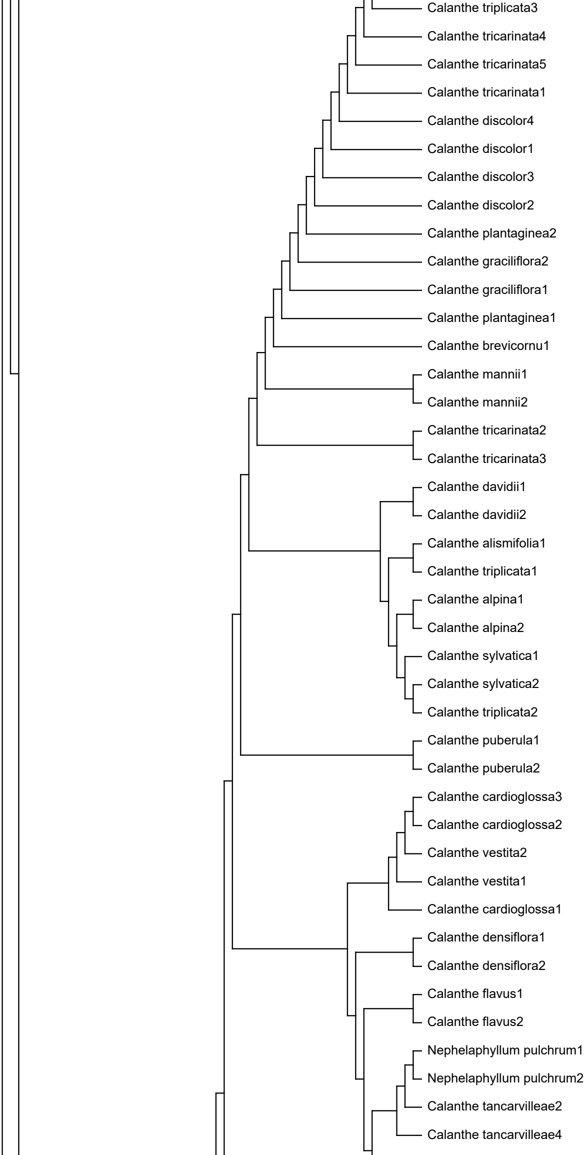
Calanthe triplicata3
Calanthe tricarinata4
Calanthe tricarinata5
Calanthe tricarinata1
Calanthe discolor4
Calanthe discolor1
Calanthe discolor3
Calanthe discolor2
Calanthe plantaginea2
Calanthe graciliflora2
Calanthe graciliflora1
Calanthe plantaginea1
Calanthe brevicornu1
Calanthe mannii1
Calanthe mannii2
Calanthe tricarinata2
Calanthe tricarinata3
Calanthe davidii1
Calanthe davidii2
Calanthe alismifolia1
Calanthe triplicata1
Calanthe alpina1
Calanthe alpina2
Calanthe sylvatica1
Calanthe sylvatica2
Calanthe triplicata2
Calanthe puberula1
Calanthe puberula2
Calanthe cardioglossa3
Calanthe cardioglossa2
Calanthe vestita2
Calanthe vestita1
Calanthe cardioglossa1
Calanthe densiflora1
Calanthe densiflora2
Calanthe flavus1
Calanthe flavus2
Nephelaphyllum pulchrum1
Nephelaphyllum pulchrum2
Calanthe tancarvilleae2
Calanthe tancarvilleae4

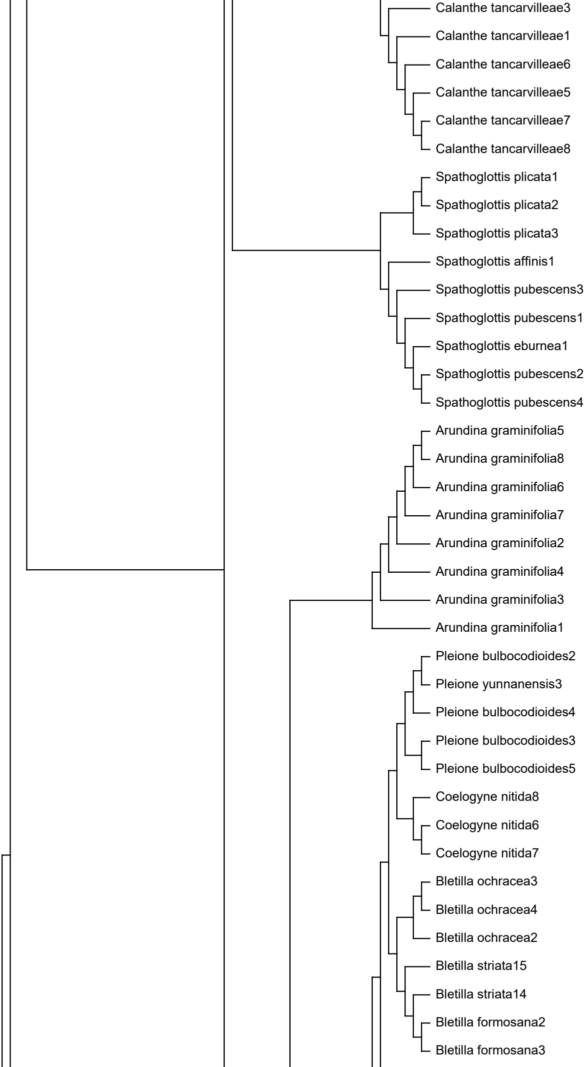
Calanthe tancarvilleae3
Calanthe tancarvilleae1
Calanthe tancarvilleae6
Calanthe tancarvilleae5
Calanthe tancarvilleae7
Calanthe tancarvilleae8
Spathoglottis plicata1
Spathoglottis plicata2
Spathoglottis plicata3
Spathoglottis affinis1
Spathoglottis pubescens3
Spathoglottis pubescens1
Spathoglottis eburnea1
Spathoglottis pubescens2
Spathoglottis pubescens4
Arundina graminifolia5
Arundina graminifolia8
Arundina graminifolia6
Arundina graminifolia7
Arundina graminifolia2
Arundina graminifolia4
Arundina graminifolia3
Arundina graminifolia1
Pleione bulbocodioides2
Pleione yunnanensis3
Pleione bulbocodioides4
Pleione bulbocodioides3
Pleione bulbocodioides5
Coelogyne nitida8
Coelogyne nitida6
Coelogyne nitida7
Bletilla ochracea3
Bletilla ochracea4
Bletilla ochracea2
Bletilla striata15
Bletilla striata14
Bletilla formosana2
Bletilla formosana3

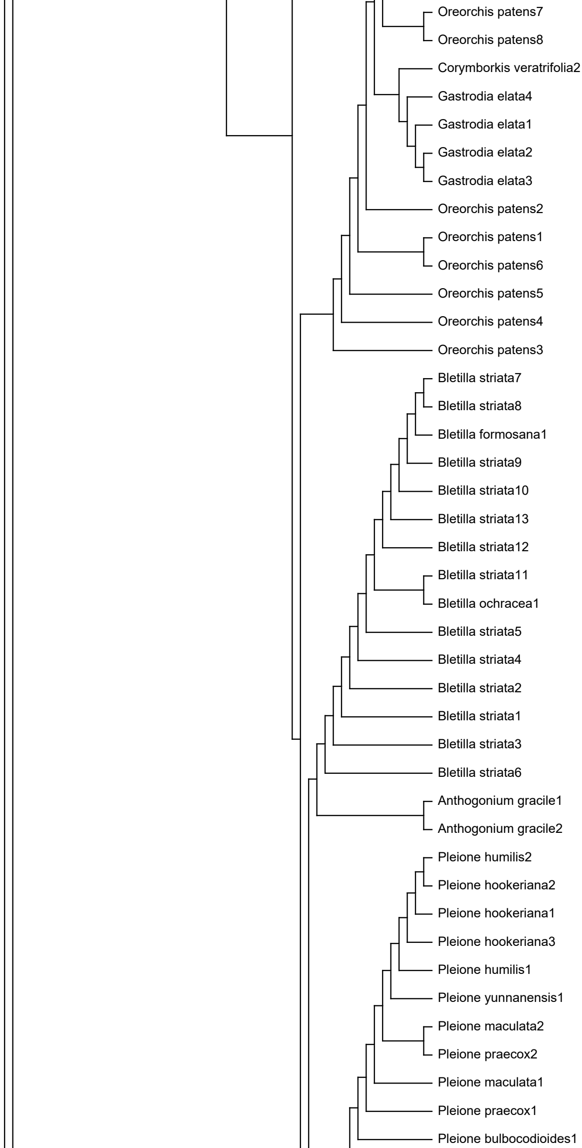

Oreorchis patens7
Oreorchis patens8
Corymborkis veratrifolia2
Gastrodia elata4
Gastrodia elata1
Gastrodia elata2
Gastrodia elata3
Oreorchis patens2
Oreorchis patens1
Oreorchis patens6
Oreorchis patens5
Oreorchis patens4
Oreorchis patens3
Bletilla striata7
Bletilla striata8
Bletilla formosana1
Bletilla striata9
Bletilla striata10
Bletilla striata13
Bletilla striata12
Bletilla striata11
Bletilla ochracea1
Bletilla striata5
Bletilla striata4
Bletilla striata2
Bletilla striata1
Bletilla striata3
Bletilla striata6
Anthogonium gracile1
Anthogonium gracile2
Pleione humilis2
Pleione hookeriana2
Pleione hookeriana1
Pleione hookeriana3
Pleione humilis1
Pleione yunnanensis1
Pleione maculata2
Pleione praecox2
Pleione maculata1
Pleione praecox1
Pleione bulbocodioides1

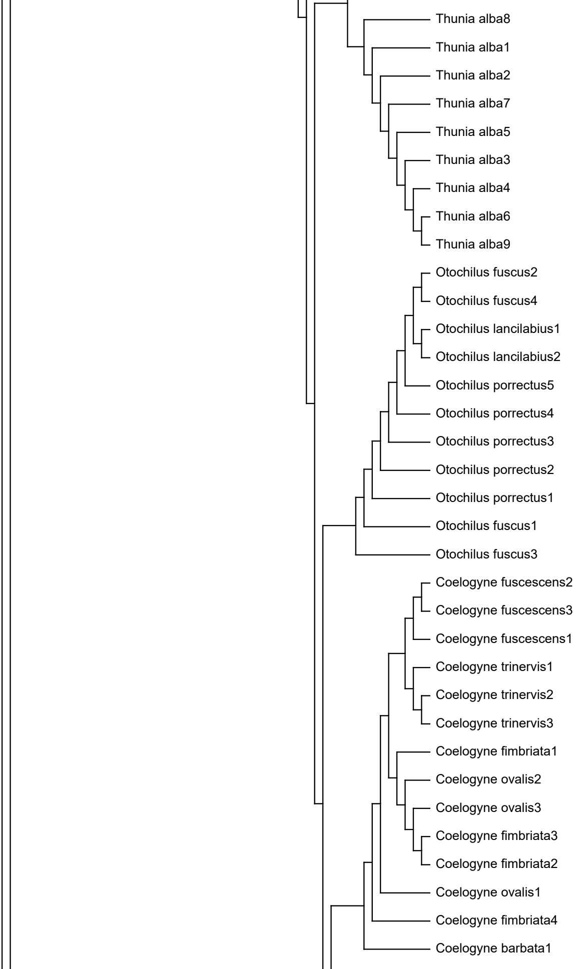
Thunia alba8
Thunia alba1
Thunia alba2
Thunia alba7
Thunia alba5
Thunia alba3
Thunia alba4
Thunia alba6
Thunia alba9
Otochilus fuscus2
Otochilus fuscus4
Otochilus lancilabius1
Otochilus lancilabius2
Otochilus porrectus5
Otochilus porrectus4
Otochilus porrectus3
Otochilus porrectus2
Otochilus porrectus1
Otochilus fuscus1
Otochilus fuscus3
Coelogyne fuscescens2
Coelogyne fuscescens3
Coelogyne fuscescens1
Coelogyne trinervis1
Coelogyne trinervis2
Coelogyne trinervis3
Coelogyne fimbriata1
Coelogyne ovalis2
Coelogyne ovalis3
Coelogyne fimbriata3
Coelogyne fimbriata2
Coelogyne ovalis1
Coelogyne fimbriata4
Coelogyne barbata1

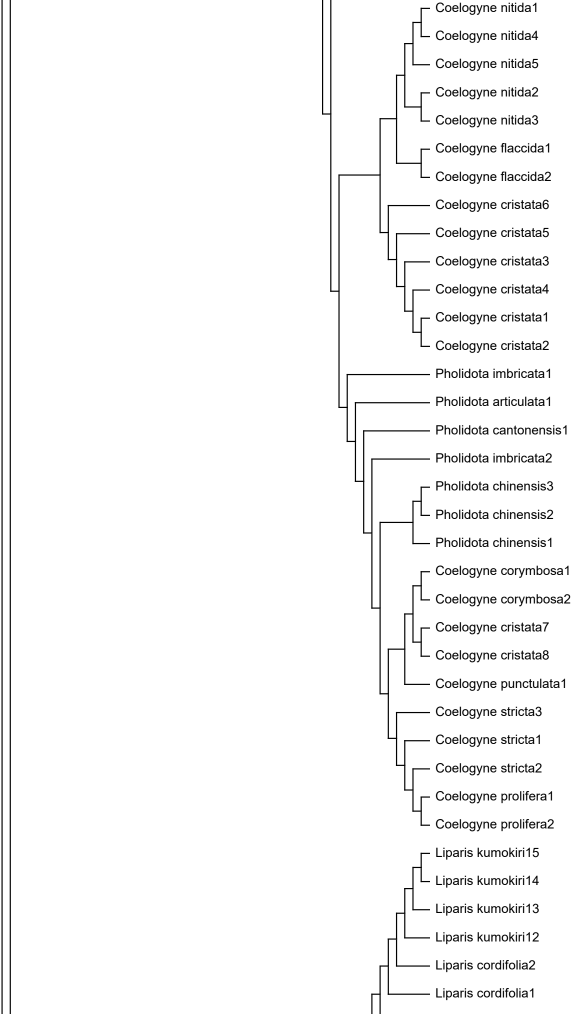
Coelogyne nitida1
Coelogyne nitida4
Coelogyne nitida5
Coelogyne nitida2
Coelogyne nitida3
Coelogyne flaccida1
Coelogyne flaccida2
Coelogyne cristata6
Coelogyne cristata5
Coelogyne cristata3
Coelogyne cristata4
Coelogyne cristata1
Coelogyne cristata2
Pholidota imbricata1
Pholidota articulata1
Pholidota cantonensis1
Pholidota imbricata2
Pholidota chinensis3
Pholidota chinensis2
Pholidota chinensis1
Coelogyne corymbosa1
Coelogyne corymbosa2
Coelogyne cristata7
Coelogyne cristata8
Coelogyne punctulata1
Coelogyne stricta3
Coelogyne stricta1
Coelogyne stricta2
Coelogyne prolifera1
Coelogyne prolifera2
Liparis kumokiri15
Liparis kumokiri14
Liparis kumokiri13
Liparis kumokiri12
Liparis cordifolia2
Liparis cordifolia1

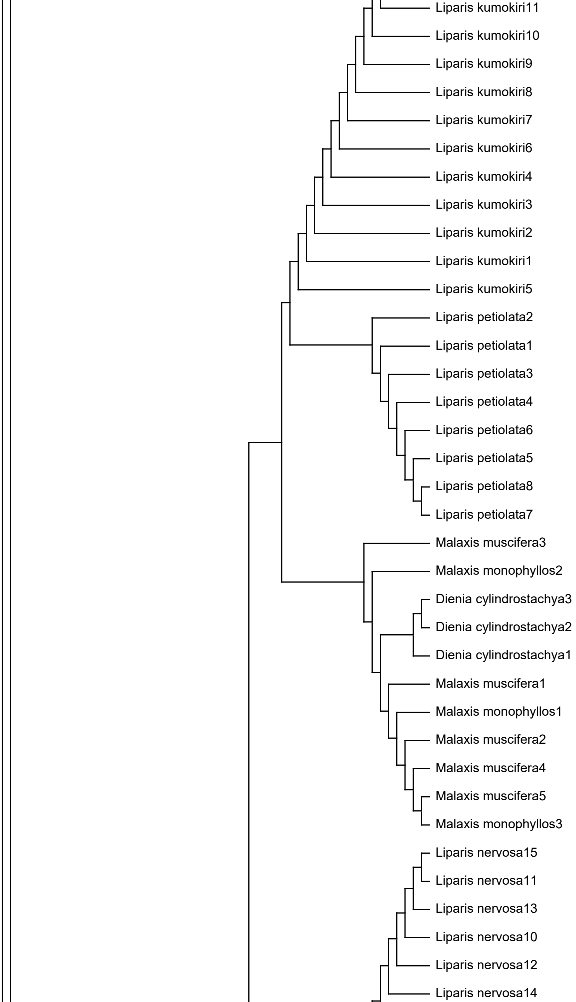

Liparis kumokiri11
Liparis kumokiri10
Liparis kumokiri9
Liparis kumokiri8
Liparis kumokiri7
Liparis kumokiri6
Liparis kumokiri4
Liparis kumokiri3
Liparis kumokiri2
Liparis kumokiri1
Liparis kumokiri5
Liparis petiolata2
Liparis petiolata1
Liparis petiolata3
Liparis petiolata4
Liparis petiolata6
Liparis petiolata5
Liparis petiolata8
Liparis petiolata7
Malaxis muscifera3
Malaxis monophyllos2
Dienia cylindrostachya3
Dienia cylindrostachya2
Dienia cylindrostachya1
Malaxis muscifera1
Malaxis monophyllos1
Malaxis muscifera2
Malaxis muscifera4
Malaxis muscifera5
Malaxis monophyllos3
Liparis nervosa15
Liparis nervosa11
Liparis nervosa13
Liparis nervosa10
Liparis nervosa12
Liparis nervosa14

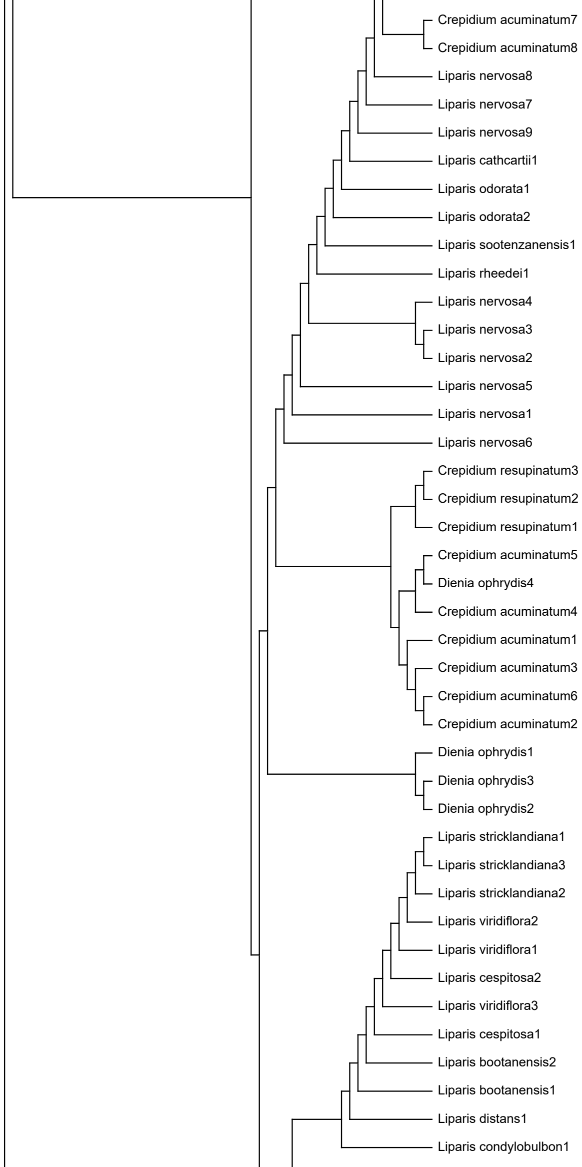
Crepidium acuminatum7
Crepidium acuminatum8
Liparis nervosa8
Liparis nervosa7
Liparis nervosa9
Liparis cathcartii1
Liparis odorata1
Liparis odorata2
Liparis sootenzanensis1
Liparis rheedei1
Liparis nervosa4
Liparis nervosa3
Liparis nervosa2
Liparis nervosa5
Liparis nervosa1
Liparis nervosa6
Crepidium resupinatum3
Crepidium resupinatum2
Crepidium resupinatum1
Crepidium acuminatum5
Dienia ophrydis4
Crepidium acuminatum4
Crepidium acuminatum1
Crepidium acuminatum3
Crepidium acuminatum6
Crepidium acuminatum2
Dienia ophrydis1
Dienia ophrydis3
Dienia ophrydis2
Liparis stricklandiana1
Liparis stricklandiana3
Liparis stricklandiana2
Liparis viridiflora2
Liparis viridiflora1
Liparis cespitosa2
Liparis viridiflora3
Liparis cespitosa1
Liparis bootanensis2
Liparis bootanensis1
Liparis distans1
Liparis condylobulbon1

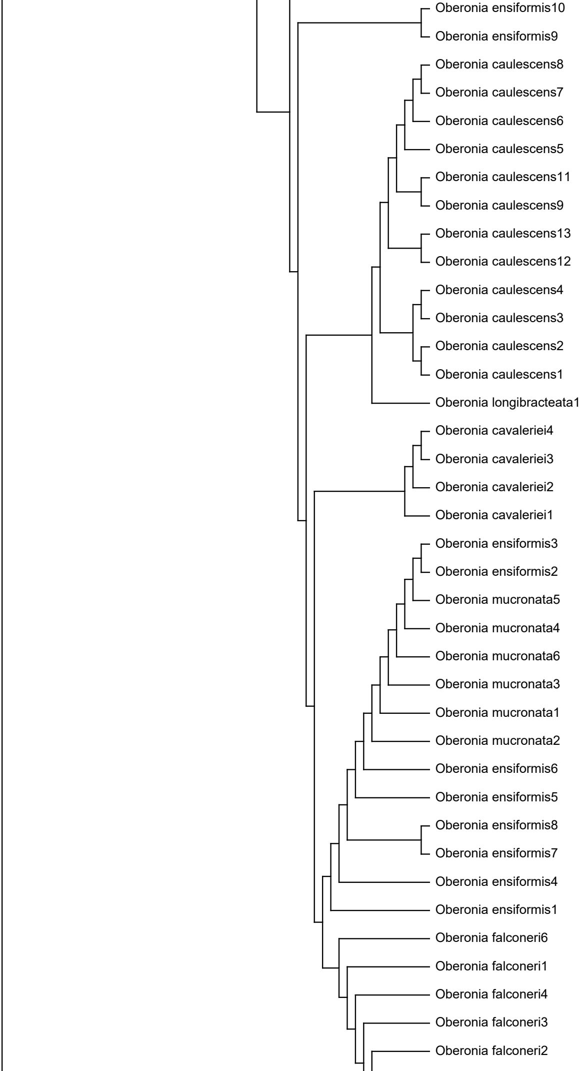
Oberonia ensiformis10
Oberonia ensiformis9
Oberonia caulescens8
Oberonia caulescens7
Oberonia caulescens6
Oberonia caulescens5
Oberonia caulescens11
Oberonia caulescens9
Oberonia caulescens13
Oberonia caulescens12
Oberonia caulescens4
Oberonia caulescens3
Oberonia caulescens2
Oberonia caulescens1
Oberonia longibracteata1
Oberonia cavaleriei4
Oberonia cavaleriei3
Oberonia cavaleriei2
Oberonia cavaleriei1
Oberonia ensiformis3
Oberonia ensiformis2
Oberonia mucronata5
Oberonia mucronata4
Oberonia mucronata6
Oberonia mucronata3
Oberonia mucronata1
Oberonia mucronata2
Oberonia ensiformis6
Oberonia ensiformis5
Oberonia ensiformis8
Oberonia ensiformis7
Oberonia ensiformis4
Oberonia ensiformis1
Oberonia falconeri6
Oberonia falconeri1
Oberonia falconeri4
Oberonia falconeri3
Oberonia falconeri2

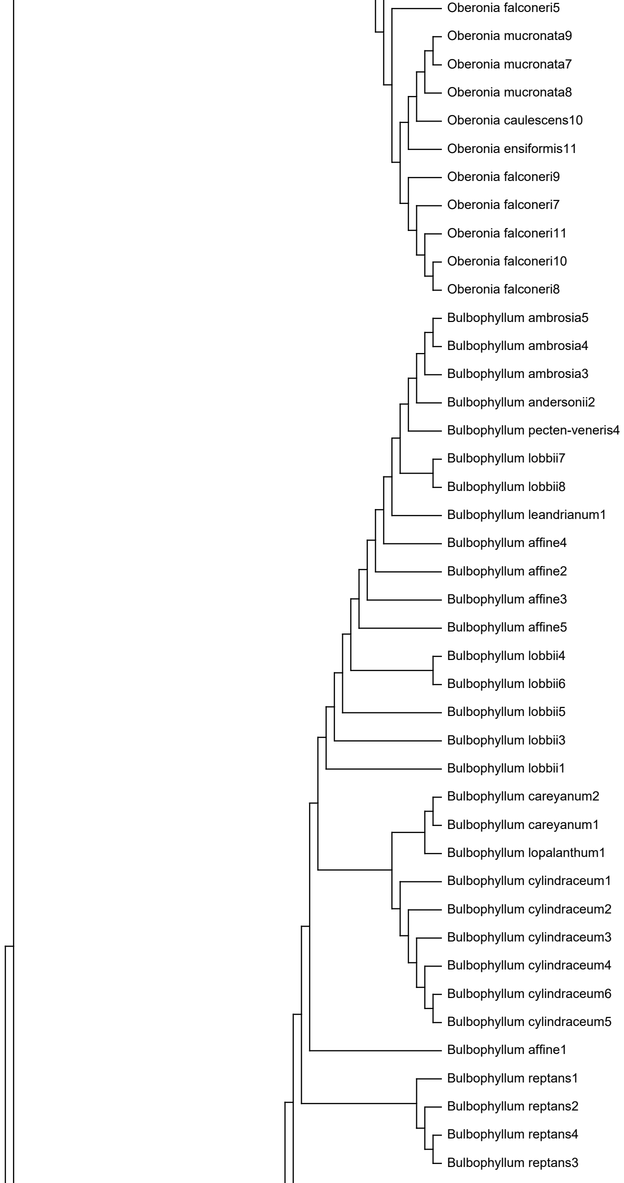
Oberonia falconeri5
Oberonia mucronata9
Oberonia mucronata7
Oberonia mucronata8
Oberonia caulescens10
Oberonia ensiformis11
Oberonia falconeri9
Oberonia falconeri7
Oberonia falconeri11
Oberonia falconeri10
Oberonia falconeri8
Bulbophyllum ambrosia5
Bulbophyllum ambrosia4
Bulbophyllum ambrosia3
Bulbophyllum andersonii2
Bulbophyllum pecten-veneris4
Bulbophyllum lobbii7
Bulbophyllum lobbii8
Bulbophyllum leandrianum1
Bulbophyllum affine4
Bulbophyllum affine2
Bulbophyllum affine3
Bulbophyllum affine5
Bulbophyllum lobbii4
Bulbophyllum lobbii6
Bulbophyllum lobbii5
Bulbophyllum lobbii3
Bulbophyllum lobbii1
Bulbophyllum careyanum2
Bulbophyllum careyanum1
Bulbophyllum lopalanthum1
Bulbophyllum cylindraceum1
Bulbophyllum cylindraceum2
Bulbophyllum cylindraceum3
Bulbophyllum cylindraceum4
Bulbophyllum cylindraceum6
Bulbophyllum cylindraceum5
Bulbophyllum affine1
Bulbophyllum reptans1
Bulbophyllum reptans2
Bulbophyllum reptans4
Bulbophyllum reptans3

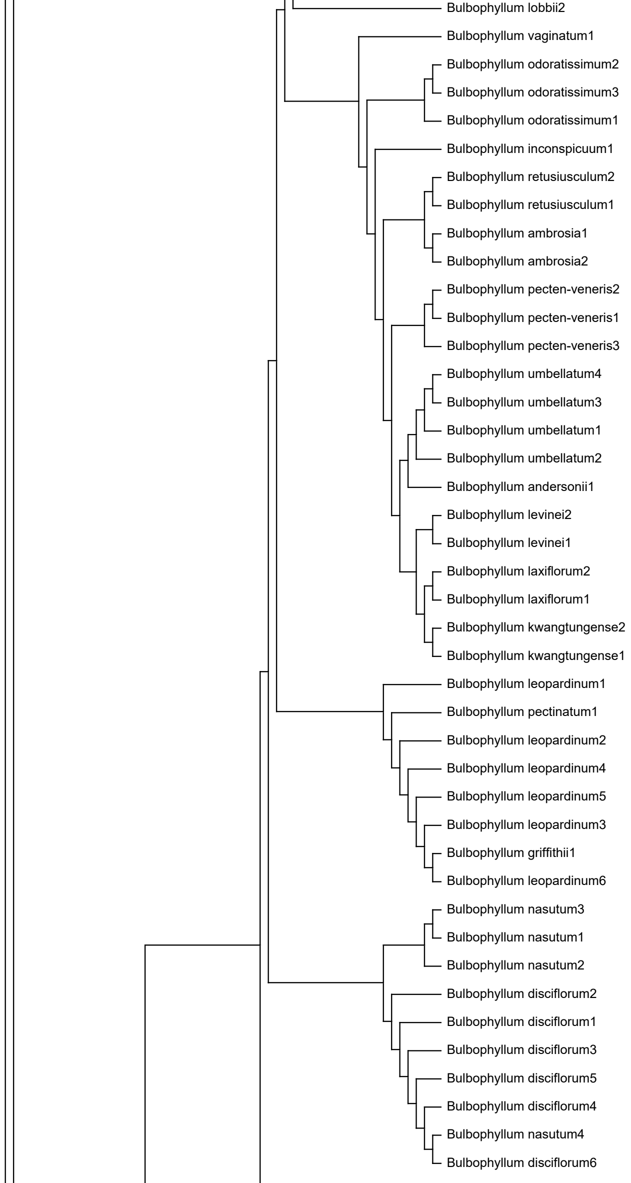
Bulbophyllum lobbii2
Bulbophyllum vaginatum1
Bulbophyllum odoratissimum2
Bulbophyllum odoratissimum3
Bulbophyllum odoratissimum1
Bulbophyllum inconspicuum1
Bulbophyllum retusiusculum2
Bulbophyllum retusiusculum1
Bulbophyllum ambrosia1
Bulbophyllum ambrosia2
Bulbophyllum pecten-veneris2
Bulbophyllum pecten-veneris1
Bulbophyllum pecten-veneris3
Bulbophyllum umbellatum4
Bulbophyllum umbellatum3
Bulbophyllum umbellatum1
Bulbophyllum umbellatum2
Bulbophyllum andersonii1
Bulbophyllum levinei2
Bulbophyllum levinei1
Bulbophyllum laxiflorum2
Bulbophyllum laxiflorum1
Bulbophyllum kwangtungense2
Bulbophyllum kwangtungense1
Bulbophyllum leopardinum1
Bulbophyllum pectinatum1
Bulbophyllum leopardinum2
Bulbophyllum leopardinum4
Bulbophyllum leopardinum5
Bulbophyllum leopardinum3
Bulbophyllum griffithii1
Bulbophyllum leopardinum6
Bulbophyllum nasutum3
Bulbophyllum nasutum1
Bulbophyllum nasutum2
Bulbophyllum disciflorum2
Bulbophyllum disciflorum1
Bulbophyllum disciflorum3
Bulbophyllum disciflorum5
Bulbophyllum disciflorum4
Bulbophyllum nasutum4
Bulbophyllum disciflorum6

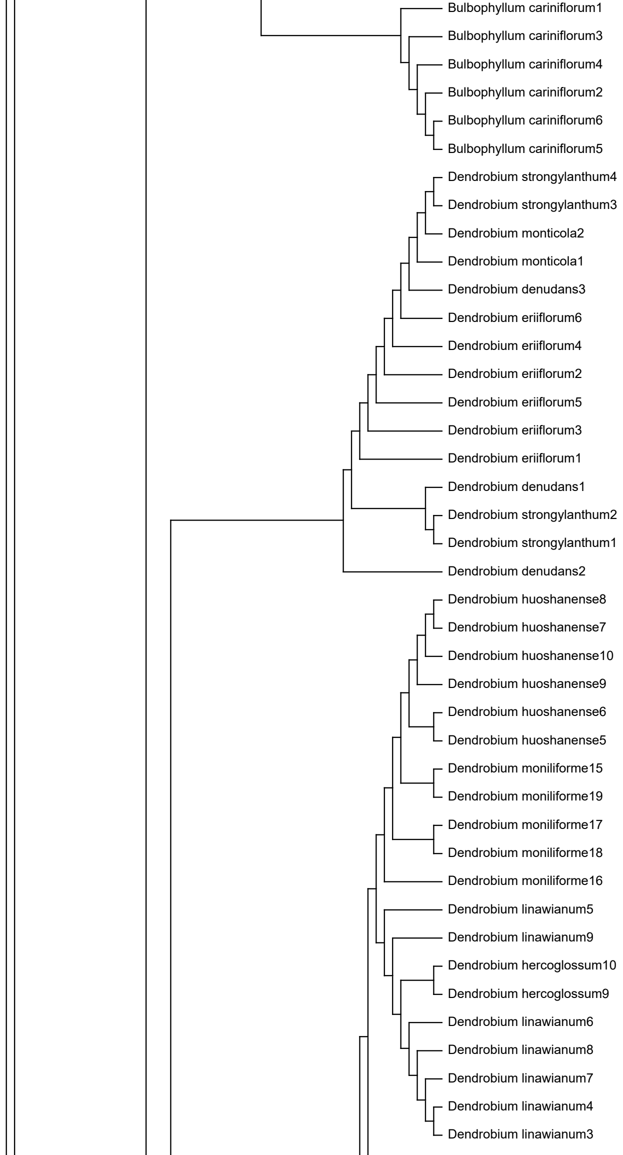
Bulbophyllum cariniflorum1
Bulbophyllum cariniflorum3
Bulbophyllum cariniflorum4
Bulbophyllum cariniflorum2
Bulbophyllum cariniflorum6
Bulbophyllum cariniflorum5
Dendrobium strongylanthum4
Dendrobium strongylanthum3
Dendrobium monticola2
Dendrobium monticola1
Dendrobium denudans3
Dendrobium eriiflorum6
Dendrobium eriiflorum4
Dendrobium eriiflorum2
Dendrobium eriiflorum5
Dendrobium eriiflorum3
Dendrobium eriiflorum1
Dendrobium denudans1
Dendrobium strongylanthum2
Dendrobium strongylanthum1
Dendrobium denudans2
Dendrobium huoshanense8
Dendrobium huoshanense7
Dendrobium huoshanense10
Dendrobium huoshanense9
Dendrobium huoshanense6
Dendrobium huoshanense5
Dendrobium moniliforme15
Dendrobium moniliforme19
Dendrobium moniliforme17
Dendrobium moniliforme18
Dendrobium moniliforme16
Dendrobium linawianum5
Dendrobium linawianum9
Dendrobium hercoglossum10
Dendrobium hercoglossum9
Dendrobium linawianum6
Dendrobium linawianum8
Dendrobium linawianum7
Dendrobium linawianum4
Dendrobium linawianum3

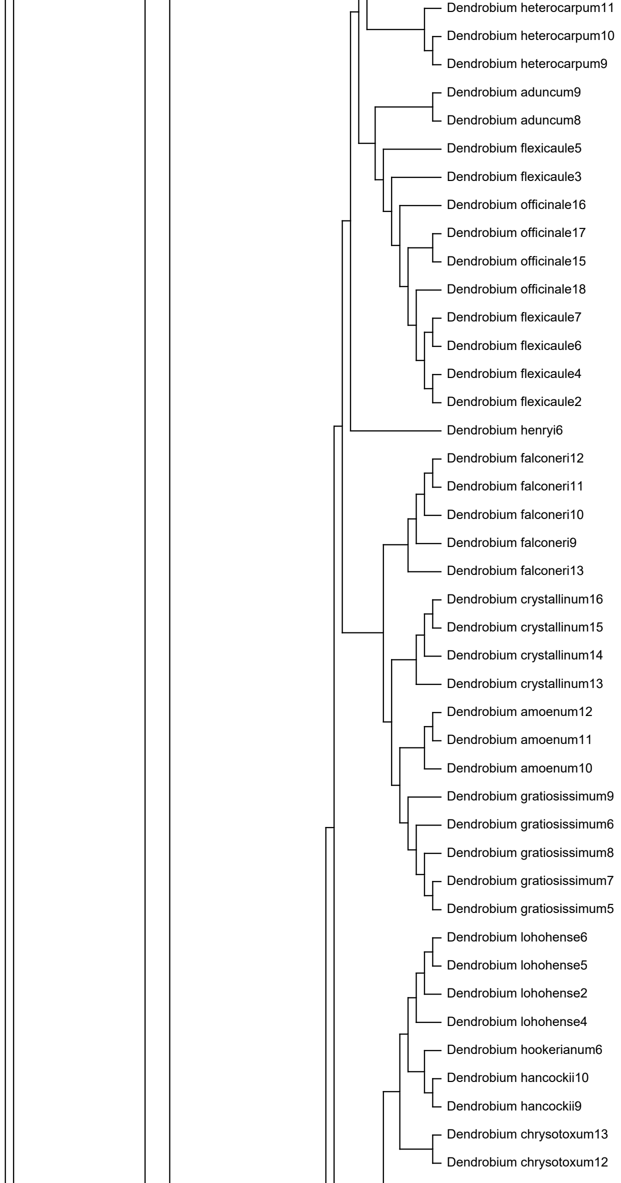
Dendrobium heterocarpum11
Dendrobium heterocarpum10
Dendrobium heterocarpum9
Dendrobium aduncum9
Dendrobium aduncum8
Dendrobium flexicaule5
Dendrobium flexicaule3
Dendrobium officinale16
Dendrobium officinale17
Dendrobium officinale15
Dendrobium officinale18
Dendrobium flexicaule7
Dendrobium flexicaule6
Dendrobium flexicaule4
Dendrobium flexicaule2
Dendrobium henryi6
Dendrobium falconeri12
Dendrobium falconeri11
Dendrobium falconeri10
Dendrobium falconeri9
Dendrobium falconeri13
Dendrobium crystallinum16
Dendrobium crystallinum15
Dendrobium crystallinum14
Dendrobium crystallinum13
Dendrobium amoenum12
Dendrobium amoenum11
Dendrobium amoenum10
Dendrobium gratiosissimum9
Dendrobium gratiosissimum6
Dendrobium gratiosissimum8
Dendrobium gratiosissimum7
Dendrobium gratiosissimum5
Dendrobium lohohense6
Dendrobium lohohense5
Dendrobium lohohense2
Dendrobium lohohense4
Dendrobium hookerianum6
Dendrobium hancockii10
Dendrobium hancockii9
Dendrobium chrysotoxum13
Dendrobium chrysotoxum12

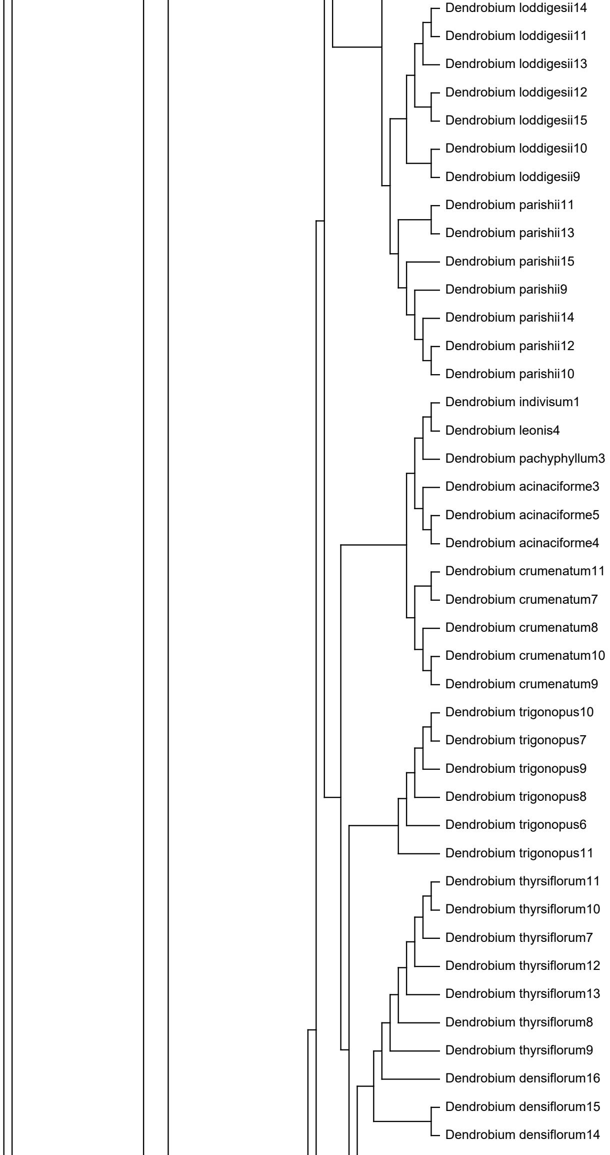

Dendrobium loddigesii14
Dendrobium loddigesii11
Dendrobium loddigesii13
Dendrobium loddigesii12
Dendrobium loddigesii15
Dendrobium loddigesii10
Dendrobium loddigesii9
Dendrobium parishii11
Dendrobium parishii13
Dendrobium parishii15
Dendrobium parishii9
Dendrobium parishii14
Dendrobium parishii12
Dendrobium parishii10
Dendrobium indivisum1
Dendrobium leonis4
Dendrobium pachyphyllum3
Dendrobium acinaciforme3
Dendrobium acinaciforme5
Dendrobium acinaciforme4
Dendrobium crumenatum11
Dendrobium crumenatum7
Dendrobium crumenatum8
Dendrobium crumenatum10
Dendrobium crumenatum9
Dendrobium trigonopus10
Dendrobium trigonopus7
Dendrobium trigonopus9
Dendrobium trigonopus8
Dendrobium trigonopus6
Dendrobium trigonopus11
Dendrobium thyrsiflorum11
Dendrobium thyrsiflorum10
Dendrobium thyrsiflorum7
Dendrobium thyrsiflorum12
Dendrobium thyrsiflorum13
Dendrobium thyrsiflorum8
Dendrobium thyrsiflorum9
Dendrobium densiflorum16
Dendrobium densiflorum15
Dendrobium densiflorum14

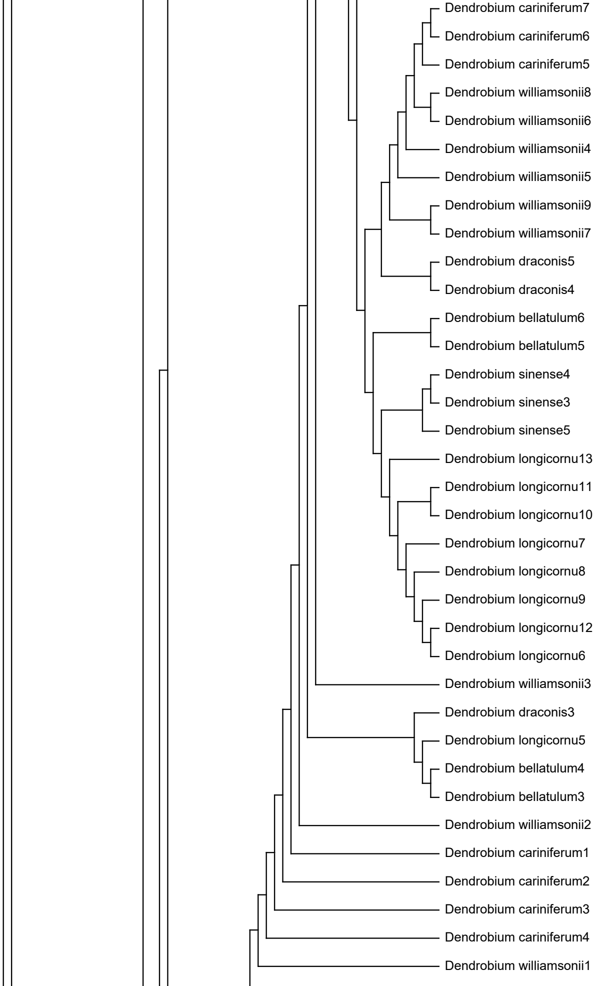
Dendrobium cariniferum7
Dendrobium cariniferum6
Dendrobium cariniferum5
Dendrobium williamsonii8
Dendrobium williamsonii6
Dendrobium williamsonii4
Dendrobium williamsonii5
Dendrobium williamsonii9
Dendrobium williamsonii7
Dendrobium draconis5
Dendrobium draconis4
Dendrobium bellatulum6
Dendrobium bellatulum5
Dendrobium sinense4
Dendrobium sinense3
Dendrobium sinense5
Dendrobium longicornu13
Dendrobium longicornu11
Dendrobium longicornu10
Dendrobium longicornu7
Dendrobium longicornu8
Dendrobium longicornu9
Dendrobium longicornu12
Dendrobium longicornu6
Dendrobium williamsonii3
Dendrobium draconis3
Dendrobium longicornu5
Dendrobium bellatulum4
Dendrobium bellatulum3
Dendrobium williamsonii2
Dendrobium cariniferum1
Dendrobium cariniferum2
Dendrobium cariniferum3
Dendrobium cariniferum4
Dendrobium williamsonii1

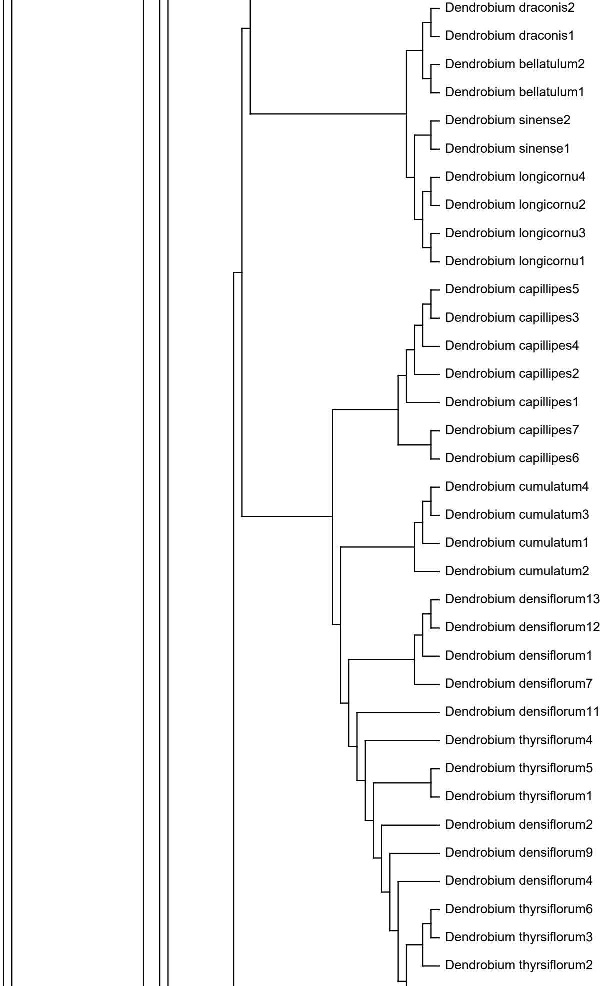
Dendrobium draconis2
Dendrobium draconis1
Dendrobium bellatulum2
Dendrobium bellatulum1
Dendrobium sinense2
Dendrobium sinense1
Dendrobium longicornu4
Dendrobium longicornu2
Dendrobium longicornu3
Dendrobium longicornu1
Dendrobium capillipes5
Dendrobium capillipes3
Dendrobium capillipes4
Dendrobium capillipes2
Dendrobium capillipes1
Dendrobium capillipes7
Dendrobium capillipes6
Dendrobium cumulatum4
Dendrobium cumulatum3
Dendrobium cumulatum1
Dendrobium cumulatum2
Dendrobium densiflorum13
Dendrobium densiflorum12
Dendrobium densiflorum1
Dendrobium densiflorum7
Dendrobium densiflorum11
Dendrobium thyrsiflorum4
Dendrobium thyrsiflorum5
Dendrobium thyrsiflorum1
Dendrobium densiflorum2
Dendrobium densiflorum9
Dendrobium densiflorum4
Dendrobium thyrsiflorum6
Dendrobium thyrsiflorum3
Dendrobium thyrsiflorum2

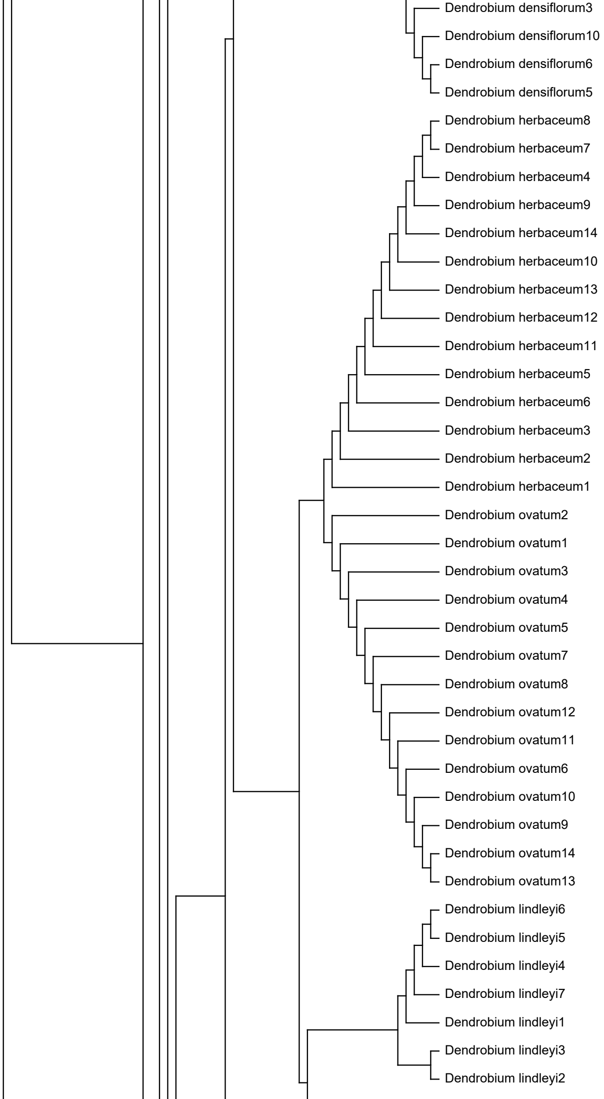
Dendrobium densiflorum3
Dendrobium densiflorum10
Dendrobium densiflorum6
Dendrobium densiflorum5
Dendrobium herbaceum8
Dendrobium herbaceum7
Dendrobium herbaceum4
Dendrobium herbaceum9
Dendrobium herbaceum14
Dendrobium herbaceum10
Dendrobium herbaceum13
Dendrobium herbaceum12
Dendrobium herbaceum11
Dendrobium herbaceum5
Dendrobium herbaceum6
Dendrobium herbaceum3
Dendrobium herbaceum2
Dendrobium herbaceum1
Dendrobium ovatum2
Dendrobium ovatum1
Dendrobium ovatum3
Dendrobium ovatum4
Dendrobium ovatum5
Dendrobium ovatum7
Dendrobium ovatum8
Dendrobium ovatum12
Dendrobium ovatum11
Dendrobium ovatum6
Dendrobium ovatum10
Dendrobium ovatum9
Dendrobium ovatum14
Dendrobium ovatum13
Dendrobium lindleyi6
Dendrobium lindleyi5
Dendrobium lindleyi4
Dendrobium lindleyi7
Dendrobium lindleyi1
Dendrobium lindleyi3
Dendrobium lindleyi2

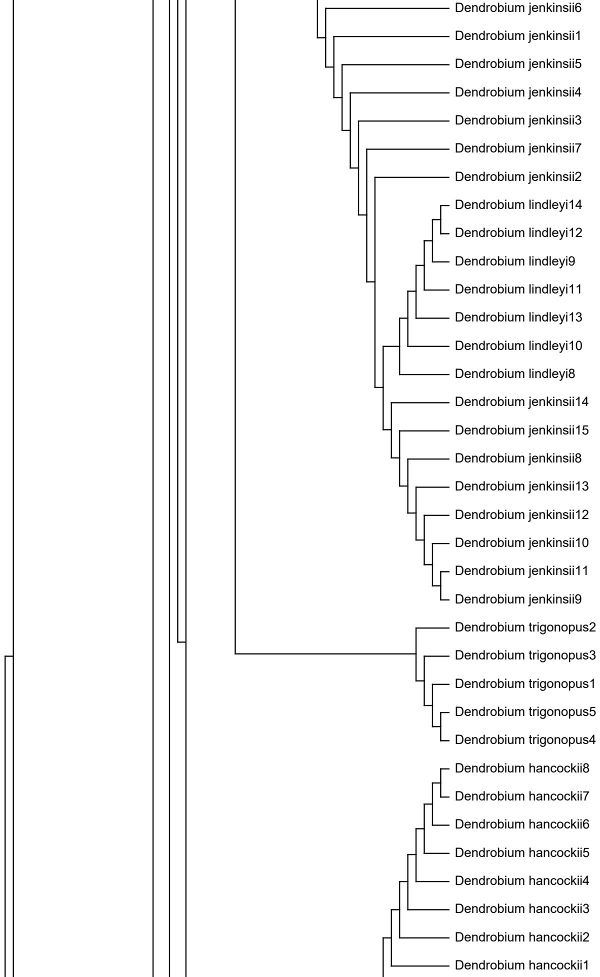
Dendrobium jenkinsii6
Dendrobium jenkinsii1
Dendrobium jenkinsii5
Dendrobium jenkinsii4
Dendrobium jenkinsii3
Dendrobium jenkinsii7
Dendrobium jenkinsii2
Dendrobium lindleyi14
Dendrobium lindleyi12
Dendrobium lindleyi9
Dendrobium lindleyi11
Dendrobium lindleyi13
Dendrobium lindleyi10
Dendrobium lindleyi8
Dendrobium jenkinsii14
Dendrobium jenkinsii15
Dendrobium jenkinsii8
Dendrobium jenkinsii13
Dendrobium jenkinsii12
Dendrobium jenkinsii10
Dendrobium jenkinsii11
Dendrobium jenkinsii9
Dendrobium trigonopus2
Dendrobium trigonopus3
Dendrobium trigonopus1
Dendrobium trigonopus5
Dendrobium trigonopus4
Dendrobium hancockii8
Dendrobium hancockii7
Dendrobium hancockii6
Dendrobium hancockii5
Dendrobium hancockii4
Dendrobium hancockii3
Dendrobium hancockii2
Dendrobium hancockii1

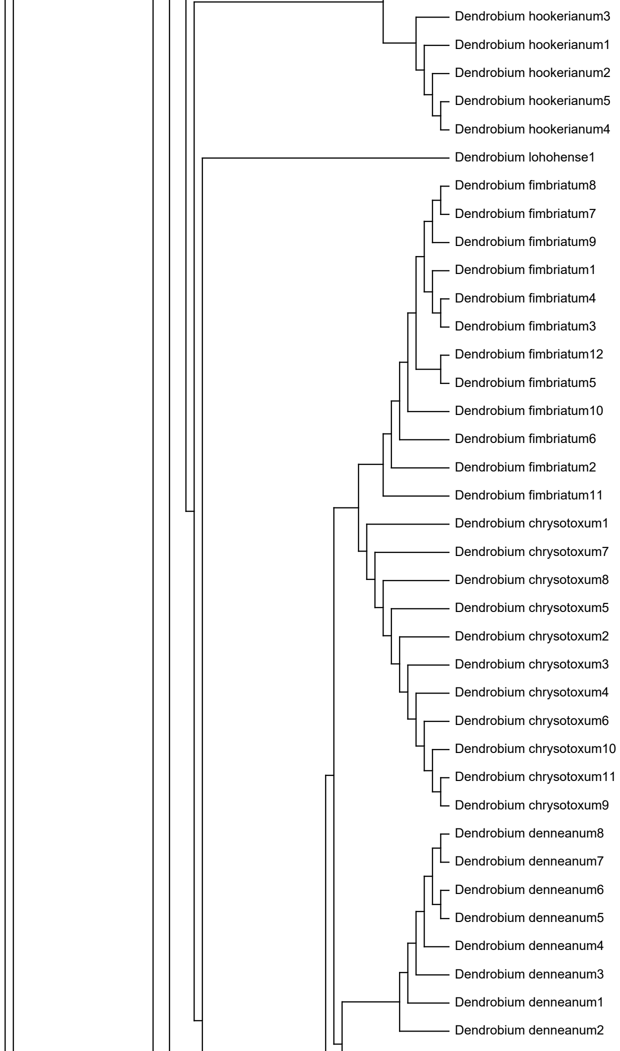
Dendrobium hookerianum3
Dendrobium hookerianum1
Dendrobium hookerianum2
Dendrobium hookerianum5
Dendrobium hookerianum4
Dendrobium lohohense1
Dendrobium fimbriatum8
Dendrobium fimbriatum7
Dendrobium fimbriatum9
Dendrobium fimbriatum1
Dendrobium fimbriatum4
Dendrobium fimbriatum3
Dendrobium fimbriatum12
Dendrobium fimbriatum5
Dendrobium fimbriatum10
Dendrobium fimbriatum6
Dendrobium fimbriatum2
Dendrobium fimbriatum11
Dendrobium chrysotoxum1
Dendrobium chrysotoxum7
Dendrobium chrysotoxum8
Dendrobium chrysotoxum5
Dendrobium chrysotoxum2
Dendrobium chrysotoxum3
Dendrobium chrysotoxum4
Dendrobium chrysotoxum6
Dendrobium chrysotoxum10
Dendrobium chrysotoxum11
Dendrobium chrysotoxum9
Dendrobium denneanum8
Dendrobium denneanum7
Dendrobium denneanum6
Dendrobium denneanum5
Dendrobium denneanum4
Dendrobium denneanum3
Dendrobium denneanum1
Dendrobium denneanum2

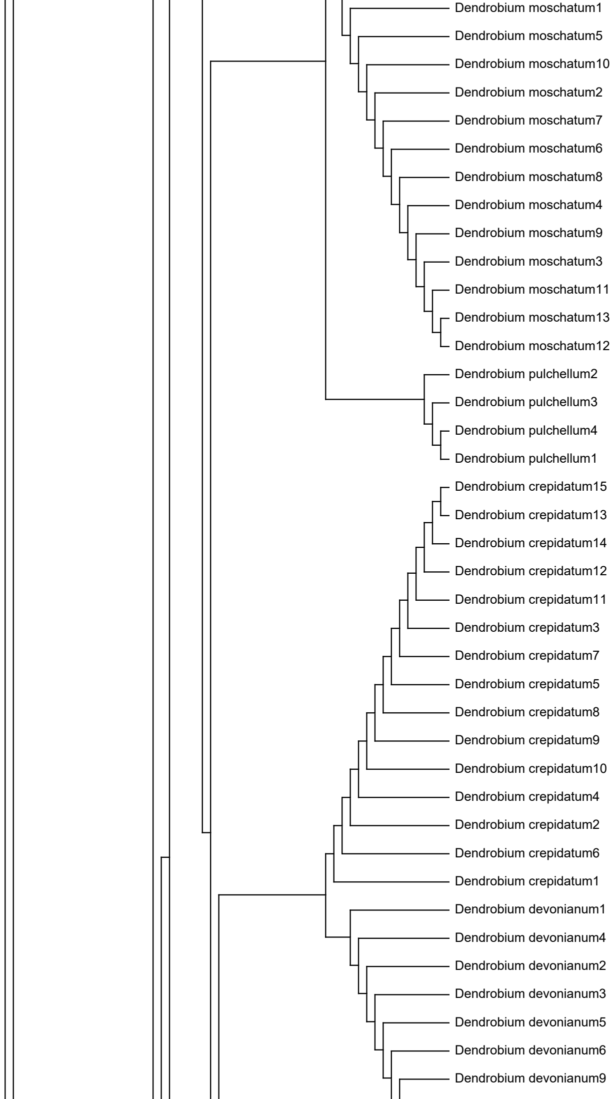
Dendrobium moschatum1
Dendrobium moschatum5
Dendrobium moschatum10
Dendrobium moschatum2
Dendrobium moschatum7
Dendrobium moschatum6
Dendrobium moschatum8
Dendrobium moschatum4
Dendrobium moschatum9
Dendrobium moschatum3
Dendrobium moschatum11
Dendrobium moschatum13
Dendrobium moschatum12
Dendrobium pulchellum2
Dendrobium pulchellum3
Dendrobium pulchellum4
Dendrobium pulchellum1
Dendrobium crepidatum15
Dendrobium crepidatum13
Dendrobium crepidatum14
Dendrobium crepidatum12
Dendrobium crepidatum11
Dendrobium crepidatum3
Dendrobium crepidatum7
Dendrobium crepidatum5
Dendrobium crepidatum8
Dendrobium crepidatum9
Dendrobium crepidatum10
Dendrobium crepidatum4
Dendrobium crepidatum2
Dendrobium crepidatum6
Dendrobium crepidatum1
Dendrobium devonianum1
Dendrobium devonianum4
Dendrobium devonianum2
Dendrobium devonianum3
Dendrobium devonianum5
Dendrobium devonianum6
Dendrobium devonianum9

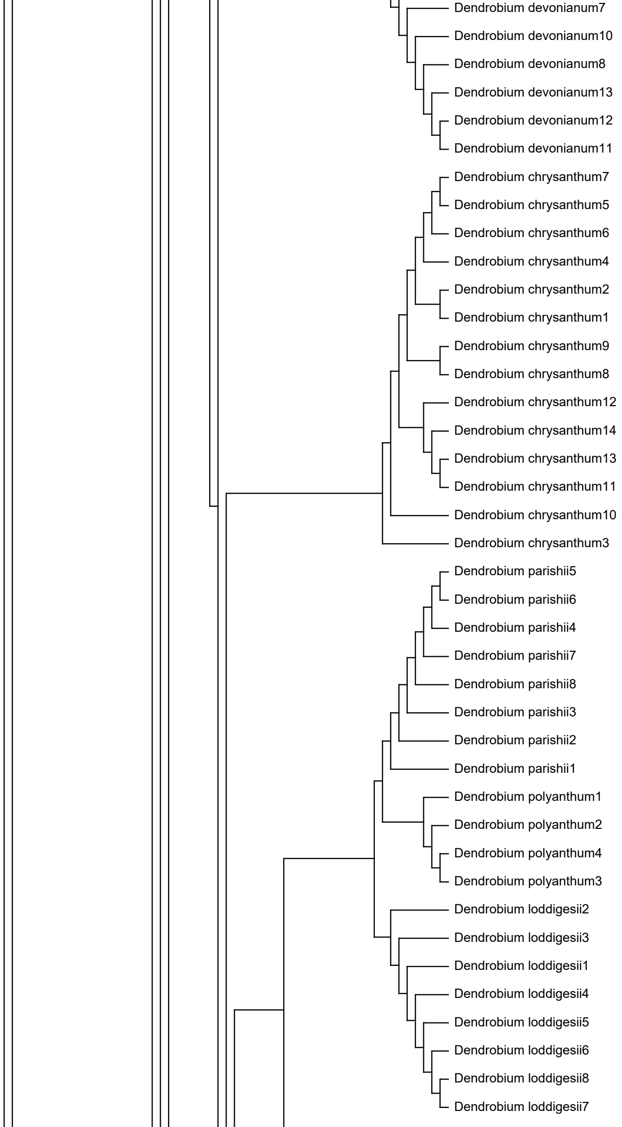

Dendrobium devonianum7
Dendrobium devonianum10
Dendrobium devonianum8
Dendrobium devonianum13
Dendrobium devonianum12
Dendrobium devonianum11
Dendrobium chrysanthum7
Dendrobium chrysanthum5
Dendrobium chrysanthum6
Dendrobium chrysanthum4
Dendrobium chrysanthum2
Dendrobium chrysanthum1
Dendrobium chrysanthum9
Dendrobium chrysanthum8
Dendrobium chrysanthum12
Dendrobium chrysanthum14
Dendrobium chrysanthum13
Dendrobium chrysanthum11
Dendrobium chrysanthum10
Dendrobium chrysanthum3
Dendrobium parishii5
Dendrobium parishii6
Dendrobium parishii4
Dendrobium parishii7
Dendrobium parishii8
Dendrobium parishii3
Dendrobium parishii2
Dendrobium parishii1
Dendrobium polyanthum1
Dendrobium polyanthum2
Dendrobium polyanthum4
Dendrobium polyanthum3
Dendrobium loddigesii2
Dendrobium loddigesii3
Dendrobium loddigesii1
Dendrobium loddigesii4
Dendrobium loddigesii5
Dendrobium loddigesii6
Dendrobium loddigesii8
Dendrobium loddigesii7

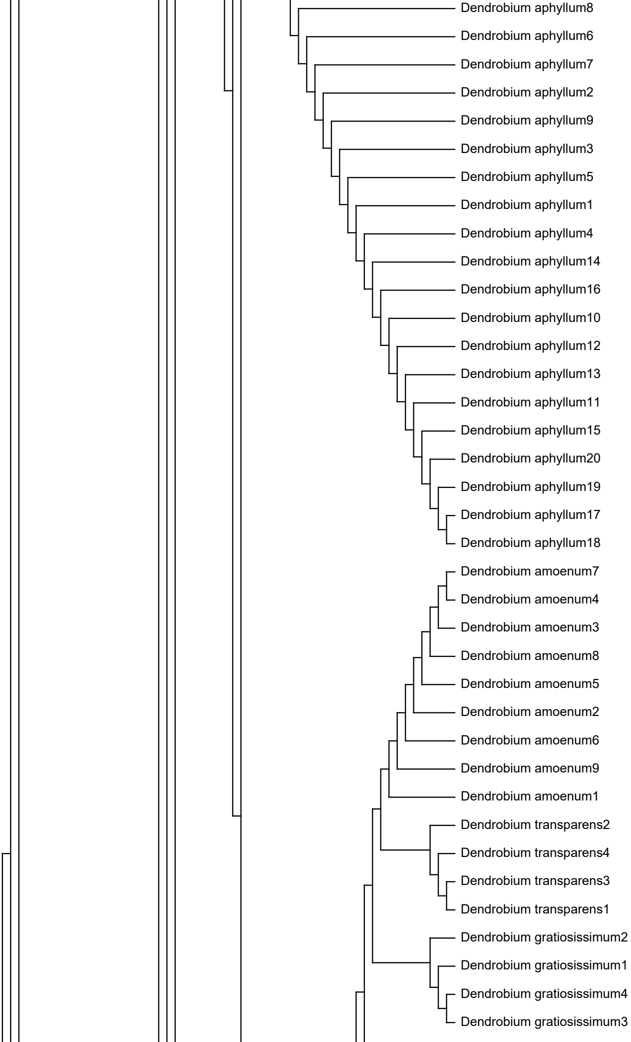
Dendrobium aphyllum8
Dendrobium aphyllum6
Dendrobium aphyllum7
Dendrobium aphyllum2
Dendrobium aphyllum9
Dendrobium aphyllum3
Dendrobium aphyllum5
Dendrobium aphyllum1
Dendrobium aphyllum4
Dendrobium aphyllum14
Dendrobium aphyllum16
Dendrobium aphyllum10
Dendrobium aphyllum12
Dendrobium aphyllum13
Dendrobium aphyllum11
Dendrobium aphyllum15
Dendrobium aphyllum20
Dendrobium aphyllum19
Dendrobium aphyllum17
Dendrobium aphyllum18
Dendrobium amoenum7
Dendrobium amoenum4
Dendrobium amoenum3
Dendrobium amoenum8
Dendrobium amoenum5
Dendrobium amoenum2
Dendrobium amoenum6
Dendrobium amoenum9
Dendrobium amoenum1
Dendrobium transparens2
Dendrobium transparens4
Dendrobium transparens3
Dendrobium transparens1
Dendrobium gratiosissimum2
Dendrobium gratiosissimum1
Dendrobium gratiosissimum4
Dendrobium gratiosissimum3

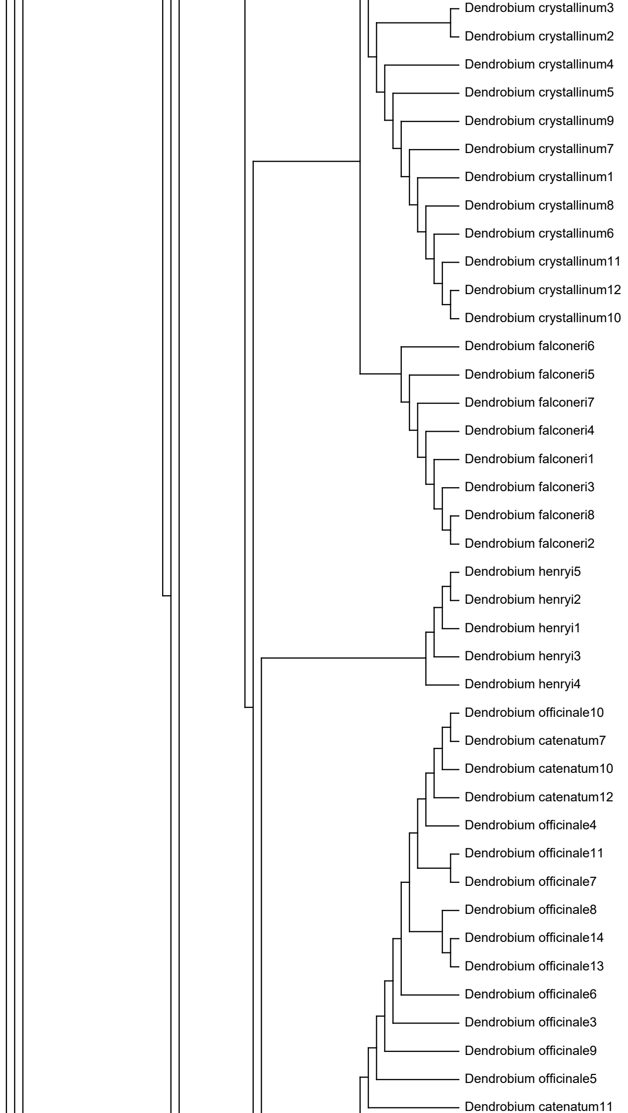
Dendrobium crystallinum3
Dendrobium crystallinum2
Dendrobium crystallinum4
Dendrobium crystallinum5
Dendrobium crystallinum9
Dendrobium crystallinum7
Dendrobium crystallinum1
Dendrobium crystallinum8
Dendrobium crystallinum6
Dendrobium crystallinum11
Dendrobium crystallinum12
Dendrobium crystallinum10
Dendrobium falconeri6
Dendrobium falconeri5
Dendrobium falconeri7
Dendrobium falconeri4
Dendrobium falconeri1
Dendrobium falconeri3
Dendrobium falconeri8
Dendrobium falconeri2
Dendrobium henryi5
Dendrobium henryi2
Dendrobium henryi1
Dendrobium henryi3
Dendrobium henryi4
Dendrobium officinale10
Dendrobium catenatum7
Dendrobium catenatum10
Dendrobium catenatum12
Dendrobium officinale4
Dendrobium officinale11
Dendrobium officinale7
Dendrobium officinale8
Dendrobium officinale14
Dendrobium officinale13
Dendrobium officinale6
Dendrobium officinale3
Dendrobium officinale9
Dendrobium officinale5
Dendrobium catenatum11

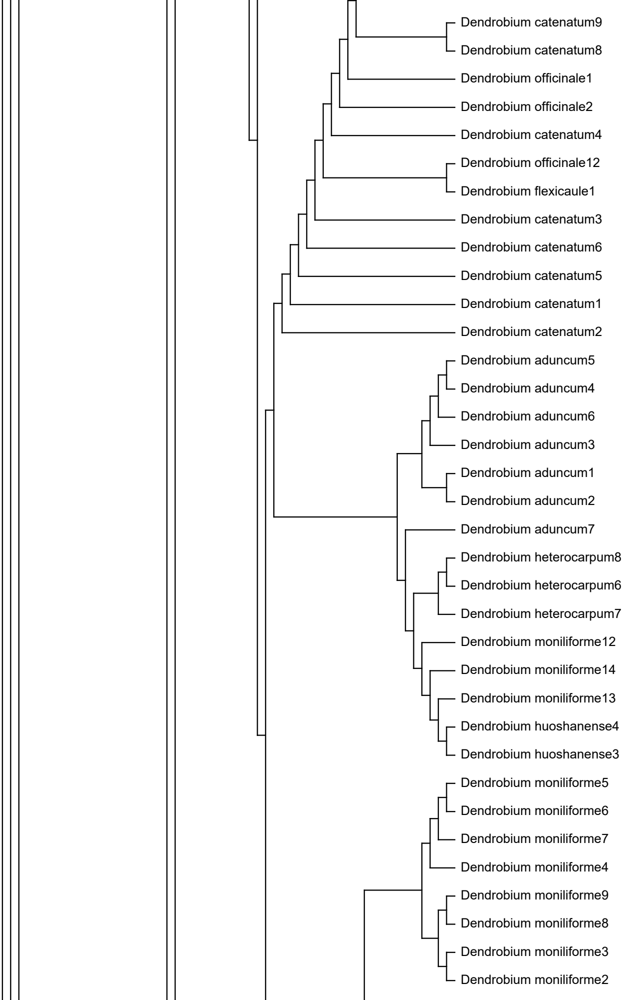
Dendrobium catenatum9
Dendrobium catenatum8
Dendrobium officinale1
Dendrobium officinale2
Dendrobium catenatum4
Dendrobium officinale12
Dendrobium flexicaule1
Dendrobium catenatum3
Dendrobium catenatum6
Dendrobium catenatum5
Dendrobium catenatum1
Dendrobium catenatum2
Dendrobium aduncum5
Dendrobium aduncum4
Dendrobium aduncum6
Dendrobium aduncum3
Dendrobium aduncum1
Dendrobium aduncum2
Dendrobium aduncum7
Dendrobium heterocarpum8
Dendrobium heterocarpum6
Dendrobium heterocarpum7
Dendrobium moniliforme12
Dendrobium moniliforme14
Dendrobium moniliforme13
Dendrobium huoshanense4
Dendrobium huoshanense3
Dendrobium moniliforme5
Dendrobium moniliforme6
Dendrobium moniliforme7
Dendrobium moniliforme4
Dendrobium moniliforme9
Dendrobium moniliforme8
Dendrobium moniliforme3
Dendrobium moniliforme2

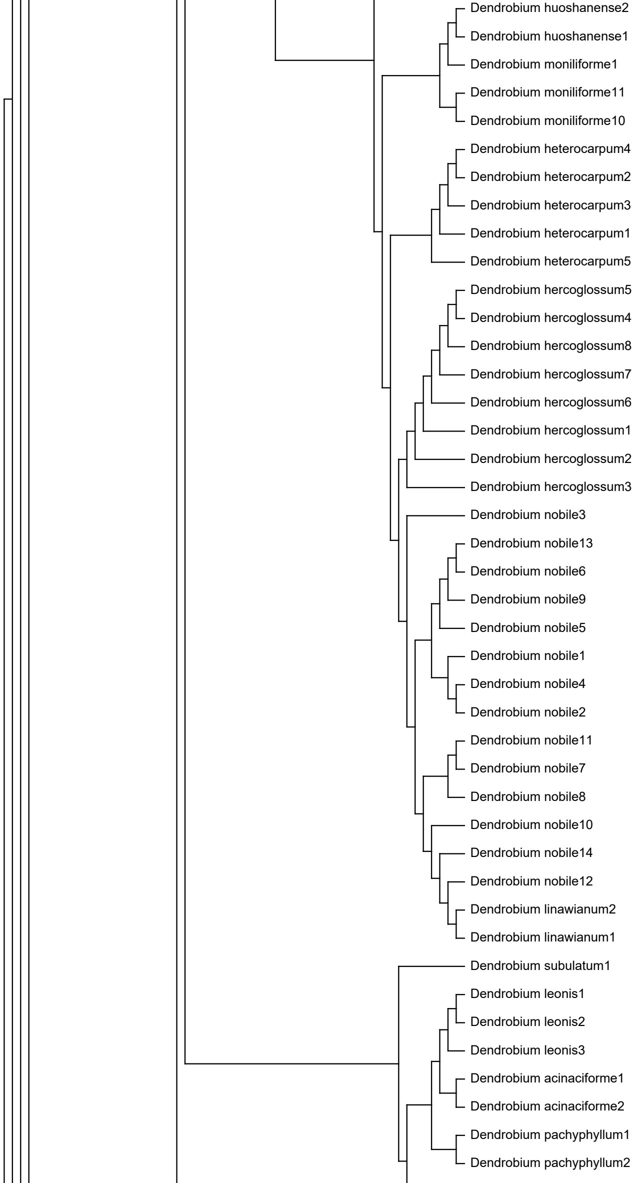
Dendrobium huoshanense2
Dendrobium huoshanense1
Dendrobium moniliforme1
Dendrobium moniliforme11
Dendrobium moniliforme10
Dendrobium heterocarpum4
Dendrobium heterocarpum2
Dendrobium heterocarpum3
Dendrobium heterocarpum1
Dendrobium heterocarpum5
Dendrobium hercoglossum5
Dendrobium hercoglossum4
Dendrobium hercoglossum8
Dendrobium hercoglossum7
Dendrobium hercoglossum6
Dendrobium hercoglossum1
Dendrobium hercoglossum2
Dendrobium hercoglossum3
Dendrobium nobile3
Dendrobium nobile13
Dendrobium nobile6
Dendrobium nobile9
Dendrobium nobile5
Dendrobium nobile1
Dendrobium nobile4
Dendrobium nobile2
Dendrobium nobile11
Dendrobium nobile7
Dendrobium nobile8
Dendrobium nobile10
Dendrobium nobile14
Dendrobium nobile12
Dendrobium linawianum2
Dendrobium linawianum1
Dendrobium subulatum1
Dendrobium leonis1
Dendrobium leonis2
Dendrobium leonis3
Dendrobium acinaciforme1
Dendrobium acinaciforme2
Dendrobium pachyphyllum1
Dendrobium pachyphyllum2

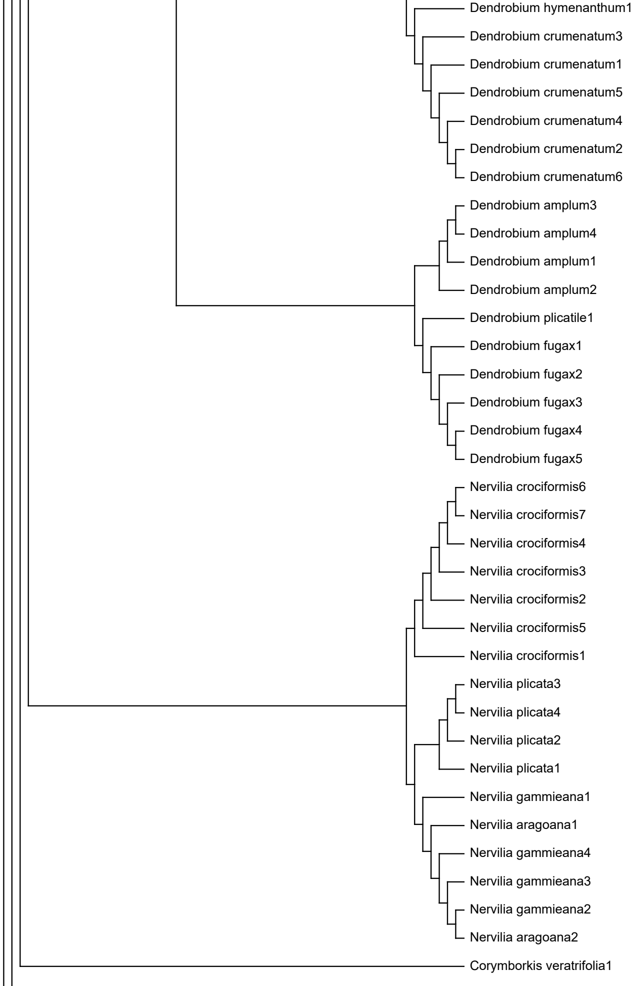
Dendrobium hymenanthum1
Dendrobium crumenatum3
Dendrobium crumenatum1
Dendrobium crumenatum5
Dendrobium crumenatum4
Dendrobium crumenatum2
Dendrobium crumenatum6
Dendrobium amplum3
Dendrobium amplum4
Dendrobium amplum1
Dendrobium amplum2
Dendrobium plicatile1
Dendrobium fugax1
Dendrobium fugax2
Dendrobium fugax3
Dendrobium fugax4
Dendrobium fugax5
Nervilia crociformis6
Nervilia crociformis7
Nervilia crociformis4
Nervilia crociformis3
Nervilia crociformis2
Nervilia crociformis5
Nervilia crociformis1
Nervilia plicata3
Nervilia plicata4
Nervilia plicata2
Nervilia plicata1
Nervilia gammieana1
Nervilia aragoana1
Nervilia gammieana4
Nervilia gammieana3
Nervilia gammieana2
Nervilia aragoana2
Corymborkis veratrifolia1

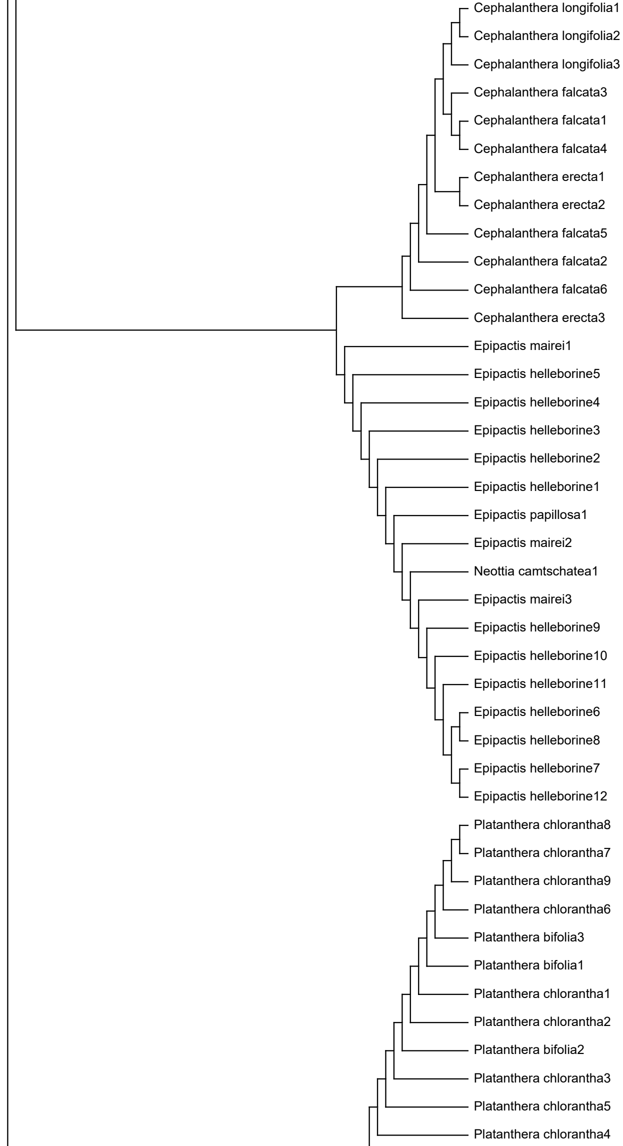
Cephalanthera longifolia1
Cephalanthera longifolia2
Cephalanthera longifolia3
Cephalanthera falcata3
Cephalanthera falcata1
Cephalanthera falcata4
Cephalanthera erecta1
Cephalanthera erecta2
Cephalanthera falcata5
Cephalanthera falcata2
Cephalanthera falcata6
Cephalanthera erecta3
Epipactis mairei1
Epipactis helleborine5
Epipactis helleborine4
Epipactis helleborine3
Epipactis helleborine2
Epipactis helleborine1
Epipactis papillosa1
Epipactis mairei2
Neottia camtschatea1
Epipactis mairei3
Epipactis helleborine9
Epipactis helleborine10
Epipactis helleborine11
Epipactis helleborine6
Epipactis helleborine8
Epipactis helleborine7
Epipactis helleborine12
Platanthera chlorantha8
Platanthera chlorantha7
Platanthera chlorantha9
Platanthera chlorantha6
Platanthera bifolia3
Platanthera bifolia1
Platanthera chlorantha1
Platanthera chlorantha2
Platanthera bifolia2
Platanthera chlorantha3
Platanthera chlorantha5
Platanthera chlorantha4

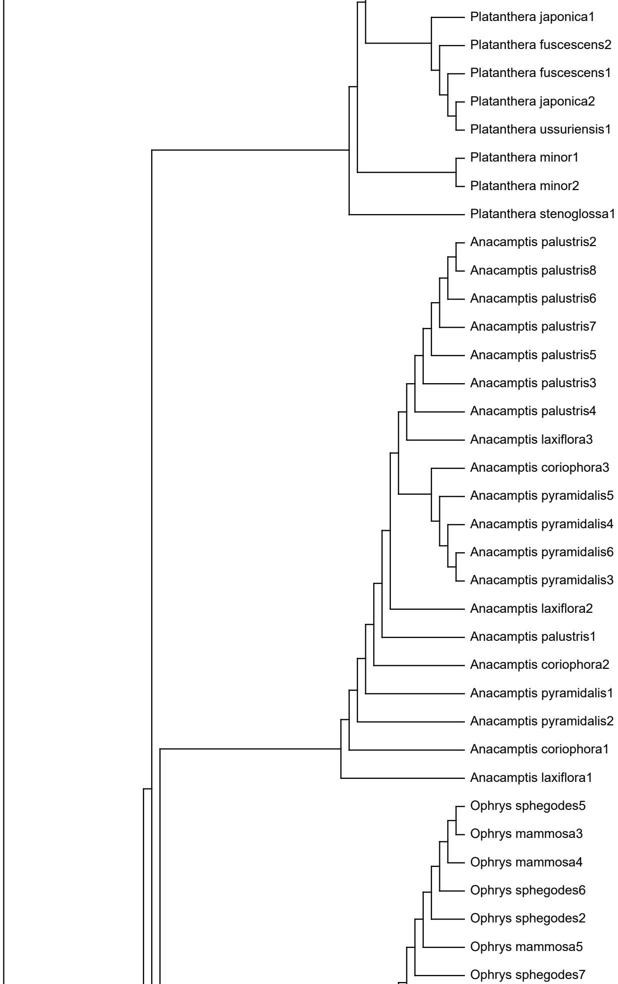
Platanthera japonica1
Platanthera fuscescens2
Platanthera fuscescens1
Platanthera japonica2
Platanthera ussuriensis1
Platanthera minor1
Platanthera minor2
Platanthera stenoglossa1
Anacamptis palustris2
Anacamptis palustris8
Anacamptis palustris6
Anacamptis palustris7
Anacamptis palustris5
Anacamptis palustris3
Anacamptis palustris4
Anacamptis laxiflora3
Anacamptis coriophora3
Anacamptis pyramidalis5
Anacamptis pyramidalis4
Anacamptis pyramidalis6
Anacamptis pyramidalis3
Anacamptis laxiflora2
Anacamptis palustris1
Anacamptis coriophora2
Anacamptis pyramidalis1
Anacamptis pyramidalis2
Anacamptis coriophora1
Anacamptis laxiflora1
Ophrys sphegodes5
Ophrys mammosa3
Ophrys mammosa4
Ophrys sphegodes6
Ophrys sphegodes2
Ophrys mammosa5
Ophrys sphegodes7

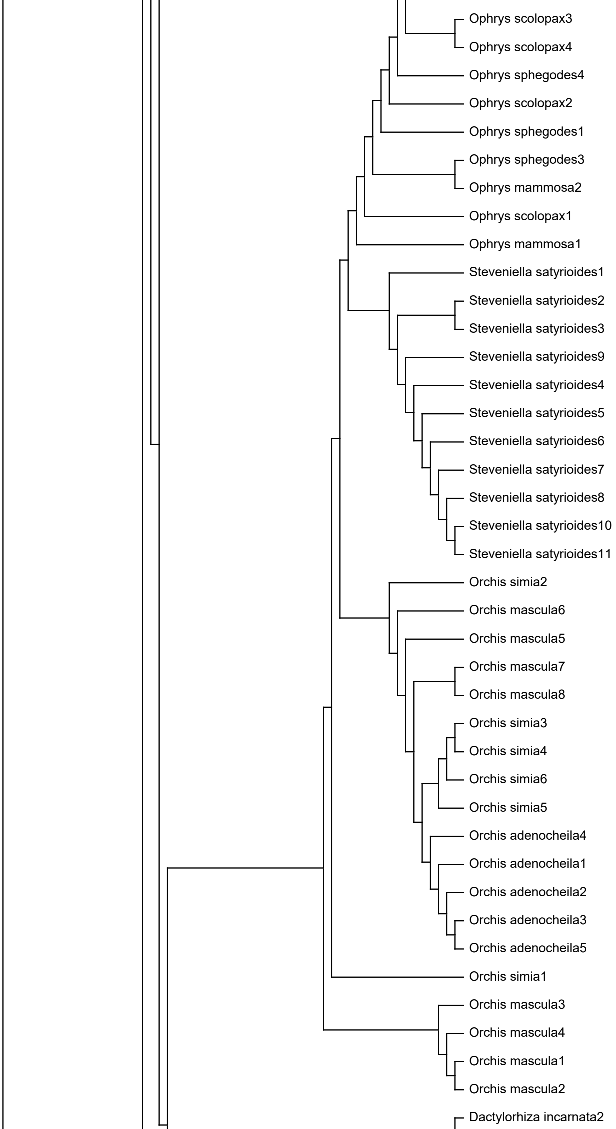
Ophrys scolopax3
Ophrys scolopax4
Ophrys sphegodes4
Ophrys scolopax2
Ophrys sphegodes1
Ophrys sphegodes3
Ophrys mammosa2
Ophrys scolopax1
Ophrys mammosa1
Steveniella satyrioides1
Steveniella satyrioides2
Steveniella satyrioides3
Steveniella satyrioides9
Steveniella satyrioides4
Steveniella satyrioides5
Steveniella satyrioides6
Steveniella satyrioides7
Steveniella satyrioides8
Steveniella satyrioides10
Steveniella satyrioides11
Orchis simia2
Orchis mascula6
Orchis mascula5
Orchis mascula7
Orchis mascula8
Orchis simia3
Orchis simia4
Orchis simia6
Orchis simia5
Orchis adenocheila4
Orchis adenocheila1
Orchis adenocheila2
Orchis adenocheila3
Orchis adenocheila5
Orchis simia1
Orchis mascula3
Orchis mascula4
Orchis mascula1
Orchis mascula2
Dactylorhiza incarnata2

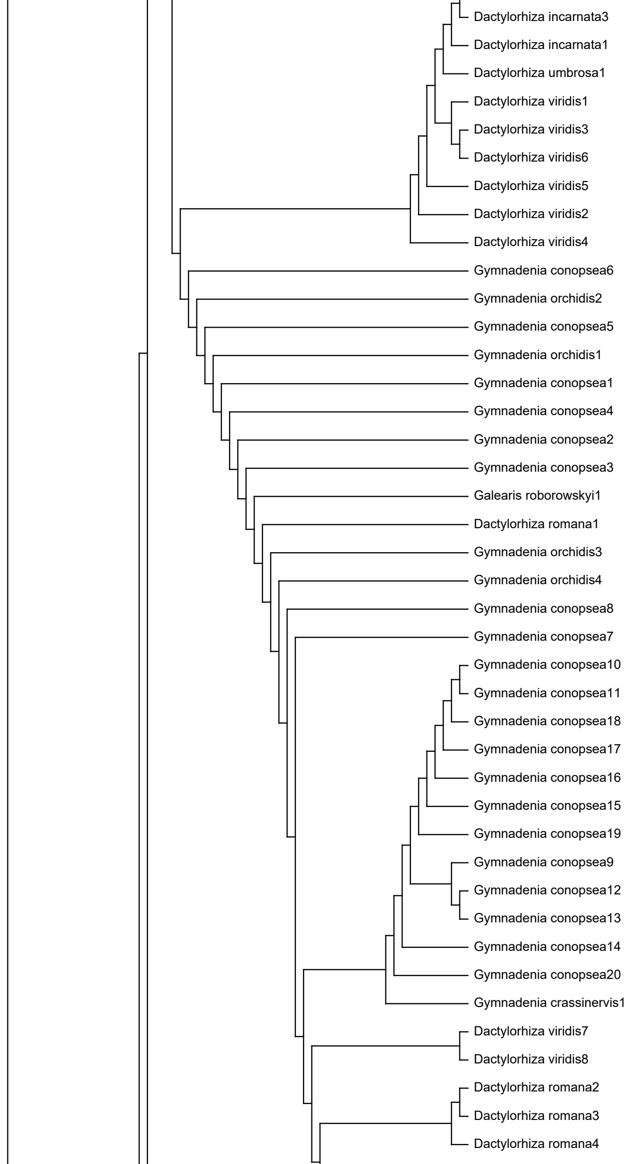
Dactylorhiza incarnata3
Dactylorhiza incarnata1
Dactylorhiza umbrosa1
Dactylorhiza viridis1
Dactylorhiza viridis3
Dactylorhiza viridis6
Dactylorhiza viridis5
Dactylorhiza viridis2
Dactylorhiza viridis4
Gymnadenia conopsea6
Gymnadenia orchidis2
Gymnadenia conopsea5
Gymnadenia orchidis1
Gymnadenia conopsea1
Gymnadenia conopsea4
Gymnadenia conopsea2
Gymnadenia conopsea3
Galearis roborowskyi1
Dactylorhiza romana1
Gymnadenia orchidis3
Gymnadenia orchidis4
Gymnadenia conopsea8
Gymnadenia conopsea7
Gymnadenia conopsea10
Gymnadenia conopsea11
Gymnadenia conopsea18
Gymnadenia conopsea17
Gymnadenia conopsea16
Gymnadenia conopsea15
Gymnadenia conopsea19
Gymnadenia conopsea9
Gymnadenia conopsea12
Gymnadenia conopsea13
Gymnadenia conopsea14
Gymnadenia conopsea20
Gymnadenia crassinervis1
Dactylorhiza viridis7
Dactylorhiza viridis8
Dactylorhiza romana2
Dactylorhiza romana3
Dactylorhiza romana4

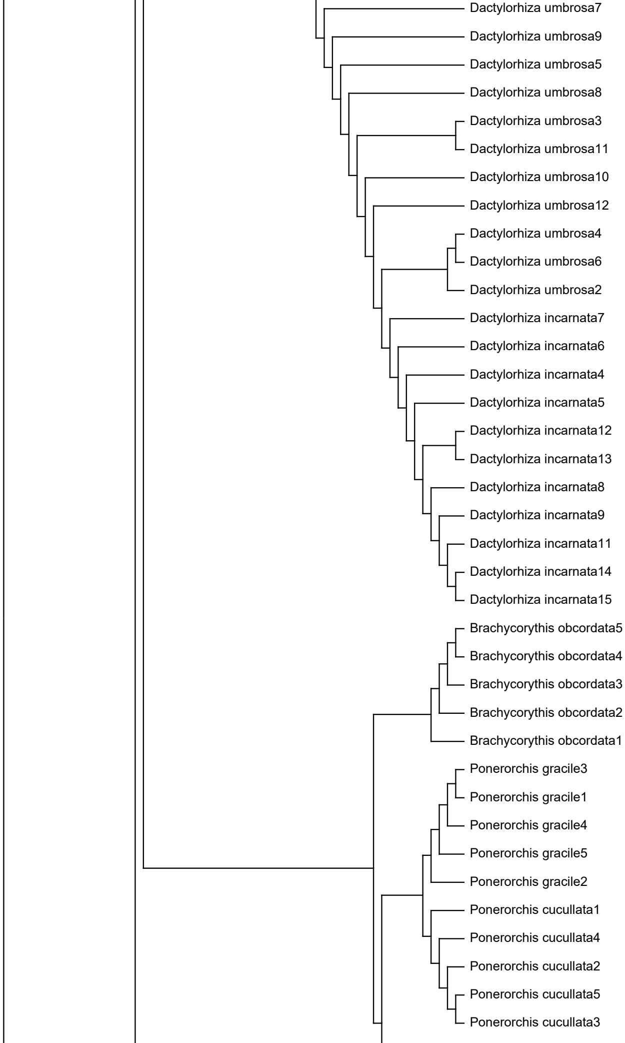
Dactylorhiza umbrosa7
Dactylorhiza umbrosa9
Dactylorhiza umbrosa5
Dactylorhiza umbrosa8
Dactylorhiza umbrosa3
Dactylorhiza umbrosa11
Dactylorhiza umbrosa10
Dactylorhiza umbrosa12
Dactylorhiza umbrosa4
Dactylorhiza umbrosa6
Dactylorhiza umbrosa2
Dactylorhiza incarnata7
Dactylorhiza incarnata6
Dactylorhiza incarnata4
Dactylorhiza incarnata5
Dactylorhiza incarnata12
Dactylorhiza incarnata13
Dactylorhiza incarnata8
Dactylorhiza incarnata9
Dactylorhiza incarnata11
Dactylorhiza incarnata14
Dactylorhiza incarnata15
Brachycorythis obcordata5
Brachycorythis obcordata4
Brachycorythis obcordata3
Brachycorythis obcordata2
Brachycorythis obcordata1
Ponerorchis gracile3
Ponerorchis gracile1
Ponerorchis gracile4
Ponerorchis gracile5
Ponerorchis gracile2
Ponerorchis cucullata1
Ponerorchis cucullata4
Ponerorchis cucullata2
Ponerorchis cucullata5
Ponerorchis cucullata3

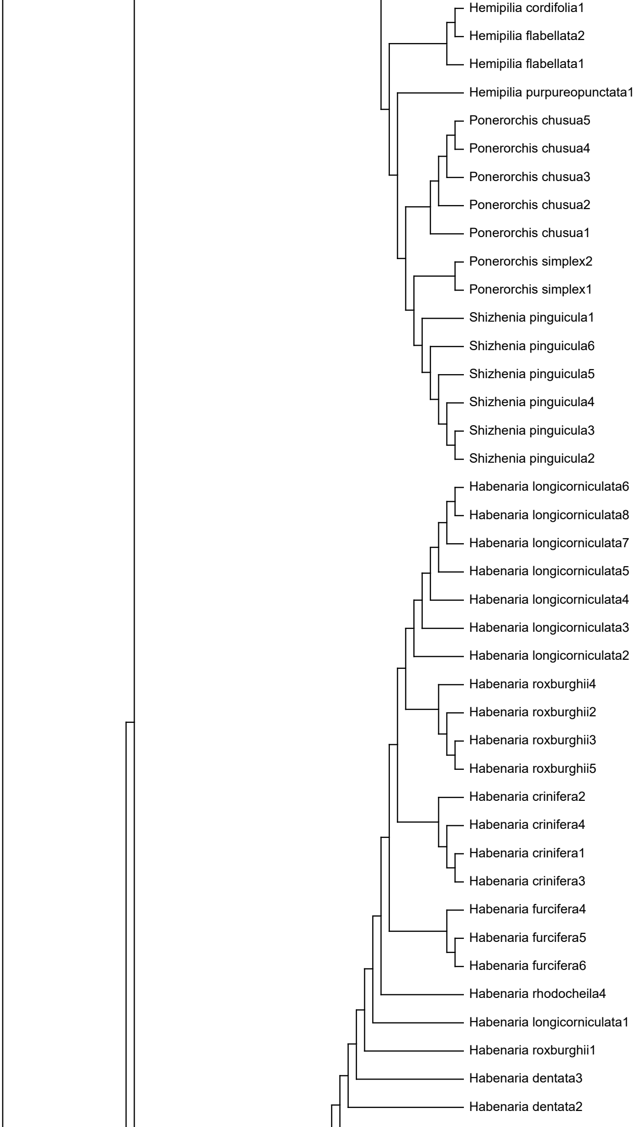

Hemipilia cordifolia1
Hemipilia flabellata2
Hemipilia flabellata1
Hemipilia purpureopunctata1
Ponerorchis chusua5
Ponerorchis chusua4
Ponerorchis chusua3
Ponerorchis chusua2
Ponerorchis chusua1
Ponerorchis simplex2
Ponerorchis simplex1
Shizhenia pinguicula1
Shizhenia pinguicula6
Shizhenia pinguicula5
Shizhenia pinguicula4
Shizhenia pinguicula3
Shizhenia pinguicula2
Habenaria longicorniculata6
Habenaria longicorniculata8
Habenaria longicorniculata7
Habenaria longicorniculata5
Habenaria longicorniculata4
Habenaria longicorniculata3
Habenaria longicorniculata2
Habenaria roxburghii4
Habenaria roxburghii2
Habenaria roxburghii3
Habenaria roxburghii5
Habenaria crinifera2
Habenaria crinifera4
Habenaria crinifera1
Habenaria crinifera3
Habenaria furcifera4
Habenaria furcifera5
Habenaria furcifera6
Habenaria rhodocheila4
Habenaria longicorniculata1
Habenaria roxburghii1
Habenaria dentata3
Habenaria dentata2

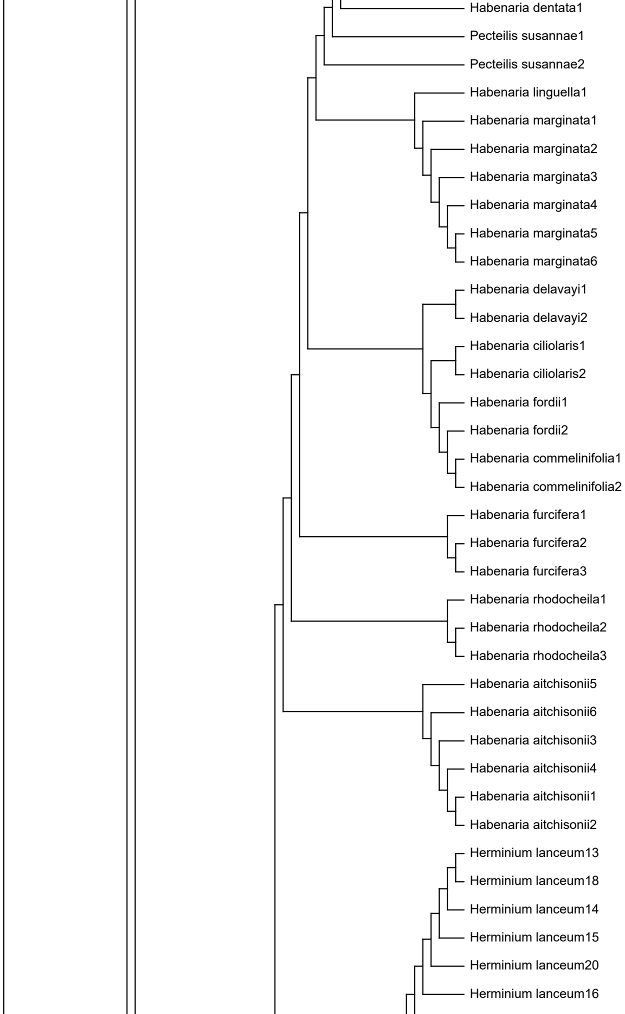

Habenaria dentata1
Pecteilis susannae1
Pecteilis susannae2
Habenaria linguella1
Habenaria marginata1
Habenaria marginata2
Habenaria marginata3
Habenaria marginata4
Habenaria marginata5
Habenaria marginata6
Habenaria delavayi1
Habenaria delavayi2
Habenaria ciliolaris1
Habenaria ciliolaris2
Habenaria fordii1
Habenaria fordii2
Habenaria commelinifolia1
Habenaria commelinifolia2
Habenaria furcifera1
Habenaria furcifera2
Habenaria furcifera3
Habenaria rhodocheila1
Habenaria rhodocheila2
Habenaria rhodocheila3
Habenaria aitchisonii5
Habenaria aitchisonii6
Habenaria aitchisonii3
Habenaria aitchisonii4
Habenaria aitchisonii1
Habenaria aitchisonii2
Herminium lanceum13
Herminium lanceum18
Herminium lanceum14
Herminium lanceum15
Herminium lanceum20
Herminium lanceum16

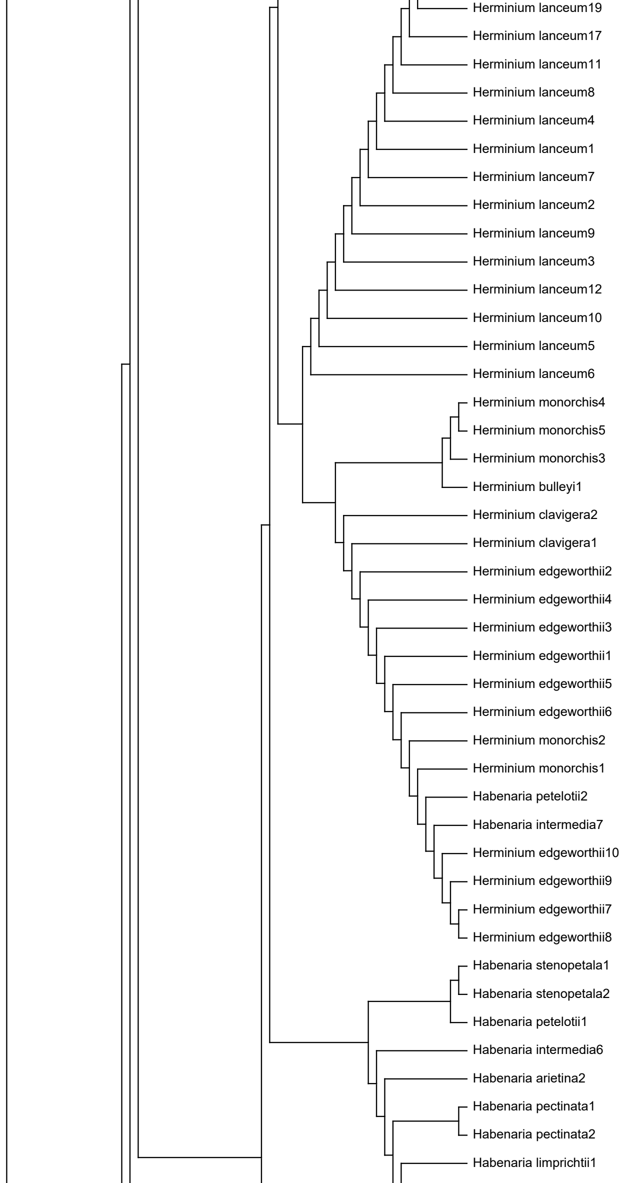
Herminium lanceum19
Herminium lanceum17
Herminium lanceum11
Herminium lanceum8
Herminium lanceum4
Herminium lanceum1
Herminium lanceum7
Herminium lanceum2
Herminium lanceum9
Herminium lanceum3
Herminium lanceum12
Herminium lanceum10
Herminium lanceum5
Herminium lanceum6
Herminium monorchis4
Herminium monorchis5
Herminium monorchis3
Herminium bulleyi1
Herminium clavigera2
Herminium clavigera1
Herminium edgeworthii2
Herminium edgeworthii4
Herminium edgeworthii3
Herminium edgeworthii1
Herminium edgeworthii5
Herminium edgeworthii6
Herminium monorchis2
Herminium monorchis1
Habenaria petelotii2
Habenaria intermedia7
Herminium edgeworthii10
Herminium edgeworthii9
Herminium edgeworthii7
Herminium edgeworthii8
Habenaria stenopetala1
Habenaria stenopetala2
Habenaria petelotii1
Habenaria intermedia6
Habenaria arietina2
Habenaria pectinata1
Habenaria pectinata2
Habenaria limprichtii1

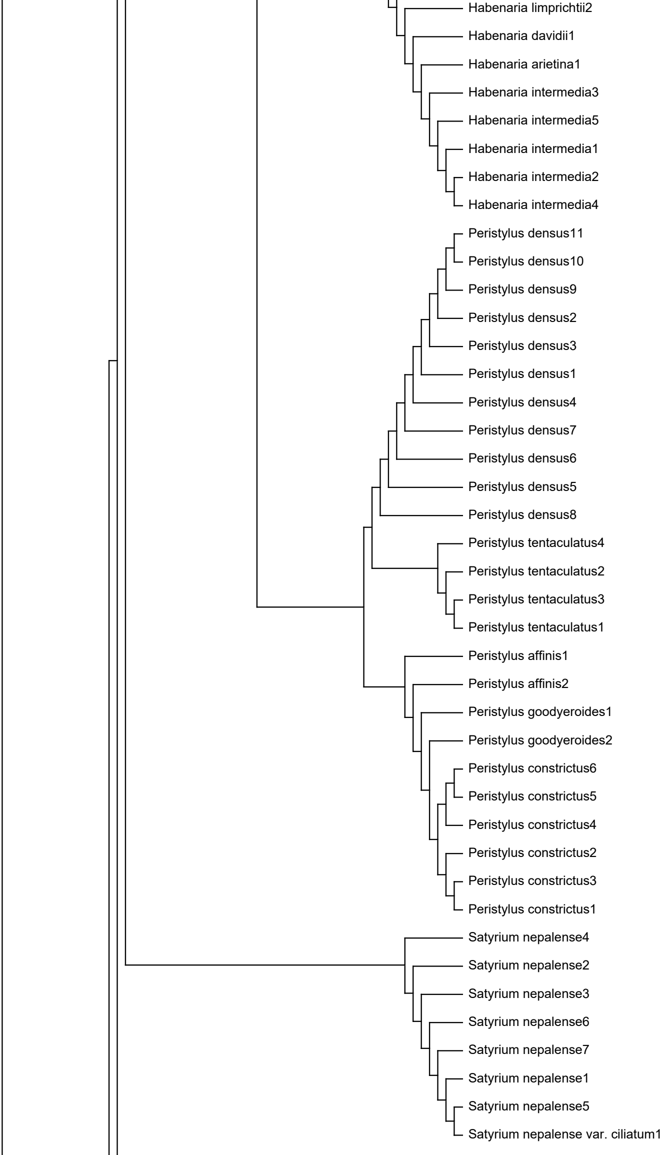
Habenaria limprichtii2
Habenaria davidii1
Habenaria arietina1
Habenaria intermedia3
Habenaria intermedia5
Habenaria intermedia1
Habenaria intermedia2
Habenaria intermedia4
Peristylus densus11
Peristylus densus10
Peristylus densus9
Peristylus densus2
Peristylus densus3
Peristylus densus1
Peristylus densus4
Peristylus densus7
Peristylus densus6
Peristylus densus5
Peristylus densus8
Peristylus tentaculatus4
Peristylus tentaculatus2
Peristylus tentaculatus3
Peristylus tentaculatus1
Peristylus affinis1
Peristylus affinis2
Peristylus goodyeroides1
Peristylus goodyeroides2
Peristylus constrictus6
Peristylus constrictus5
Peristylus constrictus4
Peristylus constrictus2
Peristylus constrictus3
Peristylus constrictus1
Satyrium nepalense4
Satyrium nepalense2
Satyrium nepalense3
Satyrium nepalense6
Satyrium nepalense7
Satyrium nepalense1
Satyrium nepalense5
Satyrium nepalense var. ciliatum1

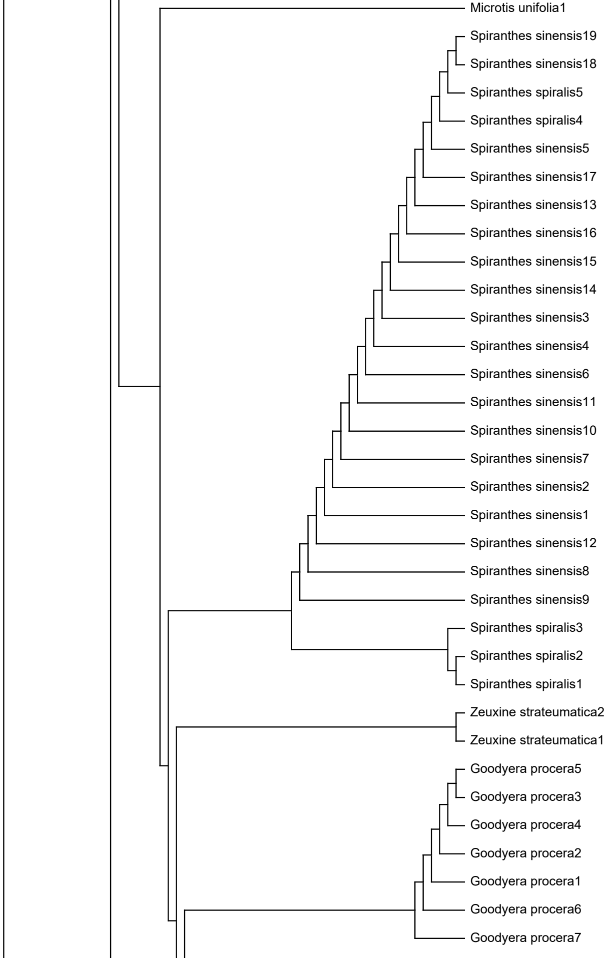
Microtis unifolia1
Spiranthes sinensis19
Spiranthes sinensis18
Spiranthes spiralis5
Spiranthes spiralis4
Spiranthes sinensis5
Spiranthes sinensis17
Spiranthes sinensis13
Spiranthes sinensis16
Spiranthes sinensis15
Spiranthes sinensis14
Spiranthes sinensis3
Spiranthes sinensis4
Spiranthes sinensis6
Spiranthes sinensis11
Spiranthes sinensis10
Spiranthes sinensis7
Spiranthes sinensis2
Spiranthes sinensis1
Spiranthes sinensis12
Spiranthes sinensis8
Spiranthes sinensis9
Spiranthes spiralis3
Spiranthes spiralis2
Spiranthes spiralis1
Zeuxine strateumatica2
Zeuxine strateumatica1
Goodyera procera5
Goodyera procera3
Goodyera procera4
Goodyera procera2
Goodyera procera1
Goodyera procera6
Goodyera procera7

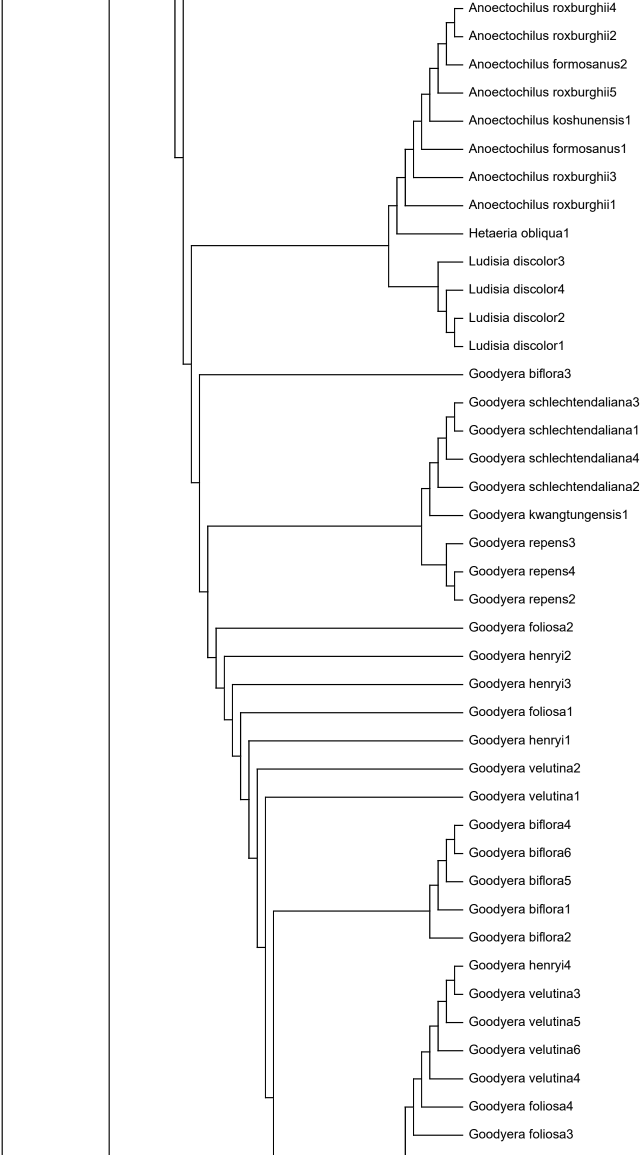
Anoectochilus roxburghii4
Anoectochilus roxburghii2
Anoectochilus formosanus2
Anoectochilus roxburghii5
Anoectochilus koshunensis1
Anoectochilus formosanus1
Anoectochilus roxburghii3
Anoectochilus roxburghii1
Hetaeria obliqua1
Ludisia discolor3
Ludisia discolor4
Ludisia discolor2
Ludisia discolor1
Goodyera biflora3
Goodyera schlechtendaliana3
Goodyera schlechtendaliana1
Goodyera schlechtendaliana4
Goodyera schlechtendaliana2
Goodyera kwangtungensis1
Goodyera repens3
Goodyera repens4
Goodyera repens2
Goodyera foliosa2
Goodyera henryi2
Goodyera henryi3
Goodyera foliosa1
Goodyera henryi1
Goodyera velutina2
Goodyera velutina1
Goodyera biflora4
Goodyera biflora6
Goodyera biflora5
Goodyera biflora1
Goodyera biflora2
Goodyera henryi4
Goodyera velutina3
Goodyera velutina5
Goodyera velutina6
Goodyera velutina4
Goodyera foliosa4
Goodyera foliosa3

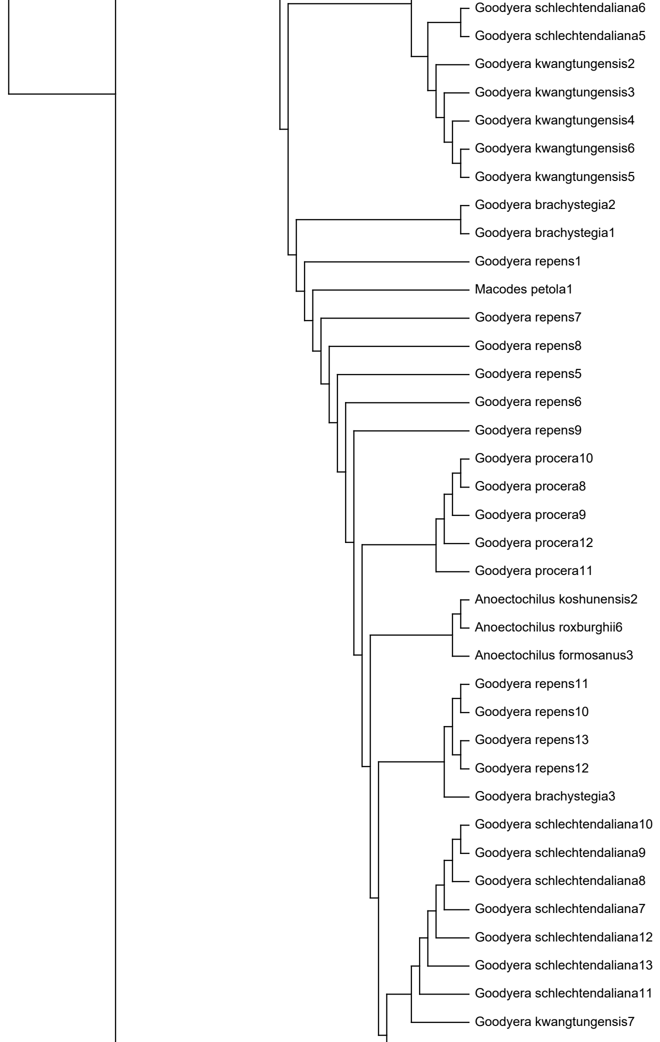
Goodyera schlechtendaliana6
Goodyera schlechtendaliana5
Goodyera kwangtungensis2
Goodyera kwangtungensis3
Goodyera kwangtungensis4
Goodyera kwangtungensis6
Goodyera kwangtungensis5
Goodyera brachystegia2
Goodyera brachystegia1
Goodyera repens1
Macodes petola1
Goodyera repens7
Goodyera repens8
Goodyera repens5
Goodyera repens6
Goodyera repens9
Goodyera procera10
Goodyera procera8
Goodyera procera9
Goodyera procera12
Goodyera procera11
Anoectochilus koshunensis2
Anoectochilus roxburghii6
Anoectochilus formosanus3
Goodyera repens11
Goodyera repens10
Goodyera repens13
Goodyera repens12
Goodyera brachystegia3
Goodyera schlechtendaliana10
Goodyera schlechtendaliana9
Goodyera schlechtendaliana8
Goodyera schlechtendaliana7
Goodyera schlechtendaliana12
Goodyera schlechtendaliana13
Goodyera schlechtendaliana11
Goodyera kwangtungensis7

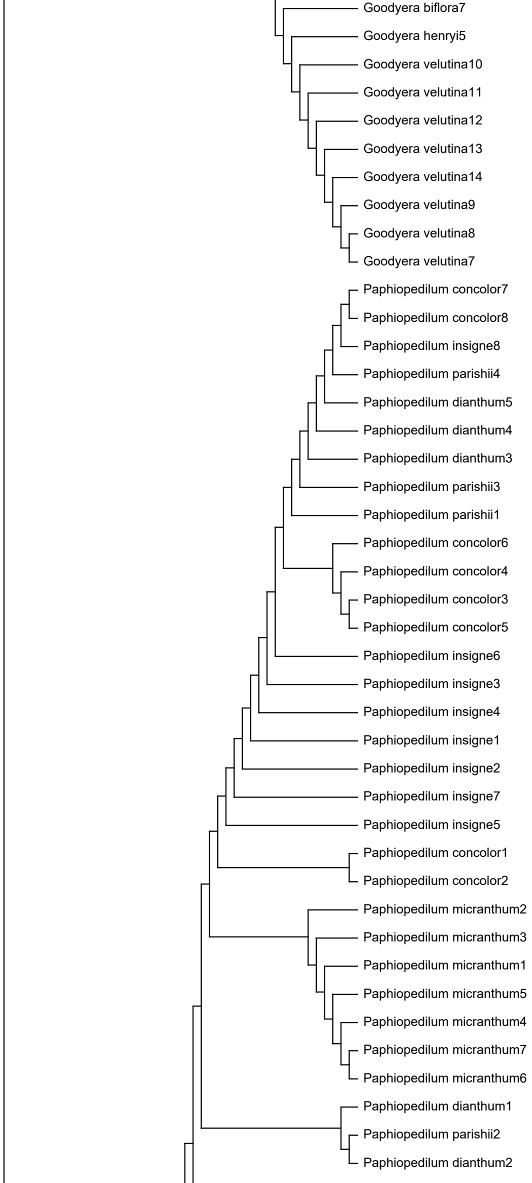

Goodyera biflora7
Goodyera henryi5
Goodyera velutina10
Goodyera velutina11
Goodyera velutina12
Goodyera velutina13
Goodyera velutina14
Goodyera velutina9
Goodyera velutina8
Goodyera velutina7
Paphiopedilum concolor7
Paphiopedilum concolor8
Paphiopedilum insigne8
Paphiopedilum parishii4
Paphiopedilum dianthum5
Paphiopedilum dianthum4
Paphiopedilum dianthum3
Paphiopedilum parishii3
Paphiopedilum parishii1
Paphiopedilum concolor6
Paphiopedilum concolor4
Paphiopedilum concolor3
Paphiopedilum concolor5
Paphiopedilum insigne6
Paphiopedilum insigne3
Paphiopedilum insigne4
Paphiopedilum insigne1
Paphiopedilum insigne2
Paphiopedilum insigne7
Paphiopedilum insigne5
Paphiopedilum concolor1
Paphiopedilum concolor2
Paphiopedilum micranthum2
Paphiopedilum micranthum3
Paphiopedilum micranthum1
Paphiopedilum micranthum5
Paphiopedilum micranthum4
Paphiopedilum micranthum7
Paphiopedilum micranthum6
Paphiopedilum dianthum1
Paphiopedilum parishii2
Paphiopedilum dianthum2

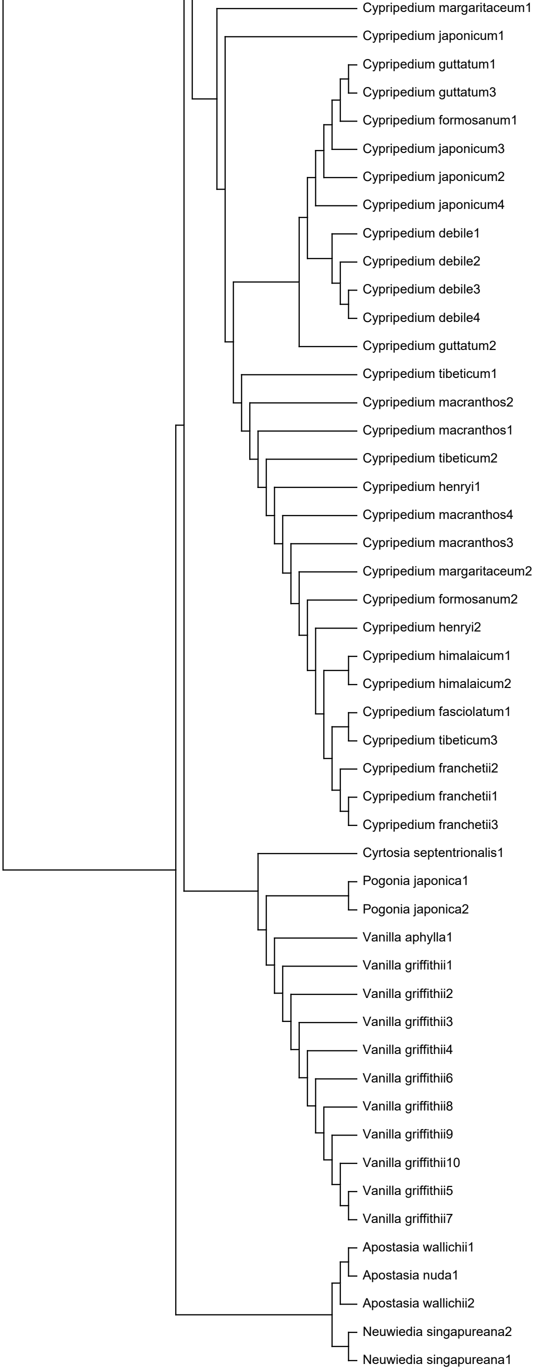
Cypripedium margaritaceum1
Cypripedium japonicum1
Cypripedium guttatum1
Cypripedium guttatum3
Cypripedium formosanum1
Cypripedium japonicum3
Cypripedium japonicum2
Cypripedium japonicum4
Cypripedium debile1
Cypripedium debile2
Cypripedium debile3
Cypripedium debile4
Cypripedium guttatum2
Cypripedium tibeticum1
Cypripedium macranthos2
Cypripedium macranthos1
Cypripedium tibeticum2
Cypripedium henryi1
Cypripedium macranthos4
Cypripedium macranthos3
Cypripedium margaritaceum2
Cypripedium formosanum2
Cypripedium henryi2
Cypripedium himalaicum1
Cypripedium himalaicum2
Cypripedium fasciolatum1
Cypripedium tibeticum3
Cypripedium franchetii2
Cypripedium franchetii1
Cypripedium franchetii3
Cyrtosia septentrionalis1
Pogonia japonica1
Pogonia japonica2
Vanilla aphylla1
Vanilla griffithii1
Vanilla griffithii2
Vanilla griffithii3
Vanilla griffithii4
Vanilla griffithii6
Vanilla griffithii8
Vanilla griffithii9
Vanilla griffithii10
Vanilla griffithii5
Vanilla griffithii7
Apostasia wallichii1
Apostasia nuda1
Apostasia wallichii2
Neuwiedia singapureana2
Neuwiedia singapureana1
