# Supplementary material for: DNA barcoding of medicinal orchids in Asia
Source: Sci Rep. 2021 Dec 8;11:23651. doi: 10.1038/s41598-021-03025-0 (PMC8654824; doi:10.1038/s41598-021-03025-0)
Supplement: Supplementary file 10 — Supplementary Figure S10. [file 41598_2021_3025_MOESM10_ESM.pdf]

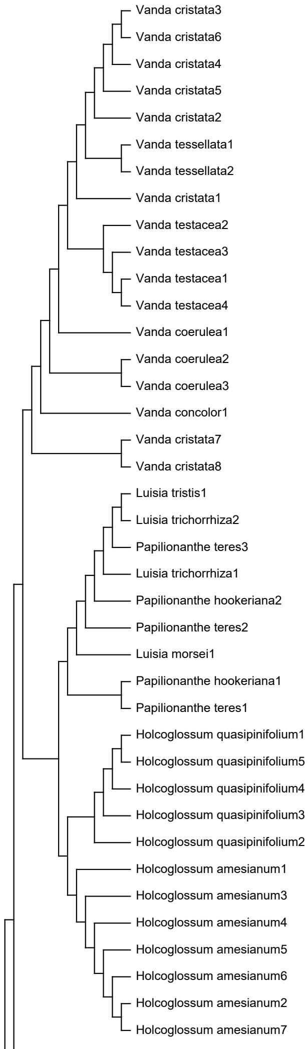
Vanda cristata3
Vanda cristata6
Vanda cristata4
Vanda cristata5
Vanda cristata2
Vanda tessellata1
Vanda tessellata2
Vanda cristata1
Vanda testacea2
Vanda testacea3
Vanda testacea1
Vanda testacea4
Vanda coerulea1
Vanda coerulea2
Vanda coerulea3
Vanda concolor1
Vanda cristata7
Vanda cristata8
Luisia tristis1
Luisia trichorrhiza2
Papilionanthe teres3
Luisia trichorrhiza1
Papilionanthe hookeriana2
Papilionanthe teres2
Luisia morsei1
Papilionanthe hookeriana1
Papilionanthe teres1
Holcoglossum quasipinifolium1
Holcoglossum quasipinifolium5
Holcoglossum quasipinifolium4
Holcoglossum quasipinifolium3
Holcoglossum quasipinifolium2
Holcoglossum amesianum1
Holcoglossum amesianum3
Holcoglossum amesianum4
Holcoglossum amesianum5
Holcoglossum amesianum6
Holcoglossum amesianum2
Holcoglossum amesianum7

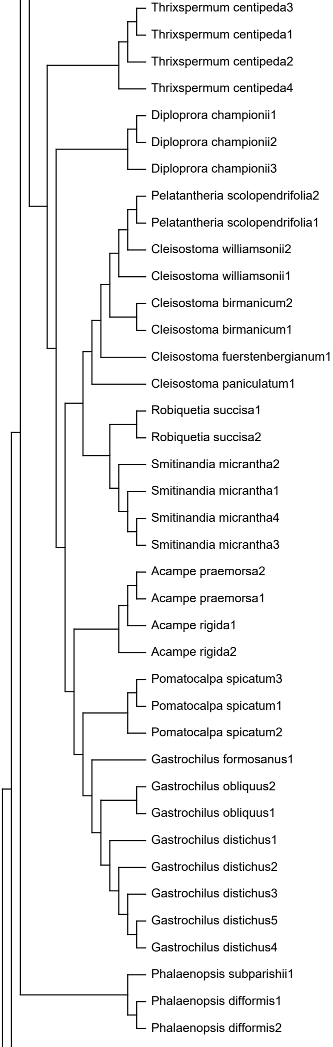
Thrixspermum centipeda3
Thrixspermum centipeda1
Thrixspermum centipeda2
Thrixspermum centipeda4
Diploprora championii1
Diploprora championii2
Diploprora championii3
Pelatantheria scolopendrifolia2
Pelatantheria scolopendrifolia1
Cleisostoma williamsonii2
Cleisostoma williamsonii1
Cleisostoma birmanicum2
Cleisostoma birmanicum1
Cleisostoma fuerstenbergianum1
Cleisostoma paniculatum1
Robiquetia succisa1
Robiquetia succisa2
Smitinandia micrantha2
Smitinandia micrantha1
Smitinandia micrantha4
Smitinandia micrantha3
Acampe praemorsa2
Acampe praemorsa1
Acampe rigida1
Acampe rigida2
Pomatocalpa spicatum3
Pomatocalpa spicatum1
Pomatocalpa spicatum2
Gastrochilus formosanus1
Gastrochilus obliquus2
Gastrochilus obliquus1
Gastrochilus distichus1
Gastrochilus distichus2
Gastrochilus distichus3
Gastrochilus distichus5
Gastrochilus distichus4
Phalaenopsis subparishii1
Phalaenopsis difformis1
Phalaenopsis difformis2

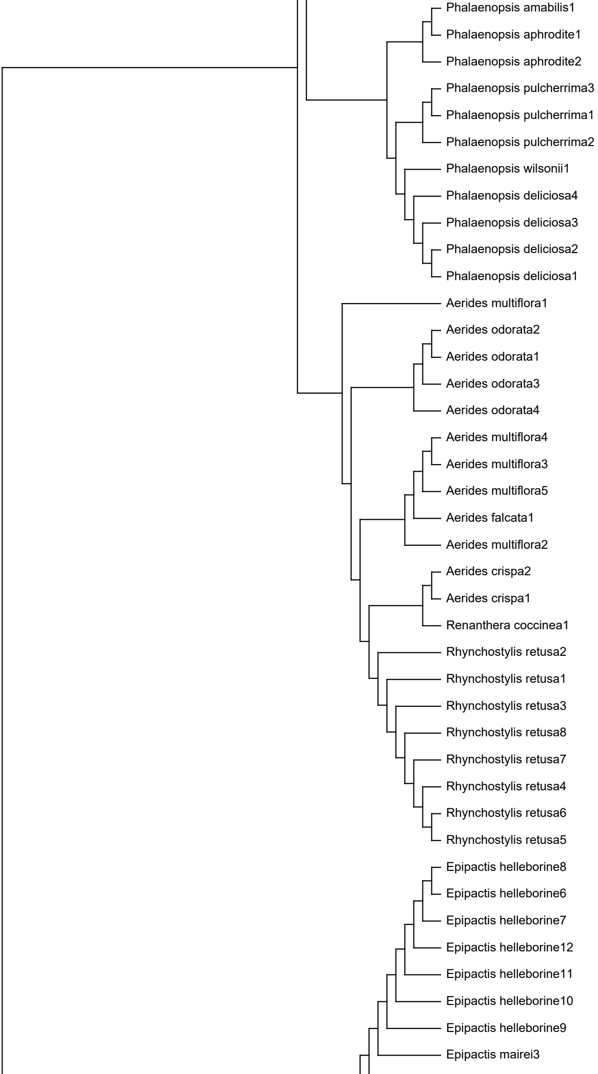
Phalaenopsis amabilis1
Phalaenopsis aphrodite1
Phalaenopsis aphrodite2
Phalaenopsis pulcherrima3
Phalaenopsis pulcherrima1
Phalaenopsis pulcherrima2
Phalaenopsis wilsonii1
Phalaenopsis deliciosa4
Phalaenopsis deliciosa3
Phalaenopsis deliciosa2
Phalaenopsis deliciosa1
Aerides multiflora1
Aerides odorata2
Aerides odorata1
Aerides odorata3
Aerides odorata4
Aerides multiflora4
Aerides multiflora3
Aerides multiflora5
Aerides falcata1
Aerides multiflora2
Aerides crispa2
Aerides crispa1
Renanthera coccinea1
Rhynchostylis retusa2
Rhynchostylis retusa1
Rhynchostylis retusa3
Rhynchostylis retusa8
Rhynchostylis retusa7
Rhynchostylis retusa4
Rhynchostylis retusa6
Rhynchostylis retusa5
Epipactis helleborine8
Epipactis helleborine6
Epipactis helleborine7
Epipactis helleborine12
Epipactis helleborine11
Epipactis helleborine10
Epipactis helleborine9
Epipactis mairei3

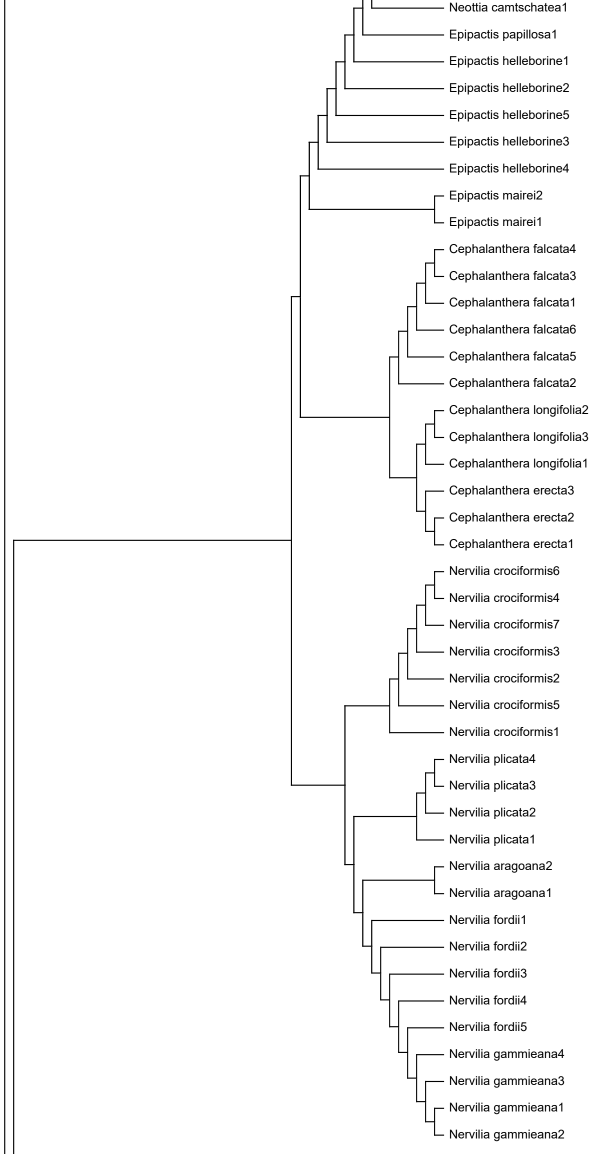
Neottia camtschatea1
Epipactis papillosa1
Epipactis helleborine1
Epipactis helleborine2
Epipactis helleborine5
Epipactis helleborine3
Epipactis helleborine4
Epipactis mairei2
Epipactis mairei1
Cephalanthera falcata4
Cephalanthera falcata3
Cephalanthera falcata1
Cephalanthera falcata6
Cephalanthera falcata5
Cephalanthera falcata2
Cephalanthera longifolia2
Cephalanthera longifolia3
Cephalanthera longifolia1
Cephalanthera erecta3
Cephalanthera erecta2
Cephalanthera erecta1
Nervilia crociformis6
Nervilia crociformis4
Nervilia crociformis7
Nervilia crociformis3
Nervilia crociformis2
Nervilia crociformis5
Nervilia crociformis1
Nervilia plicata4
Nervilia plicata3
Nervilia plicata2
Nervilia plicata1
Nervilia aragoana2
Nervilia aragoana1
Nervilia fordii1
Nervilia fordii2
Nervilia fordii3
Nervilia fordii4
Nervilia fordii5
Nervilia gammieana4
Nervilia gammieana3
Nervilia gammieana1
Nervilia gammieana2

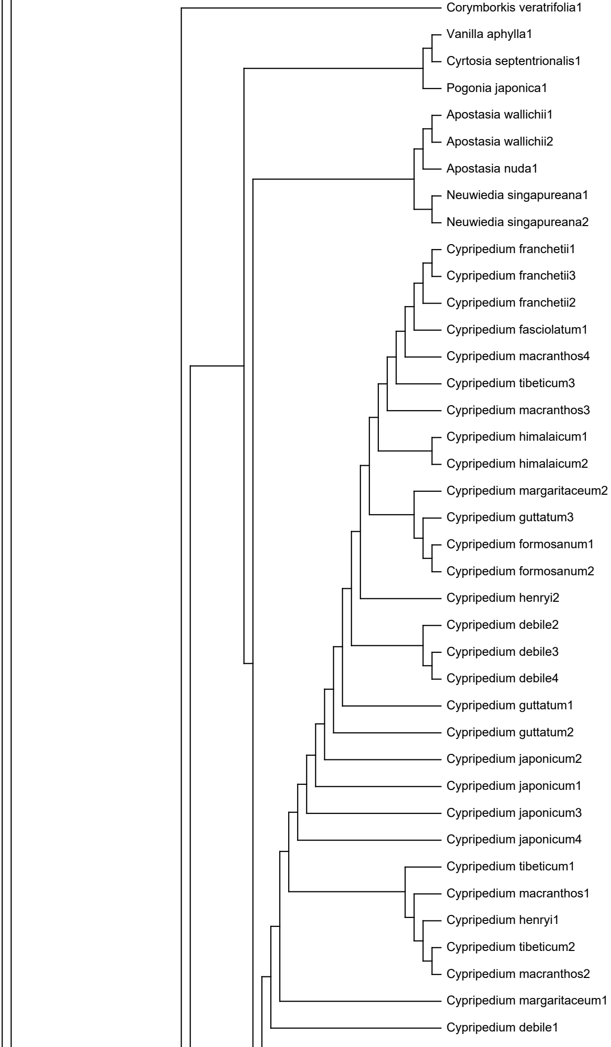
Corymborkis veratrifolia1
Vanilla aphylla1
Cyrtosia septentrionalis1
Pogonia japonica1
Apostasia wallichii1
Apostasia wallichii2
Apostasia nuda1
Neuwiedia singapureana1
Neuwiedia singapureana2
Cypripedium franchetii1
Cypripedium franchetii3
Cypripedium franchetii2
Cypripedium fasciolatum1
Cypripedium macranthos4
Cypripedium tibeticum3
Cypripedium macranthos3
Cypripedium himalaicum1
Cypripedium himalaicum2
Cypripedium margaritaceum2
Cypripedium guttatum3
Cypripedium formosanum1
Cypripedium formosanum2
Cypripedium henryi2
Cypripedium debile2
Cypripedium debile3
Cypripedium debile4
Cypripedium guttatum1
Cypripedium guttatum2
Cypripedium japonicum2
Cypripedium japonicum1
Cypripedium japonicum3
Cypripedium japonicum4
Cypripedium tibeticum1
Cypripedium macranthos1
Cypripedium henryi1
Cypripedium tibeticum2
Cypripedium macranthos2
Cypripedium margaritaceum1
Cypripedium debile1

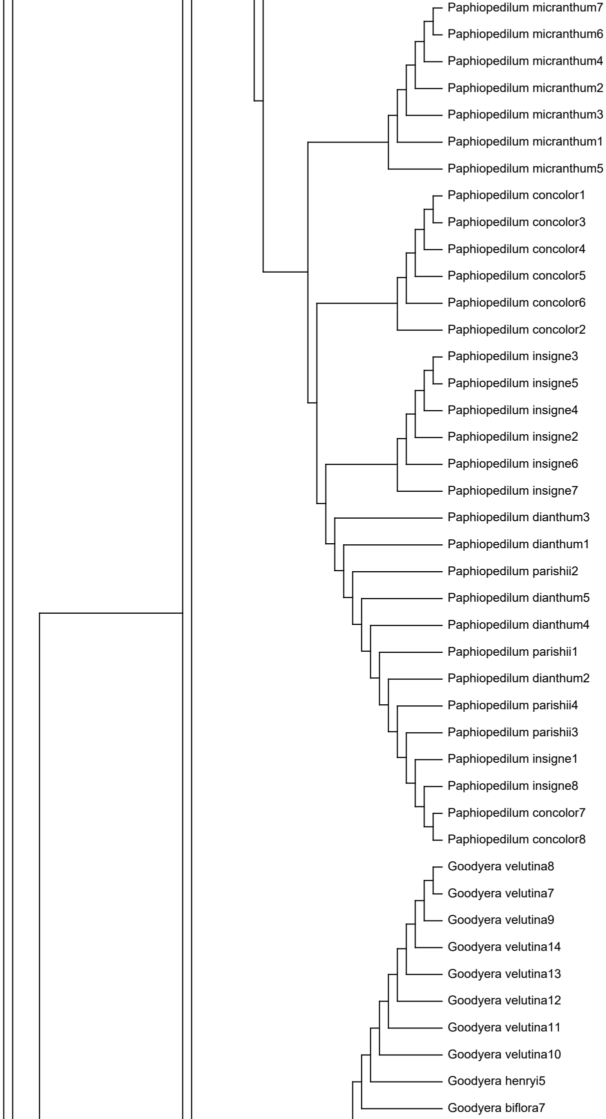

Paphiopedilum micranthum7
Paphiopedilum micranthum6
Paphiopedilum micranthum4
Paphiopedilum micranthum2
Paphiopedilum micranthum3
Paphiopedilum micranthum1
Paphiopedilum micranthum5
Paphiopedilum concolor1
Paphiopedilum concolor3
Paphiopedilum concolor4
Paphiopedilum concolor5
Paphiopedilum concolor6
Paphiopedilum concolor2
Paphiopedilum insigne3
Paphiopedilum insigne5
Paphiopedilum insigne4
Paphiopedilum insigne2
Paphiopedilum insigne6
Paphiopedilum insigne7
Paphiopedilum dianthum3
Paphiopedilum dianthum1
Paphiopedilum parishii2
Paphiopedilum dianthum5
Paphiopedilum dianthum4
Paphiopedilum parishii1
Paphiopedilum dianthum2
Paphiopedilum parishii4
Paphiopedilum parishii3
Paphiopedilum insigne1
Paphiopedilum insigne8
Paphiopedilum concolor7
Paphiopedilum concolor8
Goodyera velutina8
Goodyera velutina7
Goodyera velutina9
Goodyera velutina14
Goodyera velutina13
Goodyera velutina12
Goodyera velutina11
Goodyera velutina10
Goodyera henryi5
Goodyera biflora7

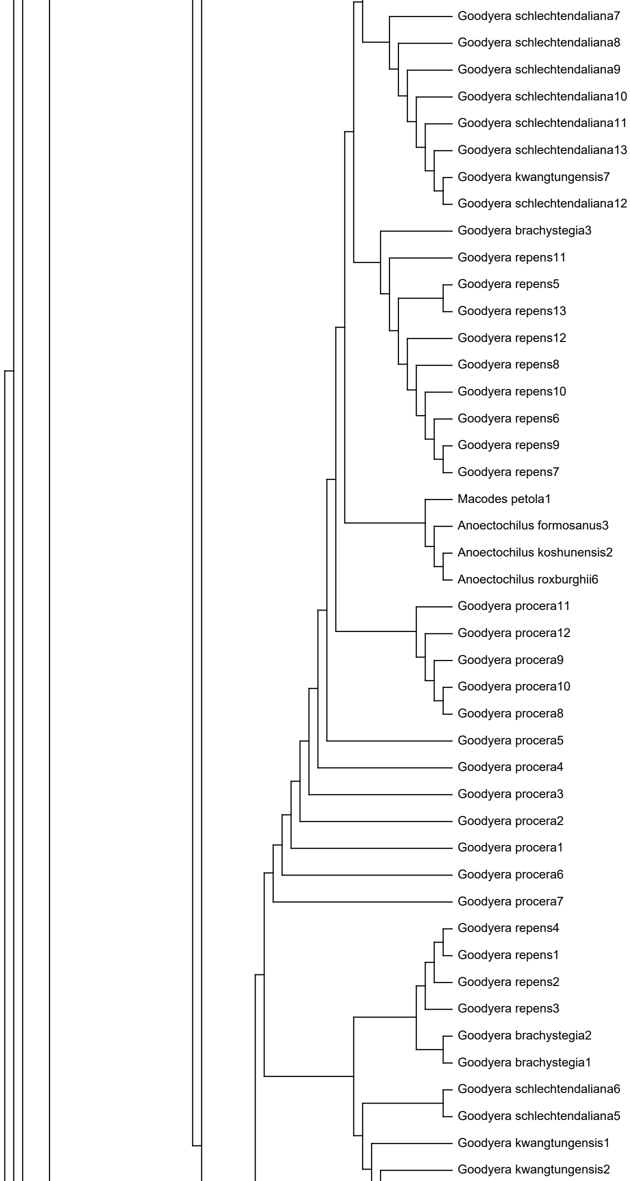
Goodyera schlechtendaliana7
Goodyera schlechtendaliana8
Goodyera schlechtendaliana9
Goodyera schlechtendaliana10
Goodyera schlechtendaliana11
Goodyera schlechtendaliana13
Goodyera kwangtungensis7
Goodyera schlechtendaliana12
Goodyera brachystegia3
Goodyera repens11
Goodyera repens5
Goodyera repens13
Goodyera repens12
Goodyera repens8
Goodyera repens10
Goodyera repens6
Goodyera repens9
Goodyera repens7
Macodes petola1
Anoectochilus formosanus3
Anoectochilus koshunensis2
Anoectochilus roxburghii6
Goodyera procera11
Goodyera procera12
Goodyera procera9
Goodyera procera10
Goodyera procera8
Goodyera procera5
Goodyera procera4
Goodyera procera3
Goodyera procera2
Goodyera procera1
Goodyera procera6
Goodyera procera7
Goodyera repens4
Goodyera repens1
Goodyera repens2
Goodyera repens3
Goodyera brachystegia2
Goodyera brachystegia1
Goodyera schlechtendaliana6
Goodyera schlechtendaliana5
Goodyera kwangtungensis1
Goodyera kwangtungensis2

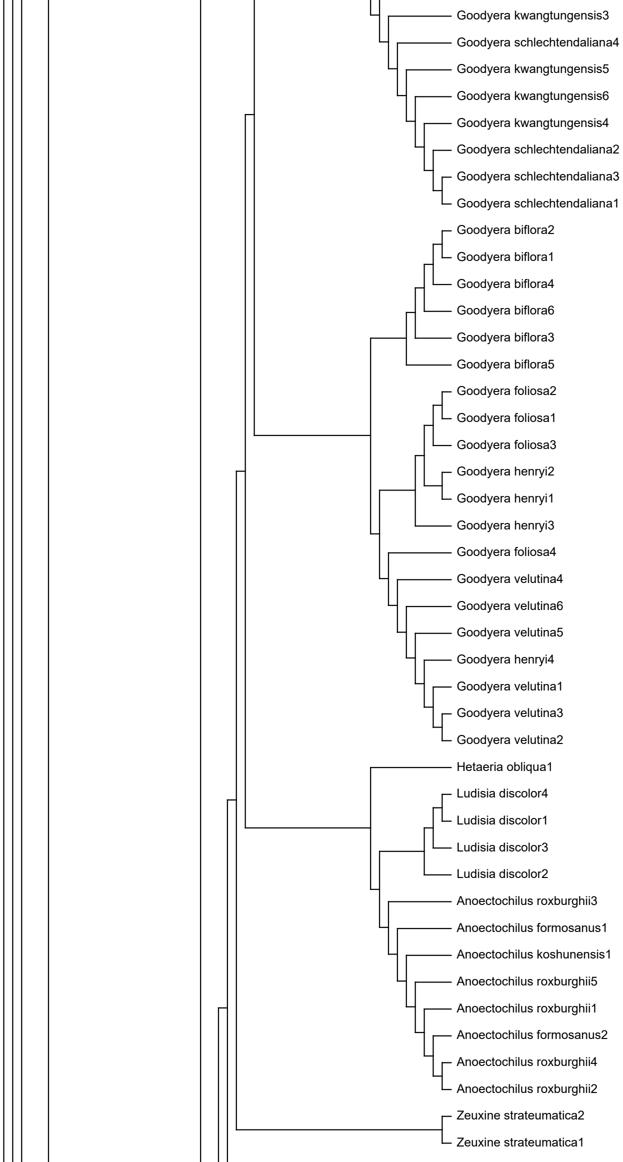

Goodyera kwangtungensis3
Goodyera schlechtendaliana4
Goodyera kwangtungensis5
Goodyera kwangtungensis6
Goodyera kwangtungensis4
Goodyera schlechtendaliana2
Goodyera schlechtendaliana3
Goodyera schlechtendaliana1
Goodyera biflora2
Goodyera biflora1
Goodyera biflora4
Goodyera biflora6
Goodyera biflora3
Goodyera biflora5
Goodyera foliosa2
Goodyera foliosa1
Goodyera foliosa3
Goodyera henryi2
Goodyera henryi1
Goodyera henryi3
Goodyera foliosa4
Goodyera velutina4
Goodyera velutina6
Goodyera velutina5
Goodyera henryi4
Goodyera velutina1
Goodyera velutina3
Goodyera velutina2
Hetaeria obliqua1
Ludisia discolor4
Ludisia discolor1
Ludisia discolor3
Ludisia discolor2
Anoectochilus roxburghii3
Anoectochilus formosanus1
Anoectochilus koshunensis1
Anoectochilus roxburghii5
Anoectochilus roxburghii1
Anoectochilus formosanus2
Anoectochilus roxburghii4
Anoectochilus roxburghii2
Zeuxine strateumatica2
Zeuxine strateumatica1

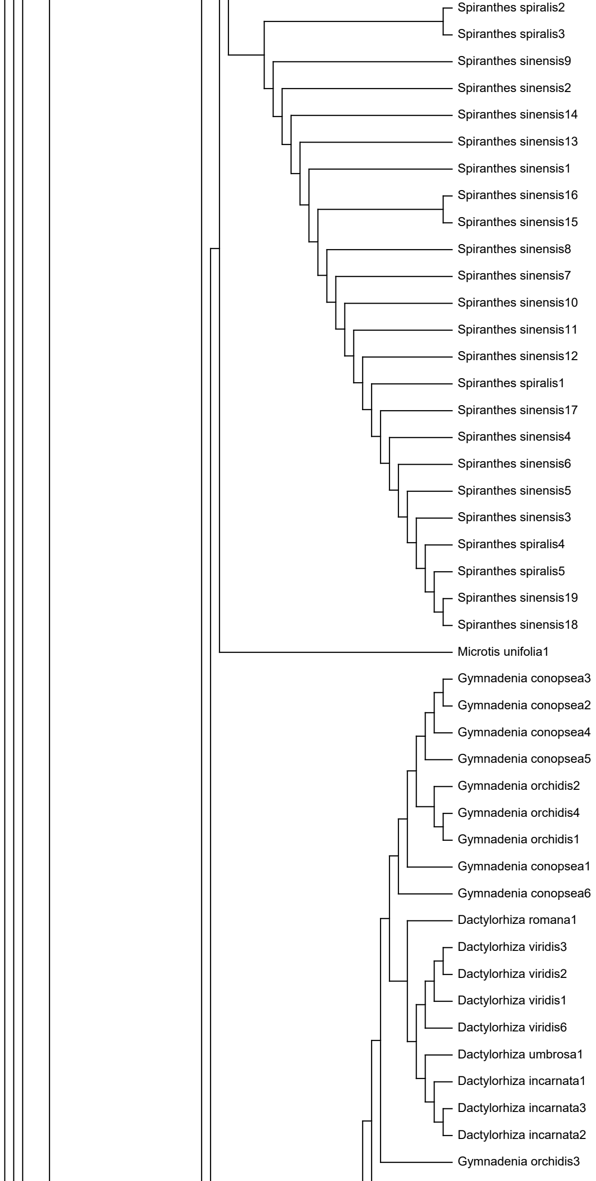
Spiranthes spiralis2
Spiranthes spiralis3
Spiranthes sinensis9
Spiranthes sinensis2
Spiranthes sinensis14
Spiranthes sinensis13
Spiranthes sinensis1
Spiranthes sinensis16
Spiranthes sinensis15
Spiranthes sinensis8
Spiranthes sinensis7
Spiranthes sinensis10
Spiranthes sinensis11
Spiranthes sinensis12
Spiranthes spiralis1
Spiranthes sinensis17
Spiranthes sinensis4
Spiranthes sinensis6
Spiranthes sinensis5
Spiranthes sinensis3
Spiranthes spiralis4
Spiranthes spiralis5
Spiranthes sinensis19
Spiranthes sinensis18
Microtis unifolia1
Gymnadenia conopsea3
Gymnadenia conopsea2
Gymnadenia conopsea4
Gymnadenia conopsea5
Gymnadenia orchidis2
Gymnadenia orchidis4
Gymnadenia orchidis1
Gymnadenia conopsea1
Gymnadenia conopsea6
Dactylorhiza romana1
Dactylorhiza viridis3
Dactylorhiza viridis2
Dactylorhiza viridis1
Dactylorhiza viridis6
Dactylorhiza umbrosa1
Dactylorhiza incarnata1
Dactylorhiza incarnata3
Dactylorhiza incarnata2
Gymnadenia orchidis3

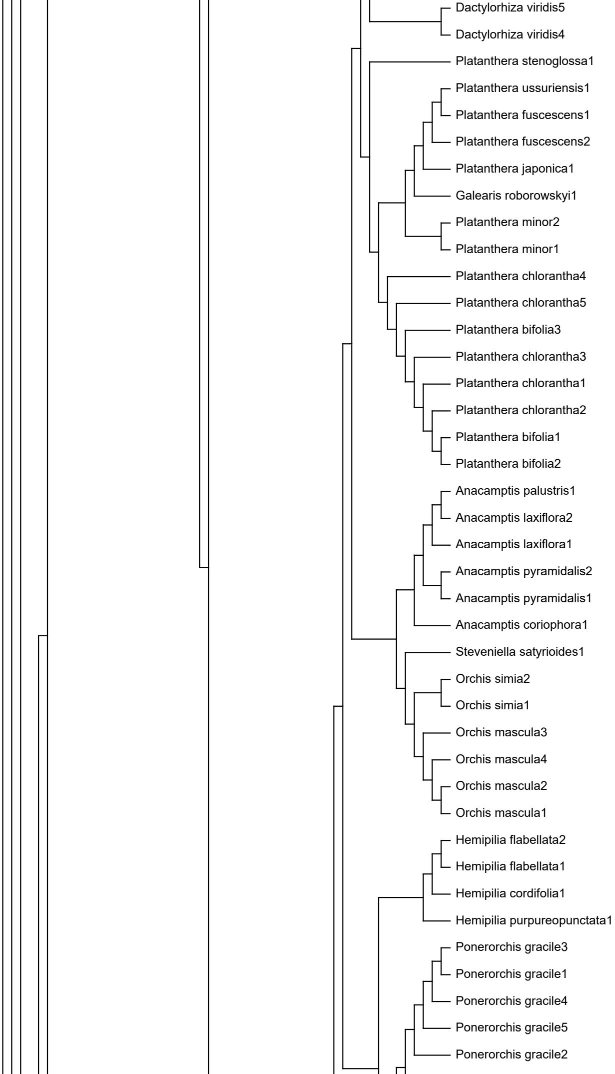
Dactylorhiza viridis5
Dactylorhiza viridis4
Platanthera stenoglossa1
Platanthera ussuriensis1
Platanthera fuscescens1
Platanthera fuscescens2
Platanthera japonica1
Galearis roborowskyi1
Platanthera minor2
Platanthera minor1
Platanthera chlorantha4
Platanthera chlorantha5
Platanthera bifolia3
Platanthera chlorantha3
Platanthera chlorantha1
Platanthera chlorantha2
Platanthera bifolia1
Platanthera bifolia2
Anacamptis palustris1
Anacamptis laxiflora2
Anacamptis laxiflora1
Anacamptis pyramidalis2
Anacamptis pyramidalis1
Anacamptis coriophora1
Steveniella satyrioides1
Orchis simia2
Orchis simia1
Orchis mascula3
Orchis mascula4
Orchis mascula2
Orchis mascula1
Hemipilia flabellata2
Hemipilia flabellata1
Hemipilia cordifolia1
Hemipilia purpureopunctata1
Ponerorchis gracile3
Ponerorchis gracile1
Ponerorchis gracile4
Ponerorchis gracile5
Ponerorchis gracile2

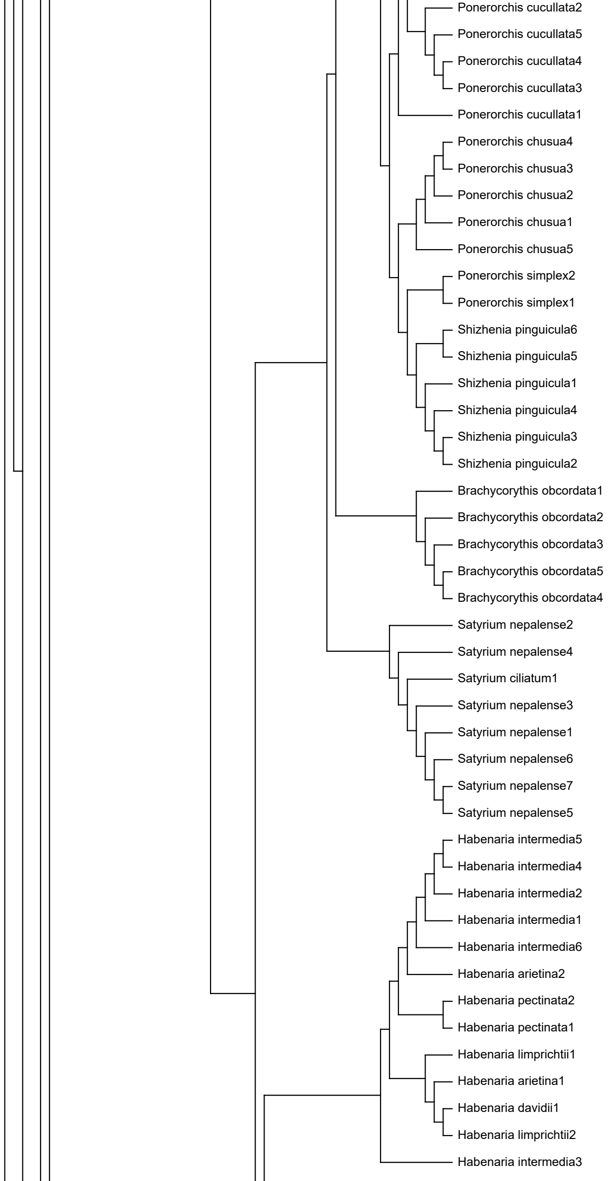
Ponerorchis cucullata2
Ponerorchis cucullata5
Ponerorchis cucullata4
Ponerorchis cucullata3
Ponerorchis cucullata1
Ponerorchis chusua4
Ponerorchis chusua3
Ponerorchis chusua2
Ponerorchis chusua1
Ponerorchis chusua5
Ponerorchis simplex2
Ponerorchis simplex1
Shizhenia pinguicula6
Shizhenia pinguicula5
Shizhenia pinguicula1
Shizhenia pinguicula4
Shizhenia pinguicula3
Shizhenia pinguicula2
Brachycorythis obcordata1
Brachycorythis obcordata2
Brachycorythis obcordata3
Brachycorythis obcordata5
Brachycorythis obcordata4
Satyrium nepalense2
Satyrium nepalense4
Satyrium ciliatum1
Satyrium nepalense3
Satyrium nepalense1
Satyrium nepalense6
Satyrium nepalense7
Satyrium nepalense5
Habenaria intermedia5
Habenaria intermedia4
Habenaria intermedia2
Habenaria intermedia1
Habenaria intermedia6
Habenaria arietina2
Habenaria pectinata2
Habenaria pectinata1
Habenaria limprichtii1
Habenaria arietina1
Habenaria davidii1
Habenaria limprichtii2
Habenaria intermedia3

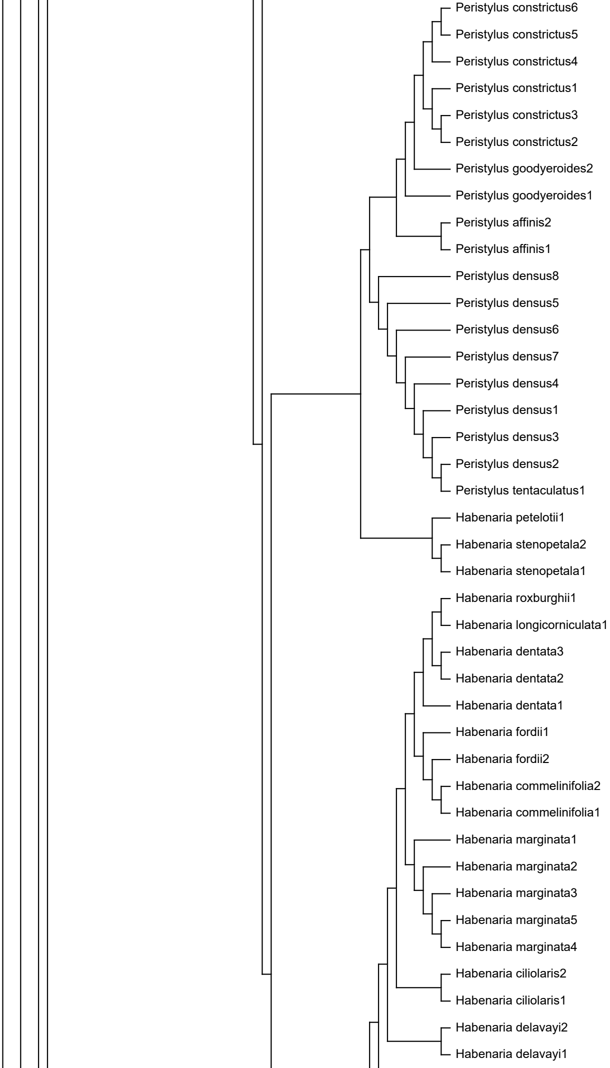
Peristylus constrictus6
Peristylus constrictus5
Peristylus constrictus4
Peristylus constrictus1
Peristylus constrictus3
Peristylus constrictus2
Peristylus goodyeroides2
Peristylus goodyeroides1
Peristylus affinis2
Peristylus affinis1
Peristylus densus8
Peristylus densus5
Peristylus densus6
Peristylus densus7
Peristylus densus4
Peristylus densus1
Peristylus densus3
Peristylus densus2
Peristylus tentaculatus1
Habenaria petelotii1
Habenaria stenopetala2
Habenaria stenopetala1
Habenaria roxburghii1
Habenaria longicorniculata1
Habenaria dentata3
Habenaria dentata2
Habenaria dentata1
Habenaria fordii1
Habenaria fordii2
Habenaria commelinifolia2
Habenaria commelinifolia1
Habenaria marginata1
Habenaria marginata2
Habenaria marginata3
Habenaria marginata5
Habenaria marginata4
Habenaria ciliolaris2
Habenaria ciliolaris1
Habenaria delavayi2
Habenaria delavayi1

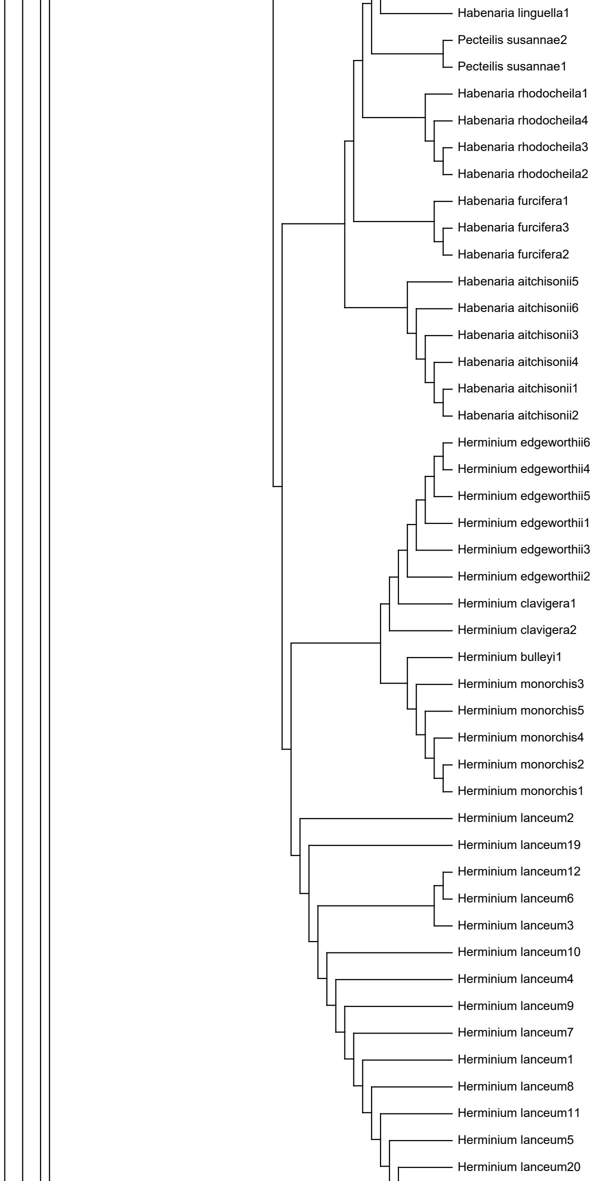

Habenaria linguella1
Pecteilis susannae2
Pecteilis susannae1
Habenaria rhodocheila1
Habenaria rhodocheila4
Habenaria rhodocheila3
Habenaria rhodocheila2
Habenaria furcifera1
Habenaria furcifera3
Habenaria furcifera2
Habenaria aitchisonii5
Habenaria aitchisonii6
Habenaria aitchisonii3
Habenaria aitchisonii4
Habenaria aitchisonii1
Habenaria aitchisonii2
Herminium edgeworthii6
Herminium edgeworthii4
Herminium edgeworthii5
Herminium edgeworthii1
Herminium edgeworthii3
Herminium edgeworthii2
Herminium clavigera1
Herminium clavigera2
Herminium bulleyi1
Herminium monorchis3
Herminium monorchis5
Herminium monorchis4
Herminium monorchis2
Herminium monorchis1
Herminium lanceum2
Herminium lanceum19
Herminium lanceum12
Herminium lanceum6
Herminium lanceum3
Herminium lanceum10
Herminium lanceum4
Herminium lanceum9
Herminium lanceum7
Herminium lanceum1
Herminium lanceum8
Herminium lanceum11
Herminium lanceum5
Herminium lanceum20

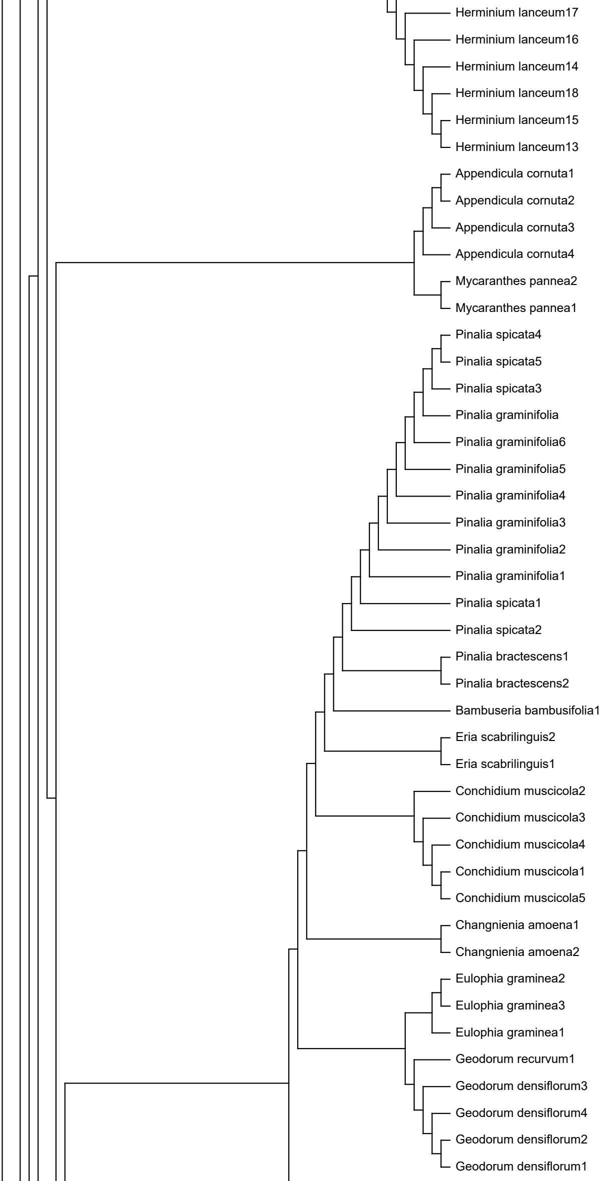
Herminium lanceum17
Herminium lanceum16
Herminium lanceum14
Herminium lanceum18
Herminium lanceum15
Herminium lanceum13
Appendicula cornuta1
Appendicula cornuta2
Appendicula cornuta3
Appendicula cornuta4
Mycaranthes pannea2
Mycaranthes pannea1
Pinalia spicata4
Pinalia spicata5
Pinalia spicata3
Pinalia graminifolia
Pinalia graminifolia6
Pinalia graminifolia5
Pinalia graminifolia4
Pinalia graminifolia3
Pinalia graminifolia2
Pinalia graminifolia1
Pinalia spicata1
Pinalia spicata2
Pinalia bractescens1
Pinalia bractescens2
Bambuseria bambusifolia1
Eria scabrilinguis2
Eria scabrilinguis1
Conchidium muscicola2
Conchidium muscicola3
Conchidium muscicola4
Conchidium muscicola1
Conchidium muscicola5
Changnienia amoena1
Changnienia amoena2
Eulophia graminea2
Eulophia graminea3
Eulophia graminea1
Geodorum recurvum1
Geodorum densiflorum3
Geodorum densiflorum4
Geodorum densiflorum2
Geodorum densiflorum1

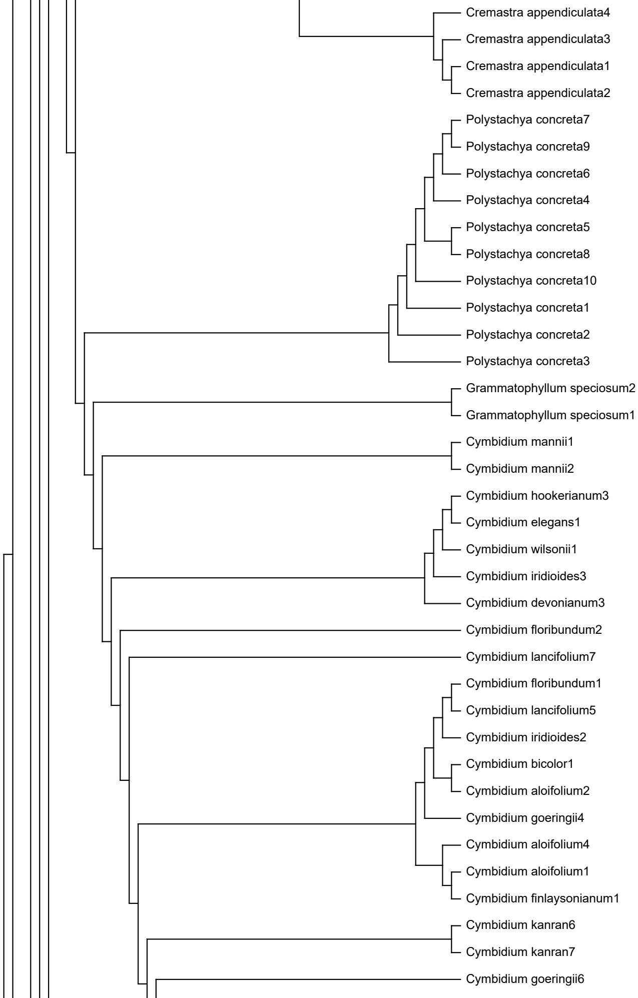

Cremastra appendiculata4
Cremastra appendiculata3
Cremastra appendiculata1
Cremastra appendiculata2
Polystachya concreta7
Polystachya concreta9
Polystachya concreta6
Polystachya concreta4
Polystachya concreta5
Polystachya concreta8
Polystachya concreta10
Polystachya concreta1
Polystachya concreta2
Polystachya concreta3
Grammatophyllum speciosum2
Grammatophyllum speciosum1
Cymbidium mannii1
Cymbidium mannii2
Cymbidium hookerianum3
Cymbidium elegans1
Cymbidium wilsonii1
Cymbidium iridioides3
Cymbidium devonianum3
Cymbidium floribundum2
Cymbidium lancifolium7
Cymbidium floribundum1
Cymbidium lancifolium5
Cymbidium iridioides2
Cymbidium bicolor1
Cymbidium aloifolium2
Cymbidium goeringii4
Cymbidium aloifolium4
Cymbidium aloifolium1
Cymbidium finlaysonianum1
Cymbidium kanran6
Cymbidium kanran7
Cymbidium goeringii6

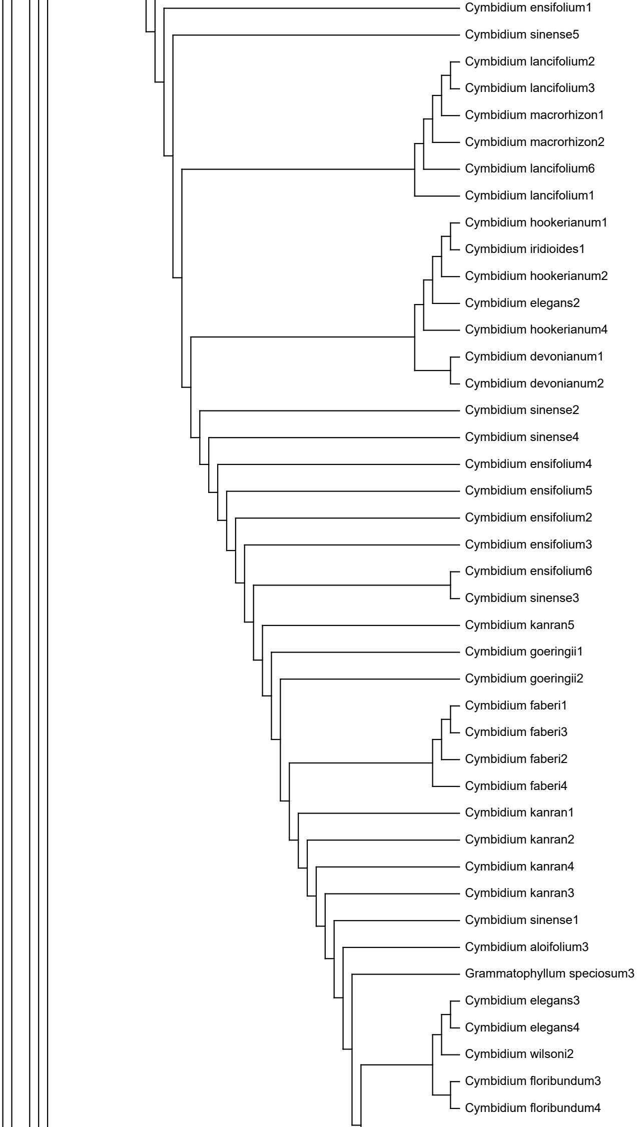
Cymbidium ensifolium1
Cymbidium sinense5
Cymbidium lancifolium2
Cymbidium lancifolium3
Cymbidium macrorhizon1
Cymbidium macrorhizon2
Cymbidium lancifolium6
Cymbidium lancifolium1
Cymbidium hookerianum1
Cymbidium iridioides1
Cymbidium hookerianum2
Cymbidium elegans2
Cymbidium hookerianum4
Cymbidium devonianum1
Cymbidium devonianum2
Cymbidium sinense2
Cymbidium sinense4
Cymbidium ensifolium4
Cymbidium ensifolium5
Cymbidium ensifolium2
Cymbidium ensifolium3
Cymbidium ensifolium6
Cymbidium sinense3
Cymbidium kanran5
Cymbidium goeringii1
Cymbidium goeringii2
Cymbidium faberi1
Cymbidium faberi3
Cymbidium faberi2
Cymbidium faberi4
Cymbidium kanran1
Cymbidium kanran2
Cymbidium kanran4
Cymbidium kanran3
Cymbidium sinense1
Cymbidium aloifolium3
Grammatophyllum speciosum3
Cymbidium elegans3
Cymbidium elegans4
Cymbidium wilsoni2
Cymbidium floribundum3
Cymbidium floribundum4

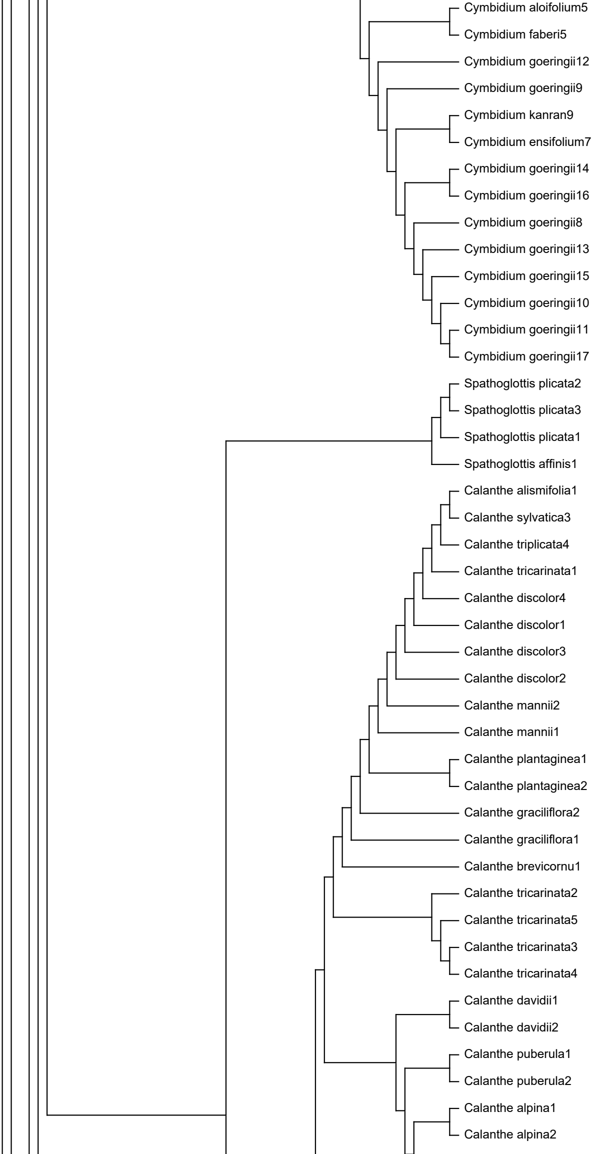
Cymbidium aloifolium5
Cymbidium faberi5
Cymbidium goeringii12
Cymbidium goeringii9
Cymbidium kanran9
Cymbidium ensifolium7
Cymbidium goeringii14
Cymbidium goeringii16
Cymbidium goeringii8
Cymbidium goeringii13
Cymbidium goeringii15
Cymbidium goeringii10
Cymbidium goeringii11
Cymbidium goeringii17
Spathoglottis plicata2
Spathoglottis plicata3
Spathoglottis plicata1
Spathoglottis affinis1
Calanthe alismifolia1
Calanthe sylvatica3
Calanthe triplicata4
Calanthe tricarinata1
Calanthe discolor4
Calanthe discolor1
Calanthe discolor3
Calanthe discolor2
Calanthe mannii2
Calanthe mannii1
Calanthe plantaginea1
Calanthe plantaginea2
Calanthe graciliflora2
Calanthe graciliflora1
Calanthe brevicornu1
Calanthe tricarinata2
Calanthe tricarinata5
Calanthe tricarinata3
Calanthe tricarinata4
Calanthe davidii1
Calanthe davidii2
Calanthe puberula1
Calanthe puberula2
Calanthe alpina1
Calanthe alpina2

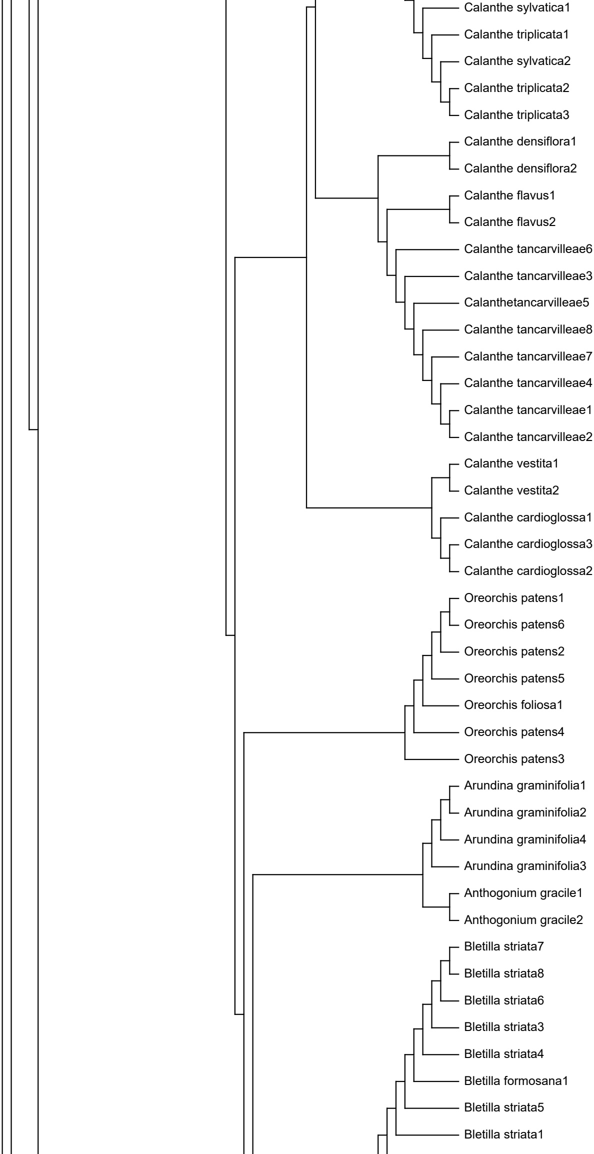
Calanthe sylvatica1
Calanthe triplicata1
Calanthe sylvatica2
Calanthe triplicata2
Calanthe triplicata3
Calanthe densiflora1
Calanthe densiflora2
Calanthe flavus1
Calanthe flavus2
Calanthe tancarvilleae6
Calanthe tancarvilleae3
Calanthetancarvilleae5
Calanthe tancarvilleae8
Calanthe tancarvilleae7
Calanthe tancarvilleae4
Calanthe tancarvilleae1
Calanthe tancarvilleae2
Calanthe vestita1
Calanthe vestita2
Calanthe cardioglossa1
Calanthe cardioglossa3
Calanthe cardioglossa2
Oreorchis patens1
Oreorchis patens6
Oreorchis patens2
Oreorchis patens5
Oreorchis foliosa1
Oreorchis patens4
Oreorchis patens3
Arundina graminifolia1
Arundina graminifolia2
Arundina graminifolia4
Arundina graminifolia3
Anthogonium gracile1
Anthogonium gracile2
Bletilla striata7
Bletilla striata8
Bletilla striata6
Bletilla striata3
Bletilla striata4
Bletilla formosana1
Bletilla striata5
Bletilla striata1

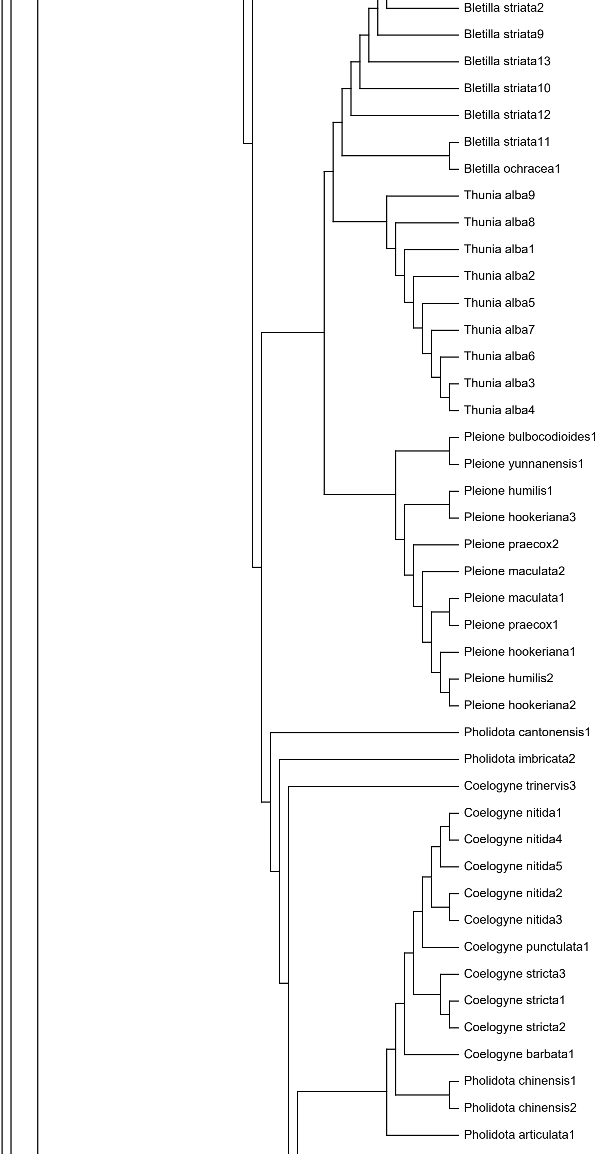
Bletilla striata2
Bletilla striata9
Bletilla striata13
Bletilla striata10
Bletilla striata12
Bletilla striata11
Bletilla ochracea1
Thunia alba9
Thunia alba8
Thunia alba1
Thunia alba2
Thunia alba5
Thunia alba7
Thunia alba6
Thunia alba3
Thunia alba4
Pleione bulbocodioides1
Pleione yunnanensis1
Pleione humilis1
Pleione hookeriana3
Pleione praecox2
Pleione maculata2
Pleione maculata1
Pleione praecox1
Pleione hookeriana1
Pleione humilis2
Pleione hookeriana2
Pholidota cantonensis1
Pholidota imbricata2
Coelogyne trinervis3
Coelogyne nitida1
Coelogyne nitida4
Coelogyne nitida5
Coelogyne nitida2
Coelogyne nitida3
Coelogyne punctulata1
Coelogyne stricta3
Coelogyne stricta1
Coelogyne stricta2
Coelogyne barbata1
Pholidota chinensis1
Pholidota chinensis2
Pholidota articulata1

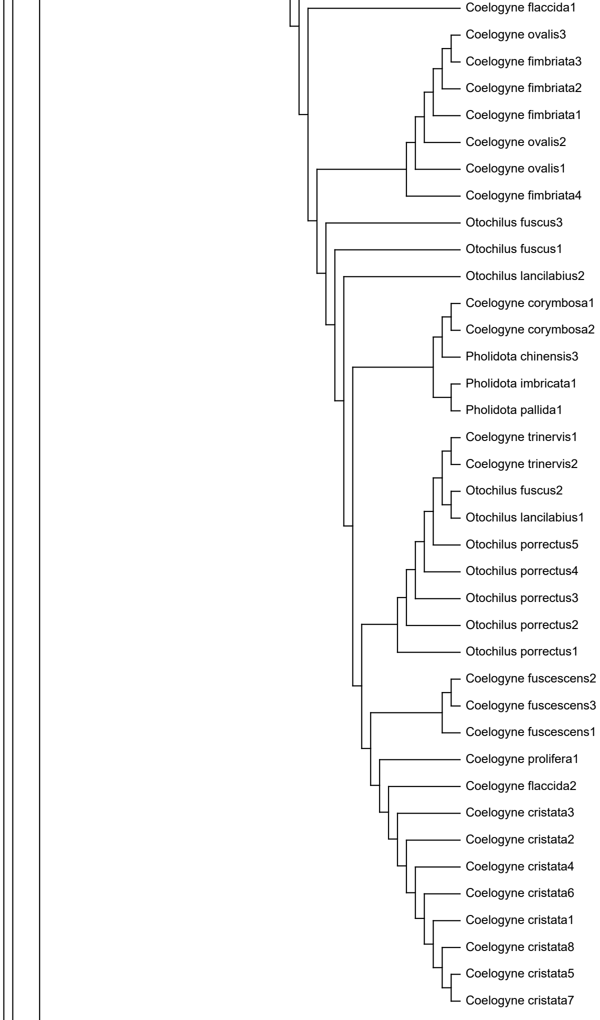

Coelogyne flaccida1
Coelogyne ovalis3
Coelogyne fimbriata3
Coelogyne fimbriata2
Coelogyne fimbriata1
Coelogyne ovalis2
Coelogyne ovalis1
Coelogyne fimbriata4
Otochilus fuscus3
Otochilus fuscus1
Otochilus lancilabius2
Coelogyne corymbosa1
Coelogyne corymbosa2
Pholidota chinensis3
Pholidota imbricata1
Pholidota pallida1
Coelogyne trinervis1
Coelogyne trinervis2
Otochilus fuscus2
Otochilus lancilabius1
Otochilus porrectus5
Otochilus porrectus4
Otochilus porrectus3
Otochilus porrectus2
Otochilus porrectus1
Coelogyne fuscescens2
Coelogyne fuscescens3
Coelogyne fuscescens1
Coelogyne prolifera1
Coelogyne flaccida2
Coelogyne cristata3
Coelogyne cristata2
Coelogyne cristata4
Coelogyne cristata6
Coelogyne cristata1
Coelogyne cristata8
Coelogyne cristata5
Coelogyne cristata7

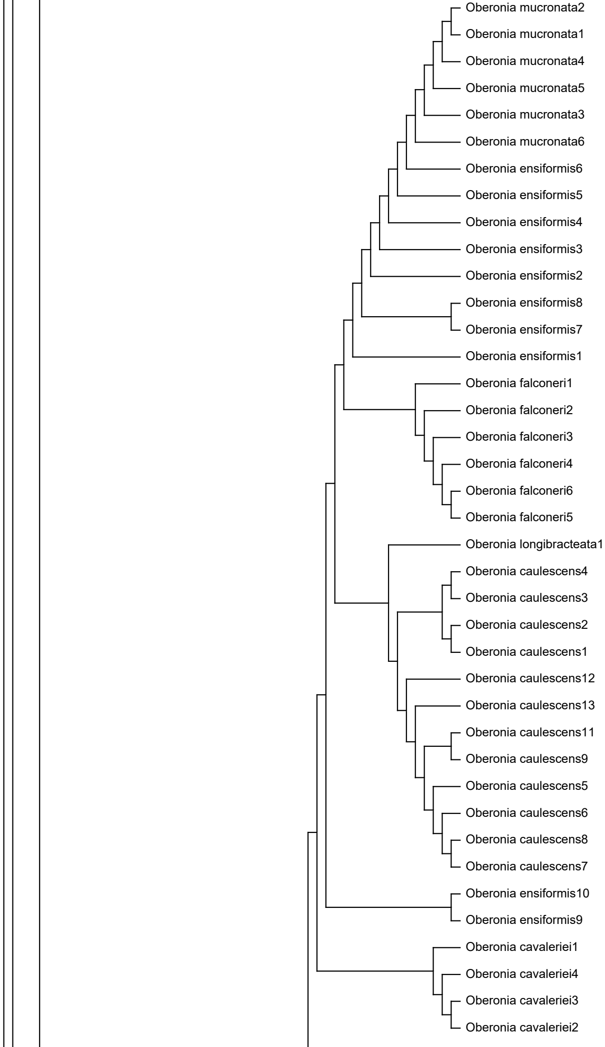
Oberonia mucronata2
Oberonia mucronata1
Oberonia mucronata4
Oberonia mucronata5
Oberonia mucronata3
Oberonia mucronata6
Oberonia ensiformis6
Oberonia ensiformis5
Oberonia ensiformis4
Oberonia ensiformis3
Oberonia ensiformis2
Oberonia ensiformis8
Oberonia ensiformis7
Oberonia ensiformis1
Oberonia falconeri1
Oberonia falconeri2
Oberonia falconeri3
Oberonia falconeri4
Oberonia falconeri6
Oberonia falconeri5
Oberonia longibracteata1
Oberonia caulescens4
Oberonia caulescens3
Oberonia caulescens2
Oberonia caulescens1
Oberonia caulescens12
Oberonia caulescens13
Oberonia caulescens11
Oberonia caulescens9
Oberonia caulescens5
Oberonia caulescens6
Oberonia caulescens8
Oberonia caulescens7
Oberonia ensiformis10
Oberonia ensiformis9
Oberonia cavaleriei1
Oberonia cavaleriei4
Oberonia cavaleriei3
Oberonia cavaleriei2

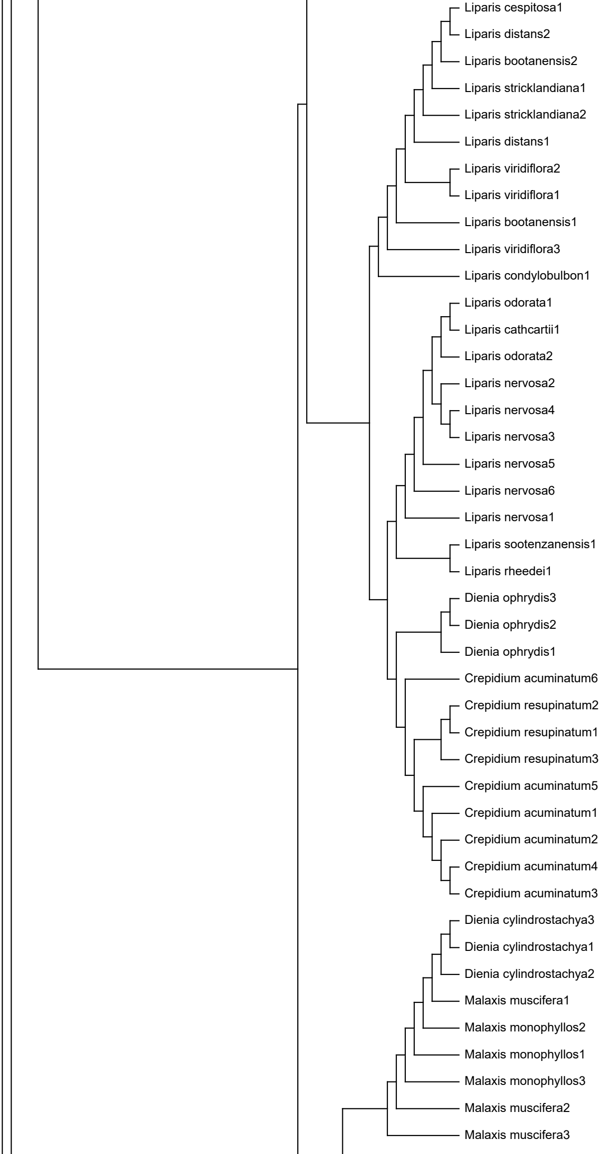
Liparis cespitosa1
Liparis distans2
Liparis bootanensis2
Liparis stricklandiana1
Liparis stricklandiana2
Liparis distans1
Liparis viridiflora2
Liparis viridiflora1
Liparis bootanensis1
Liparis viridiflora3
Liparis condylobulbon1
Liparis odorata1
Liparis cathcartii1
Liparis odorata2
Liparis nervosa2
Liparis nervosa4
Liparis nervosa3
Liparis nervosa5
Liparis nervosa6
Liparis nervosa1
Liparis sootenzanensis1
Liparis rheedei1
Dienia ophrydis3
Dienia ophrydis2
Dienia ophrydis1
Crepidium acuminatum6
Crepidium resupinatum2
Crepidium resupinatum1
Crepidium resupinatum3
Crepidium acuminatum5
Crepidium acuminatum1
Crepidium acuminatum2
Crepidium acuminatum4
Crepidium acuminatum3
Dienia cylindrostachya3
Dienia cylindrostachya1
Dienia cylindrostachya2
Malaxis muscifera1
Malaxis monophyllos2
Malaxis monophyllos1
Malaxis monophyllos3
Malaxis muscifera2
Malaxis muscifera3

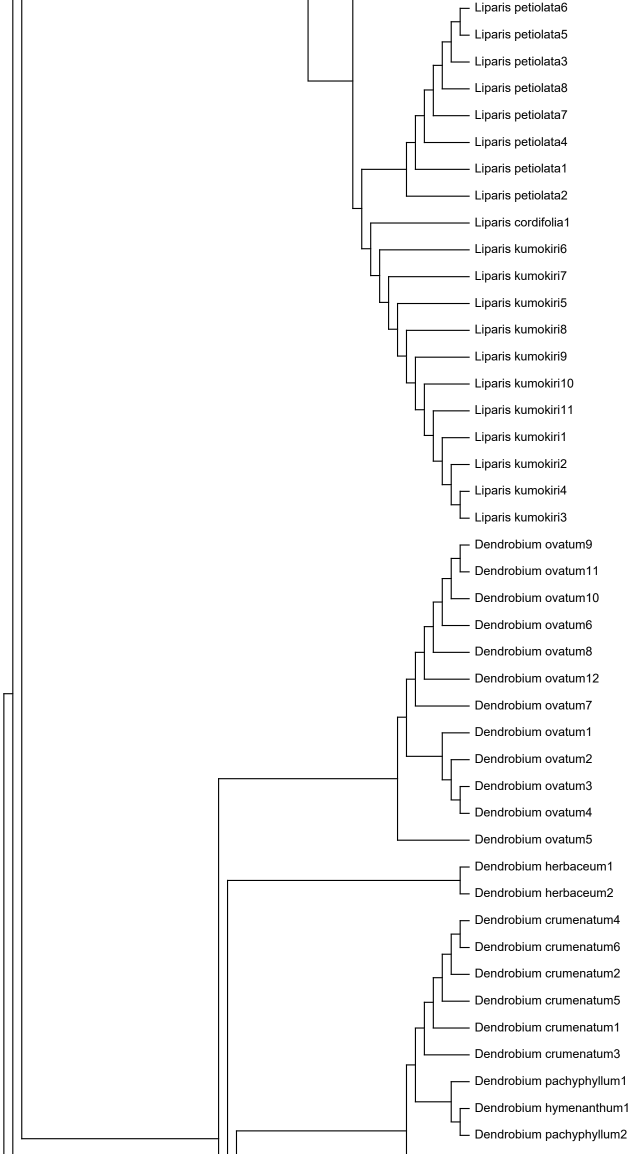
Liparis petiolata6
Liparis petiolata5
Liparis petiolata3
Liparis petiolata8
Liparis petiolata7
Liparis petiolata4
Liparis petiolata1
Liparis petiolata2
Liparis cordifolia1
Liparis kumokiri6
Liparis kumokiri7
Liparis kumokiri5
Liparis kumokiri8
Liparis kumokiri9
Liparis kumokiri10
Liparis kumokiri11
Liparis kumokiri1
Liparis kumokiri2
Liparis kumokiri4
Liparis kumokiri3
Dendrobium ovatum9
Dendrobium ovatum11
Dendrobium ovatum10
Dendrobium ovatum6
Dendrobium ovatum8
Dendrobium ovatum12
Dendrobium ovatum7
Dendrobium ovatum1
Dendrobium ovatum2
Dendrobium ovatum3
Dendrobium ovatum4
Dendrobium ovatum5
Dendrobium herbaceum1
Dendrobium herbaceum2
Dendrobium crumenatum4
Dendrobium crumenatum6
Dendrobium crumenatum2
Dendrobium crumenatum5
Dendrobium crumenatum1
Dendrobium crumenatum3
Dendrobium pachyphyllum1
Dendrobium hymenanthum1
Dendrobium pachyphyllum2

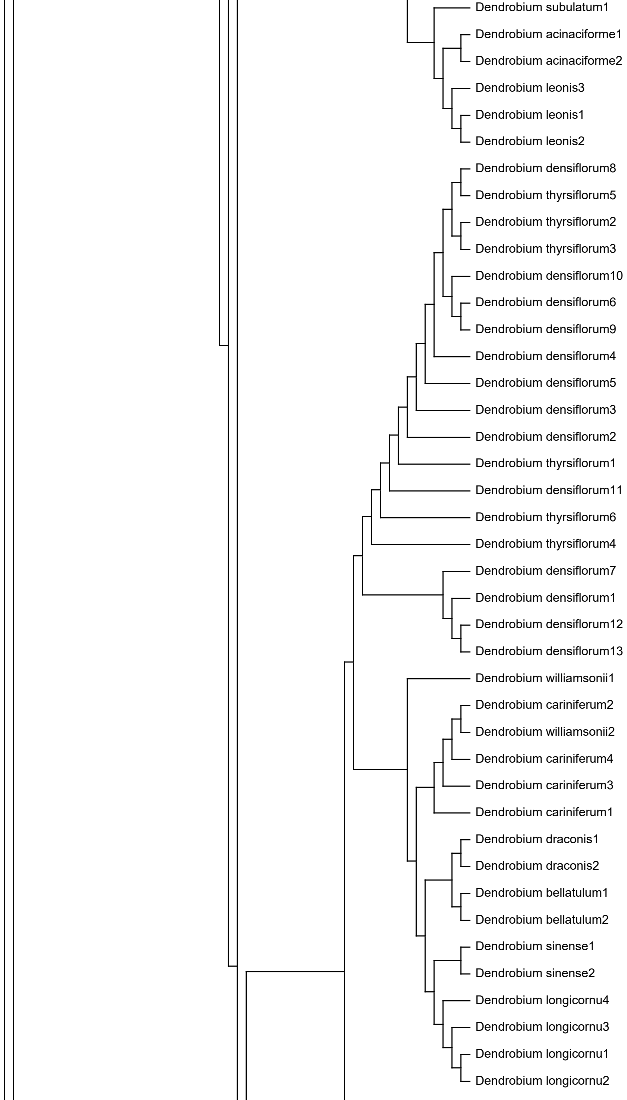

Dendrobium subulatum1
Dendrobium acinaciforme1
Dendrobium acinaciforme2
Dendrobium leonis3
Dendrobium leonis1
Dendrobium leonis2
Dendrobium densiflorum8
Dendrobium thyrsiflorum5
Dendrobium thyrsiflorum2
Dendrobium thyrsiflorum3
Dendrobium densiflorum10
Dendrobium densiflorum6
Dendrobium densiflorum9
Dendrobium densiflorum4
Dendrobium densiflorum5
Dendrobium densiflorum3
Dendrobium densiflorum2
Dendrobium thyrsiflorum1
Dendrobium densiflorum11
Dendrobium thyrsiflorum6
Dendrobium thyrsiflorum4
Dendrobium densiflorum7
Dendrobium densiflorum1
Dendrobium densiflorum12
Dendrobium densiflorum13
Dendrobium williamsonii1
Dendrobium cariniferum2
Dendrobium williamsonii2
Dendrobium cariniferum4
Dendrobium cariniferum3
Dendrobium cariniferum1
Dendrobium draconis1
Dendrobium draconis2
Dendrobium bellatulum1
Dendrobium bellatulum2
Dendrobium sinense1
Dendrobium sinense2
Dendrobium longicornu4
Dendrobium longicornu3
Dendrobium longicornu1
Dendrobium longicornu2

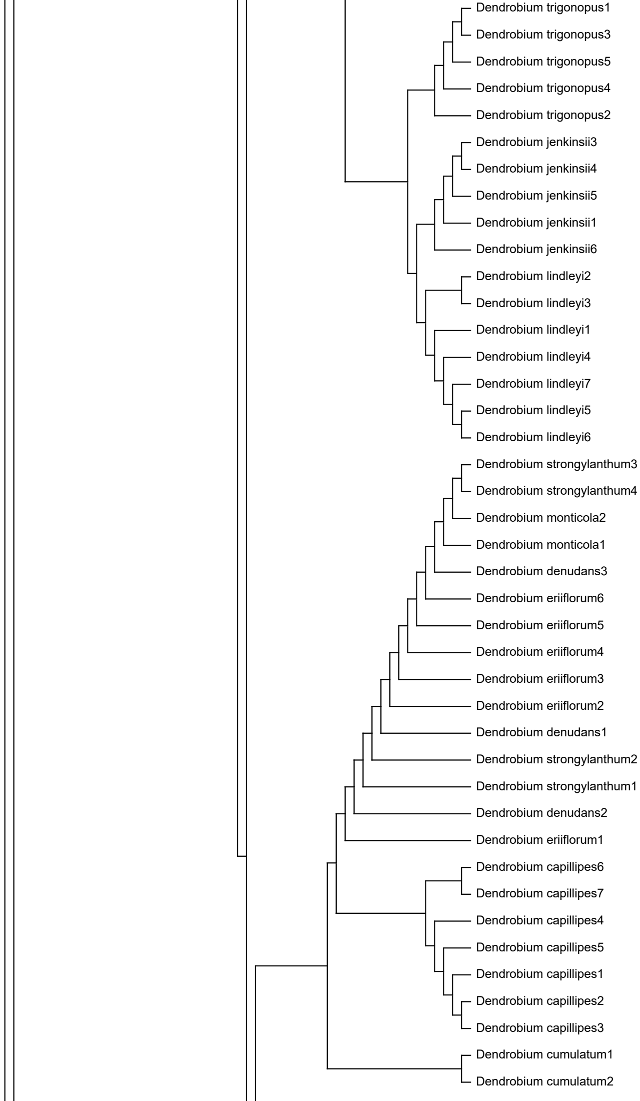
Dendrobium trigonopus1
Dendrobium trigonopus3
Dendrobium trigonopus5
Dendrobium trigonopus4
Dendrobium trigonopus2
Dendrobium jenkinsii3
Dendrobium jenkinsii4
Dendrobium jenkinsii5
Dendrobium jenkinsii1
Dendrobium jenkinsii6
Dendrobium lindleyi2
Dendrobium lindleyi3
Dendrobium lindleyi1
Dendrobium lindleyi4
Dendrobium lindleyi7
Dendrobium lindleyi5
Dendrobium lindleyi6
Dendrobium strongylanthum3
Dendrobium strongylanthum4
Dendrobium monticola2
Dendrobium monticola1
Dendrobium denudans3
Dendrobium eriiflorum6
Dendrobium eriiflorum5
Dendrobium eriiflorum4
Dendrobium eriiflorum3
Dendrobium eriiflorum2
Dendrobium denudans1
Dendrobium strongylanthum2
Dendrobium strongylanthum1
Dendrobium denudans2
Dendrobium eriiflorum1
Dendrobium capillipes6
Dendrobium capillipes7
Dendrobium capillipes4
Dendrobium capillipes5
Dendrobium capillipes1
Dendrobium capillipes2
Dendrobium capillipes3
Dendrobium cumulatum1
Dendrobium cumulatum2

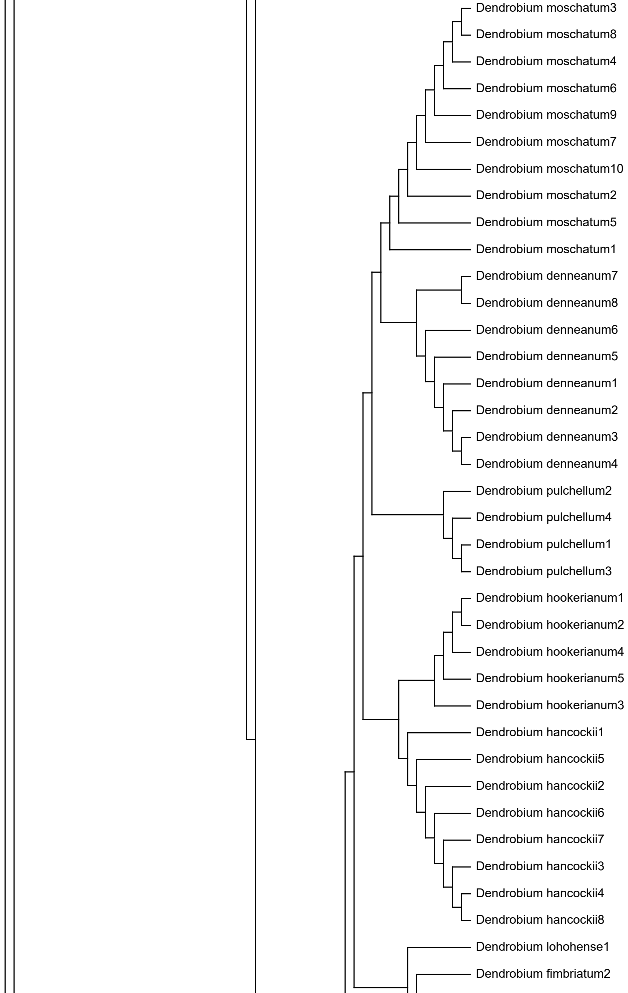
Dendrobium moschatum3
Dendrobium moschatum8
Dendrobium moschatum4
Dendrobium moschatum6
Dendrobium moschatum9
Dendrobium moschatum7
Dendrobium moschatum10
Dendrobium moschatum2
Dendrobium moschatum5
Dendrobium moschatum1
Dendrobium denneanum7
Dendrobium denneanum8
Dendrobium denneanum6
Dendrobium denneanum5
Dendrobium denneanum1
Dendrobium denneanum2
Dendrobium denneanum3
Dendrobium denneanum4
Dendrobium pulchellum2
Dendrobium pulchellum4
Dendrobium pulchellum1
Dendrobium pulchellum3
Dendrobium hookerianum1
Dendrobium hookerianum2
Dendrobium hookerianum4
Dendrobium hookerianum5
Dendrobium hookerianum3
Dendrobium hancockii1
Dendrobium hancockii5
Dendrobium hancockii2
Dendrobium hancockii6
Dendrobium hancockii7
Dendrobium hancockii3
Dendrobium hancockii4
Dendrobium hancockii8
Dendrobium lohohense1
Dendrobium fimbriatum2

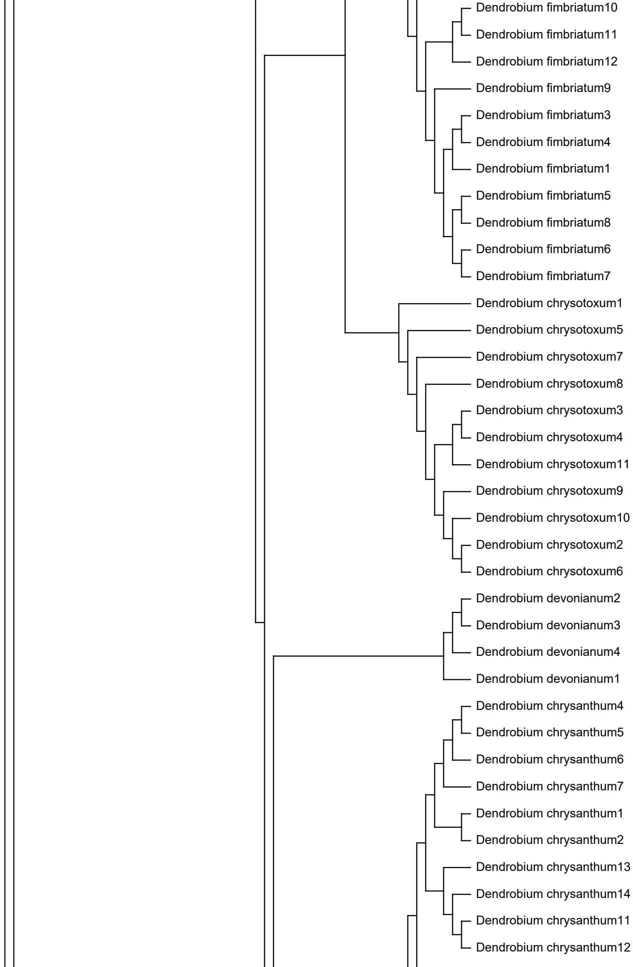
Dendrobium fimbriatum10
Dendrobium fimbriatum11
Dendrobium fimbriatum12
Dendrobium fimbriatum9
Dendrobium fimbriatum3
Dendrobium fimbriatum4
Dendrobium fimbriatum1
Dendrobium fimbriatum5
Dendrobium fimbriatum8
Dendrobium fimbriatum6
Dendrobium fimbriatum7
Dendrobium chrysotoxum1
Dendrobium chrysotoxum5
Dendrobium chrysotoxum7
Dendrobium chrysotoxum8
Dendrobium chrysotoxum3
Dendrobium chrysotoxum4
Dendrobium chrysotoxum11
Dendrobium chrysotoxum9
Dendrobium chrysotoxum10
Dendrobium chrysotoxum2
Dendrobium chrysotoxum6
Dendrobium devonianum2
Dendrobium devonianum3
Dendrobium devonianum4
Dendrobium devonianum1
Dendrobium chrysanthum4
Dendrobium chrysanthum5
Dendrobium chrysanthum6
Dendrobium chrysanthum7
Dendrobium chrysanthum1
Dendrobium chrysanthum2
Dendrobium chrysanthum13
Dendrobium chrysanthum14
Dendrobium chrysanthum11
Dendrobium chrysanthum12

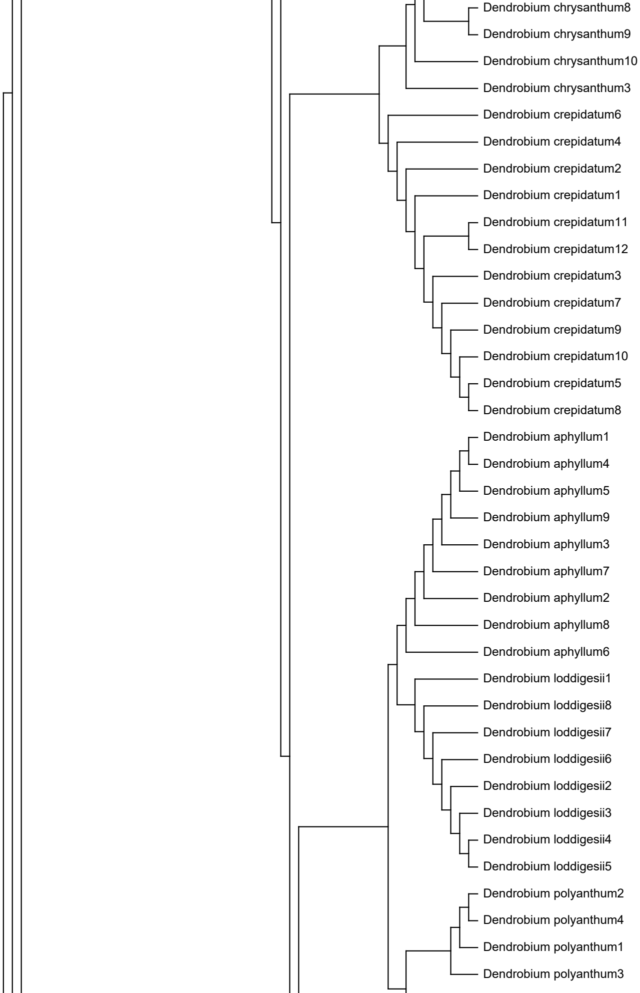
Dendrobium chrysanthum8
Dendrobium chrysanthum9
Dendrobium chrysanthum10
Dendrobium chrysanthum3
Dendrobium crepidatum6
Dendrobium crepidatum4
Dendrobium crepidatum2
Dendrobium crepidatum1
Dendrobium crepidatum11
Dendrobium crepidatum12
Dendrobium crepidatum3
Dendrobium crepidatum7
Dendrobium crepidatum9
Dendrobium crepidatum10
Dendrobium crepidatum5
Dendrobium crepidatum8
Dendrobium aphyllum1
Dendrobium aphyllum4
Dendrobium aphyllum5
Dendrobium aphyllum9
Dendrobium aphyllum3
Dendrobium aphyllum7
Dendrobium aphyllum2
Dendrobium aphyllum8
Dendrobium aphyllum6
Dendrobium loddigesii1
Dendrobium loddigesii8
Dendrobium loddigesii7
Dendrobium loddigesii6
Dendrobium loddigesii2
Dendrobium loddigesii3
Dendrobium loddigesii4
Dendrobium loddigesii5
Dendrobium polyanthum2
Dendrobium polyanthum4
Dendrobium polyanthum1
Dendrobium polyanthum3

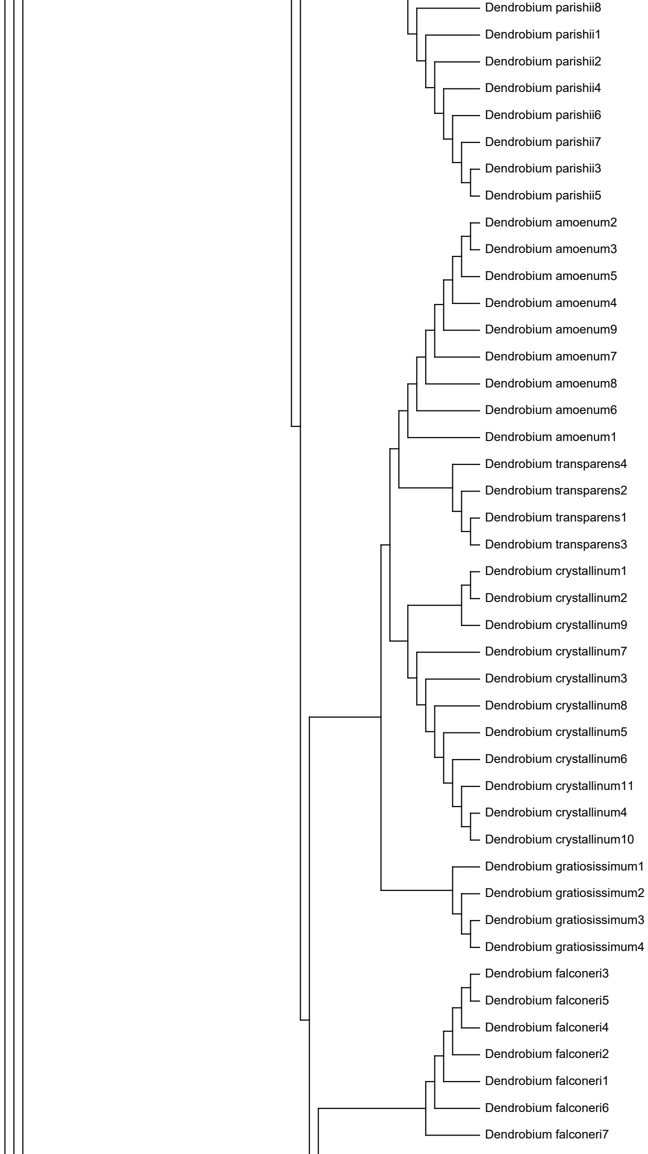
Dendrobium parishii8
Dendrobium parishii1
Dendrobium parishii2
Dendrobium parishii4
Dendrobium parishii6
Dendrobium parishii7
Dendrobium parishii3
Dendrobium parishii5
Dendrobium amoenum2
Dendrobium amoenum3
Dendrobium amoenum5
Dendrobium amoenum4
Dendrobium amoenum9
Dendrobium amoenum7
Dendrobium amoenum8
Dendrobium amoenum6
Dendrobium amoenum1
Dendrobium transparens4
Dendrobium transparens2
Dendrobium transparens1
Dendrobium transparens3
Dendrobium crystallinum1
Dendrobium crystallinum2
Dendrobium crystallinum9
Dendrobium crystallinum7
Dendrobium crystallinum3
Dendrobium crystallinum8
Dendrobium crystallinum5
Dendrobium crystallinum6
Dendrobium crystallinum11
Dendrobium crystallinum4
Dendrobium crystallinum10
Dendrobium gratiosissimum1
Dendrobium gratiosissimum2
Dendrobium gratiosissimum3
Dendrobium gratiosissimum4
Dendrobium falconeri3
Dendrobium falconeri5
Dendrobium falconeri4
Dendrobium falconeri2
Dendrobium falconeri1
Dendrobium falconeri6
Dendrobium falconeri7

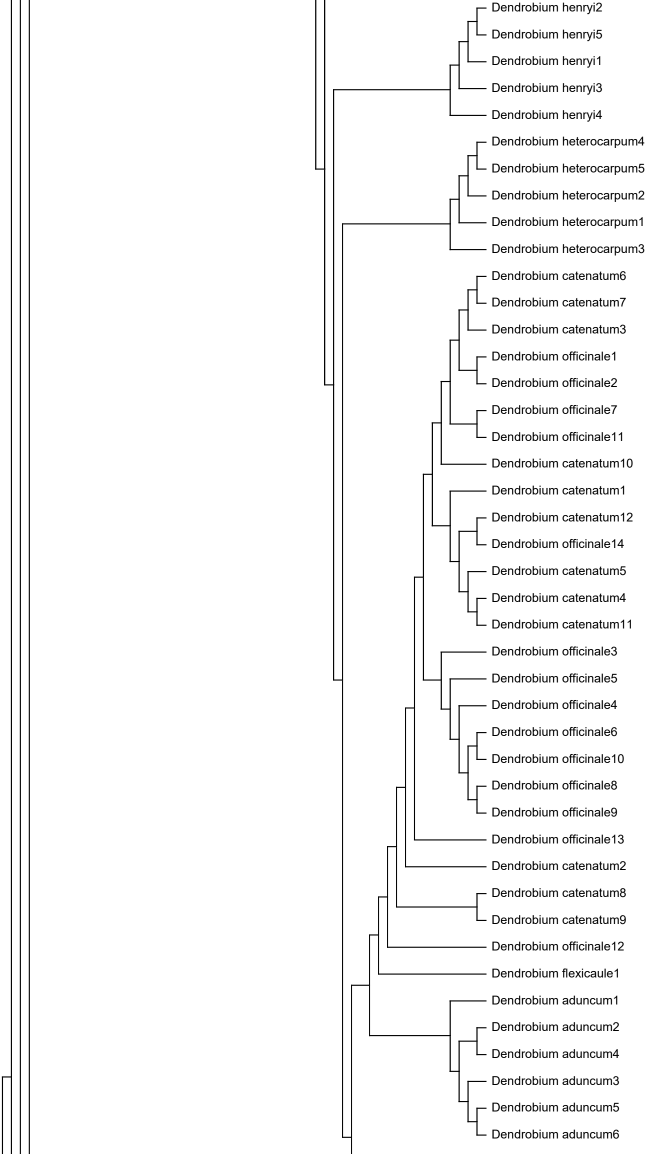
Dendrobium henryi2
Dendrobium henryi5
Dendrobium henryi1
Dendrobium henryi3
Dendrobium henryi4
Dendrobium heterocarpum4
Dendrobium heterocarpum5
Dendrobium heterocarpum2
Dendrobium heterocarpum1
Dendrobium heterocarpum3
Dendrobium catenatum6
Dendrobium catenatum7
Dendrobium catenatum3
Dendrobium officinale1
Dendrobium officinale2
Dendrobium officinale7
Dendrobium officinale11
Dendrobium catenatum10
Dendrobium catenatum1
Dendrobium catenatum12
Dendrobium officinale14
Dendrobium catenatum5
Dendrobium catenatum4
Dendrobium catenatum11
Dendrobium officinale3
Dendrobium officinale5
Dendrobium officinale4
Dendrobium officinale6
Dendrobium officinale10
Dendrobium officinale8
Dendrobium officinale9
Dendrobium officinale13
Dendrobium catenatum2
Dendrobium catenatum8
Dendrobium catenatum9
Dendrobium officinale12
Dendrobium flexicaule1
Dendrobium aduncum1
Dendrobium aduncum2
Dendrobium aduncum4
Dendrobium aduncum3
Dendrobium aduncum5
Dendrobium aduncum6

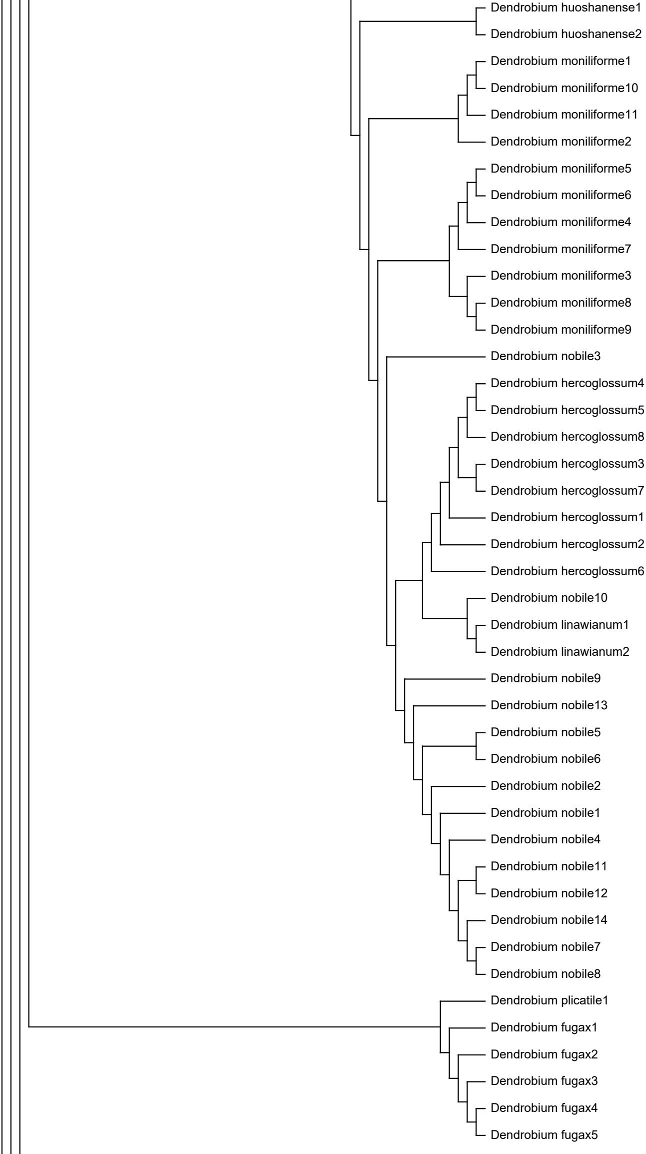

Dendrobium huoshanense1
Dendrobium huoshanense2
Dendrobium moniliforme1
Dendrobium moniliforme10
Dendrobium moniliforme11
Dendrobium moniliforme2
Dendrobium moniliforme5
Dendrobium moniliforme6
Dendrobium moniliforme4
Dendrobium moniliforme7
Dendrobium moniliforme3
Dendrobium moniliforme8
Dendrobium moniliforme9
Dendrobium nobile3
Dendrobium hercoglossum4
Dendrobium hercoglossum5
Dendrobium hercoglossum8
Dendrobium hercoglossum3
Dendrobium hercoglossum7
Dendrobium hercoglossum1
Dendrobium hercoglossum2
Dendrobium hercoglossum6
Dendrobium nobile10
Dendrobium linawianum1
Dendrobium linawianum2
Dendrobium nobile9
Dendrobium nobile13
Dendrobium nobile5
Dendrobium nobile6
Dendrobium nobile2
Dendrobium nobile1
Dendrobium nobile4
Dendrobium nobile11
Dendrobium nobile12
Dendrobium nobile14
Dendrobium nobile7
Dendrobium nobile8
Dendrobium plicatile1
Dendrobium fugax1
Dendrobium fugax2
Dendrobium fugax3
Dendrobium fugax4
Dendrobium fugax5

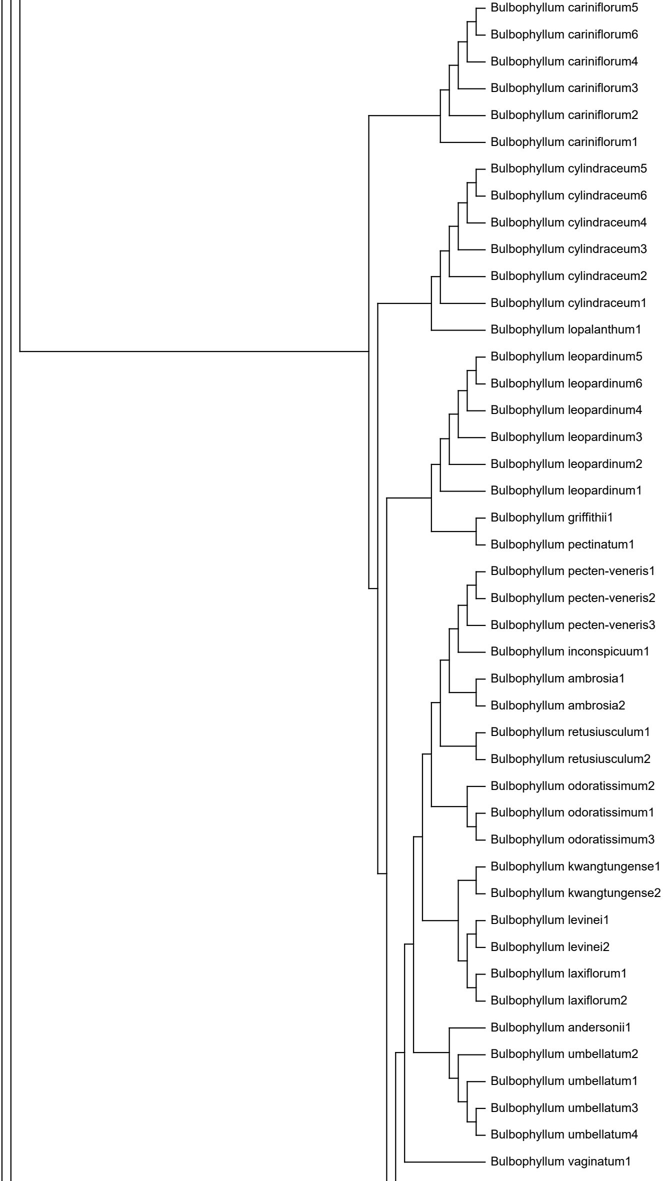
Bulbophyllum cariniflorum5
Bulbophyllum cariniflorum6
Bulbophyllum cariniflorum4
Bulbophyllum cariniflorum3
Bulbophyllum cariniflorum2
Bulbophyllum cariniflorum1
Bulbophyllum cylindraceum5
Bulbophyllum cylindraceum6
Bulbophyllum cylindraceum4
Bulbophyllum cylindraceum3
Bulbophyllum cylindraceum2
Bulbophyllum cylindraceum1
Bulbophyllum lopalanthum1
Bulbophyllum leopardinum5
Bulbophyllum leopardinum6
Bulbophyllum leopardinum4
Bulbophyllum leopardinum3
Bulbophyllum leopardinum2
Bulbophyllum leopardinum1
Bulbophyllum griffithii1
Bulbophyllum pectinatum1
Bulbophyllum pecten-veneris1
Bulbophyllum pecten-veneris2
Bulbophyllum pecten-veneris3
Bulbophyllum inconspicuum1
Bulbophyllum ambrosia1
Bulbophyllum ambrosia2
Bulbophyllum retusiusculum1
Bulbophyllum retusiusculum2
Bulbophyllum odoratissimum2
Bulbophyllum odoratissimum1
Bulbophyllum odoratissimum3
Bulbophyllum kwangtungense1
Bulbophyllum kwangtungense2
Bulbophyllum levinei1
Bulbophyllum levinei2
Bulbophyllum laxiflorum1
Bulbophyllum laxiflorum2
Bulbophyllum andersonii1
Bulbophyllum umbellatum2
Bulbophyllum umbellatum1
Bulbophyllum umbellatum3
Bulbophyllum umbellatum4
Bulbophyllum vaginatum1

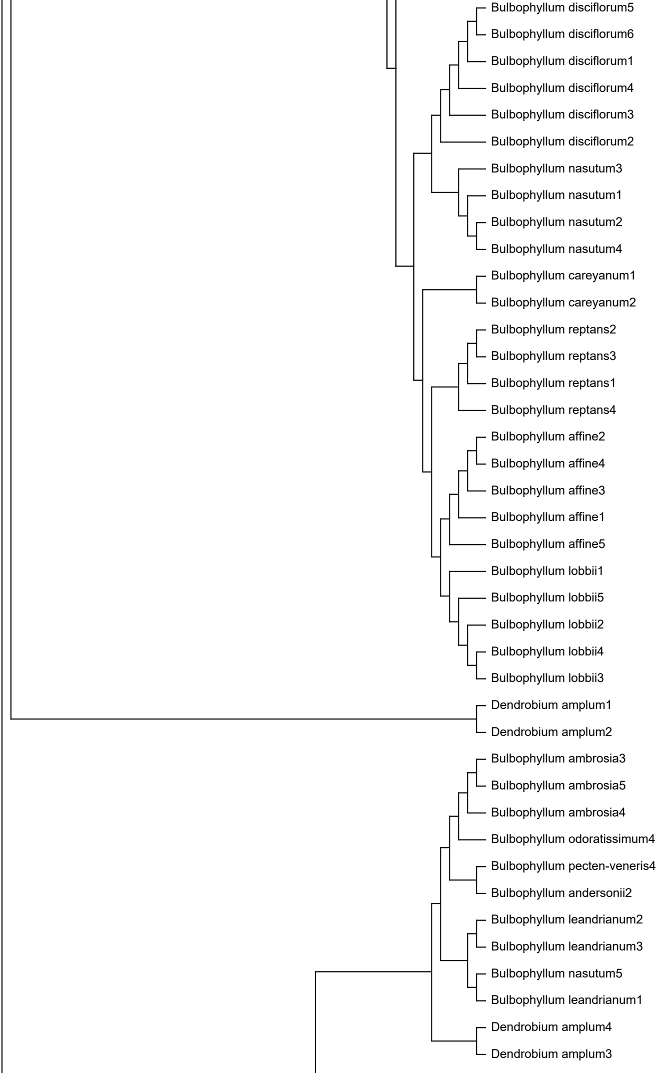
Bulbophyllum disciflorum5
Bulbophyllum disciflorum6
Bulbophyllum disciflorum1
Bulbophyllum disciflorum4
Bulbophyllum disciflorum3
Bulbophyllum disciflorum2
Bulbophyllum nasutum3
Bulbophyllum nasutum1
Bulbophyllum nasutum2
Bulbophyllum nasutum4
Bulbophyllum careyanum1
Bulbophyllum careyanum2
Bulbophyllum reptans2
Bulbophyllum reptans3
Bulbophyllum reptans1
Bulbophyllum reptans4
Bulbophyllum affine2
Bulbophyllum affine4
Bulbophyllum affine3
Bulbophyllum affine1
Bulbophyllum affine5
Bulbophyllum lobbii1
Bulbophyllum lobbii5
Bulbophyllum lobbii2
Bulbophyllum lobbii4
Bulbophyllum lobbii3
Dendrobium amplum1
Dendrobium amplum2
Bulbophyllum ambrosia3
Bulbophyllum ambrosia5
Bulbophyllum ambrosia4
Bulbophyllum odoratissimum4
Bulbophyllum pecten-veneris4
Bulbophyllum andersonii2
Bulbophyllum leandrianum2
Bulbophyllum leandrianum3
Bulbophyllum nasutum5
Bulbophyllum leandrianum1
Dendrobium amplum4
Dendrobium amplum3

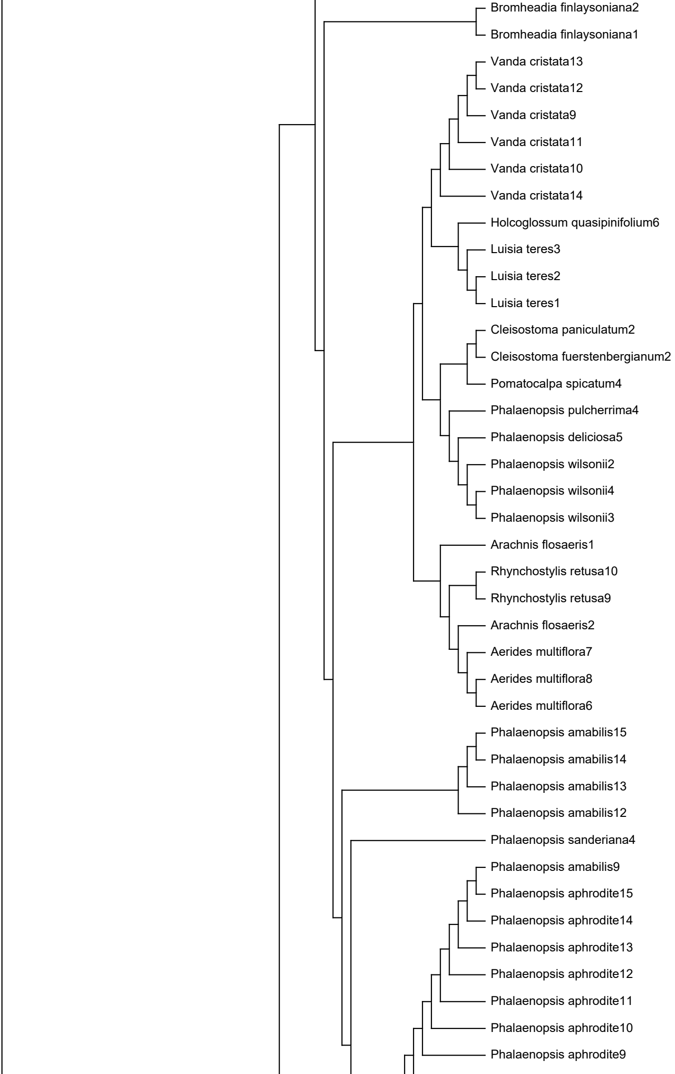

Bromheadia finlaysoniana2
Bromheadia finlaysoniana1
Vanda cristata13
Vanda cristata12
Vanda cristata9
Vanda cristata11
Vanda cristata10
Vanda cristata14
Holcoglossum quasipinifolium6
Luisia teres3
Luisia teres2
Luisia teres1
Cleisostoma paniculatum2
Cleisostoma fuerstenbergianum2
Pomatocalpa spicatum4
Phalaenopsis pulcherrima4
Phalaenopsis deliciosa5
Phalaenopsis wilsonii2
Phalaenopsis wilsonii4
Phalaenopsis wilsonii3
Arachnis flosaeris1
Rhynchostylis retusa10
Rhynchostylis retusa9
Arachnis flosaeris2
Aerides multiflora7
Aerides multiflora8
Aerides multiflora6
Phalaenopsis amabilis15
Phalaenopsis amabilis14
Phalaenopsis amabilis13
Phalaenopsis amabilis12
Phalaenopsis sanderiana4
Phalaenopsis amabilis9
Phalaenopsis aphrodite15
Phalaenopsis aphrodite14
Phalaenopsis aphrodite13
Phalaenopsis aphrodite12
Phalaenopsis aphrodite11
Phalaenopsis aphrodite10
Phalaenopsis aphrodite9

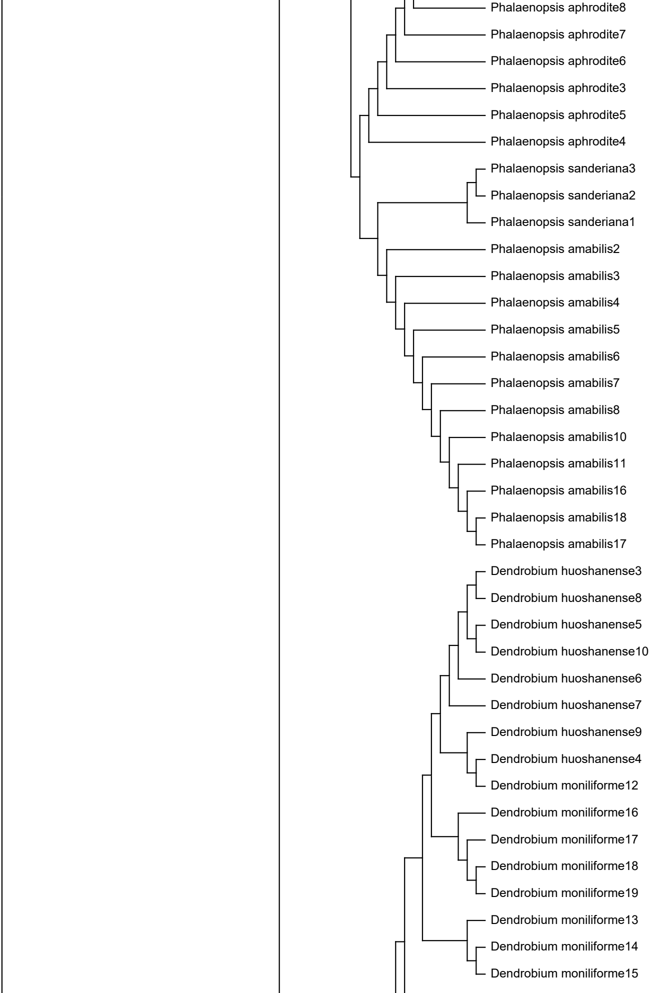
Phalaenopsis aphrodite8
Phalaenopsis aphrodite7
Phalaenopsis aphrodite6
Phalaenopsis aphrodite3
Phalaenopsis aphrodite5
Phalaenopsis aphrodite4
Phalaenopsis sanderiana3
Phalaenopsis sanderiana2
Phalaenopsis sanderiana1
Phalaenopsis amabilis2
Phalaenopsis amabilis3
Phalaenopsis amabilis4
Phalaenopsis amabilis5
Phalaenopsis amabilis6
Phalaenopsis amabilis7
Phalaenopsis amabilis8
Phalaenopsis amabilis10
Phalaenopsis amabilis11
Phalaenopsis amabilis16
Phalaenopsis amabilis18
Phalaenopsis amabilis17
Dendrobium huoshanense3
Dendrobium huoshanense8
Dendrobium huoshanense5
Dendrobium huoshanense10
Dendrobium huoshanense6
Dendrobium huoshanense7
Dendrobium huoshanense9
Dendrobium huoshanense4
Dendrobium moniliforme12
Dendrobium moniliforme16
Dendrobium moniliforme17
Dendrobium moniliforme18
Dendrobium moniliforme19
Dendrobium moniliforme13
Dendrobium moniliforme14
Dendrobium moniliforme15

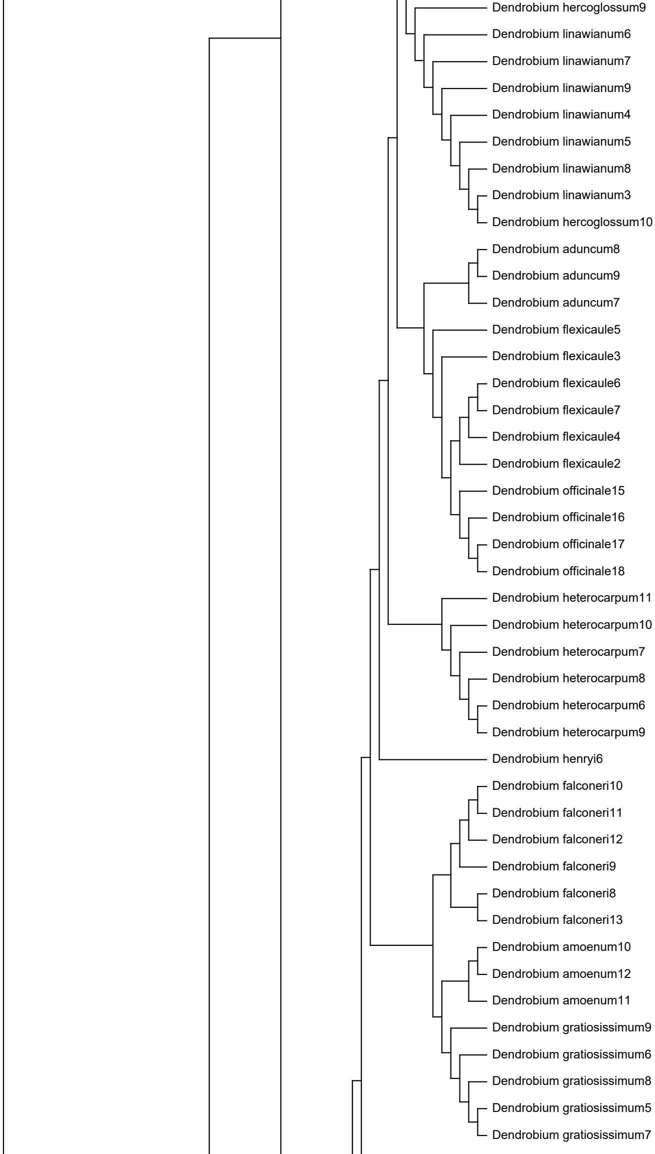
Dendrobium hercoglossum9
Dendrobium linawianum6
Dendrobium linawianum7
Dendrobium linawianum9
Dendrobium linawianum4
Dendrobium linawianum5
Dendrobium linawianum8
Dendrobium linawianum3
Dendrobium hercoglossum10
Dendrobium aduncum8
Dendrobium aduncum9
Dendrobium aduncum7
Dendrobium flexicaule5
Dendrobium flexicaule3
Dendrobium flexicaule6
Dendrobium flexicaule7
Dendrobium flexicaule4
Dendrobium flexicaule2
Dendrobium officinale15
Dendrobium officinale16
Dendrobium officinale17
Dendrobium officinale18
Dendrobium heterocarpum11
Dendrobium heterocarpum10
Dendrobium heterocarpum7
Dendrobium heterocarpum8
Dendrobium heterocarpum6
Dendrobium heterocarpum9
Dendrobium henryi6
Dendrobium falconeri10
Dendrobium falconeri11
Dendrobium falconeri12
Dendrobium falconeri9
Dendrobium falconeri8
Dendrobium falconeri13
Dendrobium amoenum10
Dendrobium amoenum12
Dendrobium amoenum11
Dendrobium gratiosissimum9
Dendrobium gratiosissimum6
Dendrobium gratiosissimum8
Dendrobium gratiosissimum5
Dendrobium gratiosissimum7

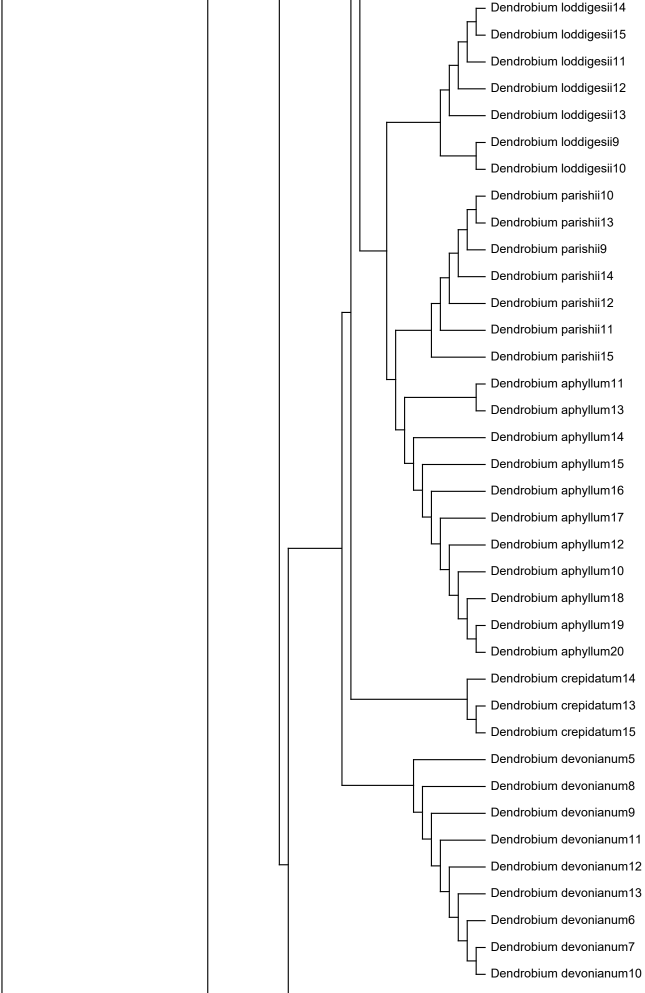

Dendrobium loddigesii14
Dendrobium loddigesii15
Dendrobium loddigesii11
Dendrobium loddigesii12
Dendrobium loddigesii13
Dendrobium loddigesii9
Dendrobium loddigesii10
Dendrobium parishii10
Dendrobium parishii13
Dendrobium parishii9
Dendrobium parishii14
Dendrobium parishii12
Dendrobium parishii11
Dendrobium parishii15
Dendrobium aphyllum11
Dendrobium aphyllum13
Dendrobium aphyllum14
Dendrobium aphyllum15
Dendrobium aphyllum16
Dendrobium aphyllum17
Dendrobium aphyllum12
Dendrobium aphyllum10
Dendrobium aphyllum18
Dendrobium aphyllum19
Dendrobium aphyllum20
Dendrobium crepidatum14
Dendrobium crepidatum13
Dendrobium crepidatum15
Dendrobium devonianum5
Dendrobium devonianum8
Dendrobium devonianum9
Dendrobium devonianum11
Dendrobium devonianum12
Dendrobium devonianum13
Dendrobium devonianum6
Dendrobium devonianum7
Dendrobium devonianum10

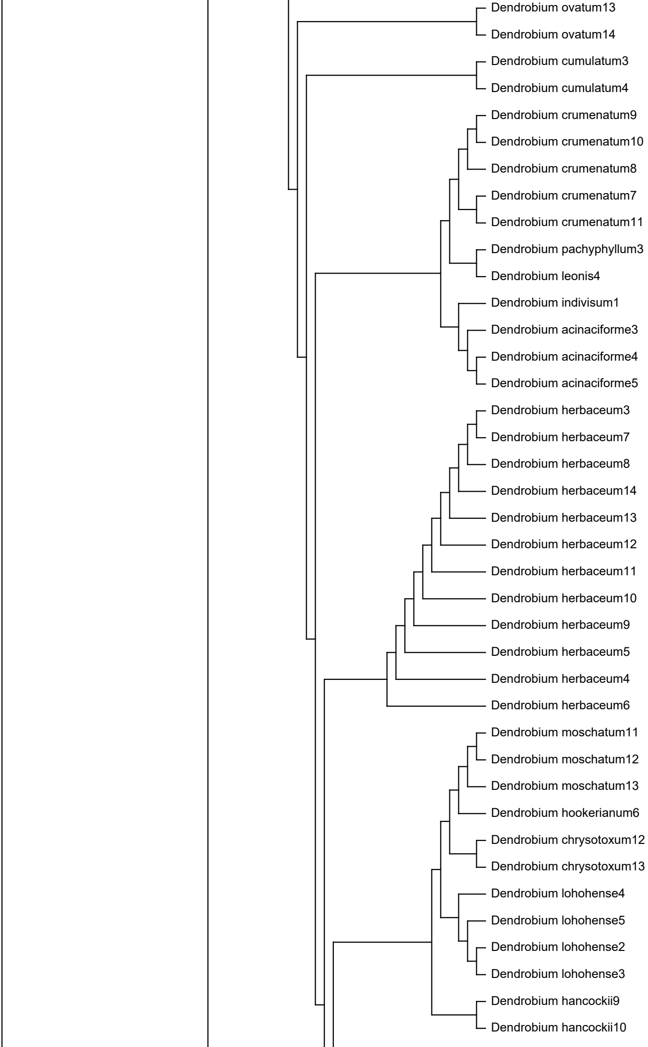
Dendrobium ovatum13
Dendrobium ovatum14
Dendrobium cumulatum3
Dendrobium cumulatum4
Dendrobium crumenatum9
Dendrobium crumenatum10
Dendrobium crumenatum8
Dendrobium crumenatum7
Dendrobium crumenatum11
Dendrobium pachyphyllum3
Dendrobium leonis4
Dendrobium indivisum1
Dendrobium acinaciforme3
Dendrobium acinaciforme4
Dendrobium acinaciforme5
Dendrobium herbaceum3
Dendrobium herbaceum7
Dendrobium herbaceum8
Dendrobium herbaceum14
Dendrobium herbaceum13
Dendrobium herbaceum12
Dendrobium herbaceum11
Dendrobium herbaceum10
Dendrobium herbaceum9
Dendrobium herbaceum5
Dendrobium herbaceum4
Dendrobium herbaceum6
Dendrobium moschatum11
Dendrobium moschatum12
Dendrobium moschatum13
Dendrobium hookerianum6
Dendrobium chrysotoxum12
Dendrobium chrysotoxum13
Dendrobium lohohense4
Dendrobium lohohense5
Dendrobium lohohense2
Dendrobium lohohense3
Dendrobium hancockii9
Dendrobium hancockii10

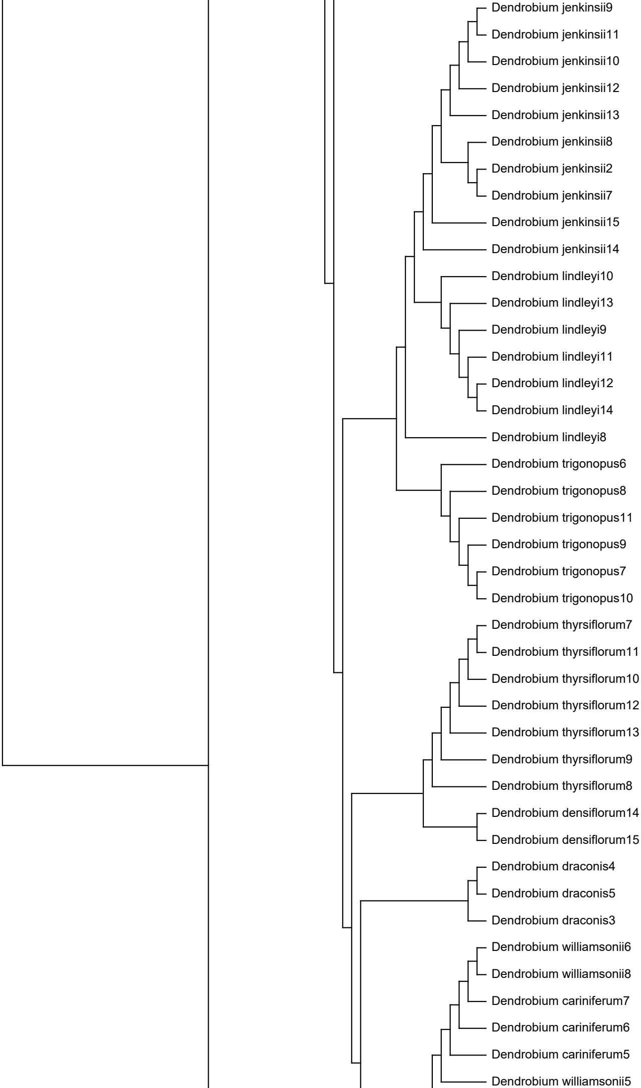

Dendrobium jenkinsii9
Dendrobium jenkinsii11
Dendrobium jenkinsii10
Dendrobium jenkinsii12
Dendrobium jenkinsii13
Dendrobium jenkinsii8
Dendrobium jenkinsii2
Dendrobium jenkinsii7
Dendrobium jenkinsii15
Dendrobium jenkinsii14
Dendrobium lindleyi10
Dendrobium lindleyi13
Dendrobium lindleyi9
Dendrobium lindleyi11
Dendrobium lindleyi12
Dendrobium lindleyi14
Dendrobium lindleyi8
Dendrobium trigonopus6
Dendrobium trigonopus8
Dendrobium trigonopus11
Dendrobium trigonopus9
Dendrobium trigonopus7
Dendrobium trigonopus10
Dendrobium thyrsiflorum7
Dendrobium thyrsiflorum11
Dendrobium thyrsiflorum10
Dendrobium thyrsiflorum12
Dendrobium thyrsiflorum13
Dendrobium thyrsiflorum9
Dendrobium thyrsiflorum8
Dendrobium densiflorum14
Dendrobium densiflorum15
Dendrobium draconis4
Dendrobium draconis5
Dendrobium draconis3
Dendrobium williamsonii6
Dendrobium williamsonii8
Dendrobium cariniferum7
Dendrobium cariniferum6
Dendrobium cariniferum5
Dendrobium williamsonii5

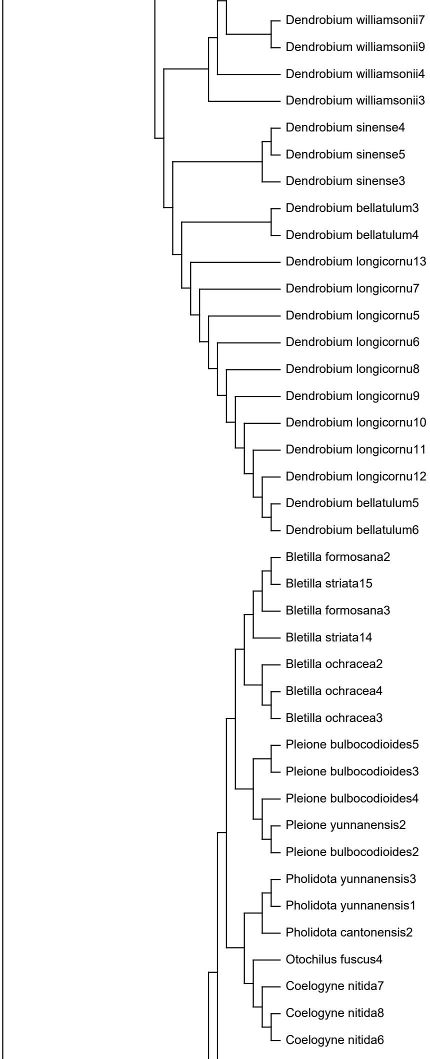
Dendrobium williamsonii7
Dendrobium williamsonii9
Dendrobium williamsonii4
Dendrobium williamsonii3
Dendrobium sinense4
Dendrobium sinense5
Dendrobium sinense3
Dendrobium bellatulum3
Dendrobium bellatulum4
Dendrobium longicornu13
Dendrobium longicornu7
Dendrobium longicornu5
Dendrobium longicornu6
Dendrobium longicornu8
Dendrobium longicornu9
Dendrobium longicornu10
Dendrobium longicornu11
Dendrobium longicornu12
Dendrobium bellatulum5
Dendrobium bellatulum6
Bletilla formosana2
Bletilla striata15
Bletilla formosana3
Bletilla striata14
Bletilla ochracea2
Bletilla ochracea4
Bletilla ochracea3
Pleione bulbocodioides5
Pleione bulbocodioides3
Pleione bulbocodioides4
Pleione yunnanensis2
Pleione bulbocodioides2
Pholidota yunnanensis3
Pholidota yunnanensis1
Pholidota cantonensis2
Otochilus fuscus4
Coelogyne nitida7
Coelogyne nitida8
Coelogyne nitida6

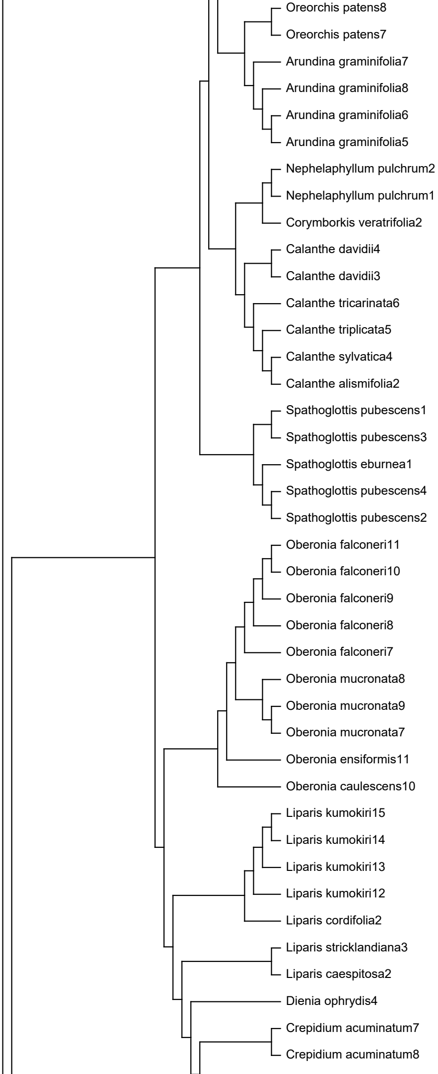
Oreorchis patens8
Oreorchis patens7
Arundina graminifolia7
Arundina graminifolia8
Arundina graminifolia6
Arundina graminifolia5
Nephelaphyllum pulchrum2
Nephelaphyllum pulchrum1
Corymborkis veratrifolia2
Calanthe davidii4
Calanthe davidii3
Calanthe tricarinata6
Calanthe triplicata5
Calanthe sylvatica4
Calanthe alismifolia2
Spathoglottis pubescens1
Spathoglottis pubescens3
Spathoglottis eburnea1
Spathoglottis pubescens4
Spathoglottis pubescens2
Oberonia falconeri11
Oberonia falconeri10
Oberonia falconeri9
Oberonia falconeri8
Oberonia falconeri7
Oberonia mucronata8
Oberonia mucronata9
Oberonia mucronata7
Oberonia ensiformis11
Oberonia caulescens10
Liparis kumokiri15
Liparis kumokiri14
Liparis kumokiri13
Liparis kumokiri12
Liparis cordifolia2
Liparis stricklandiana3
Liparis caespitosa2
Dienia ophrydis4
Crepidium acuminatum7
Crepidium acuminatum8

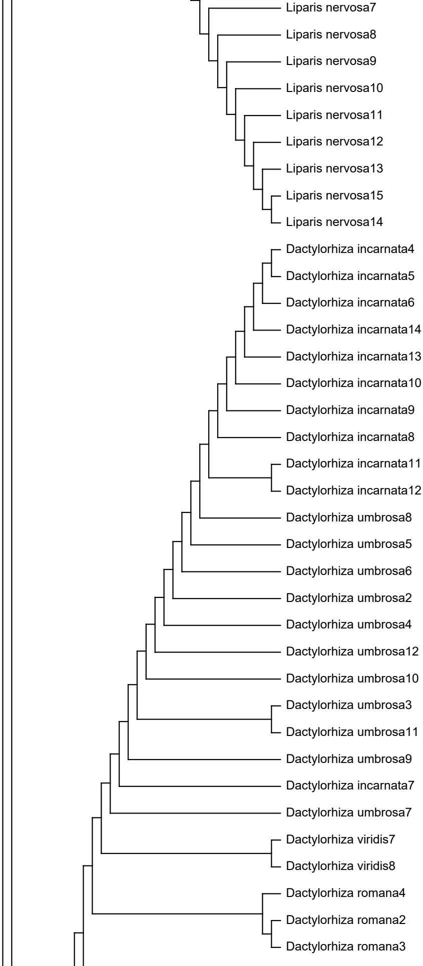
Liparis nervosa7
Liparis nervosa8
Liparis nervosa9
Liparis nervosa10
Liparis nervosa11
Liparis nervosa12
Liparis nervosa13
Liparis nervosa15
Liparis nervosa14
Dactylorhiza incarnata4
Dactylorhiza incarnata5
Dactylorhiza incarnata6
Dactylorhiza incarnata14
Dactylorhiza incarnata13
Dactylorhiza incarnata10
Dactylorhiza incarnata9
Dactylorhiza incarnata8
Dactylorhiza incarnata11
Dactylorhiza incarnata12
Dactylorhiza umbrosa8
Dactylorhiza umbrosa5
Dactylorhiza umbrosa6
Dactylorhiza umbrosa2
Dactylorhiza umbrosa4
Dactylorhiza umbrosa12
Dactylorhiza umbrosa10
Dactylorhiza umbrosa3
Dactylorhiza umbrosa11
Dactylorhiza umbrosa9
Dactylorhiza incarnata7
Dactylorhiza umbrosa7
Dactylorhiza viridis7
Dactylorhiza viridis8
Dactylorhiza romana4
Dactylorhiza romana2
Dactylorhiza romana3

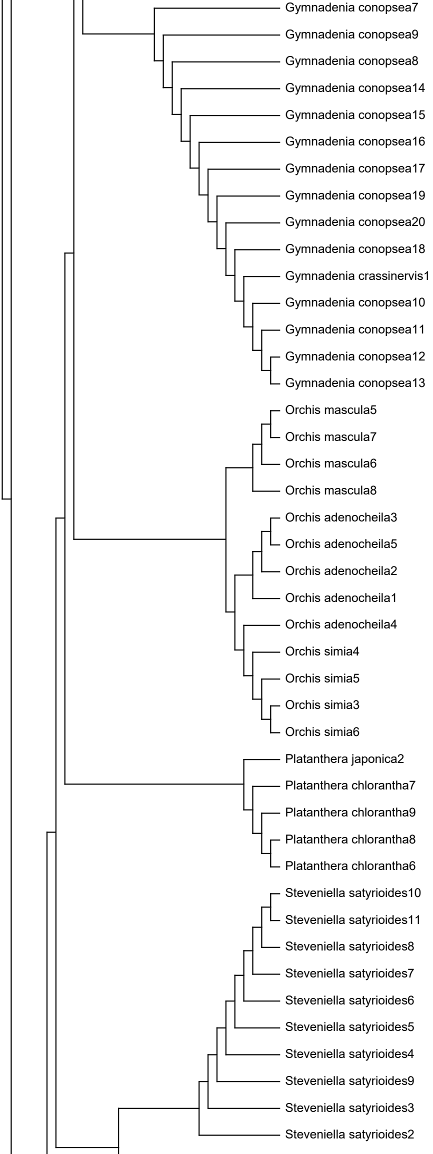
Gymnadenia conopsea7
Gymnadenia conopsea9
Gymnadenia conopsea8
Gymnadenia conopsea14
Gymnadenia conopsea15
Gymnadenia conopsea16
Gymnadenia conopsea17
Gymnadenia conopsea19
Gymnadenia conopsea20
Gymnadenia conopsea18
Gymnadenia crassinervis1
Gymnadenia conopsea10
Gymnadenia conopsea11
Gymnadenia conopsea12
Gymnadenia conopsea13
Orchis mascula5
Orchis mascula7
Orchis mascula6
Orchis mascula8
Orchis adenocheila3
Orchis adenocheila5
Orchis adenocheila2
Orchis adenocheila1
Orchis adenocheila4
Orchis simia4
Orchis simia5
Orchis simia3
Orchis simia6
Platanthera japonica2
Platanthera chlorantha7
Platanthera chlorantha9
Platanthera chlorantha8
Platanthera chlorantha6
Steveniella satyrioides10
Steveniella satyrioides11
Steveniella satyrioides8
Steveniella satyrioides7
Steveniella satyrioides6
Steveniella satyrioides5
Steveniella satyrioides4
Steveniella satyrioides9
Steveniella satyrioides3
Steveniella satyrioides2

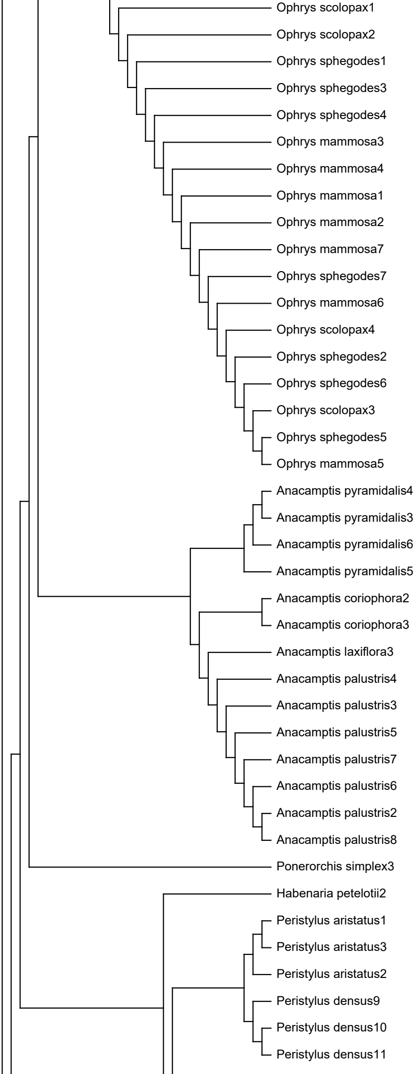
Ophrys scolopax1
Ophrys scolopax2
Ophrys sphegodes1
Ophrys sphegodes3
Ophrys sphegodes4
Ophrys mammosa3
Ophrys mammosa4
Ophrys mammosa1
Ophrys mammosa2
Ophrys mammosa7
Ophrys sphegodes7
Ophrys mammosa6
Ophrys scolopax4
Ophrys sphegodes2
Ophrys sphegodes6
Ophrys scolopax3
Ophrys sphegodes5
Ophrys mammosa5
Anacamptis pyramidalis4
Anacamptis pyramidalis3
Anacamptis pyramidalis6
Anacamptis pyramidalis5
Anacamptis coriophora2
Anacamptis coriophora3
Anacamptis laxiflora3
Anacamptis palustris4
Anacamptis palustris3
Anacamptis palustris5
Anacamptis palustris7
Anacamptis palustris6
Anacamptis palustris2
Anacamptis palustris8
Ponerorchis simplex3
Habenaria petelotii2
Peristylus aristatus1
Peristylus aristatus3
Peristylus aristatus2
Peristylus densus9
Peristylus densus10
Peristylus densus11

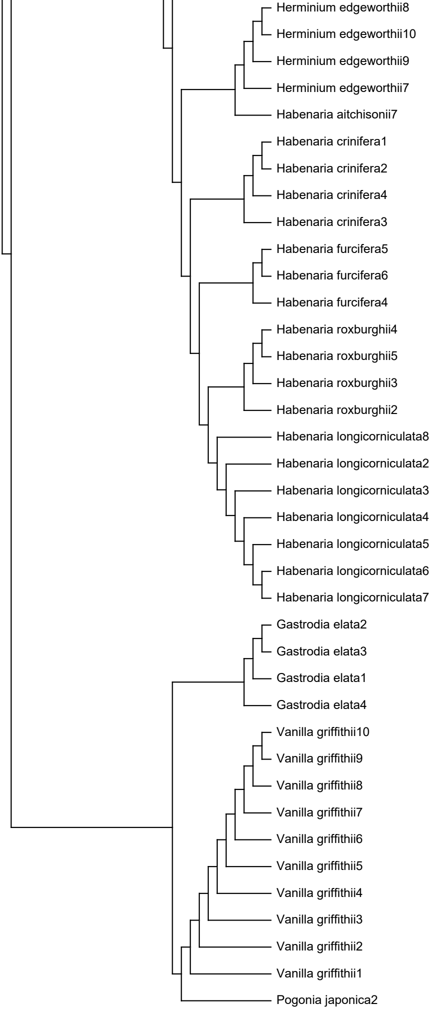
Herminium edgeworthii8
Herminium edgeworthii10
Herminium edgeworthii9
Herminium edgeworthii7
Habenaria aitchisonii7
Habenaria crinifera1
Habenaria crinifera2
Habenaria crinifera4
Habenaria crinifera3
Habenaria furcifera5
Habenaria furcifera6
Habenaria furcifera4
Habenaria roxburghii4
Habenaria roxburghii5
Habenaria roxburghii3
Habenaria roxburghii2
Habenaria longicorniculata8
Habenaria longicorniculata2
Habenaria longicorniculata3
Habenaria longicorniculata4
Habenaria longicorniculata5
Habenaria longicorniculata6
Habenaria longicorniculata7
Gastrodia elata2
Gastrodia elata3
Gastrodia elata1
Gastrodia elata4
Vanilla griffithii10
Vanilla griffithii9
Vanilla griffithii8
Vanilla griffithii7
Vanilla griffithii6
Vanilla griffithii5
Vanilla griffithii4
Vanilla griffithii3
Vanilla griffithii2
Vanilla griffithii1
Pogonia japonica2
