# Supplementary material for: DNA barcoding of medicinal orchids in Asia
Source: Sci Rep. 2021 Dec 8;11:23651. doi: 10.1038/s41598-021-03025-0 (PMC8654824; doi:10.1038/s41598-021-03025-0)
Supplement: Supplementary file 11 — Supplementary Figure S11. [file 41598_2021_3025_MOESM11_ESM.pdf]

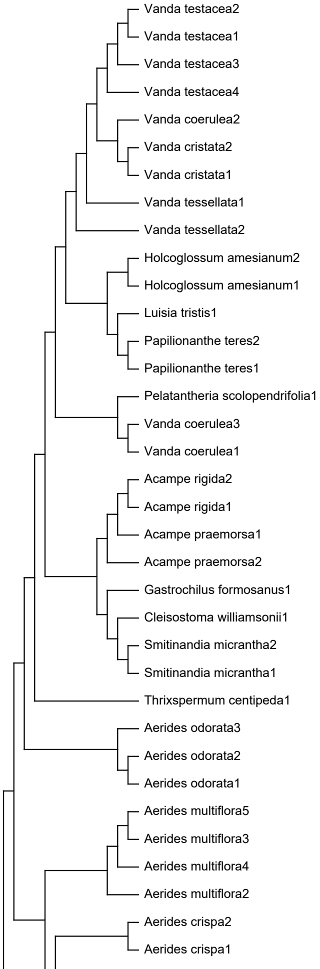
Vanda testacea2
Vanda testacea1
Vanda testacea3
Vanda testacea4
Vanda coerulea2
Vanda cristata2
Vanda cristata1
Vanda tessellata1
Vanda tessellata2
Holcoglossum amesianum2
Holcoglossum amesianum1
Luisia tristis1
Papilionanthe teres2
Papilionanthe teres1
Pelatantheria scolopendrifolia1
Vanda coerulea3
Vanda coerulea1
Acampe rigida2
Acampe rigida1
Acampe praemorsa1
Acampe praemorsa2
Gastrochilus formosanus1
Cleisostoma williamsonii1
Smitinandia micrantha2
Smitinandia micrantha1
Thrixspermum centipeda1
Aerides odorata3
Aerides odorata2
Aerides odorata1
Aerides multiflora5
Aerides multiflora3
Aerides multiflora4
Aerides multiflora2
Aerides crispa2
Aerides crispa1

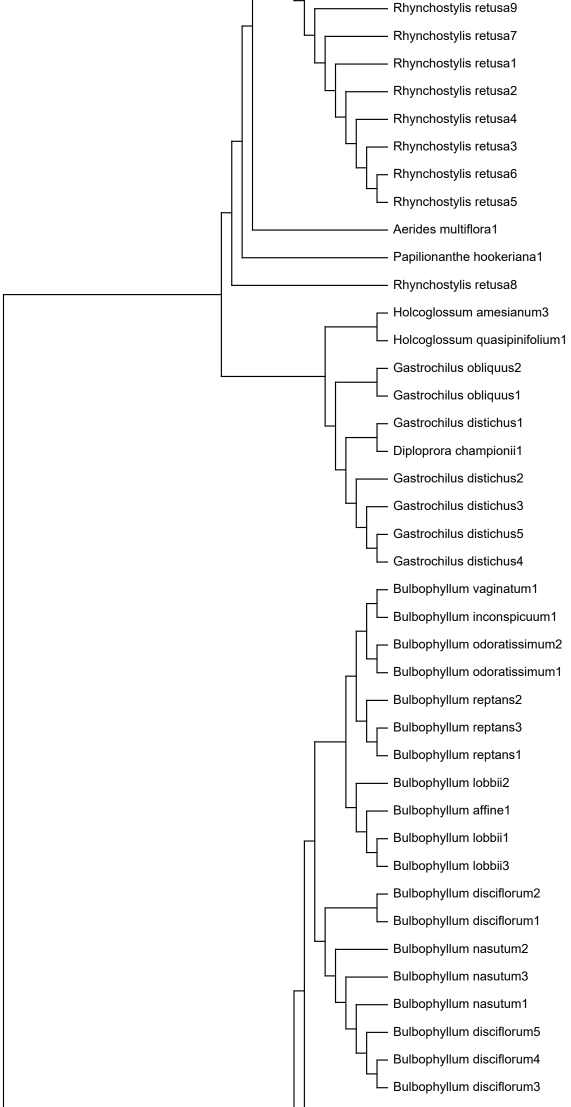
Rhynchostylis retusa9
Rhynchostylis retusa7
Rhynchostylis retusa1
Rhynchostylis retusa2
Rhynchostylis retusa4
Rhynchostylis retusa3
Rhynchostylis retusa6
Rhynchostylis retusa5
Aerides multiflora1
Papilionanthe hookeriana1
Rhynchostylis retusa8
Holcoglossum amesianum3
Holcoglossum quasipinifolium1
Gastrochilus obliquus2
Gastrochilus obliquus1
Gastrochilus distichus1
Diploprora championii1
Gastrochilus distichus2
Gastrochilus distichus3
Gastrochilus distichus5
Gastrochilus distichus4
Bulbophyllum vaginatum1
Bulbophyllum inconspicuum1
Bulbophyllum odoratissimum2
Bulbophyllum odoratissimum1
Bulbophyllum reptans2
Bulbophyllum reptans3
Bulbophyllum reptans1
Bulbophyllum lobbii2
Bulbophyllum affine1
Bulbophyllum lobbii1
Bulbophyllum lobbii3
Bulbophyllum disciflorum2
Bulbophyllum disciflorum1
Bulbophyllum nasutum2
Bulbophyllum nasutum3
Bulbophyllum nasutum1
Bulbophyllum disciflorum5
Bulbophyllum disciflorum4
Bulbophyllum disciflorum3

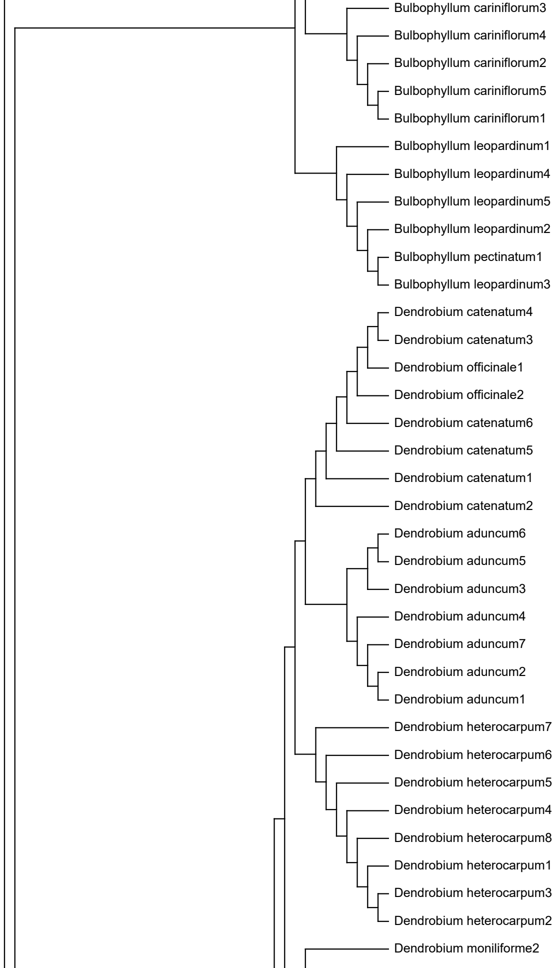
Bulbophyllum cariniflorum3
Bulbophyllum cariniflorum4
Bulbophyllum cariniflorum2
Bulbophyllum cariniflorum5
Bulbophyllum cariniflorum1
Bulbophyllum leopardinum1
Bulbophyllum leopardinum4
Bulbophyllum leopardinum5
Bulbophyllum leopardinum2
Bulbophyllum pectinatum1
Bulbophyllum leopardinum3
Dendrobium catenatum4
Dendrobium catenatum3
Dendrobium officinale1
Dendrobium officinale2
Dendrobium catenatum6
Dendrobium catenatum5
Dendrobium catenatum1
Dendrobium catenatum2
Dendrobium aduncum6
Dendrobium aduncum5
Dendrobium aduncum3
Dendrobium aduncum4
Dendrobium aduncum7
Dendrobium aduncum2
Dendrobium aduncum1
Dendrobium heterocarpum7
Dendrobium heterocarpum6
Dendrobium heterocarpum5
Dendrobium heterocarpum4
Dendrobium heterocarpum8
Dendrobium heterocarpum1
Dendrobium heterocarpum3
Dendrobium heterocarpum2
Dendrobium moniliforme2

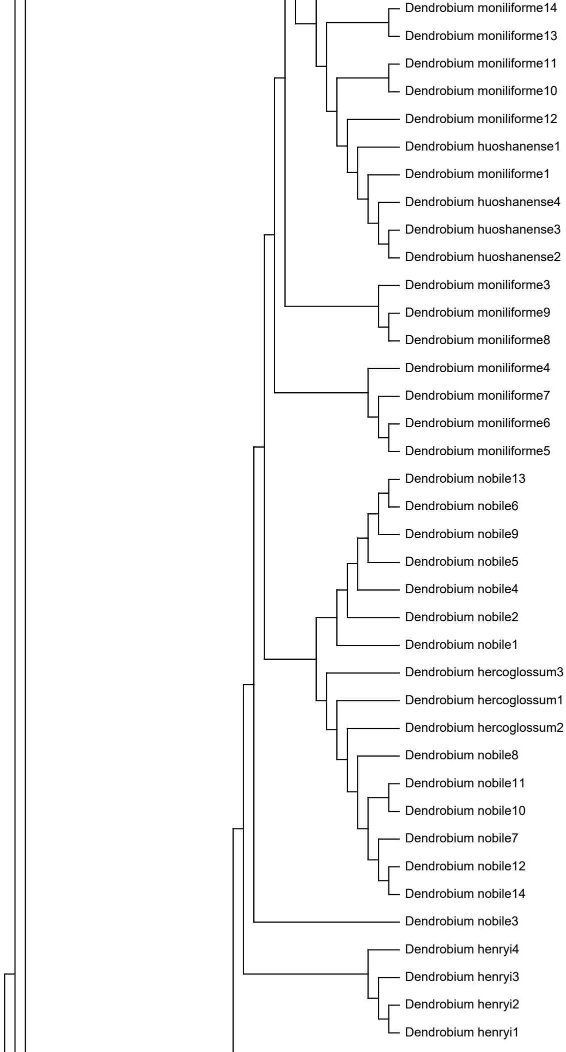
Dendrobium moniliforme14
Dendrobium moniliforme13
Dendrobium moniliforme11
Dendrobium moniliforme10
Dendrobium moniliforme12
Dendrobium huoshanense1
Dendrobium moniliforme1
Dendrobium huoshanense4
Dendrobium huoshanense3
Dendrobium huoshanense2
Dendrobium moniliforme3
Dendrobium moniliforme9
Dendrobium moniliforme8
Dendrobium moniliforme4
Dendrobium moniliforme7
Dendrobium moniliforme6
Dendrobium moniliforme5
Dendrobium nobile13
Dendrobium nobile6
Dendrobium nobile9
Dendrobium nobile5
Dendrobium nobile4
Dendrobium nobile2
Dendrobium nobile1
Dendrobium hercoglossum3
Dendrobium hercoglossum1
Dendrobium hercoglossum2
Dendrobium nobile8
Dendrobium nobile11
Dendrobium nobile10
Dendrobium nobile7
Dendrobium nobile12
Dendrobium nobile14
Dendrobium nobile3
Dendrobium henryi4
Dendrobium henryi3
Dendrobium henryi2
Dendrobium henryi1

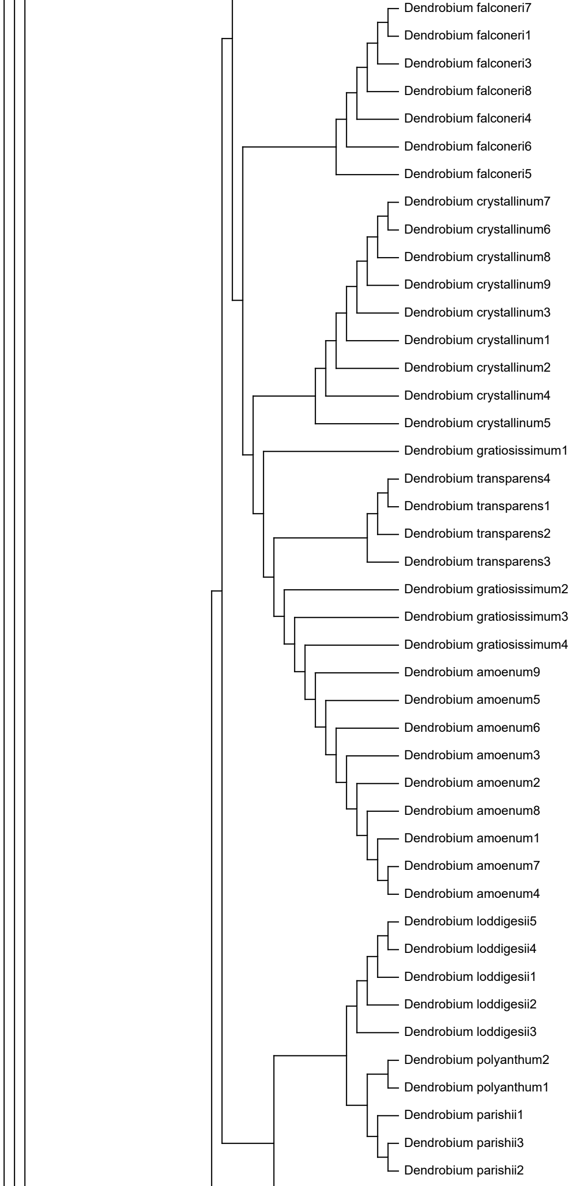
Dendrobium falconeri7
Dendrobium falconeri1
Dendrobium falconeri3
Dendrobium falconeri8
Dendrobium falconeri4
Dendrobium falconeri6
Dendrobium falconeri5
Dendrobium crystallinum7
Dendrobium crystallinum6
Dendrobium crystallinum8
Dendrobium crystallinum9
Dendrobium crystallinum3
Dendrobium crystallinum1
Dendrobium crystallinum2
Dendrobium crystallinum4
Dendrobium crystallinum5
Dendrobium gratiosissimum1
Dendrobium transparens4
Dendrobium transparens1
Dendrobium transparens2
Dendrobium transparens3
Dendrobium gratiosissimum2
Dendrobium gratiosissimum3
Dendrobium gratiosissimum4
Dendrobium amoenum9
Dendrobium amoenum5
Dendrobium amoenum6
Dendrobium amoenum3
Dendrobium amoenum2
Dendrobium amoenum8
Dendrobium amoenum1
Dendrobium amoenum7
Dendrobium amoenum4
Dendrobium loddigesii5
Dendrobium loddigesii4
Dendrobium loddigesii1
Dendrobium loddigesii2
Dendrobium loddigesii3
Dendrobium polyanthum2
Dendrobium polyanthum1
Dendrobium parishii1
Dendrobium parishii3
Dendrobium parishii2

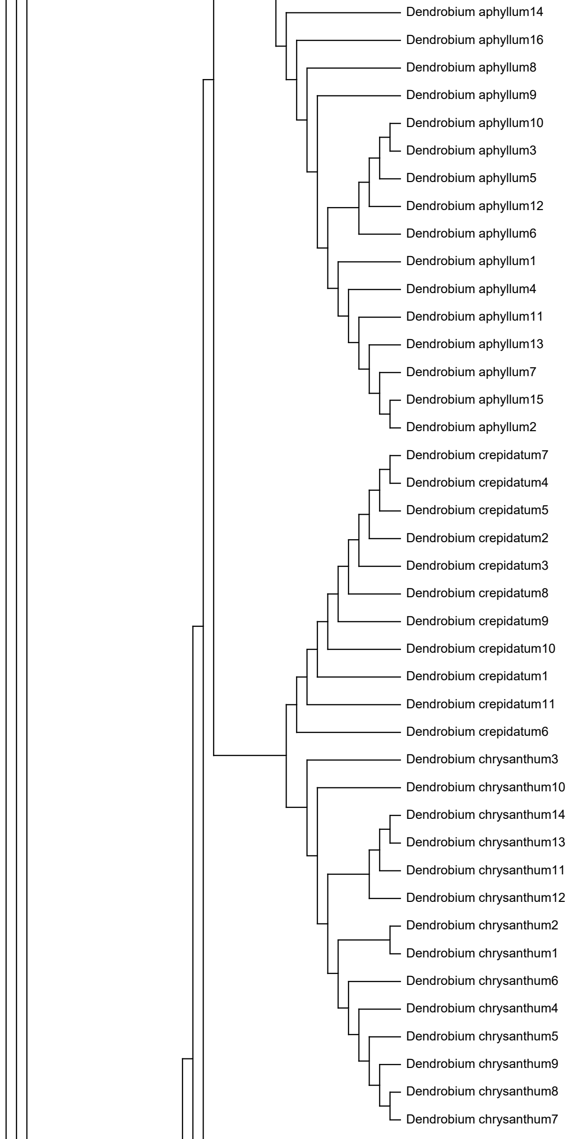
Dendrobium aphyllum14
Dendrobium aphyllum16
Dendrobium aphyllum8
Dendrobium aphyllum9
Dendrobium aphyllum10
Dendrobium aphyllum3
Dendrobium aphyllum5
Dendrobium aphyllum12
Dendrobium aphyllum6
Dendrobium aphyllum1
Dendrobium aphyllum4
Dendrobium aphyllum11
Dendrobium aphyllum13
Dendrobium aphyllum7
Dendrobium aphyllum15
Dendrobium aphyllum2
Dendrobium crepidatum7
Dendrobium crepidatum4
Dendrobium crepidatum5
Dendrobium crepidatum2
Dendrobium crepidatum3
Dendrobium crepidatum8
Dendrobium crepidatum9
Dendrobium crepidatum10
Dendrobium crepidatum1
Dendrobium crepidatum11
Dendrobium crepidatum6
Dendrobium chrysanthum3
Dendrobium chrysanthum10
Dendrobium chrysanthum14
Dendrobium chrysanthum13
Dendrobium chrysanthum11
Dendrobium chrysanthum12
Dendrobium chrysanthum2
Dendrobium chrysanthum1
Dendrobium chrysanthum6
Dendrobium chrysanthum4
Dendrobium chrysanthum5
Dendrobium chrysanthum9
Dendrobium chrysanthum8
Dendrobium chrysanthum7

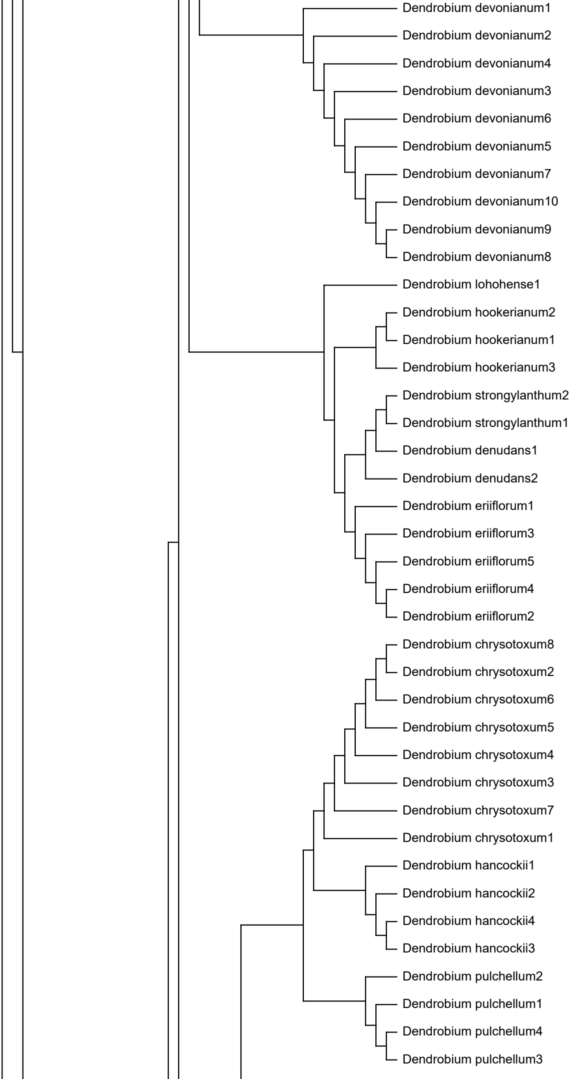
Dendrobium devonianum1
Dendrobium devonianum2
Dendrobium devonianum4
Dendrobium devonianum3
Dendrobium devonianum6
Dendrobium devonianum5
Dendrobium devonianum7
Dendrobium devonianum10
Dendrobium devonianum9
Dendrobium devonianum8
Dendrobium lohohense1
Dendrobium hookerianum2
Dendrobium hookerianum1
Dendrobium hookerianum3
Dendrobium strongylanthum2
Dendrobium strongylanthum1
Dendrobium denudans1
Dendrobium denudans2
Dendrobium eriiflorum1
Dendrobium eriiflorum3
Dendrobium eriiflorum5
Dendrobium eriiflorum4
Dendrobium eriiflorum2
Dendrobium chrysotoxum8
Dendrobium chrysotoxum2
Dendrobium chrysotoxum6
Dendrobium chrysotoxum5
Dendrobium chrysotoxum4
Dendrobium chrysotoxum3
Dendrobium chrysotoxum7
Dendrobium chrysotoxum1
Dendrobium hancockii1
Dendrobium hancockii2
Dendrobium hancockii4
Dendrobium hancockii3
Dendrobium pulchellum2
Dendrobium pulchellum1
Dendrobium pulchellum4
Dendrobium pulchellum3

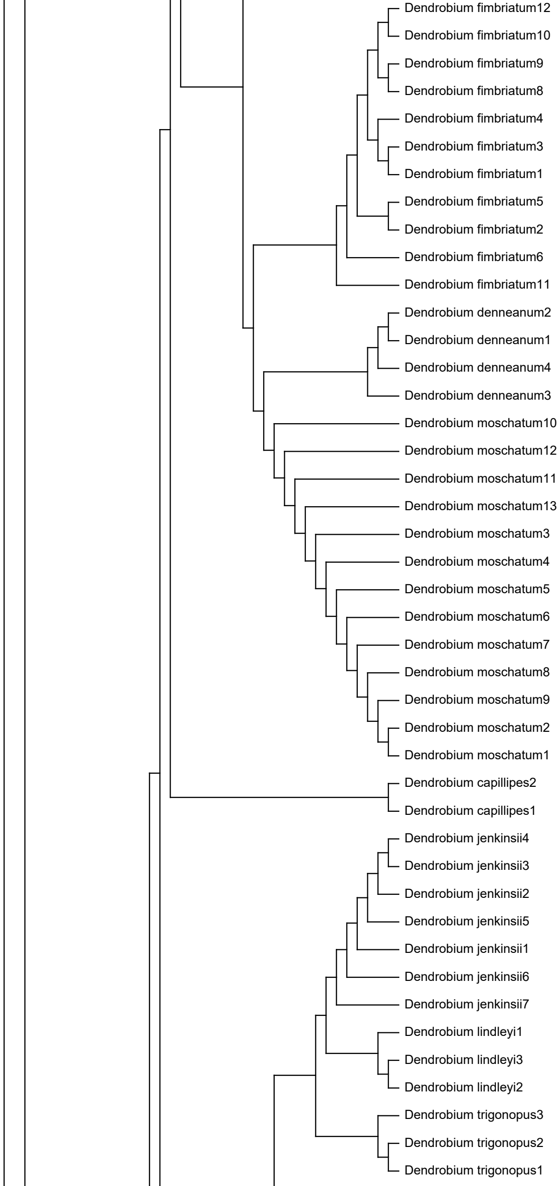
Dendrobium fimbriatum12
Dendrobium fimbriatum10
Dendrobium fimbriatum9
Dendrobium fimbriatum8
Dendrobium fimbriatum4
Dendrobium fimbriatum3
Dendrobium fimbriatum1
Dendrobium fimbriatum5
Dendrobium fimbriatum2
Dendrobium fimbriatum6
Dendrobium fimbriatum11
Dendrobium denneanum2
Dendrobium denneanum1
Dendrobium denneanum4
Dendrobium denneanum3
Dendrobium moschatum10
Dendrobium moschatum12
Dendrobium moschatum11
Dendrobium moschatum13
Dendrobium moschatum3
Dendrobium moschatum4
Dendrobium moschatum5
Dendrobium moschatum6
Dendrobium moschatum7
Dendrobium moschatum8
Dendrobium moschatum9
Dendrobium moschatum2
Dendrobium moschatum1
Dendrobium capillipes2
Dendrobium capillipes1
Dendrobium jenkinsii4
Dendrobium jenkinsii3
Dendrobium jenkinsii2
Dendrobium jenkinsii5
Dendrobium jenkinsii1
Dendrobium jenkinsii6
Dendrobium jenkinsii7
Dendrobium lindleyi1
Dendrobium lindleyi3
Dendrobium lindleyi2
Dendrobium trigonopus3
Dendrobium trigonopus2
Dendrobium trigonopus1

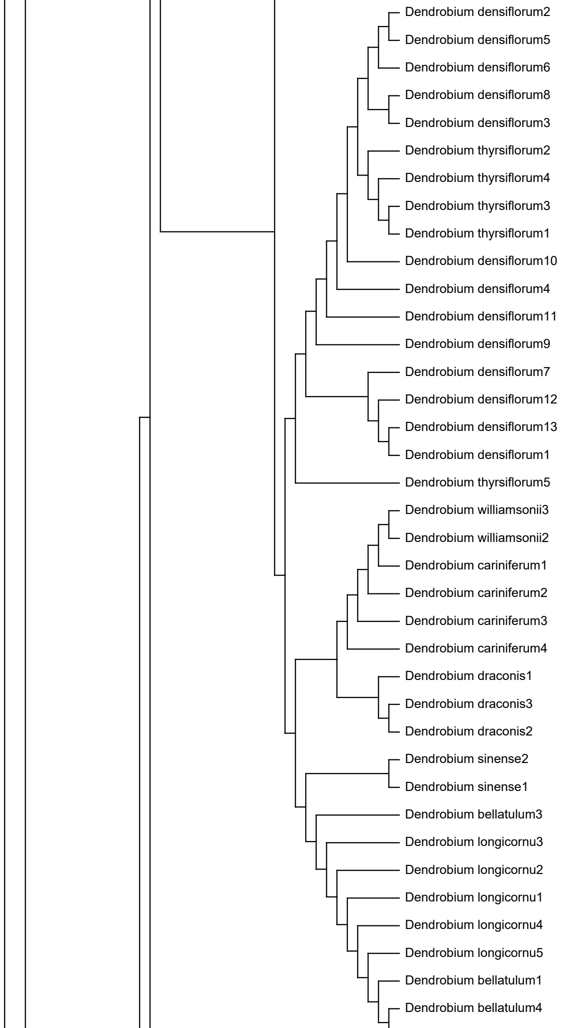
Dendrobium densiflorum2
Dendrobium densiflorum5
Dendrobium densiflorum6
Dendrobium densiflorum8
Dendrobium densiflorum3
Dendrobium thyrsiflorum2
Dendrobium thyrsiflorum4
Dendrobium thyrsiflorum3
Dendrobium thyrsiflorum1
Dendrobium densiflorum10
Dendrobium densiflorum4
Dendrobium densiflorum11
Dendrobium densiflorum9
Dendrobium densiflorum7
Dendrobium densiflorum12
Dendrobium densiflorum13
Dendrobium densiflorum1
Dendrobium thyrsiflorum5
Dendrobium williamsonii3
Dendrobium williamsonii2
Dendrobium cariniferum1
Dendrobium cariniferum2
Dendrobium cariniferum3
Dendrobium cariniferum4
Dendrobium draconis1
Dendrobium draconis3
Dendrobium draconis2
Dendrobium sinense2
Dendrobium sinense1
Dendrobium bellatulum3
Dendrobium longicornu3
Dendrobium longicornu2
Dendrobium longicornu1
Dendrobium longicornu4
Dendrobium longicornu5
Dendrobium bellatulum1
Dendrobium bellatulum4

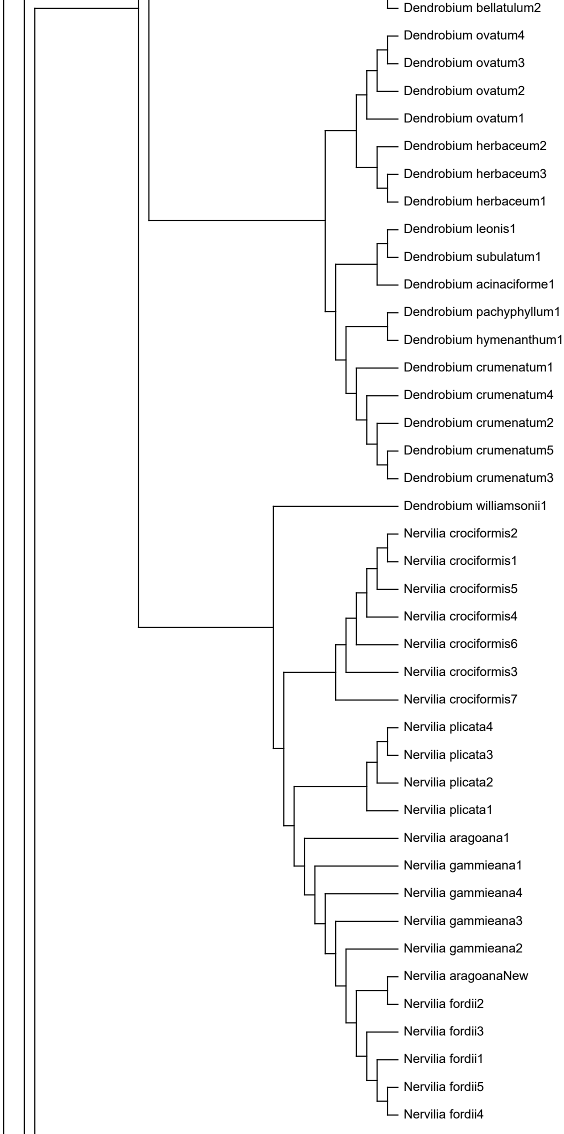
Dendrobium bellatulum2
Dendrobium ovatum4
Dendrobium ovatum3
Dendrobium ovatum2
Dendrobium ovatum1
Dendrobium herbaceum2
Dendrobium herbaceum3
Dendrobium herbaceum1
Dendrobium leonis1
Dendrobium subulatum1
Dendrobium acinaciforme1
Dendrobium pachyphyllum1
Dendrobium hymenanthum1
Dendrobium crumenatum1
Dendrobium crumenatum4
Dendrobium crumenatum2
Dendrobium crumenatum5
Dendrobium crumenatum3
Dendrobium williamsonii1
Nervilia crociformis2
Nervilia crociformis1
Nervilia crociformis5
Nervilia crociformis4
Nervilia crociformis6
Nervilia crociformis3
Nervilia crociformis7
Nervilia plicata4
Nervilia plicata3
Nervilia plicata2
Nervilia plicata1
Nervilia aragoana1
Nervilia gammieana1
Nervilia gammieana4
Nervilia gammieana3
Nervilia gammieana2
Nervilia aragoanaNew
Nervilia fordii2
Nervilia fordii3
Nervilia fordii1
Nervilia fordii5
Nervilia fordii4

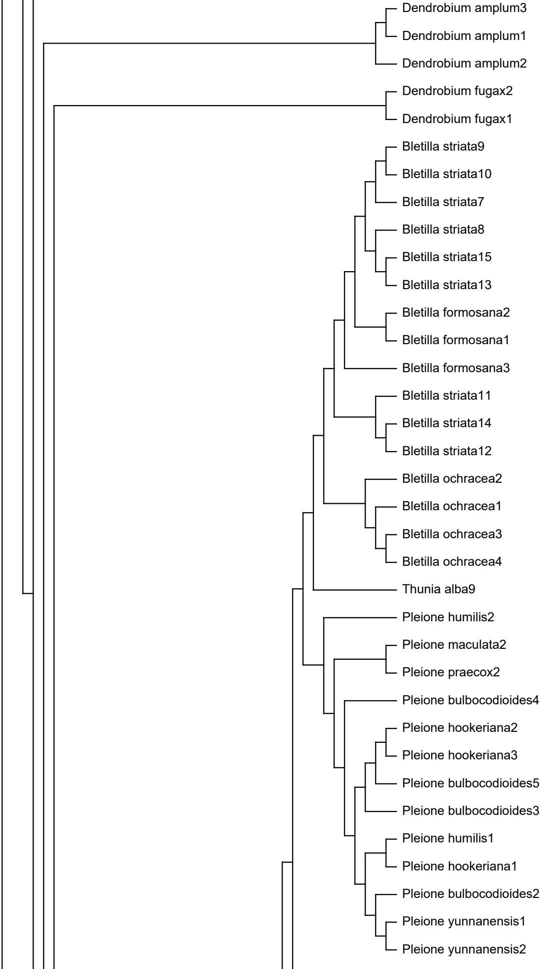
Dendrobium amplum3
Dendrobium amplum1
Dendrobium amplum2
Dendrobium fugax2
Dendrobium fugax1
Bletilla striata9
Bletilla striata10
Bletilla striata7
Bletilla striata8
Bletilla striata15
Bletilla striata13
Bletilla formosana2
Bletilla formosana1
Bletilla formosana3
Bletilla striata11
Bletilla striata14
Bletilla striata12
Bletilla ochracea2
Bletilla ochracea1
Bletilla ochracea3
Bletilla ochracea4
Thunia alba9
Pleione humilis2
Pleione maculata2
Pleione praecox2
Pleione bulbocodioides4
Pleione hookeriana2
Pleione hookeriana3
Pleione bulbocodioides5
Pleione bulbocodioides3
Pleione humilis1
Pleione hookeriana1
Pleione bulbocodioides2
Pleione yunnanensis1
Pleione yunnanensis2

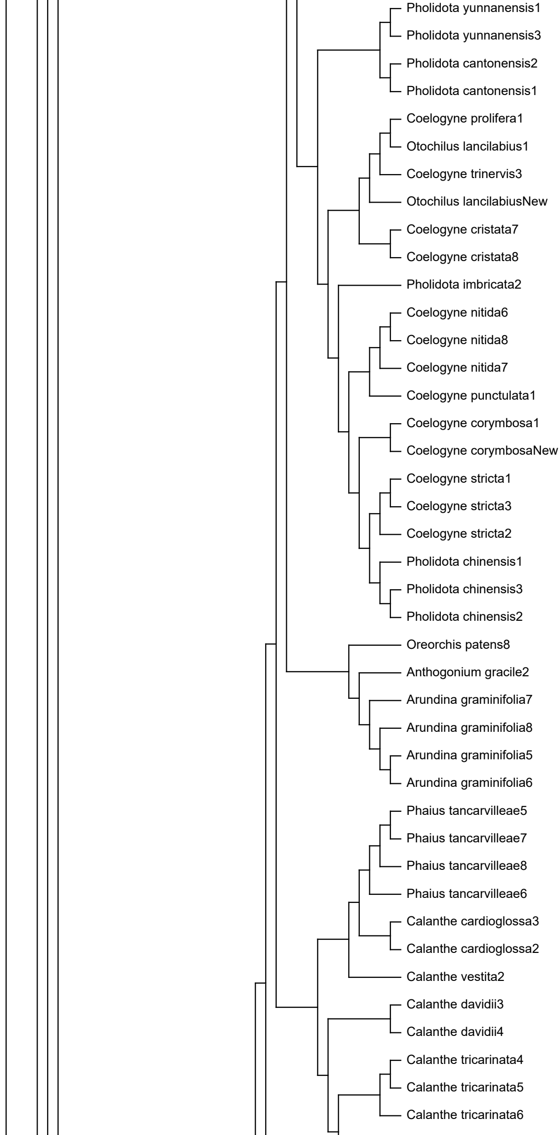

Pholidota yunnanensis1
Pholidota yunnanensis3
Pholidota cantonensis2
Pholidota cantonensis1
Coelogyne prolifera1
Otochilus lancilabius1
Coelogyne trinervis3
Otochilus lancilabiusNew
Coelogyne cristata7
Coelogyne cristata8
Pholidota imbricata2
Coelogyne nitida6
Coelogyne nitida8
Coelogyne nitida7
Coelogyne punctulata1
Coelogyne corymbosa1
Coelogyne corymbosaNew
Coelogyne stricta1
Coelogyne stricta3
Coelogyne stricta2
Pholidota chinensis1
Pholidota chinensis3
Pholidota chinensis2
Oreorchis patens8
Anthogonium gracile2
Arundina graminifolia7
Arundina graminifolia8
Arundina graminifolia5
Arundina graminifolia6
Phaius tancarvilleae5
Phaius tancarvilleae7
Phaius tancarvilleae8
Phaius tancarvilleae6
Calanthe cardioglossa3
Calanthe cardioglossa2
Calanthe vestita2
Calanthe davidii3
Calanthe davidii4
Calanthe tricarinata4
Calanthe tricarinata5
Calanthe tricarinata6

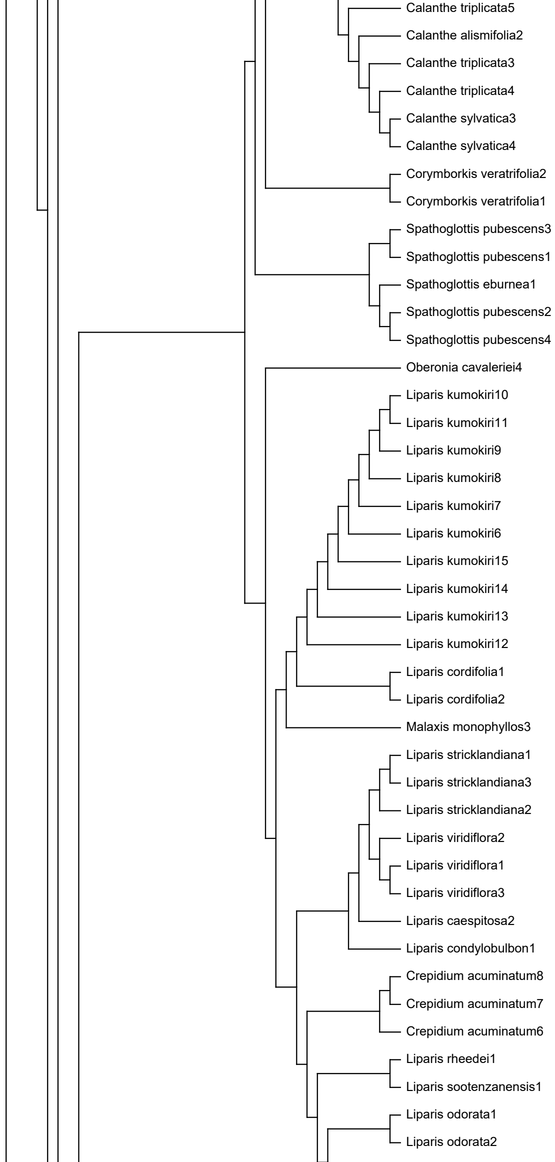

Calanthe triplicata5
Calanthe alismifolia2
Calanthe triplicata3
Calanthe triplicata4
Calanthe sylvatica3
Calanthe sylvatica4
Corymborkis veratrifolia2
Corymborkis veratrifolia1
Spathoglottis pubescens3
Spathoglottis pubescens1
Spathoglottis eburnea1
Spathoglottis pubescens2
Spathoglottis pubescens4
Oberonia cavaleriei4
Liparis kumokiri10
Liparis kumokiri11
Liparis kumokiri9
Liparis kumokiri8
Liparis kumokiri7
Liparis kumokiri6
Liparis kumokiri15
Liparis kumokiri14
Liparis kumokiri13
Liparis kumokiri12
Liparis cordifolia1
Liparis cordifolia2
Malaxis monophyllos3
Liparis stricklandiana1
Liparis stricklandiana3
Liparis stricklandiana2
Liparis viridiflora2
Liparis viridiflora1
Liparis viridiflora3
Liparis caespitosa2
Liparis condylobulbon1
Crepidium acuminatum8
Crepidium acuminatum7
Crepidium acuminatum6
Liparis rheedei1
Liparis sootenzanensis1
Liparis odorata1
Liparis odorata2

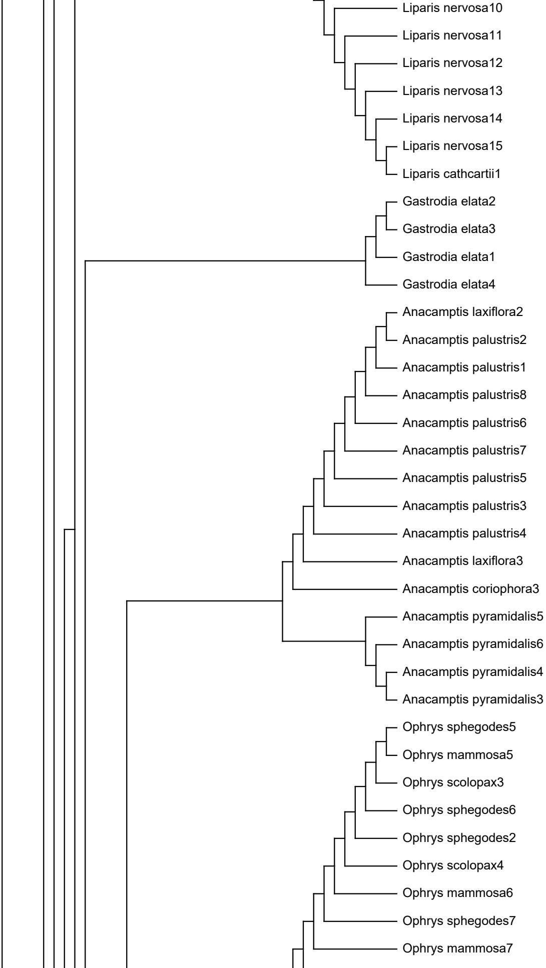
Liparis nervosa10
Liparis nervosa11
Liparis nervosa12
Liparis nervosa13
Liparis nervosa14
Liparis nervosa15
Liparis cathcartii1
Gastrodia elata2
Gastrodia elata3
Gastrodia elata1
Gastrodia elata4
Anacamptis laxiflora2
Anacamptis palustris2
Anacamptis palustris1
Anacamptis palustris8
Anacamptis palustris6
Anacamptis palustris7
Anacamptis palustris5
Anacamptis palustris3
Anacamptis palustris4
Anacamptis laxiflora3
Anacamptis coriophora3
Anacamptis pyramidalis5
Anacamptis pyramidalis6
Anacamptis pyramidalis4
Anacamptis pyramidalis3
Ophrys sphegodes5
Ophrys mammosa5
Ophrys scolopax3
Ophrys sphegodes6
Ophrys sphegodes2
Ophrys scolopax4
Ophrys mammosa6
Ophrys sphegodes7
Ophrys mammosa7

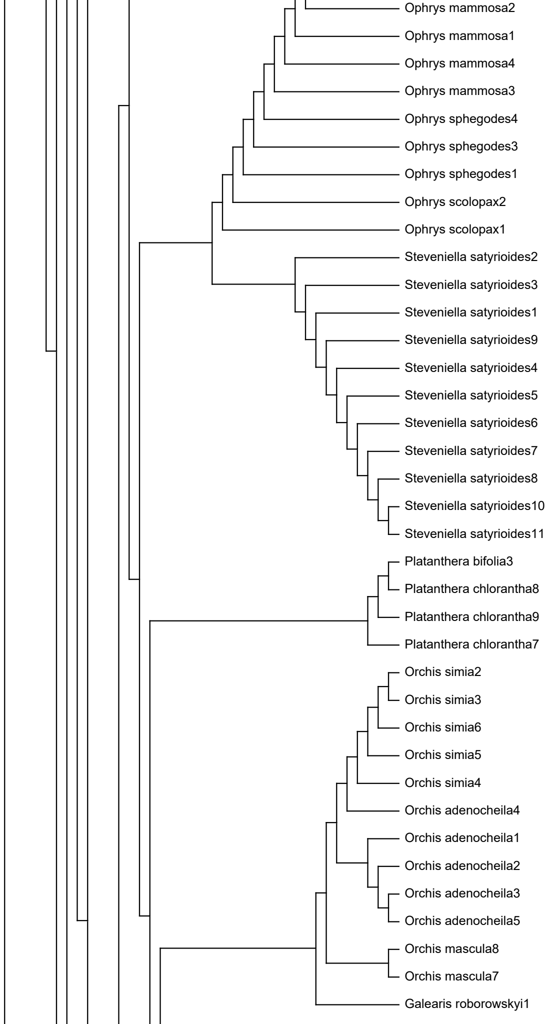
Ophrys mammosa2
Ophrys mammosa1
Ophrys mammosa4
Ophrys mammosa3
Ophrys sphegodes4
Ophrys sphegodes3
Ophrys sphegodes1
Ophrys scolopax2
Ophrys scolopax1
Steveniella satyrioides2
Steveniella satyrioides3
Steveniella satyrioides1
Steveniella satyrioides9
Steveniella satyrioides4
Steveniella satyrioides5
Steveniella satyrioides6
Steveniella satyrioides7
Steveniella satyrioides8
Steveniella satyrioides10
Steveniella satyrioides11
Platanthera bifolia3
Platanthera chlorantha8
Platanthera chlorantha9
Platanthera chlorantha7
Orchis simia2
Orchis simia3
Orchis simia6
Orchis simia5
Orchis simia4
Orchis adenocheila4
Orchis adenocheila1
Orchis adenocheila2
Orchis adenocheila3
Orchis adenocheila5
Orchis mascula8
Orchis mascula7
Galearis roborowskyi1

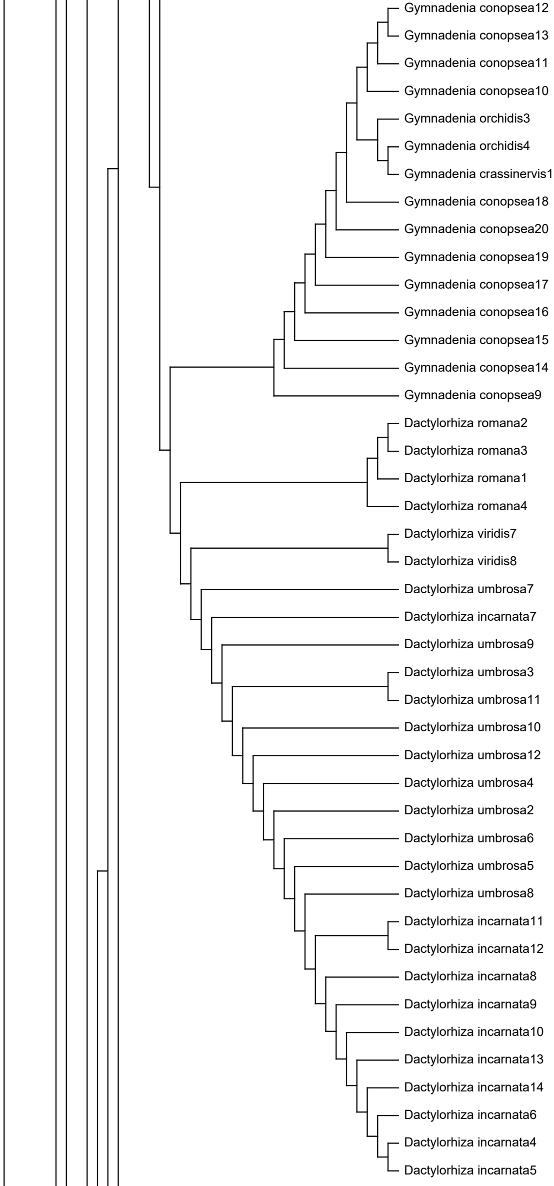

Gymnadenia conopsea12
Gymnadenia conopsea13
Gymnadenia conopsea11
Gymnadenia conopsea10
Gymnadenia orchidis3
Gymnadenia orchidis4
Gymnadenia crassinervis1
Gymnadenia conopsea18
Gymnadenia conopsea20
Gymnadenia conopsea19
Gymnadenia conopsea17
Gymnadenia conopsea16
Gymnadenia conopsea15
Gymnadenia conopsea14
Gymnadenia conopsea9
Dactylorhiza romana2
Dactylorhiza romana3
Dactylorhiza romana1
Dactylorhiza romana4
Dactylorhiza viridis7
Dactylorhiza viridis8
Dactylorhiza umbrosa7
Dactylorhiza incarnata7
Dactylorhiza umbrosa9
Dactylorhiza umbrosa3
Dactylorhiza umbrosa11
Dactylorhiza umbrosa10
Dactylorhiza umbrosa12
Dactylorhiza umbrosa4
Dactylorhiza umbrosa2
Dactylorhiza umbrosa6
Dactylorhiza umbrosa5
Dactylorhiza umbrosa8
Dactylorhiza incarnata11
Dactylorhiza incarnata12
Dactylorhiza incarnata8
Dactylorhiza incarnata9
Dactylorhiza incarnata10
Dactylorhiza incarnata13
Dactylorhiza incarnata14
Dactylorhiza incarnata6
Dactylorhiza incarnata4
Dactylorhiza incarnata5

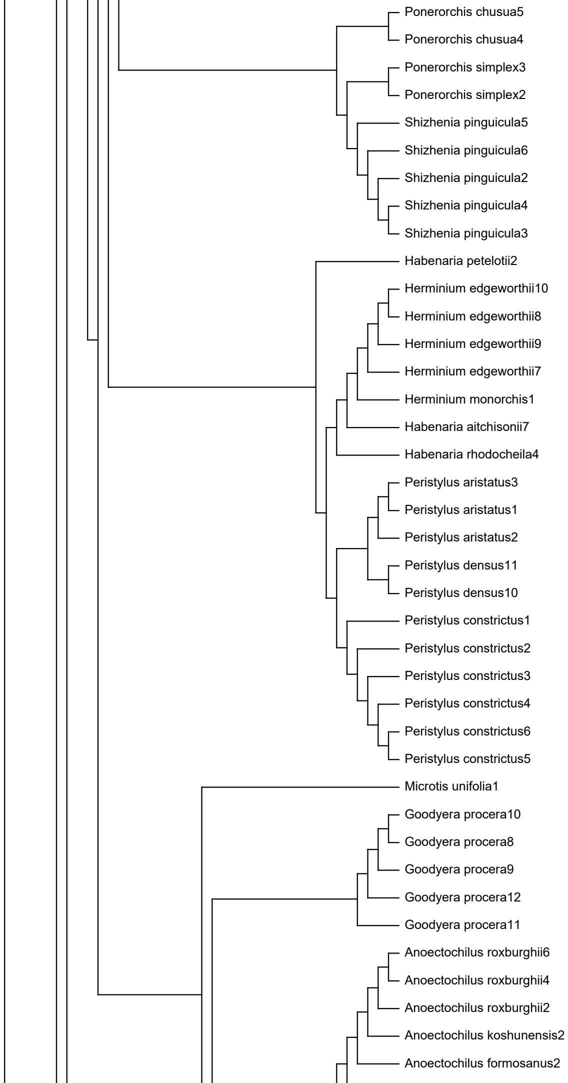

Ponerorchis chusua5
Ponerorchis chusua4
Ponerorchis simplex3
Ponerorchis simplex2
Shizhenia pinguicula5
Shizhenia pinguicula6
Shizhenia pinguicula2
Shizhenia pinguicula4
Shizhenia pinguicula3
Habenaria petelotii2
Herminium edgeworthii10
Herminium edgeworthii8
Herminium edgeworthii9
Herminium edgeworthii7
Herminium monorchis1
Habenaria aitchisonii7
Habenaria rhodocheila4
Peristylus aristatus3
Peristylus aristatus1
Peristylus aristatus2
Peristylus densus11
Peristylus densus10
Peristylus constrictus1
Peristylus constrictus2
Peristylus constrictus3
Peristylus constrictus4
Peristylus constrictus6
Peristylus constrictus5
Microtis unifolia1
Goodyera procera10
Goodyera procera8
Goodyera procera9
Goodyera procera12
Goodyera procera11
Anoectochilus roxburghii6
Anoectochilus roxburghii4
Anoectochilus roxburghii2
Anoectochilus koshunensis2
Anoectochilus formosanus2

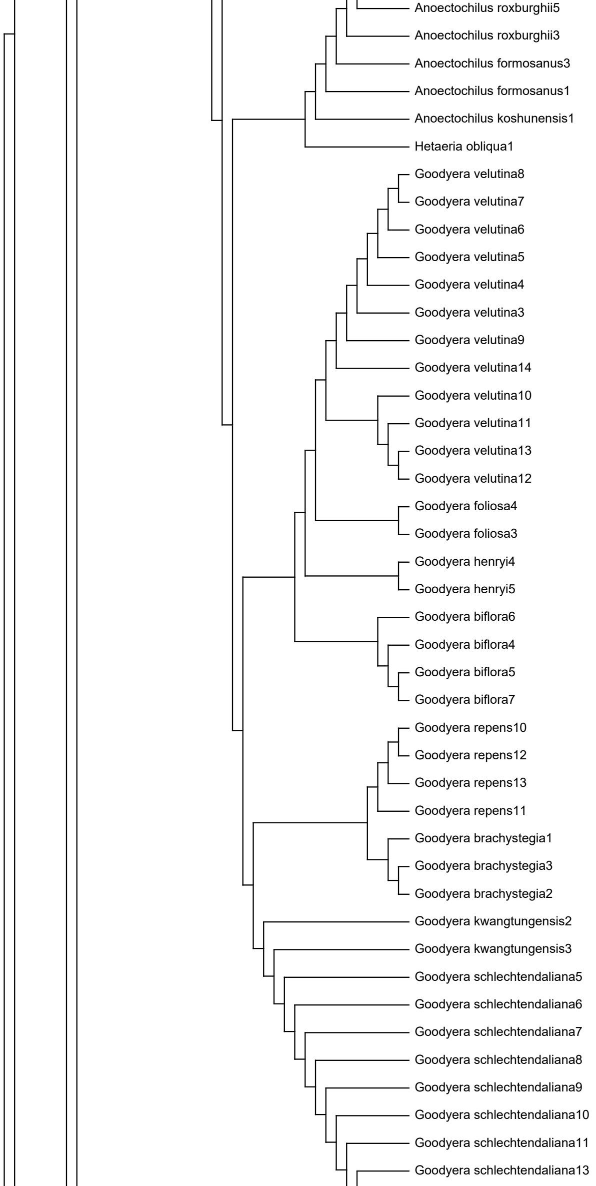
Anoectochilus roxburghii5
Anoectochilus roxburghii3
Anoectochilus formosanus3
Anoectochilus formosanus1
Anoectochilus koshunensis1
Hetaeria obliqua1
Goodyera velutina8
Goodyera velutina7
Goodyera velutina6
Goodyera velutina5
Goodyera velutina4
Goodyera velutina3
Goodyera velutina9
Goodyera velutina14
Goodyera velutina10
Goodyera velutina11
Goodyera velutina13
Goodyera velutina12
Goodyera foliosa4
Goodyera foliosa3
Goodyera henryi4
Goodyera henryi5
Goodyera biflora6
Goodyera biflora4
Goodyera biflora5
Goodyera biflora7
Goodyera repens10
Goodyera repens12
Goodyera repens13
Goodyera repens11
Goodyera brachystegia1
Goodyera brachystegia3
Goodyera brachystegia2
Goodyera kwangtungensis2
Goodyera kwangtungensis3
Goodyera schlechtendaliana5
Goodyera schlechtendaliana6
Goodyera schlechtendaliana7
Goodyera schlechtendaliana8
Goodyera schlechtendaliana9
Goodyera schlechtendaliana10
Goodyera schlechtendaliana11
Goodyera schlechtendaliana13

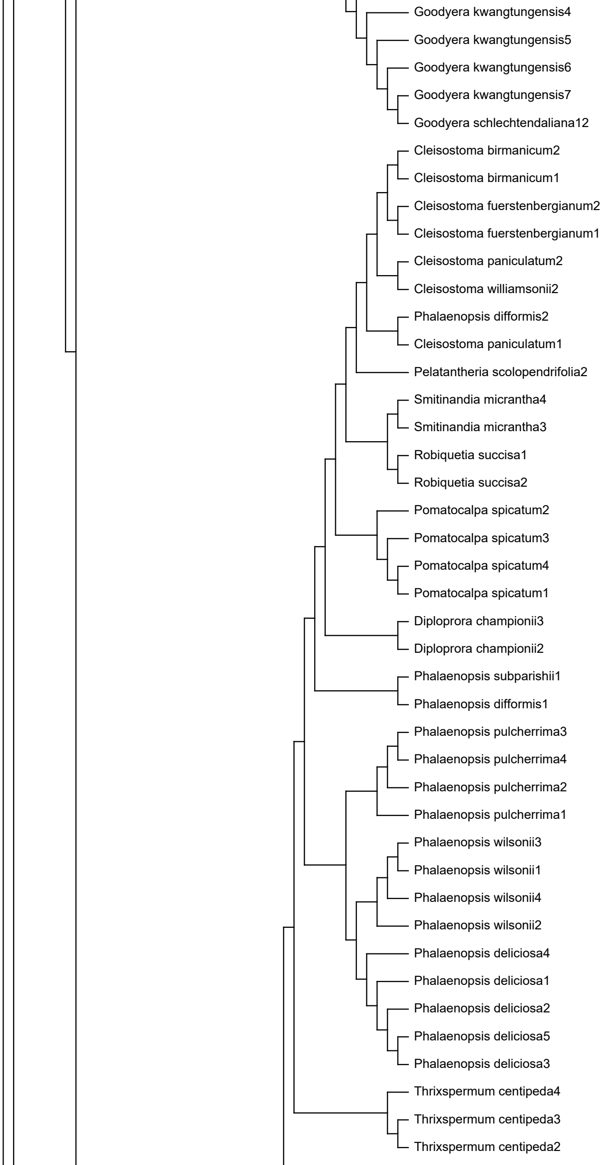
Goodyera kwangtungensis4
Goodyera kwangtungensis5
Goodyera kwangtungensis6
Goodyera kwangtungensis7
Goodyera schlechtendaliana12
Cleisostoma birmanicum2
Cleisostoma birmanicum1
Cleisostoma fuerstenbergianum2
Cleisostoma fuerstenbergianum1
Cleisostoma paniculatum2
Cleisostoma williamsonii2
Phalaenopsis difformis2
Cleisostoma paniculatum1
Pelatantheria scolopendrifolia2
Smitinandia micrantha4
Smitinandia micrantha3
Robiquetia succisa1
Robiquetia succisa2
Pomatocalpa spicatum2
Pomatocalpa spicatum3
Pomatocalpa spicatum4
Pomatocalpa spicatum1
Diploprora championii3
Diploprora championii2
Phalaenopsis subparishii1
Phalaenopsis difformis1
Phalaenopsis pulcherrima3
Phalaenopsis pulcherrima4
Phalaenopsis pulcherrima2
Phalaenopsis pulcherrima1
Phalaenopsis wilsonii3
Phalaenopsis wilsonii1
Phalaenopsis wilsonii4
Phalaenopsis wilsonii2
Phalaenopsis deliciosa4
Phalaenopsis deliciosa1
Phalaenopsis deliciosa2
Phalaenopsis deliciosa5
Phalaenopsis deliciosa3
Thrixspermum centipeda4
Thrixspermum centipeda3
Thrixspermum centipeda2

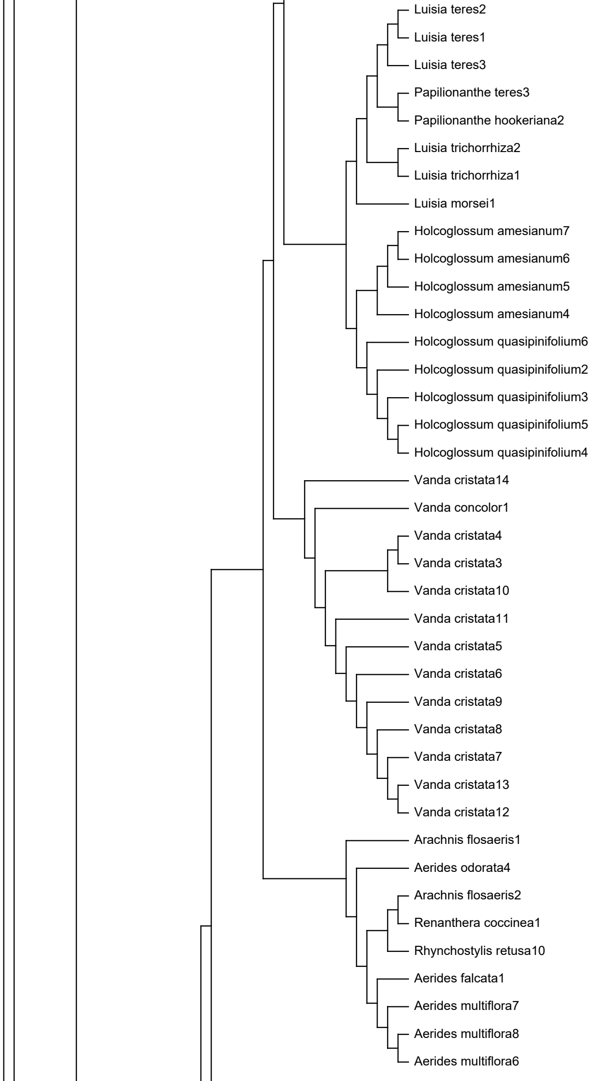

Luisia teres2
Luisia teres1
Luisia teres3
Papilionanthe teres3
Papilionanthe hookeriana2
Luisia trichorrhiza2
Luisia trichorrhiza1
Luisia morsei1
Holcoglossum amesianum7
Holcoglossum amesianum6
Holcoglossum amesianum5
Holcoglossum amesianum4
Holcoglossum quasipinifolium6
Holcoglossum quasipinifolium2
Holcoglossum quasipinifolium3
Holcoglossum quasipinifolium5
Holcoglossum quasipinifolium4
Vanda cristata14
Vanda concolor1
Vanda cristata4
Vanda cristata3
Vanda cristata10
Vanda cristata11
Vanda cristata5
Vanda cristata6
Vanda cristata9
Vanda cristata8
Vanda cristata7
Vanda cristata13
Vanda cristata12
Arachnis flosaeris1
Aerides odorata4
Arachnis flosaeris2
Renanthera coccinea1
Rhynchostylis retusa10
Aerides falcata1
Aerides multiflora7
Aerides multiflora8
Aerides multiflora6

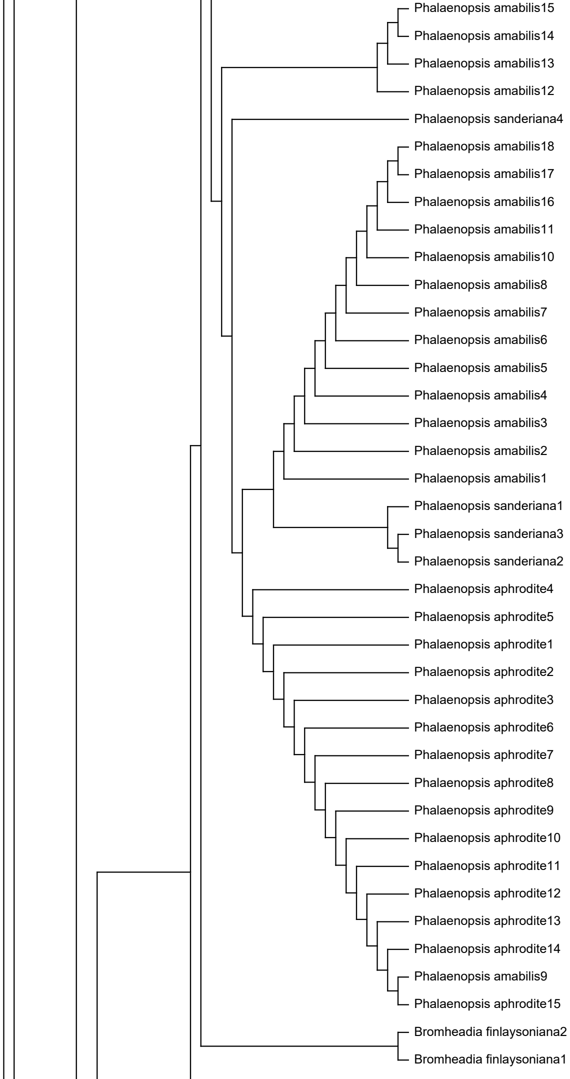
Phalaenopsis amabilis15
Phalaenopsis amabilis14
Phalaenopsis amabilis13
Phalaenopsis amabilis12
Phalaenopsis sanderiana4
Phalaenopsis amabilis18
Phalaenopsis amabilis17
Phalaenopsis amabilis16
Phalaenopsis amabilis11
Phalaenopsis amabilis10
Phalaenopsis amabilis8
Phalaenopsis amabilis7
Phalaenopsis amabilis6
Phalaenopsis amabilis5
Phalaenopsis amabilis4
Phalaenopsis amabilis3
Phalaenopsis amabilis2
Phalaenopsis amabilis1
Phalaenopsis sanderiana1
Phalaenopsis sanderiana3
Phalaenopsis sanderiana2
Phalaenopsis aphrodite4
Phalaenopsis aphrodite5
Phalaenopsis aphrodite1
Phalaenopsis aphrodite2
Phalaenopsis aphrodite3
Phalaenopsis aphrodite6
Phalaenopsis aphrodite7
Phalaenopsis aphrodite8
Phalaenopsis aphrodite9
Phalaenopsis aphrodite10
Phalaenopsis aphrodite11
Phalaenopsis aphrodite12
Phalaenopsis aphrodite13
Phalaenopsis aphrodite14
Phalaenopsis amabilis9
Phalaenopsis aphrodite15
Bromheadia finlaysoniana2
Bromheadia finlaysoniana1

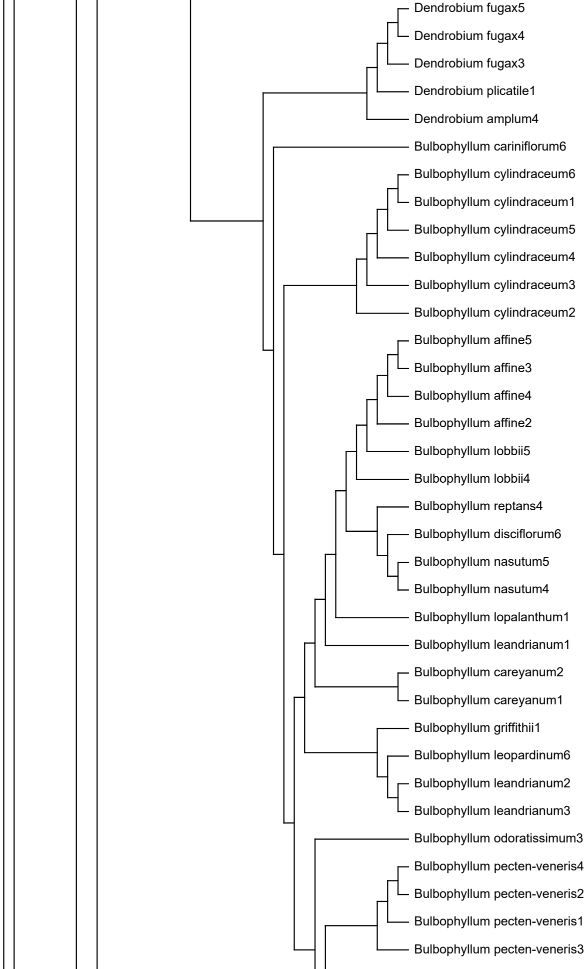
Dendrobium fugax5
Dendrobium fugax4
Dendrobium fugax3
Dendrobium plicatile1
Dendrobium amplum4
Bulbophyllum cariniflorum6
Bulbophyllum cylindraceum6
Bulbophyllum cylindraceum1
Bulbophyllum cylindraceum5
Bulbophyllum cylindraceum4
Bulbophyllum cylindraceum3
Bulbophyllum cylindraceum2
Bulbophyllum affine5
Bulbophyllum affine3
Bulbophyllum affine4
Bulbophyllum affine2
Bulbophyllum lobbii5
Bulbophyllum lobbii4
Bulbophyllum reptans4
Bulbophyllum disciflorum6
Bulbophyllum nasutum5
Bulbophyllum nasutum4
Bulbophyllum lopalanthum1
Bulbophyllum leandrianum1
Bulbophyllum careyanum2
Bulbophyllum careyanum1
Bulbophyllum griffithii1
Bulbophyllum leopardinum6
Bulbophyllum leandrianum2
Bulbophyllum leandrianum3
Bulbophyllum odoratissimum3
Bulbophyllum pecten-veneris4
Bulbophyllum pecten-veneris2
Bulbophyllum pecten-veneris1
Bulbophyllum pecten-veneris3

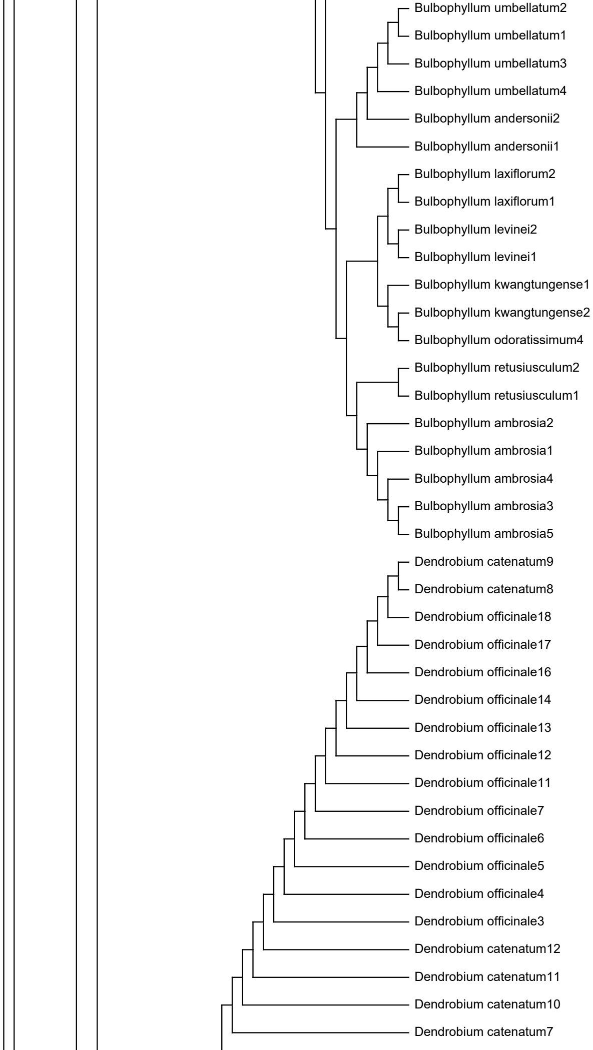
Bulbophyllum umbellatum2
Bulbophyllum umbellatum1
Bulbophyllum umbellatum3
Bulbophyllum umbellatum4
Bulbophyllum andersonii2
Bulbophyllum andersonii1
Bulbophyllum laxiflorum2
Bulbophyllum laxiflorum1
Bulbophyllum levinei2
Bulbophyllum levinei1
Bulbophyllum kwangtungense1
Bulbophyllum kwangtungense2
Bulbophyllum odoratissimum4
Bulbophyllum retusiusculum2
Bulbophyllum retusiusculum1
Bulbophyllum ambrosia2
Bulbophyllum ambrosia1
Bulbophyllum ambrosia4
Bulbophyllum ambrosia3
Bulbophyllum ambrosia5
Dendrobium catenatum9
Dendrobium catenatum8
Dendrobium officinale18
Dendrobium officinale17
Dendrobium officinale16
Dendrobium officinale14
Dendrobium officinale13
Dendrobium officinale12
Dendrobium officinale11
Dendrobium officinale7
Dendrobium officinale6
Dendrobium officinale5
Dendrobium officinale4
Dendrobium officinale3
Dendrobium catenatum12
Dendrobium catenatum11
Dendrobium catenatum10
Dendrobium catenatum7

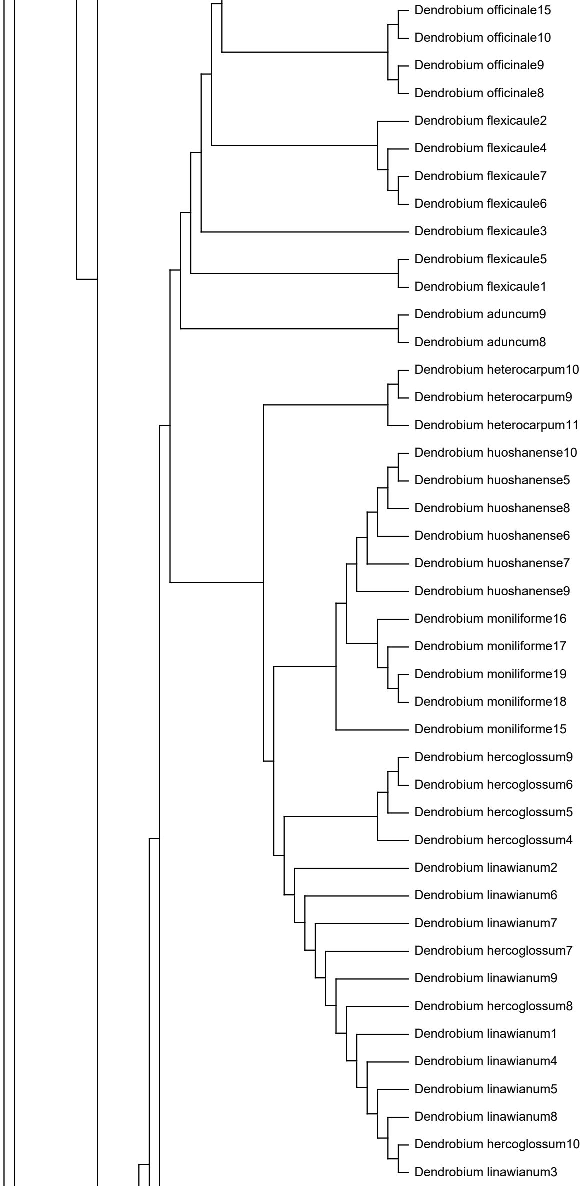
Dendrobium officinale15
Dendrobium officinale10
Dendrobium officinale9
Dendrobium officinale8
Dendrobium flexicaule2
Dendrobium flexicaule4
Dendrobium flexicaule7
Dendrobium flexicaule6
Dendrobium flexicaule3
Dendrobium flexicaule5
Dendrobium flexicaule1
Dendrobium aduncum9
Dendrobium aduncum8
Dendrobium heterocarpum10
Dendrobium heterocarpum9
Dendrobium heterocarpum11
Dendrobium huoshanense10
Dendrobium huoshanense5
Dendrobium huoshanense8
Dendrobium huoshanense6
Dendrobium huoshanense7
Dendrobium huoshanense9
Dendrobium moniliforme16
Dendrobium moniliforme17
Dendrobium moniliforme19
Dendrobium moniliforme18
Dendrobium moniliforme15
Dendrobium hercoglossum9
Dendrobium hercoglossum6
Dendrobium hercoglossum5
Dendrobium hercoglossum4
Dendrobium linawianum2
Dendrobium linawianum6
Dendrobium linawianum7
Dendrobium hercoglossum7
Dendrobium linawianum9
Dendrobium hercoglossum8
Dendrobium linawianum1
Dendrobium linawianum4
Dendrobium linawianum5
Dendrobium linawianum8
Dendrobium hercoglossum10
Dendrobium linawianum3

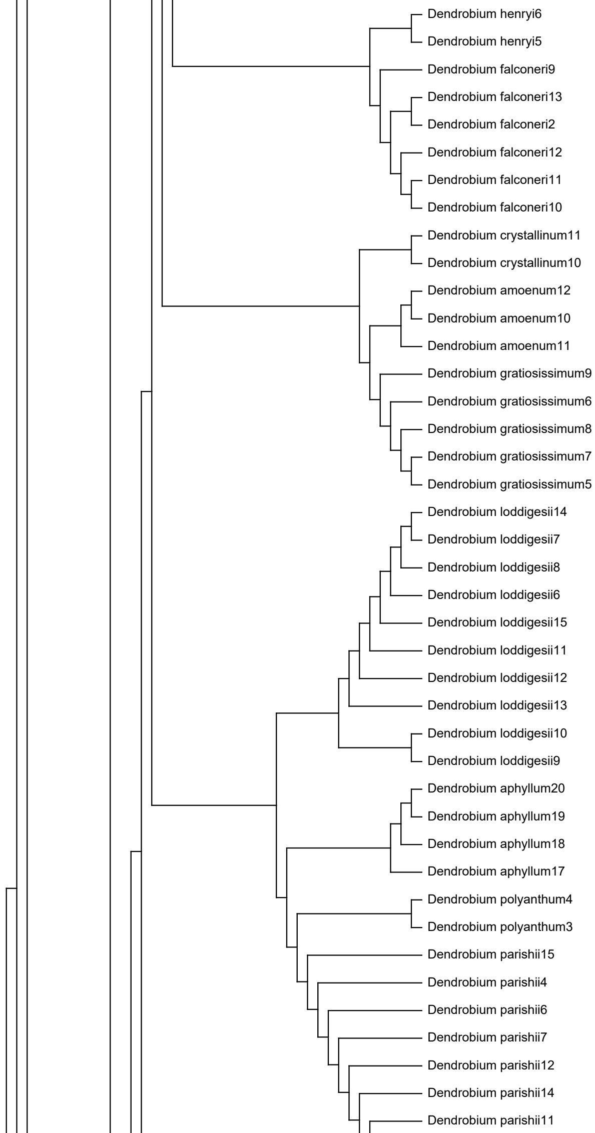
Dendrobium henryi6
Dendrobium henryi5
Dendrobium falconeri9
Dendrobium falconeri13
Dendrobium falconeri2
Dendrobium falconeri12
Dendrobium falconeri11
Dendrobium falconeri10
Dendrobium crystallinum11
Dendrobium crystallinum10
Dendrobium amoenum12
Dendrobium amoenum10
Dendrobium amoenum11
Dendrobium gratiosissimum9
Dendrobium gratiosissimum6
Dendrobium gratiosissimum8
Dendrobium gratiosissimum7
Dendrobium gratiosissimum5
Dendrobium loddigesii14
Dendrobium loddigesii7
Dendrobium loddigesii8
Dendrobium loddigesii6
Dendrobium loddigesii15
Dendrobium loddigesii11
Dendrobium loddigesii12
Dendrobium loddigesii13
Dendrobium loddigesii10
Dendrobium loddigesii9
Dendrobium aphyllum20
Dendrobium aphyllum19
Dendrobium aphyllum18
Dendrobium aphyllum17
Dendrobium polyanthum4
Dendrobium polyanthum3
Dendrobium parishii15
Dendrobium parishii4
Dendrobium parishii6
Dendrobium parishii7
Dendrobium parishii12
Dendrobium parishii14
Dendrobium parishii11

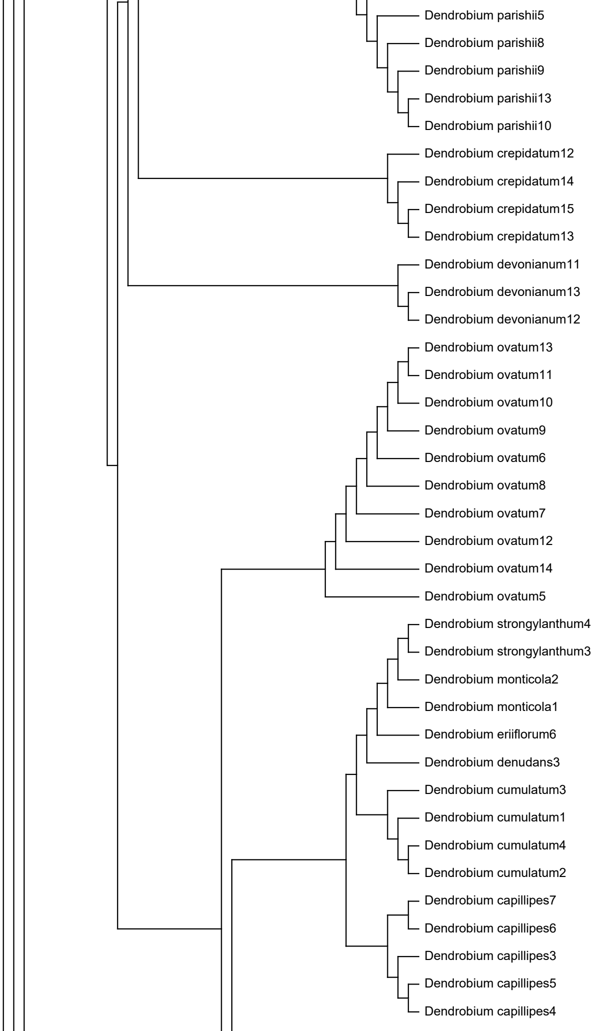
Dendrobium parishii5
Dendrobium parishii8
Dendrobium parishii9
Dendrobium parishii13
Dendrobium parishii10
Dendrobium crepidatum12
Dendrobium crepidatum14
Dendrobium crepidatum15
Dendrobium crepidatum13
Dendrobium devonianum11
Dendrobium devonianum13
Dendrobium devonianum12
Dendrobium ovatum13
Dendrobium ovatum11
Dendrobium ovatum10
Dendrobium ovatum9
Dendrobium ovatum6
Dendrobium ovatum8
Dendrobium ovatum7
Dendrobium ovatum12
Dendrobium ovatum14
Dendrobium ovatum5
Dendrobium strongylanthum4
Dendrobium strongylanthum3
Dendrobium monticola2
Dendrobium monticola1
Dendrobium eriiflorum6
Dendrobium denudans3
Dendrobium cumulatum3
Dendrobium cumulatum1
Dendrobium cumulatum4
Dendrobium cumulatum2
Dendrobium capillipes7
Dendrobium capillipes6
Dendrobium capillipes3
Dendrobium capillipes5
Dendrobium capillipes4

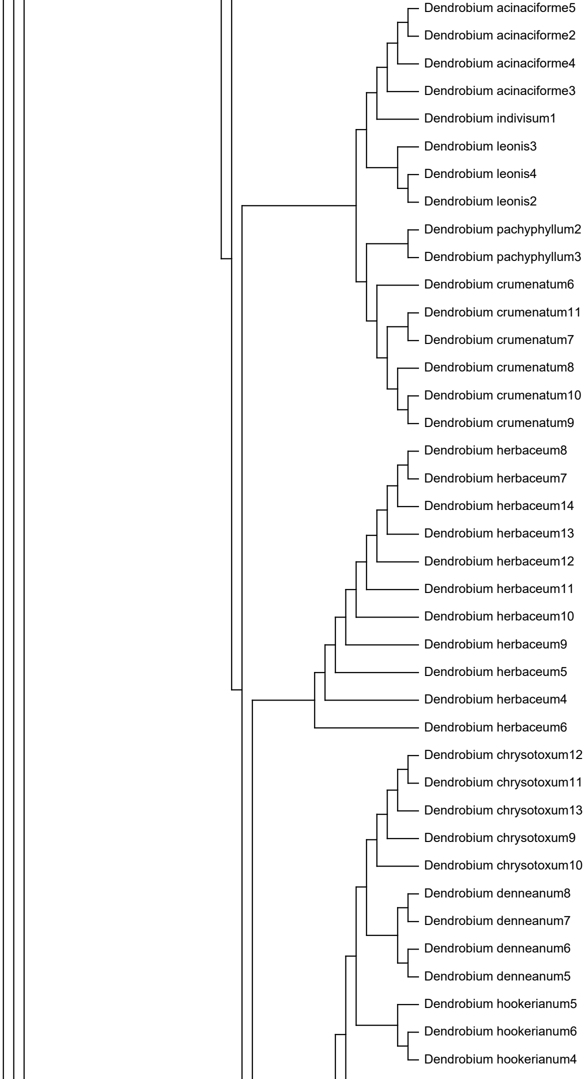
Dendrobium acinaciforme5
Dendrobium acinaciforme2
Dendrobium acinaciforme4
Dendrobium acinaciforme3
Dendrobium indivisum1
Dendrobium leonis3
Dendrobium leonis4
Dendrobium leonis2
Dendrobium pachyphyllum2
Dendrobium pachyphyllum3
Dendrobium crumenatum6
Dendrobium crumenatum11
Dendrobium crumenatum7
Dendrobium crumenatum8
Dendrobium crumenatum10
Dendrobium crumenatum9
Dendrobium herbaceum8
Dendrobium herbaceum7
Dendrobium herbaceum14
Dendrobium herbaceum13
Dendrobium herbaceum12
Dendrobium herbaceum11
Dendrobium herbaceum10
Dendrobium herbaceum9
Dendrobium herbaceum5
Dendrobium herbaceum4
Dendrobium herbaceum6
Dendrobium chrysotoxum12
Dendrobium chrysotoxum11
Dendrobium chrysotoxum13
Dendrobium chrysotoxum9
Dendrobium chrysotoxum10
Dendrobium denneanum8
Dendrobium denneanum7
Dendrobium denneanum6
Dendrobium denneanum5
Dendrobium hookerianum5
Dendrobium hookerianum6
Dendrobium hookerianum4

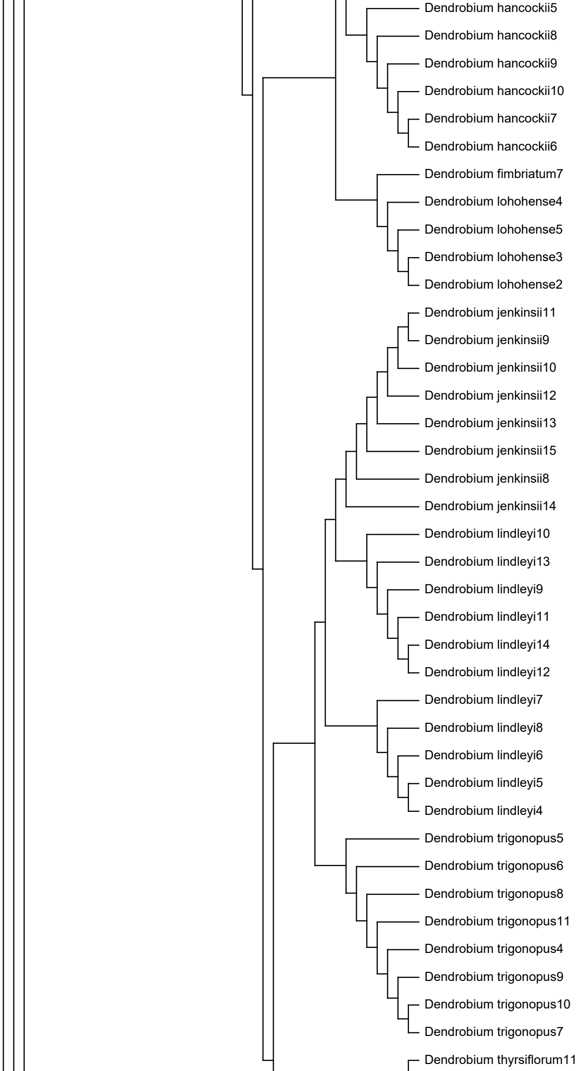
Dendrobium hancockii5
Dendrobium hancockii8
Dendrobium hancockii9
Dendrobium hancockii10
Dendrobium hancockii7
Dendrobium hancockii6
Dendrobium fimbriatum7
Dendrobium lohohense4
Dendrobium lohohense5
Dendrobium lohohense3
Dendrobium lohohense2
Dendrobium jenkinsii11
Dendrobium jenkinsii9
Dendrobium jenkinsii10
Dendrobium jenkinsii12
Dendrobium jenkinsii13
Dendrobium jenkinsii15
Dendrobium jenkinsii8
Dendrobium jenkinsii14
Dendrobium lindleyi10
Dendrobium lindleyi13
Dendrobium lindleyi9
Dendrobium lindleyi11
Dendrobium lindleyi14
Dendrobium lindleyi12
Dendrobium lindleyi7
Dendrobium lindleyi8
Dendrobium lindleyi6
Dendrobium lindleyi5
Dendrobium lindleyi4
Dendrobium trigonopus5
Dendrobium trigonopus6
Dendrobium trigonopus8
Dendrobium trigonopus11
Dendrobium trigonopus4
Dendrobium trigonopus9
Dendrobium trigonopus10
Dendrobium trigonopus7
Dendrobium thyrsiflorum11

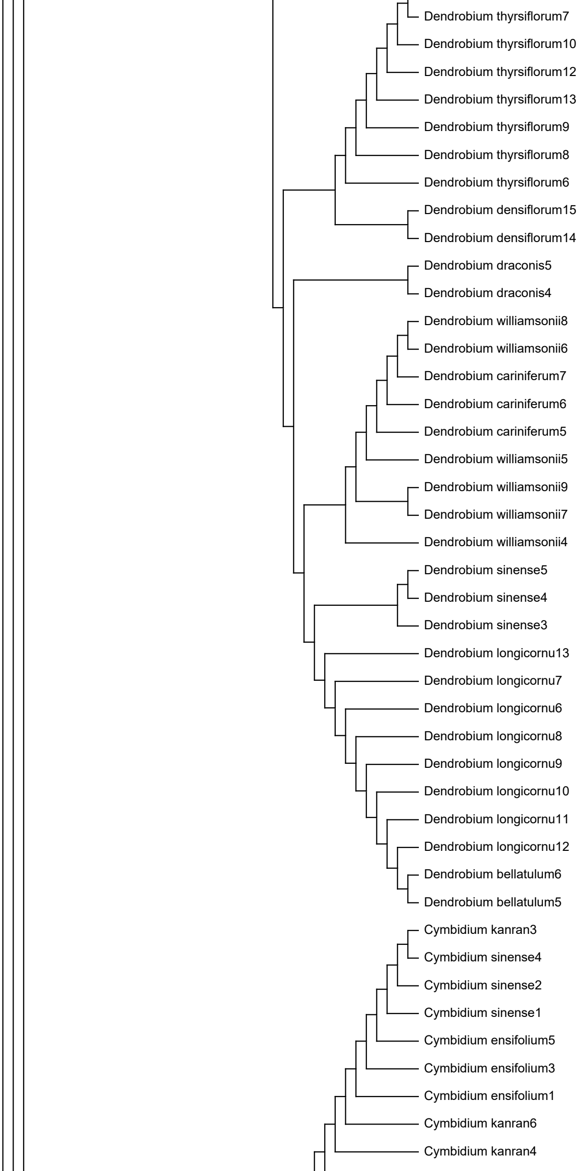
Dendrobium thyrsiflorum7
Dendrobium thyrsiflorum10
Dendrobium thyrsiflorum12
Dendrobium thyrsiflorum13
Dendrobium thyrsiflorum9
Dendrobium thyrsiflorum8
Dendrobium thyrsiflorum6
Dendrobium densiflorum15
Dendrobium densiflorum14
Dendrobium draconis5
Dendrobium draconis4
Dendrobium williamsonii8
Dendrobium williamsonii6
Dendrobium cariniferum7
Dendrobium cariniferum6
Dendrobium cariniferum5
Dendrobium williamsonii5
Dendrobium williamsonii9
Dendrobium williamsonii7
Dendrobium williamsonii4
Dendrobium sinense5
Dendrobium sinense4
Dendrobium sinense3
Dendrobium longicornu13
Dendrobium longicornu7
Dendrobium longicornu6
Dendrobium longicornu8
Dendrobium longicornu9
Dendrobium longicornu10
Dendrobium longicornu11
Dendrobium longicornu12
Dendrobium bellatulum6
Dendrobium bellatulum5
Cymbidium kanran3
Cymbidium sinense4
Cymbidium sinense2
Cymbidium sinense1
Cymbidium ensifolium5
Cymbidium ensifolium3
Cymbidium ensifolium1
Cymbidium kanran6
Cymbidium kanran4

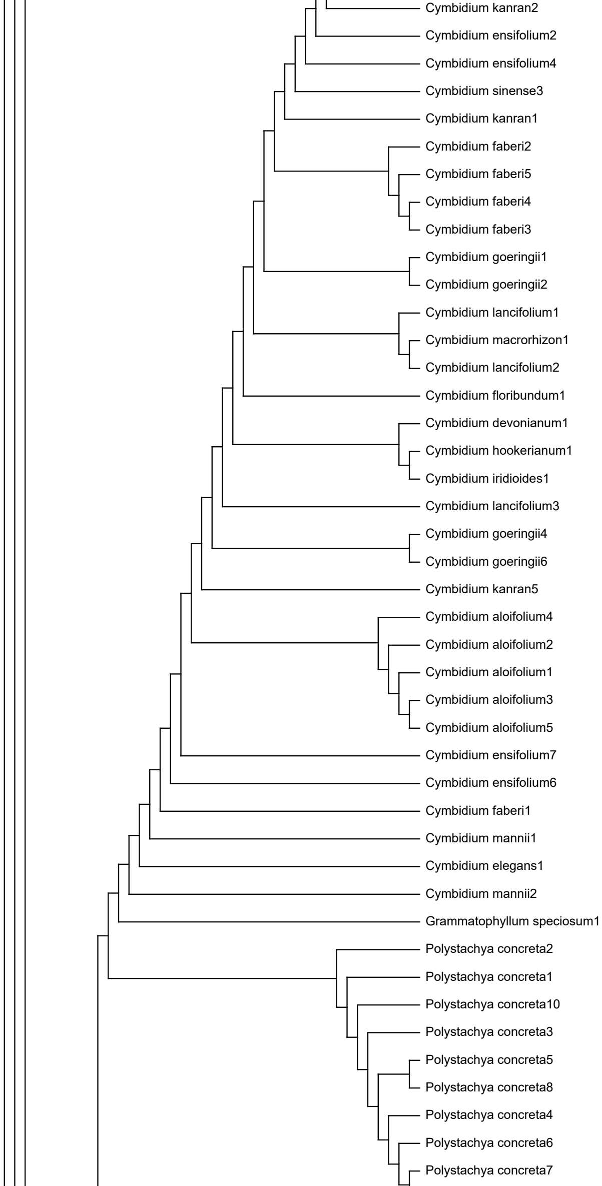
Cymbidium kanran2
Cymbidium ensifolium2
Cymbidium ensifolium4
Cymbidium sinense3
Cymbidium kanran1
Cymbidium faberi2
Cymbidium faberi5
Cymbidium faberi4
Cymbidium faberi3
Cymbidium goeringii1
Cymbidium goeringii2
Cymbidium lancifolium1
Cymbidium macrorhizon1
Cymbidium lancifolium2
Cymbidium floribundum1
Cymbidium devonianum1
Cymbidium hookerianum1
Cymbidium iridioides1
Cymbidium lancifolium3
Cymbidium goeringii4
Cymbidium goeringii6
Cymbidium kanran5
Cymbidium aloifolium4
Cymbidium aloifolium2
Cymbidium aloifolium1
Cymbidium aloifolium3
Cymbidium aloifolium5
Cymbidium ensifolium7
Cymbidium ensifolium6
Cymbidium faberi1
Cymbidium mannii1
Cymbidium elegans1
Cymbidium mannii2
Grammatophyllum speciosum1
Polystachya concreta2
Polystachya concreta1
Polystachya concreta10
Polystachya concreta3
Polystachya concreta5
Polystachya concreta8
Polystachya concreta4
Polystachya concreta6
Polystachya concreta7

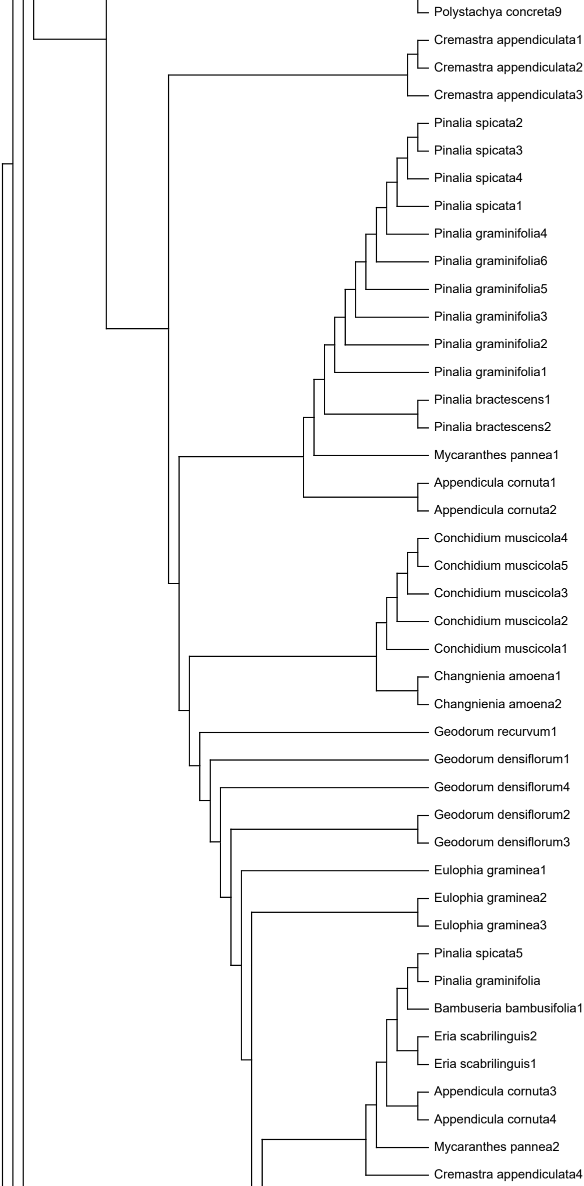
Polystachya concreta9
Cremastra appendiculata1
Cremastra appendiculata2
Cremastra appendiculata3
Pinalia spicata2
Pinalia spicata3
Pinalia spicata4
Pinalia spicata1
Pinalia graminifolia4
Pinalia graminifolia6
Pinalia graminifolia5
Pinalia graminifolia3
Pinalia graminifolia2
Pinalia graminifolia1
Pinalia bractescens1
Pinalia bractescens2
Mycaranthes pannea1
Appendicula cornuta1
Appendicula cornuta2
Conchidium muscicola4
Conchidium muscicola5
Conchidium muscicola3
Conchidium muscicola2
Conchidium muscicola1
Changnienia amoena1
Changnienia amoena2
Geodorum recurvum1
Geodorum densiflorum1
Geodorum densiflorum4
Geodorum densiflorum2
Geodorum densiflorum3
Eulophia graminea1
Eulophia graminea2
Eulophia graminea3
Pinalia spicata5
Pinalia graminifolia
Bambuseria bambusifolia1
Eria scabrilinguis2
Eria scabrilinguis1
Appendicula cornuta3
Appendicula cornuta4
Mycaranthes pannea2
Cremastra appendiculata4

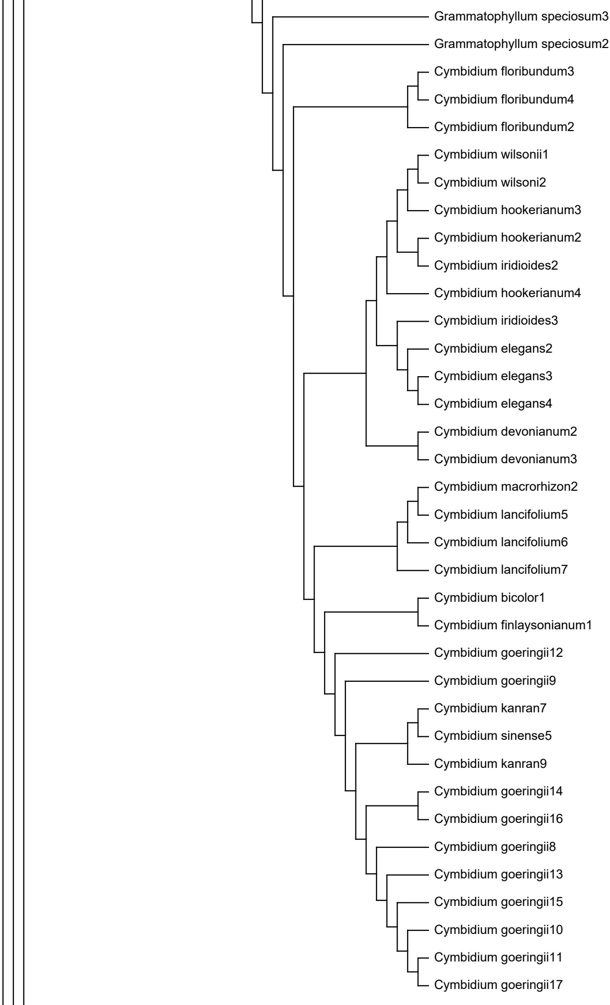

Grammatophyllum speciosum3
Grammatophyllum speciosum2
Cymbidium floribundum3
Cymbidium floribundum4
Cymbidium floribundum2
Cymbidium wilsonii1
Cymbidium wilsoni2
Cymbidium hookerianum3
Cymbidium hookerianum2
Cymbidium iridioides2
Cymbidium hookerianum4
Cymbidium iridioides3
Cymbidium elegans2
Cymbidium elegans3
Cymbidium elegans4
Cymbidium devonianum2
Cymbidium devonianum3
Cymbidium macrorhizon2
Cymbidium lancifolium5
Cymbidium lancifolium6
Cymbidium lancifolium7
Cymbidium bicolor1
Cymbidium finlaysonianum1
Cymbidium goeringii12
Cymbidium goeringii9
Cymbidium kanran7
Cymbidium sinense5
Cymbidium kanran9
Cymbidium goeringii14
Cymbidium goeringii16
Cymbidium goeringii8
Cymbidium goeringii13
Cymbidium goeringii15
Cymbidium goeringii10
Cymbidium goeringii11
Cymbidium goeringii17

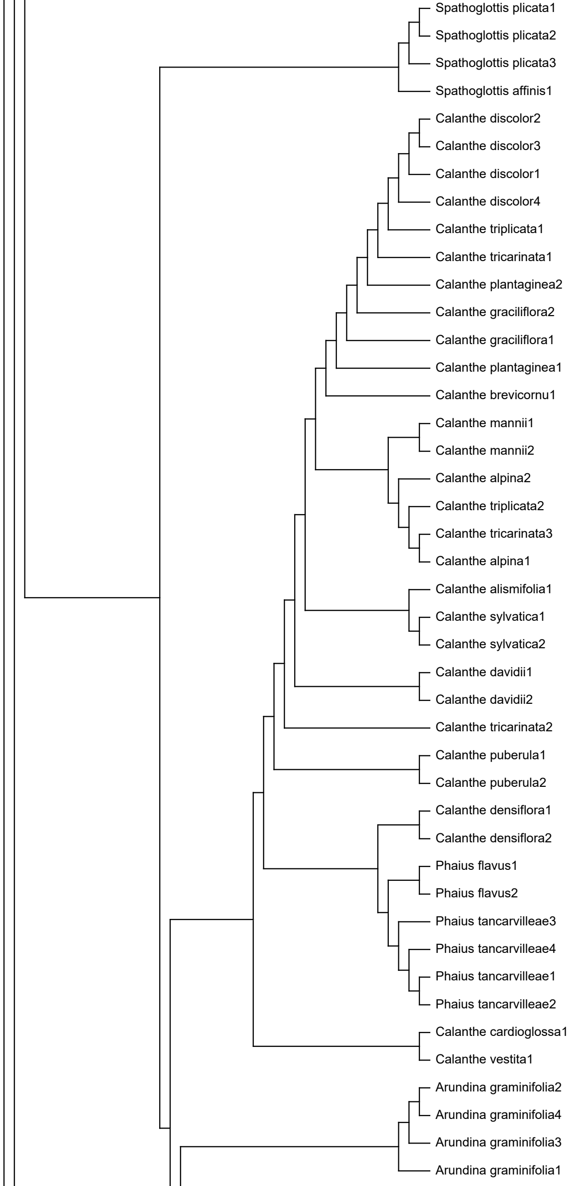
Spathoglottis plicata1
Spathoglottis plicata2
Spathoglottis plicata3
Spathoglottis affinis1
Calanthe discolor2
Calanthe discolor3
Calanthe discolor1
Calanthe discolor4
Calanthe triplicata1
Calanthe tricarinata1
Calanthe plantaginea2
Calanthe graciliflora2
Calanthe graciliflora1
Calanthe plantaginea1
Calanthe brevicornu1
Calanthe mannii1
Calanthe mannii2
Calanthe alpina2
Calanthe triplicata2
Calanthe tricarinata3
Calanthe alpina1
Calanthe alismifolia1
Calanthe sylvatica1
Calanthe sylvatica2
Calanthe davidii1
Calanthe davidii2
Calanthe tricarinata2
Calanthe puberula1
Calanthe puberula2
Calanthe densiflora1
Calanthe densiflora2
Phaius flavus1
Phaius flavus2
Phaius tancarvilleae3
Phaius tancarvilleae4
Phaius tancarvilleae1
Phaius tancarvilleae2
Calanthe cardioglossa1
Calanthe vestita1
Arundina graminifolia2
Arundina graminifolia4
Arundina graminifolia3
Arundina graminifolia1

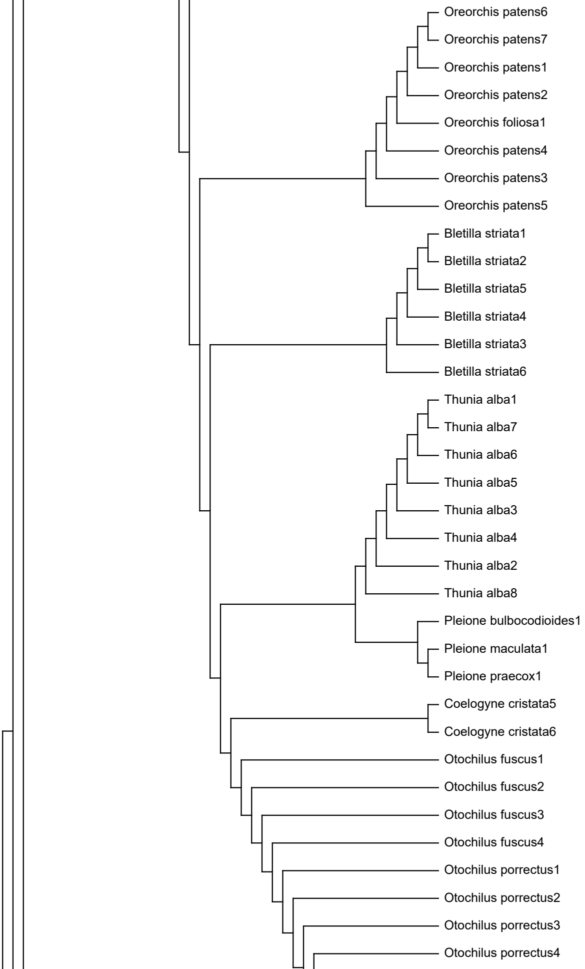
Oreorchis patens6
Oreorchis patens7
Oreorchis patens1
Oreorchis patens2
Oreorchis foliosa1
Oreorchis patens4
Oreorchis patens3
Oreorchis patens5
Bletilla striata1
Bletilla striata2
Bletilla striata5
Bletilla striata4
Bletilla striata3
Bletilla striata6
Thunia alba1
Thunia alba7
Thunia alba6
Thunia alba5
Thunia alba3
Thunia alba4
Thunia alba2
Thunia alba8
Pleione bulbocodioides1
Pleione maculata1
Pleione praecox1
Coelogyne cristata5
Coelogyne cristata6
Otochilus fuscus1
Otochilus fuscus2
Otochilus fuscus3
Otochilus fuscus4
Otochilus porrectus1
Otochilus porrectus2
Otochilus porrectus3
Otochilus porrectus4

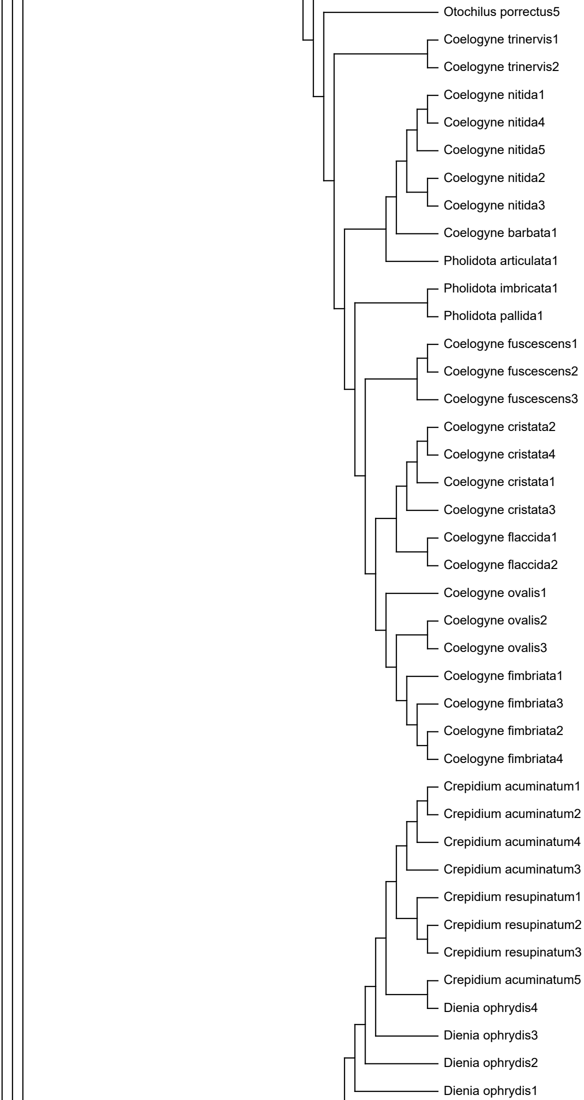
Otochilus porrectus5
Coelogyne trinervis1
Coelogyne trinervis2
Coelogyne nitida1
Coelogyne nitida4
Coelogyne nitida5
Coelogyne nitida2
Coelogyne nitida3
Coelogyne barbata1
Pholidota articulata1
Pholidota imbricata1
Pholidota pallida1
Coelogyne fuscescens1
Coelogyne fuscescens2
Coelogyne fuscescens3
Coelogyne cristata2
Coelogyne cristata4
Coelogyne cristata1
Coelogyne cristata3
Coelogyne flaccida1
Coelogyne flaccida2
Coelogyne ovalis1
Coelogyne ovalis2
Coelogyne ovalis3
Coelogyne fimbriata1
Coelogyne fimbriata3
Coelogyne fimbriata2
Coelogyne fimbriata4
Crepidium acuminatum1
Crepidium acuminatum2
Crepidium acuminatum4
Crepidium acuminatum3
Crepidium resupinatum1
Crepidium resupinatum2
Crepidium resupinatum3
Crepidium acuminatum5
Dienia ophrydis4
Dienia ophrydis3
Dienia ophrydis2
Dienia ophrydis1

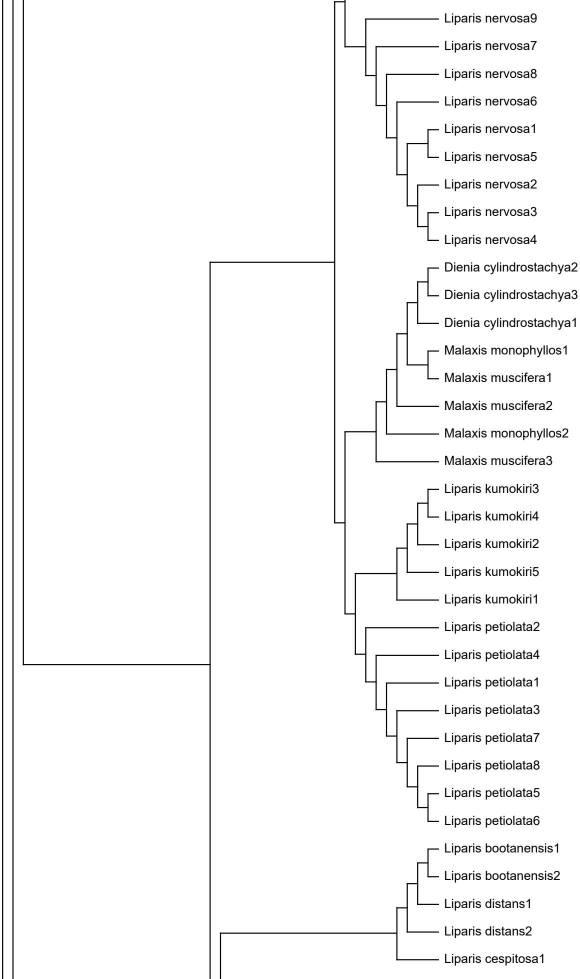
Liparis nervosa9
Liparis nervosa7
Liparis nervosa8
Liparis nervosa6
Liparis nervosa1
Liparis nervosa5
Liparis nervosa2
Liparis nervosa3
Liparis nervosa4
Dienia cylindrostachya2
Dienia cylindrostachya3
Dienia cylindrostachya1
Malaxis monophyllos1
Malaxis muscifera1
Malaxis muscifera2
Malaxis monophyllos2
Malaxis muscifera3
Liparis kumokiri3
Liparis kumokiri4
Liparis kumokiri2
Liparis kumokiri5
Liparis kumokiri1
Liparis petiolata2
Liparis petiolata4
Liparis petiolata1
Liparis petiolata3
Liparis petiolata7
Liparis petiolata8
Liparis petiolata5
Liparis petiolata6
Liparis bootanensis1
Liparis bootanensis2
Liparis distans1
Liparis distans2
Liparis cespitosa1

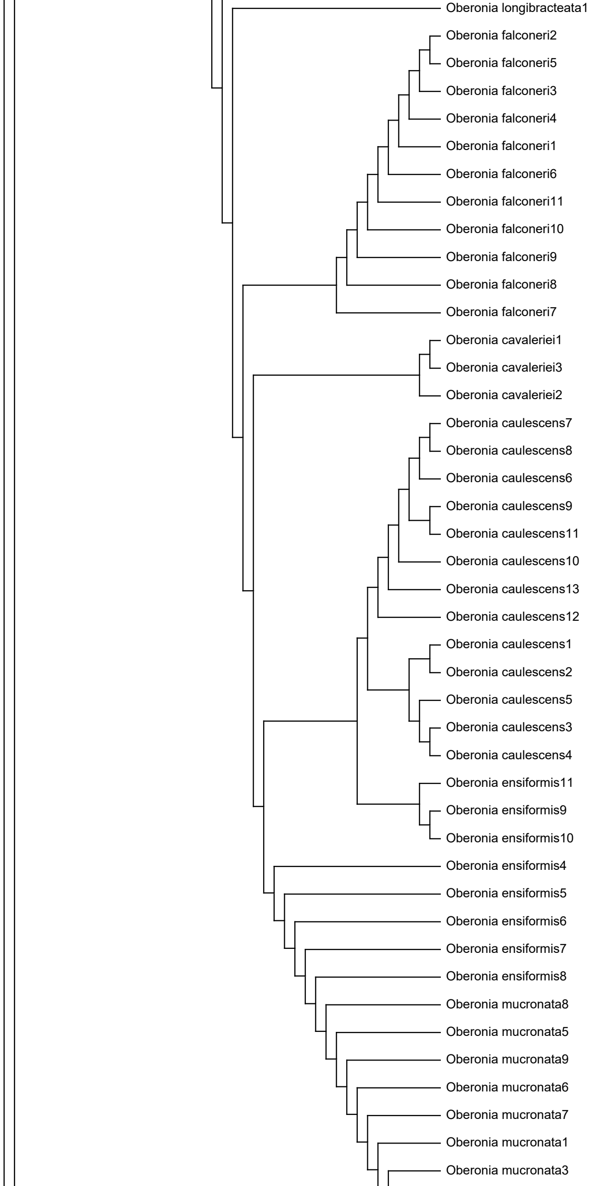
Oberonia longibracteata1
Oberonia falconeri2
Oberonia falconeri5
Oberonia falconeri3
Oberonia falconeri4
Oberonia falconeri1
Oberonia falconeri6
Oberonia falconeri11
Oberonia falconeri10
Oberonia falconeri9
Oberonia falconeri8
Oberonia falconeri7
Oberonia cavaleriei1
Oberonia cavaleriei3
Oberonia cavaleriei2
Oberonia caulescens7
Oberonia caulescens8
Oberonia caulescens6
Oberonia caulescens9
Oberonia caulescens11
Oberonia caulescens10
Oberonia caulescens13
Oberonia caulescens12
Oberonia caulescens1
Oberonia caulescens2
Oberonia caulescens5
Oberonia caulescens3
Oberonia caulescens4
Oberonia ensiformis11
Oberonia ensiformis9
Oberonia ensiformis10
Oberonia ensiformis4
Oberonia ensiformis5
Oberonia ensiformis6
Oberonia ensiformis7
Oberonia ensiformis8
Oberonia mucronata8
Oberonia mucronata5
Oberonia mucronata9
Oberonia mucronata6
Oberonia mucronata7
Oberonia mucronata1
Oberonia mucronata3

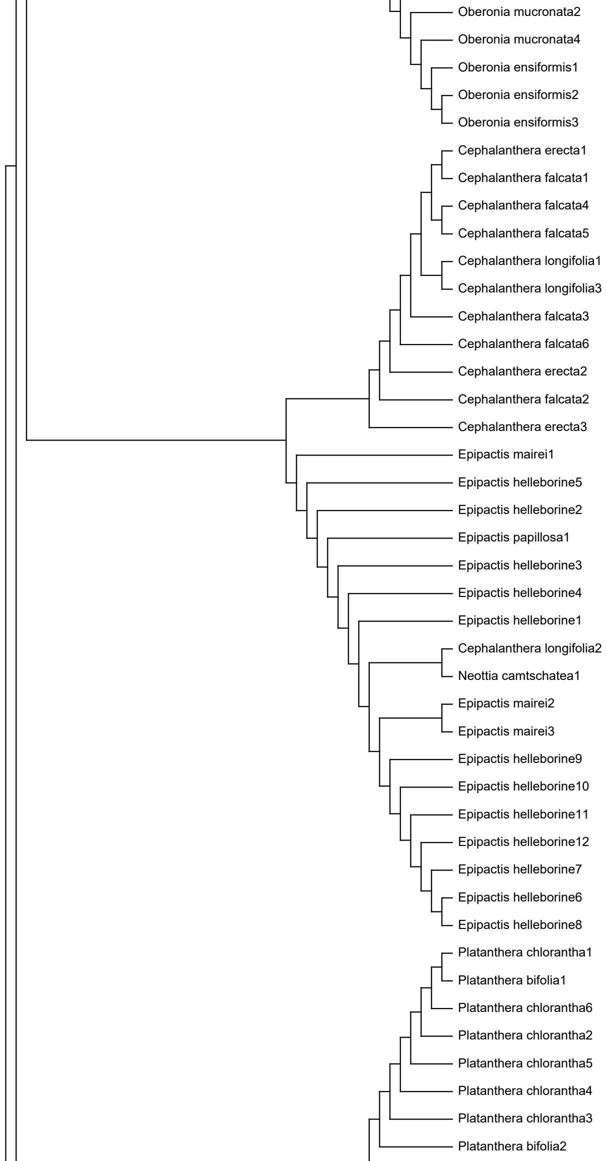
Oberonia mucronata2
Oberonia mucronata4
Oberonia ensiformis1
Oberonia ensiformis2
Oberonia ensiformis3
Cephalanthera erecta1
Cephalanthera falcata1
Cephalanthera falcata4
Cephalanthera falcata5
Cephalanthera longifolia1
Cephalanthera longifolia3
Cephalanthera falcata3
Cephalanthera falcata6
Cephalanthera erecta2
Cephalanthera falcata2
Cephalanthera erecta3
Epipactis mairei1
Epipactis helleborine5
Epipactis helleborine2
Epipactis papillosa1
Epipactis helleborine3
Epipactis helleborine4
Epipactis helleborine1
Cephalanthera longifolia2
Neottia camtschatea1
Epipactis mairei2
Epipactis mairei3
Epipactis helleborine9
Epipactis helleborine10
Epipactis helleborine11
Epipactis helleborine12
Epipactis helleborine7
Epipactis helleborine6
Epipactis helleborine8
Platanthera chlorantha1
Platanthera bifolia1
Platanthera chlorantha6
Platanthera chlorantha2
Platanthera chlorantha5
Platanthera chlorantha4
Platanthera chlorantha3
Platanthera bifolia2

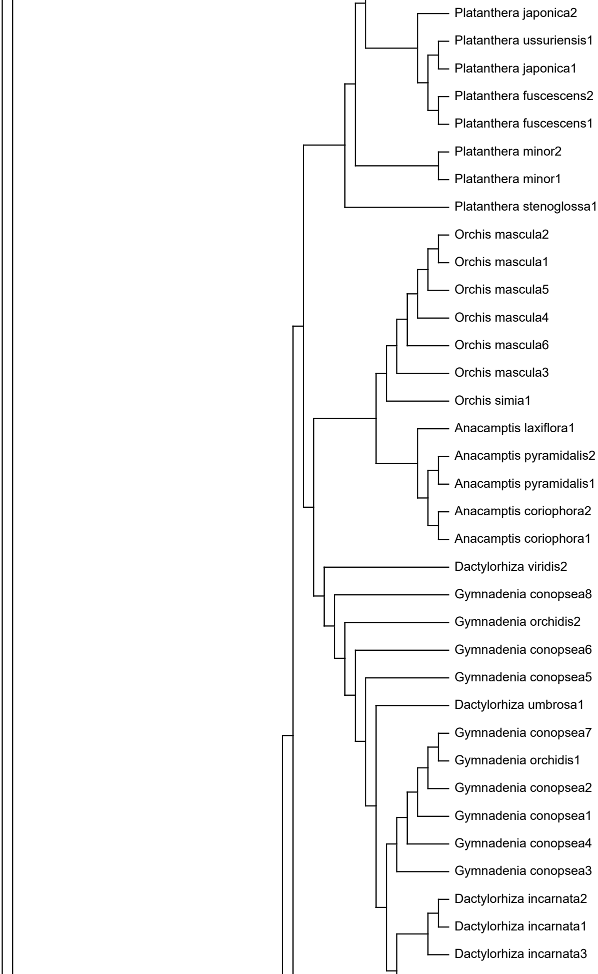

Platanthera japonica2
Platanthera ussuriensis1
Platanthera japonica1
Platanthera fuscescens2
Platanthera fuscescens1
Platanthera minor2
Platanthera minor1
Platanthera stenoglossa1
Orchis mascula2
Orchis mascula1
Orchis mascula5
Orchis mascula4
Orchis mascula6
Orchis mascula3
Orchis simia1
Anacamptis laxiflora1
Anacamptis pyramidalis2
Anacamptis pyramidalis1
Anacamptis coriophora2
Anacamptis coriophora1
Dactylorhiza viridis2
Gymnadenia conopsea8
Gymnadenia orchidis2
Gymnadenia conopsea6
Gymnadenia conopsea5
Dactylorhiza umbrosa1
Gymnadenia conopsea7
Gymnadenia orchidis1
Gymnadenia conopsea2
Gymnadenia conopsea1
Gymnadenia conopsea4
Gymnadenia conopsea3
Dactylorhiza incarnata2
Dactylorhiza incarnata1
Dactylorhiza incarnata3

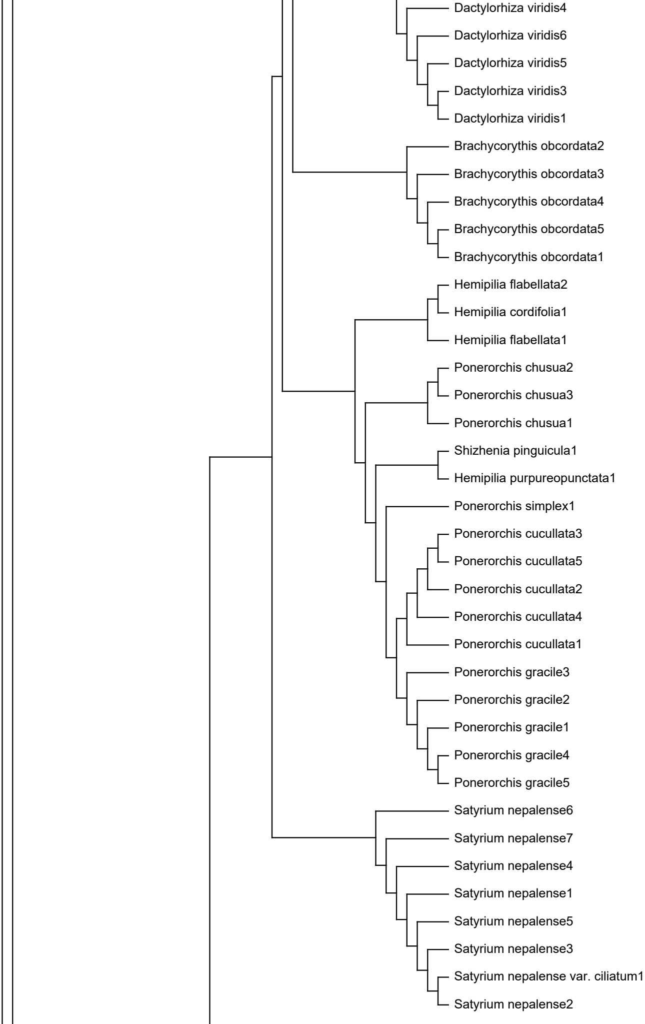
Dactylorhiza viridis4
Dactylorhiza viridis6
Dactylorhiza viridis5
Dactylorhiza viridis3
Dactylorhiza viridis1
Brachycorythis obcordata2
Brachycorythis obcordata3
Brachycorythis obcordata4
Brachycorythis obcordata5
Brachycorythis obcordata1
Hemipilia flabellata2
Hemipilia cordifolia1
Hemipilia flabellata1
Ponerorchis chusua2
Ponerorchis chusua3
Ponerorchis chusua1
Shizhenia pinguicula1
Hemipilia purpureopunctata1
Ponerorchis simplex1
Ponerorchis cucullata3
Ponerorchis cucullata5
Ponerorchis cucullata2
Ponerorchis cucullata4
Ponerorchis cucullata1
Ponerorchis gracile3
Ponerorchis gracile2
Ponerorchis gracile1
Ponerorchis gracile4
Ponerorchis gracile5
Satyrium nepalense6
Satyrium nepalense7
Satyrium nepalense4
Satyrium nepalense1
Satyrium nepalense5
Satyrium nepalense3
Satyrium nepalense var. ciliatum1
Satyrium nepalense2

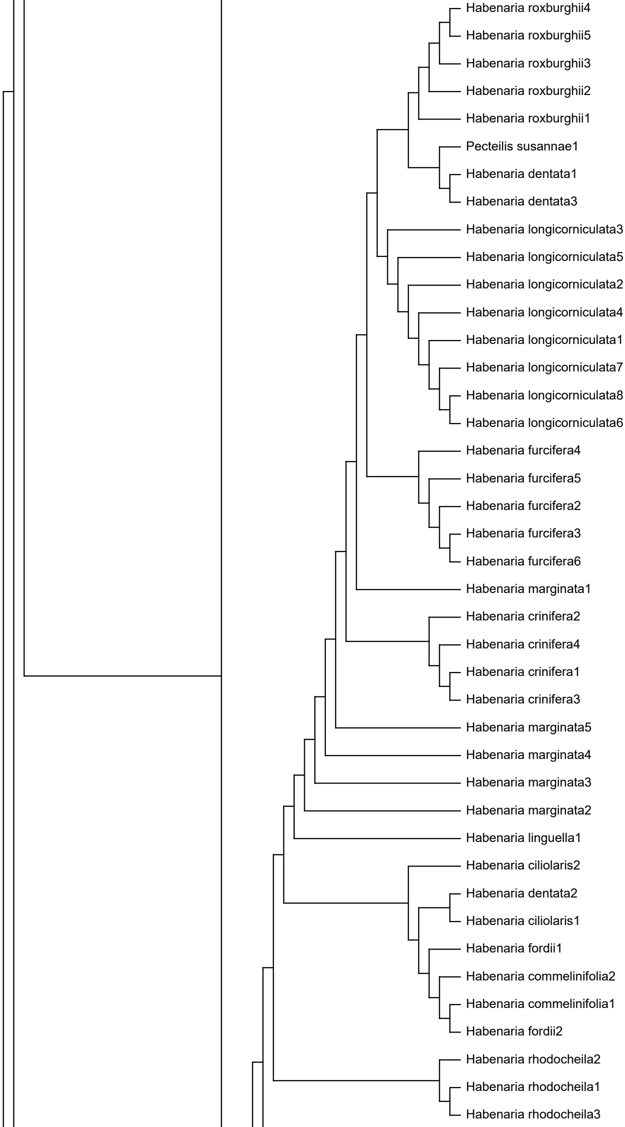
Habenaria roxburghii4
Habenaria roxburghii5
Habenaria roxburghii3
Habenaria roxburghii2
Habenaria roxburghii1
Pecteilis susannae1
Habenaria dentata1
Habenaria dentata3
Habenaria longicorniculata3
Habenaria longicorniculata5
Habenaria longicorniculata2
Habenaria longicorniculata4
Habenaria longicorniculata1
Habenaria longicorniculata7
Habenaria longicorniculata8
Habenaria longicorniculata6
Habenaria furcifera4
Habenaria furcifera5
Habenaria furcifera2
Habenaria furcifera3
Habenaria furcifera6
Habenaria marginata1
Habenaria crinifera2
Habenaria crinifera4
Habenaria crinifera1
Habenaria crinifera3
Habenaria marginata5
Habenaria marginata4
Habenaria marginata3
Habenaria marginata2
Habenaria linguella1
Habenaria ciliolaris2
Habenaria dentata2
Habenaria ciliolaris1
Habenaria fordii1
Habenaria commelinifolia2
Habenaria commelinifolia1
Habenaria fordii2
Habenaria rhodocheila2
Habenaria rhodocheila1
Habenaria rhodocheila3

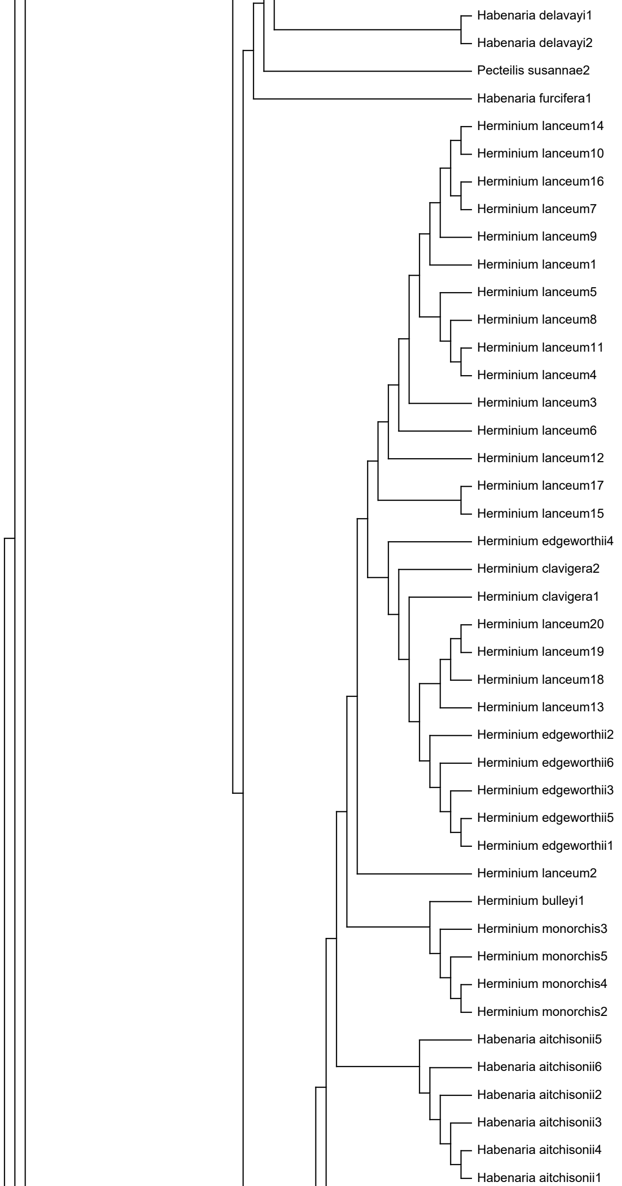
Habenaria delavayi1
Habenaria delavayi2
Pecteilis susannae2
Habenaria furcifera1
Herminium lanceum14
Herminium lanceum10
Herminium lanceum16
Herminium lanceum7
Herminium lanceum9
Herminium lanceum1
Herminium lanceum5
Herminium lanceum8
Herminium lanceum11
Herminium lanceum4
Herminium lanceum3
Herminium lanceum6
Herminium lanceum12
Herminium lanceum17
Herminium lanceum15
Herminium edgeworthii4
Herminium clavigera2
Herminium clavigera1
Herminium lanceum20
Herminium lanceum19
Herminium lanceum18
Herminium lanceum13
Herminium edgeworthii2
Herminium edgeworthii6
Herminium edgeworthii3
Herminium edgeworthii5
Herminium edgeworthii1
Herminium lanceum2
Herminium bulleyi1
Herminium monorchis3
Herminium monorchis5
Herminium monorchis4
Herminium monorchis2
Habenaria aitchisonii5
Habenaria aitchisonii6
Habenaria aitchisonii2
Habenaria aitchisonii3
Habenaria aitchisonii4
Habenaria aitchisonii1

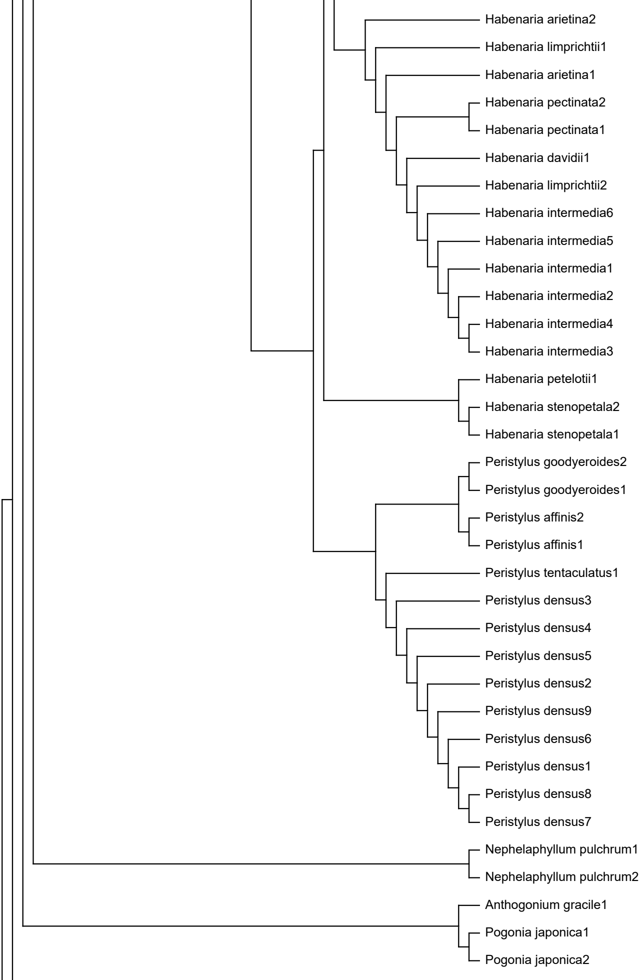
Habenaria arietina2
Habenaria limprichtii1
Habenaria arietina1
Habenaria pectinata2
Habenaria pectinata1
Habenaria davidii1
Habenaria limprichtii2
Habenaria intermedia6
Habenaria intermedia5
Habenaria intermedia1
Habenaria intermedia2
Habenaria intermedia4
Habenaria intermedia3
Habenaria petelotii1
Habenaria stenopetala2
Habenaria stenopetala1
Peristylus goodyeroides2
Peristylus goodyeroides1
Peristylus affinis2
Peristylus affinis1
Peristylus tentaculatus1
Peristylus densus3
Peristylus densus4
Peristylus densus5
Peristylus densus2
Peristylus densus9
Peristylus densus6
Peristylus densus1
Peristylus densus8
Peristylus densus7
Nephelaphyllum pulchrum1
Nephelaphyllum pulchrum2
Anthogonium gracile1
Pogonia japonica1
Pogonia japonica2

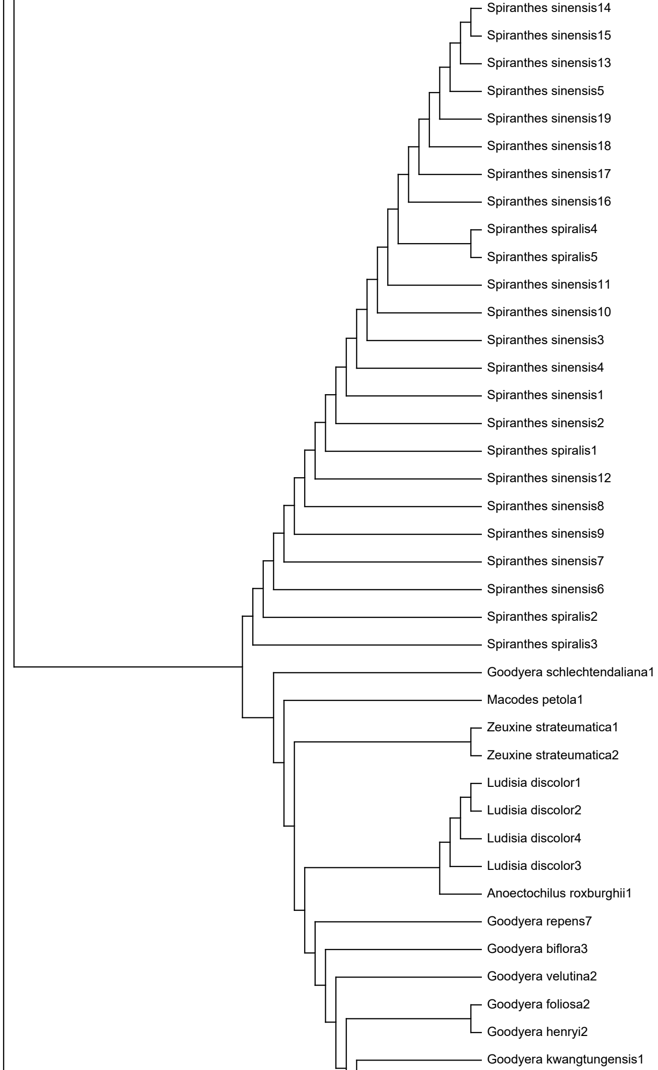
Spiranthes sinensis14
Spiranthes sinensis15
Spiranthes sinensis13
Spiranthes sinensis5
Spiranthes sinensis19
Spiranthes sinensis18
Spiranthes sinensis17
Spiranthes sinensis16
Spiranthes spiralis4
Spiranthes spiralis5
Spiranthes sinensis11
Spiranthes sinensis10
Spiranthes sinensis3
Spiranthes sinensis4
Spiranthes sinensis1
Spiranthes sinensis2
Spiranthes spiralis1
Spiranthes sinensis12
Spiranthes sinensis8
Spiranthes sinensis9
Spiranthes sinensis7
Spiranthes sinensis6
Spiranthes spiralis2
Spiranthes spiralis3
Goodyera schlechtendaliana1
Macodes petola1
Zeuxine strateumatica1
Zeuxine strateumatica2
Ludisia discolor1
Ludisia discolor2
Ludisia discolor4
Ludisia discolor3
Anoectochilus roxburghii1
Goodyera repens7
Goodyera biflora3
Goodyera velutina2
Goodyera foliosa2
Goodyera henryi2
Goodyera kwangtungensis1

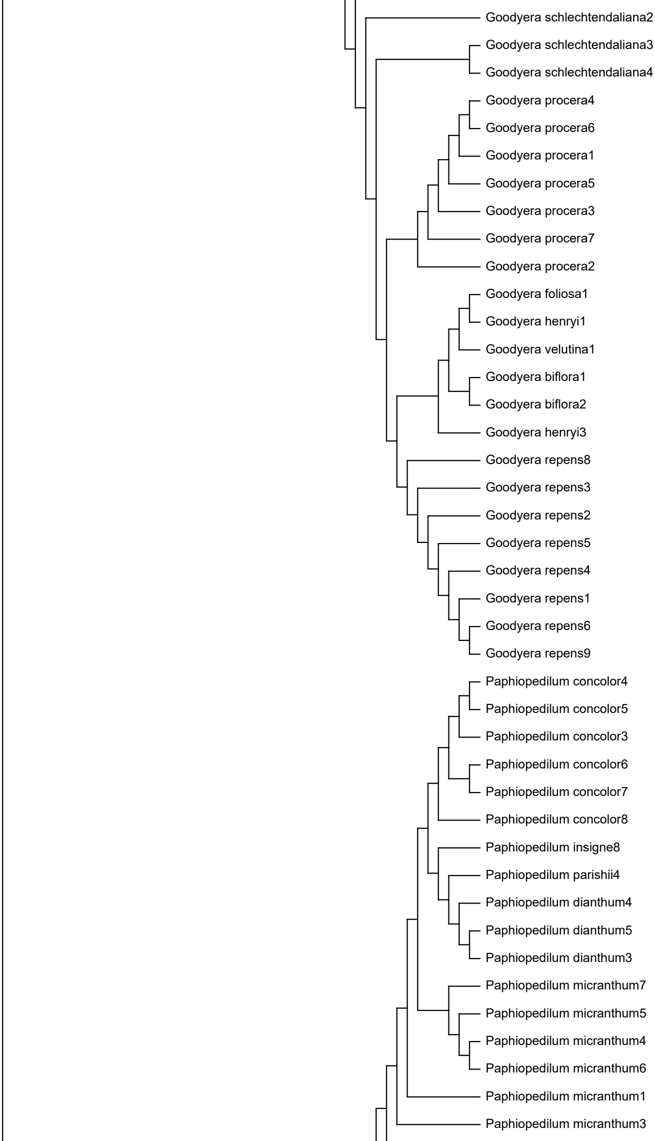
Goodyera schlechtendaliana2
Goodyera schlechtendaliana3
Goodyera schlechtendaliana4
Goodyera procera4
Goodyera procera6
Goodyera procera1
Goodyera procera5
Goodyera procera3
Goodyera procera7
Goodyera procera2
Goodyera foliosa1
Goodyera henryi1
Goodyera velutina1
Goodyera biflora1
Goodyera biflora2
Goodyera henryi3
Goodyera repens8
Goodyera repens3
Goodyera repens2
Goodyera repens5
Goodyera repens4
Goodyera repens1
Goodyera repens6
Goodyera repens9
Paphiopedilum concolor4
Paphiopedilum concolor5
Paphiopedilum concolor3
Paphiopedilum concolor6
Paphiopedilum concolor7
Paphiopedilum concolor8
Paphiopedilum insigne8
Paphiopedilum parishii4
Paphiopedilum dianthum4
Paphiopedilum dianthum5
Paphiopedilum dianthum3
Paphiopedilum micranthum7
Paphiopedilum micranthum5
Paphiopedilum micranthum4
Paphiopedilum micranthum6
Paphiopedilum micranthum1
Paphiopedilum micranthum3

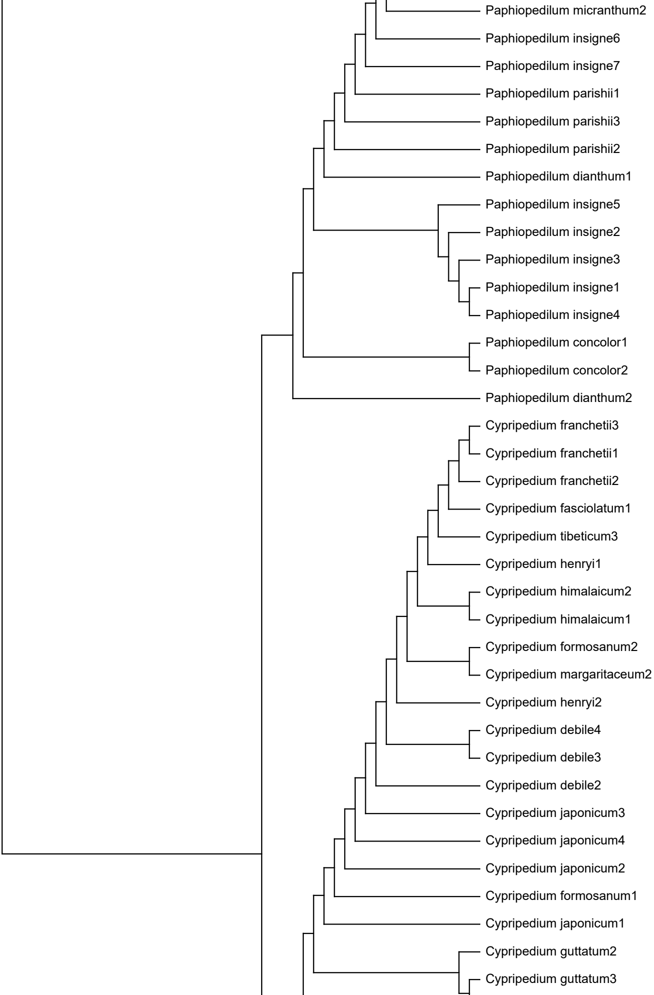
Paphiopedilum micranthum2
Paphiopedilum insigne6
Paphiopedilum insigne7
Paphiopedilum parishii1
Paphiopedilum parishii3
Paphiopedilum parishii2
Paphiopedilum dianthum1
Paphiopedilum insigne5
Paphiopedilum insigne2
Paphiopedilum insigne3
Paphiopedilum insigne1
Paphiopedilum insigne4
Paphiopedilum concolor1
Paphiopedilum concolor2
Paphiopedilum dianthum2
Cypripedium franchetii3
Cypripedium franchetii1
Cypripedium franchetii2
Cypripedium fasciolatum1
Cypripedium tibeticum3
Cypripedium henryi1
Cypripedium himalaicum2
Cypripedium himalaicum1
Cypripedium formosanum2
Cypripedium margaritaceum2
Cypripedium henryi2
Cypripedium debile4
Cypripedium debile3
Cypripedium debile2
Cypripedium japonicum3
Cypripedium japonicum4
Cypripedium japonicum2
Cypripedium formosanum1
Cypripedium japonicum1
Cypripedium guttatum2
Cypripedium guttatum3

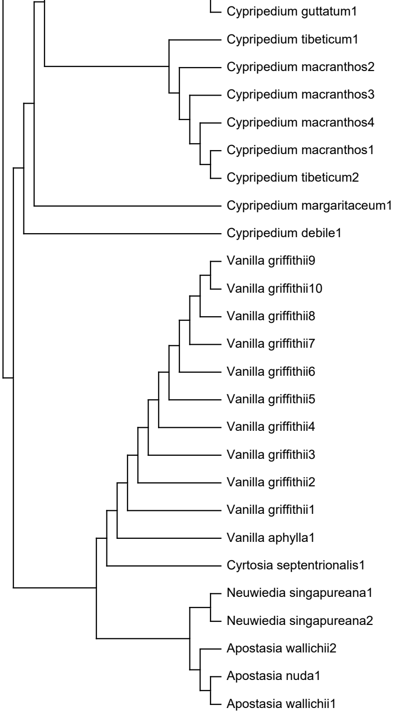
Cypripedium guttatum1
Cypripedium tibeticum1
Cypripedium macranthos2
Cypripedium macranthos3
Cypripedium macranthos4
Cypripedium macranthos1
Cypripedium tibeticum2
Cypripedium margaritaceum1
Cypripedium debile1
Vanilla griffithii9
Vanilla griffithii10
Vanilla griffithii8
Vanilla griffithii7
Vanilla griffithii6
Vanilla griffithii5
Vanilla griffithii4
Vanilla griffithii3
Vanilla griffithii2
Vanilla griffithii1
Vanilla aphylla1
Cyrtosia septentrionalis1
Neuwiedia singapureana1
Neuwiedia singapureana2
Apostasia wallichii2
Apostasia nuda1
Apostasia wallichii1
