# Supplementary material for: DNA barcoding of medicinal orchids in Asia
Source: Sci Rep. 2021 Dec 8;11:23651. doi: 10.1038/s41598-021-03025-0 (PMC8654824; doi:10.1038/s41598-021-03025-0)
Supplement: Supplementary file 12 — Supplementary Figure S12. [file 41598_2021_3025_MOESM12_ESM.pdf]

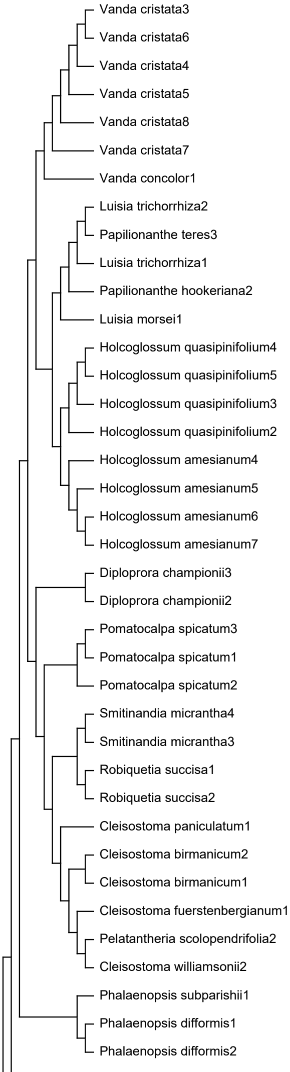
Vanda cristata3
Vanda cristata6
Vanda cristata4
Vanda cristata5
Vanda cristata8
Vanda cristata7
Vanda concolor1
Luisia trichorrhiza2
Papilionanthe teres3
Luisia trichorrhiza1
Papilionanthe hookeriana2
Luisia morsei1
Holcoglossum quasipinifolium4
Holcoglossum quasipinifolium5
Holcoglossum quasipinifolium3
Holcoglossum quasipinifolium2
Holcoglossum amesianum4
Holcoglossum amesianum5
Holcoglossum amesianum6
Holcoglossum amesianum7
Diploprora championii3
Diploprora championii2
Pomatocalpa spicatum3
Pomatocalpa spicatum1
Pomatocalpa spicatum2
Smitinandia micrantha4
Smitinandia micrantha3
Robiquetia succisa1
Robiquetia succisa2
Cleisostoma paniculatum1
Cleisostoma birmanicum2
Cleisostoma birmanicum1
Cleisostoma fuerstenbergianum1
Pelatantheria scolopendrifolia2
Cleisostoma williamsonii2
Phalaenopsis subparishii1
Phalaenopsis difformis1
Phalaenopsis difformis2

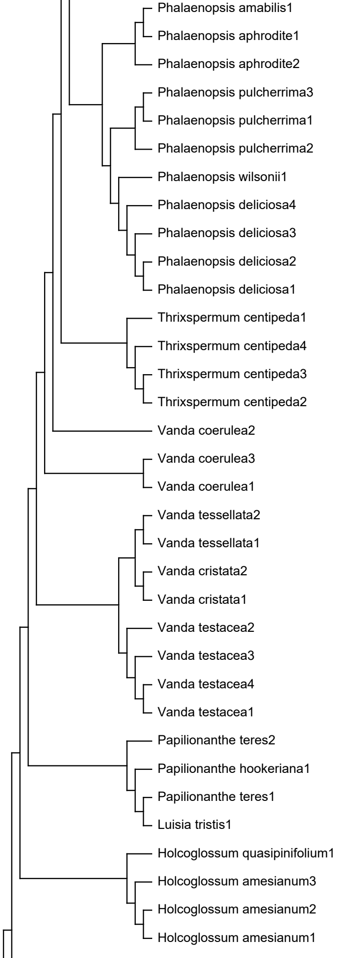
Phalaenopsis amabilis1
Phalaenopsis aphrodite1
Phalaenopsis aphrodite2
Phalaenopsis pulcherrima3
Phalaenopsis pulcherrima1
Phalaenopsis pulcherrima2
Phalaenopsis wilsonii1
Phalaenopsis deliciosa4
Phalaenopsis deliciosa3
Phalaenopsis deliciosa2
Phalaenopsis deliciosa1
Thrixspermum centipeda1
Thrixspermum centipeda4
Thrixspermum centipeda3
Thrixspermum centipeda2
Vanda coerulea2
Vanda coerulea3
Vanda coerulea1
Vanda tessellata2
Vanda tessellata1
Vanda cristata2
Vanda cristata1
Vanda testacea2
Vanda testacea3
Vanda testacea4
Vanda testacea1
Papilionanthe teres2
Papilionanthe hookeriana1
Papilionanthe teres1
Luisia tristis1
Holcoglossum quasipinifolium1
Holcoglossum amesianum3
Holcoglossum amesianum2
Holcoglossum amesianum1

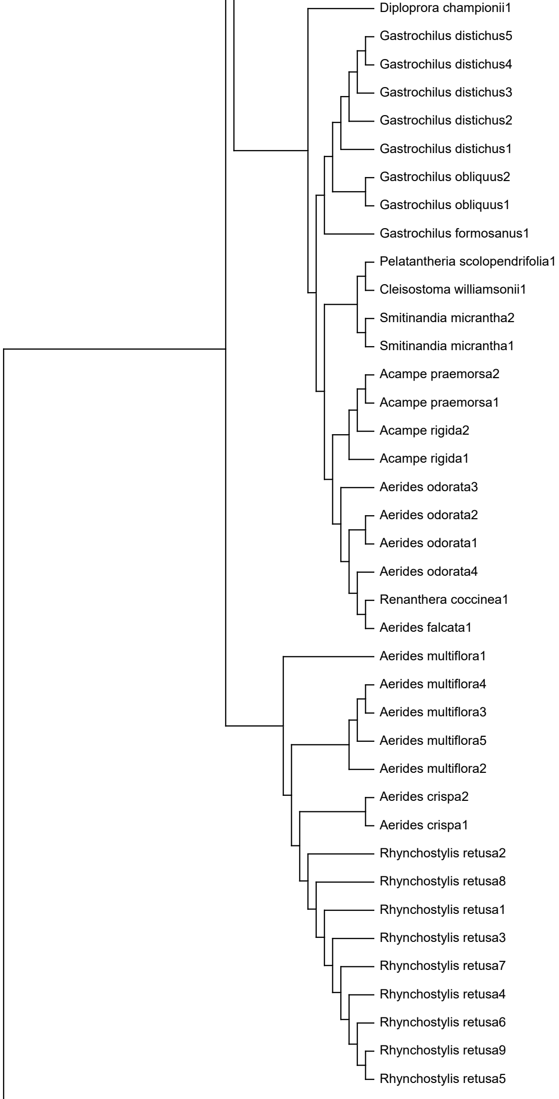
Diploprora championii1
Gastrochilus distichus5
Gastrochilus distichus4
Gastrochilus distichus3
Gastrochilus distichus2
Gastrochilus distichus1
Gastrochilus obliquus2
Gastrochilus obliquus1
Gastrochilus formosanus1
Pelatantheria scolopendrifolia1
Cleisostoma williamsonii1
Smitinandia micrantha2
Smitinandia micrantha1
Acampe praemorsa2
Acampe praemorsa1
Acampe rigida2
Acampe rigida1
Aerides odorata3
Aerides odorata2
Aerides odorata1
Aerides odorata4
Renanthera coccinea1
Aerides falcata1
Aerides multiflora1
Aerides multiflora4
Aerides multiflora3
Aerides multiflora5
Aerides multiflora2
Aerides crispa2
Aerides crispa1
Rhynchostylis retusa2
Rhynchostylis retusa8
Rhynchostylis retusa1
Rhynchostylis retusa3
Rhynchostylis retusa7
Rhynchostylis retusa4
Rhynchostylis retusa6
Rhynchostylis retusa9
Rhynchostylis retusa5

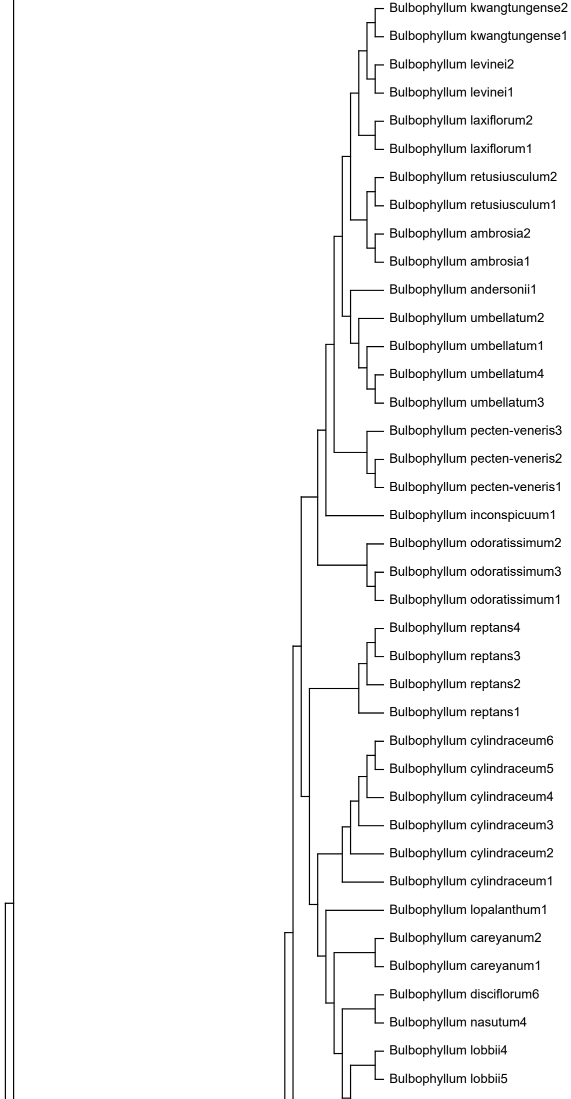
Bulbophyllum kwangtungense2
Bulbophyllum kwangtungense1
Bulbophyllum levinei2
Bulbophyllum levinei1
Bulbophyllum laxiflorum2
Bulbophyllum laxiflorum1
Bulbophyllum retusiusculum2
Bulbophyllum retusiusculum1
Bulbophyllum ambrosia2
Bulbophyllum ambrosia1
Bulbophyllum andersonii1
Bulbophyllum umbellatum2
Bulbophyllum umbellatum1
Bulbophyllum umbellatum4
Bulbophyllum umbellatum3
Bulbophyllum pecten-veneris3
Bulbophyllum pecten-veneris2
Bulbophyllum pecten-veneris1
Bulbophyllum inconspicuum1
Bulbophyllum odoratissimum2
Bulbophyllum odoratissimum3
Bulbophyllum odoratissimum1
Bulbophyllum reptans4
Bulbophyllum reptans3
Bulbophyllum reptans2
Bulbophyllum reptans1
Bulbophyllum cylindraceum6
Bulbophyllum cylindraceum5
Bulbophyllum cylindraceum4
Bulbophyllum cylindraceum3
Bulbophyllum cylindraceum2
Bulbophyllum cylindraceum1
Bulbophyllum lopalanthum1
Bulbophyllum careyanum2
Bulbophyllum careyanum1
Bulbophyllum disciflorum6
Bulbophyllum nasutum4
Bulbophyllum lobbii4
Bulbophyllum lobbii5

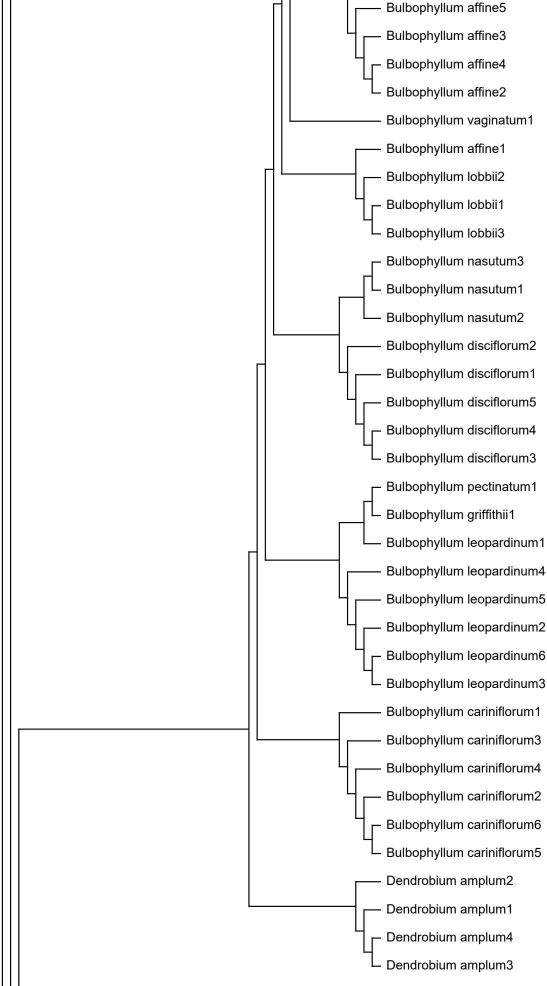

Bulbophyllum affine5
Bulbophyllum affine3
Bulbophyllum affine4
Bulbophyllum affine2
Bulbophyllum vaginatum1
Bulbophyllum affine1
Bulbophyllum lobbii2
Bulbophyllum lobbii1
Bulbophyllum lobbii3
Bulbophyllum nasutum3
Bulbophyllum nasutum1
Bulbophyllum nasutum2
Bulbophyllum disciflorum2
Bulbophyllum disciflorum1
Bulbophyllum disciflorum5
Bulbophyllum disciflorum4
Bulbophyllum disciflorum3
Bulbophyllum pectinatum1
Bulbophyllum griffithii1
Bulbophyllum leopardinum1
Bulbophyllum leopardinum4
Bulbophyllum leopardinum5
Bulbophyllum leopardinum2
Bulbophyllum leopardinum6
Bulbophyllum leopardinum3
Bulbophyllum cariniflorum1
Bulbophyllum cariniflorum3
Bulbophyllum cariniflorum4
Bulbophyllum cariniflorum2
Bulbophyllum cariniflorum6
Bulbophyllum cariniflorum5
Dendrobium amplum2
Dendrobium amplum1
Dendrobium amplum4
Dendrobium amplum3

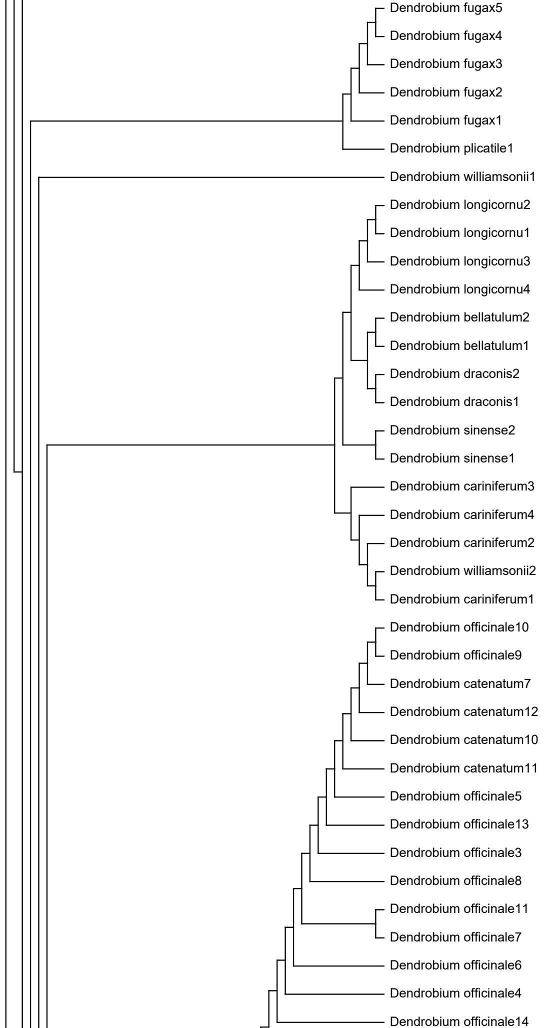
Dendrobium fugax5
Dendrobium fugax4
Dendrobium fugax3
Dendrobium fugax2
Dendrobium fugax1
Dendrobium plicatile1
Dendrobium williamsonii1
Dendrobium longicornu2
Dendrobium longicornu1
Dendrobium longicornu3
Dendrobium longicornu4
Dendrobium bellatulum2
Dendrobium bellatulum1
Dendrobium draconis2
Dendrobium draconis1
Dendrobium sinense2
Dendrobium sinense1
Dendrobium cariniferum3
Dendrobium cariniferum4
Dendrobium cariniferum2
Dendrobium williamsonii2
Dendrobium cariniferum1
Dendrobium officinale10
Dendrobium officinale9
Dendrobium catenatum7
Dendrobium catenatum12
Dendrobium catenatum10
Dendrobium catenatum11
Dendrobium officinale5
Dendrobium officinale13
Dendrobium officinale3
Dendrobium officinale8
Dendrobium officinale11
Dendrobium officinale7
Dendrobium officinale6
Dendrobium officinale4
Dendrobium officinale14

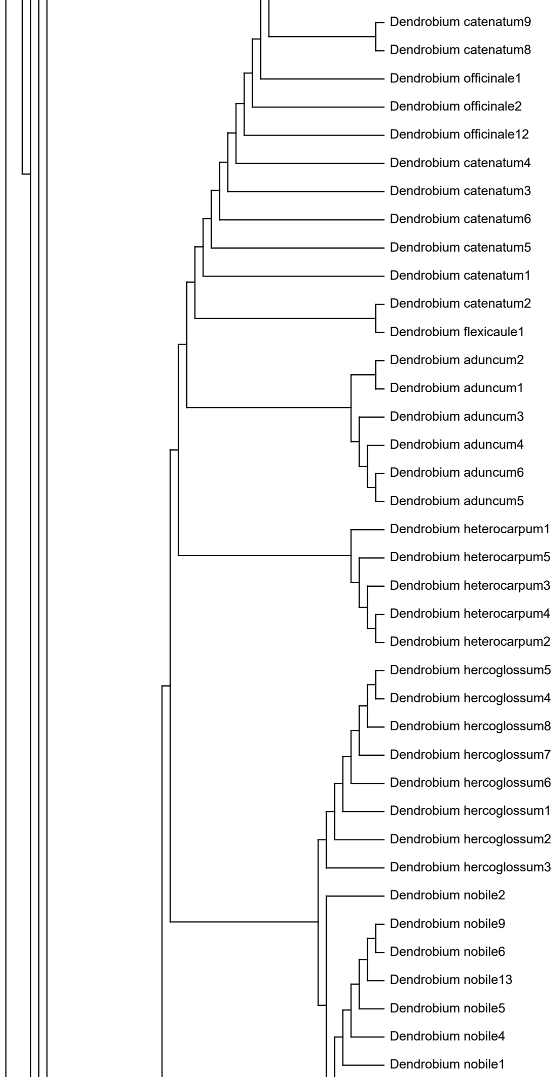
Dendrobium catenatum9
Dendrobium catenatum8
Dendrobium officinale1
Dendrobium officinale2
Dendrobium officinale12
Dendrobium catenatum4
Dendrobium catenatum3
Dendrobium catenatum6
Dendrobium catenatum5
Dendrobium catenatum1
Dendrobium catenatum2
Dendrobium flexicaule1
Dendrobium aduncum2
Dendrobium aduncum1
Dendrobium aduncum3
Dendrobium aduncum4
Dendrobium aduncum6
Dendrobium aduncum5
Dendrobium heterocarpum1
Dendrobium heterocarpum5
Dendrobium heterocarpum3
Dendrobium heterocarpum4
Dendrobium heterocarpum2
Dendrobium hercoglossum5
Dendrobium hercoglossum4
Dendrobium hercoglossum8
Dendrobium hercoglossum7
Dendrobium hercoglossum6
Dendrobium hercoglossum1
Dendrobium hercoglossum2
Dendrobium hercoglossum3
Dendrobium nobile2
Dendrobium nobile9
Dendrobium nobile6
Dendrobium nobile13
Dendrobium nobile5
Dendrobium nobile4
Dendrobium nobile1

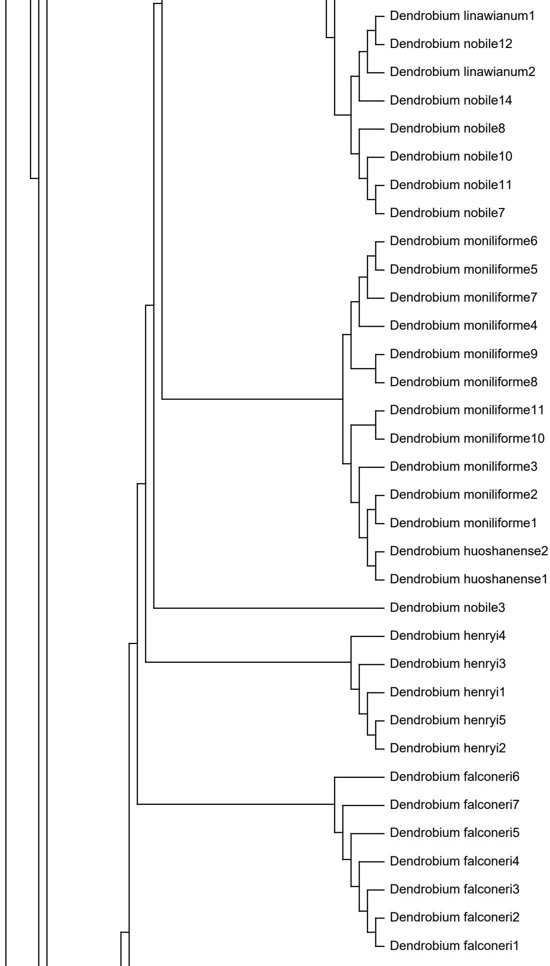

Dendrobium linawianum1
Dendrobium nobile12
Dendrobium linawianum2
Dendrobium nobile14
Dendrobium nobile8
Dendrobium nobile10
Dendrobium nobile11
Dendrobium nobile7
Dendrobium moniliforme6
Dendrobium moniliforme5
Dendrobium moniliforme7
Dendrobium moniliforme4
Dendrobium moniliforme9
Dendrobium moniliforme8
Dendrobium moniliforme11
Dendrobium moniliforme10
Dendrobium moniliforme3
Dendrobium moniliforme2
Dendrobium moniliforme1
Dendrobium huoshanense2
Dendrobium huoshanense1
Dendrobium nobile3
Dendrobium henryi4
Dendrobium henryi3
Dendrobium henryi1
Dendrobium henryi5
Dendrobium henryi2
Dendrobium falconeri6
Dendrobium falconeri7
Dendrobium falconeri5
Dendrobium falconeri4
Dendrobium falconeri3
Dendrobium falconeri2
Dendrobium falconeri1

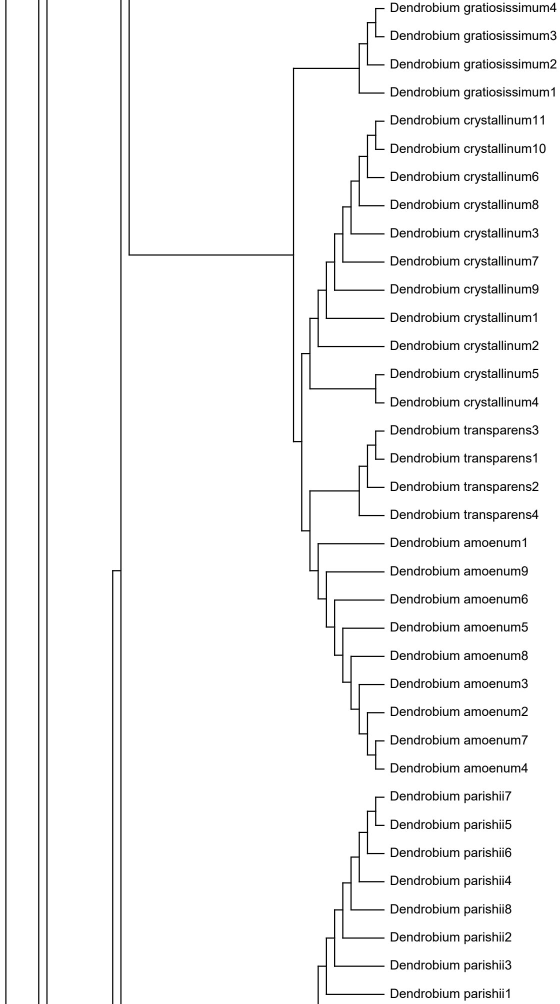

Dendrobium gratiosissimum4
Dendrobium gratiosissimum3
Dendrobium gratiosissimum2
Dendrobium gratiosissimum1
Dendrobium crystallinum11
Dendrobium crystallinum10
Dendrobium crystallinum6
Dendrobium crystallinum8
Dendrobium crystallinum3
Dendrobium crystallinum7
Dendrobium crystallinum9
Dendrobium crystallinum1
Dendrobium crystallinum2
Dendrobium crystallinum5
Dendrobium crystallinum4
Dendrobium transparens3
Dendrobium transparens1
Dendrobium transparens2
Dendrobium transparens4
Dendrobium amoenum1
Dendrobium amoenum9
Dendrobium amoenum6
Dendrobium amoenum5
Dendrobium amoenum8
Dendrobium amoenum3
Dendrobium amoenum2
Dendrobium amoenum7
Dendrobium amoenum4
Dendrobium parishii7
Dendrobium parishii5
Dendrobium parishii6
Dendrobium parishii4
Dendrobium parishii8
Dendrobium parishii2
Dendrobium parishii3
Dendrobium parishii1

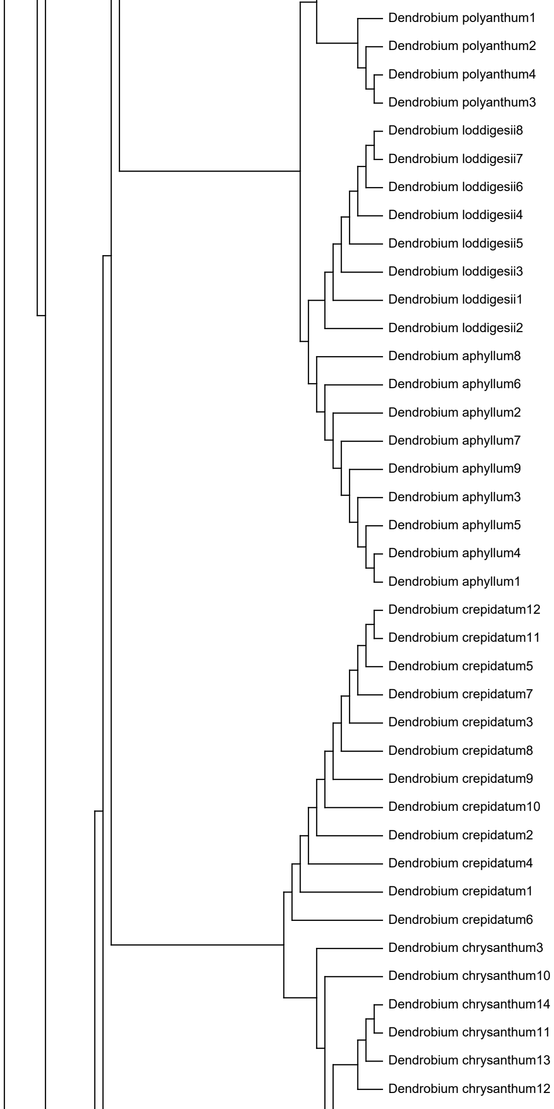

Dendrobium polyanthum1
Dendrobium polyanthum2
Dendrobium polyanthum4
Dendrobium polyanthum3
Dendrobium loddigesii8
Dendrobium loddigesii7
Dendrobium loddigesii6
Dendrobium loddigesii4
Dendrobium loddigesii5
Dendrobium loddigesii3
Dendrobium loddigesii1
Dendrobium loddigesii2
Dendrobium aphyllum8
Dendrobium aphyllum6
Dendrobium aphyllum2
Dendrobium aphyllum7
Dendrobium aphyllum9
Dendrobium aphyllum3
Dendrobium aphyllum5
Dendrobium aphyllum4
Dendrobium aphyllum1
Dendrobium crepidatum12
Dendrobium crepidatum11
Dendrobium crepidatum5
Dendrobium crepidatum7
Dendrobium crepidatum3
Dendrobium crepidatum8
Dendrobium crepidatum9
Dendrobium crepidatum10
Dendrobium crepidatum2
Dendrobium crepidatum4
Dendrobium crepidatum1
Dendrobium crepidatum6
Dendrobium chrysanthum3
Dendrobium chrysanthum10
Dendrobium chrysanthum14
Dendrobium chrysanthum11
Dendrobium chrysanthum13
Dendrobium chrysanthum12

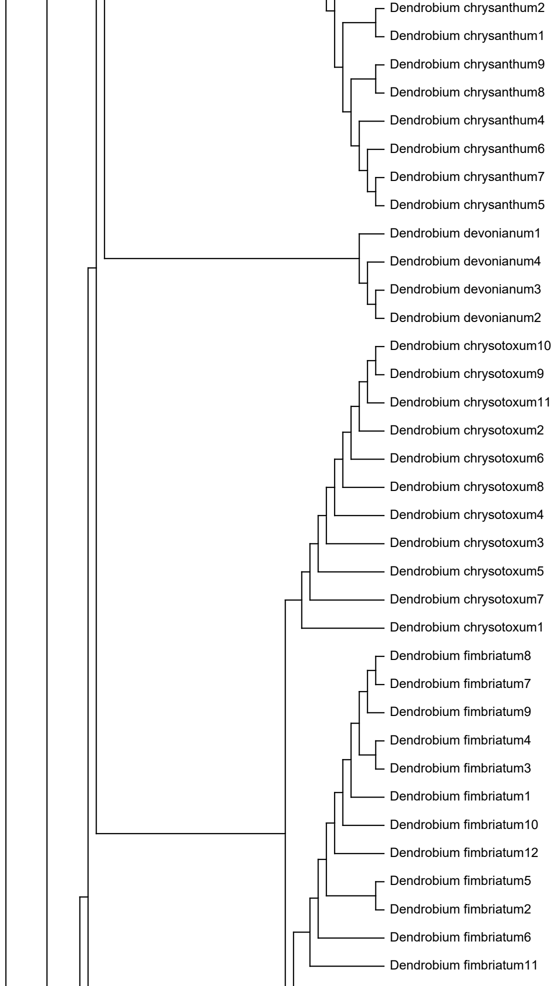
Dendrobium chrysanthum2
Dendrobium chrysanthum1
Dendrobium chrysanthum9
Dendrobium chrysanthum8
Dendrobium chrysanthum4
Dendrobium chrysanthum6
Dendrobium chrysanthum7
Dendrobium chrysanthum5
Dendrobium devonianum1
Dendrobium devonianum4
Dendrobium devonianum3
Dendrobium devonianum2
Dendrobium chrysotoxum10
Dendrobium chrysotoxum9
Dendrobium chrysotoxum11
Dendrobium chrysotoxum2
Dendrobium chrysotoxum6
Dendrobium chrysotoxum8
Dendrobium chrysotoxum4
Dendrobium chrysotoxum3
Dendrobium chrysotoxum5
Dendrobium chrysotoxum7
Dendrobium chrysotoxum1
Dendrobium fimbriatum8
Dendrobium fimbriatum7
Dendrobium fimbriatum9
Dendrobium fimbriatum4
Dendrobium fimbriatum3
Dendrobium fimbriatum1
Dendrobium fimbriatum10
Dendrobium fimbriatum12
Dendrobium fimbriatum5
Dendrobium fimbriatum2
Dendrobium fimbriatum6
Dendrobium fimbriatum11

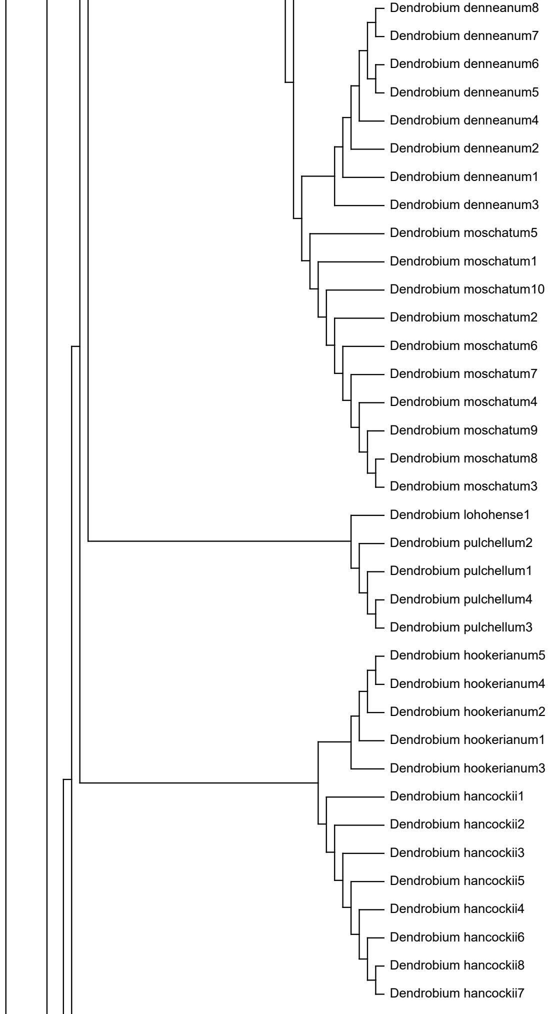
Dendrobium denneanum8
Dendrobium denneanum7
Dendrobium denneanum6
Dendrobium denneanum5
Dendrobium denneanum4
Dendrobium denneanum2
Dendrobium denneanum1
Dendrobium denneanum3
Dendrobium moschatum5
Dendrobium moschatum1
Dendrobium moschatum10
Dendrobium moschatum2
Dendrobium moschatum6
Dendrobium moschatum7
Dendrobium moschatum4
Dendrobium moschatum9
Dendrobium moschatum8
Dendrobium moschatum3
Dendrobium lohohense1
Dendrobium pulchellum2
Dendrobium pulchellum1
Dendrobium pulchellum4
Dendrobium pulchellum3
Dendrobium hookerianum5
Dendrobium hookerianum4
Dendrobium hookerianum2
Dendrobium hookerianum1
Dendrobium hookerianum3
Dendrobium hancockii1
Dendrobium hancockii2
Dendrobium hancockii3
Dendrobium hancockii5
Dendrobium hancockii4
Dendrobium hancockii6
Dendrobium hancockii8
Dendrobium hancockii7

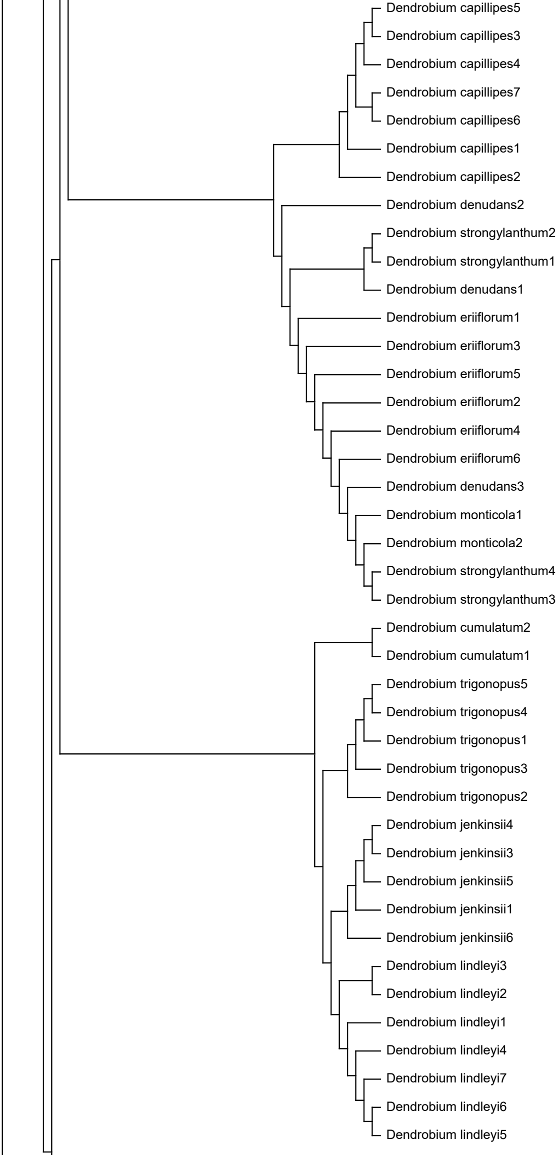
Dendrobium capillipes5
Dendrobium capillipes3
Dendrobium capillipes4
Dendrobium capillipes7
Dendrobium capillipes6
Dendrobium capillipes1
Dendrobium capillipes2
Dendrobium denudans2
Dendrobium strongylanthum2
Dendrobium strongylanthum1
Dendrobium denudans1
Dendrobium eriiflorum1
Dendrobium eriiflorum3
Dendrobium eriiflorum5
Dendrobium eriiflorum2
Dendrobium eriiflorum4
Dendrobium eriiflorum6
Dendrobium denudans3
Dendrobium monticola1
Dendrobium monticola2
Dendrobium strongylanthum4
Dendrobium strongylanthum3
Dendrobium cumulatum2
Dendrobium cumulatum1
Dendrobium trigonopus5
Dendrobium trigonopus4
Dendrobium trigonopus1
Dendrobium trigonopus3
Dendrobium trigonopus2
Dendrobium jenkinsii4
Dendrobium jenkinsii3
Dendrobium jenkinsii5
Dendrobium jenkinsii1
Dendrobium jenkinsii6
Dendrobium lindleyi3
Dendrobium lindleyi2
Dendrobium lindleyi1
Dendrobium lindleyi4
Dendrobium lindleyi7
Dendrobium lindleyi6
Dendrobium lindleyi5

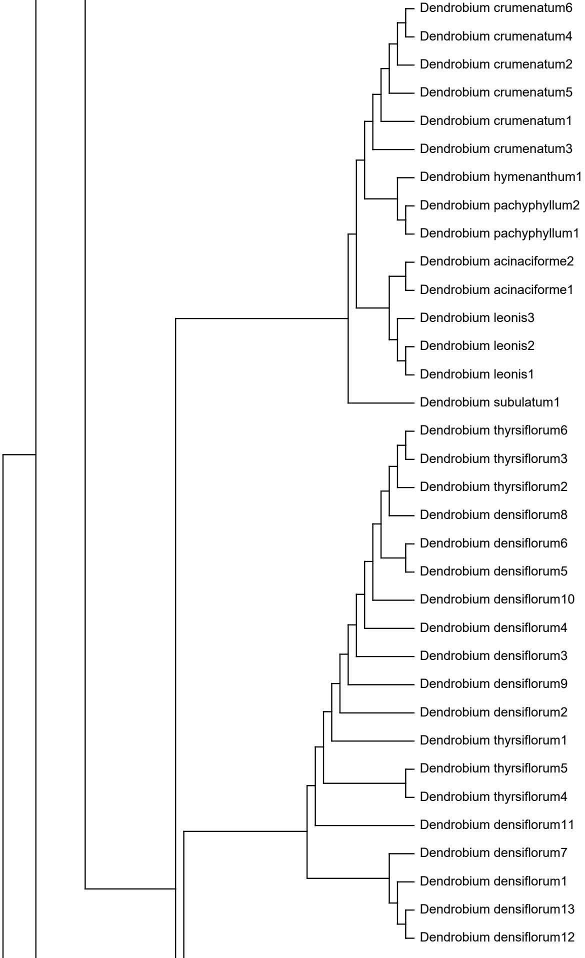
Dendrobium crumenatum6
Dendrobium crumenatum4
Dendrobium crumenatum2
Dendrobium crumenatum5
Dendrobium crumenatum1
Dendrobium crumenatum3
Dendrobium hymenanthum1
Dendrobium pachyphyllum2
Dendrobium pachyphyllum1
Dendrobium acinaciforme2
Dendrobium acinaciforme1
Dendrobium leonis3
Dendrobium leonis2
Dendrobium leonis1
Dendrobium subulatum1
Dendrobium thyrsiflorum6
Dendrobium thyrsiflorum3
Dendrobium thyrsiflorum2
Dendrobium densiflorum8
Dendrobium densiflorum6
Dendrobium densiflorum5
Dendrobium densiflorum10
Dendrobium densiflorum4
Dendrobium densiflorum3
Dendrobium densiflorum9
Dendrobium densiflorum2
Dendrobium thyrsiflorum1
Dendrobium thyrsiflorum5
Dendrobium thyrsiflorum4
Dendrobium densiflorum11
Dendrobium densiflorum7
Dendrobium densiflorum1
Dendrobium densiflorum13
Dendrobium densiflorum12

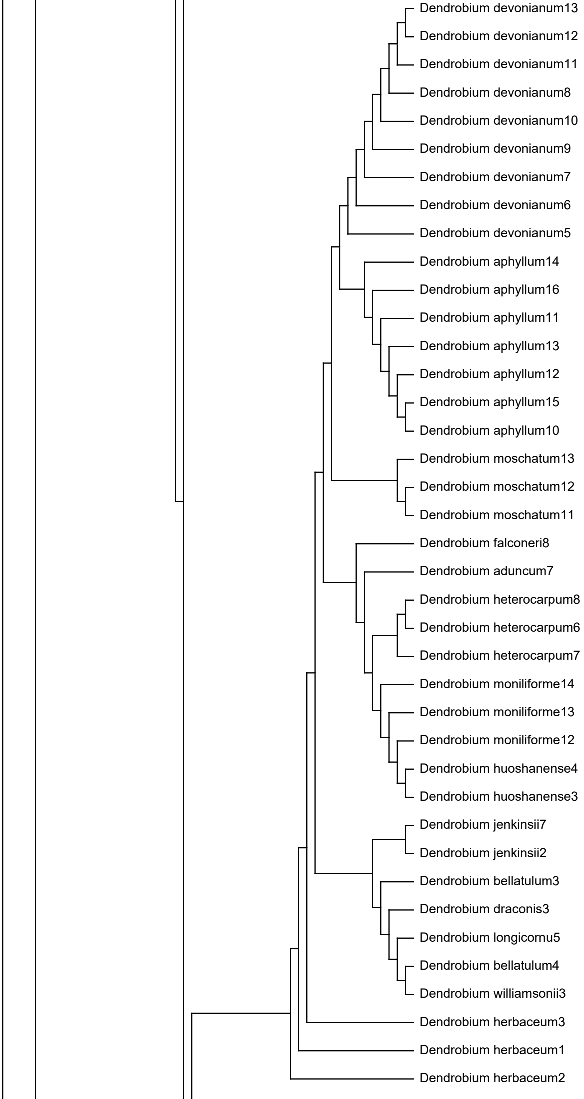
Dendrobium devonianum13
Dendrobium devonianum12
Dendrobium devonianum11
Dendrobium devonianum8
Dendrobium devonianum10
Dendrobium devonianum9
Dendrobium devonianum7
Dendrobium devonianum6
Dendrobium devonianum5
Dendrobium aphyllum14
Dendrobium aphyllum16
Dendrobium aphyllum11
Dendrobium aphyllum13
Dendrobium aphyllum12
Dendrobium aphyllum15
Dendrobium aphyllum10
Dendrobium moschatum13
Dendrobium moschatum12
Dendrobium moschatum11
Dendrobium falconeri8
Dendrobium aduncum7
Dendrobium heterocarpum8
Dendrobium heterocarpum6
Dendrobium heterocarpum7
Dendrobium moniliforme14
Dendrobium moniliforme13
Dendrobium moniliforme12
Dendrobium huoshanense4
Dendrobium huoshanense3
Dendrobium jenkinsii7
Dendrobium jenkinsii2
Dendrobium bellatulum3
Dendrobium draconis3
Dendrobium longicornu5
Dendrobium bellatulum4
Dendrobium williamsonii3
Dendrobium herbaceum3
Dendrobium herbaceum1
Dendrobium herbaceum2

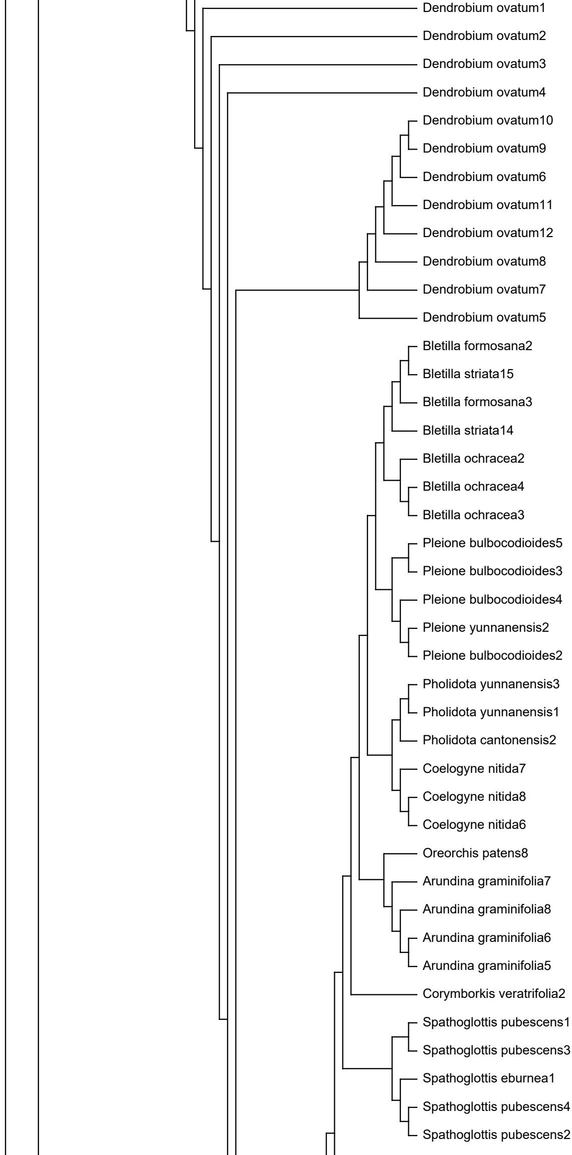

Dendrobium ovatum1
Dendrobium ovatum2
Dendrobium ovatum3
Dendrobium ovatum4
Dendrobium ovatum10
Dendrobium ovatum9
Dendrobium ovatum6
Dendrobium ovatum11
Dendrobium ovatum12
Dendrobium ovatum8
Dendrobium ovatum7
Dendrobium ovatum5
Bletilla formosana2
Bletilla striata15
Bletilla formosana3
Bletilla striata14
Bletilla ochracea2
Bletilla ochracea4
Bletilla ochracea3
Pleione bulbocodioides5
Pleione bulbocodioides3
Pleione bulbocodioides4
Pleione yunnanensis2
Pleione bulbocodioides2
Pholidota yunnanensis3
Pholidota yunnanensis1
Pholidota cantonensis2
Coelogyne nitida7
Coelogyne nitida8
Coelogyne nitida6
Oreorchis patens8
Arundina graminifolia7
Arundina graminifolia8
Arundina graminifolia6
Arundina graminifolia5
Corymborkis veratrifolia2
Spathoglottis pubescens1
Spathoglottis pubescens3
Spathoglottis eburnea1
Spathoglottis pubescens4
Spathoglottis pubescens2

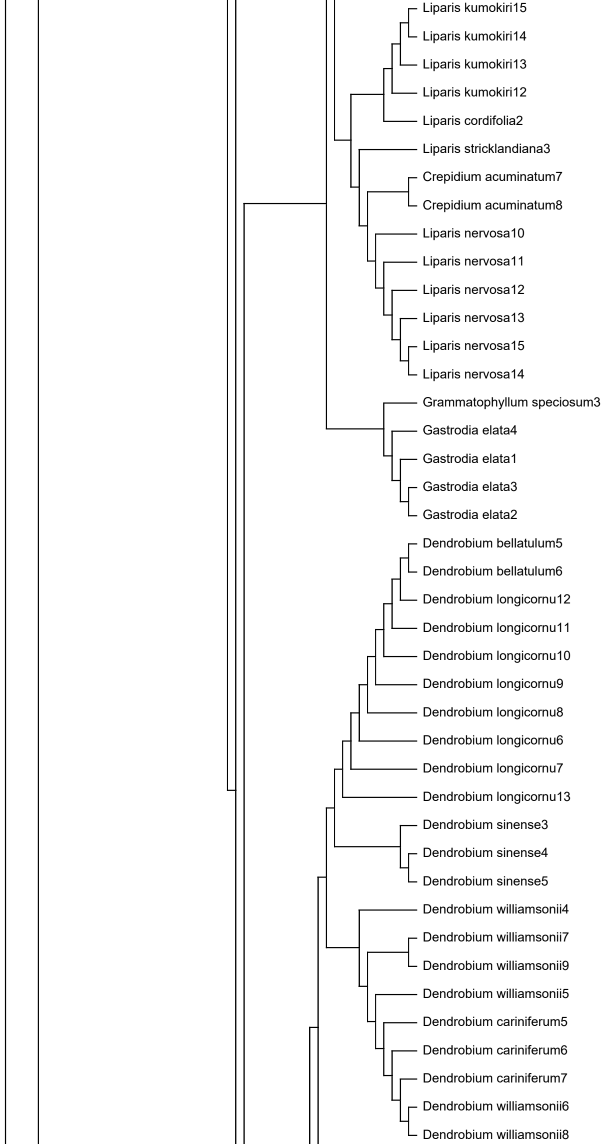

Liparis kumokiri15
Liparis kumokiri14
Liparis kumokiri13
Liparis kumokiri12
Liparis cordifolia2
Liparis stricklandiana3
Crepidium acuminatum7
Crepidium acuminatum8
Liparis nervosa10
Liparis nervosa11
Liparis nervosa12
Liparis nervosa13
Liparis nervosa15
Liparis nervosa14
Grammatophyllum speciosum3
Gastrodia elata4
Gastrodia elata1
Gastrodia elata3
Gastrodia elata2
Dendrobium bellatulum5
Dendrobium bellatulum6
Dendrobium longicornu12
Dendrobium longicornu11
Dendrobium longicornu10
Dendrobium longicornu9
Dendrobium longicornu8
Dendrobium longicornu6
Dendrobium longicornu7
Dendrobium longicornu13
Dendrobium sinense3
Dendrobium sinense4
Dendrobium sinense5
Dendrobium williamsonii4
Dendrobium williamsonii7
Dendrobium williamsonii9
Dendrobium williamsonii5
Dendrobium cariniferum5
Dendrobium cariniferum6
Dendrobium cariniferum7
Dendrobium williamsonii6
Dendrobium williamsonii8

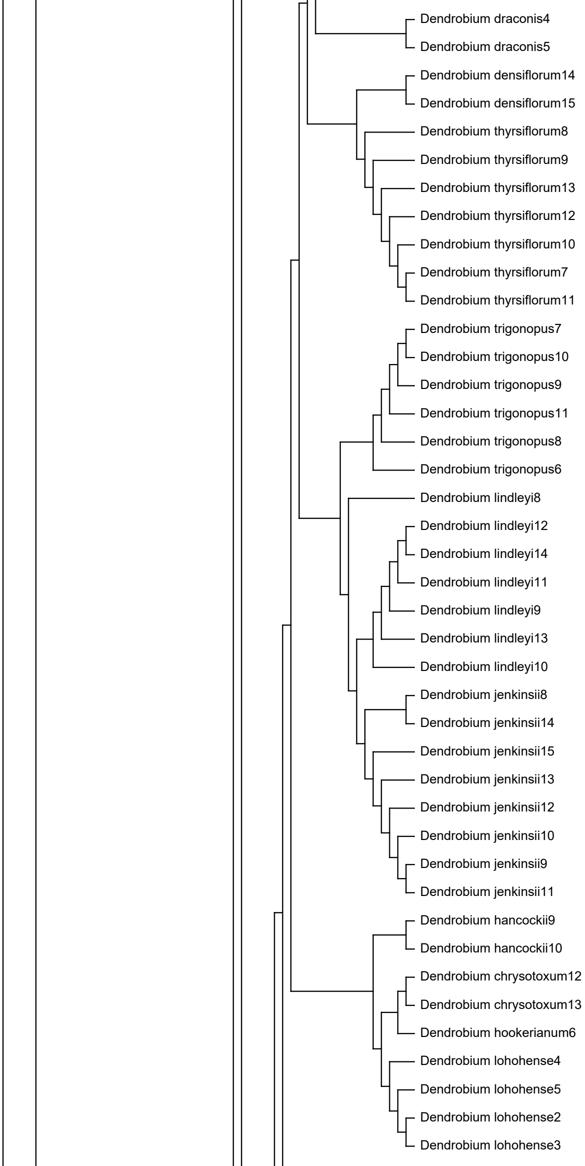

Dendrobium draconis4
Dendrobium draconis5
Dendrobium densiflorum14
Dendrobium densiflorum15
Dendrobium thyrsiflorum8
Dendrobium thyrsiflorum9
Dendrobium thyrsiflorum13
Dendrobium thyrsiflorum12
Dendrobium thyrsiflorum10
Dendrobium thyrsiflorum7
Dendrobium thyrsiflorum11
Dendrobium trigonopus7
Dendrobium trigonopus10
Dendrobium trigonopus9
Dendrobium trigonopus11
Dendrobium trigonopus8
Dendrobium trigonopus6
Dendrobium lindleyi8
Dendrobium lindleyi12
Dendrobium lindleyi14
Dendrobium lindleyi11
Dendrobium lindleyi9
Dendrobium lindleyi13
Dendrobium lindleyi10
Dendrobium jenkinsii8
Dendrobium jenkinsii14
Dendrobium jenkinsii15
Dendrobium jenkinsii13
Dendrobium jenkinsii12
Dendrobium jenkinsii10
Dendrobium jenkinsii9
Dendrobium jenkinsii11
Dendrobium hancockii9
Dendrobium hancockii10
Dendrobium chrysotoxum12
Dendrobium chrysotoxum13
Dendrobium hookerianum6
Dendrobium lohohense4
Dendrobium lohohense5
Dendrobium lohohense2
Dendrobium lohohense3

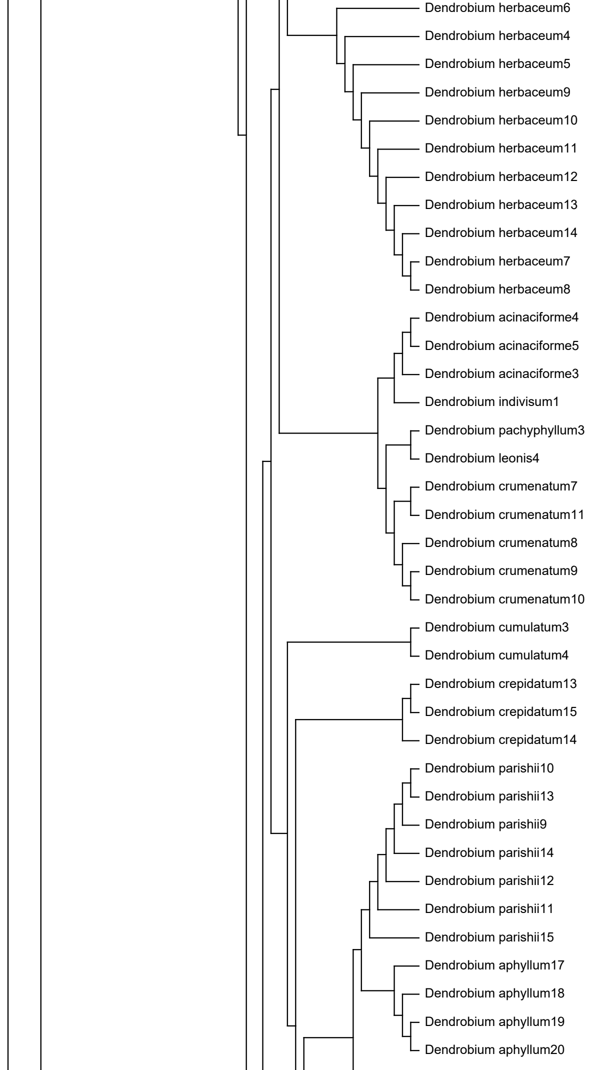
Dendrobium herbaceum6
Dendrobium herbaceum4
Dendrobium herbaceum5
Dendrobium herbaceum9
Dendrobium herbaceum10
Dendrobium herbaceum11
Dendrobium herbaceum12
Dendrobium herbaceum13
Dendrobium herbaceum14
Dendrobium herbaceum7
Dendrobium herbaceum8
Dendrobium acinaciforme4
Dendrobium acinaciforme5
Dendrobium acinaciforme3
Dendrobium indivisum1
Dendrobium pachyphyllum3
Dendrobium leonis4
Dendrobium crumenatum7
Dendrobium crumenatum11
Dendrobium crumenatum8
Dendrobium crumenatum9
Dendrobium crumenatum10
Dendrobium cumulatum3
Dendrobium cumulatum4
Dendrobium crepidatum13
Dendrobium crepidatum15
Dendrobium crepidatum14
Dendrobium parishii10
Dendrobium parishii13
Dendrobium parishii9
Dendrobium parishii14
Dendrobium parishii12
Dendrobium parishii11
Dendrobium parishii15
Dendrobium aphyllum17
Dendrobium aphyllum18
Dendrobium aphyllum19
Dendrobium aphyllum20

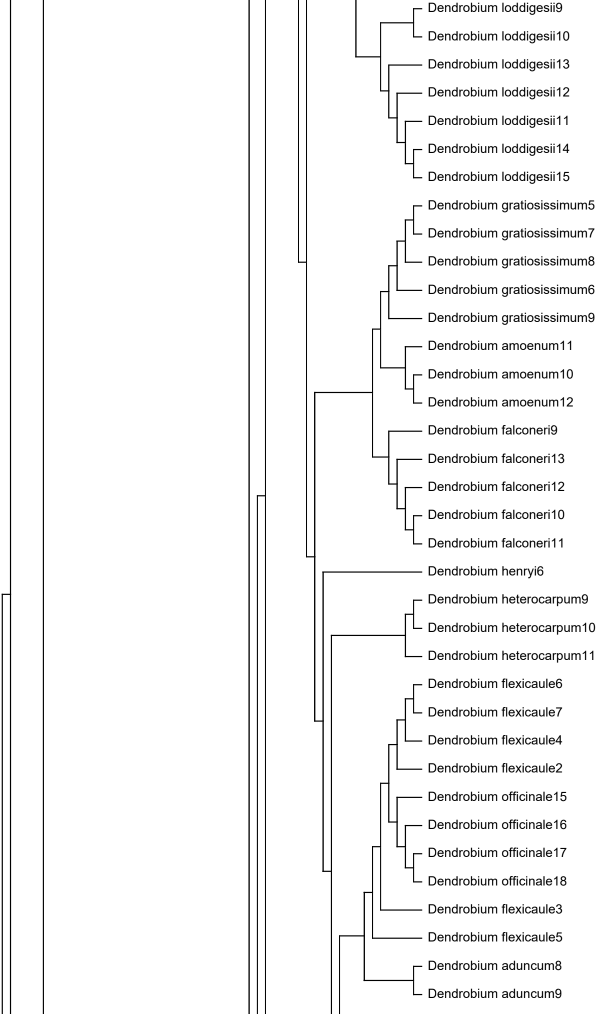
Dendrobium loddigesii9
Dendrobium loddigesii10
Dendrobium loddigesii13
Dendrobium loddigesii12
Dendrobium loddigesii11
Dendrobium loddigesii14
Dendrobium loddigesii15
Dendrobium gratiosissimum5
Dendrobium gratiosissimum7
Dendrobium gratiosissimum8
Dendrobium gratiosissimum6
Dendrobium gratiosissimum9
Dendrobium amoenum11
Dendrobium amoenum10
Dendrobium amoenum12
Dendrobium falconeri9
Dendrobium falconeri13
Dendrobium falconeri12
Dendrobium falconeri10
Dendrobium falconeri11
Dendrobium henryi6
Dendrobium heterocarpum9
Dendrobium heterocarpum10
Dendrobium heterocarpum11
Dendrobium flexicaule6
Dendrobium flexicaule7
Dendrobium flexicaule4
Dendrobium flexicaule2
Dendrobium officinale15
Dendrobium officinale16
Dendrobium officinale17
Dendrobium officinale18
Dendrobium flexicaule3
Dendrobium flexicaule5
Dendrobium aduncum8
Dendrobium aduncum9

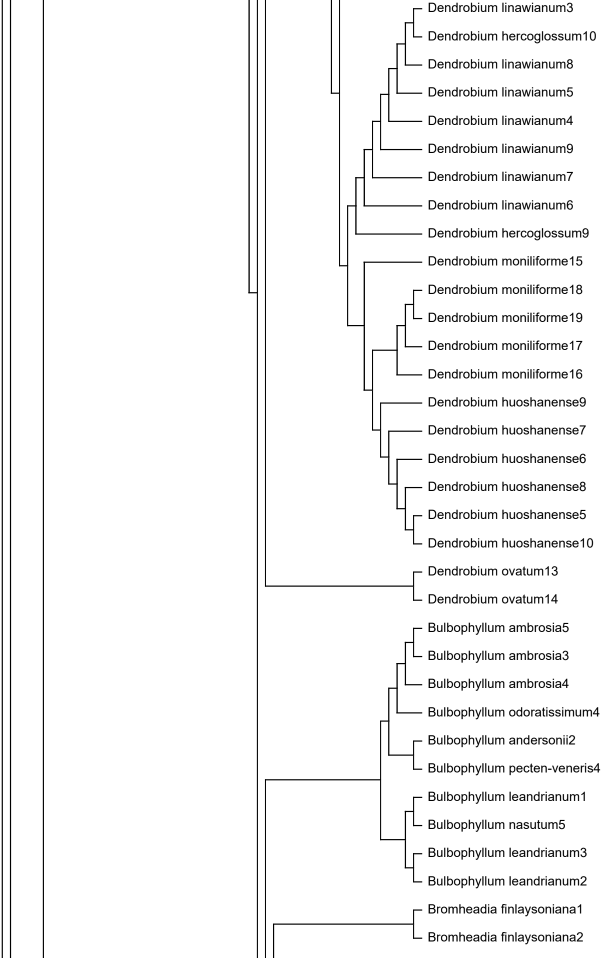
Dendrobium linawianum3
Dendrobium hercoglossum10
Dendrobium linawianum8
Dendrobium linawianum5
Dendrobium linawianum4
Dendrobium linawianum9
Dendrobium linawianum7
Dendrobium linawianum6
Dendrobium hercoglossum9
Dendrobium moniliforme15
Dendrobium moniliforme18
Dendrobium moniliforme19
Dendrobium moniliforme17
Dendrobium moniliforme16
Dendrobium huoshanense9
Dendrobium huoshanense7
Dendrobium huoshanense6
Dendrobium huoshanense8
Dendrobium huoshanense5
Dendrobium huoshanense10
Dendrobium ovatum13
Dendrobium ovatum14
Bulbophyllum ambrosia5
Bulbophyllum ambrosia3
Bulbophyllum ambrosia4
Bulbophyllum odoratissimum4
Bulbophyllum andersonii2
Bulbophyllum pecten-veneris4
Bulbophyllum leandrianum1
Bulbophyllum nasutum5
Bulbophyllum leandrianum3
Bulbophyllum leandrianum2
Bromheadia finlaysoniana1
Bromheadia finlaysoniana2

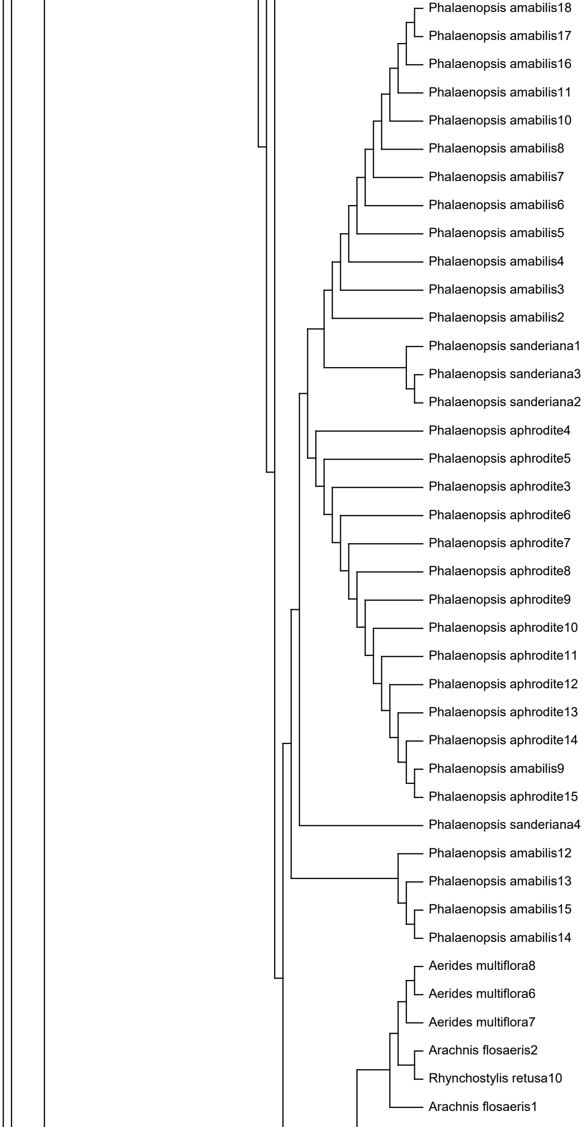
Phalaenopsis amabilis18
Phalaenopsis amabilis17
Phalaenopsis amabilis16
Phalaenopsis amabilis11
Phalaenopsis amabilis10
Phalaenopsis amabilis8
Phalaenopsis amabilis7
Phalaenopsis amabilis6
Phalaenopsis amabilis5
Phalaenopsis amabilis4
Phalaenopsis amabilis3
Phalaenopsis amabilis2
Phalaenopsis sanderiana1
Phalaenopsis sanderiana3
Phalaenopsis sanderiana2
Phalaenopsis aphrodite4
Phalaenopsis aphrodite5
Phalaenopsis aphrodite3
Phalaenopsis aphrodite6
Phalaenopsis aphrodite7
Phalaenopsis aphrodite8
Phalaenopsis aphrodite9
Phalaenopsis aphrodite10
Phalaenopsis aphrodite11
Phalaenopsis aphrodite12
Phalaenopsis aphrodite13
Phalaenopsis aphrodite14
Phalaenopsis amabilis9
Phalaenopsis aphrodite15
Phalaenopsis sanderiana4
Phalaenopsis amabilis12
Phalaenopsis amabilis13
Phalaenopsis amabilis15
Phalaenopsis amabilis14
Aerides multiflora8
Aerides multiflora6
Aerides multiflora7
Arachnis flosaeris2
Rhynchostylis retusa10
Arachnis flosaeris1

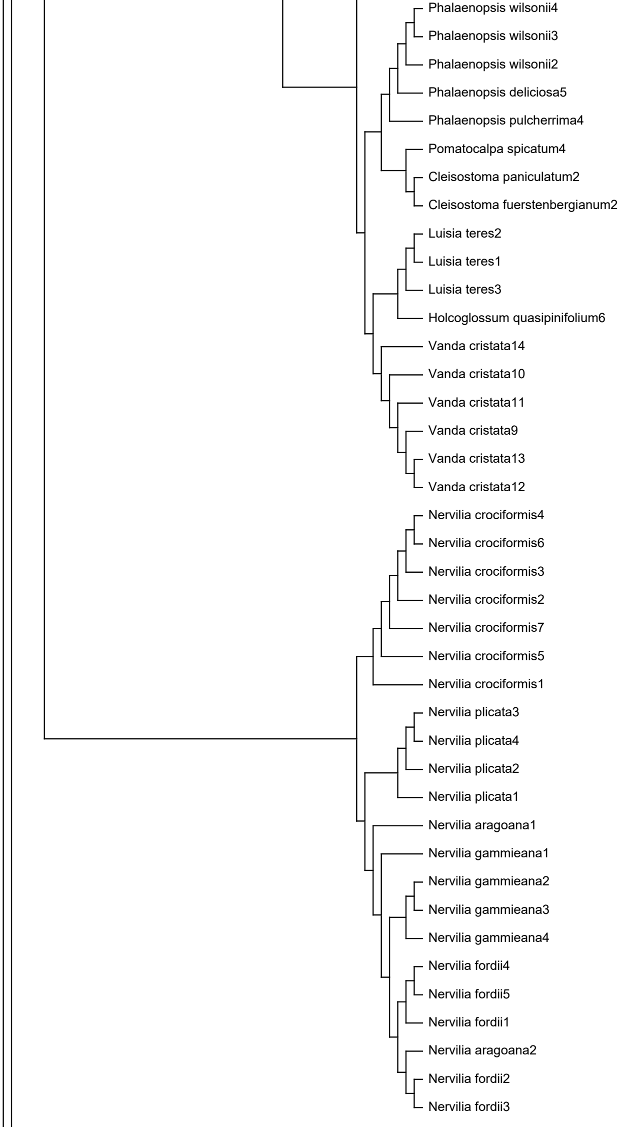

Phalaenopsis wilsonii4
Phalaenopsis wilsonii3
Phalaenopsis wilsonii2
Phalaenopsis deliciosa5
Phalaenopsis pulcherrima4
Pomatocalpa spicatum4
Cleisostoma paniculatum2
Cleisostoma fuerstenbergianum2
Luisia teres2
Luisia teres1
Luisia teres3
Holcoglossum quasipinifolium6
Vanda cristata14
Vanda cristata10
Vanda cristata11
Vanda cristata9
Vanda cristata13
Vanda cristata12
Nervilia crociformis4
Nervilia crociformis6
Nervilia crociformis3
Nervilia crociformis2
Nervilia crociformis7
Nervilia crociformis5
Nervilia crociformis1
Nervilia plicata3
Nervilia plicata4
Nervilia plicata2
Nervilia plicata1
Nervilia aragoana1
Nervilia gammieana1
Nervilia gammieana2
Nervilia gammieana3
Nervilia gammieana4
Nervilia fordii4
Nervilia fordii5
Nervilia fordii1
Nervilia aragoana2
Nervilia fordii2
Nervilia fordii3

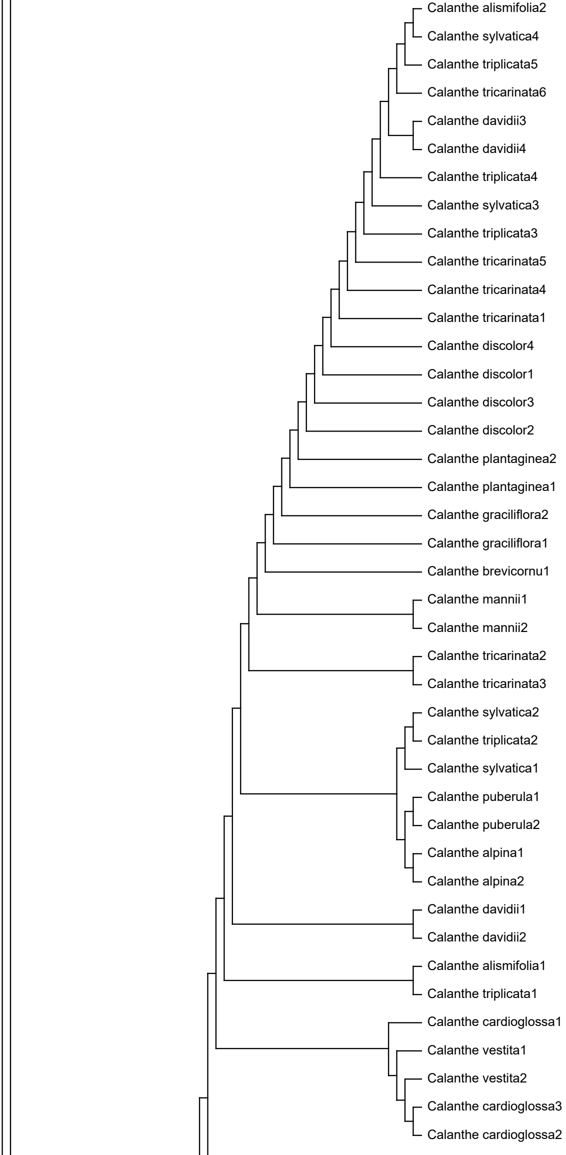
Calanthe alismifolia2
Calanthe sylvatica4
Calanthe triplicata5
Calanthe tricarinata6
Calanthe davidii3
Calanthe davidii4
Calanthe triplicata4
Calanthe sylvatica3
Calanthe triplicata3
Calanthe tricarinata5
Calanthe tricarinata4
Calanthe tricarinata1
Calanthe discolor4
Calanthe discolor1
Calanthe discolor3
Calanthe discolor2
Calanthe plantaginea2
Calanthe plantaginea1
Calanthe graciliflora2
Calanthe graciliflora1
Calanthe brevicornu1
Calanthe mannii1
Calanthe mannii2
Calanthe tricarinata2
Calanthe tricarinata3
Calanthe sylvatica2
Calanthe triplicata2
Calanthe sylvatica1
Calanthe puberula1
Calanthe puberula2
Calanthe alpina1
Calanthe alpina2
Calanthe davidii1
Calanthe davidii2
Calanthe alismifolia1
Calanthe triplicata1
Calanthe cardioglossa1
Calanthe vestita1
Calanthe vestita2
Calanthe cardioglossa3
Calanthe cardioglossa2

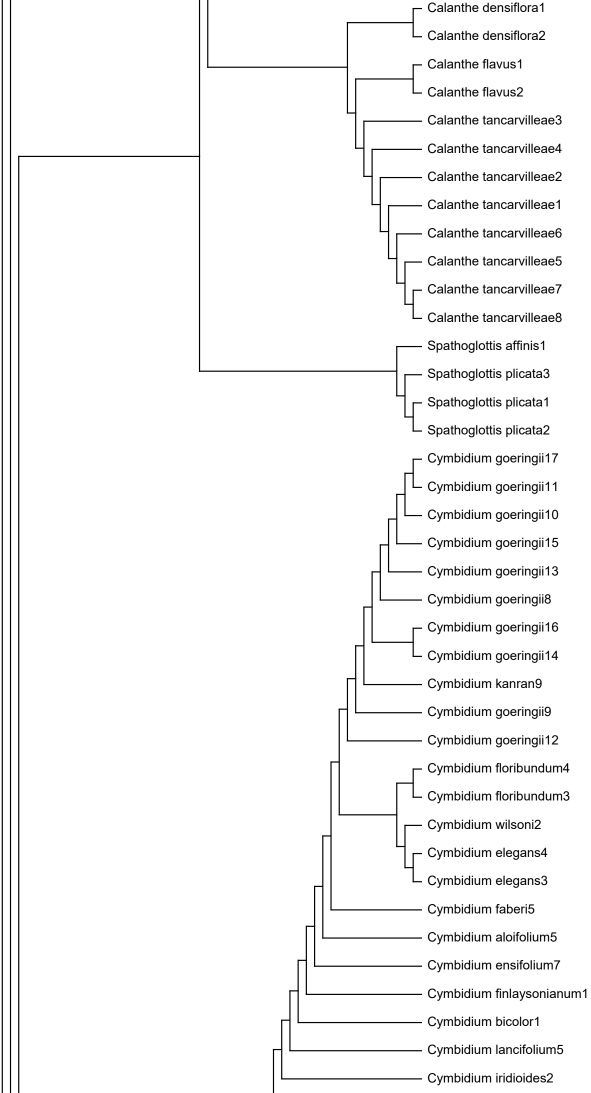
Calanthe densiflora1
Calanthe densiflora2
Calanthe flavus1
Calanthe flavus2
Calanthe tancarvilleae3
Calanthe tancarvilleae4
Calanthe tancarvilleae2
Calanthe tancarvilleae1
Calanthe tancarvilleae6
Calanthe tancarvilleae5
Calanthe tancarvilleae7
Calanthe tancarvilleae8
Spathoglottis affinis1
Spathoglottis plicata3
Spathoglottis plicata1
Spathoglottis plicata2
Cymbidium goeringii17
Cymbidium goeringii11
Cymbidium goeringii10
Cymbidium goeringii15
Cymbidium goeringii13
Cymbidium goeringii8
Cymbidium goeringii16
Cymbidium goeringii14
Cymbidium kanran9
Cymbidium goeringii9
Cymbidium goeringii12
Cymbidium floribundum4
Cymbidium floribundum3
Cymbidium wilsoni2
Cymbidium elegans4
Cymbidium elegans3
Cymbidium faberi5
Cymbidium aloifolium5
Cymbidium ensifolium7
Cymbidium finlaysonianum1
Cymbidium bicolor1
Cymbidium lancifolium5
Cymbidium iridioides2

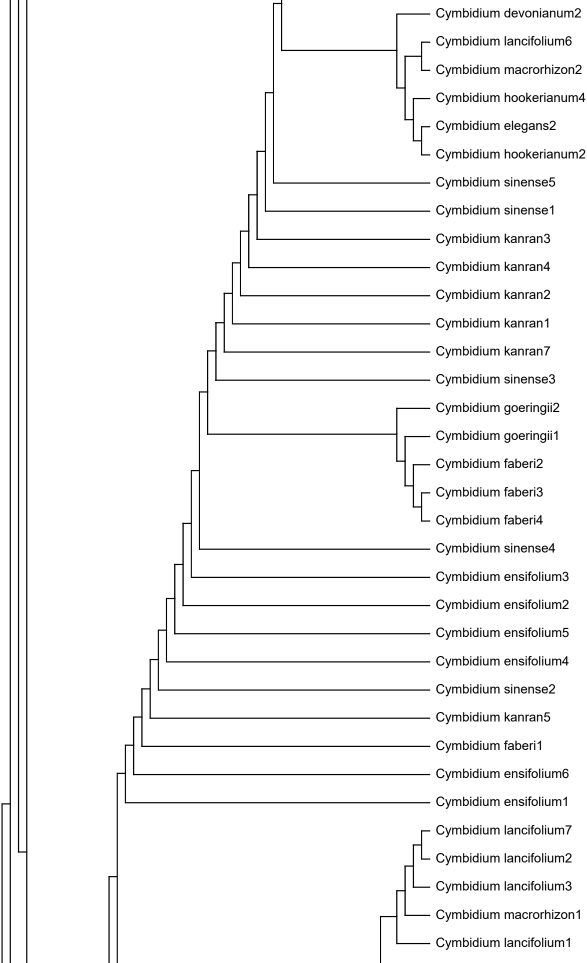
Cymbidium devonianum2
Cymbidium lancifolium6
Cymbidium macrorhizon2
Cymbidium hookerianum4
Cymbidium elegans2
Cymbidium hookerianum2
Cymbidium sinense5
Cymbidium sinense1
Cymbidium kanran3
Cymbidium kanran4
Cymbidium kanran2
Cymbidium kanran1
Cymbidium kanran7
Cymbidium sinense3
Cymbidium goeringii2
Cymbidium goeringii1
Cymbidium faberi2
Cymbidium faberi3
Cymbidium faberi4
Cymbidium sinense4
Cymbidium ensifolium3
Cymbidium ensifolium2
Cymbidium ensifolium5
Cymbidium ensifolium4
Cymbidium sinense2
Cymbidium kanran5
Cymbidium faberi1
Cymbidium ensifolium6
Cymbidium ensifolium1
Cymbidium lancifolium7
Cymbidium lancifolium2
Cymbidium lancifolium3
Cymbidium macrorhizon1
Cymbidium lancifolium1

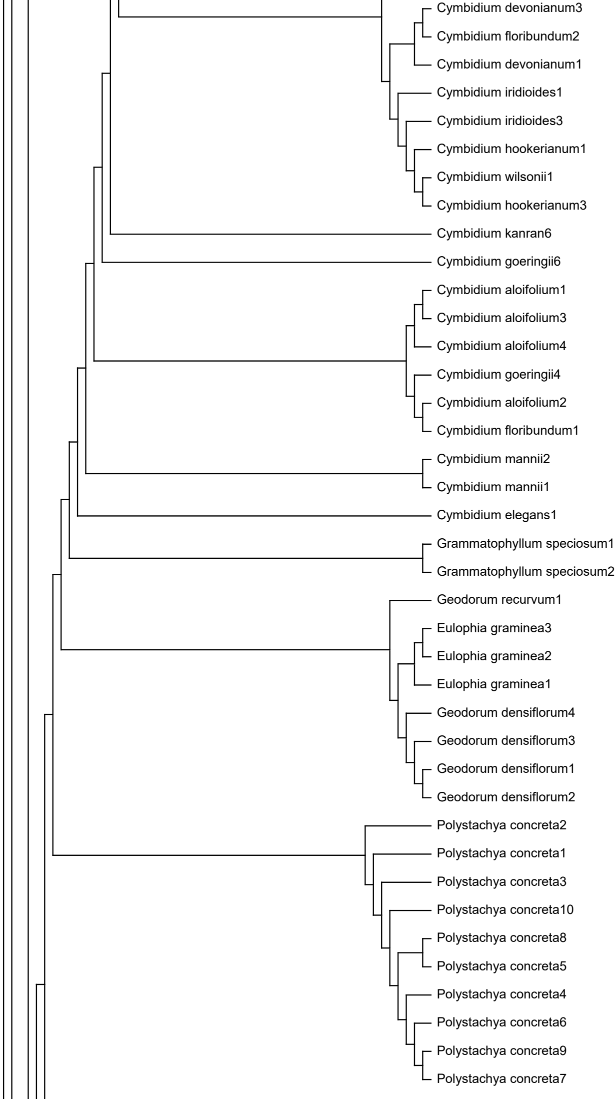

Cymbidium devonianum3
Cymbidium floribundum2
Cymbidium devonianum1
Cymbidium iridioides1
Cymbidium iridioides3
Cymbidium hookerianum1
Cymbidium wilsonii1
Cymbidium hookerianum3
Cymbidium kanran6
Cymbidium goeringii6
Cymbidium aloifolium1
Cymbidium aloifolium3
Cymbidium aloifolium4
Cymbidium goeringii4
Cymbidium aloifolium2
Cymbidium floribundum1
Cymbidium mannii2
Cymbidium mannii1
Cymbidium elegans1
Grammatophyllum speciosum1
Grammatophyllum speciosum2
Geodorum recurvum1
Eulophia graminea3
Eulophia graminea2
Eulophia graminea1
Geodorum densiflorum4
Geodorum densiflorum3
Geodorum densiflorum1
Geodorum densiflorum2
Polystachya concreta2
Polystachya concreta1
Polystachya concreta3
Polystachya concreta10
Polystachya concreta8
Polystachya concreta5
Polystachya concreta4
Polystachya concreta6
Polystachya concreta9
Polystachya concreta7

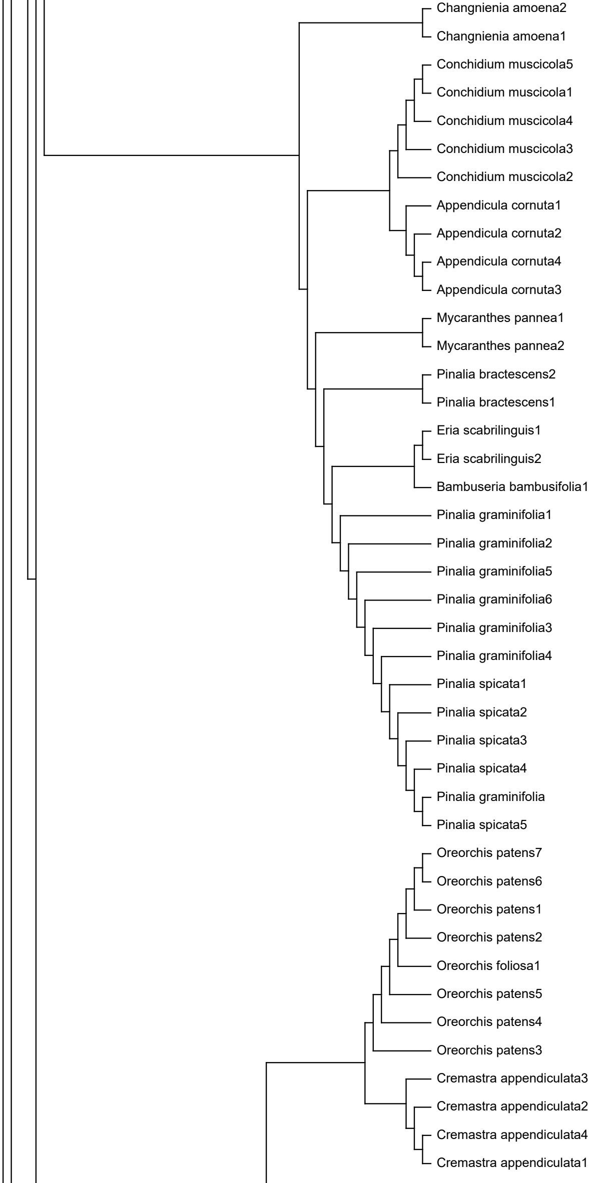
Changnienia amoena2
Changnienia amoena1
Conchidium muscicola5
Conchidium muscicola1
Conchidium muscicola4
Conchidium muscicola3
Conchidium muscicola2
Appendicula cornuta1
Appendicula cornuta2
Appendicula cornuta4
Appendicula cornuta3
Mycaranthes pannea1
Mycaranthes pannea2
Pinalia bractescens2
Pinalia bractescens1
Eria scabrilinguis1
Eria scabrilinguis2
Bambuseria bambusifolia1
Pinalia graminifolia1
Pinalia graminifolia2
Pinalia graminifolia5
Pinalia graminifolia6
Pinalia graminifolia3
Pinalia graminifolia4
Pinalia spicata1
Pinalia spicata2
Pinalia spicata3
Pinalia spicata4
Pinalia graminifolia
Pinalia spicata5
Oreorchis patens7
Oreorchis patens6
Oreorchis patens1
Oreorchis patens2
Oreorchis foliosa1
Oreorchis patens5
Oreorchis patens4
Oreorchis patens3
Cremastra appendiculata3
Cremastra appendiculata2
Cremastra appendiculata4
Cremastra appendiculata1

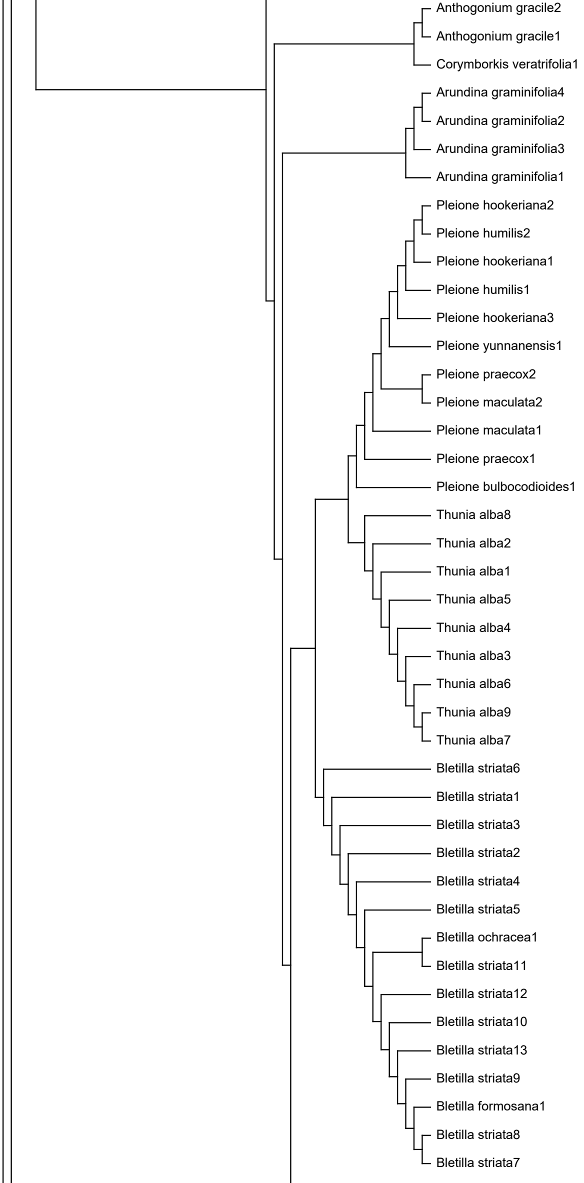

Anthogonium gracile2
Anthogonium gracile1
Corymborkis veratrifolia1
Arundina graminifolia4
Arundina graminifolia2
Arundina graminifolia3
Arundina graminifolia1
Pleione hookeriana2
Pleione humilis2
Pleione hookeriana1
Pleione humilis1
Pleione hookeriana3
Pleione yunnanensis1
Pleione praecox2
Pleione maculata2
Pleione maculata1
Pleione praecox1
Pleione bulbocodioides1
Thunia alba8
Thunia alba2
Thunia alba1
Thunia alba5
Thunia alba4
Thunia alba3
Thunia alba6
Thunia alba9
Thunia alba7
Bletilla striata6
Bletilla striata1
Bletilla striata3
Bletilla striata2
Bletilla striata4
Bletilla striata5
Bletilla ochracea1
Bletilla striata11
Bletilla striata12
Bletilla striata10
Bletilla striata13
Bletilla striata9
Bletilla formosana1
Bletilla striata8
Bletilla striata7

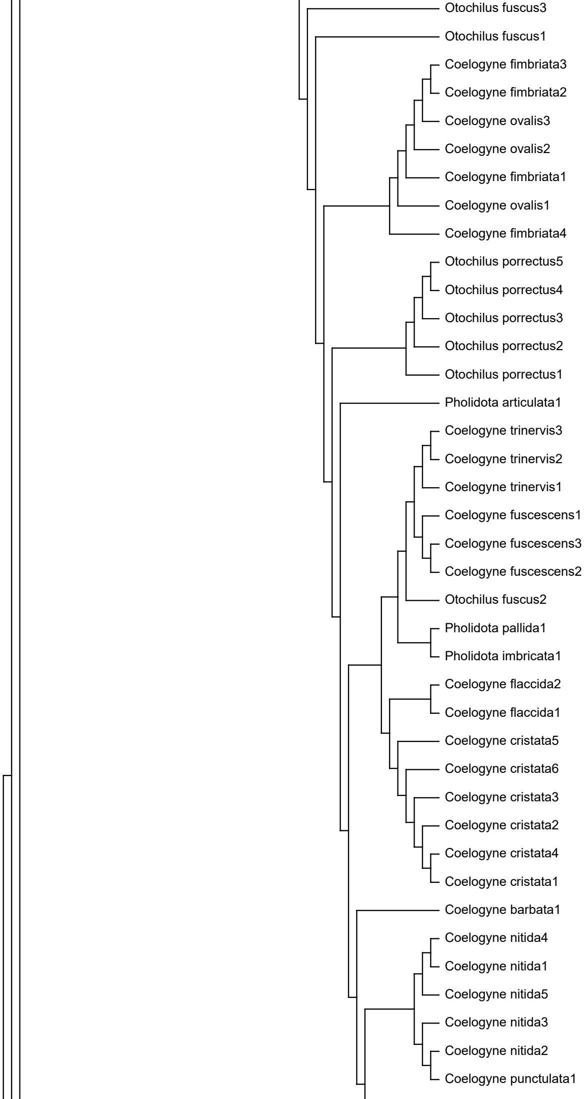
Otochilus fuscus3
Otochilus fuscus1
Coelogyne fimbriata3
Coelogyne fimbriata2
Coelogyne ovalis3
Coelogyne ovalis2
Coelogyne fimbriata1
Coelogyne ovalis1
Coelogyne fimbriata4
Otochilus porrectus5
Otochilus porrectus4
Otochilus porrectus3
Otochilus porrectus2
Otochilus porrectus1
Pholidota articulata1
Coelogyne trinervis3
Coelogyne trinervis2
Coelogyne trinervis1
Coelogyne fuscescens1
Coelogyne fuscescens3
Coelogyne fuscescens2
Otochilus fuscus2
Pholidota pallida1
Pholidota imbricata1
Coelogyne flaccida2
Coelogyne flaccida1
Coelogyne cristata5
Coelogyne cristata6
Coelogyne cristata3
Coelogyne cristata2
Coelogyne cristata4
Coelogyne cristata1
Coelogyne barbata1
Coelogyne nitida4
Coelogyne nitida1
Coelogyne nitida5
Coelogyne nitida3
Coelogyne nitida2
Coelogyne punctulata1

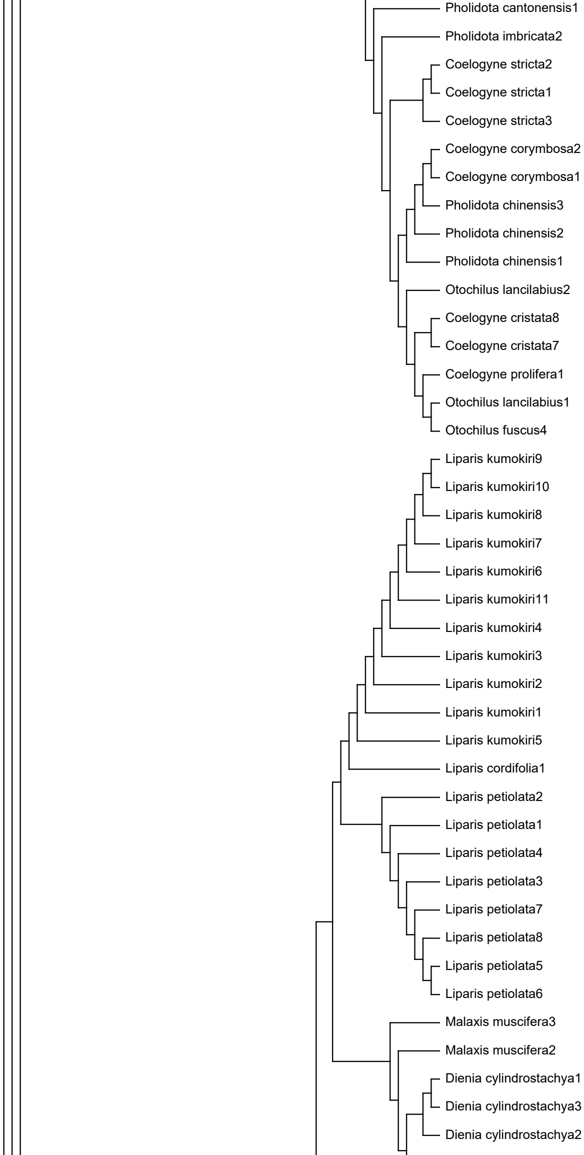
Pholidota cantonensis1
Pholidota imbricata2
Coelogyne stricta2
Coelogyne stricta1
Coelogyne stricta3
Coelogyne corymbosa2
Coelogyne corymbosa1
Pholidota chinensis3
Pholidota chinensis2
Pholidota chinensis1
Otochilus lancilabius2
Coelogyne cristata8
Coelogyne cristata7
Coelogyne prolifera1
Otochilus lancilabius1
Otochilus fuscus4
Liparis kumokiri9
Liparis kumokiri10
Liparis kumokiri8
Liparis kumokiri7
Liparis kumokiri6
Liparis kumokiri11
Liparis kumokiri4
Liparis kumokiri3
Liparis kumokiri2
Liparis kumokiri1
Liparis kumokiri5
Liparis cordifolia1
Liparis petiolata2
Liparis petiolata1
Liparis petiolata4
Liparis petiolata3
Liparis petiolata7
Liparis petiolata8
Liparis petiolata5
Liparis petiolata6
Malaxis muscifera3
Malaxis muscifera2
Dienia cylindrostachya1
Dienia cylindrostachya3
Dienia cylindrostachya2

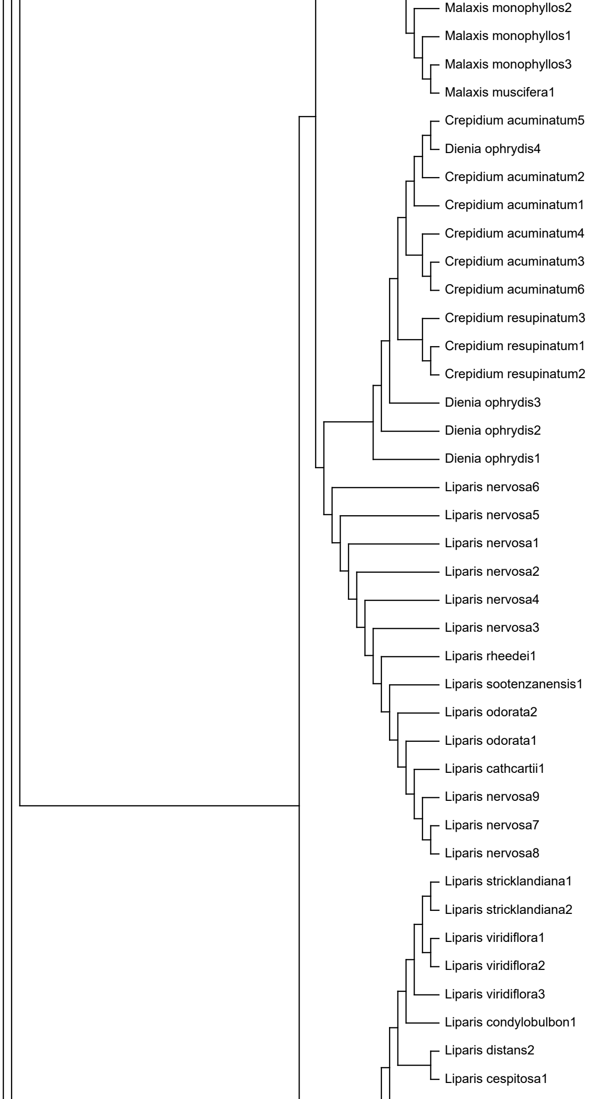
Malaxis monophyllos2
Malaxis monophyllos1
Malaxis monophyllos3
Malaxis muscifera1
Crepidium acuminatum5
Dienia ophrydis4
Crepidium acuminatum2
Crepidium acuminatum1
Crepidium acuminatum4
Crepidium acuminatum3
Crepidium acuminatum6
Crepidium resupinatum3
Crepidium resupinatum1
Crepidium resupinatum2
Dienia ophrydis3
Dienia ophrydis2
Dienia ophrydis1
Liparis nervosa6
Liparis nervosa5
Liparis nervosa1
Liparis nervosa2
Liparis nervosa4
Liparis nervosa3
Liparis rheedei1
Liparis sootenzanensis1
Liparis odorata2
Liparis odorata1
Liparis cathcartii1
Liparis nervosa9
Liparis nervosa7
Liparis nervosa8
Liparis stricklandiana1
Liparis stricklandiana2
Liparis viridiflora1
Liparis viridiflora2
Liparis viridiflora3
Liparis condylobulbon1
Liparis distans2
Liparis cespitosa1

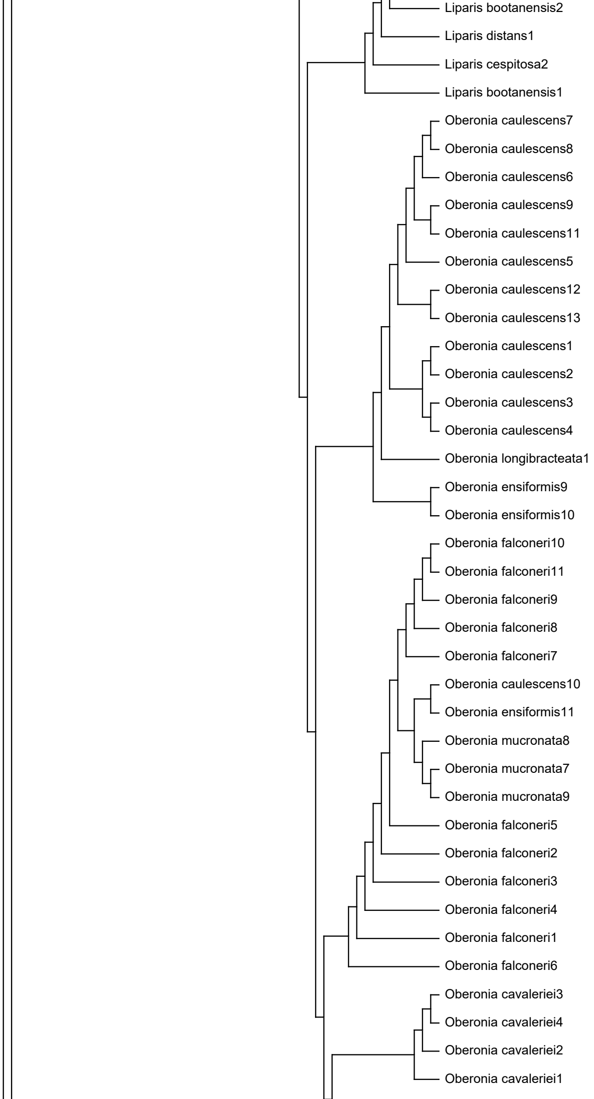
Liparis bootanensis2
Liparis distans1
Liparis cespitosa2
Liparis bootanensis1
Oberonia caulescens7
Oberonia caulescens8
Oberonia caulescens6
Oberonia caulescens9
Oberonia caulescens11
Oberonia caulescens5
Oberonia caulescens12
Oberonia caulescens13
Oberonia caulescens1
Oberonia caulescens2
Oberonia caulescens3
Oberonia caulescens4
Oberonia longibracteata1
Oberonia ensiformis9
Oberonia ensiformis10
Oberonia falconeri10
Oberonia falconeri11
Oberonia falconeri9
Oberonia falconeri8
Oberonia falconeri7
Oberonia caulescens10
Oberonia ensiformis11
Oberonia mucronata8
Oberonia mucronata7
Oberonia mucronata9
Oberonia falconeri5
Oberonia falconeri2
Oberonia falconeri3
Oberonia falconeri4
Oberonia falconeri1
Oberonia falconeri6
Oberonia cavaleriei3
Oberonia cavaleriei4
Oberonia cavaleriei2
Oberonia cavaleriei1

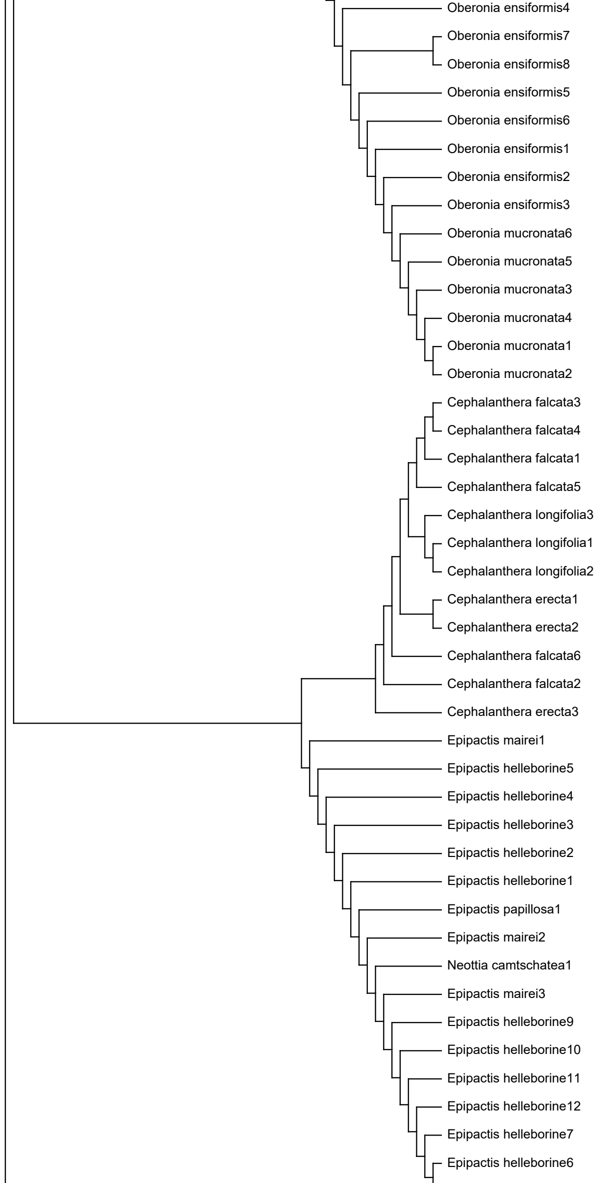
Oberonia ensiformis4
Oberonia ensiformis7
Oberonia ensiformis8
Oberonia ensiformis5
Oberonia ensiformis6
Oberonia ensiformis1
Oberonia ensiformis2
Oberonia ensiformis3
Oberonia mucronata6
Oberonia mucronata5
Oberonia mucronata3
Oberonia mucronata4
Oberonia mucronata1
Oberonia mucronata2
Cephalanthera falcata3
Cephalanthera falcata4
Cephalanthera falcata1
Cephalanthera falcata5
Cephalanthera longifolia3
Cephalanthera longifolia1
Cephalanthera longifolia2
Cephalanthera erecta1
Cephalanthera erecta2
Cephalanthera falcata6
Cephalanthera falcata2
Cephalanthera erecta3
Epipactis mairei1
Epipactis helleborine5
Epipactis helleborine4
Epipactis helleborine3
Epipactis helleborine2
Epipactis helleborine1
Epipactis papillosa1
Epipactis mairei2
Neottia camtschatea1
Epipactis mairei3
Epipactis helleborine9
Epipactis helleborine10
Epipactis helleborine11
Epipactis helleborine12
Epipactis helleborine7
Epipactis helleborine6

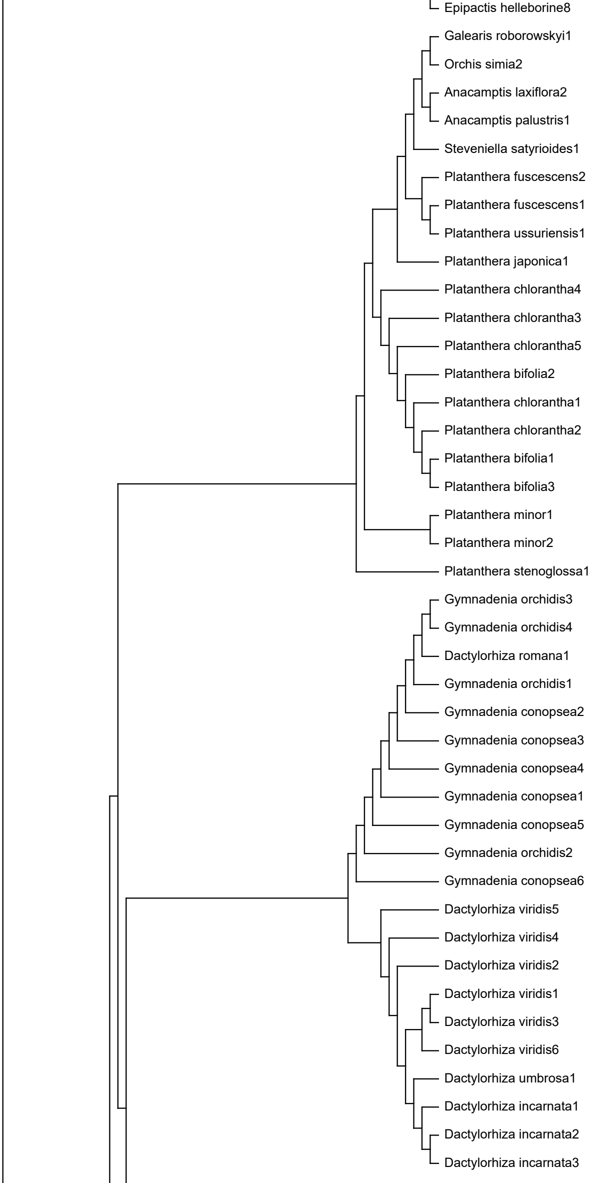
Epipactis helleborine8
Galearis roborowskyi1
Orchis simia2
Anacamptis laxiflora2
Anacamptis palustris1
Steveniella satyrioides1
Platanthera fuscescens2
Platanthera fuscescens1
Platanthera ussuriensis1
Platanthera japonica1
Platanthera chlorantha4
Platanthera chlorantha3
Platanthera chlorantha5
Platanthera bifolia2
Platanthera chlorantha1
Platanthera chlorantha2
Platanthera bifolia1
Platanthera bifolia3
Platanthera minor1
Platanthera minor2
Platanthera stenoglossa1
Gymnadenia orchidis3
Gymnadenia orchidis4
Dactylorhiza romana1
Gymnadenia orchidis1
Gymnadenia conopsea2
Gymnadenia conopsea3
Gymnadenia conopsea4
Gymnadenia conopsea1
Gymnadenia conopsea5
Gymnadenia orchidis2
Gymnadenia conopsea6
Dactylorhiza viridis5
Dactylorhiza viridis4
Dactylorhiza viridis2
Dactylorhiza viridis1
Dactylorhiza viridis3
Dactylorhiza viridis6
Dactylorhiza umbrosa1
Dactylorhiza incarnata1
Dactylorhiza incarnata2
Dactylorhiza incarnata3

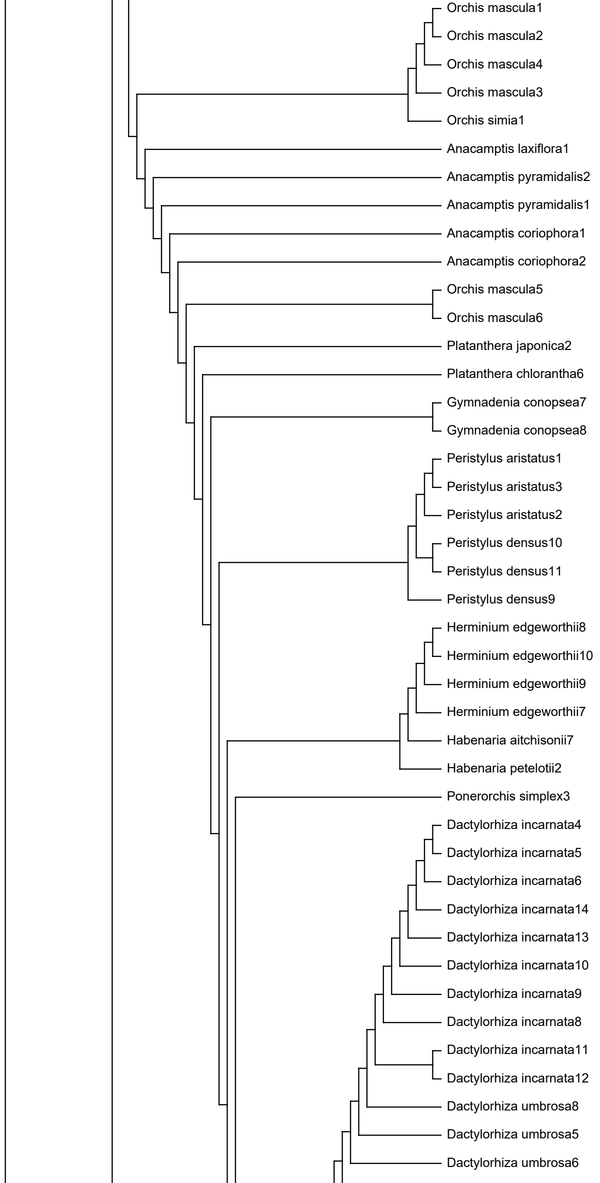

Orchis mascula1
Orchis mascula2
Orchis mascula4
Orchis mascula3
Orchis simia1
Anacamptis laxiflora1
Anacamptis pyramidalis2
Anacamptis pyramidalis1
Anacamptis coriophora1
Anacamptis coriophora2
Orchis mascula5
Orchis mascula6
Platanthera japonica2
Platanthera chlorantha6
Gymnadenia conopsea7
Gymnadenia conopsea8
Peristylus aristatus1
Peristylus aristatus3
Peristylus aristatus2
Peristylus densus10
Peristylus densus11
Peristylus densus9
Herminium edgeworthii8
Herminium edgeworthii10
Herminium edgeworthii9
Herminium edgeworthii7
Habenaria aitchisonii7
Habenaria petelotii2
Ponerorchis simplex3
Dactylorhiza incarnata4
Dactylorhiza incarnata5
Dactylorhiza incarnata6
Dactylorhiza incarnata14
Dactylorhiza incarnata13
Dactylorhiza incarnata10
Dactylorhiza incarnata9
Dactylorhiza incarnata8
Dactylorhiza incarnata11
Dactylorhiza incarnata12
Dactylorhiza umbrosa8
Dactylorhiza umbrosa5
Dactylorhiza umbrosa6

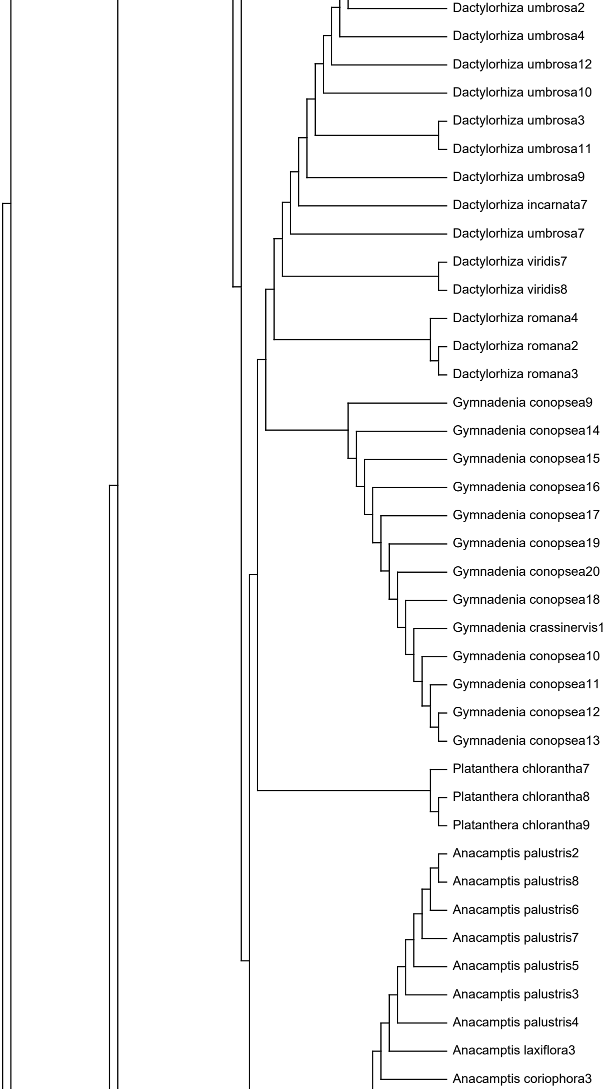

Dactylorhiza umbrosa2
Dactylorhiza umbrosa4
Dactylorhiza umbrosa12
Dactylorhiza umbrosa10
Dactylorhiza umbrosa3
Dactylorhiza umbrosa11
Dactylorhiza umbrosa9
Dactylorhiza incarnata7
Dactylorhiza umbrosa7
Dactylorhiza viridis7
Dactylorhiza viridis8
Dactylorhiza romana4
Dactylorhiza romana2
Dactylorhiza romana3
Gymnadenia conopsea9
Gymnadenia conopsea14
Gymnadenia conopsea15
Gymnadenia conopsea16
Gymnadenia conopsea17
Gymnadenia conopsea19
Gymnadenia conopsea20
Gymnadenia conopsea18
Gymnadenia crassinervis1
Gymnadenia conopsea10
Gymnadenia conopsea11
Gymnadenia conopsea12
Gymnadenia conopsea13
Platanthera chlorantha7
Platanthera chlorantha8
Platanthera chlorantha9
Anacamptis palustris2
Anacamptis palustris8
Anacamptis palustris6
Anacamptis palustris7
Anacamptis palustris5
Anacamptis palustris3
Anacamptis palustris4
Anacamptis laxiflora3
Anacamptis coriophora3

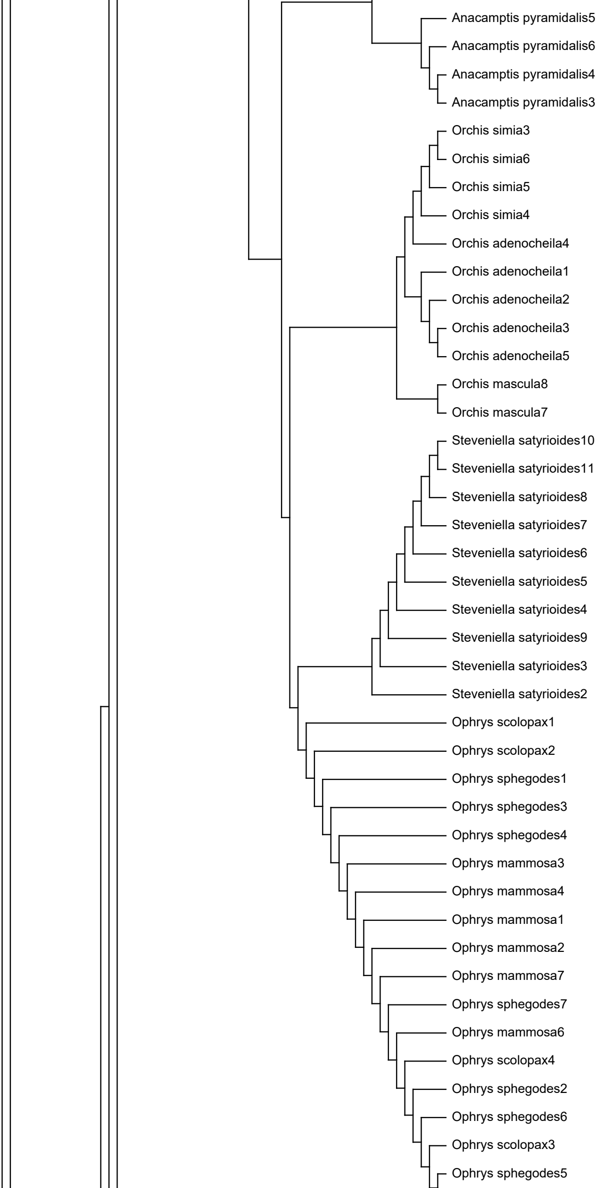
Anacamptis pyramidalis5
Anacamptis pyramidalis6
Anacamptis pyramidalis4
Anacamptis pyramidalis3
Orchis simia3
Orchis simia6
Orchis simia5
Orchis simia4
Orchis adenocheila4
Orchis adenocheila1
Orchis adenocheila2
Orchis adenocheila3
Orchis adenocheila5
Orchis mascula8
Orchis mascula7
Steveniella satyrioides10
Steveniella satyrioides11
Steveniella satyrioides8
Steveniella satyrioides7
Steveniella satyrioides6
Steveniella satyrioides5
Steveniella satyrioides4
Steveniella satyrioides9
Steveniella satyrioides3
Steveniella satyrioides2
Ophrys scolopax1
Ophrys scolopax2
Ophrys sphegodes1
Ophrys sphegodes3
Ophrys sphegodes4
Ophrys mammosa3
Ophrys mammosa4
Ophrys mammosa1
Ophrys mammosa2
Ophrys mammosa7
Ophrys sphegodes7
Ophrys mammosa6
Ophrys scolopax4
Ophrys sphegodes2
Ophrys sphegodes6
Ophrys scolopax3
Ophrys sphegodes5

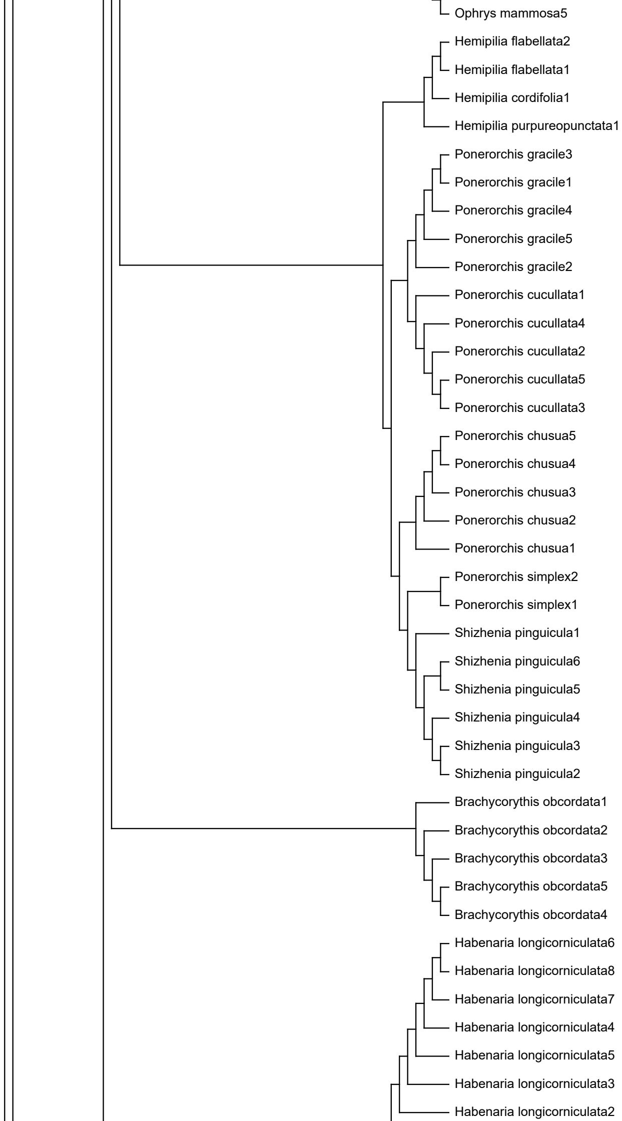
Ophrys mammosa5
Hemipilia flabellata2
Hemipilia flabellata1
Hemipilia cordifolia1
Hemipilia purpureopunctata1
Ponerorchis gracile3
Ponerorchis gracile1
Ponerorchis gracile4
Ponerorchis gracile5
Ponerorchis gracile2
Ponerorchis cucullata1
Ponerorchis cucullata4
Ponerorchis cucullata2
Ponerorchis cucullata5
Ponerorchis cucullata3
Ponerorchis chusua5
Ponerorchis chusua4
Ponerorchis chusua3
Ponerorchis chusua2
Ponerorchis chusua1
Ponerorchis simplex2
Ponerorchis simplex1
Shizhenia pinguicula1
Shizhenia pinguicula6
Shizhenia pinguicula5
Shizhenia pinguicula4
Shizhenia pinguicula3
Shizhenia pinguicula2
Brachycorythis obcordata1
Brachycorythis obcordata2
Brachycorythis obcordata3
Brachycorythis obcordata5
Brachycorythis obcordata4
Habenaria longicorniculata6
Habenaria longicorniculata8
Habenaria longicorniculata7
Habenaria longicorniculata4
Habenaria longicorniculata5
Habenaria longicorniculata3
Habenaria longicorniculata2

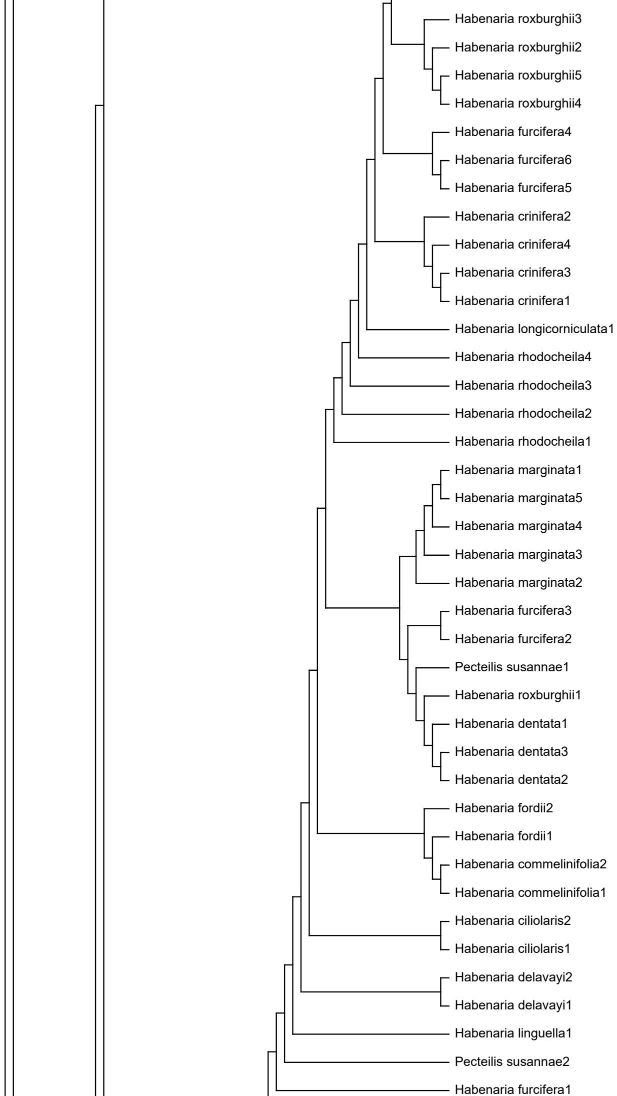
Habenaria roxburghii3
Habenaria roxburghii2
Habenaria roxburghii5
Habenaria roxburghii4
Habenaria furcifera4
Habenaria furcifera6
Habenaria furcifera5
Habenaria crinifera2
Habenaria crinifera4
Habenaria crinifera3
Habenaria crinifera1
Habenaria longicorniculata1
Habenaria rhodocheila4
Habenaria rhodocheila3
Habenaria rhodocheila2
Habenaria rhodocheila1
Habenaria marginata1
Habenaria marginata5
Habenaria marginata4
Habenaria marginata3
Habenaria marginata2
Habenaria furcifera3
Habenaria furcifera2
Pecteilis susannae1
Habenaria roxburghii1
Habenaria dentata1
Habenaria dentata3
Habenaria dentata2
Habenaria fordii2
Habenaria fordii1
Habenaria commelinifolia2
Habenaria commelinifolia1
Habenaria ciliolaris2
Habenaria ciliolaris1
Habenaria delavayi2
Habenaria delavayi1
Habenaria linguella1
Pecteilis susannae2
Habenaria furcifera1

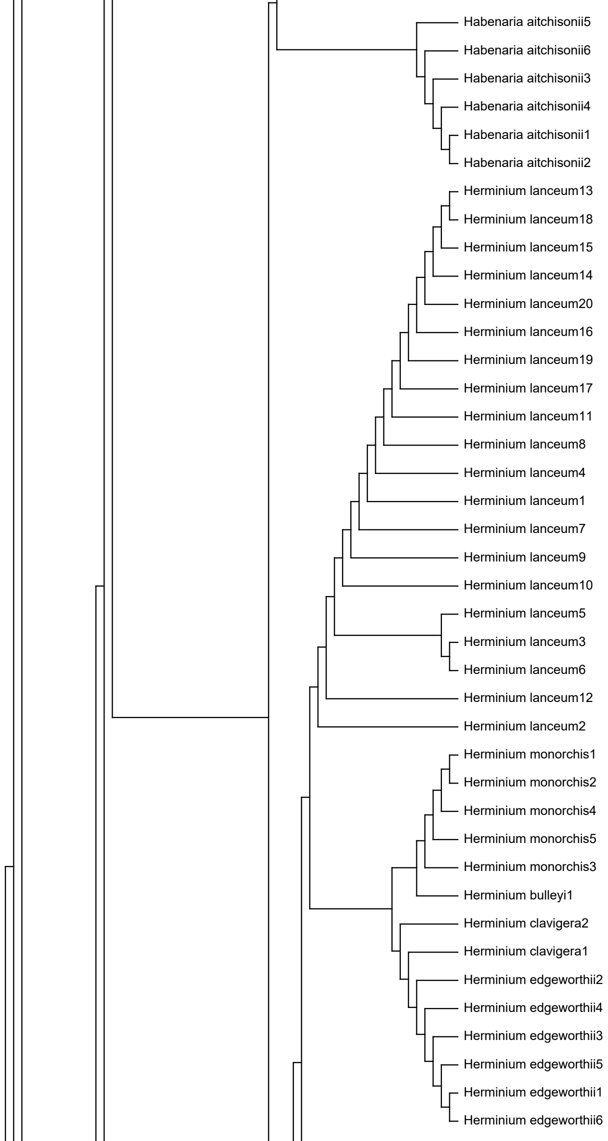
Habenaria aitchisonii5
Habenaria aitchisonii6
Habenaria aitchisonii3
Habenaria aitchisonii4
Habenaria aitchisonii1
Habenaria aitchisonii2
Herminium lanceum13
Herminium lanceum18
Herminium lanceum15
Herminium lanceum14
Herminium lanceum20
Herminium lanceum16
Herminium lanceum19
Herminium lanceum17
Herminium lanceum11
Herminium lanceum8
Herminium lanceum4
Herminium lanceum1
Herminium lanceum7
Herminium lanceum9
Herminium lanceum10
Herminium lanceum5
Herminium lanceum3
Herminium lanceum6
Herminium lanceum12
Herminium lanceum2
Herminium monorchis1
Herminium monorchis2
Herminium monorchis4
Herminium monorchis5
Herminium monorchis3
Herminium bulleyi1
Herminium clavigera2
Herminium clavigera1
Herminium edgeworthii2
Herminium edgeworthii4
Herminium edgeworthii3
Herminium edgeworthii5
Herminium edgeworthii1
Herminium edgeworthii6

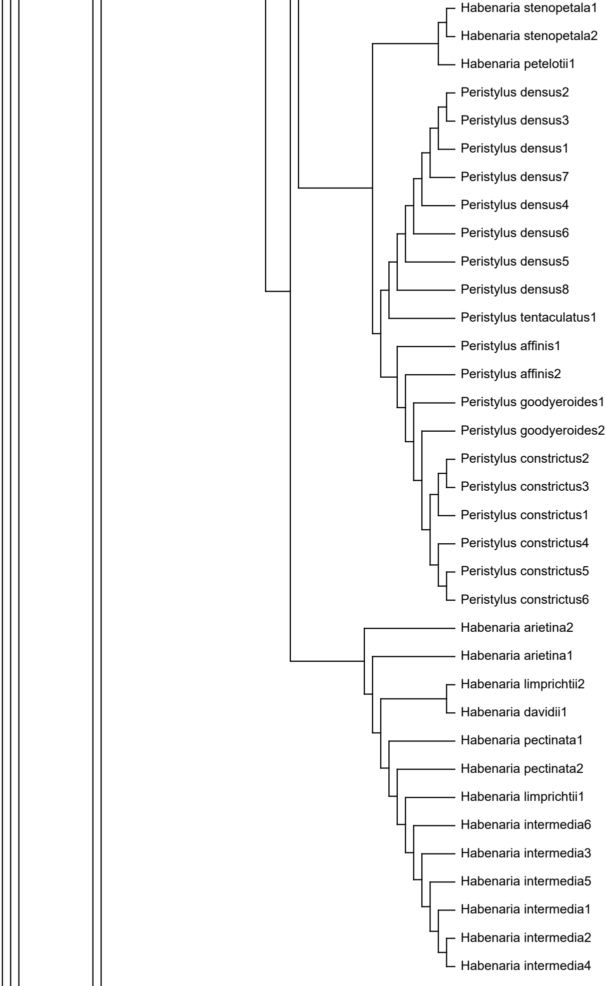
Habenaria stenopetala1
Habenaria stenopetala2
Habenaria petelotii1
Peristylus densus2
Peristylus densus3
Peristylus densus1
Peristylus densus7
Peristylus densus4
Peristylus densus6
Peristylus densus5
Peristylus densus8
Peristylus tentaculatus1
Peristylus affinis1
Peristylus affinis2
Peristylus goodyeroides1
Peristylus goodyeroides2
Peristylus constrictus2
Peristylus constrictus3
Peristylus constrictus1
Peristylus constrictus4
Peristylus constrictus5
Peristylus constrictus6
Habenaria arietina2
Habenaria arietina1
Habenaria limprichtii2
Habenaria davidii1
Habenaria pectinata1
Habenaria pectinata2
Habenaria limprichtii1
Habenaria intermedia6
Habenaria intermedia3
Habenaria intermedia5
Habenaria intermedia1
Habenaria intermedia2
Habenaria intermedia4

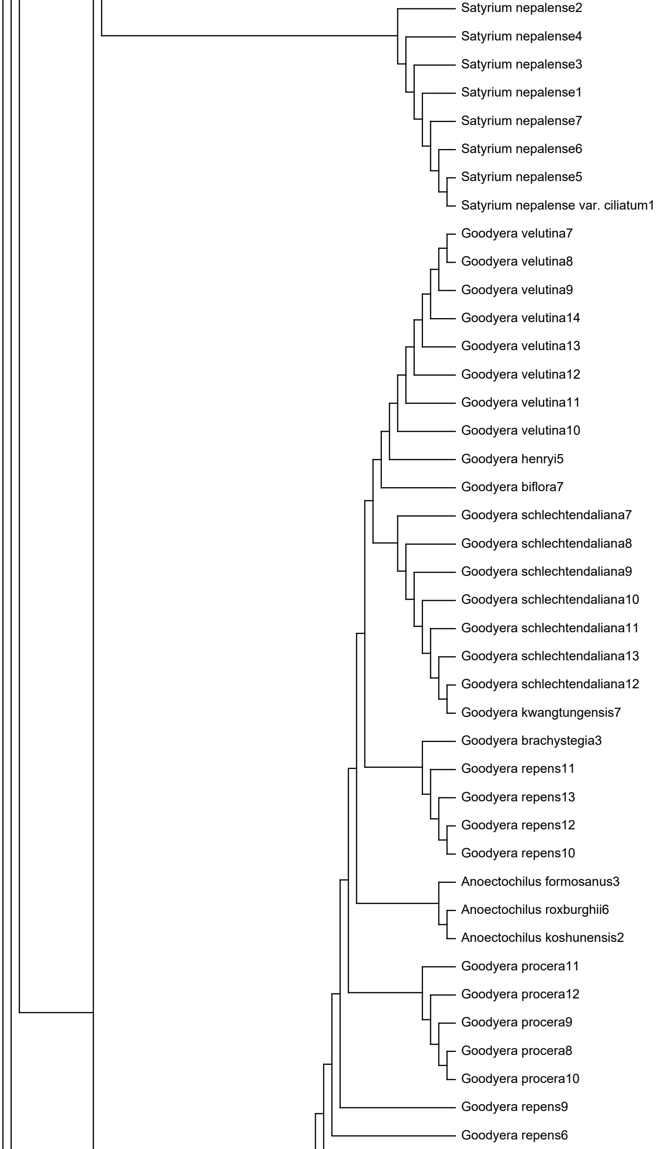
Satyrium nepalense2
Satyrium nepalense4
Satyrium nepalense3
Satyrium nepalense1
Satyrium nepalense7
Satyrium nepalense6
Satyrium nepalense5
Satyrium nepalense var. ciliatum1
Goodyera velutina7
Goodyera velutina8
Goodyera velutina9
Goodyera velutina14
Goodyera velutina13
Goodyera velutina12
Goodyera velutina11
Goodyera velutina10
Goodyera henryi5
Goodyera biflora7
Goodyera schlechtendaliana7
Goodyera schlechtendaliana8
Goodyera schlechtendaliana9
Goodyera schlechtendaliana10
Goodyera schlechtendaliana11
Goodyera schlechtendaliana13
Goodyera schlechtendaliana12
Goodyera kwangtungensis7
Goodyera brachystegia3
Goodyera repens11
Goodyera repens13
Goodyera repens12
Goodyera repens10
Anoectochilus formosanus3
Anoectochilus roxburghii6
Anoectochilus koshunensis2
Goodyera procera11
Goodyera procera12
Goodyera procera9
Goodyera procera8
Goodyera procera10
Goodyera repens9
Goodyera repens6

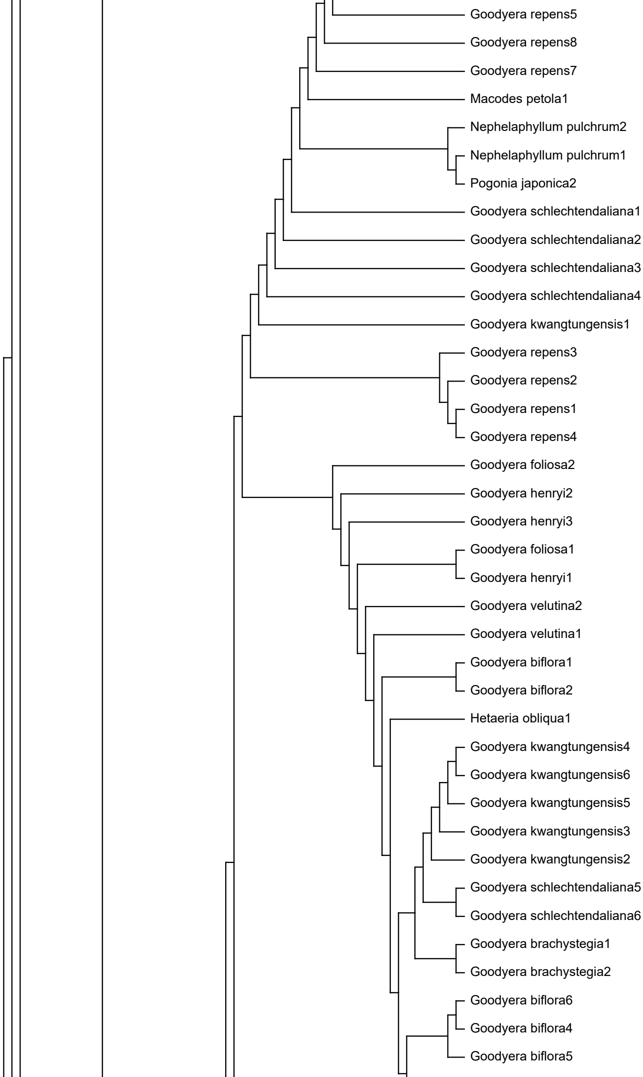
Goodyera repens5
Goodyera repens8
Goodyera repens7
Macodes petola1
Nephelaphyllum pulchrum2
Nephelaphyllum pulchrum1
Pogonia japonica2
Goodyera schlechtendaliana1
Goodyera schlechtendaliana2
Goodyera schlechtendaliana3
Goodyera schlechtendaliana4
Goodyera kwangtungensis1
Goodyera repens3
Goodyera repens2
Goodyera repens1
Goodyera repens4
Goodyera foliosa2
Goodyera henryi2
Goodyera henryi3
Goodyera foliosa1
Goodyera henryi1
Goodyera velutina2
Goodyera velutina1
Goodyera biflora1
Goodyera biflora2
Hetaeria obliqua1
Goodyera kwangtungensis4
Goodyera kwangtungensis6
Goodyera kwangtungensis5
Goodyera kwangtungensis3
Goodyera kwangtungensis2
Goodyera schlechtendaliana5
Goodyera schlechtendaliana6
Goodyera brachystegia1
Goodyera brachystegia2
Goodyera biflora6
Goodyera biflora4
Goodyera biflora5

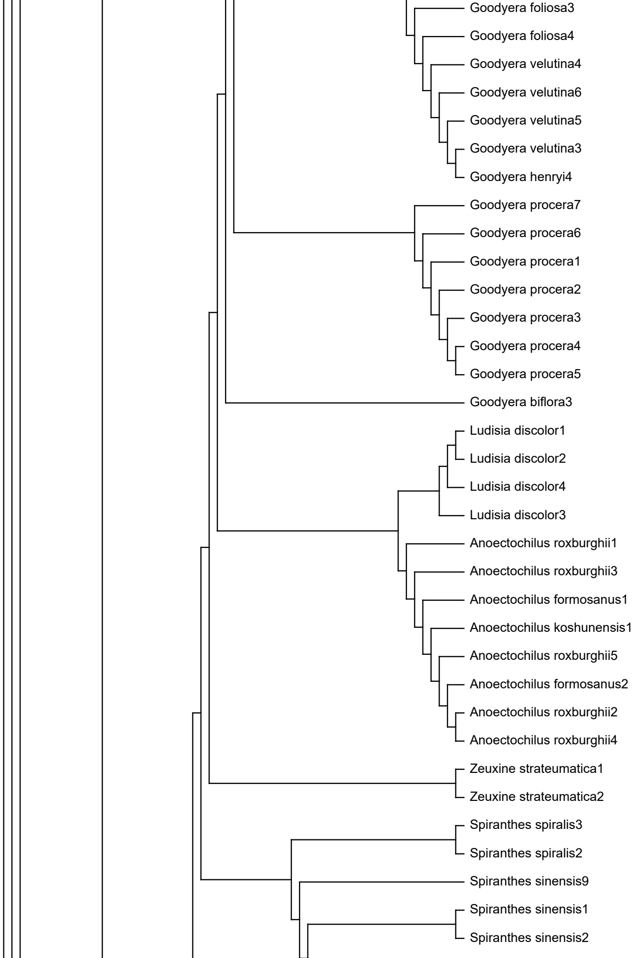
Goodyera foliosa3
Goodyera foliosa4
Goodyera velutina4
Goodyera velutina6
Goodyera velutina5
Goodyera velutina3
Goodyera henryi4
Goodyera procera7
Goodyera procera6
Goodyera procera1
Goodyera procera2
Goodyera procera3
Goodyera procera4
Goodyera procera5
Goodyera biflora3
Ludisia discolor1
Ludisia discolor2
Ludisia discolor4
Ludisia discolor3
Anoectochilus roxburghii1
Anoectochilus roxburghii3
Anoectochilus formosanus1
Anoectochilus koshunensis1
Anoectochilus roxburghii5
Anoectochilus formosanus2
Anoectochilus roxburghii2
Anoectochilus roxburghii4
Zeuxine strateumatica1
Zeuxine strateumatica2
Spiranthes spiralis3
Spiranthes spiralis2
Spiranthes sinensis9
Spiranthes sinensis1
Spiranthes sinensis2

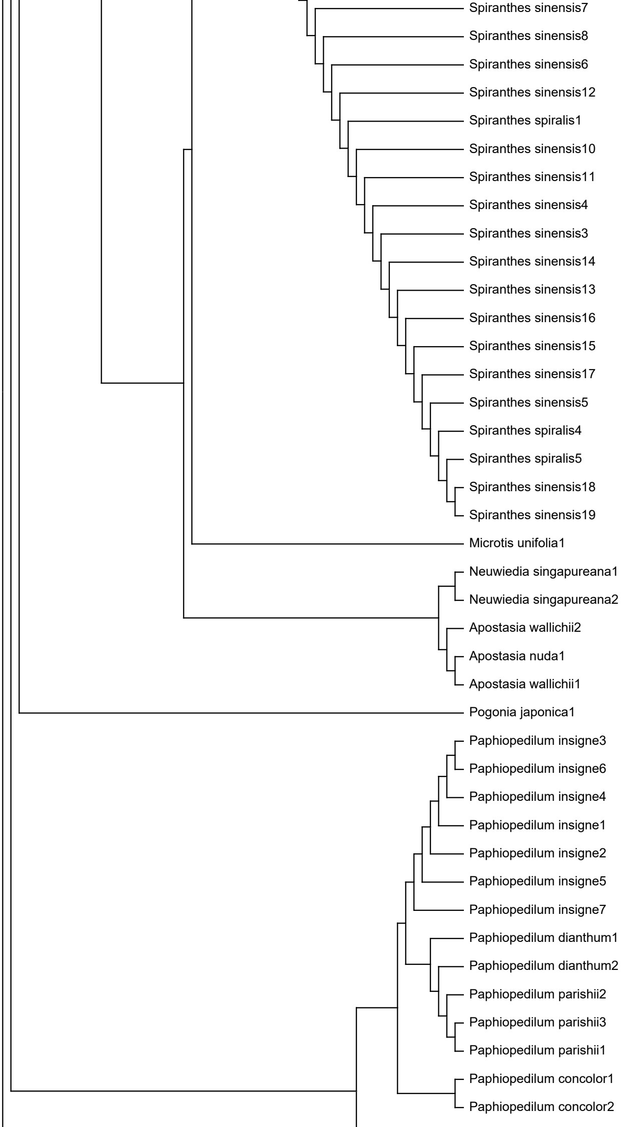
Spiranthes sinensis7
Spiranthes sinensis8
Spiranthes sinensis6
Spiranthes sinensis12
Spiranthes spiralis1
Spiranthes sinensis10
Spiranthes sinensis11
Spiranthes sinensis4
Spiranthes sinensis3
Spiranthes sinensis14
Spiranthes sinensis13
Spiranthes sinensis16
Spiranthes sinensis15
Spiranthes sinensis17
Spiranthes sinensis5
Spiranthes spiralis4
Spiranthes spiralis5
Spiranthes sinensis18
Spiranthes sinensis19
Microtis unifolia1
Neuwiedia singapureana1
Neuwiedia singapureana2
Apostasia wallichii2
Apostasia nuda1
Apostasia wallichii1
Pogonia japonica1
Paphiopedilum insigne3
Paphiopedilum insigne6
Paphiopedilum insigne4
Paphiopedilum insigne1
Paphiopedilum insigne2
Paphiopedilum insigne5
Paphiopedilum insigne7
Paphiopedilum dianthum1
Paphiopedilum dianthum2
Paphiopedilum parishii2
Paphiopedilum parishii3
Paphiopedilum parishii1
Paphiopedilum concolor1
Paphiopedilum concolor2

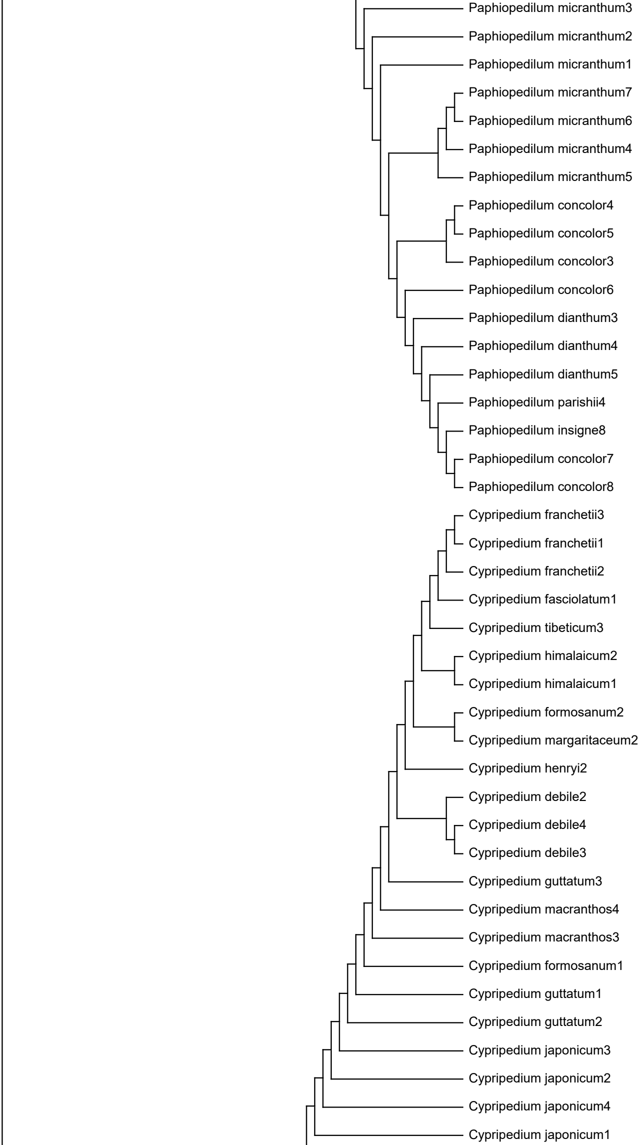
Paphiopedilum micranthum3
Paphiopedilum micranthum2
Paphiopedilum micranthum1
Paphiopedilum micranthum7
Paphiopedilum micranthum6
Paphiopedilum micranthum4
Paphiopedilum micranthum5
Paphiopedilum concolor4
Paphiopedilum concolor5
Paphiopedilum concolor3
Paphiopedilum concolor6
Paphiopedilum dianthum3
Paphiopedilum dianthum4
Paphiopedilum dianthum5
Paphiopedilum parishii4
Paphiopedilum insigne8
Paphiopedilum concolor7
Paphiopedilum concolor8
Cypripedium franchetii3
Cypripedium franchetii1
Cypripedium franchetii2
Cypripedium fasciolatum1
Cypripedium tibeticum3
Cypripedium himalaicum2
Cypripedium himalaicum1
Cypripedium formosanum2
Cypripedium margaritaceum2
Cypripedium henryi2
Cypripedium debile2
Cypripedium debile4
Cypripedium debile3
Cypripedium guttatum3
Cypripedium macranthos4
Cypripedium macranthos3
Cypripedium formosanum1
Cypripedium guttatum1
Cypripedium guttatum2
Cypripedium japonicum3
Cypripedium japonicum2
Cypripedium japonicum4
Cypripedium japonicum1

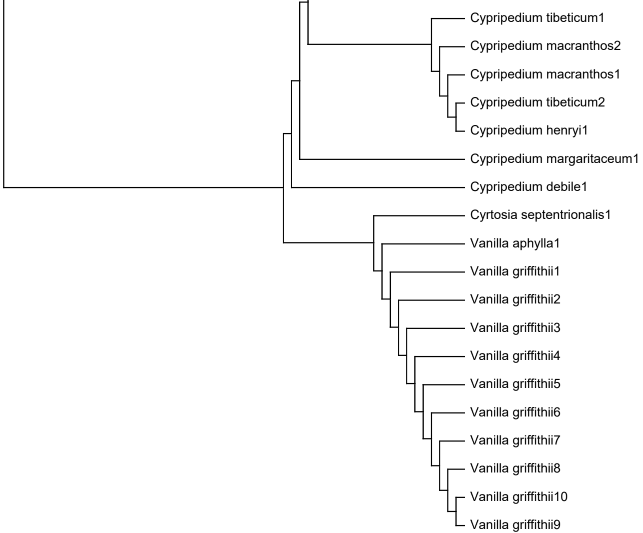

Cypripedium tibeticum1
Cypripedium macranthos2
Cypripedium macranthos1
Cypripedium tibeticum2
Cypripedium henryi1
Cypripedium margaritaceum1
Cypripedium debile1
Cyrtosia septentrionalis1
Vanilla aphylla1
Vanilla griffithii1
Vanilla griffithii2
Vanilla griffithii3
Vanilla griffithii4
Vanilla griffithii5
Vanilla griffithii6
Vanilla griffithii7
Vanilla griffithii8
Vanilla griffithii10
Vanilla griffithii9
