# Supplementary material for: DNA barcoding of medicinal orchids in Asia
Source: Sci Rep. 2021 Dec 8;11:23651. doi: 10.1038/s41598-021-03025-0 (PMC8654824; doi:10.1038/s41598-021-03025-0)
Supplement: Supplementary file 13 — Supplementary Figure S13. [file 41598_2021_3025_MOESM13_ESM.pdf]

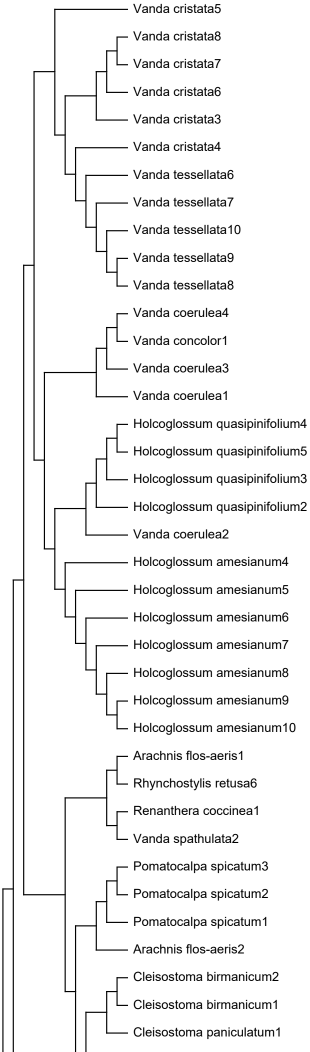
Vanda cristata5
Vanda cristata8
Vanda cristata7
Vanda cristata6
Vanda cristata3
Vanda cristata4
Vanda tessellata6
Vanda tessellata7
Vanda tessellata10
Vanda tessellata9
Vanda tessellata8
Vanda coerulea4
Vanda concolor1
Vanda coerulea3
Vanda coerulea1
Holcoglossum quasipinifolium4
Holcoglossum quasipinifolium5
Holcoglossum quasipinifolium3
Holcoglossum quasipinifolium2
Vanda coerulea2
Holcoglossum amesianum4
Holcoglossum amesianum5
Holcoglossum amesianum6
Holcoglossum amesianum7
Holcoglossum amesianum8
Holcoglossum amesianum9
Holcoglossum amesianum10
Arachnis flos-aeris1
Rhynchostylis retusa6
Renanthera coccinea1
Vanda spathulata2
Pomatocalpa spicatum3
Pomatocalpa spicatum2
Pomatocalpa spicatum1
Arachnis flos-aeris2
Cleisostoma birmanicum2
Cleisostoma birmanicum1
Cleisostoma paniculatum1

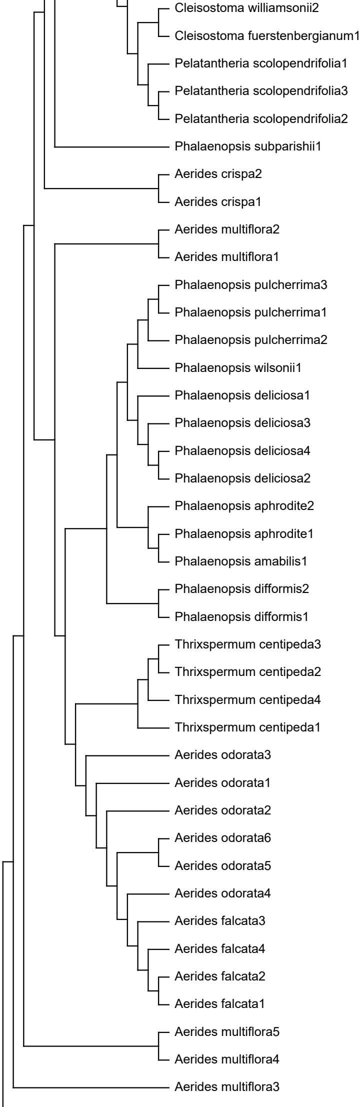
Cleisostoma williamsonii2
Cleisostoma fuerstenbergianum1
Pelatantheria scolopendrifolia1
Pelatantheria scolopendrifolia3
Pelatantheria scolopendrifolia2
Phalaenopsis subparishii1
Aerides crispa2
Aerides crispa1
Aerides multiflora2
Aerides multiflora1
Phalaenopsis pulcherrima3
Phalaenopsis pulcherrima1
Phalaenopsis pulcherrima2
Phalaenopsis wilsonii1
Phalaenopsis deliciosa1
Phalaenopsis deliciosa3
Phalaenopsis deliciosa4
Phalaenopsis deliciosa2
Phalaenopsis aphrodite2
Phalaenopsis aphrodite1
Phalaenopsis amabilis1
Phalaenopsis difformis2
Phalaenopsis difformis1
Thrixspermum centipeda3
Thrixspermum centipeda2
Thrixspermum centipeda4
Thrixspermum centipeda1
Aerides odorata3
Aerides odorata1
Aerides odorata2
Aerides odorata6
Aerides odorata5
Aerides odorata4
Aerides falcata3
Aerides falcata4
Aerides falcata2
Aerides falcata1
Aerides multiflora5
Aerides multiflora4
Aerides multiflora3

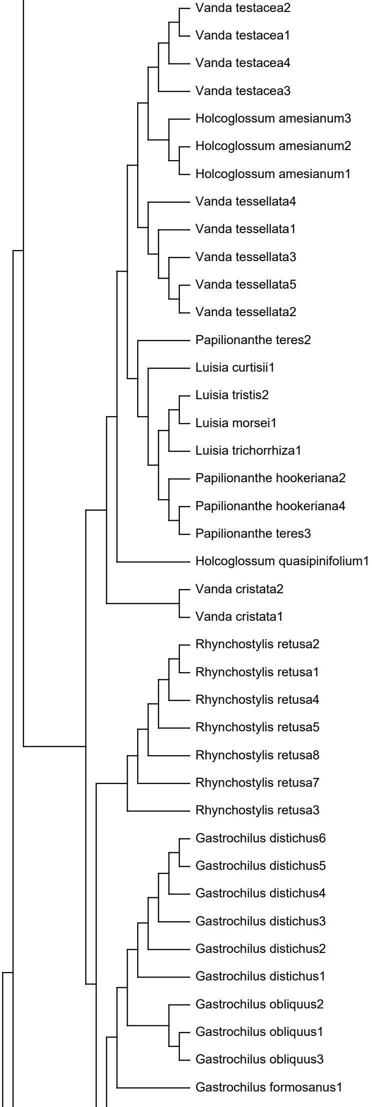
Vanda testacea2
Vanda testacea1
Vanda testacea4
Vanda testacea3
Holcoglossum amesianum3
Holcoglossum amesianum2
Holcoglossum amesianum1
Vanda tessellata4
Vanda tessellata1
Vanda tessellata3
Vanda tessellata5
Vanda tessellata2
Papilionanthe teres2
Luisia curtisii1
Luisia tristis2
Luisia morsei1
Luisia trichorrhiza1
Papilionanthe hookeriana2
Papilionanthe hookeriana4
Papilionanthe teres3
Holcoglossum quasipinifolium1
Vanda cristata2
Vanda cristata1
Rhynchostylis retusa2
Rhynchostylis retusa1
Rhynchostylis retusa4
Rhynchostylis retusa5
Rhynchostylis retusa8
Rhynchostylis retusa7
Rhynchostylis retusa3
Gastrochilus distichus6
Gastrochilus distichus5
Gastrochilus distichus4
Gastrochilus distichus3
Gastrochilus distichus2
Gastrochilus distichus1
Gastrochilus obliquus2
Gastrochilus obliquus1
Gastrochilus obliquus3
Gastrochilus formosanus1

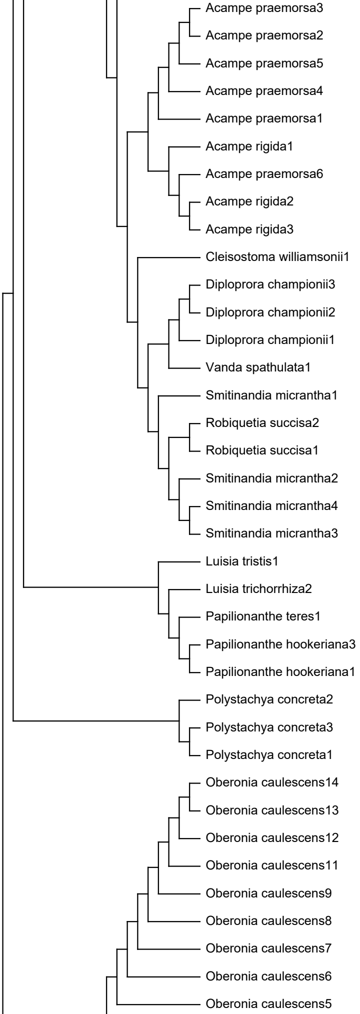
Acampe praemorsa3
Acampe praemorsa2
Acampe praemorsa5
Acampe praemorsa4
Acampe praemorsa1
Acampe rigida1
Acampe praemorsa6
Acampe rigida2
Acampe rigida3
Cleisostoma williamsonii1
Diploprora championii3
Diploprora championii2
Diploprora championii1
Vanda spathulata1
Smitinandia micrantha1
Robiquetia succisa2
Robiquetia succisa1
Smitinandia micrantha2
Smitinandia micrantha4
Smitinandia micrantha3
Luisia tristis1
Luisia trichorrhiza2
Papilionanthe teres1
Papilionanthe hookeriana3
Papilionanthe hookeriana1
Polystachya concreta2
Polystachya concreta3
Polystachya concreta1
Oberonia caulescens14
Oberonia caulescens13
Oberonia caulescens12
Oberonia caulescens11
Oberonia caulescens9
Oberonia caulescens8
Oberonia caulescens7
Oberonia caulescens6
Oberonia caulescens5

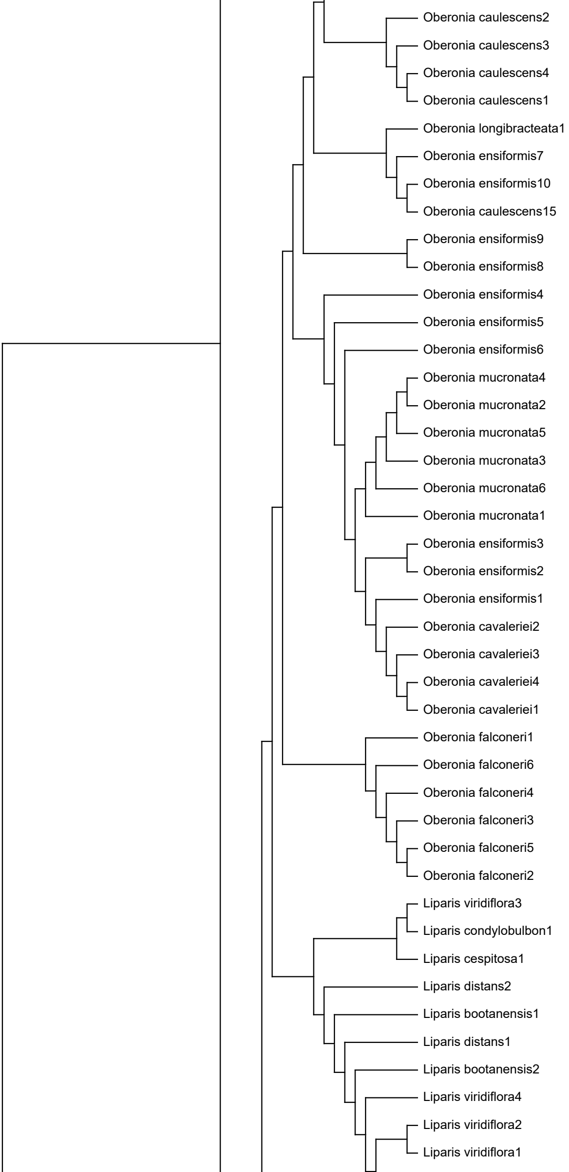
Oberonia caulescens2
Oberonia caulescens3
Oberonia caulescens4
Oberonia caulescens1
Oberonia longibracteata1
Oberonia ensiformis7
Oberonia ensiformis10
Oberonia caulescens15
Oberonia ensiformis9
Oberonia ensiformis8
Oberonia ensiformis4
Oberonia ensiformis5
Oberonia ensiformis6
Oberonia mucronata4
Oberonia mucronata2
Oberonia mucronata5
Oberonia mucronata3
Oberonia mucronata6
Oberonia mucronata1
Oberonia ensiformis3
Oberonia ensiformis2
Oberonia ensiformis1
Oberonia cavaleriei2
Oberonia cavaleriei3
Oberonia cavaleriei4
Oberonia cavaleriei1
Oberonia falconeri1
Oberonia falconeri6
Oberonia falconeri4
Oberonia falconeri3
Oberonia falconeri5
Oberonia falconeri2
Liparis viridiflora3
Liparis condylobulbon1
Liparis cespitosa1
Liparis distans2
Liparis bootanensis1
Liparis distans1
Liparis bootanensis2
Liparis viridiflora4
Liparis viridiflora2
Liparis viridiflora1

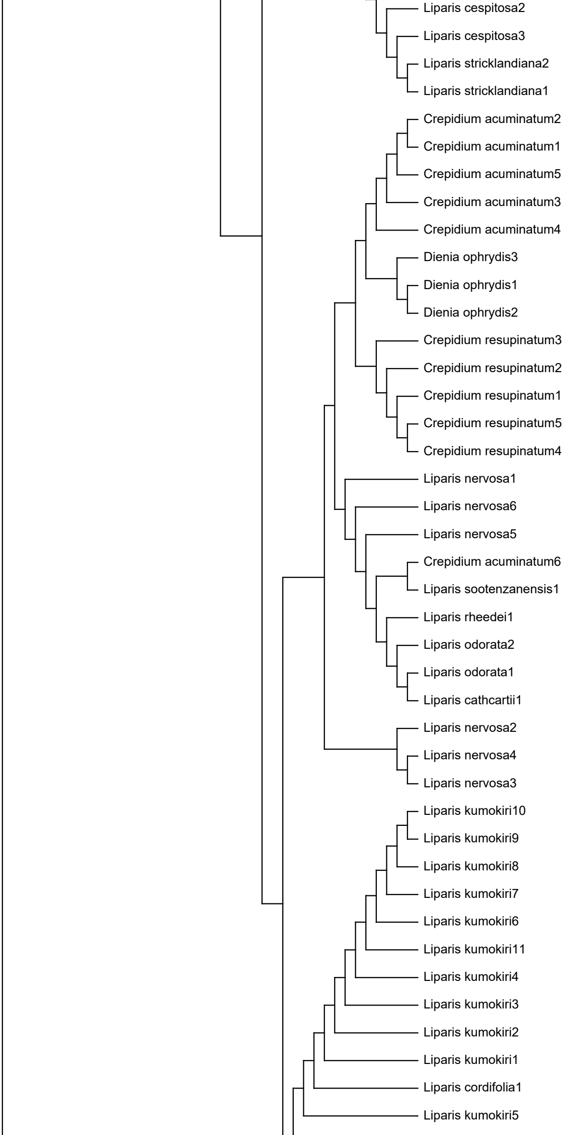
Liparis cespitosa2
Liparis cespitosa3
Liparis stricklandiana2
Liparis stricklandiana1
Crepidium acuminatum2
Crepidium acuminatum1
Crepidium acuminatum5
Crepidium acuminatum3
Crepidium acuminatum4
Dienia ophrydis3
Dienia ophrydis1
Dienia ophrydis2
Crepidium resupinatum3
Crepidium resupinatum2
Crepidium resupinatum1
Crepidium resupinatum5
Crepidium resupinatum4
Liparis nervosa1
Liparis nervosa6
Liparis nervosa5
Crepidium acuminatum6
Liparis sootenzanensis1
Liparis rheedei1
Liparis odorata2
Liparis odorata1
Liparis cathcartii1
Liparis nervosa2
Liparis nervosa4
Liparis nervosa3
Liparis kumokiri10
Liparis kumokiri9
Liparis kumokiri8
Liparis kumokiri7
Liparis kumokiri6
Liparis kumokiri11
Liparis kumokiri4
Liparis kumokiri3
Liparis kumokiri2
Liparis kumokiri1
Liparis cordifolia1
Liparis kumokiri5

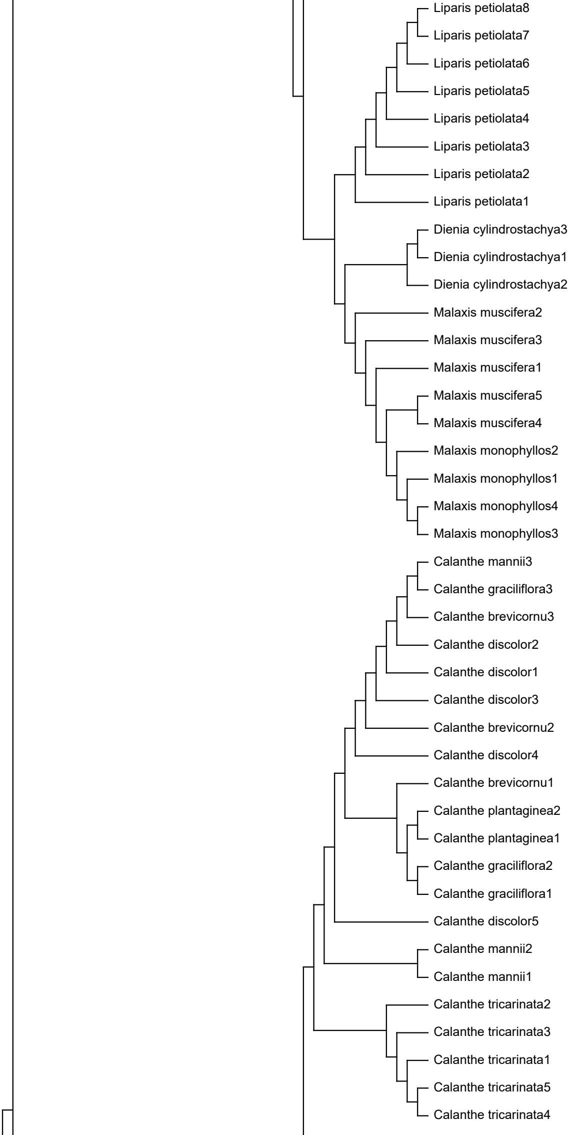
Liparis petiolata8
Liparis petiolata7
Liparis petiolata6
Liparis petiolata5
Liparis petiolata4
Liparis petiolata3
Liparis petiolata2
Liparis petiolata1
Dienia cylindrostachya3
Dienia cylindrostachya1
Dienia cylindrostachya2
Malaxis muscifera2
Malaxis muscifera3
Malaxis muscifera1
Malaxis muscifera5
Malaxis muscifera4
Malaxis monophyllos2
Malaxis monophyllos1
Malaxis monophyllos4
Malaxis monophyllos3
Calanthe mannii3
Calanthe graciliflora3
Calanthe brevicornu3
Calanthe discolor2
Calanthe discolor1
Calanthe discolor3
Calanthe brevicornu2
Calanthe discolor4
Calanthe brevicornu1
Calanthe plantaginea2
Calanthe plantaginea1
Calanthe graciliflora2
Calanthe graciliflora1
Calanthe discolor5
Calanthe mannii2
Calanthe mannii1
Calanthe tricarinata2
Calanthe tricarinata3
Calanthe tricarinata1
Calanthe tricarinata5
Calanthe tricarinata4

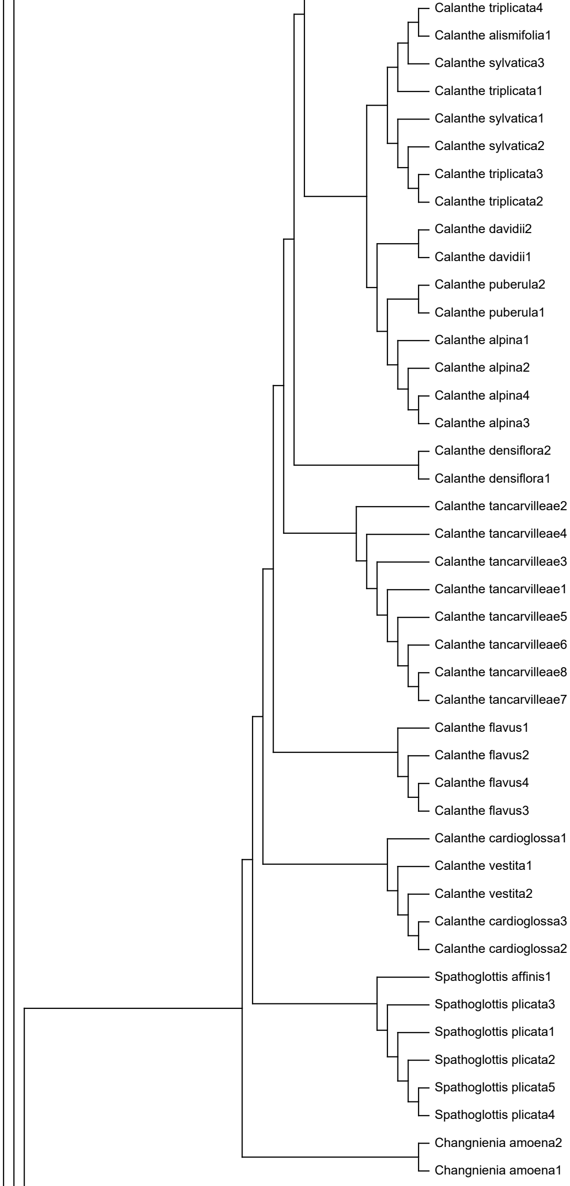
Calanthe triplicata4
Calanthe alismifolia1
Calanthe sylvatica3
Calanthe triplicata1
Calanthe sylvatica1
Calanthe sylvatica2
Calanthe triplicata3
Calanthe triplicata2
Calanthe davidii2
Calanthe davidii1
Calanthe puberula2
Calanthe puberula1
Calanthe alpina1
Calanthe alpina2
Calanthe alpina4
Calanthe alpina3
Calanthe densiflora2
Calanthe densiflora1
Calanthe tancarvilleae2
Calanthe tancarvilleae4
Calanthe tancarvilleae3
Calanthe tancarvilleae1
Calanthe tancarvilleae5
Calanthe tancarvilleae6
Calanthe tancarvilleae8
Calanthe tancarvilleae7
Calanthe flavus1
Calanthe flavus2
Calanthe flavus4
Calanthe flavus3
Calanthe cardioglossa1
Calanthe vestita1
Calanthe vestita2
Calanthe cardioglossa3
Calanthe cardioglossa2
Spathoglottis affinis1
Spathoglottis plicata3
Spathoglottis plicata1
Spathoglottis plicata2
Spathoglottis plicata5
Spathoglottis plicata4
Changnienia amoena2
Changnienia amoena1

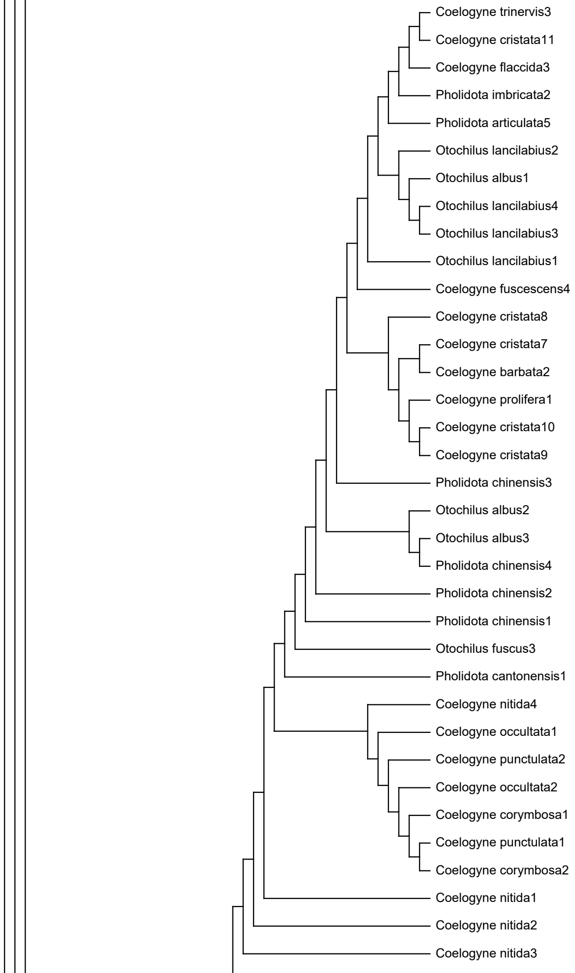
Coelogyne trinervis3
Coelogyne cristata11
Coelogyne flaccida3
Pholidota imbricata2
Pholidota articulata5
Otochilus lancilabius2
Otochilus albus1
Otochilus lancilabius4
Otochilus lancilabius3
Otochilus lancilabius1
Coelogyne fuscescens4
Coelogyne cristata8
Coelogyne cristata7
Coelogyne barbata2
Coelogyne prolifera1
Coelogyne cristata10
Coelogyne cristata9
Pholidota chinensis3
Otochilus albus2
Otochilus albus3
Pholidota chinensis4
Pholidota chinensis2
Pholidota chinensis1
Otochilus fuscus3
Pholidota cantonensis1
Coelogyne nitida4
Coelogyne occultata1
Coelogyne punctulata2
Coelogyne occultata2
Coelogyne corymbosa1
Coelogyne punctulata1
Coelogyne corymbosa2
Coelogyne nitida1
Coelogyne nitida2
Coelogyne nitida3

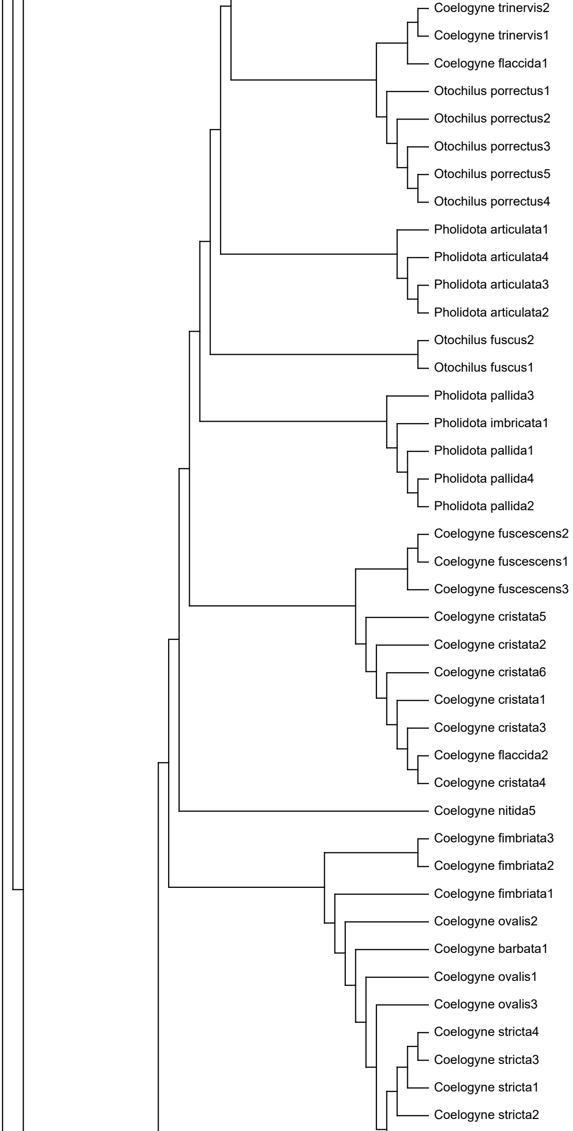
Coelogyne trinervis2
Coelogyne trinervis1
Coelogyne flaccida1
Otochilus porrectus1
Otochilus porrectus2
Otochilus porrectus3
Otochilus porrectus5
Otochilus porrectus4
Pholidota articulata1
Pholidota articulata4
Pholidota articulata3
Pholidota articulata2
Otochilus fuscus2
Otochilus fuscus1
Pholidota pallida3
Pholidota imbricata1
Pholidota pallida1
Pholidota pallida4
Pholidota pallida2
Coelogyne fuscescens2
Coelogyne fuscescens1
Coelogyne fuscescens3
Coelogyne cristata5
Coelogyne cristata2
Coelogyne cristata6
Coelogyne cristata1
Coelogyne cristata3
Coelogyne flaccida2
Coelogyne cristata4
Coelogyne nitida5
Coelogyne fimbriata3
Coelogyne fimbriata2
Coelogyne fimbriata1
Coelogyne ovalis2
Coelogyne barbata1
Coelogyne ovalis1
Coelogyne ovalis3
Coelogyne stricta4
Coelogyne stricta3
Coelogyne stricta1
Coelogyne stricta2

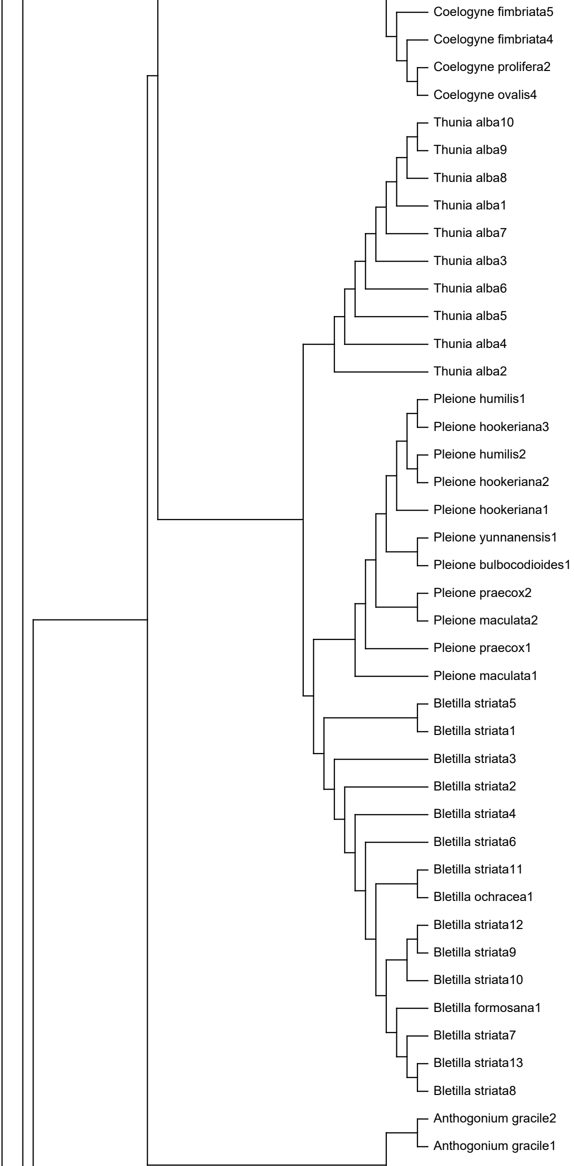
Coelogyne fimbriata5
Coelogyne fimbriata4
Coelogyne prolifera2
Coelogyne ovalis4
Thunia alba10
Thunia alba9
Thunia alba8
Thunia alba1
Thunia alba7
Thunia alba3
Thunia alba6
Thunia alba5
Thunia alba4
Thunia alba2
Pleione humilis1
Pleione hookeriana3
Pleione humilis2
Pleione hookeriana2
Pleione hookeriana1
Pleione yunnanensis1
Pleione bulbocodioides1
Pleione praecox2
Pleione maculata2
Pleione praecox1
Pleione maculata1
Bletilla striata5
Bletilla striata1
Bletilla striata3
Bletilla striata2
Bletilla striata4
Bletilla striata6
Bletilla striata11
Bletilla ochracea1
Bletilla striata12
Bletilla striata9
Bletilla striata10
Bletilla formosana1
Bletilla striata7
Bletilla striata13
Bletilla striata8
Anthogonium gracile2
Anthogonium gracile1

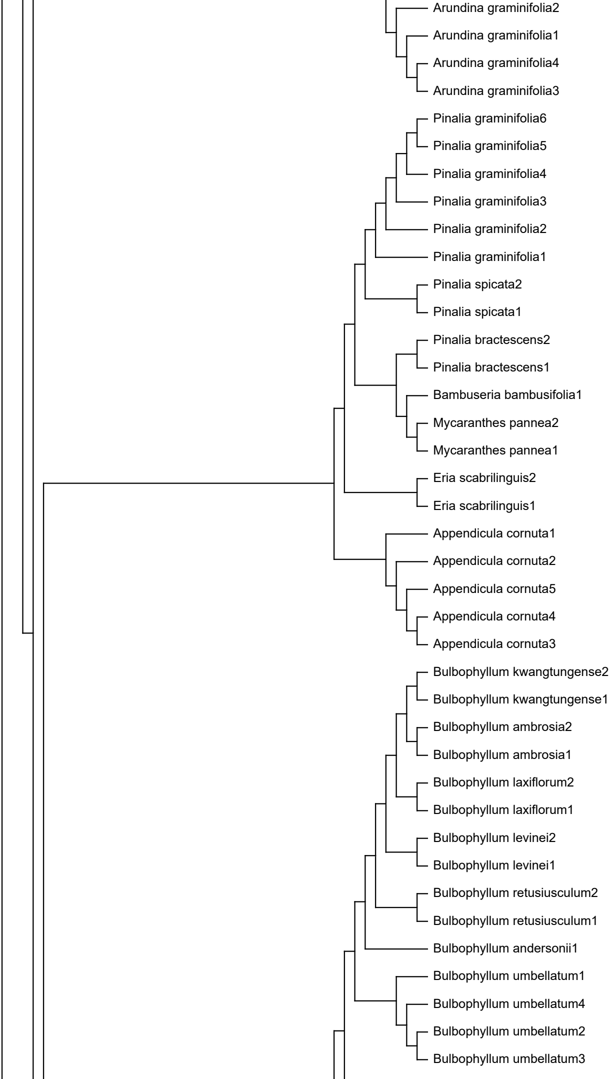
Arundina graminifolia2
Arundina graminifolia1
Arundina graminifolia4
Arundina graminifolia3
Pinalia graminifolia6
Pinalia graminifolia5
Pinalia graminifolia4
Pinalia graminifolia3
Pinalia graminifolia2
Pinalia graminifolia1
Pinalia spicata2
Pinalia spicata1
Pinalia bractescens2
Pinalia bractescens1
Bambuseria bambusifolia1
Mycaranthes pannea2
Mycaranthes pannea1
Eria scabrilinguis2
Eria scabrilinguis1
Appendicula cornuta1
Appendicula cornuta2
Appendicula cornuta5
Appendicula cornuta4
Appendicula cornuta3
Bulbophyllum kwangtungense2
Bulbophyllum kwangtungense1
Bulbophyllum ambrosia2
Bulbophyllum ambrosia1
Bulbophyllum laxiflorum2
Bulbophyllum laxiflorum1
Bulbophyllum levinei2
Bulbophyllum levinei1
Bulbophyllum retusiusculum2
Bulbophyllum retusiusculum1
Bulbophyllum andersonii1
Bulbophyllum umbellatum1
Bulbophyllum umbellatum4
Bulbophyllum umbellatum2
Bulbophyllum umbellatum3

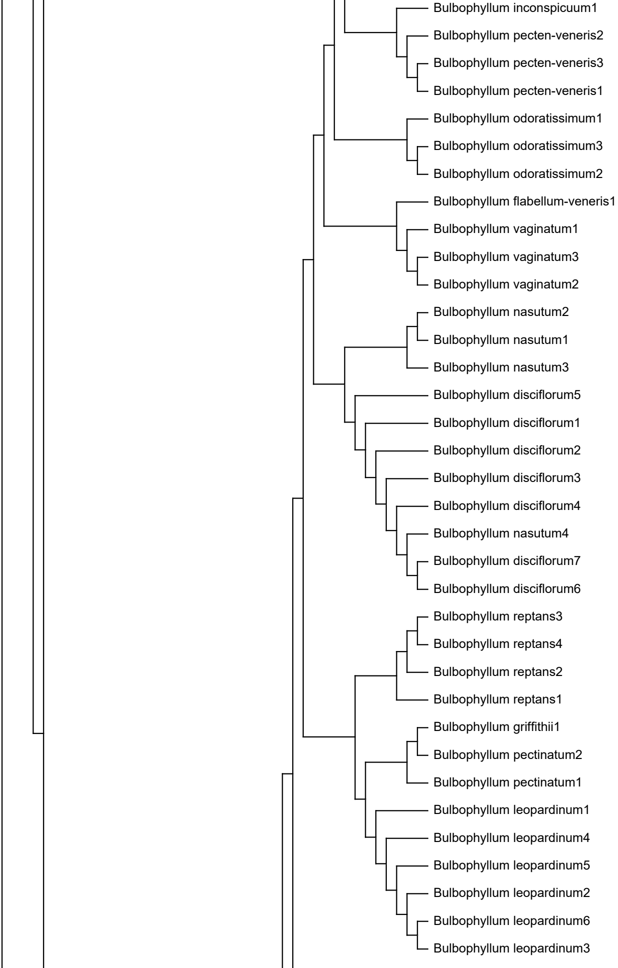
Bulbophyllum inconspicuum1
Bulbophyllum pecten-veneris2
Bulbophyllum pecten-veneris3
Bulbophyllum pecten-veneris1
Bulbophyllum odoratissimum1
Bulbophyllum odoratissimum3
Bulbophyllum odoratissimum2
Bulbophyllum flabellum-veneris1
Bulbophyllum vaginatum1
Bulbophyllum vaginatum3
Bulbophyllum vaginatum2
Bulbophyllum nasutum2
Bulbophyllum nasutum1
Bulbophyllum nasutum3
Bulbophyllum disciflorum5
Bulbophyllum disciflorum1
Bulbophyllum disciflorum2
Bulbophyllum disciflorum3
Bulbophyllum disciflorum4
Bulbophyllum nasutum4
Bulbophyllum disciflorum7
Bulbophyllum disciflorum6
Bulbophyllum reptans3
Bulbophyllum reptans4
Bulbophyllum reptans2
Bulbophyllum reptans1
Bulbophyllum griffithii1
Bulbophyllum pectinatum2
Bulbophyllum pectinatum1
Bulbophyllum leopardinum1
Bulbophyllum leopardinum4
Bulbophyllum leopardinum5
Bulbophyllum leopardinum2
Bulbophyllum leopardinum6
Bulbophyllum leopardinum3

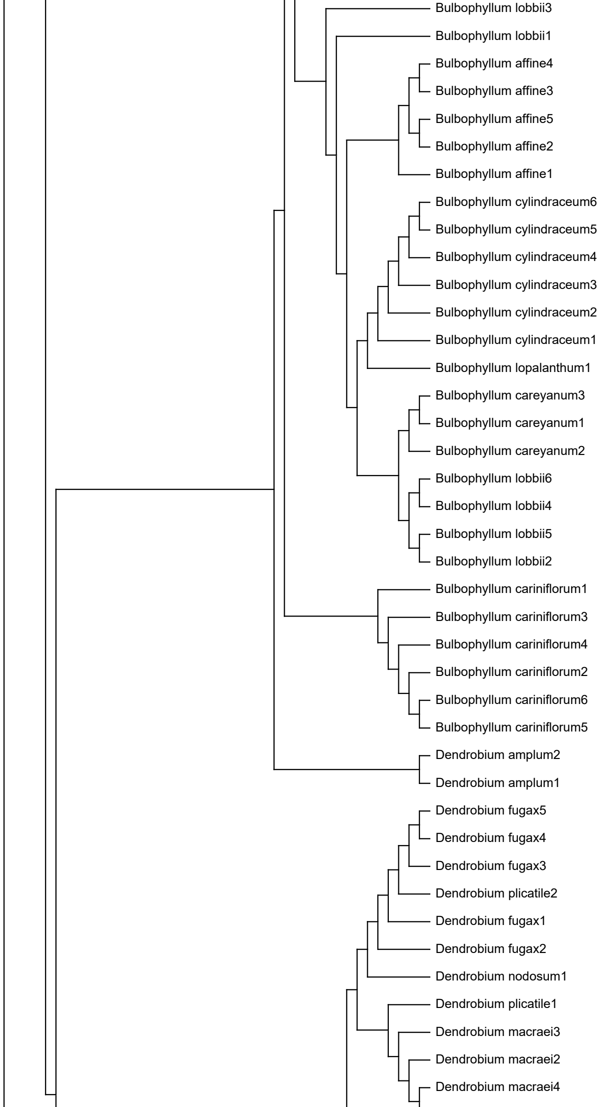
Bulbophyllum lobbii3
Bulbophyllum lobbii1
Bulbophyllum affine4
Bulbophyllum affine3
Bulbophyllum affine5
Bulbophyllum affine2
Bulbophyllum affine1
Bulbophyllum cylindraceum6
Bulbophyllum cylindraceum5
Bulbophyllum cylindraceum4
Bulbophyllum cylindraceum3
Bulbophyllum cylindraceum2
Bulbophyllum cylindraceum1
Bulbophyllum lopalanthum1
Bulbophyllum careyanum3
Bulbophyllum careyanum1
Bulbophyllum careyanum2
Bulbophyllum lobbii6
Bulbophyllum lobbii4
Bulbophyllum lobbii5
Bulbophyllum lobbii2
Bulbophyllum cariniflorum1
Bulbophyllum cariniflorum3
Bulbophyllum cariniflorum4
Bulbophyllum cariniflorum2
Bulbophyllum cariniflorum6
Bulbophyllum cariniflorum5
Dendrobium amplum2
Dendrobium amplum1
Dendrobium fugax5
Dendrobium fugax4
Dendrobium fugax3
Dendrobium plicatile2
Dendrobium fugax1
Dendrobium fugax2
Dendrobium nodosum1
Dendrobium plicatile1
Dendrobium macraei3
Dendrobium macraei2
Dendrobium macraei4

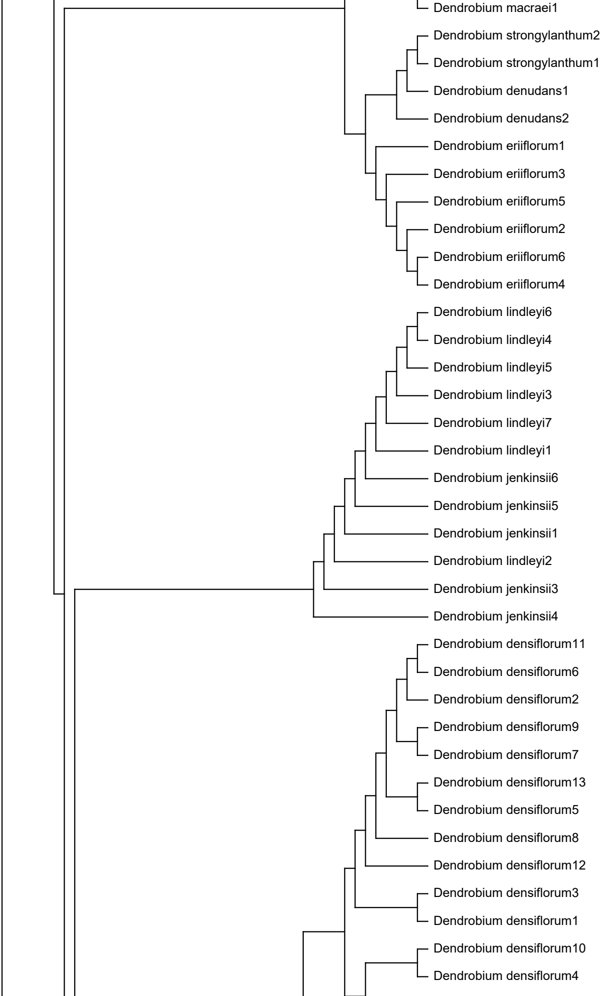
Dendrobium macraei1
Dendrobium strongylanthum2
Dendrobium strongylanthum1
Dendrobium denudans1
Dendrobium denudans2
Dendrobium eriiflorum1
Dendrobium eriiflorum3
Dendrobium eriiflorum5
Dendrobium eriiflorum2
Dendrobium eriiflorum6
Dendrobium eriiflorum4
Dendrobium lindleyi6
Dendrobium lindleyi4
Dendrobium lindleyi5
Dendrobium lindleyi3
Dendrobium lindleyi7
Dendrobium lindleyi1
Dendrobium jenkinsii6
Dendrobium jenkinsii5
Dendrobium jenkinsii1
Dendrobium lindleyi2
Dendrobium jenkinsii3
Dendrobium jenkinsii4
Dendrobium densiflorum11
Dendrobium densiflorum6
Dendrobium densiflorum2
Dendrobium densiflorum9
Dendrobium densiflorum7
Dendrobium densiflorum13
Dendrobium densiflorum5
Dendrobium densiflorum8
Dendrobium densiflorum12
Dendrobium densiflorum3
Dendrobium densiflorum1
Dendrobium densiflorum10
Dendrobium densiflorum4

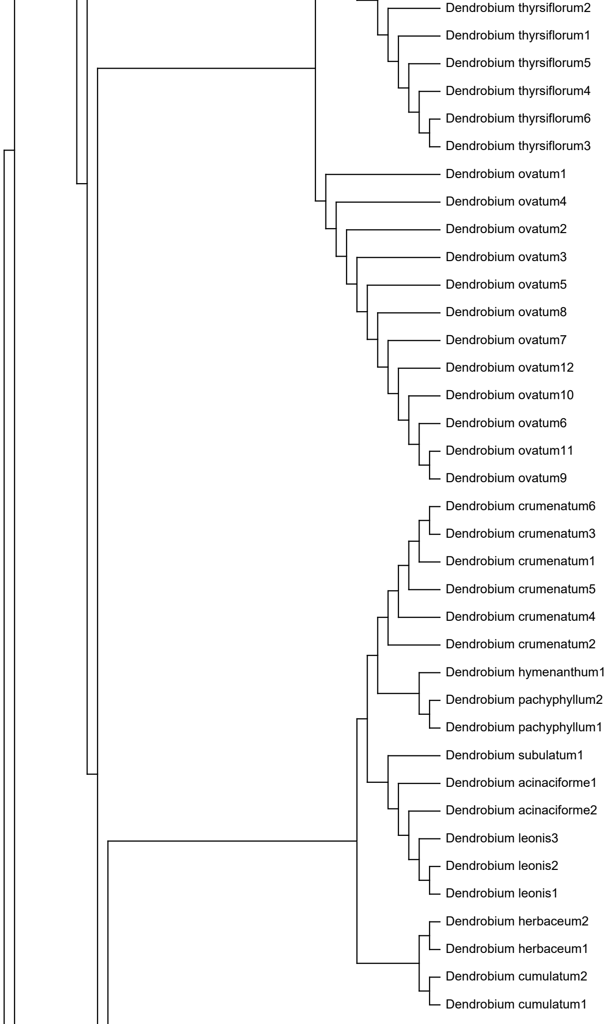
Dendrobium thyrsiflorum2
Dendrobium thyrsiflorum1
Dendrobium thyrsiflorum5
Dendrobium thyrsiflorum4
Dendrobium thyrsiflorum6
Dendrobium thyrsiflorum3
Dendrobium ovatum1
Dendrobium ovatum4
Dendrobium ovatum2
Dendrobium ovatum3
Dendrobium ovatum5
Dendrobium ovatum8
Dendrobium ovatum7
Dendrobium ovatum12
Dendrobium ovatum10
Dendrobium ovatum6
Dendrobium ovatum11
Dendrobium ovatum9
Dendrobium crumenatum6
Dendrobium crumenatum3
Dendrobium crumenatum1
Dendrobium crumenatum5
Dendrobium crumenatum4
Dendrobium crumenatum2
Dendrobium hymenanthum1
Dendrobium pachyphyllum2
Dendrobium pachyphyllum1
Dendrobium subulatum1
Dendrobium acinaciforme1
Dendrobium acinaciforme2
Dendrobium leonis3
Dendrobium leonis2
Dendrobium leonis1
Dendrobium herbaceum2
Dendrobium herbaceum1
Dendrobium cumulatum2
Dendrobium cumulatum1

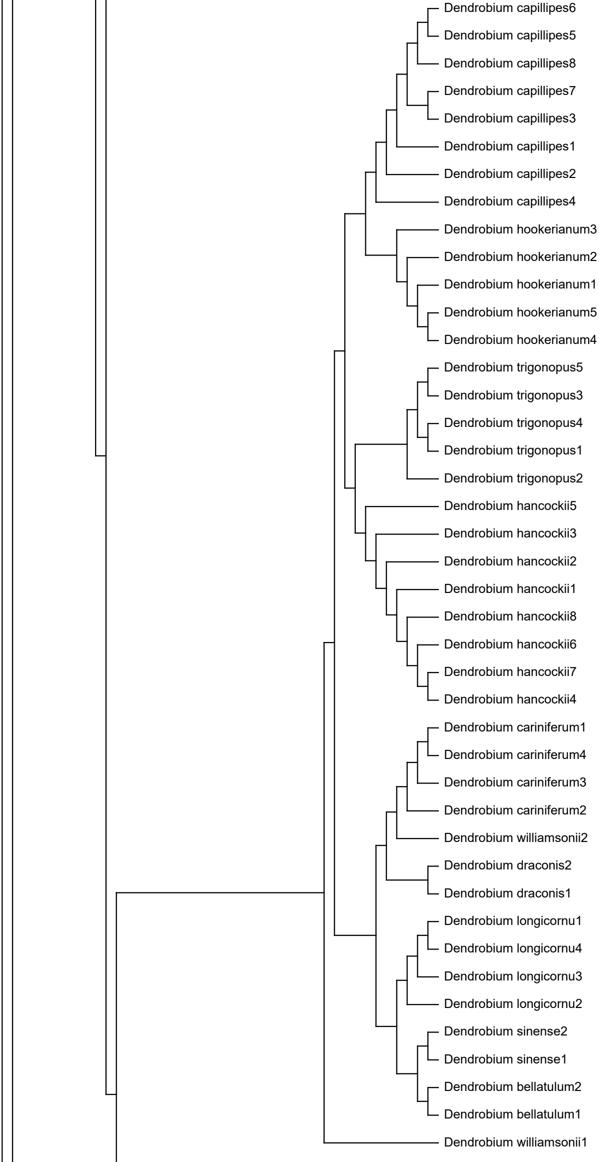
Dendrobium capillipes6
Dendrobium capillipes5
Dendrobium capillipes8
Dendrobium capillipes7
Dendrobium capillipes3
Dendrobium capillipes1
Dendrobium capillipes2
Dendrobium capillipes4
Dendrobium hookerianum3
Dendrobium hookerianum2
Dendrobium hookerianum1
Dendrobium hookerianum5
Dendrobium hookerianum4
Dendrobium trigonopus5
Dendrobium trigonopus3
Dendrobium trigonopus4
Dendrobium trigonopus1
Dendrobium trigonopus2
Dendrobium hancockii5
Dendrobium hancockii3
Dendrobium hancockii2
Dendrobium hancockii1
Dendrobium hancockii8
Dendrobium hancockii6
Dendrobium hancockii7
Dendrobium hancockii4
Dendrobium cariniferum1
Dendrobium cariniferum4
Dendrobium cariniferum3
Dendrobium cariniferum2
Dendrobium williamsonii2
Dendrobium draconis2
Dendrobium draconis1
Dendrobium longicornu1
Dendrobium longicornu4
Dendrobium longicornu3
Dendrobium longicornu2
Dendrobium sinense2
Dendrobium sinense1
Dendrobium bellatulum2
Dendrobium bellatulum1
Dendrobium williamsonii1

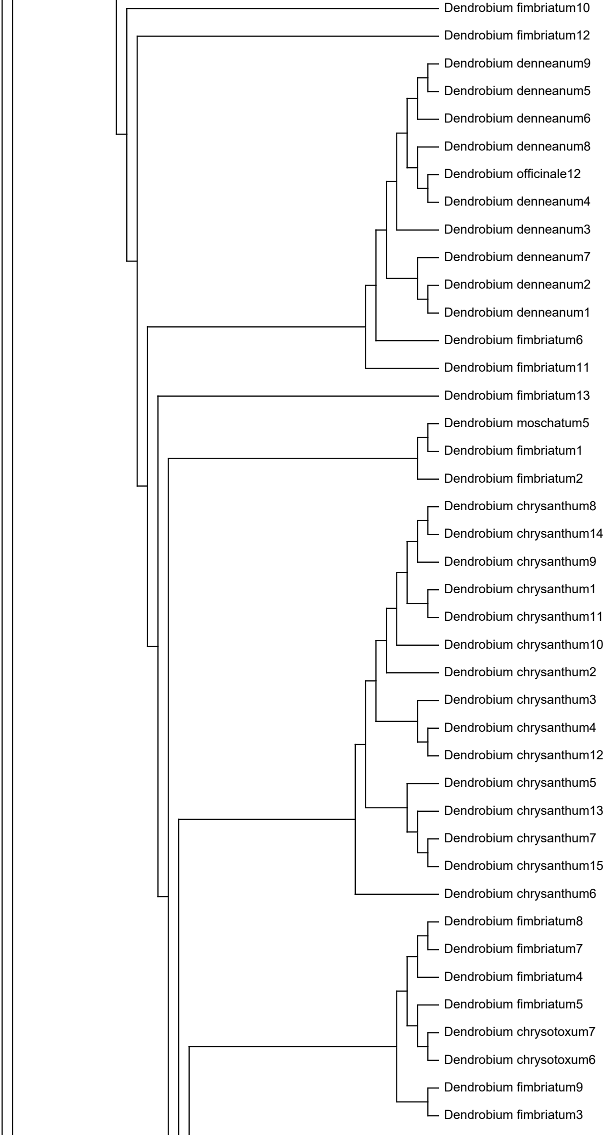

Dendrobium fimbriatum10
Dendrobium fimbriatum12
Dendrobium denneanum9
Dendrobium denneanum5
Dendrobium denneanum6
Dendrobium denneanum8
Dendrobium officinale12
Dendrobium denneanum4
Dendrobium denneanum3
Dendrobium denneanum7
Dendrobium denneanum2
Dendrobium denneanum1
Dendrobium fimbriatum6
Dendrobium fimbriatum11
Dendrobium fimbriatum13
Dendrobium moschatum5
Dendrobium fimbriatum1
Dendrobium fimbriatum2
Dendrobium chrysanthum8
Dendrobium chrysanthum14
Dendrobium chrysanthum9
Dendrobium chrysanthum1
Dendrobium chrysanthum11
Dendrobium chrysanthum10
Dendrobium chrysanthum2
Dendrobium chrysanthum3
Dendrobium chrysanthum4
Dendrobium chrysanthum12
Dendrobium chrysanthum5
Dendrobium chrysanthum13
Dendrobium chrysanthum7
Dendrobium chrysanthum15
Dendrobium chrysanthum6
Dendrobium fimbriatum8
Dendrobium fimbriatum7
Dendrobium fimbriatum4
Dendrobium fimbriatum5
Dendrobium chrysotoxum7
Dendrobium chrysotoxum6
Dendrobium fimbriatum9
Dendrobium fimbriatum3

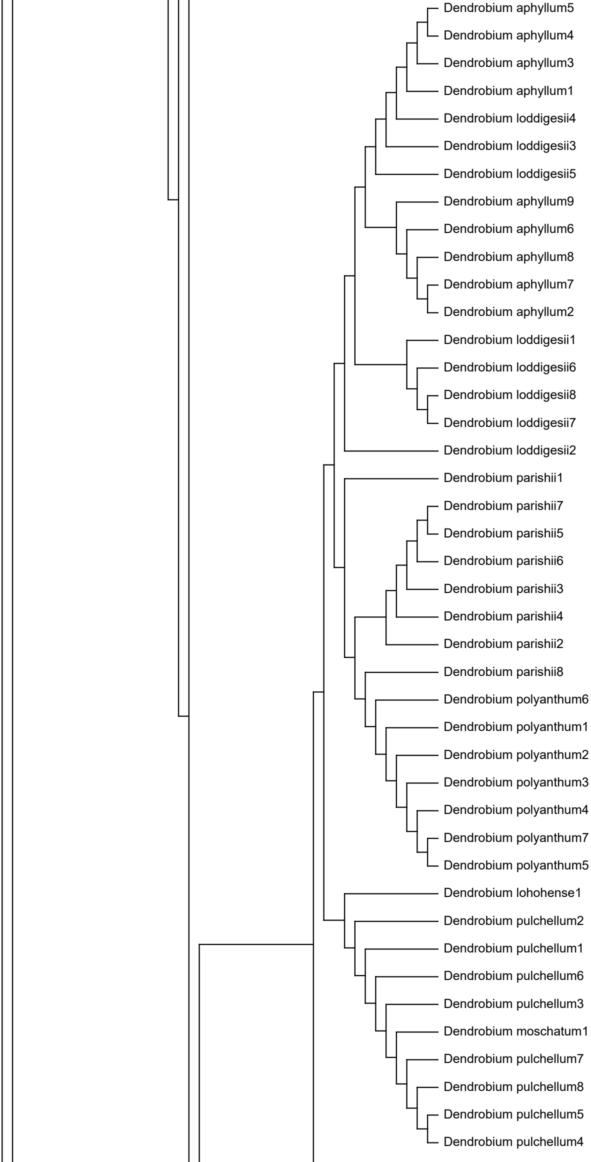

Dendrobium aphyllum5
Dendrobium aphyllum4
Dendrobium aphyllum3
Dendrobium aphyllum1
Dendrobium loddigesii4
Dendrobium loddigesii3
Dendrobium loddigesii5
Dendrobium aphyllum9
Dendrobium aphyllum6
Dendrobium aphyllum8
Dendrobium aphyllum7
Dendrobium aphyllum2
Dendrobium loddigesii1
Dendrobium loddigesii6
Dendrobium loddigesii8
Dendrobium loddigesii7
Dendrobium loddigesii2
Dendrobium parishii1
Dendrobium parishii7
Dendrobium parishii5
Dendrobium parishii6
Dendrobium parishii3
Dendrobium parishii4
Dendrobium parishii2
Dendrobium parishii8
Dendrobium polyanthum6
Dendrobium polyanthum1
Dendrobium polyanthum2
Dendrobium polyanthum3
Dendrobium polyanthum4
Dendrobium polyanthum7
Dendrobium polyanthum5
Dendrobium lohohense1
Dendrobium pulchellum2
Dendrobium pulchellum1
Dendrobium pulchellum6
Dendrobium pulchellum3
Dendrobium moschatum1
Dendrobium pulchellum7
Dendrobium pulchellum8
Dendrobium pulchellum5
Dendrobium pulchellum4

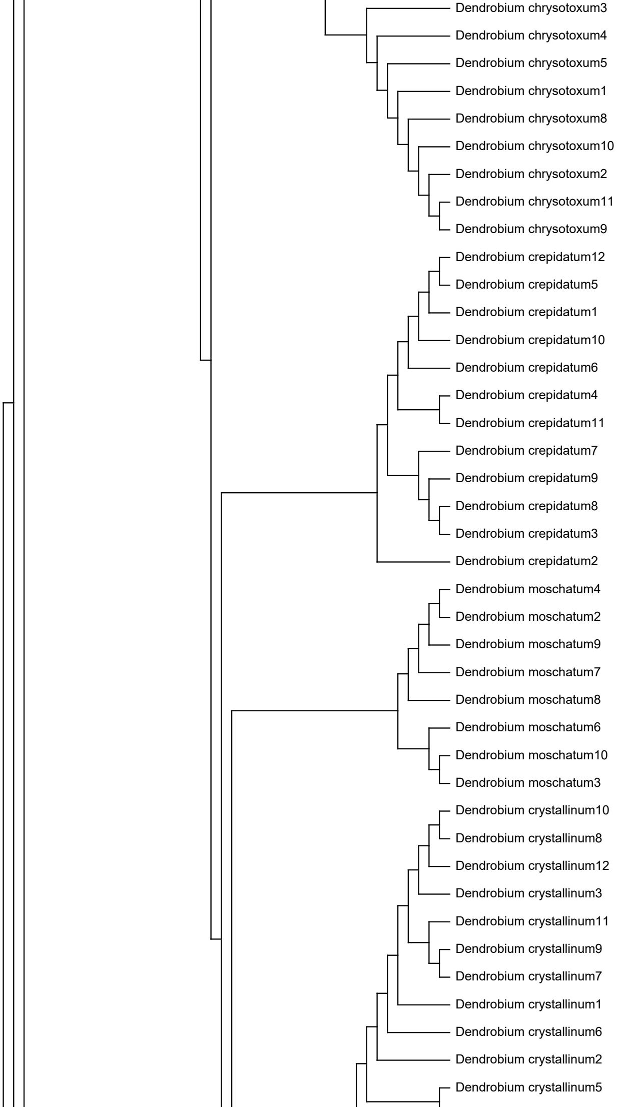

Dendrobium chrysotoxum3
Dendrobium chrysotoxum4
Dendrobium chrysotoxum5
Dendrobium chrysotoxum1
Dendrobium chrysotoxum8
Dendrobium chrysotoxum10
Dendrobium chrysotoxum2
Dendrobium chrysotoxum11
Dendrobium chrysotoxum9
Dendrobium crepidatum12
Dendrobium crepidatum5
Dendrobium crepidatum1
Dendrobium crepidatum10
Dendrobium crepidatum6
Dendrobium crepidatum4
Dendrobium crepidatum11
Dendrobium crepidatum7
Dendrobium crepidatum9
Dendrobium crepidatum8
Dendrobium crepidatum3
Dendrobium crepidatum2
Dendrobium moschatum4
Dendrobium moschatum2
Dendrobium moschatum9
Dendrobium moschatum7
Dendrobium moschatum8
Dendrobium moschatum6
Dendrobium moschatum10
Dendrobium moschatum3
Dendrobium crystallinum10
Dendrobium crystallinum8
Dendrobium crystallinum12
Dendrobium crystallinum3
Dendrobium crystallinum11
Dendrobium crystallinum9
Dendrobium crystallinum7
Dendrobium crystallinum1
Dendrobium crystallinum6
Dendrobium crystallinum2
Dendrobium crystallinum5

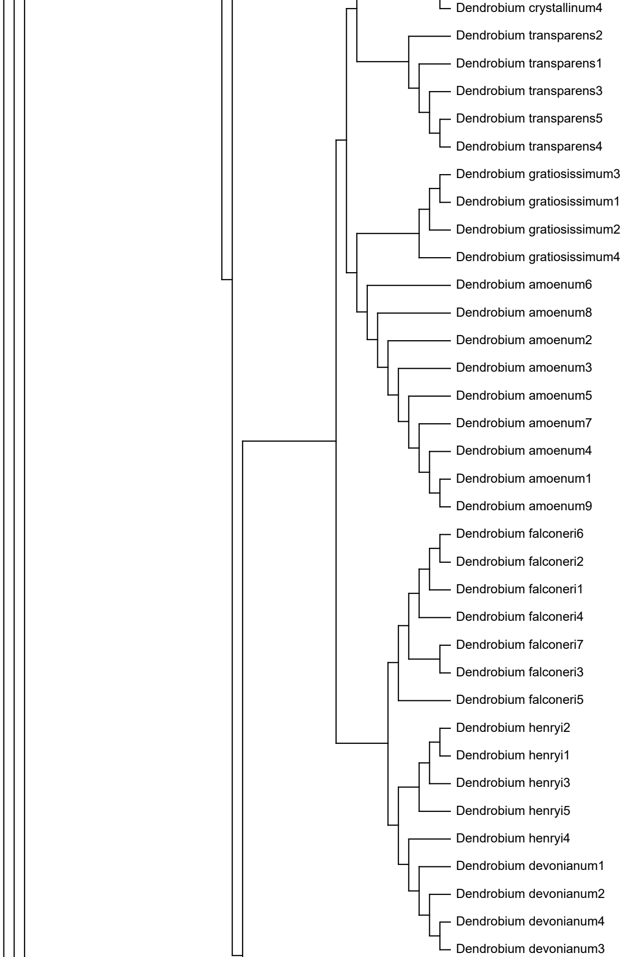

Dendrobium crystallinum4
Dendrobium transparens2
Dendrobium transparens1
Dendrobium transparens3
Dendrobium transparens5
Dendrobium transparens4
Dendrobium gratiosissimum3
Dendrobium gratiosissimum1
Dendrobium gratiosissimum2
Dendrobium gratiosissimum4
Dendrobium amoenum6
Dendrobium amoenum8
Dendrobium amoenum2
Dendrobium amoenum3
Dendrobium amoenum5
Dendrobium amoenum7
Dendrobium amoenum4
Dendrobium amoenum1
Dendrobium amoenum9
Dendrobium falconeri6
Dendrobium falconeri2
Dendrobium falconeri1
Dendrobium falconeri4
Dendrobium falconeri7
Dendrobium falconeri3
Dendrobium falconeri5
Dendrobium henryi2
Dendrobium henryi1
Dendrobium henryi3
Dendrobium henryi5
Dendrobium henryi4
Dendrobium devonianum1
Dendrobium devonianum2
Dendrobium devonianum4
Dendrobium devonianum3

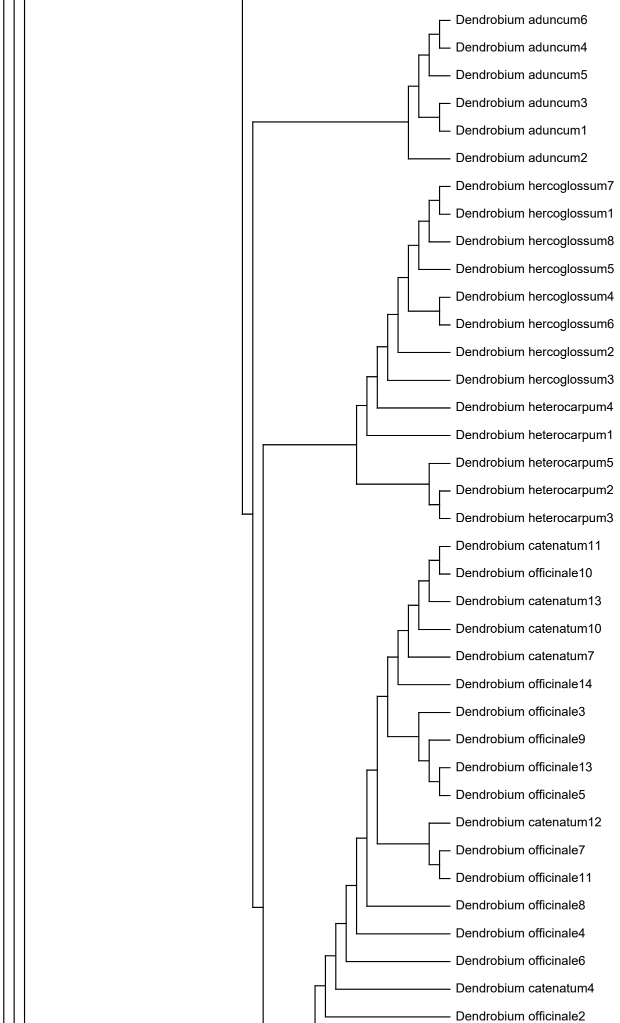
Dendrobium aduncum6
Dendrobium aduncum4
Dendrobium aduncum5
Dendrobium aduncum3
Dendrobium aduncum1
Dendrobium aduncum2
Dendrobium hercoglossum7
Dendrobium hercoglossum1
Dendrobium hercoglossum8
Dendrobium hercoglossum5
Dendrobium hercoglossum4
Dendrobium hercoglossum6
Dendrobium hercoglossum2
Dendrobium hercoglossum3
Dendrobium heterocarpum4
Dendrobium heterocarpum1
Dendrobium heterocarpum5
Dendrobium heterocarpum2
Dendrobium heterocarpum3
Dendrobium catenatum11
Dendrobium officinale10
Dendrobium catenatum13
Dendrobium catenatum10
Dendrobium catenatum7
Dendrobium officinale14
Dendrobium officinale3
Dendrobium officinale9
Dendrobium officinale13
Dendrobium officinale5
Dendrobium catenatum12
Dendrobium officinale7
Dendrobium officinale11
Dendrobium officinale8
Dendrobium officinale4
Dendrobium officinale6
Dendrobium catenatum4
Dendrobium officinale2

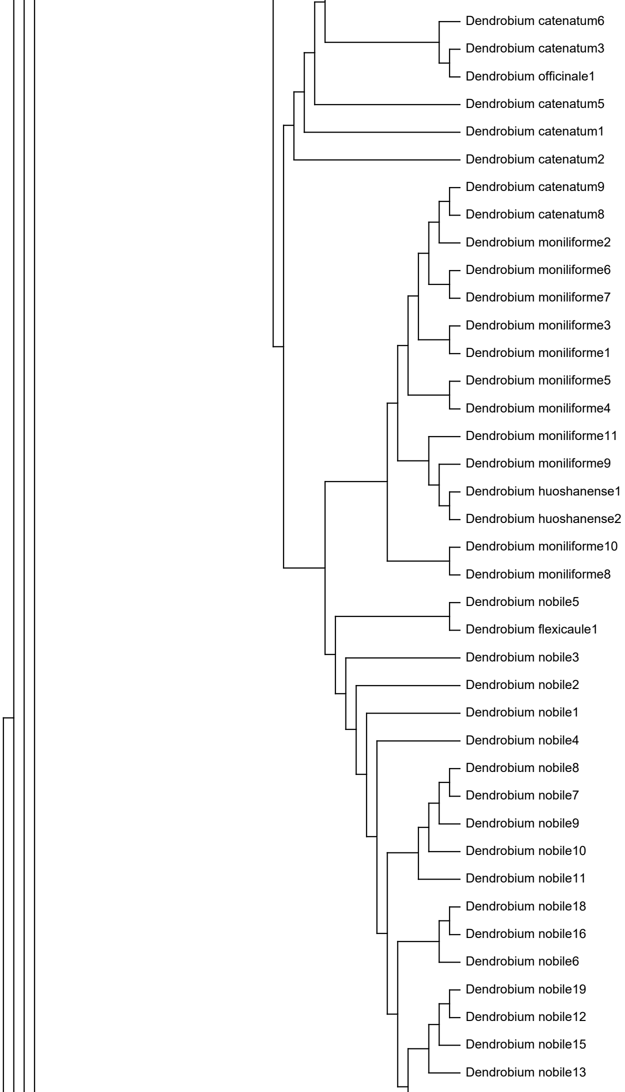
Dendrobium catenatum6
Dendrobium catenatum3
Dendrobium officinale1
Dendrobium catenatum5
Dendrobium catenatum1
Dendrobium catenatum2
Dendrobium catenatum9
Dendrobium catenatum8
Dendrobium moniliforme2
Dendrobium moniliforme6
Dendrobium moniliforme7
Dendrobium moniliforme3
Dendrobium moniliforme1
Dendrobium moniliforme5
Dendrobium moniliforme4
Dendrobium moniliforme11
Dendrobium moniliforme9
Dendrobium huoshanense1
Dendrobium huoshanense2
Dendrobium moniliforme10
Dendrobium moniliforme8
Dendrobium nobile5
Dendrobium flexicaule1
Dendrobium nobile3
Dendrobium nobile2
Dendrobium nobile1
Dendrobium nobile4
Dendrobium nobile8
Dendrobium nobile7
Dendrobium nobile9
Dendrobium nobile10
Dendrobium nobile11
Dendrobium nobile18
Dendrobium nobile16
Dendrobium nobile6
Dendrobium nobile19
Dendrobium nobile12
Dendrobium nobile15
Dendrobium nobile13

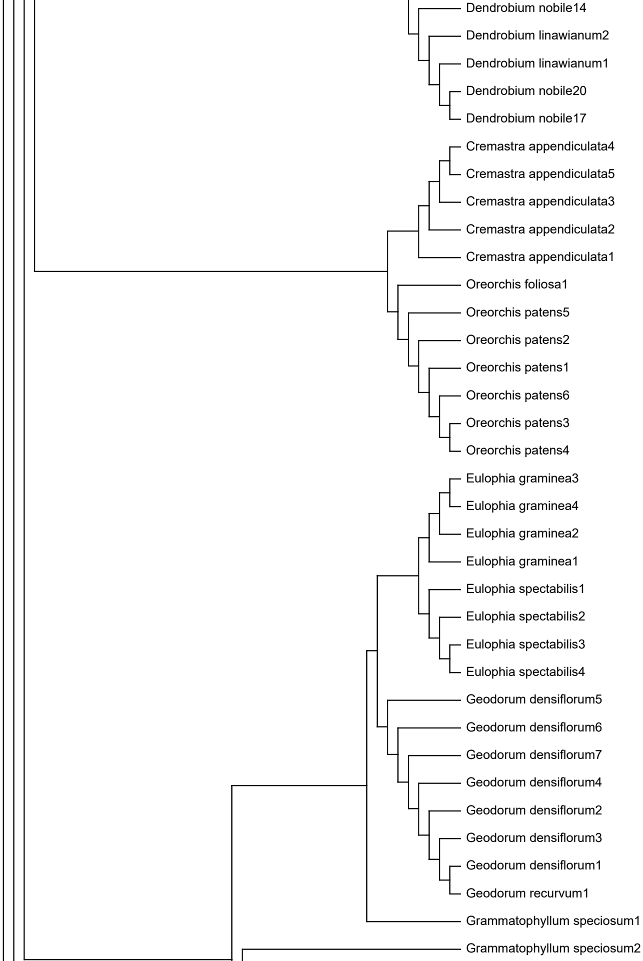
Dendrobium nobile14
Dendrobium linawianum2
Dendrobium linawianum1
Dendrobium nobile20
Dendrobium nobile17
Cremastra appendiculata4
Cremastra appendiculata5
Cremastra appendiculata3
Cremastra appendiculata2
Cremastra appendiculata1
Oreorchis foliosa1
Oreorchis patens5
Oreorchis patens2
Oreorchis patens1
Oreorchis patens6
Oreorchis patens3
Oreorchis patens4
Eulophia graminea3
Eulophia graminea4
Eulophia graminea2
Eulophia graminea1
Eulophia spectabilis1
Eulophia spectabilis2
Eulophia spectabilis3
Eulophia spectabilis4
Geodorum densiflorum5
Geodorum densiflorum6
Geodorum densiflorum7
Geodorum densiflorum4
Geodorum densiflorum2
Geodorum densiflorum3
Geodorum densiflorum1
Geodorum recurvum1
Grammatophyllum speciosum1
Grammatophyllum speciosum2

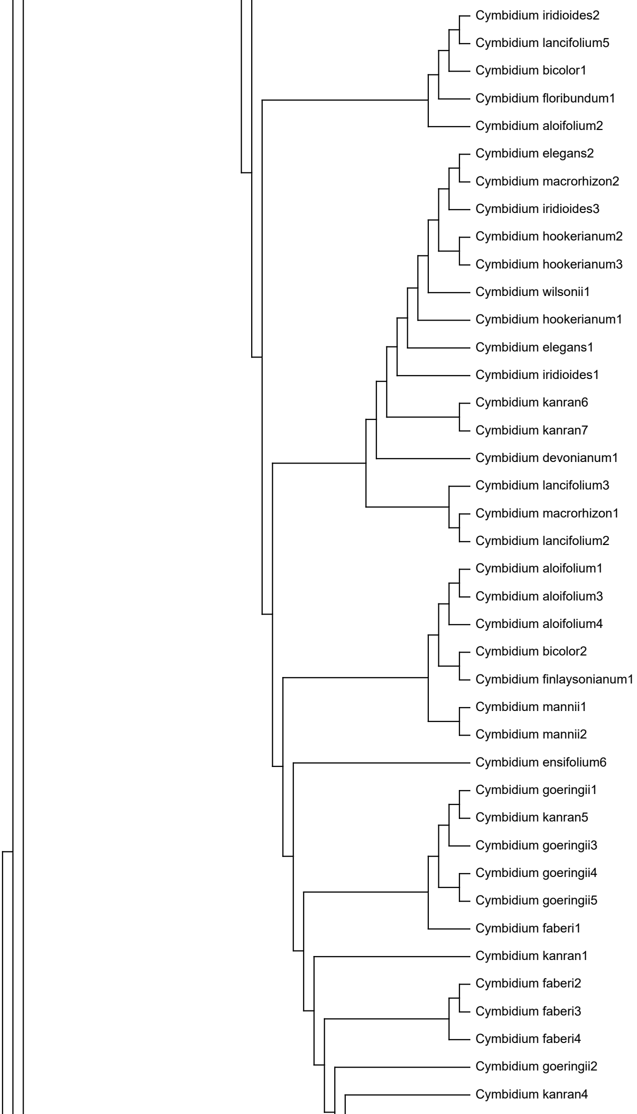
Cymbidium iridioides2
Cymbidium lancifolium5
Cymbidium bicolor1
Cymbidium floribundum1
Cymbidium aloifolium2
Cymbidium elegans2
Cymbidium macrorhizon2
Cymbidium iridioides3
Cymbidium hookerianum2
Cymbidium hookerianum3
Cymbidium wilsonii1
Cymbidium hookerianum1
Cymbidium elegans1
Cymbidium iridioides1
Cymbidium kanran6
Cymbidium kanran7
Cymbidium devonianum1
Cymbidium lancifolium3
Cymbidium macrorhizon1
Cymbidium lancifolium2
Cymbidium aloifolium1
Cymbidium aloifolium3
Cymbidium aloifolium4
Cymbidium bicolor2
Cymbidium finlaysonianum1
Cymbidium mannii1
Cymbidium mannii2
Cymbidium ensifolium6
Cymbidium goeringii1
Cymbidium kanran5
Cymbidium goeringii3
Cymbidium goeringii4
Cymbidium goeringii5
Cymbidium faberi1
Cymbidium kanran1
Cymbidium faberi2
Cymbidium faberi3
Cymbidium faberi4
Cymbidium goeringii2
Cymbidium kanran4

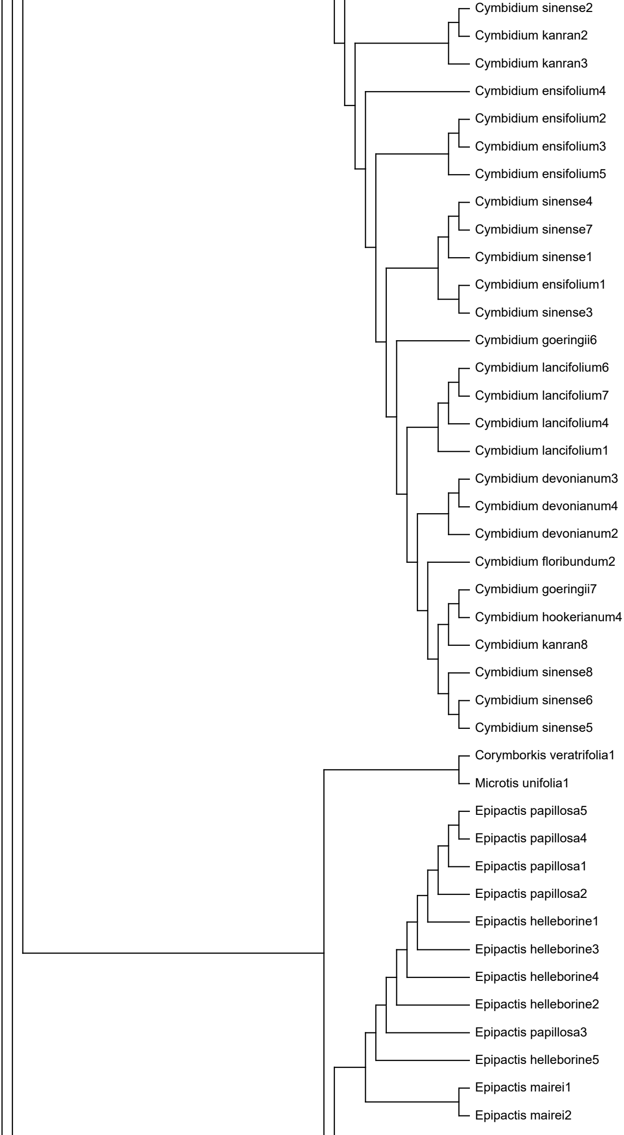
Cymbidium sinense2
Cymbidium kanran2
Cymbidium kanran3
Cymbidium ensifolium4
Cymbidium ensifolium2
Cymbidium ensifolium3
Cymbidium ensifolium5
Cymbidium sinense4
Cymbidium sinense7
Cymbidium sinense1
Cymbidium ensifolium1
Cymbidium sinense3
Cymbidium goeringii6
Cymbidium lancifolium6
Cymbidium lancifolium7
Cymbidium lancifolium4
Cymbidium lancifolium1
Cymbidium devonianum3
Cymbidium devonianum4
Cymbidium devonianum2
Cymbidium floribundum2
Cymbidium goeringii7
Cymbidium hookerianum4
Cymbidium kanran8
Cymbidium sinense8
Cymbidium sinense6
Cymbidium sinense5
Corymborkis veratrifolia1
Microtis unifolia1
Epipactis papillosa5
Epipactis papillosa4
Epipactis papillosa1
Epipactis papillosa2
Epipactis helleborine1
Epipactis helleborine3
Epipactis helleborine4
Epipactis helleborine2
Epipactis papillosa3
Epipactis helleborine5
Epipactis mairei1
Epipactis mairei2

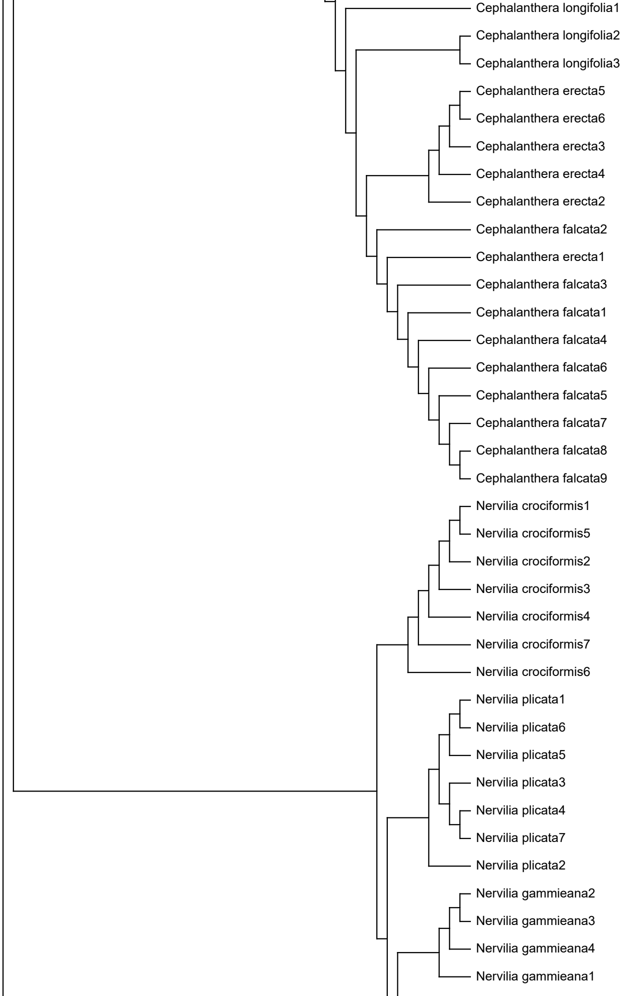
Cephalanthera longifolia1
Cephalanthera longifolia2
Cephalanthera longifolia3
Cephalanthera erecta5
Cephalanthera erecta6
Cephalanthera erecta3
Cephalanthera erecta4
Cephalanthera erecta2
Cephalanthera falcata2
Cephalanthera erecta1
Cephalanthera falcata3
Cephalanthera falcata1
Cephalanthera falcata4
Cephalanthera falcata6
Cephalanthera falcata5
Cephalanthera falcata7
Cephalanthera falcata8
Cephalanthera falcata9
Nervilia crociformis1
Nervilia crociformis5
Nervilia crociformis2
Nervilia crociformis3
Nervilia crociformis4
Nervilia crociformis7
Nervilia crociformis6
Nervilia plicata1
Nervilia plicata6
Nervilia plicata5
Nervilia plicata3
Nervilia plicata4
Nervilia plicata7
Nervilia plicata2
Nervilia gammieana2
Nervilia gammieana3
Nervilia gammieana4
Nervilia gammieana1

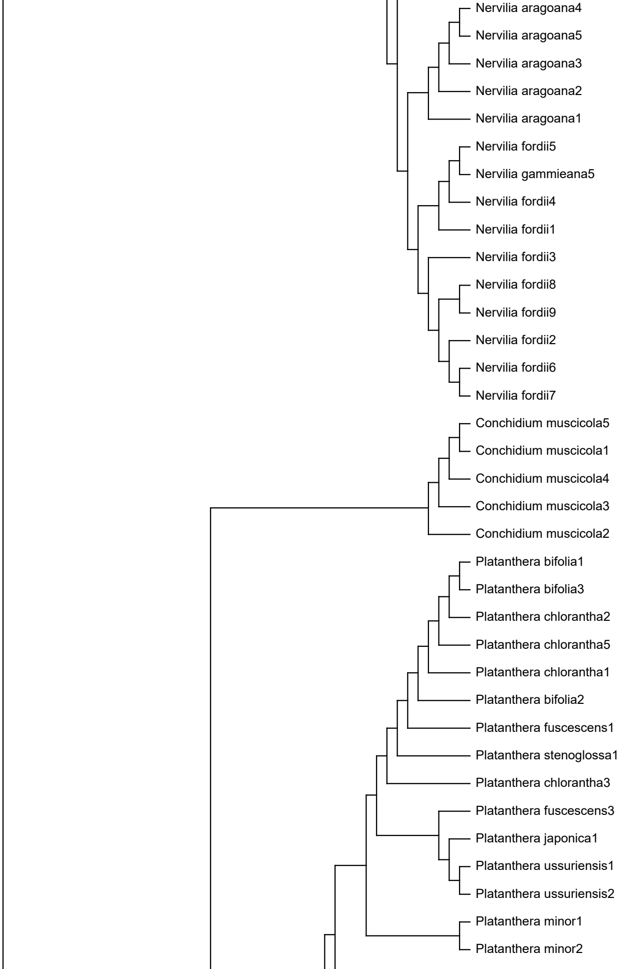
Nervilia aragoana4
Nervilia aragoana5
Nervilia aragoana3
Nervilia aragoana2
Nervilia aragoana1
Nervilia fordii5
Nervilia gammieana5
Nervilia fordii4
Nervilia fordii1
Nervilia fordii3
Nervilia fordii8
Nervilia fordii9
Nervilia fordii2
Nervilia fordii6
Nervilia fordii7
Conchidium muscicola5
Conchidium muscicola1
Conchidium muscicola4
Conchidium muscicola3
Conchidium muscicola2
Platanthera bifolia1
Platanthera bifolia3
Platanthera chlorantha2
Platanthera chlorantha5
Platanthera chlorantha1
Platanthera bifolia2
Platanthera fuscescens1
Platanthera stenoglossa1
Platanthera chlorantha3
Platanthera fuscescens3
Platanthera japonica1
Platanthera ussuriensis1
Platanthera ussuriensis2
Platanthera minor1
Platanthera minor2

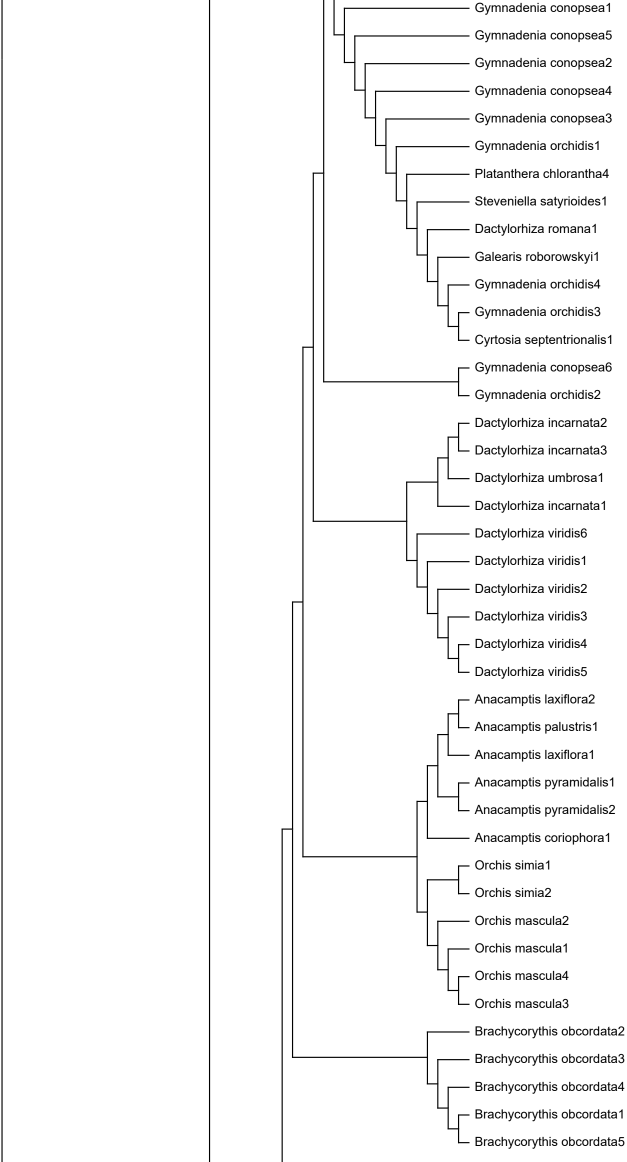

Gymnadenia conopsea1
Gymnadenia conopsea5
Gymnadenia conopsea2
Gymnadenia conopsea4
Gymnadenia conopsea3
Gymnadenia orchidis1
Platanthera chlorantha4
Steveniella satyrioides1
Dactylorhiza romana1
Galearis roborowskyi1
Gymnadenia orchidis4
Gymnadenia orchidis3
Cyrtosia septentrionalis1
Gymnadenia conopsea6
Gymnadenia orchidis2
Dactylorhiza incarnata2
Dactylorhiza incarnata3
Dactylorhiza umbrosa1
Dactylorhiza incarnata1
Dactylorhiza viridis6
Dactylorhiza viridis1
Dactylorhiza viridis2
Dactylorhiza viridis3
Dactylorhiza viridis4
Dactylorhiza viridis5
Anacamptis laxiflora2
Anacamptis palustris1
Anacamptis laxiflora1
Anacamptis pyramidalis1
Anacamptis pyramidalis2
Anacamptis coriophora1
Orchis simia1
Orchis simia2
Orchis mascula2
Orchis mascula1
Orchis mascula4
Orchis mascula3
Brachycorythis obcordata2
Brachycorythis obcordata3
Brachycorythis obcordata4
Brachycorythis obcordata1
Brachycorythis obcordata5

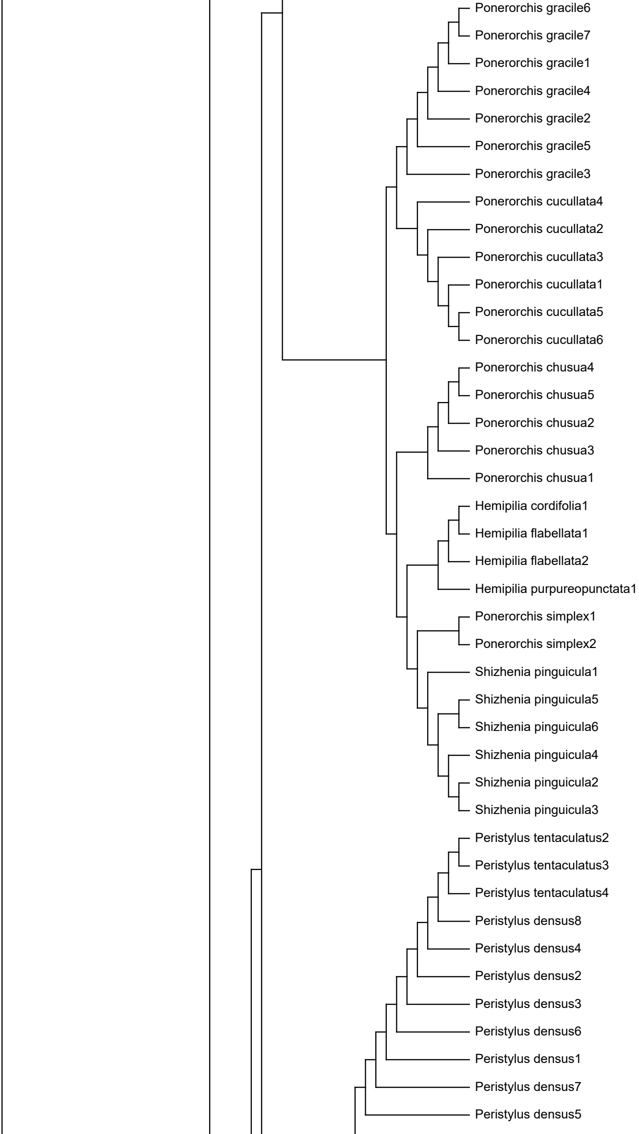
Ponerorchis gracile6
Ponerorchis gracile7
Ponerorchis gracile1
Ponerorchis gracile4
Ponerorchis gracile2
Ponerorchis gracile5
Ponerorchis gracile3
Ponerorchis cucullata4
Ponerorchis cucullata2
Ponerorchis cucullata3
Ponerorchis cucullata1
Ponerorchis cucullata5
Ponerorchis cucullata6
Ponerorchis chusua4
Ponerorchis chusua5
Ponerorchis chusua2
Ponerorchis chusua3
Ponerorchis chusua1
Hemipilia cordifolia1
Hemipilia flabellata1
Hemipilia flabellata2
Hemipilia purpureopunctata1
Ponerorchis simplex1
Ponerorchis simplex2
Shizhenia pinguicula1
Shizhenia pinguicula5
Shizhenia pinguicula6
Shizhenia pinguicula4
Shizhenia pinguicula2
Shizhenia pinguicula3
Peristylus tentaculatus2
Peristylus tentaculatus3
Peristylus tentaculatus4
Peristylus densus8
Peristylus densus4
Peristylus densus2
Peristylus densus3
Peristylus densus6
Peristylus densus1
Peristylus densus7
Peristylus densus5

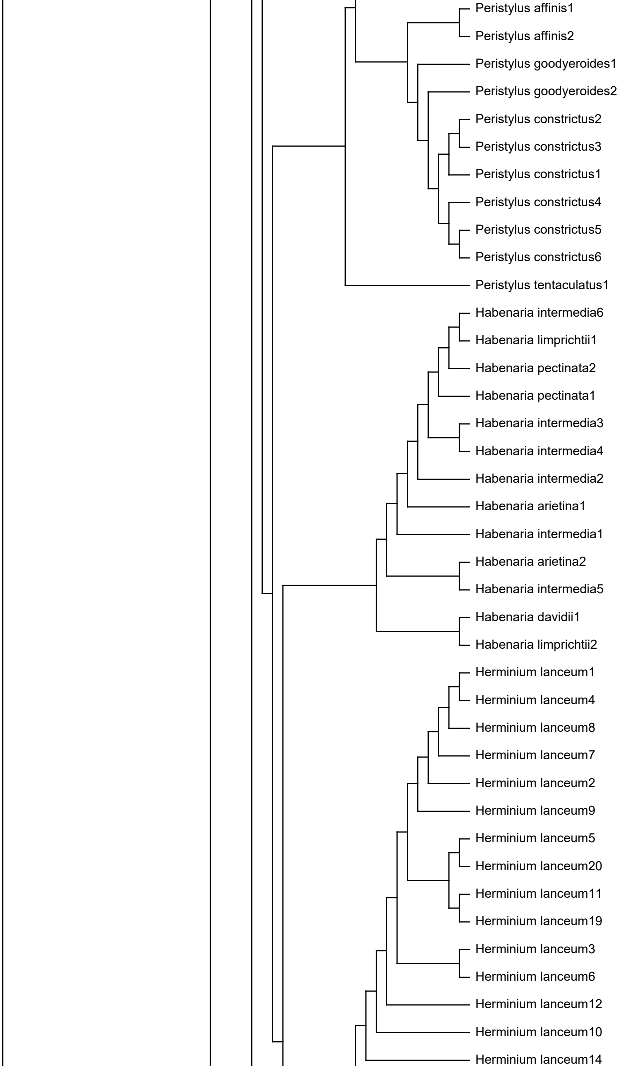

Peristylus affinis1
Peristylus affinis2
Peristylus goodyeroides1
Peristylus goodyeroides2
Peristylus constrictus2
Peristylus constrictus3
Peristylus constrictus1
Peristylus constrictus4
Peristylus constrictus5
Peristylus constrictus6
Peristylus tentaculatus1
Habenaria intermedia6
Habenaria limprichtii1
Habenaria pectinata2
Habenaria pectinata1
Habenaria intermedia3
Habenaria intermedia4
Habenaria intermedia2
Habenaria arietina1
Habenaria intermedia1
Habenaria arietina2
Habenaria intermedia5
Habenaria davidii1
Habenaria limprichtii2
Herminium lanceum1
Herminium lanceum4
Herminium lanceum8
Herminium lanceum7
Herminium lanceum2
Herminium lanceum9
Herminium lanceum5
Herminium lanceum20
Herminium lanceum11
Herminium lanceum19
Herminium lanceum3
Herminium lanceum6
Herminium lanceum12
Herminium lanceum10
Herminium lanceum14

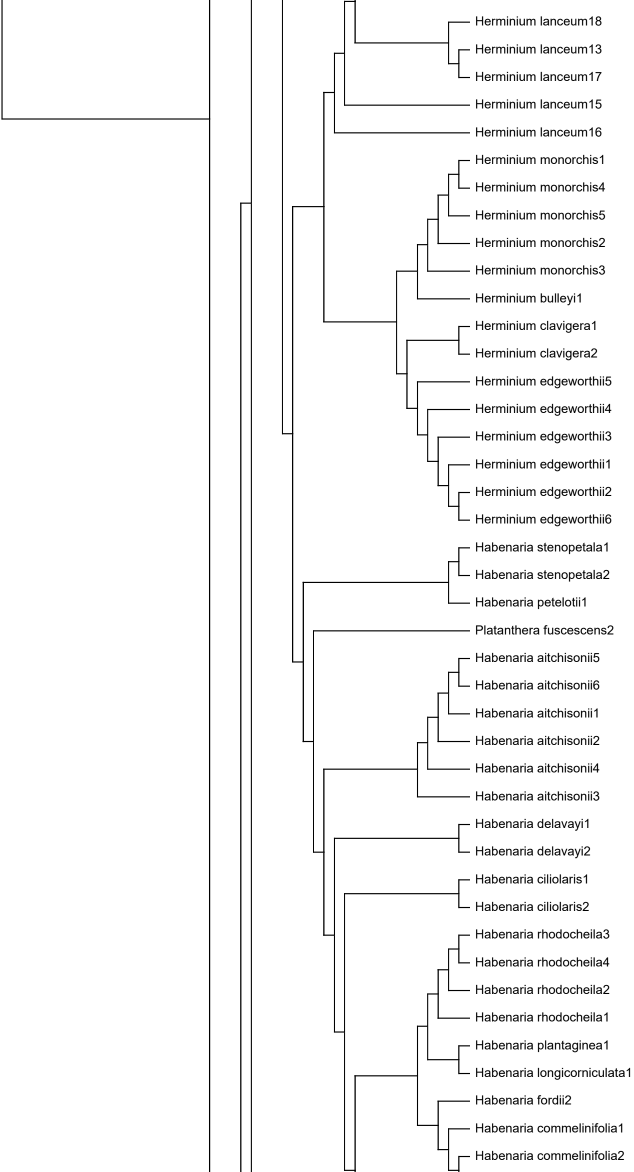
Herminium lanceum18
Herminium lanceum13
Herminium lanceum17
Herminium lanceum15
Herminium lanceum16
Herminium monorchis1
Herminium monorchis4
Herminium monorchis5
Herminium monorchis2
Herminium monorchis3
Herminium bulleyi1
Herminium clavigera1
Herminium clavigera2
Herminium edgeworthii5
Herminium edgeworthii4
Herminium edgeworthii3
Herminium edgeworthii1
Herminium edgeworthii2
Herminium edgeworthii6
Habenaria stenopetala1
Habenaria stenopetala2
Habenaria petelotii1
Platanthera fuscescens2
Habenaria aitchisonii5
Habenaria aitchisonii6
Habenaria aitchisonii1
Habenaria aitchisonii2
Habenaria aitchisonii4
Habenaria aitchisonii3
Habenaria delavayi1
Habenaria delavayi2
Habenaria ciliolaris1
Habenaria ciliolaris2
Habenaria rhodocheila3
Habenaria rhodocheila4
Habenaria rhodocheila2
Habenaria rhodocheila1
Habenaria plantaginea1
Habenaria longicorniculata1
Habenaria fordii2
Habenaria commelinifolia1
Habenaria commelinifolia2

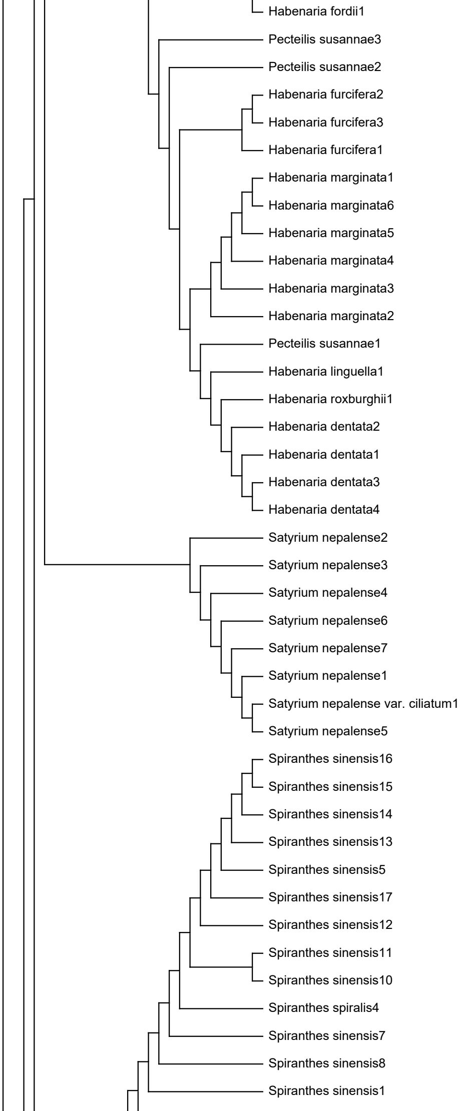

Habenaria fordii1
Pecteilis susannae3
Pecteilis susannae2
Habenaria furcifera2
Habenaria furcifera3
Habenaria furcifera1
Habenaria marginata1
Habenaria marginata6
Habenaria marginata5
Habenaria marginata4
Habenaria marginata3
Habenaria marginata2
Pecteilis susannae1
Habenaria linguella1
Habenaria roxburghii1
Habenaria dentata2
Habenaria dentata1
Habenaria dentata3
Habenaria dentata4
Satyrium nepalense2
Satyrium nepalense3
Satyrium nepalense4
Satyrium nepalense6
Satyrium nepalense7
Satyrium nepalense1
Satyrium nepalense var. ciliatum1
Satyrium nepalense5
Spiranthes sinensis16
Spiranthes sinensis15
Spiranthes sinensis14
Spiranthes sinensis13
Spiranthes sinensis5
Spiranthes sinensis17
Spiranthes sinensis12
Spiranthes sinensis11
Spiranthes sinensis10
Spiranthes spiralis4
Spiranthes sinensis7
Spiranthes sinensis8
Spiranthes sinensis1

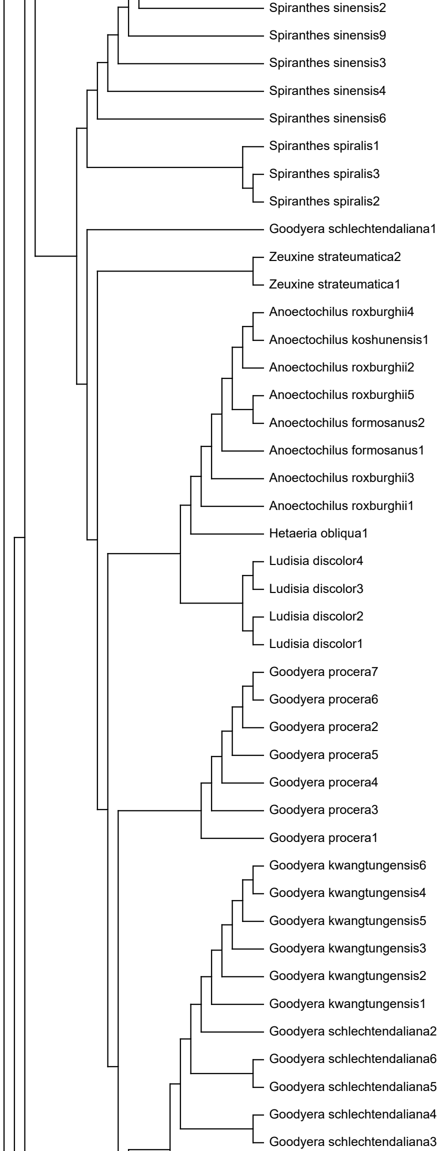
Spiranthes sinensis2
Spiranthes sinensis9
Spiranthes sinensis3
Spiranthes sinensis4
Spiranthes sinensis6
Spiranthes spiralis1
Spiranthes spiralis3
Spiranthes spiralis2
Goodyera schlechtendaliana1
Zeuxine strateumatica2
Zeuxine strateumatica1
Anoectochilus roxburghii4
Anoectochilus koshunensis1
Anoectochilus roxburghii2
Anoectochilus roxburghii5
Anoectochilus formosanus2
Anoectochilus formosanus1
Anoectochilus roxburghii3
Anoectochilus roxburghii1
Hetaeria obliqua1
Ludisia discolor4
Ludisia discolor3
Ludisia discolor2
Ludisia discolor1
Goodyera procera7
Goodyera procera6
Goodyera procera2
Goodyera procera5
Goodyera procera4
Goodyera procera3
Goodyera procera1
Goodyera kwangtungensis6
Goodyera kwangtungensis4
Goodyera kwangtungensis5
Goodyera kwangtungensis3
Goodyera kwangtungensis2
Goodyera kwangtungensis1
Goodyera schlechtendaliana2
Goodyera schlechtendaliana6
Goodyera schlechtendaliana5
Goodyera schlechtendaliana4
Goodyera schlechtendaliana3

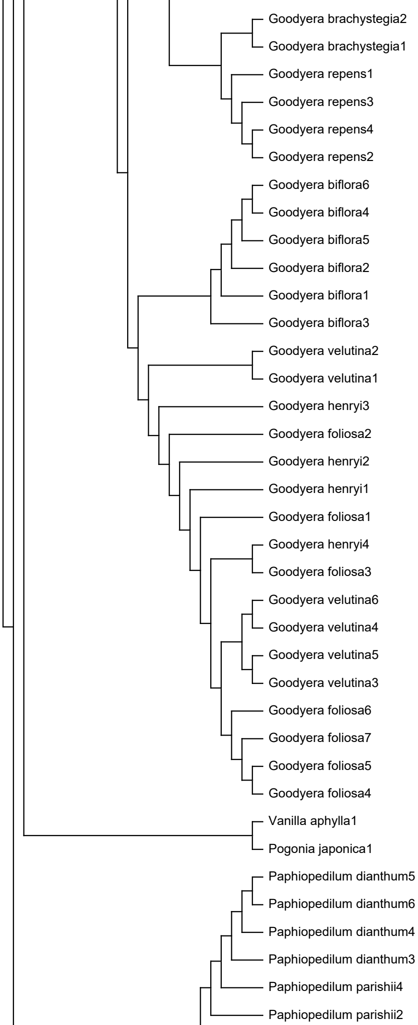
Goodyera brachystegia2
Goodyera brachystegia1
Goodyera repens1
Goodyera repens3
Goodyera repens4
Goodyera repens2
Goodyera biflora6
Goodyera biflora4
Goodyera biflora5
Goodyera biflora2
Goodyera biflora1
Goodyera biflora3
Goodyera velutina2
Goodyera velutina1
Goodyera henryi3
Goodyera foliosa2
Goodyera henryi2
Goodyera henryi1
Goodyera foliosa1
Goodyera henryi4
Goodyera foliosa3
Goodyera velutina6
Goodyera velutina4
Goodyera velutina5
Goodyera velutina3
Goodyera foliosa6
Goodyera foliosa7
Goodyera foliosa5
Goodyera foliosa4
Vanilla aphylla1
Pogonia japonica1
Paphiopedilum dianthum5
Paphiopedilum dianthum6
Paphiopedilum dianthum4
Paphiopedilum dianthum3
Paphiopedilum parishii4
Paphiopedilum parishii2

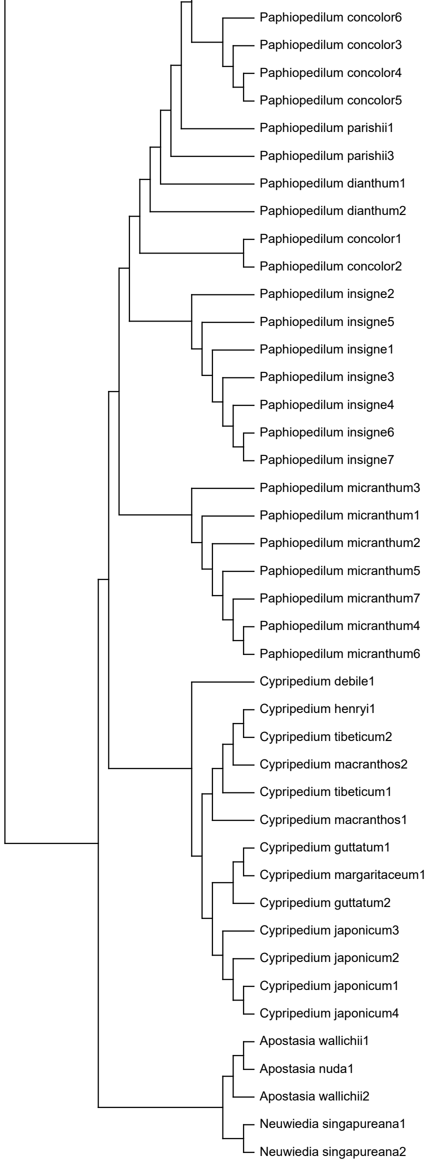
Paphiopedilum concolor6
Paphiopedilum concolor3
Paphiopedilum concolor4
Paphiopedilum concolor5
Paphiopedilum parishii1
Paphiopedilum parishii3
Paphiopedilum dianthum1
Paphiopedilum dianthum2
Paphiopedilum concolor1
Paphiopedilum concolor2
Paphiopedilum insigne2
Paphiopedilum insigne5
Paphiopedilum insigne1
Paphiopedilum insigne3
Paphiopedilum insigne4
Paphiopedilum insigne6
Paphiopedilum insigne7
Paphiopedilum micranthum3
Paphiopedilum micranthum1
Paphiopedilum micranthum2
Paphiopedilum micranthum5
Paphiopedilum micranthum7
Paphiopedilum micranthum4
Paphiopedilum micranthum6
Cypripedium debile1
Cypripedium henryi1
Cypripedium tibeticum2
Cypripedium macranthos2
Cypripedium tibeticum1
Cypripedium macranthos1
Cypripedium guttatum1
Cypripedium margaritaceum1
Cypripedium guttatum2
Cypripedium japonicum3
Cypripedium japonicum2
Cypripedium japonicum1
Cypripedium japonicum4
Apostasia wallichii1
Apostasia nuda1
Apostasia wallichii2
Neuwiedia singapureana1
Neuwiedia singapureana2
